# Red-light-mediated Barton decarboxylation reaction and one-pot wavelength-selective transformations†

**DOI:** 10.1039/d3sc03643j

**Published:** 2023-09-28

**Authors:** Hiroki Yamamoto, Kohei Yamaoka, Ann Shinohara, Kouhei Shibata, Ken-ichi Takao, Akihiro Ogura

**Affiliations:** a Department of Applied Chemistry, Keio University Hiyoshi, Kohoku-ku Yokohama 223-8522 Japan takao@applc.keio.ac.jp ogura@applc.keio.ac.jp

## Abstract

In organic chemistry, selecting mild conditions for transformations and saving energy are increasingly important for achieving sustainable development goals. Herein, we describe a red-light-mediated Barton decarboxylation using readily available red-light-emitting diodes as the energy source and zinc tetraphenylporphyrin as the catalyst, avoiding explosive or hazardous reagents or external heating. Mechanistic studies suggest that the reaction probably proceeds *via* Dexter energy transfer between the activated catalyst and the Barton ester. Furthermore, a one-pot wavelength-selective reaction within the visible light range is developed in combination with a blue-light-mediated photoredox reaction, demonstrating the compatibility of two photochemical transformations based on mechanistic differences. This one-pot process expands the limits of the decarboxylative Giese reaction beyond polarity matching.

## Abstract 2

Red-light-mediated Barton decarboxylation using catalytic zinc tetraphenylporphyrin was developed. One-pot wavelength-selective reaction within the visible light range was achieved in combination with blue-light-mediated photoredox reaction.

## Graphical abstract

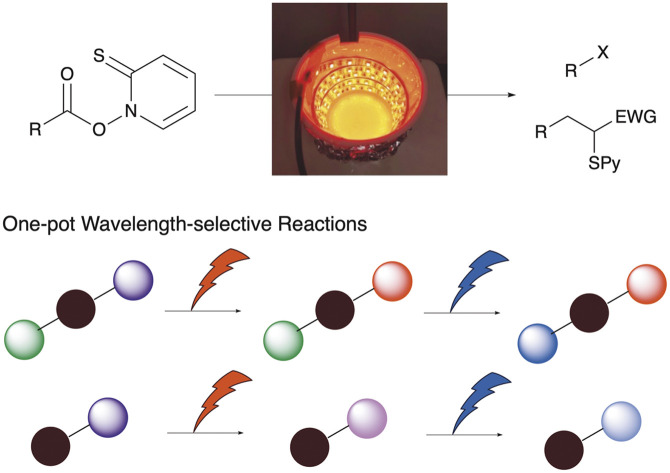

## Introduction

Visible-light-mediated organic transformations have progressed enormously in the past decade. In particular, photoredox chemistry has attracted widespread attention and various reactions have been developed.^1–9^ Photoredox chemistry depends on the electrochemical potential of the catalyst activated by light energy. Blue light or white light containing short wavelengths has generally been used for activation because the high energy of blue light (450 nm: 266 kJ mol^−1^) can easily meet the electron potential required for reactions to occur. However, blue light-emitting diode (LED) light sources need higher electric voltages compared with those with longer wavelengths, and there are also concerns about adverse health effects, for example, on circadian rhythms^10^ and the retina.^11^ Furthermore, the low penetration rate of short wavelength-light can cause problems, especially in scaling up reactions.

In this context, red light has started to gather attention from organic chemists as a source of energy for organic reactions because of its safety. In addition to widely used singlet oxygen generation,^12–16^ red-light-mediated organic transformation involving the photoredox mechanism or intramolecular charge transfer have been reported.^17–33^ Very recently, we reported the red-light-mediated Barton–McCombie reaction.^34^ In the presence of chlorophyll a as the catalyst^35^ and tris(trimethylsilyl)silane or a Hantzsch ester as the hydrogen source, the methyl xanthate moiety was removed under red-light irradiation. The reaction mechanism was probably *via* the formation of a complex between the substrate and the photocatalyst, followed by charge transfer.^36^ The new conditions were safer and milder than the original conditions.^37^

Based on this previous work, we wondered whether wavelength-selective activation of a molecule could be achieved through sequential, preferably one-pot, visible-light irradiation. The narrow emission wavelength band of LEDs would be beneficial for activating the catalyst selectively, and thus we expected new chemical selectivity would be possible. Although this type of reaction selectivity has been achieved in polymer synthesis,^38–41^ its use in fine organic synthesis has been rare. Despite the recent achievement of one-pot white/blue sequential transformation,^42^ sequential chemical transformation by a specific wavelength of visible light in a one-pot reaction has not been reported. With numerous examples of blue-light-mediated redox reactions,^1–9^ we considered that to achieve selectivity, namely, that the red-light-mediated reaction should not affect the redox-active moiety, a red-light-triggered reaction *not* involving a redox mechanism would be preferable. Thus, energy transfer catalysis would be an attractive choice.^43,44^ Among the candidate reactions for exploring this idea, we decided to focus on the decarboxylative functionalization reaction under red-light irradiation because its blue light-mediated counterpart has been investigated extensively with many examples of redox reactions.^1–9^ Herein, we describe a red-light-mediated Barton decarboxylation reaction *via* an energy transfer mechanism and one-pot wavelength-selective decarboxylative functionalization reactions.

## Results and discussion

### Red-light-mediated decarboxylative reactions

First, we established the simple red-light-mediated Barton decarboxylation reaction.^45,46^ Although high-energy UV light directly excites the substrate, the red-light-mediated reaction has not been reported. Thus, model compound 1a was irradiated with 4 W red LEDs in the presence of photocatalysts and a hydrogen source (see Table S1† for summary of optimization). The optimized conditions used 4 equiv of *t*-dodecanethiol as the hydrogen source, 0.1 mol% zinc tetraphenylporphyrin (ZnTPP)^47,48^ as the catalyst, 0.2 M acetonitrile and 15 min of red-light irradiation to provide decarboxylated product 2a. These conditions do not require an explosive azo reagent, a toxic organotin reagent, or energy-consuming heat or UV sources.^49,50^ Use of *t*-dodecanethiol is preferable, because unlike low molecular weight thiols, *t*-dodecanethiol is regarded as a low-odour reagent. Also, reaction time was much shorter than the direct excitation in the original study.^45,46^ Shorter irradiation times (15 min) and lower voltages than the conventional blue-light-mediated reaction (several hours to days) also save energy.

Having optimized the reaction conditions, we investigated the substrate scope. Various Barton esters were irradiated with red LEDs in the presence of ZnTPP (Table 1). A range of hydroxy protecting groups were tolerated (2b to 2g), including a UV-labile *o*-nitrobenzyl group (2h).^51,52^ Terminal olefin (2i) and phenylpropiolate (2j) moieties were also compatible, and products *via* tertiary (2l) or benzyl radicals (2m) were obtained in moderate yields. Notably redox-active phthalimide esters^53–55^ were stable under the conditions (2n to 2p). A wide variety of biology-related carboxylic acids were also smoothly converted to the corresponding decarboxylated products with an occasional slight modification (2q to 2u). Barton ester derivatives from biotin and amino acids were unstable to silica gel purification,^56^ but the one-pot conversion from the carboxylic acid afforded decarboxybiotin and amines (2v to 2y). In contrast, substrates that generated certain benzyl or phenyl radicals gave unsatisfactory results (1z to 1ac); the high electronic stability of benzyl radicals with an electron-donating group lead to low hydrogen atom transfer reactivity against thiol, which resulted in side reactions, such as homodimerization or ketone formation.^57^ For benzoate, hydrogenation with thiol occurred before decarboxylation at ambient temperature.^58^

**Table 1:** Substrate scope of red-light-mediated Barton decarboxylation

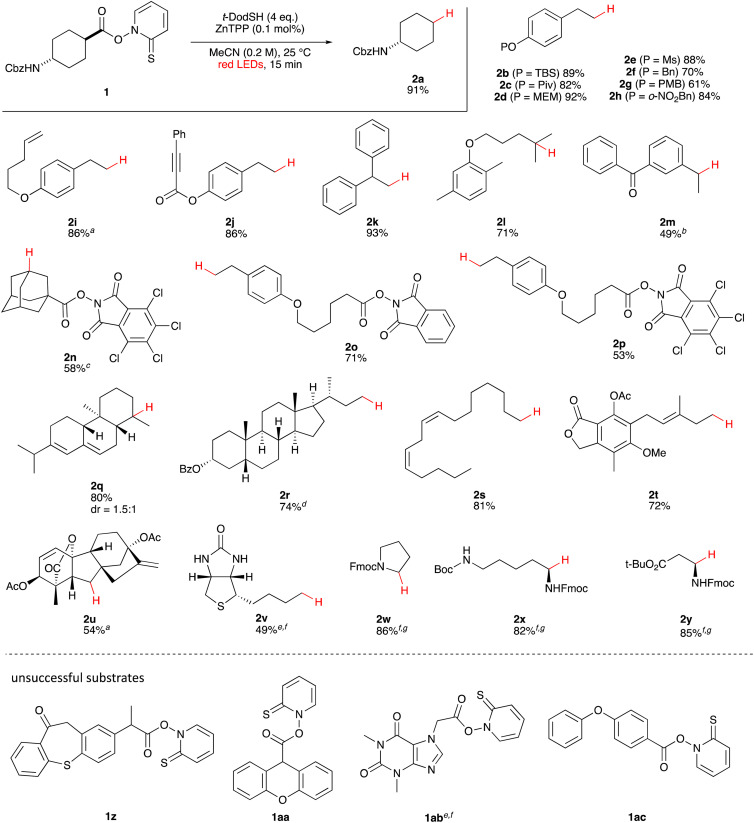

a 60 min with 0.5 mol% ZnTPP.

b 16 h.

c 0.05 M in CH_2_Cl_2_.

d 30 min in benzene.

e 15 min in DMF.

f One pot from corresponding carboxylic acid.

g 15 min in DMF.

We then investigated decarboxylative functionalization reactions.^59^ Model Barton ester 3 was irradiated with red LEDs in the presence of ZnTPP and various reagents (Table 2, see Tables S2 to S10† for optimization). Halogenation (4a–4c),^60^ oxygenation (4d),^61^ nitrogenation (4e^62^ and 4f^63,64^), sulfidation (4g),^65,66^ selenidation (4h),^65,66^ and borylation (4i)^67,68^ all worked well, and afforded the corresponding functionalized products in good yields. However, decarboxylative fluorination^69^ was not possible due to the incompatibility of Barton esters with fluorine sources.

**Table 2:** Red-light-mediated Barton decarboxylative functionalization

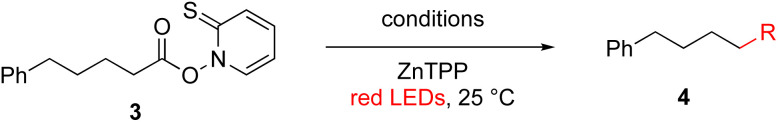				
Entry	R	Product	Conditions	Yield
1	Cl	4a	Cl_3_CCCl_3_	73%
2	Br	4b	CBrCl_3_, toluene	55%
3	I	4c	CH_2_I_2_, toluene	70%
4	OH	4d	O_2_, *t*-BuSH, EtOH then P(OMe)_3_	75%
5	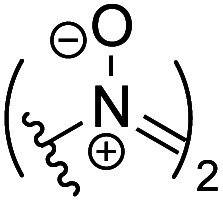	4e	Ph_3_CSNO, CH_2_Cl_2_/toluene	61%
6	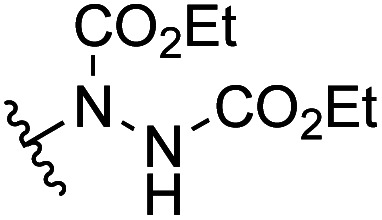	4f	DEAD, TTMSS, toluene	57%
7	SPh	4g	PhSSPh, DMSO	84%
8	SePh	4h	PhSeSePh, CH_2_Cl_2_	91%
9	Bpin	4i	B_2_cat_2_, DMF then pinacol, Et_3_N	67%

Another important reaction using a decarboxylated radical intermediate is the Giese reaction, which is a radical-mediated conjugate addition.^70–72^ The decarboxylative Giese reaction has been a focus of research^64,73–77^ because it enables convenient chemical bond recombination beyond the classical synthon approach. Thus, Barton ester 5 was treated with various unsaturated carbonyl compounds (6) in the presence of ZnTPP and red-light irradiation (Table 3, see Table S11† for optimization). Acrylates (entries 1 and 2), vinyl ketone (entry 3), and acrylonitrile (entry 4) all afforded corresponding conjugate addition products 7 in moderate to good yields, with pyridyl sulfide 8 as a minor product. The highest yield was obtained with a simple acrylic acid as the radical acceptor (entry 5), whereas the redox-active phthalimidyl ester only gave a small amount of 8 (entry 6). Substitution generally decreased the yield.^70^ Methyl methacrylate afforded the Giese product in low yield (entry 7), and crotonate did not provide 7 (entry 8). Extra addition of various Lewis acids^78^ did not improve the yields. Whereas fumarate and maleate with a lower LUMO afforded 7 in moderate yield (entries 9 and 10), cyclic ketones gave 8 as the major product (entries 11 and 12).

**Table 3:** Substrate scope of red-light-mediated Barton decarboxylative Giese reaction

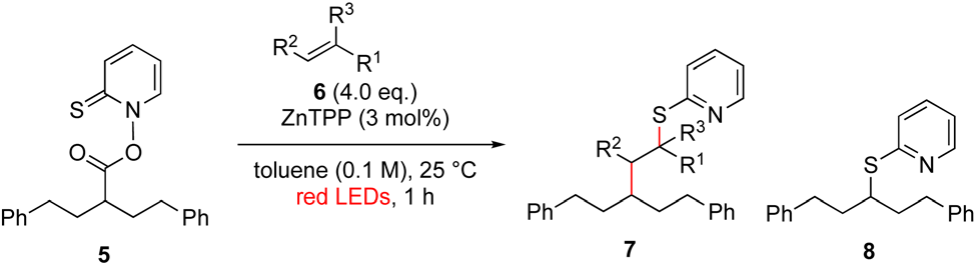							
Entry	Reagent		R^1^	R^2^	R^3^	Yield	
						7	8
1	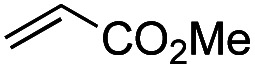	6a	CO_2_Me	H	H	67	20
2	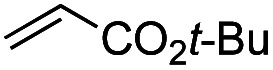	6b	CO_2_*t*-Bu	H	H	50	22
3	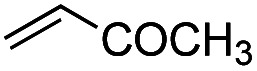	6c	COCH_3_	H	H	65	21
4	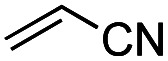	6d	CN	H	H	55	17
5	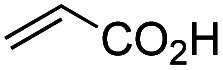	6e	CO_2_H	H	H	78	18
6	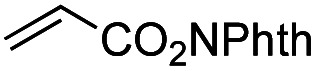	6f	CO_2_NPhth	H	H	ND	13
7	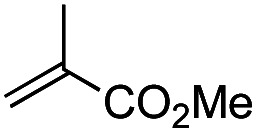	6g	CO_2_Me	H	Me	35	37
8	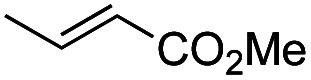	6h	CO_2_Me	Me	H	ND	65
9	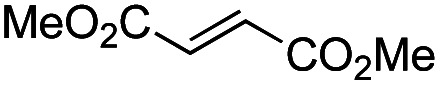	6i	CO_2_Me	CO_2_Me	H	50^a^	17
10	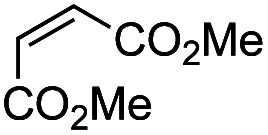	6j	CO_2_Me	CO_2_Me	H	30^a^	66
11	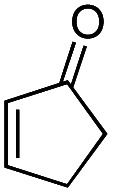	6k	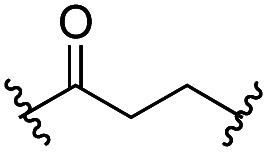		H	ND	54
12	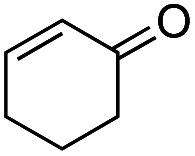	6l	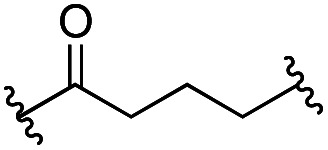		H	ND	68

a d.r. = 5 : 1.

### One-pot wavelength-selective transformations

**Scheme 1:**
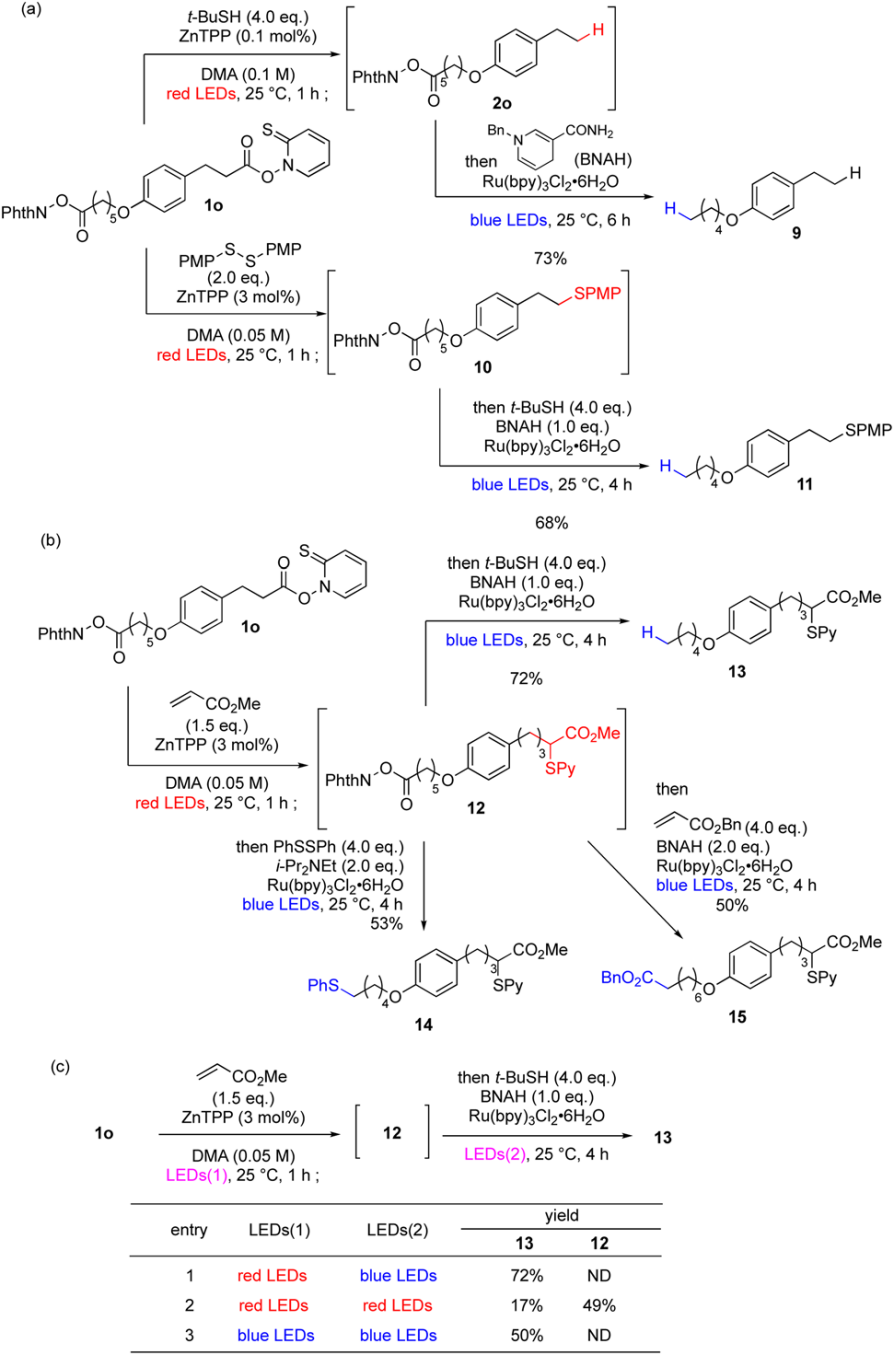
Parallel wavelength-selective sequential one-pot transformations. (a) One-pot decarboxylation–decarboxylation using red and blue light, respectively. (b) One-pot decarboxylative Giese reaction-decarboxylation using red and blue light, respectively. (c) Control experiments using red light only or blue light only.

Having established red-light-mediated transformations, we moved on to wavelength-selective sequential transformations (Scheme 1a). First, we performed a *parallel* reaction, in which one molecule with two photoactivated functional groups was transformed sequentially in a wavelength-selective one-pot reaction. To differentiate the two photoreactive functional groups, 1o was selected as the substrate, which carries a red-light-reactive Barton ester moiety and a blue-light-reactive phthalimidyl ester moiety. Treatment with *t*-butyl mercaptan and ZnTPP under red-light irradiation afforded 2o as the intermediary product, as in Table 1. Subsequently, 1-benzyl-1,4-dihydronicotinamide (BNAH) and a ruthenium complex were added to the reaction mixture and it was irradiated by blue light.^79^ Photoredox decarboxylation proceeded smoothly to provide 9 in good overall yield. Similarly, red-light-mediated decarboxylative sulfidation provided intermediate 10, which upon blue-light-mediated decarboxylation afforded 11 in good overall yield. Importantly, the presence of ZnTPP did not affect the reactivity of the ruthenium catalyst, and the bisulfide was not observed. Next, 1o was subjected to red-light-mediated Giese reaction with methyl acrylate to afford intermediary product 12 (Scheme 1b). One-pot photoredox decarboxylation with blue light gave expected product 13 in 72% yield. Furthermore, blue-light-mediated decarboxylative sulfidation^80^ or another Giese reaction^81^ of 12 proceeded to give 14 and 15, respectively, thus achieving chemoselective functionalization based on one-pot wavelength-selective photoactivation (more examples in Scheme S1†). To prove the wavelength dependence of these transformations, 1o was converted to 13 with different combinations of light wavelengths (Scheme 1c). Optimized red-blue irradiation afforded 13 in 72% yield (entry 1). However, red–red (entry 2) or blue–blue irradiation (entry 3) decreased the yield greatly and increased the amount of byproducts, which may be explained by insufficient activation or uncontrolled attack by the catalyst, respectively, indicating that activation of photocatalyst with appropriate wavelength is necessary for optimum performance.

In addition to the *parallel* reactions, a sequential *series* reaction was investigated (Table 4a). We define the *series* reaction as a one-pot reaction^82^ in which the photoactivated functional group is converted into another photoactivated functional group, which is then subjected to another photoreaction. Because 5 could be converted to carboxylic acid 7e by our red-light-mediated Giese reaction with acrylic acid (Table 3), optimization of the subsequent decarboxylation was first performed based on the conditions developed by MacMillan group^83^ and Nicewicz group^84^ (see Table S12† for detail). A biphasic system with the addition of tetrabutylammonium iodide as a phase transfer catalyst greatly improved the yield of this single reaction, and gave 16a in 95% yield. When the two reactions were performed as a one-pot reaction, desired product 16a was obtained in satisfactory overall yield of 67% (see Table S13† for optimization).

**Table 4:** Series wavelength-selective sequential one-pot transformations. (a) One-pot decarboxylative Giese reaction-decarboxylation using red and blue light, respectively. (b) One-pot decarboxylative Giese reaction-decarboxylative hydroxylation using red and blue light, respectively, followed by reduction

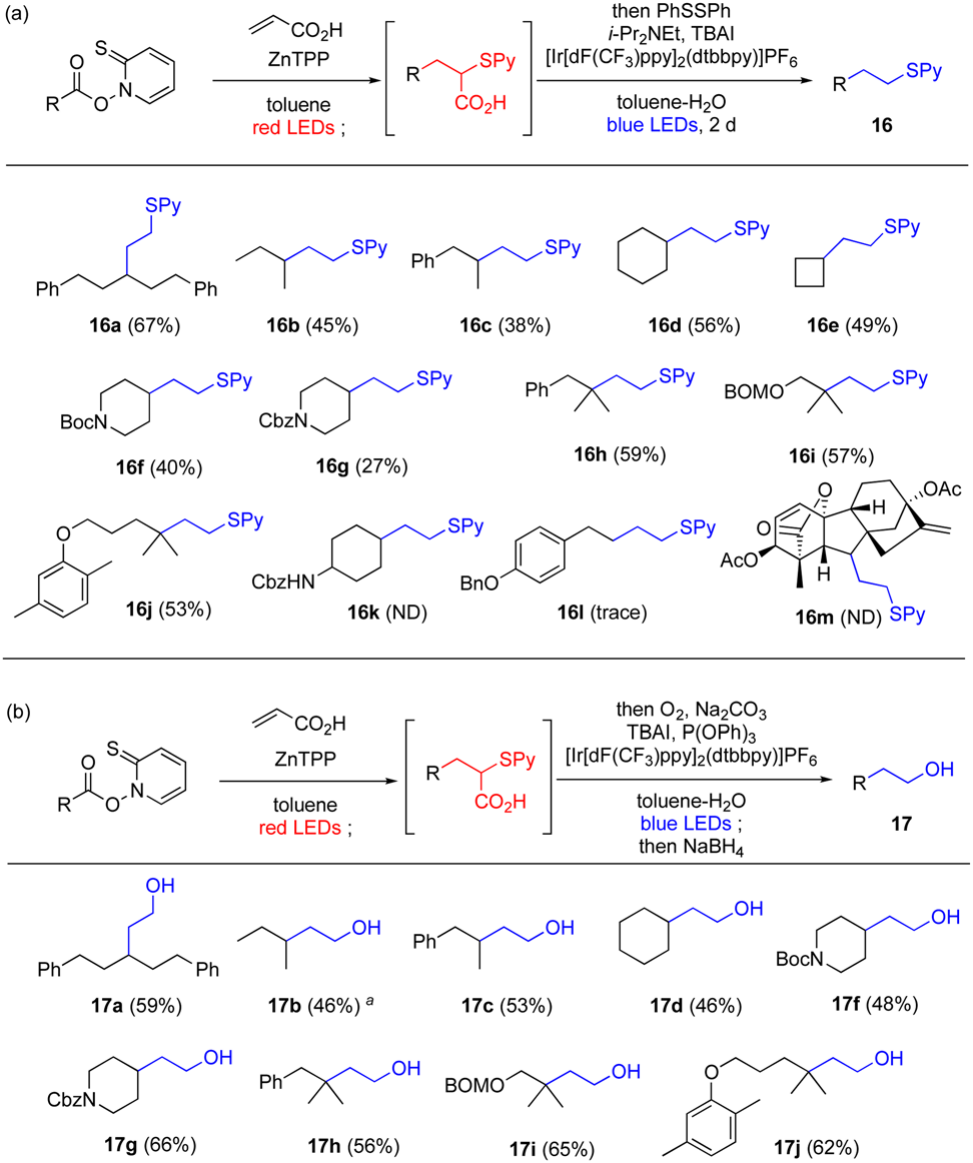

a GC yield.

The Giese reaction and decarboxylative oxygenation were chosen for a further example of a *series* reaction. First, the decarboxylative oxygenation was optimized independently (see Table S14† for optimization). Thus, 7e was subjected to conditions established by MacMillan group,^85^ and reductive treatment afforded alcohol 17a. The reaction likely involved the decarboxylative formation of the thioester,^85^ followed by additional reduction (detailed proposed mechanism in Scheme S2†).^86^ The addition of phosphine increased the yield, which suggested that phosphine-mediated heterolytic cleavage of the hydroperoxide moiety converted the peroxide to the corresponding aldehyde by expelling pyridine thiol (see Scheme S3†). This reaction can be conducted as a wavelength-selective one-pot reaction with the red-light-mediated Barton decarboxylative Giese reaction without major modification (Table 4b).

Under the conditions to give 16a and 17a described above, the substrate scope for this one-pot wavelength-selective transformation was studied (Table 4). For the Giese reaction/decarboxylation reaction, a wide variety of Barton esters that give secondary or tertiary radicals afforded the corresponding thioethers generally in medium to good yields (Table 4a, 16a–16k). However, in some cases, the complexation of the reaction system (16g) or insufficient substrate solubility (16k) led to unsatisfactory results. Substrates without α-carbon substitution (16l) or with high steric congestion (16m) did not afford the desired products because of an unproductive Giese reaction. The Giese reaction/decarboxylative oxygenation also proceeded, affording the corresponding alcohols in satisfactory yields (Table 4b, 17a–17j). It should be emphasized that the products obtained by these one-pot sequences are hard to construct *via* normal Giese reactions using vinyl sulfides or enol equivalents because they are polarity mismatched. This one-pot procedure enables polarity switching,^87^ broadening the utility of the Giese reaction.

### Mechanistic analysis

**Fig. 1:**
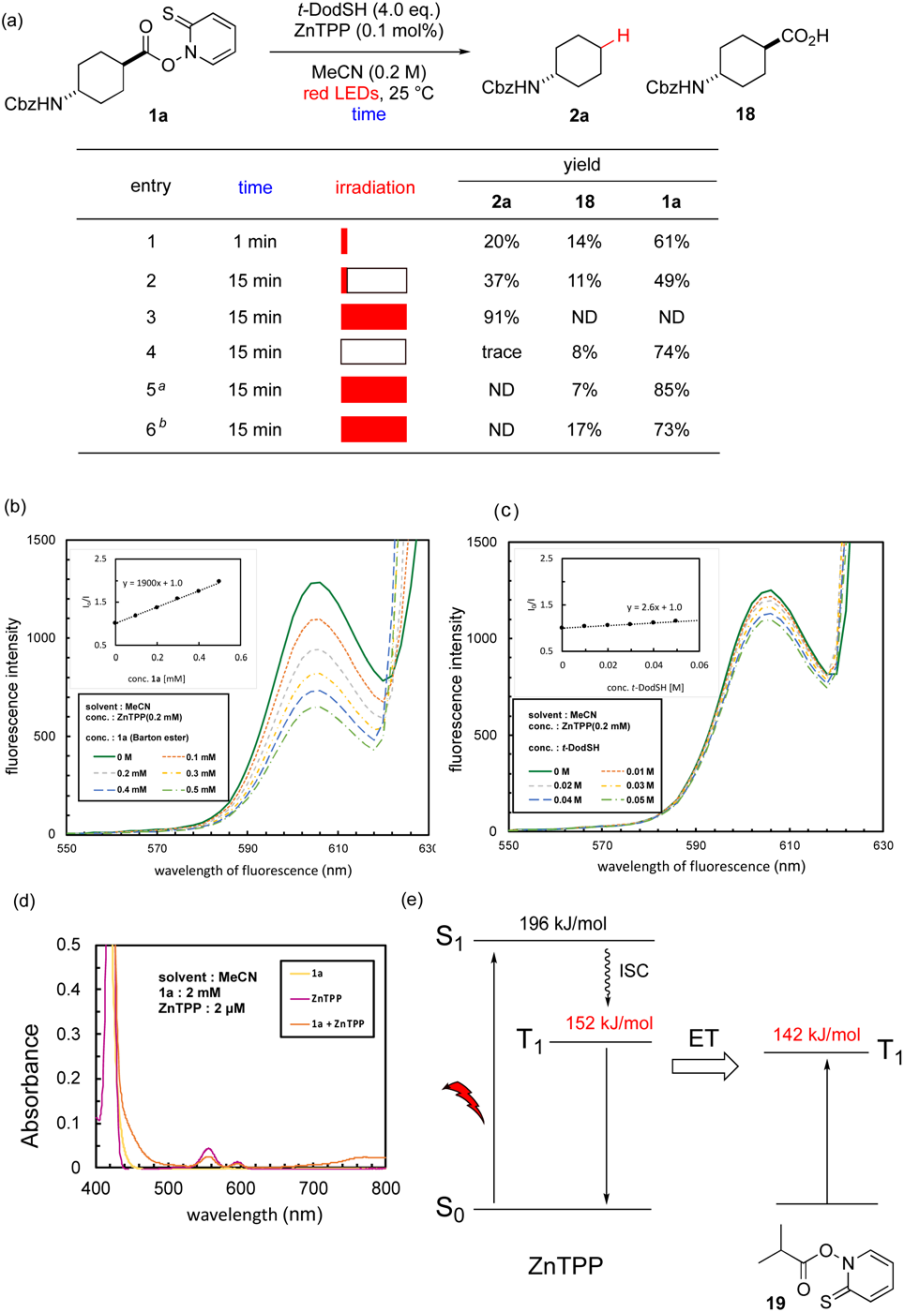
Mechanistic experiments. (a) Control experiments with different irradiation times and reagents. Red filled rectangles show red-light irradiation, and white parts indicate stirring with light shielding. *^a^*Without ZnTPP. *^b^*Without *t*-DodSH. (b) Photoluminescence spectra of quenching experiment of ZnTPP with Barton ester 1a. The inset gives the Stern–Volmer plot of the corrected quenching. (c) Photoluminescence spectra of quenching experiments of ZnTPP with *t*-DodSH. The inset gives the Stern–Volmer plot of the corrected quenching. (d) Absorbance spectrum of Barton ester 1a and ZnTPP. (e) Energy diagram of ZnTPP and Barton ester 19.

We performed several experiments to elucidate the mechanism of the red-light-mediated reaction. First, the reaction with 1a was run for only 1 min and immediately quenched, which gave 20% of 2a (entry 1, Fig. 1a). Irradiating the reaction flask for 1 min, and then stirring for another 14 min increased the yield to 37% (entry 2). This observation shows that the reaction involved a chain mechanism, which proceeded autonomously after initiation. However, comparison with entry 3 (irradiation time: 15 min) suggests that the chain length was not long enough for the reaction to be completed. The irradiation, catalyst, and thiol were all essential for the reaction because the removal of any of these factors resulted in a total loss of yield (entries 4–6).

Stern–Volmer quenching experiments were performed for ZnTPP in the presence of 1a (Fig. 1b) or *t*-dodecanethiol (Fig. 1c). Although *t*-dodecanethiol was not involved in fluorescence quenching of ZnTPP, 1a showed efficient quenching. Photon flow was calculated according to the procedure for the red-light range,^88^ and the quantum yield was calculated to be 62, which strongly supports the chain mechanism. The chain length was estimated to be 63 (see ESI† for details).^89^

The following five mechanisms for radical generation could be proposed: (i) a redox pathway, where photoactivated ZnTPP in the S_1_ or T_1_ state reduces the Barton ester; (ii) triplet–triplet annihilation of ZnTPP to form S_2_ state species, which reduce or sensitize the Barton ester; (iii) Förster energy transfer, where S_1_ state ZnTPP passes energy to the Barton ester and activates it to the S_1_ state; or (iv) Dexter energy transfer, where T_1_ state ZnTPP exchanges electrons with the Barton ester to generate the T_1_ state; (v) excitation of complex between Barton ester and ZnTPP followed by charge transfer.

First, a single-electron transfer pathway was considered. The reduction potential of 1a was measured as −1.76 V *vs.* saturated calomel electrode (SCE) in acetonitrile by differential pulse voltammetry. The redox potentials of singlet activated state and triplet state ZnTPP are −1.36 and −0.88 V (*vs.* SCE),^90,91^ respectively, according to the Rehm–Weller equation. These data suggest that a simple photoredox pathway is not feasible.

Castellano and coworkers recently showed that triplet–triplet annihilation of ZnTPP produces the high-energy S_2_ state of ZnTPP, which triggers polymerization.^91^ In their experiment, ZnTPP was excited to the S_1_ state, and S_2_ Soret-band fluorescence generated through triplet–triplet annihilation was quenched by an acrylate, whereas the S_1_ Q-band emission remained intact. However, in our system, the Q-band emission was quenched by the Barton ester, which suggests that a completely different mechanism occurred. Furthermore, redox-active esters remained intact in the red-light-mediated reaction (*e.g.*, 2n, 2o, 2p in Table 1); these functional groups are expected to be more easily reduced by a photoredox catalyst (*E*_1/2_ = −1.20 V *vs.* SCE for phthalimidyl^92^ and *E*_1/2_ = −0.79 V *vs.* SCE for tetrachlorophthalimidyl^93^) than the Barton ester. The chemoselective reaction of the Barton ester moiety suggests that there is a different pathway from the redox mechanism. In addition, ZnTPP remained intact in the reaction mixture (see ESI†), which suggests that an irreversible redox pathway is unlikely. Thus, although we cannot completely rule out a limited occurrence of the triplet–triplet annihilation pathway, there was sufficient evidence to suggest another mechanism occurred.

Förster energy transfer is not feasible due to absence of large absorption peak in the red or near-infrared region (Fig. 1d). Thus, we focused on Dexter energy transfer.^94–97^ In this mechanism, electron exchange occurs between an excited sensitizer and the substrate. This type of reaction is known to be solvent-independent, which we observed during our optimization (Table S1†). For this reaction to occur, the T_1_ energy of the sensitizer (152 kJ mol^−1^ for ZnTPP)^90^ should be larger than that of the acceptor. We performed a DFT calculation^94^ for model Barton ester 19, and the T_1_ state was above the S_0_ state by 142 kJ mol^−1^, which was ideal for the Dexter energy transfer mechanism to happen (Fig. 1e). Although a red-light-triggered energy transfer mechanism has been proposed between pheophorbide a and dithiocarbonate, the thermodynamic validity has not been discussed.^39^ Usually, Dexter energy transfer occurs from triplet state, and the singlet excited state is not involved. This should result in no change in fluorescence, whereas we observed quenching of fluorescence by the Barton ester (Fig. 1b). This phenomenon can be interpreted as an external heavy-atom effect^98^ from the sulfur atom of the Barton ester that facilitates intersystem crossing to the T_1_ state, which appears as formal quenching of Soret-band fluorescence. This mechanism is supported by the partial formation of a complex between ZnTPP and Barton ester 19 because a slight change in the absorption spectrum (460–500 nm, >720 nm) is observed when ZnTPP and 19 coexist (Fig. 1d). On the other hand, direct excitation of this complex, the fifth possibility, is unlikely: we performed calculation on complex between ZnTPP and 19, which revealed that HOMO–LUMO energy gap was 260 kJ mol^−1^, corresponding to 459 nm of light, which cannot be covered by red-light range.

## Conclusions

We have developed a red-light-mediated Barton decarboxylation reaction *via* Dexter energy transfer. The reaction was characterized by remarkably mild conditions, absence of hazardous materials, and low energy consumption. A number of related decarboxylative conversions, including new carbon–carbon bond formation, were also achieved in good yield. Furthermore, one-pot wavelength-selective transformation within the visible light region was achieved by using blue and red light, which contributed to polarity switching and broadens the scope of the Giese reaction. Further research on red light as a reaction energy source is currently underway in our laboratory.

## Data availability

All supporting data is provided in the ESI.†

## Author contributions

A. O. conceived the project. H. Y., K. Y., A. S., and K. S. performed the experimental studies. A. O. performed computational study. K. T. and A. O. prepared the manuscript.

## Conflicts of interest

There are no conflicts to declare.

## Supplementary Material

SC-014-D3SC03643J-s001
