# Supplementary material for: Red-light-mediated Barton decarboxylation reaction and one-pot wavelength-selective transformations
Source: Chem Sci. 2023 Sep 28;14(40):11243–50. doi: 10.1039/d3sc03643j (PMC10583705; doi:10.1039/d3sc03643j)
Supplement: SC-014-D3SC03643J-s001 [file SC-014-D3SC03643J-s001.pdf]

Electronic Supplementary Material (ESI) for Chemical Science.
This journal is © The Royal Society of Chemistry 2023

Supporting Information

Table of Contents

1.	Optimization	S2
2.	Additional reaction examples	S14
3.	Proposed mechanism for thioester reduction	S16
4.	Experimental procedures	S18
5.	Stern–Volmer emission quenching	S77
6.	Differential pulse voltammetry	S79
7.	HPLC profiles of ZnTPP before/after the reaction	S80
8.	UV-vis spectra of ZnTPP and $Ru(bpy)_3Cl_2$	S81
9.	Computational methods	S82
10.	^{1}H and ^{13}C NMR spectra of new compounds	S87

1. Optimization

Table S1: Summary of optimization for red light-mediated Barton decarboxylation.

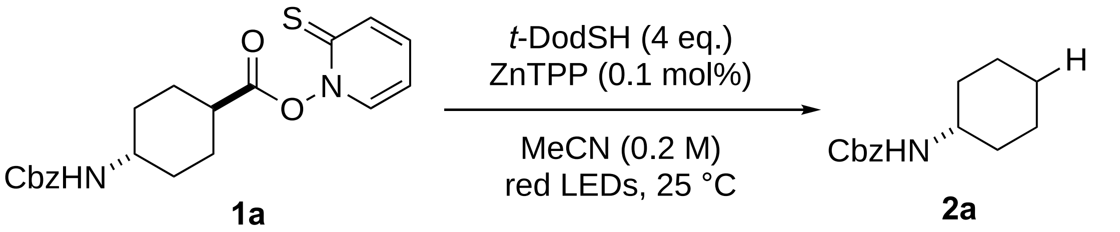

entry	difference from optimized conditions	yield (%)			
		**2a**	**S1**	**18**	**1a**
1	(none)	91	ND	ND	ND
2[a,b,c]	*t*-BuSH as hydrogen source	83	5	ND	ND
3[a,b,c]	*n*-Bu_3SnH as hydrogen source	46	13	ND	ND
4[a,b,c]	PhSH as hydrogen source	54	ND	ND	ND
5[a,b,c]	Hantzsch ester as hydrogen source	trace	41	ND	ND
6[a,b]	DMA as solvent	88	ND	ND	ND
7[a,b]	DMF as solvent	87	ND	ND	ND
8[a,b]	CH_2Cl_2 as solvent	80	ND	ND	ND
9[a,b]	EtOAc as solvent	60	ND	ND	36
10[a,b]	benzene as solvent	44	ND	ND	52
11[a,b]	EtOH as solvent	64	ND	ND	ND
12[a]	0.1 M	89	ND	ND	ND
13[a]	1.0 M	36	ND	10	30
14	no ZnTPP, no irradiation, 50 °C, 6 h	73	ND	ND	ND
15	TEMPO (4.0 eq.)	ND	ND	ND	78

10 mg scale and 15 min unless otherwise noted. ND = not detected.
[a] Chlorophyll a instead of ZnTPP. [b] 0.05 M.
[c] DMSO as solvent. 3 mol% catalyst. 5 h.

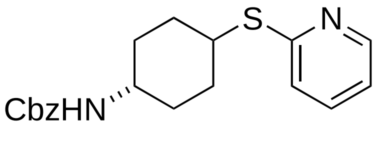

S1

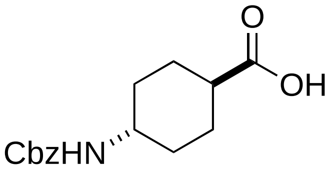

18

Table S2: Summary of optimization for red light-mediated Barton decarboxylative chlorination.

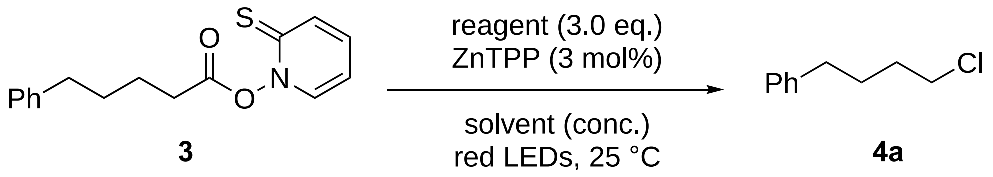

entry	reagent	solvent	conc. (M)	yield (%)	
				**4a**	**S2**
1	NCS	toluene	0.05	ND	ND
2	TMSCl	toluene	0.05	ND	ND
3	CCl_4	toluene	0.05	ND	37
4	PhSCl	toluene	0.05	ND	ND
5	hexachloroethane	toluene	0.05	ND	34
6	–	CCl_4	0.05	51	16
7	–	CCl_4	0.025	47	11
8	–	CCl_4	0.1	42	16
9	–	CCl_4	0.2	20	15
10	–	$Cl_2HCCHCl_2$	0.05	ND	–[a]
11	hexachloroethane	CCl_4	0.05	73	12
12	hexachloroethane[b]	CCl_4	0.05	69	8

20 mg scale and 1 h.
[a] obtained as inseparable mixture with an unidentified byproduct.
[b] 6.0 eq. was used.

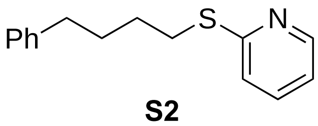

S2

Table S3: Summary of optimization for red light-mediated Barton decarboxylative bromination.

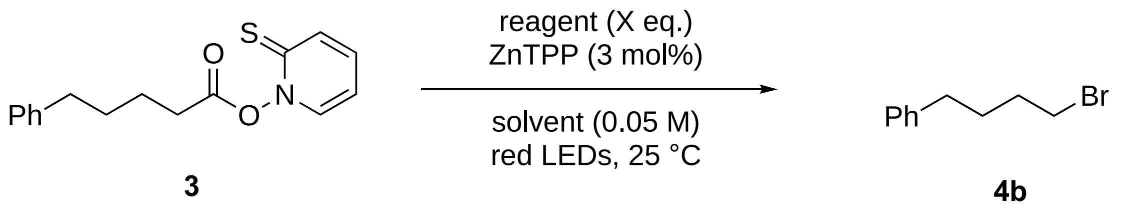

entry	reagent	X eq.	solvent	yield (%)			
				**4b**	**S2**	**S3**	**3**
1	NBS	4.0	DMSO	ND	ND	ND	ND
2	NBA	4.0	DMSO	ND	58	ND	ND
3	TMSBr	4.0	DMSO	ND	20	ND	ND
4	CH_2BrCl	4.0	DMSO	ND	30	ND	6
5	CBr_4	4.0	DMSO	23	50	ND	ND
6	$CBrCl_3$	4.0	DMSO	44[a]	36[a]	ND	ND
7	$CBrCl_3$	3.0	DMSO	50	14	20	ND
8	$CBrCl_3$	2.0	DMSO	43	ND	20	ND
9	$CBrCl_3$	1.0	DMSO	44	ND	20	ND
10	$CBrCl_3$	3.0	DMF	28	ND	19	ND
11	$CBrCl_3$	3.0	CH_3CN	39	ND	trace	ND
12	$CBrCl_3$	3.0	EtOH	ND	ND	7	ND
13	$CBrCl_3$	3.0	CH_2Cl_2	53	ND	14	ND
14	$CBrCl_3$	3.0	toluene	55	ND	14	ND

10 mg scale and 1 h.
[a] contains small amount of chlorinated compound **4a**.

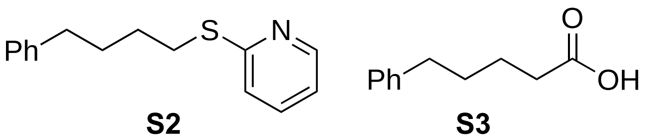

S2 **S3**

Table S4: Summary of optimization for red light-mediated Barton decarboxylative iodination.

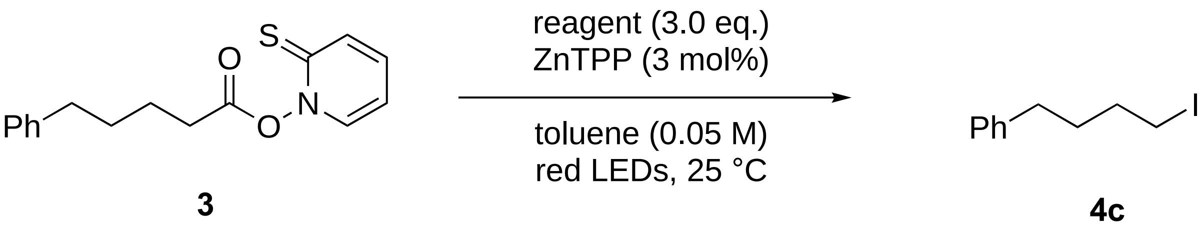

entry	reagent	yield (%) 4c	yield (%) S2
1	NIS	7	3
2	CHI_3	23	6
3	CH_2I_2	70	trace
4	CH_3I	8	33
5	CH_2ClI	56	8

10 mg scale and 1 h.

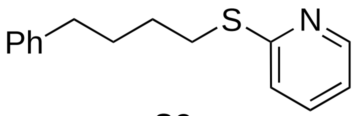

S2

Table S5: Summary of optimization for red light-mediated Barton decarboxylative oxygenation.

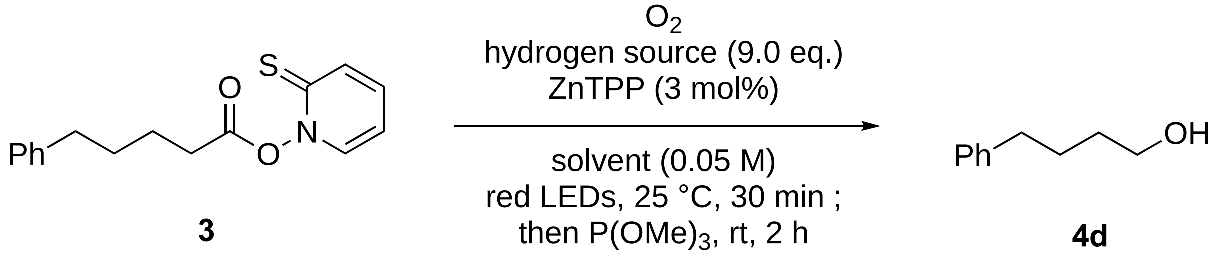

entry	hydrogen source	X eq.	solvent	yield (%)
1	*t*-BuSH	0.05	toluene	38
2	*t*-BuSH	0.05	CH_2Cl_2	21
3	*t*-BuSH	0.05	CH_3CN	15
4	*t*-BuSH	0.05	DMF	19
5	*t*-BuSH	0.05	EtOH	75
6	*t*-BuSH	0.1	EtOH	64
7	*t*-BuSH	0.025	EtOH	27
8	PhSH	0.05	EtOH	35
9	*t*-DodSH	0.05	EtOH	42
10	TTMSS	0.05	EtOH	59
11	Et_3SiH	0.05	EtOH	51
12	Ph_2SiH_2	0.05	EtOH	54
13	*n*-Bu_3SnH	0.05	EtOH	39
14[a]	*t*-BuSH	0.05	EtOH	57

10 mg scale.
[a] 4.0 eq. of *t*-BuSH.

Table S6: Summary of optimization for red light-mediated Barton decarboxylative nitrosation.

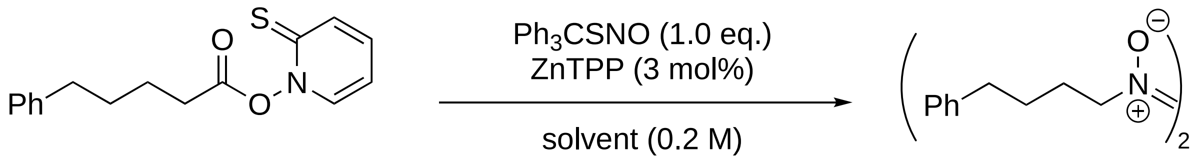

3 **4e**

entry	solvent	yield (%)
1	CH_3CN	31
2	DMSO	14
3	DMF	30
4	THF	19
5	toluene	51
6	CH_2Cl_2	39
7	DMF/toluene (2:1)	37
8	CH_2Cl_2/toluene (2:1)	50
9[a]	CH_2Cl_2/toluene (2:1)	34
10[b]	CH_2Cl_2/toluene (2:1)	49
11[b,c]	CH_2Cl_2/toluene (2:1)	53
12[b,d]	CH_2Cl_2/toluene (2:1)	61

10 mg scale and 1.5 h unless otherwise noted. ND = not detected.
In this optimization, solubility of Ph_3CSNO to various solvent was problematic.
This is why the CH_2Cl_2/toluene mixed solvent was chosen as optimum solvent.
[a] 2.0 eq. of Ph_3CSNO was used. [b] solution of 1z and Ph_3CSNO was slowly added.
[c] 0.1 M. [d] 0.05 M.

Table S7: Full optimization for red light-mediated Barton decarboxylative hydrazination.

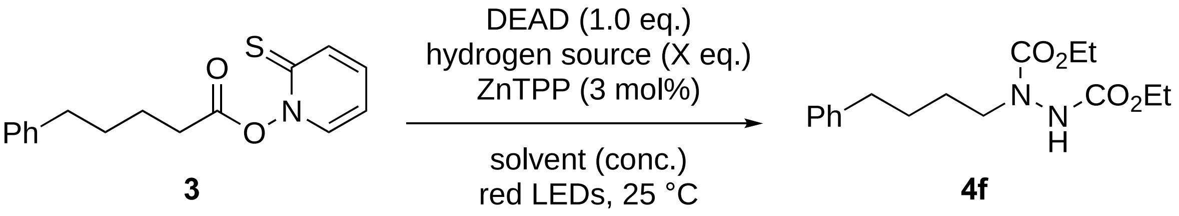

entry	hydrogen source	X eq.	solvent	conc. (M)	yield (%)
1	Ph_3CSH	4.0	CH_2Cl_2/toluene (2:1)	0.05	ND
2	TTMSS	4.0	CH_2Cl_2/toluene (2:1)	0.05	16
3	TTMSS	4.0	toluene	0.05	35
4	TTMSS	4.0	CH_2Cl_2	0.05	31
5	TTMSS	4.0	CH_3CN	0.05	27
6	TTMSS	4.0	DMF	0.05	23
7	TTMSS	4.0	EtOH	0.05	25
8	TTMSS	4.0	toluene	0.1	57
9	TTMSS	4.0	toluene	0.2	49
10[a]	TTMSS	4.0	toluene	0.1	55
11[b]	TTMSS	4.0	toluene	0.1	40
12	Et_3SiH	4.0	toluene	0.1	17
13	Ph_2SiH_2	4.0	toluene	0.1	28
14	TTMSS	2.0	toluene	0.1	24
15	TTMSS	1.0	toluene	0.1	20
16	TTMSS	8.0	toluene	0.1	45

10 mg scale and 1.5 h unless otherwise noted. ND = not detected.
[a] 1.5 eq. of DEAD was used. [b] 2.0 eq. of DEAD was used.

Table S8: Full optimization for red light-mediated Barton decarboxylative sulfidation.

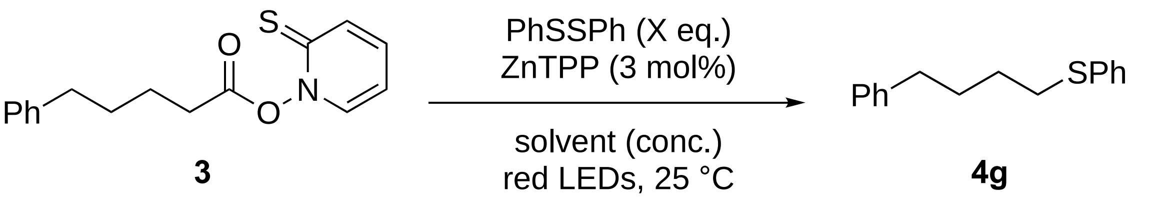

entry	X eq.	solvent	conc. (M)	yield (%)
1	2.0	DMSO	0.05	84
2	2.0	DMF	0.05	62
3	2.0	CH_3CN	0.05	29
4	2.0	CH_2Cl_2	0.05	15
5	2.0	toluene	0.05	23
6	2.0	EtOH	0.05	16
7	2.0	DMSO	0.1	64
8	2.0	DMSO	0.2	67
9	1.5	DMSO	0.05	66
10	1.0	DMSO	0.05	61
11	4.0	DMSO	0.05	50

10 mg scale and 1.5 h.

Table S9: Summary of optimization for red light-mediated Barton decarboxylative selenidation.

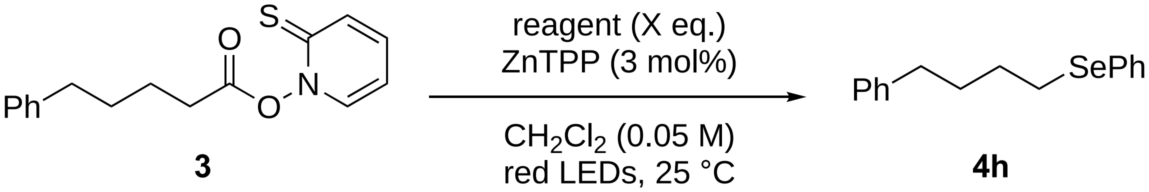

entry	reagent	X eq.	yield (%)	
			**4h**	**S3**
1	PhSeBr	1.0	trace	quant.
2	$(PhSe)_2$	1.0	91	trace
3	$(PhSe)_2$	0.5	85	trace

10 mg scale and 1 h.

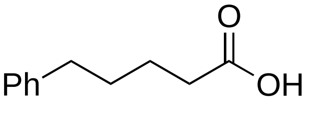

S3

Table S10: Full optimization for red light-mediated Barton decarboxylative borylation.

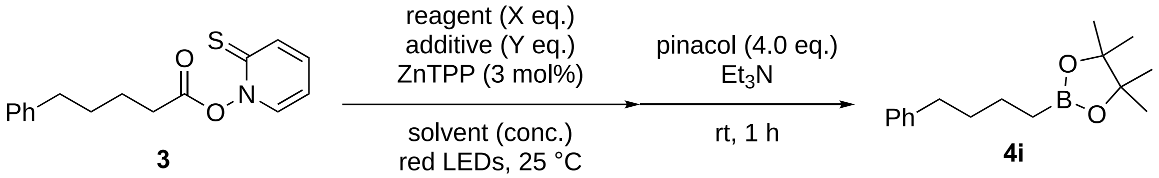

entry	reagent	X eq.	additive	Y eq.	solvent	conc. (M)	yield (%)			
							**4i**	**S2**	**S4**	**S5**
1[a]	B_2pin_2	4.0	none	–	DMF	0.05	ND	44	ND	ND
2[a]	B_2pin_2	4.0	Et_3N	4.0	DMF	0.05	ND	25	ND	ND
3[a]	B_2pin_2	4.0	pyridine	4.0	DMF	0.05	ND	23	ND	ND
4[a]	B_2pin_2	4.0	*t*-BuOK	4.0	DMF	0.05	ND	54	ND	ND
5[a]	B_2pin_2	4.0	CsF	4.0	DMF	0.05	ND	34	ND	ND
6	$B_2(OH)_2$	4.0	none	–	DMF	0.2	15	2	ND	5
7	B_2cat_2	4.0	none	–	DMF	0.2	28	9	10	ND
8	B_2cat_2	4.0	none	–	DMA	0.2	25	trace	ND	ND
9	B_2cat_2	4.0	none	–	DMSO	0.2	ND	5	ND	ND
10	B_2cat_2	4.0	none	–	CH_3CN	0.2	11	trace	trace	22
11	B_2cat_2	4.0	none	–	EtOAc	0.2	12	trace	31	ND
12	B_2cat_2	4.0	none	–	toluene	0.2	14	trace	7	12
13	B_2cat_2	4.0	none	–	MeOH	0.2	24	trace	ND	ND
14	B_2cat_2	4.0	none	–	THF	0.2	23	trace	trace	ND
15	B_2cat_2	3.0	none	–	DMF	0.2	40	3	ND	ND
16	B_2cat_2	2.0	none	–	DMF	0.2	48	trace	ND	ND
17	B_2cat_2	1.0	none	–	DMF	0.2	8	18	ND	ND
18	B_2cat_2	2.0	pyridine	2.0	DMF	0.2	18	trace	ND	ND
19	B_2cat_2	2.0	Et_3N	2.0	DMF	0.2	40	11	ND	ND
20	B_2cat_2	2.0	DABCO	2.0	DMF	0.2	24	21	ND	ND
21	B_2cat_2	2.0	*t*-BuOK	2.0	DMF	0.2	22	4	ND	ND
22	B_2cat_2	2.0	HMPA	2.0	DMF	0.2	58	3	ND	ND
23	B_2cat_2	2.0	Ph_3P	2.0	DMF	0.2	30	trace	ND	ND
24	B_2cat_2	2.0	H_2O	20	DMF	0.2	52	11	ND	ND
25	B_2cat_2	2.0	DMPU	2.0	DMF	0.2	54	ND	ND	ND
26	B_2cat_2	2.0	NMP	2.0	DMF	0.2	51	ND	ND	ND
27	B_2cat_2	2.0	HMPA	10	DMF	0.2	53	ND	ND	ND
28	B_2cat_2	2.0	HMPA	2.0	DMF	0.1	63	ND	ND	ND
29	B_2cat_2	2.0	HMPA	2.0	DMF	0.05	51	ND	ND	ND
30	B_2cat_2	2.0	HMPA	2.0	DMF	0.025	67	ND	ND	ND
31	B_2cat_2	2.0	HMPA	2.0	DMF	0.01	67	ND	ND	ND

20 mg scale and 1 h.
[a] second reaction was not conducted.

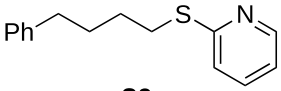

S2

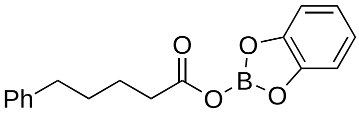

S4

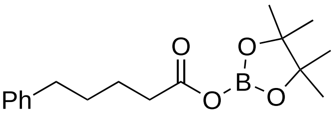

S5

Table S11: Summary of optimization for red light-mediated Barton decarboxylative Giese reaction.

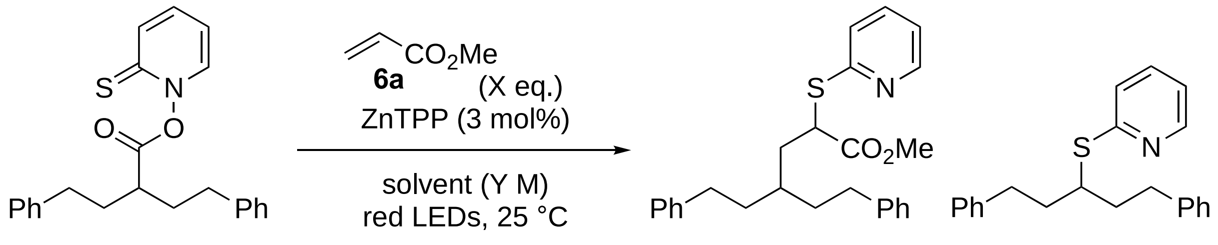

entry	X eq.	solvent	Y M	yield (%)	
				**7a**	**8a**
1	4.0	DMSO	0.1	35	11
2	4.0	DMF	0.1	49	20
3	4.0	MeCN	0.1	33	21
4	4.0	EtOH	0.1	trace	29
5	4.0	CH_2Cl_2	0.1	62	30
6	4.0	toluene	0.1	70	20
7	4.0	toluene	0.05	56	24
8	4.0	toluene	0.2	62	20
9	2.0	toluene	0.1	53	29
10	8.0	toluene	0.1	62	13

10 mg scale and 1 h.

Table S12: Summary of optimization for blue light-mediated decarboxylation of **7e**.

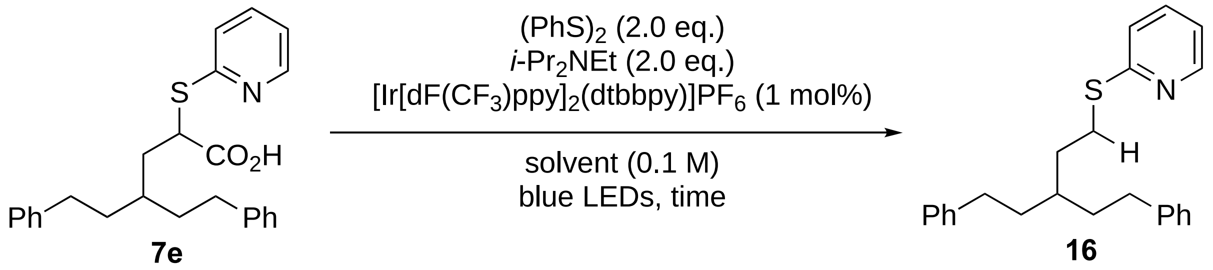

entry	solvent	time	additive	yield (%)	
				**16**	**7e**
1	toluene	1 d	–	13	ND
2	toluene/H_2O (10:1)	1 d	–	36	49
3	CH_3OH/H_2O (10:1)	1 d	–	16	47
4	CH_3CN/H_2O (10:1)	1 d	–	ND	ND
5	DMF/H_2O (10:1)	2 d	–	61	20
6	toluene/H_2O (10:1)	2 d	–	66	20
7	toluene/H_2O (10:1)	3 d	–	60	ND
8	toluene/H_2O (10:1)	3 d	TBAI	92	ND
9	toluene/H_2O (10:1)	1 d	TBAI	95	ND

Table S13: Summary of optimization for one-pot Giese reaction/decarboxylation of **5**.

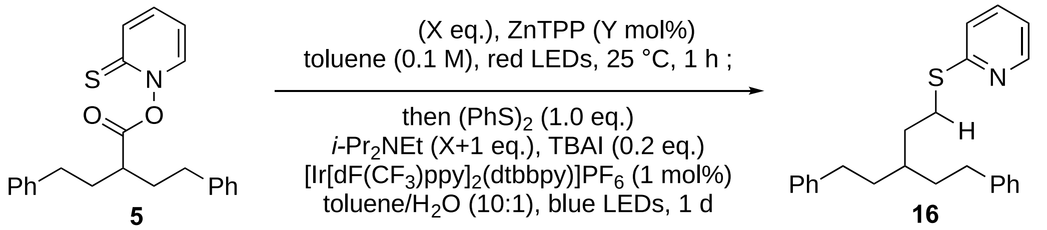

entry	X eq.	Y mol%	yield (%)		
			**16**	**8**	**7e**
1[a]	4.0	3	ND	10	92 [b]
2	4.0	3	13	11	40
3	3.0	3	64	10	trace
4	2.5	3	67	15	ND
5	2.0	3	60	18	ND
6	1.0	3	38	26	8
7	2.5	1	49	15	ND
8	2.5	10	19	11	45

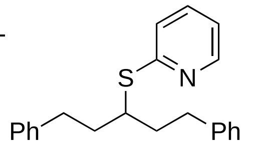

8

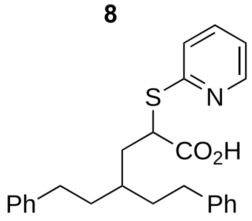

7e

a) with 4.0 eq. of *i*-Pr_2NEt
b) with inseparable impurities

Table S14: Summary of optimization for decarboxylative generation of alcohol **17** from **7e**.

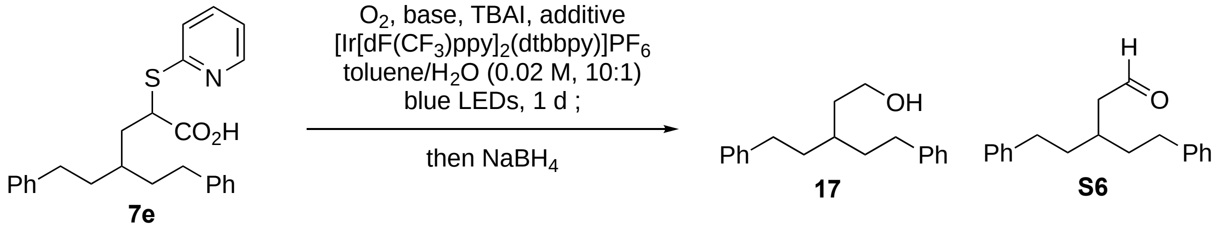

entry	base	additive	$NaBH_4$	yield (%)	
				**17**	**S6**
1	Na_2CO_3	–	–	ND	17
2	Na_2CO_3	–	○	46	ND
3	Na_2CO_3	PPh_3	○	64	ND
4	Na_2CO_3	$P(OPh)_3$	○	80	ND
5	K_2CO_3	$P(OPh)_3$	○	63	ND
6	NaOH	$P(OPh)_3$	○	32	ND
7	K_2HPO_4	$P(OPh)_3$	○	66	ND
8	*i*-Pr_2NEt	$P(OPh)_3$	○	52	ND

2. Additional reaction examples

Table S15: Examples for Barton decarboxylative selenidation.

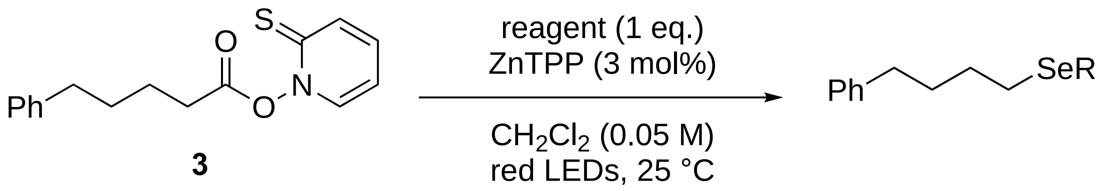

entry	reagent	yield (%)	
1	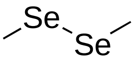	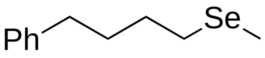	**S7** 89%
2	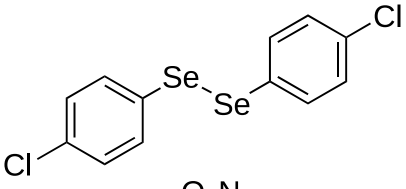	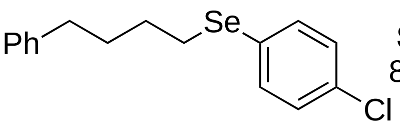	**S8** 85%
3	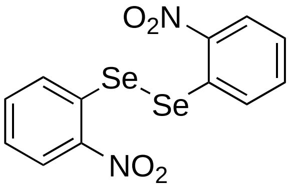	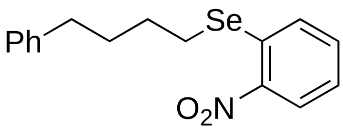	**S9** ND

10 mg scale and 1 h.

Scheme S1: Examples for one-pot Barton decarboxylative reaction/blue light-mediated reactions.

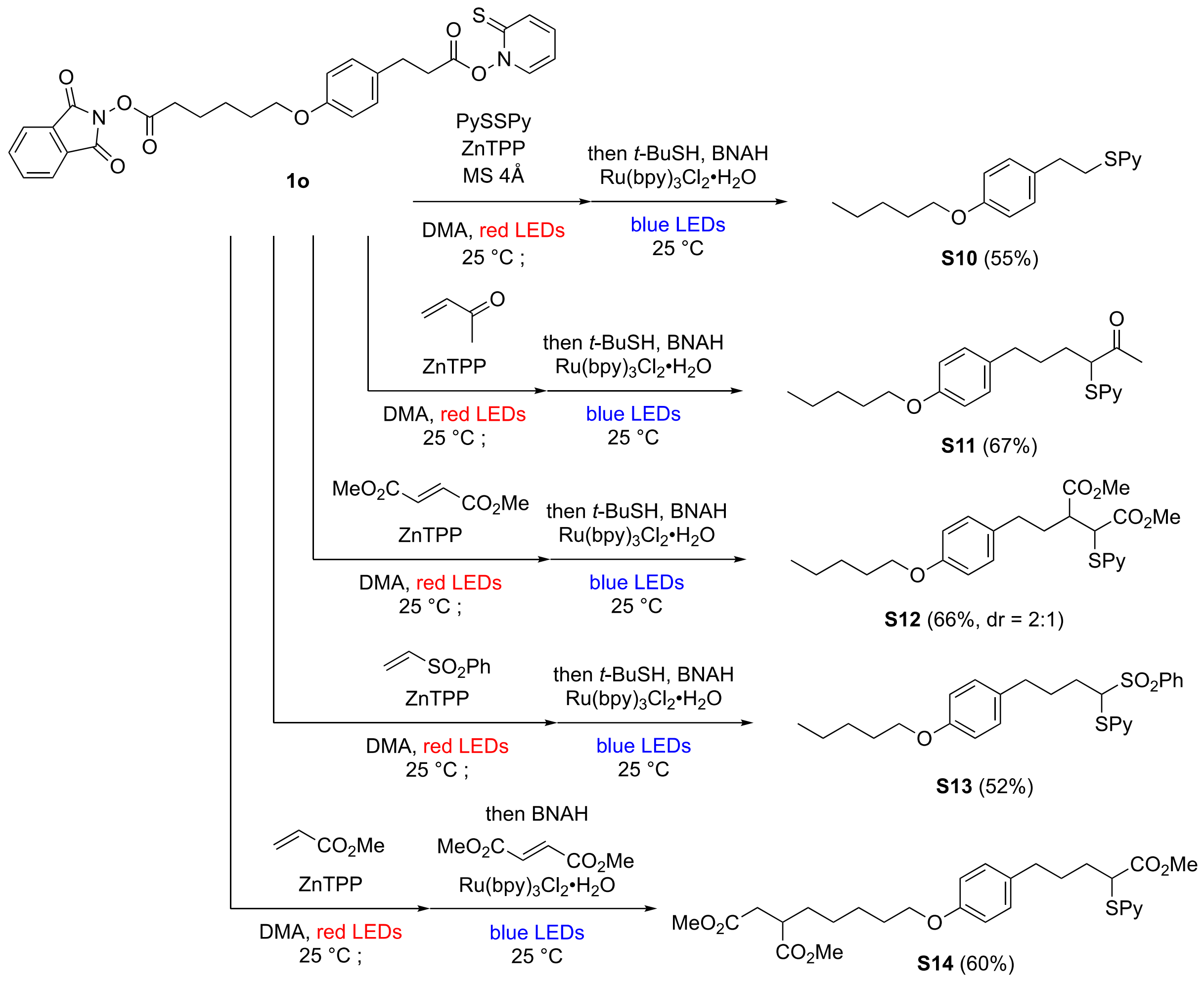

3. Proposed mechanism for thioester reduction

Scheme S2: Proposed reaction mechanism for decarboxylative generation of aldehyde from **7e**. (A) Decarboxylation of **7e** to generate thioester. (B) Reduction of thioester to aldehyde.

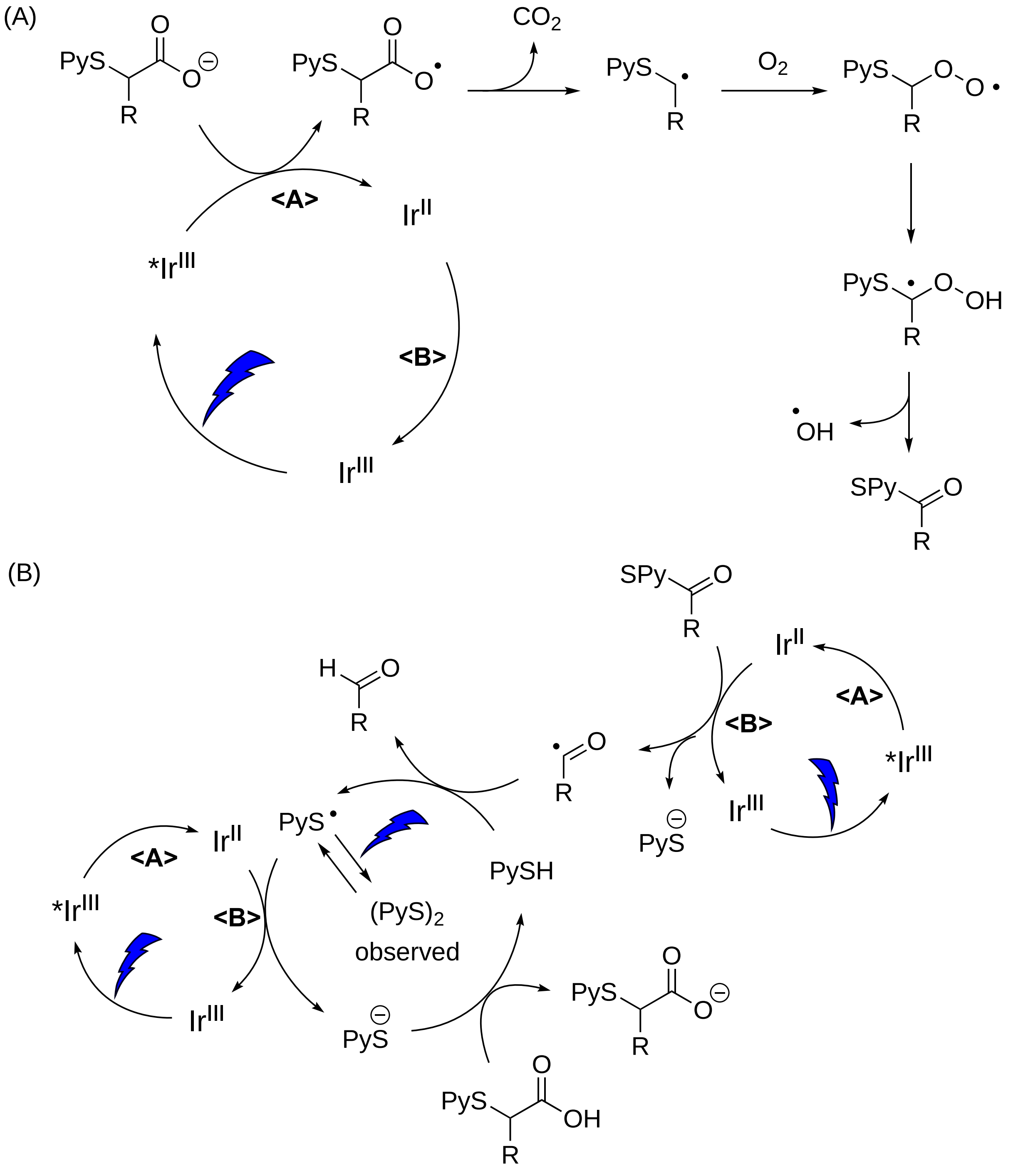

Scheme S3: Proposed reaction mechanism for decarboxylative generation of aldehyde from **7e** in the presence of phosphine.

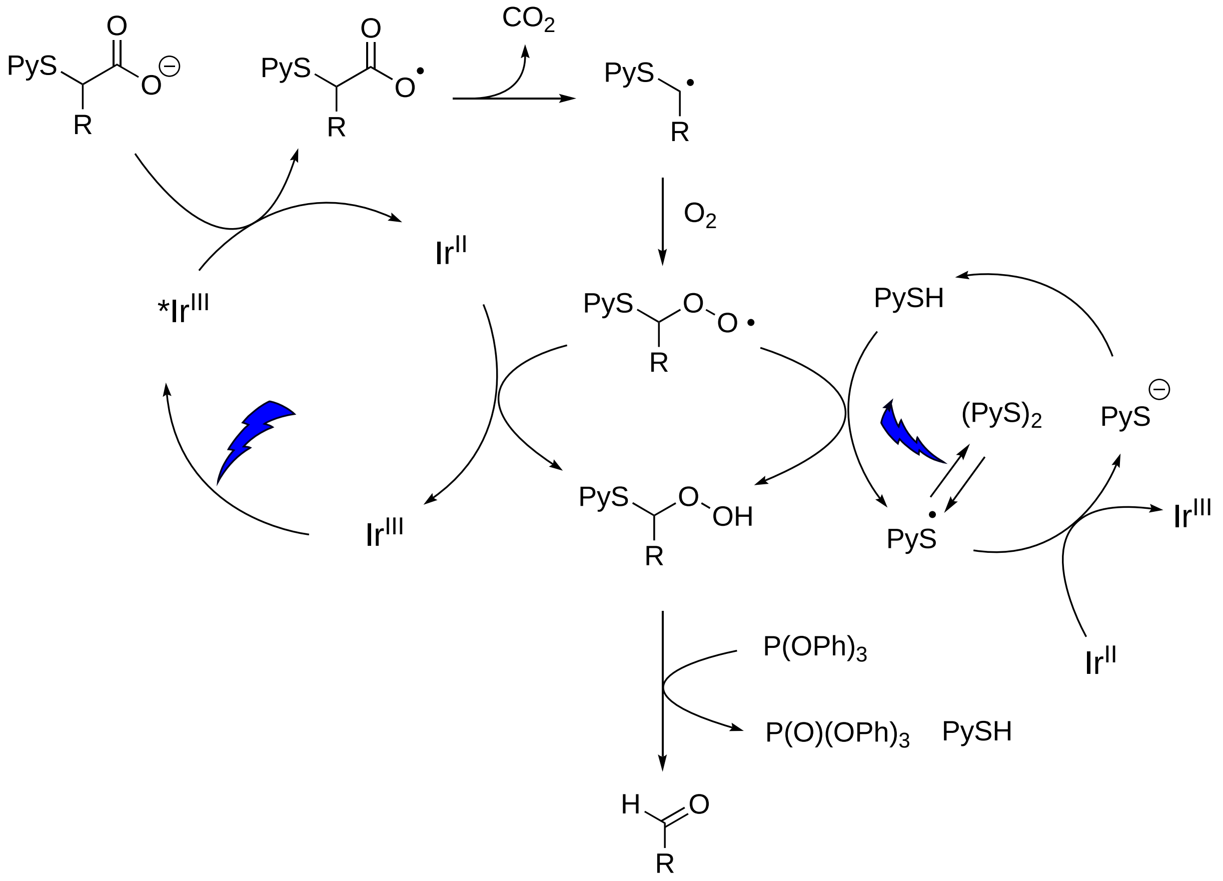

4. Experimental procedures

General methods. Melting points are uncorrected. Specific rotations were measured in a 100 mm cell. ^{1}H HMR spectra were recorded at 400 MHz or 500 MHz with tetramethylsilane (0 ppm), residual chloroform (7.26 ppm) or residual dimethyl sulfoxide (2.54 ppm) as an internal standard on a JEOL JNM-ECS400 (400 MHz), JEOL JNM-ECZ400 (400 MHz) or JEOL JNM-ECA500 (500 MHz) spectrometer. ^{13}C NMR spectra were recorded at 100 MHz or 125 MHz, and referenced to residual chloroform (77 ppm) or residual dimethyl sulfoxide (39.5 ppm). High-resolution mass spectra (HRMS) were measured by the ESI mode on a Waters LCT premier XE spectrometer. The diffuse reflectance UV-vis spectra were measured by Shimadzu UV-3600Plus with wavelength range between 400 nm and 800 nm. Fluorescence spectra were measured by Shimadzu RF-6000. Gas chromatography (GC) analysis was performed using a CBP-10 capillary column (25 m $\times$ 0.22 mm, film thickness 0.25 μm). HPLC analysis was performed with Daicel Chiralcel OD-H 0.46 cmΦ $\times$ 25 cm column. Thin-layer chromatography (TLC) was performed on Merck Kieselgel 60 F_{254} plates. The crude reaction mixtures and extracted materials were purified by chromatography on silica gel (Fuji Silysia, PSQ-100B) or PTLC (Merck). Combined organic extracts were dried over anhydrous Na_2SO_4. Solvents were removed from the reaction mixture and the combined organic extracts by concentration under reduced pressure using an evaporator with bath at 35–45 °C. Yields are isolated yields unless otherwise noted.

Light-mediated reaction setup. A strip of red LEDs (4W) was purchased from akibaLED PIKARIKAN, Japan. The strip was coiled and pasted inside a plastic cup of 8 cm diameter (Figure S1a). The reaction flask equipped with a magnetic stir bar was placed at the center of the cup and covered with aluminum foil. The reaction mixture was irradiated by red LEDs from 2.5 cm distance with continuous stirring (Figure S1b, S1c). The plastic cup was cooled by an external water bath when necessary.

For blue light-mediated reactions, a strip of blue LEDs (10W) was used and set up as described above.

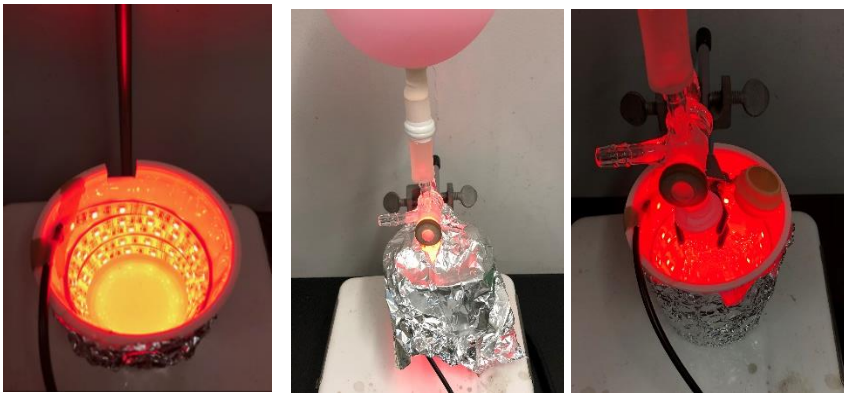

Figure S1. Reaction setup. (a, left) a strip of LEDs is coiled on inner face of plastic cup. (b, center) (c, right) the reaction flask is irradiated by red LEDs.

3-[4-(*tert*-Butyldimethylsilyloxy)phenyl]propionic acid (S16).

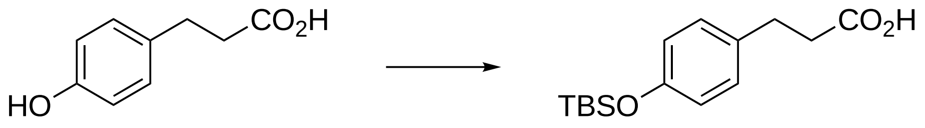

S15 **S16**

The following reaction was carried out under Ar. To a cooled (0 °C) stirred solution of 3-(4-hydroxyphenyl)propionic acid (**S15**) (201 mg, 1.21 mmol) in DMF (2 mL) were added TBSCl (405 mg, 2.70 mmol) and imidazole (286 mg, 4.20 mmol). After being stirred at room temperature for 1 h, the mixture was diluted with H_2O (10 mL) and extracted with hexane (10 mL×3). The combined extracts were dried and concentrated under reduced pressure to provide crude silyl ester, which was used in the next step without further purification.

To a stirred solution of crude silyl ester obtained above in MeOH/THF (1:1, 2 mL) was added K_2CO_3 (347 mg, 2.50 mmol). After being stirred at room temperature for 14 h, the mixture was quenched with 1 M aqueous HCl (10 mL) and extracted with EtOAc (10 mL×3). The combined extracts were dried and concentrated under reduced pressure. The residue was purified by column chromatography on silica gel (EtOAc/hexane, 1:8) to provide 148 mg (43%) of **S16** as white crystals. mp 59-61 °C. TLC R_f 0.31-0.56 (EtOAc/hexane, 1:1). IR (KBr): 3400-2500, 2950, 1718, 1655 cm^{-1}. ^1H NMR (500 MHz, $CDCl_3$): δ 7.06 (d, 2H, J =8.3 Hz, H-2, 6 of Ar), 6.76 (d, 2H, J =8.3 Hz, H-3, 5 of Ar), 2.89 (t, 2H, J =7.6 Hz, H-3, 3'), 2.65 (t, 2H, J =7.6 Hz, H-2, 2'), 0.97 (s, 9H, t-Bu), 0.18 (s, 6H, -Me). $^{13}C\{^1H\}$ NMR (125 MHz, $CDCl_3$): δ 178.8, 154.1, 132.8, 129.1 (2C), 120.1 (2C), 35.8, 29.8, 25.7 (3C), 18.2, -4.4 (2C). HRMS (ESI-TOF) m/z: $[M + H]^+$ Calcd for $C_{15}H_{25}O_3Si$ 281.1573; Found 281.1572.

3-[4-(Pivaloyloxy)phenyl]propionic acid (S17).

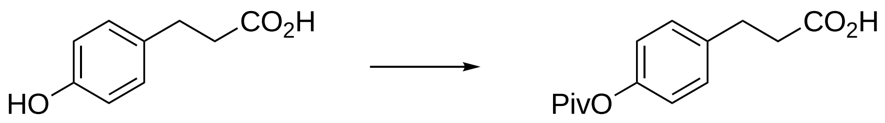

S15 **S17**

The following reaction was carried out under Ar. To a cooled (0 °C) stirred solution of 3-(4-hydroxyphenyl)propionic acid (**S15**) (202 mg, 1.21 mmol) in CH_2Cl_2 (4 mL) were added PivCl (0.293 mL, 2.41 mmol), Et_3N (0.334 mL, 2.41 mmol), and DMAP (14.9 mg, 0.122 mmol). After being stirred at room temperature for 2 d, the mixture was quenched with 1 M aqueous HCl (1 mL), diluted with H_2O (10 mL) and extracted with CH_2Cl_2 (10 mL×3). The combined extracts were washed with saturated brine (10 mL), dried and concentrated under reduced pressure. The residue was purified by column chromatography on silica gel (EtOAc/toluene, 1:1) to provide 208 mg (69%) of **S17** as white crystals. mp 88-91 °C. TLC R_f 0-0.40 (EtOAc/toluene, 1:1). IR (KBr): 3500-2500, 2975, 1752, 1716, 1695 cm^{-1}. ^1H NMR (500 MHz, $CDCl_3$): δ 7.10 (d, 2H, J =8.5 Hz, H-2, 6 of Ar), 6.80 (d, 2H, J =8.5 Hz, H-3, 5 of Ar), 2.95 (t, 2H, J =7.8 Hz, H-3, 3'), 2.67 (t, 2H, J =7.8 Hz, H-2, 2'), 1.35 (s, 9H, t-Bu). $^{13}C\{^1H\}$ NMR (125 MHz, $CDCl_3$): δ 178.5, 177.2, 149.5, 137.4, 129.2 (2C), 121.5 (2C), 39.0, 35.5, 29.9, 27.1 (3C). HRMS (ESI-TOF) m/z: $[M + Na]^+$ Calcd for $C_{14}H_{18}O_4Na$ 273.1103;

Found 273.1111.

3-[4-(Methanesulfonyloxy)phenyl]propionic acid (S18).

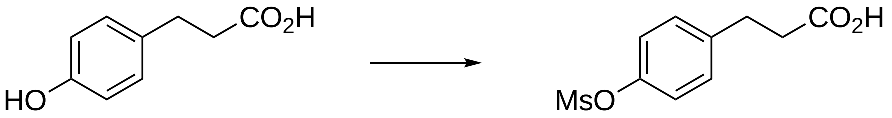

S15 **S18**

The following reaction was carried out under Ar. To a cooled (0 °C) stirred solution of 3-(4-hydroxyphenyl)propionic acid (**S15**) (200 mg, 1.20 mmol) in H_2O (12 mL) were added NaOH (181 mg, 4.52 mmol) and MsCl (0.116 mL, 1.50 mmol). After being stirred at room temperature for 18 h, the mixture was quenched with 1 M aqueous HCl (10 mL). The insoluble solids were collected by filtration and washed well with H_2O. The solids were dissolved in CH_2Cl_2 (20 mL) and washed with H_2O (10 mL×3) and saturated brine (10 mL), sequentially. The organic layer was dried and concentrated under reduced pressure. The residue was purified by column chromatography on silica gel (EtOAc/hexane, 1:1) to provide 130 mg (44%) of **S18** as white crystals. mp 98-101 °C. TLC R_f 0-0.27 (EtOAc/hexane, 4:1). IR (KBr): 3500-2500, 3067, 1709 cm^{-1}. ^{1}H NMR (500 MHz, $CDCl_3$): δ 7.26 (d, 2H, *J* =8.5 Hz, H-2, 6 of Ar), 7.10 (d, 2H, *J* =8.5 Hz, H-3, 5 of Ar), 3.14 (s, 3H, -Me), 2.97 (t, 2H, *J* =7.5 Hz, H-3, 3'), 2.69 (t, 2H, *J* =7.5 Hz, H-2, 2'). ^{13}C{^{1}H} NMR (125 MHz, $CDCl_3$): δ 177.7, 147.7, 139.6, 129.9 (2C), 122.1 (2C), 37.3, 35.2, 29.8. HRMS (ESI-TOF) *m*/*z*: [M + K]$^+$ Calcd for $C_{10}H_{12}KO_5S$ 283.0043; Found 283.0046.

3-[4-(Benzyloxy)phenyl]propionic acid (S19).

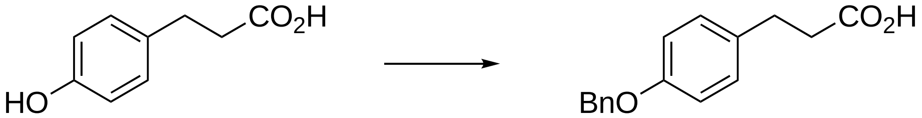

S15 **S19**

The following reaction was carried out under Ar. To a stirred solution of 3-(4-hydroxyphenyl)propionic acid (**S15**) (202 mg, 1.22 mmol) in THF (6 mL) were added BnBr (0.150 mL, 1.27 mmol), KOH (169 mg, 3.01 mmol), and NaI (3.6 mg, 0.024 mmol). The mixture was refluxed for 18 h, and H_2O (6 mL) was added. After being refluxed for 2 h, the mixture was quenched with 3 M aqueous HCl (10 mL) and extracted with CH_2Cl_2 (10 mL×3). The combined extracts were washed with saturated brine (10 mL), dried and concentrated under reduced pressure. The residue was purified by column chromatography on silica gel (EtOAc/toluene, 1:10) to provide 157 mg (51%) of **S19** as white crystals. mp 90-93 °C. TLC R_f 0.38-0.53 (EtOAc/hexane, 1:1). IR (KBr): 3500-2500, 3030, 1696 cm^{-1}. ^{1}H NMR (500 MHz, $CDCl_3$): δ 7.43 (d, 2H, *J* =7.3 Hz, H-2, 6 of Ph), 7.39 (t, 2H, *J* =7.3 Hz, H-3, 5 of Ph), 7.33 (t, 1H, *J* =7.3 Hz, H-4 of Ph), 7.13 (d, 2H, *J* =8.5 Hz, H-2, 6 of Ar), 6.92 (d, 2H, *J* =8.5 Hz, H-3, 5 of Ar), 5.05 (s, 2H, Bn), 2.91 (t, 2H, *J* =7.9 Hz, H-3, 3'), 2.66 (t, 2H, *J* =7.9 Hz, H-

2, 2’). $^{13}C\{^1H\}$ NMR (125 MHz, $CDCl_3$): δ 178.6, 157.4, 137.1, 132.5, 129.2 (2C), 128.6 (2C), 127.9, 127.4 (2C), 114.9 (2C), 70.0, 35.8, 29.7. HRMS (ESI-TOF) *m*/*z*: $[M + Na]^+$ Calcd for $C_{16}H_{16}NaO_3$ 279.0997; Found 279.1003.

3-[4-(*p*-Methoxybenzyloxy)phenyl]propionic acid (S20).

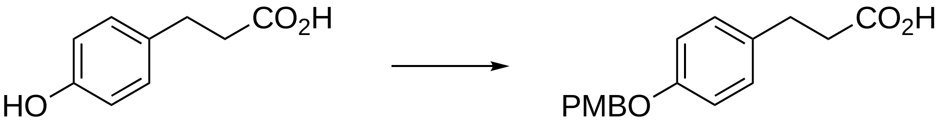

S15 **S20**

The following reaction was carried out under Ar. To a stirred solution of 3-(4-hydroxyphenyl)propionic acid (**S15**) (99.8 mg, 0.601 mmol) in DMF (1 mL) were added PMBCl (0.330 mL, 2.42 mmol), K_2CO_3 (333 mg, 2.41 mmol), and KI (24.8 mg, 0.149 mmol). The mixture was stirred at 60 °C for 2 h, and 20wt% aqueous NaOH (1.2 mL) was added. After being stirred at 60 °C for 2 h, the mixture was quenched with 1 M aqueous HCl (15 mL) and extracted with toluene (50 mL). The organic layer was washed with H_2O (30 mL×4) and saturated brine (20 mL), sequentially. The organic layer was dried and concentrated under reduced pressure. The residue was purified by recrystallization from toluene to provide 57.2 mg (33%) of **S20** as white crystals. mp 131-134 °C. TLC R_f 0.21-0.44 (EtOAc/toluene, 1:1). IR (KBr): 3200-2500, 2932, 1710 cm^{-1}. ^1H NMR (500 MHz, $(CD_3)_2SO$): δ 7.35 (d, 2H, *J* =8.8 Hz, H-2, 6 of Ar), 7.11 (d, 2H, *J* =8.8 Hz, H-2, 6 of PMB), 6.92 (d, 2H, *J* =8.8 Hz, H-3, 5 of Ar), 6.88 (d, 2H, *J* =8.8 Hz, H-3, 5 of PMB), 4.95 (s, 2H, Bn), 3.74 (s, 3H, -Me), 2.72 (t, 2H, *J* =7.6 Hz, H-3, 3’), 2.48 (t, 2H, *J* =7.6 Hz, H-2, 2’). $^{13}C\{^1H\}$ NMR (125 MHz, $(CD_3)_2SO$): δ 173.9, 158.9, 156.7, 132.9, 129.5 (2C), 129.2 (2C), 129.1, 114.6 (2C), 113.8 (2C), 68.9, 55.1, 35.6, 29.5. HRMS (ESI-TOF) *m*/*z*: $[M + H]^+$ Calcd for $C_{17}H_{19}O_{34}$ 287.1283; Found 287.1291.

3-[4-(*o*-Nitrobenzyloxy)phenyl]propionic acid (S21).

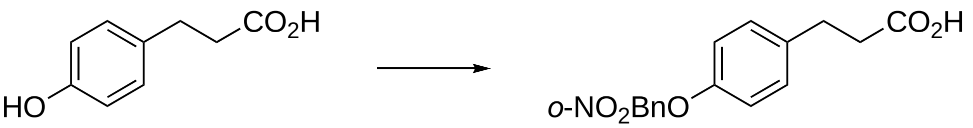

S15 **S21**

The following reaction was carried out under Ar. To a stirred solution of 3-(4-hydroxyphenyl)propionic acid (**S15**) (202 mg, 1.22 mmol) in DMF (2 mL) were added *o*-NO_2BnBr (986 mg, 4.56 mmol), K_2CO_3 (675 mg, 4.89 mmol), and KI (39.4 mg, 0.237 mmol). The mixture was stirred at 60 °C for 3 h, and 20wt% aqueous NaOH (2.4 mL) was added. After being stirred at 60 °C for 1 h, the mixture was quenched with 1 M aqueous HCl (30 mL) and extracted with $CHCl_3$ (10 mL×3). The combined extracts were extracted with 0.1 M aqueous NaOH (10 mL×2). The combined aqueous layers were acidified with 1 M aqueous HCl (6 mL) and extracted with $CHCl_3$ (30 mL). The organic layer was washed with saturated brine (10 mL), dried and concentrated under reduced pressure. The residue was purified by column chromatography on silica gel (EtOAc/toluene, 1:10) to provide 263 mg (72%) of **S21**

as yellow crystals. mp 124-126 °C. TLC R_f 0-0.33 (EtOAc/toluene, 1:4). IR (KBr): 3500-2500, 2919, 1708 cm^{-1}. ^{1}H NMR (500 MHz, $(CD_3)_2SO$): δ 8.11 (d, 1H, *J* =8.5 Hz, H-3 of *o*-NO_2Bn), 7.77 (s, 1H, *o*-NO_2Bn), 7.76 (s, 1H, *o*-NO_2Bn), 7.65 (m, 1H, *o*-NO_2Bn), 7.19 (d, 2H, *J* =8.5 Hz, H-2, 6 of Ar), 6.95 (d, 2H, *J* =8.5 Hz, H-3, 5 of Ar), 5.46 (s, 2H, *o*-NO_2Bn), 2.79 (t, 2H, *J* =7.5 Hz, H-3, 3'), 2.52 (t, 2H, *J* =7.5 Hz, H-2, 2'). $^{13}C\{^1H\}$ NMR (125 MHz, $(CD_3)_2SO$): δ 173.9, 156.2, 147.5, 134.0, 133.6, 132.7, 129.4 (2C), 129.2, 129.1, 124.8, 114.6 (2C), 66.3, 35.5, 29.5. HRMS (ESI-TOF) *m*/*z*: $[M + H]^+$ Calcd for $C_{16}H_{16}NO_5$ 302.1028; Found 302.1029.

***tert*-Butyl 3-(4-hydroxyphenyl)propionate (S22).**

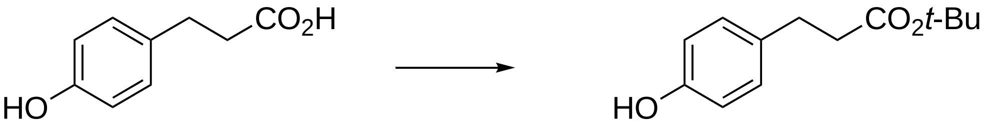

S15 **S22**

The following reaction was carried out under Ar. To a stirred solution of 3-(4-hydroxyphenyl)propionic acid (**S15**) (3.00 g, 18.1 mmol) in DMF (18 mL) was added CDI (3.53 g, 21.8 mmol). The mixture was stirred at 40 °C for 7 h, and dry *tert*-butyl alcohol (5.15mL, 54.1 mmol) and DBU (5.40 mL, 36.1 mmol) were added. After being stirred at 80 °C for 18 h, the mixture was diluted with H_2O (100 mL) and extracted with Et_2O (20 mL×10). The combined extracts were washed with saturated brine (20 mL), dried and concentrated under reduced pressure. The residue was purified by column chromatography on silica gel (EtOAc/hexane, 1:8) to provide 2.50 g (62%) of **S22** as colorless crystals. mp 38-42 °C. TLC R_f 0.72 (EtOAc/hexane, 1:2). IR (KBr): 3362, 2977, 1690 cm^{-1}. ^{1}H NMR (500 MHz, $CDCl_3$): δ 7.04 (d, 2H, *J* =8.5 Hz, H-2, 6 of Ar), 6.73 (d, 2H, *J* =8.5 Hz, H-3, 5 of Ar), 5.52 (br s, 1H, -OH), 2.83 (t, 2H, *J* =7.8 Hz, H-3, 3'), 2.51 (t, 2H, *J* =7.8 Hz, H-2, 2'), 1.42 (s, 9H, *t*-Bu). $^{13}C\{^1H\}$ NMR (125 MHz, $CDCl_3$): δ 172.8, 154.1, 132.6, 129.4 (2C), 115.2 (2C), 80.6, 37.4, 30.3, 28.0 (3C). HRMS (ESI-TOF) *m*/*z*: $[M + H]^+$ Calcd for $C_{13}H_{19}O_3$ 223.1334; Found 223.1338.

***tert*-Butyl 3-[4-(2-methoxyethoxymethoxy)phenyl]propionate (S23).**

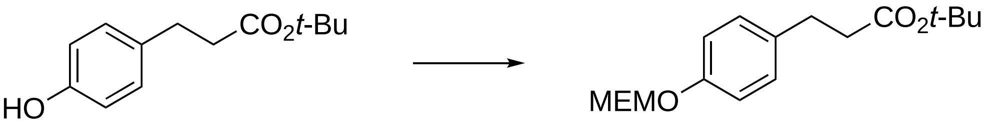

S22 **S23**

The following reaction was carried out under Ar. To a cooled (0 °C) stirred solution of **S22** (99.8 mg, 0.449 mmol) in THF (7 mL) was added NaH (60% in oil, 53.7 mg, 1.34 mmol). The mixture was stirred at 0 °C for 30 min, and MEMCl (0.130 mL, 1.14 mmol) was added at 0 °C. After being stirred at room temperature for 14 h, the mixture was diluted with saturated aqueous $NaHCO_3$ (20 mL) and extracted with CH_2Cl_2 (10 mL×3). The combined extracts were washed with H_2O (10 mL×3) and saturated brine (10 mL), sequentially. The organic layer was dried and concentrated under reduced pressure. The residue was purified by column

chromatography on silica gel (EtOAc/hexane, 1:10) to provide 117 mg (84%) of **S23** as a colorless oil. TLC R_f 0.56 (EtOAc/hexane, 1:4). IR (neat): 2977, 1729 cm^{-1}. ^{1}H NMR (500 MHz, $CDCl_3$): δ 7.10 (d, 2H, J =8.8 Hz, H-2, 6 of Ar), 6.96 (d, 2H, J =8.8 Hz, H-3, 5 of Ar), 5.24 (s, 2H, -OCH_2O-), 3.81 (t, 2H, J =4.8 Hz, -OCH_2CH_2O-), 3.55 (t, 2H, J =4.8 Hz, -OCH_2CH_2O-), 3.37 (s, 3H, -OCH_3), 2.84 (t, 2H, J =7.6 Hz, H-3, 3'), 2.50 (t, 2H, J =7.6 Hz, H-2, 2'), 1.41 (s, 9H, *t*-Bu). ^{13}C{^{1}H} NMR (125 MHz, $CDCl_3$): δ 172.3, 155.6, 134.2, 129.2 (2C), 116.2 (2C), 93.6, 80.3, 76.7, 67.5, 59.0, 37.3, 30.3, 28.0 (3C). HRMS (ESI-TOF) *m/z*: $[M + H]^+$ Calcd for $C_{17}H_{27}O_5$ 311.1858; Found 311.1854.

3-[4-(2-Methoxyethoxymethoxy)phenyl]propionic acid (S24).

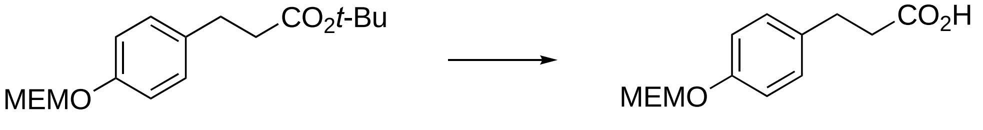

S23 **S24**

The following reaction was carried out under Ar. To a stirred solution of **S23** (117 mg, 0.378 mmol) in MeOH/H_2O (5:2, 6 mL) was added NaOH (95.5 mg, 2.39 mmol). After being stirred at room temperature for 15 h, the mixture was quenched with 1 M aqueous HCl (3 mL) and extracted with $CHCl_3$ (10 mL×3). The combined extracts were washed with saturated brine (20 mL), dried and concentrated under reduced pressure. The residue was purified by column chromatography on silica gel (EtOAc/hexane, 1:1) to provide 92.0 mg (96%) of **S24** as a colorless oil. TLC R_f 0-0.34 (EtOAc/hexane, 2:1). IR (neat): 3600-2500, 2926, 1711 cm^{-1}. ^{1}H NMR (500 MHz, $CDCl_3$): δ 7.12 (d, 2H, J =8.5 Hz, H-2, 6 of Ar), 6.98 (d, 2H, J =8.5 Hz, H-3, 5 of Ar), 5.24 (s, 2H, -OCH_2O-), 3.82 (t, 2H, J =4.6 Hz, -OCH_2CH_2O-), 3.56 (t, 2H, J =4.6 Hz, -OCH_2CH_2O-), 3.37 (s, 3H, -OCH_3), 2.90 (t, 2H, J =7.8 Hz, H-3, 3'), 2.64 (t, 2H, J =7.8 Hz, H-2, 2'). ^{13}C{^{1}H} NMR (125 MHz, $CDCl_3$): δ 178.3, 155.8, 133.6, 129.2 (2C), 116.4 (2C), 93.5, 76.7, 67.5, 59.0, 35.7, 29.8. HRMS (ESI-TOF) *m/z*: $[M + Na]^+$ Calcd for $C_{13}H_{18}O_5Na$ 277.1052; Found 277.1046.

***tert*-Butyl 3-[4-(4-pentenyloxy)phenyl]propionate (S25).**

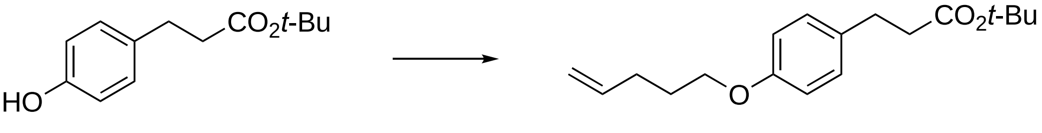

S22 **S25**

The following reaction was carried out under Ar. To a stirred solution of **S22** (257 mg, 1.15 mmol) in DMF (2 mL) were added K_2CO_3 (667 mg, 4.83 mmol), KI (40.4 mg, 0.243 mmol), and 5-bromo-1-pentene (0.550 mL, 4.65 mmol). After being stirred at 60 °C for 17 h, the mixture was diluted with H_2O (10 mL) and extracted with Et_2O (10 mL×3). The combined extracts were washed saturated brine (10 mL), dried and concentrated under reduced pressure. The residue was purified by column chromatography on silica gel (EtOAc/hexane, 1:20) to provide 309 mg (92%) of **S25** as a colorless oil. TLC R_f 0.69 (EtOAc/hexane, 1:4). IR (neat):

3500-2500, 2977, 1730 cm^{-1}. ^{1}H NMR (500 MHz, $CDCl_3$): δ 7.10 (d, 2H, *J* =8.8 Hz, H-2, 6 of Ar), 6.82 (d, 2H, *J* =8.8 Hz, H-3, 5 of Ar), 5.85 (m, 1H, H-4 of 4-pentenyloxy), 5.06 (d, 1H, *J* =17.0 Hz, H-5 of 4-pentenyloxy), 5.00 (d, 1H, *J* =10.6 Hz, H-5' of 4-pentenyloxy) , 3.94 (t, 2H, *J* =6.8 Hz, H-1, 1' of 4-pentenyloxy), 2.85 (t, 2H, *J* =7.9 Hz, H-3, 3') , 2.50 (t, 2H, *J* =7.9 Hz, H-2, 2'), 2.23 (q, 2H, *J* =6.8 Hz, H-3, 3' of 4-pentenyloxy), 1.87 (quin, 2H, *J* =6.8 Hz, H-2, 2' of 4-pentenyloxy), 1.42 (s, 9H, *t*-Bu). ^{13}C{^{1}H} NMR (125 MHz, $CDCl_3$): δ 172.3, 157.4, 137.8, 132.7, 129.2 (2C), 115.1, 114.4 (2C), 80.2, 67.1, 37.4, 30.2, 30.1, 28.4, 28.0 (3C). HRMS (ESI-TOF) *m*/*z*: [M + H]$^+$ Calcd for $C_{18}H_{27}O_3$ 291.1960; Found 291.1969.

3-[4-(4-Pentenyloxy)phenyl]propionic acid (S26).

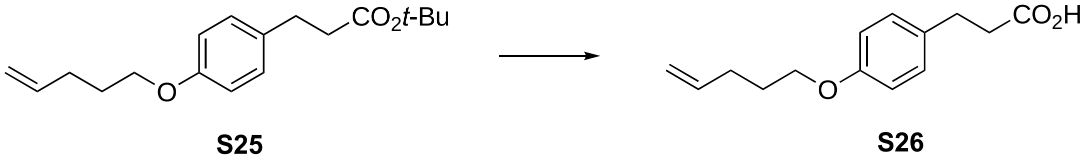

To a stirred solution of **S25** (196 mg, 0.673 mmol) in MeOH/H_2O (2:1, 2 mL) was added NaOH (95.5 mg, 2.46 mmol). After being refluxed for 2 h, the mixture was quenched with 1 M aqueous HCl (4 mL), diluted with H_2O (10 mL) and extracted with $CHCl_3$ (10 mL×3). The combined extracts were washed with saturated brine (20 mL), dried and concentrated under reduced pressure. The residue was purified by column chromatography on silica gel (EtOAc/hexane, 1:4) to provide 158 mg (quant.) of **S26** as white crystals. mp 68-69 °C. TLC R_f 0-0.25 (EtOAc/hexane, 1:4). IR (KBr): 2949, 1721 cm^{-1}. ^{1}H NMR (400 MHz, $CDCl_3$): δ 7.11 (d, 2H, *J* =8.8 Hz, H-2, 6 of Ar), 6.83 (d, 2H, *J* =8.8 Hz, H-3, 5 of Ar), 5.85 (m, 1H, H-4 of 4-pentenyloxy), 5.06 (d, 1H, *J* =17.2 Hz, H-5 of 4-pentenyloxy), 4.99 (d, 1H, *J* =10.2 Hz, H-5' of 4-pentenyloxy), 3.94 (t, 2H, *J* =6.8 Hz, H-1, 1' of 4-pentenyloxy), 2.90 (t, 2H, *J* =7.7 Hz, H-3, 3'), 2.65 (t, 2H, *J* =7.7 Hz, H-2, 2'), 2.23 (q, 2H, *J* =6.8 Hz, H-3, 3' of 4-pentenyloxy), 1.87 (quin, 2H, *J* =6.8 Hz, H-2, 2' of 4-pentenyloxy). ^{13}C{^{1}H} NMR (125 MHz, $CDCl_3$): δ 178.7, 157.6, 137.8, 132.1, 129.2 (2C), 115.1, 114.5 (2C), 67.2, 35.8, 30.1, 29.7, 28.4. HRMS (ESI-TOF) *m*/*z*: [M + H]$^+$ Calcd for $C_{14}H_{19}O_3$ 235.1334; Found 235.1325.

***tert*-Butyl 3-[4-(3-phenylpropioloxy)phenyl]propionate (S27).**

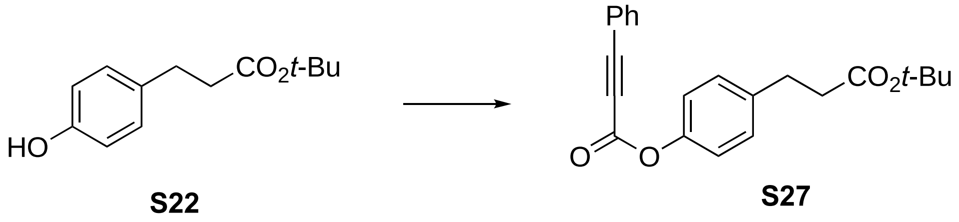

The following reaction was carried out under Ar. To a stirred solution of **S22** (227 mg, 1.02 mmol) in CH_2Cl_2 (1 mL) were added phenylpropiolic acid (157 mg, 1.07 mmol), EDCI·HCl (363 mg, 1.89 mmol), and DMAP (12.3 mg, 0.101 mmol). After being stirred at room temperature for 2 h, the mixture was diluted with CH_2Cl_2 (10 mL) and washed with 5wt% aqueous $NaHCO_3$ (10 mL×3), H_2O (10 mL×3) and saturated brine (10 mL), sequentially. The

organic layer was dried and concentrated under reduced pressure. The residue was purified by column chromatography on silica gel (EtOAc/hexane, 1:20) to provide 155 mg (43%) of **S27** as white crystals. mp 65-69 °C. TLC R_f 0.59 (EtOAc/hexane, 1:2). IR (KBr): 2970, 2234, 1720 cm^{-1}. ^{1}H NMR (500 MHz, $CDCl_3$): δ 7.83 (d, 2H, *J* =7.6 Hz, H-2, 6 of Ph), 7.49 (t, 1H, *J* =7.6 Hz, H-4 of Ph), 7.41 (t, 2H, *J* =7.6 Hz, H-3, 5 of Ph), 7.24 (d, 2H, *J* =8.8 Hz, H-2, 6 of Ar), 7.10 (d, 2H, *J* =8.8 Hz, H-3, 5 of Ar), 2.92 (t, 2H, *J* =7.8 Hz, H-3, 3') , 2.54 (t, 2H, *J* =7.8 Hz, H-2, 2'), 1.42 (s, 9H, *t*-Bu). ^{13}C{^{1}H} NMR (125 MHz, $CDCl_3$): δ 172.1, 152.5, 148.4, 139.0, 133.2 (2C), 131.0, 129.5 (2C), 128.7 (2C), 121.3 (2C), 119.3, 88.6, 80.5, 80.3, 36.9, 30.5, 28.1 (3C). HRMS (ESI-TOF) *m*/*z*: [M + H]$^+$ Calcd for $C_{22}H_{23}O_4$ 351.1596; Found 351.1595.

3-[4-(3-Phenylpropioloxy)phenyl]propionic acid (S28).

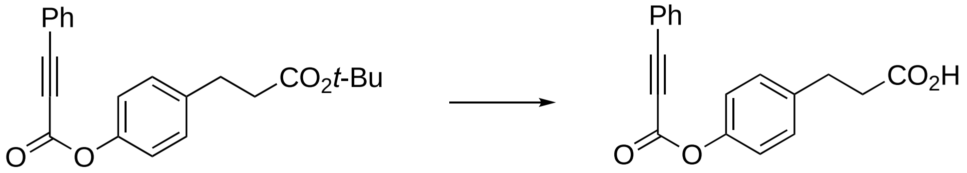

S27 **S28**

The following reaction was carried out under Ar. To a cooled (0 °C) stirred solution of **S27** (151 mg, 0.430 mmol) in CH_2Cl_2 (1 mL) was added TFA (1 mL). After being stirred at room temperature for 2 h, the mixture was concentrated under reduced pressure. The residue was purified by column chromatography on silica gel (EtOAc/hexane, 1:2) to provide 122 mg (97%) of **S28** as white crystals. mp 93-96 °C. TLC R_f 0-0.30 (EtOAc/hexane, 1:2). IR (KBr): 3200-2500, 2937, 2223, 1726, 1702 cm^{-1}. ^{1}H NMR (400 MHz, $CDCl_3$): δ 7.63 (d, 2H, *J* =7.3 Hz, H-2, 6 of Ph), 7.49 (t, 1H, *J* =7.3 Hz, H-4 of Ph), 7.41 (t, 2H, *J* =7.3 Hz, H-3, 5 of Ph), 7.26 (d, 2H, *J* =8.6 Hz, H-2, 6 of Ar), 7.12 (d, 2H, *J* =8.6 Hz, H-3, 5 of Ar), 2.98 (t, 2H, *J* =7.7 Hz, H-3, 3') , 2.70 (t, 2H, *J* =7.7 Hz, H-2, 2'). ^{13}C{^{1}H} NMR (100 MHz, $CDCl_3$): δ 177.7, 152.5, 148.6, 138.4, 133.2 (2C), 131.0, 129.4 (2C), 128.7 (2C), 121.5 (2C), 119.2, 88.7, 80.2, 35.3, 29.9. HRMS (ESI-TOF) *m*/*z*: [M + H]$^+$ Calcd for $C_{18}H_{15}O_4$ 295.0970; Found 295.0968.

***tert*-Butyl 3-[4-(5-carboxylpentyloxy)phenyl]propionate (S29).**

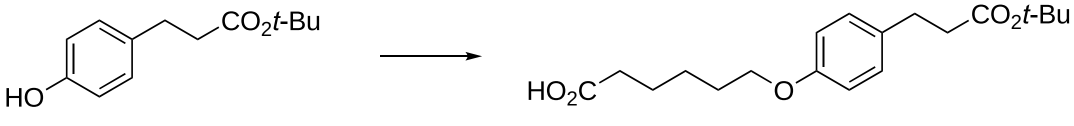

S22 **S29**

The following reaction was carried out under Ar. To a cooled (0 °C) stirred solution of **S22** (668 mg, 3.01 mmol) in DMF (9 mL) was added NaH (60% in oil, 300 mg, 7.50 mmol). The mixture was stirred at 0 °C for 1 h, and a solution of 6-bromohexanoic acid (586 mg, 3.00 mmol) in DMF (6 mL) was added at 0 °C. After being stirred at room temperature for 2 d, the mixture was acidified with 1 M aqueous HCl (6 mL) to pH2, diluted with EtOAc (20 mL) and

washed with H_2O (20 mL×3). The organic layer was dried and concentrated under reduced pressure. The residue was purified by column chromatography on silica gel (EtOAc/hexane, 1:8) to provide 865 mg (86%) of **S29** as white crystals. mp 39-42 °C. TLC R_f 0-0.40 (EtOAc/hexane, 1:2). IR (KBr): 3500-2500, 2942, 1725, 1713 cm^{-1}. ^{1}H NMR (500 MHz, $CDCl_3$): δ 7.09 (d, 2H, *J* =8.5 Hz, H-2, 6 of Ar), 6.80 (d, 2H, *J* =8.5 Hz, H-3, 5 of Ar), 3.93 (t, 2H, *J* =6.8 Hz, H-1, 1' of pentyloxy), 2.84 (t, 2H, *J* =7.8 Hz, H-3, 3'), 2.50 (t, 2H, *J* =7.8 Hz, H-2, 2'), 2.39 (t, 2H, *J* =7.6 Hz, H-5, 5' of pentyloxy), 1.79 (quin, 2H, *J* =6.8 Hz, H-2, 2' of pentyloxy), 1.71 (quin, 2H, *J* =7.6 Hz, H-4, 4' of pentyloxy), 1.57-1.48 (m, 2H, H-3, 3' of pentyloxy), 1.42 (s, 9H, *t*-Bu). ^{13}C{^{1}H} NMR (125 MHz, $CDCl_3$): δ 179.4, 172.4, 157.4, 132.8, 129.2 (2C), 114.4 (2C), 80.3, 67.6. 37.4, 33.9, 30.3, 28.9, 28.1 (3C), 25.6, 24.4. HRMS (ESI-TOF) *m*/*z*: $[M + H]^+$ Calcd for $C_{19}H_{29}O_5$ 337.2015; Found 337.2016.

tert-Butyl 3-[4-(5-(phthalimidyloxycarbonyl)pentyloxy)phenyl]propionate (S30).

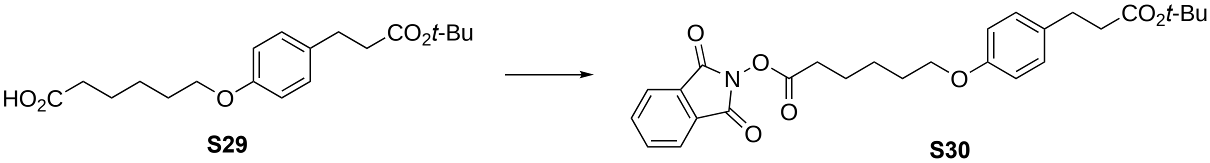

The following reaction was carried out under Ar. To a stirred solution of **S29** (1.92 g, 5.71 mmol) in CH_2Cl_2 (12 mL) were added *N*-hydroxyphthalimide (1.12 g, 6.87 mmol) and EDCI·HCl (1.64 g, 8.56 mmol). After being stirred at room temperature for 16 h, the mixture was diluted with CH_2Cl_2 (50 mL) and washed with saturated aqueous $NaHCO_3$ (20 mL×3), H_2O (20 mL×3) and saturated brine (20 mL), sequentially. The organic layer was dried and concentrated under reduced pressure. The residue was purified by column chromatography on silica gel (EtOAc/hexane, 1:5) to provide 2.58 g (94%) of **S30** as white crystals. mp 58-60 °C. TLC R_f 0.59 (EtOAc/hexane, 1:2). IR (KBr): 2943, 1819, 1787, 1741, 1698 cm^{-1}. ^{1}H NMR (500 MHz, $CDCl_3$): δ 7.92-7.86 (m, 2H, H-3, 6 of phthalimidyl), 7.82-7.77 (m, 2H, H-4, 5 of phthalimidyl), 7.10 (d, 2H, *J* =8.8 Hz, H-2, 6 of Ar), 6.82 (d, 2H, *J* =8.8 Hz, H-3, 5 of Ar), 3.96 (t, 2H, *J* =6.3 Hz, H-1, 1' of pentyloxy), 2.84 (t, 2H, *J* =7.9 Hz, H-3, 3'), 2.71 (t, 2H, *J* =7.5 Hz, H-5, 5' of pentyloxy), 2.50 (t, 2H, *J* =7.9 Hz, H-2, 2'), 1.91-1.80 (m, 4H, H-2, 2', 4, 4' of pentyloxy), 1.67-1.60 (m, 2H, H-3, 3' of pentyloxy), 1.42 (s, 9H, *t*-Bu). ^{13}C{^{1}H} NMR (125 MHz, $CDCl_3$): δ 172.4, 169.5, 162.0 (2C), 157.4, 134.7 (2C), 132.8, 129.2 (2C), 128.9 (2C), 124.0 (2C), 114.4 (2C), 80.2, 67.5, 37.4, 30.9, 30.3, 28.7, 28.1 (3C), 25.4, 24.4. HRMS (ESI-TOF) *m*/*z*: $[M + H]^+$ Calcd for $C_{27}H_{32}NO_7$ 482.2179; Found 482.2175.

3-[4-(5-(Phthalimidyloxycarbonyl)pentyloxy)phenyl]propionic acid (S31).

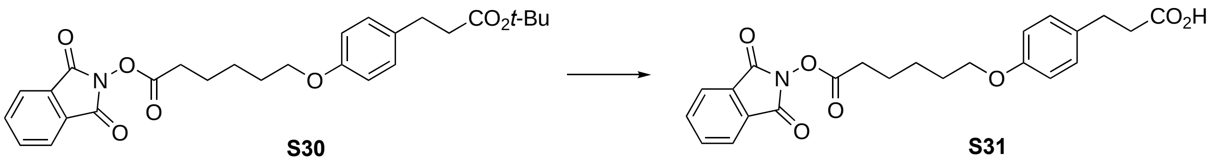

The following reaction was carried out under Ar. To a cooled (0 °C) stirred solution of **S30**

(2.58 g, 5.36 mmol) in CH_2Cl_2 (13 mL) was added TFA (13 mL). After being stirred at room temperature for 2 h, the mixture was concentrated under reduced pressure. The residue was purified by recrystallization from CH_2Cl_2/hexane to provide 2.12 g (93%) of **S31** as white crystals. mp 100-103 °C. TLC R_f 0-0.34 (EtOAc/hexane, 2:1). IR (KBr): 3400-2500, 2941, 1821, 1788, 1746 cm^{-1}. ^{1}H NMR (400 MHz, $CDCl_3$): δ 7.92-7.86 (m, 2H, H-3, 6 of phthalimidyl), 7.82-7.76 (m, 2H, H-4, 5 of phthalimidyl), 7.11 (d, 2H, *J* =8.4 Hz, H-2, 6 of Ar), 6.83 (d, 2H, *J* =8.4 Hz, H-3, 5 of Ar), 3.97 (t, 2H, *J* =6.4 Hz, H-1, 1' of pentyloxy), 2.90 (t, 2H, *J* =7.8 Hz, H-3, 3'), 2.71 (t, 2H, *J* =7.4 Hz, H-5, 5' of pentyloxy), 2.65 (t, 2H, *J* =7.8 Hz, H-2, 2'), 1.92-1.80 (m, 4H, H-2, 2', 4, 4' of pentyloxy), 1.69-1.58 (m, 2H, H-3, 3' of pentyloxy). ^{13}C{^{1}H} NMR (125 MHz, $CDCl_3$): δ 178.5, 169.5, 162.0 (2C), 157.5, 134.7 (2C), 132.1, 129.2 (2C), 128.9 (2C), 123.9 (2C), 114.6 (2C), 67.5, 35.8, 30.9, 29.8, 28.8, 25.4, 24.4. HRMS (ESI-TOF) *m*/*z*: $[M + H]^+$ Calcd for $C_{23}H_{24}NO_7$ 426.1553; Found 426.1546.

***tert*-Butyl 3-[4-(5-(3,4,5,6-tetrachlorophthalimidyloxycarbonyl)pentyloxy)phenyl]propionate (S32).**

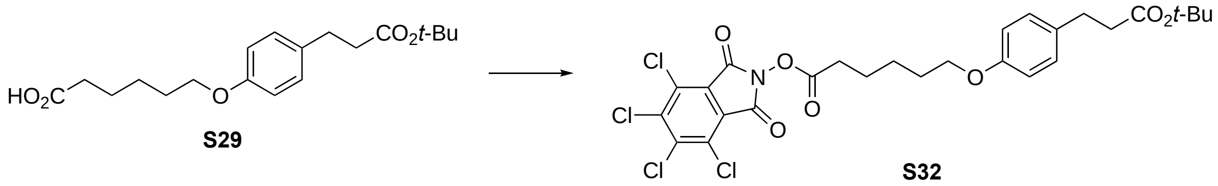

The following reaction was carried out under Ar. To a stirred solution of **S29** (363 mg, 1.08 mmol) in CH_2Cl_2 (2 mL) were added *N*-hydroxytetrachlorophthalimide (300 mg, 0.998 mmol) and EDCI·HCl (313 mg, 1.63 mmol). After being stirred at room temperature for 14 h, the mixture was diluted with CH_2Cl_2 (30 mL) and washed with H_2O (20 mL×5) and saturated brine (20 mL), sequentially. The organic layer was dried and concentrated under reduced pressure. The residue was purified by column chromatography on silica gel (EtOAc/hexane, 1:10) to provide 360 mg (58%) of **S32** as white solids. TLC R_f 0.80 (EtOAc/hexane, 1:2). IR (KBr): 2932, 1821, 1794, 1749, 1727 cm^{-1}. ^{1}H NMR (500 MHz, $CDCl_3$): δ 7.10 (d, 2H, *J* =8.8 Hz, H-2, 6 of Ar), 6.81 (d, 2H, *J* =8.8 Hz, H-3, 5 of Ar), 3.96 (t, 2H, *J* =6.5 Hz, H-1, 1' of pentyloxy), 2.84 (t, 2H, *J* =7.6 Hz, H-3, 3'), 2.71 (t, 2H, *J* =7.5 Hz, H-5, 5' of pentyloxy), 2.50 (t, 2H, *J* =7.6 Hz, H-2, 2'), 1.91-1.80 (m, 4H, H-2, 2', 4, 4' of pentyloxy), 1.67-1.59 (m, 2H, H-3, 3' of pentyloxy), 1.42 (s, 9H, *t*-Bu). ^{13}C{^{1}H} NMR (125 MHz, $CDCl_3$): δ 172.4, 169.0, 157.6 (2C), 157.3, 141.0 (2C), 132.8, 130.5 (2C), 129.2 (2C), 124.7 (2C), 114.4 (2C), 80.3, 67.4, 37.4, 30.8, 30.3, 28.8, 28.1 (3C), 25.4, 24.5. HRMS (ESI-TOF) *m*/*z*: $[M + H]^+$ Calcd for $C_{27}H_{28}Cl_4NO_7$ 618.0620; Found 618.0620.

3-[4-(5-(3,4,5,6-Tetrachlorophthalimidyloxycarbonyl)pentyloxy)phenyl]propionic acid (S33).

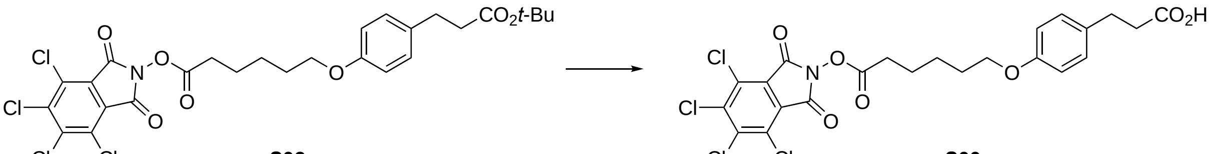

The following reaction was carried out under Ar. To a cooled (0 °C) stirred solution of **S32** (346 mg, 0.559 mmol) in CH_2Cl_2 (1 mL) was added TFA (1 mL). After being stirred at room temperature for 2 h, the mixture was concentrated under reduced pressure. The residue was purified by recrystallization from CH_2Cl_2/hexane to provide 272 mg (86%) of **S33** as yellow crystals. mp 124-127 °C. TLC R_f 0-0.33 (EtOAc/hexane, 1:2). IR (KBr): 3500-2500, 2949, 1822, 1794, 1747, 1708 cm^{-1}. ^{1}H NMR (400 MHz, $CDCl_3$): δ 7.11 (d, 2H, *J* =8.4 Hz, H-2, 6 of Ar), 6.83 (d, 2H, *J* =8.4 Hz, H-3, 5 of Ar), 3.96 (t, 2H, *J* =6.2 Hz, H-1, 1' of pentyloxy), 2.90 (t, 2H, *J* =7.7 Hz, H-3, 3'), 2.71 (t, 2H, *J* =7.4 Hz, H-5, 5' of pentyloxy), 2.65 (t, 2H, *J* =7.7 Hz, H-2, 2'), 1.92-1.80 (m, 4H, H-2, 2', 4, 4' of pentyloxy), 1.69-1.58 (m, 2H, H-3, 3' of pentyloxy). ^{13}C{^{1}H} NMR (100 MHz, $CDCl_3$): δ 177.7, 169.0, 157.6 (3C), 141.0 (2C), 132.2, 130.5 (2C), 129.2 (2C), 124.7 (2C), 114.5 (2C), 67.4, 35.7, 30.8, 29.8, 28.8, 25.4, 24.5. HRMS (ESI-TOF) *m/z*: $[M + H]^+$ Calcd for $C_{23}H_{20}Cl_4NO_7$ 561.9994; Found 561.9995.

3-*O*-Benzoyl-lithocholic acid (S35).

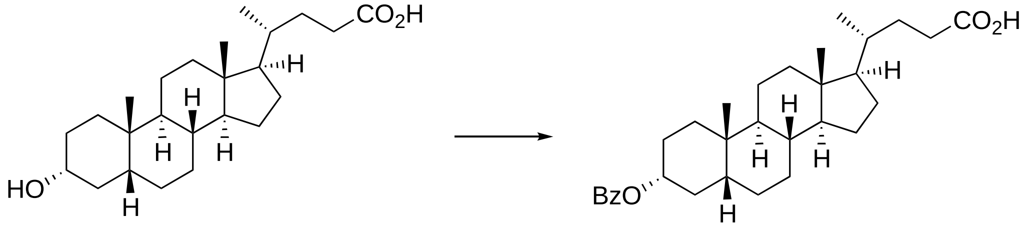

The following reaction was carried out under Ar. To a stirred solution of lithocholic acid (202 mg, 0.536 mmol) in THF (8 mL) were added benzoyl chloride (55.0 μL, 0.473 mmol) and pyridine (106 μL, 1.32 mmol). After being stirred at 70 °C for 13 h, the mixture was acidified with 1 M aqueous HCl to pH2, diluted with H_2O (20 mL) and extracted with EtOAc (20 mL×2). The combined extracts were washed with saturated brine (20 mL), dried and concentrated under reduced pressure. The residue was purified by column chromatography on silica gel (EtOAc/hexane, 1:10) to provide 90.4 mg (40%) of **S35** as white solids. TLC R_f 0.32-0.65 (EtOAc/hexane, 1:1). IR (KBr): 3600-2500, 2939, 1715 cm^{-1}. ^{1}H NMR (500 MHz, $CDCl_3$): δ 8.05 (d, 2H, *J* =7.8 Hz, H-2, 6 of Bz), 7.54 (t, 1H, *J* =7.8 Hz, H-4 of Bz), 7.43 (t, 2H, *J* =7.8 Hz, H-3, 5 of Bz), 4.97 (m, 1H, H-3), 2.40 (m, 1H), 2.26 (m, 1H), 2.03-1.93 (m, 2H), 1.92-1.76 (m, 5H), 1.68 (m, 1H), 1.63-1.50 (m, 3H) , 1.49-1.39 (m, 5H), 1.35 (m, 1H), 1.31-1.25 (m, 3H), 1.19 (m, 1H), 1.15-1.04 (m, 5H) 0.96 (s, 3H, H-19), 0.93 (d, 3H, *J* =6.5 Hz, H-21), 0.66 (s, 3H, H-18). ^{13}C{^{1}H} NMR (125 MHz, $CDCl_3$): δ 180.1, 166.2, 132.7, 130.9, 129.5 (2C), 128.2 (2C), 75.0, 56.5, 56.0, 42.7, 41.9, 40.5, 40.1, 35.8, 35.3, 35.1, 34.6, 32.3, 31.0, 30.8, 28.2, 27.0, 26.7, 26.3, 24.2, 23.4, 20.9, 18.2, 12.0. HRMS (ESI-TOF) *m/z*: $[M + H]^+$ Calcd for $C_{31}H_{45}O_4$ 481.3318; Found 481.3329.

O-Acetyl-mycophenolic acid (S37).

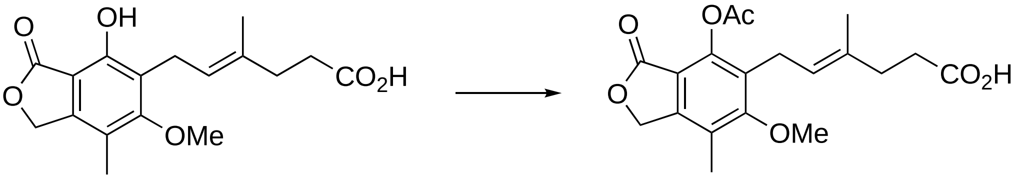

S36 **S37**

The following reaction was carried out under Ar. To a cooled (0 °C) stirred solution of mycophenolic acid (200 mg, 0.624 mmol) in pyridine (1 mL) were added Ac_2O (0.588 mL, 6.24 mmol) and DMAP (1.3 mg, 0.011 mmol). After being stirred at 0 °C for 2 h, the mixture was poured onto crushed ice (12 g), acidified with 1 M aqueous HCl (15 mL) to pH2 and extracted with EtOAc (15 mL×3). The combined extracts were washed with saturated brine (15 mL), dried and concentrated under reduced pressure. The residue was purified by column chromatography on silica gel (EtOAc/hexane/AcOH, 100:100:1) to provide 196 mg (87%) of **S37** as white crystals. mp 124-126 °C. TLC R_f 0.63 (EtOAc/hexane, 2:1). IR (KBr): 3400-2500, 2896, 1765, 1723 cm^{-1}. ^{1}H NMR (400 MHz, $CDCl_3$): δ 5.17-5.09 (m, 3H, C=C*H*, CO_2CH_2), 3.78 (s, 3H, OMe), 3.35 (d, 2H, J =6.0 Hz, C=CH-C*H*$_2$), 2.44-2.37 (m, 2H, CO_2H-C*H*$_2$), 2.39 (s, 3H, OAc), 2.29 (t, 2H, J =7.0 Hz, CO_2H-CH_2-C*H*$_2$), 2.22 (s, 3H, Ar-C*H*$_3$), 1.78 (s, 3H, C*H*$_3$-C=CH). $^{13}C\{^{1}H\}$ NMR (125 MHz, $CDCl_3$): δ 178.0, 169.1, 168.3, 162.6, 146.2, 145.9, 134.2, 129.1, 123.1, 122.4, 113.5, 68.4, 61.2, 34.1, 32.4, 23.5, 20.5, 16.2, 11.8. HRMS (ESI-TOF) m/z: $[M + H]^+$ Calcd for $C_{19}H_{23}O_7$ 363.1444; Found 363.1435.

2,7-Di-O-acetyl-gibberellic acid (S39).

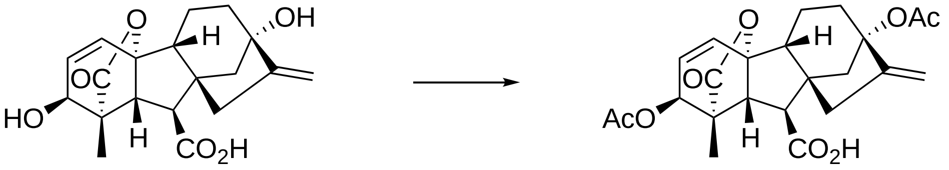

S38 **S39**

The following reaction was carried out under Ar. To a stirred solution of gibberellic acid (199 mg, 0.576 mmol) in pyridine (0.9 mL) were added Ac_2O (0.544 mL, 5.77 mmol) and DMAP (2.2 mg, 0.018 mmol). After being stirred at room temperature for 16 h, the mixture was acidified with 1 M aqueous HCl (2 mL) to pH2, diluted with CH_2Cl_2 (10 mL) and washed with 1 M aqueous HCl (2 mL×3). The organic layer was washed with saturated brine (10 mL), dried and concentrated under reduced pressure. The residue was purified by column chromatography on silica gel (EtOAc/hexane/AcOH, 100:100:1) to provide 196 mg (79%) of **S39** as white crystals. mp 168-170 °C. TLC R_f 0-0.63 (EtOAc/hexane, 2:1). $[\alpha]^{26}_{D}$ +157 (c 1.00, $CHCl_3$). IR (KBr): 3700-2900, 2941, 1781, 1741 cm^{-1}. ^{1}H NMR (400 MHz, $CDCl_3$): δ 6.37 (d, 1H, J =9.8 Hz, H-4), 5.87 (dd, 1H, J =9.8, 3.8 Hz, H-3), 5.33 (d, 1H, J =3.8 Hz, H-2), 5.17 (s, 1H, H-12), 5.02 (s, 1H, H-12'), 3.27 (d, 1H, J =11.0 Hz, H-10a), 2.81 (d, 1H, J =11.0 Hz, H-10), 2.50-2.34 (m, 3H), 2.28 (d, 1H, J =10.8 Hz), 2.20 (d, 1H, J =10.8 Hz), 2.13 (s, 3H,

OAc), 2.03 (s, 3H, OAc), 2.02-1.91 (m, 2H), 1.81 (m, 1H), 1.71 (m, 1H), 1.19 (s, 3H, H-14). $^{13}C\{^1H\}$ NMR (100 MHz, $CDCl_3$): δ 176.9, 176.1, 170.1 (2C), 153.2, 134.1, 129.2, 108.4, 89.9, 84.1, 70.2, 53.1, 52.1, 51.0, 50.8, 50.1, 42.4, 39.5, 36.4, 22.0, 20.8, 16.8, 14.4. HRMS (ESI-TOF) *m*/*z*: $[M + H]^+$ Calcd for $C_{23}H_{27}O_8$ 431.1706; Found 431.1715.

***trans*-4-Benzyloxycarbonylaminocyclohexanecarboxylic acid, 2-thioxopyridinyl ester (1a).**

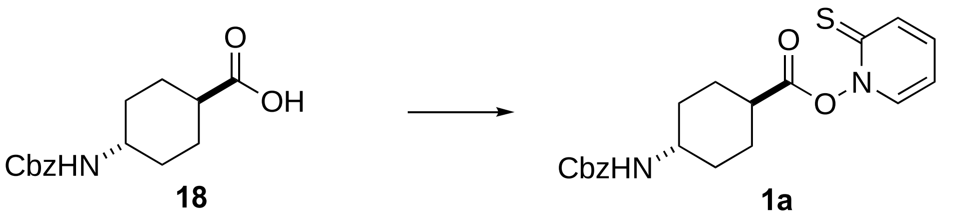

The following reaction was carried out under Ar. To a stirred solution of **18** (1.17 g, 4.23 mmol) in CH_2Cl_2 (14 mL) were added oxalyl chloride (0.402 mL, 4.66 mmol) and DMF (0.02 mL, 0.3 mmol). The mixture was stirred at room temperature for 30 min, and the flask was protected from light with aluminum foil. Then 2-mercaptopyridine *N*-oxide sodium salt (698 mg, 4.68 mmol) was added. After being stirred at room temperature for 1 h, the mixture was filtered and the filtrate was concentrated under reduced pressure. The residue was purified by column chromatography on silica gel (EtOAc/hexane, 4:1) to provide 682 mg (42%) of **1a** as yellow crystals. mp 132-136 °C. TLC R_f 0.72 (EtOAc/hexane, 4:1). IR (KBr): 3311, 2926, 1781, 1685 cm^{-1}. ^1H NMR (400 MHz, $CDCl_3$): δ 7.68 (d, 1H, *J* =8.7 Hz, H-6 of 2-thioxopyridinyl), 7.53 (d, 1H, *J* =7.1 Hz, H-3 of 2-thioxopyridinyl), 7.39-7.30 (m, 5H, -Ph of Cbz), 7.20 (dd, 1H, *J* = 8.7, 7.1 Hz, H-5 of 2-thioxopyridinyl), 6.63 (t, 1H, *J* = 7.1 Hz, H-4 of 2-thioxopyridinyl), 5.09 (s, 2H, $-CH_2-$ of Cbz), 4.65 (d, 1H, *J* = 7.2 Hz, -NH-), 3.56 (m, 1H, H-4), 2.69 (t, 1H, *J* =12.6 Hz, H-1), 2.31 (br d, 2H, *J* = 12.6 Hz, H_{eq}-2, 6), 2.18 (br d, 2H, *J* = 12.6 Hz, H_{eq}-3, 5), 1.76 (q, 2H, *J* = 12.6 Hz, H_{ax}-2, 6), 1.23 (q, 2H, *J* = 12.6 Hz, H_{ax}-3, 5). $^{13}C\{^1H\}$ NMR (125 MHz, $CDCl_3$): δ 175.8, 170.6, 155.5, 137.5 (2C), 136.4, 133.5, 128.5 (3C), 128.1 (2C), 112.6, 66.7, 49.1, 40.3, 32.1 (2C), 27.6 (2C). HRMS (ESI-TOF) *m*/*z*: $[M + H]^+$ Calcd for $C_{20}H_{23}N_2O_4S$ 387.1379; Found 387.1366.

3-[4-(*tert*-Butyldimethylsilyloxy)phenyl]propionic acid, 2-thioxopyridinyl ester (1b).

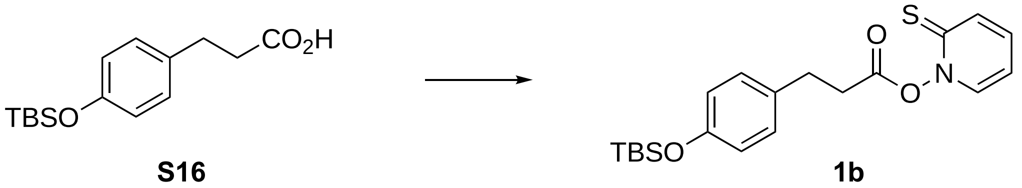

The following reaction was carried out under Ar and in the flask protected from light with aluminum foil. To a stirred solution of 2-mercaptopyridine *N*-oxide (45.9 mg, 0.361 mmol) and EDCI·HCl (81.1 mg, 0.423 mmol) in CH_2Cl_2 (0.4 mL) was added a solution of **S16** (59.8 mg, 0.213 mmol) in CH_2Cl_2 (0.6 mL). After being stirred at room temperature for 1 h, the mixture was diluted with CH_2Cl_2 (10 mL) and washed with 5wt% aqueous $NaHCO_3$ (10

mL×3), H_2O (10 mL×3) and saturated brine (10 mL), sequentially. The organic layer was dried and concentrated under reduced pressure. The residue was purified by column chromatography on silica gel (EtOAc/hexane, 1:4) to provide 53.1 mg (64%) of **1b** as a yellow oil. TLC R_f 0.67 (EtOAc/hexane, 1:1). IR (neat): 2955, 2930, 1809 cm^{-1}. ^1H NMR (400 MHz, $CDCl_3$): δ 7.69 (d, 1H, *J* =8.8 Hz, H-6 of 2-thioxopyridinyl), 7.39 (d, 1H, *J* =6.7 Hz, H-3 of 2-thioxopyridinyl), 7.20 (dd, 1H, *J* =8.8, 6.7 Hz, H-5 of 2-thioxopyridinyl), 7.11 (d, 2H, *J* =8.2 Hz, H-2, 6 of Ar), 6.79 (d, 2H, *J* =8.2 Hz, H-3, 5 of Ar), 6.61 (t, 1H, *J* = 6.7 Hz, H-4 of 2-thioxopyridinyl), 3.10-2.98 (m, 4H, H-2, 2', 3, 3'), 0.98 (s, 9H, *t*-Bu), 0.19 (s, 6H, -Me). $^{13}C\{^1H\}$ NMR (100 MHz, $CDCl_3$): δ 175.8, 168.3, 154.3, 137.6, 137.4, 133.5, 131.9, 129.3 (2C), 120.2 (2C), 112.6, 33.6, 29.6, 25.7 (3C), 18.2, -4.4 (2C). HRMS (ESI-TOF) *m*/*z*: $[M + H]^+$ Calcd for $C_{20}H_{28}NO_3SSi$ 390.1559; Found 390.1562.

3-[4-(Pivaloyloxy)phenyl]propionic acid, 2-thioxopyridinyl ester (1c).

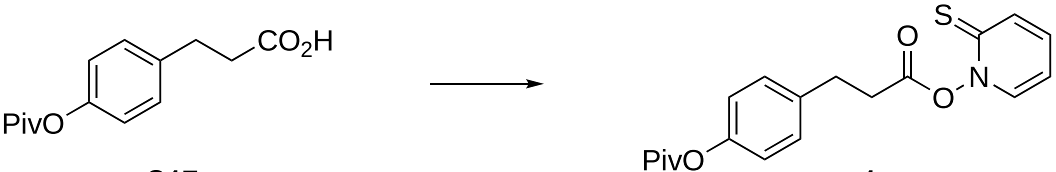

As described for the preparation of **1b**, compound **S17** (49.8 mg, 0.199 mmol) was converted to 34.9 mg (49%) of **1c**. Compound **1c** was obtained as a yellow oil. TLC R_f 0.67 (EtOAc/hexane, 1:1). IR (neat): 3026, 1808, 1745 cm^{-1}. ^1H NMR (500 MHz, $CDCl_3$): δ 7.68 (dd, 1H, *J* =8.6, 1.7 Hz, H-6 of 2-thioxopyridinyl), 7.35 (dd, 1H, *J* =6.9, 1.6 Hz, H-3 of 2-thioxopyridinyl), 7.28 (d, 2H, *J* =8.8 Hz, H-2, 6 of Ar), 7.19 (ddd, 1H, *J* =8.6, 6.9, 1.6 Hz, H-5 of 2-thioxopyridinyl), 7.00 (d, 2H, *J* =8.8 Hz, H-3, 5 of Ar), 6.61 (td, 1H, *J* = 6.9, 1.7 Hz, H-4 of 2-thioxopyridinyl), 3.14 (t, 2H, *J* =7.4 Hz, H-3, 3'), 3.04 (t, 2H, *J* =7.4 Hz, H-2, 2'), 1.35 (s, 9H, *t*-Bu). $^{13}C\{^1H\}$ NMR (125 MHz, $CDCl_3$): δ 177.2, 175.8, 168.0, 149.8, 137.7, 137.3, 136.5, 133.6, 129.4 (2C), 121.7 (2C), 112.7, 39.0, 33.3, 29.8, 27.1 (3C). HRMS (ESI-TOF) *m*/*z*: $[M + H]^+$ Calcd for $C_{19}H_{22}NO_4S$ 360.1270; Found 360.1261.

3-[4-(2-Methoxyethoxymethoxy)phenyl]propionic acid, 2-thioxopyridinyl ester (1d).

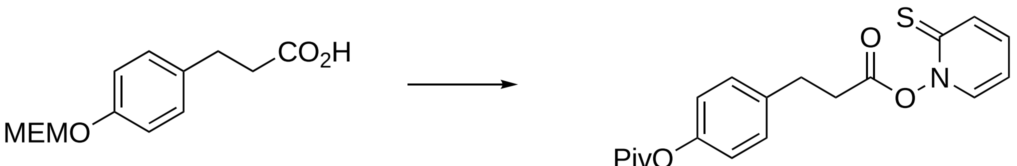

As described for the preparation of **1b**, compound **S24** (43.6 mg, 0.171 mmol) was converted to 48.3 mg (78%) of **1d**. Compound **1d** was obtained as a yellow oil. TLC R_f 0.25 (EtOAc/hexane, 3:1). IR (neat): 2928, 1808 cm^{-1}. ^1H NMR (500 MHz, $CDCl_3$): δ 7.69 (dd, 1H, *J* =9.0, 1.8 Hz, H-6 of 2-thioxopyridinyl), 7.44 (dd, 1H, *J* =7.2, 1.8 Hz, H-3 of 2-thioxopyridinyl), 7.22-7.16 (m, 3H, H-5 of 2-thioxopyridinyl, H-2, 6 of Ar), 7.01 (d, 2H, *J*

=8.5 Hz, H-3, 5 of Ar), 6.61 (td, 1H, *J* = 7.2, 1.8 Hz, H-4 of 2-thioxopyridinyl), 5.26 (s, 2H, -OCH_2O-), 3.84-3.80 (m, 2H, -OCH_2CH_2O-), 3.58-3.54 (m, 2H, -OCH_2CH_2O-), 3.38 (s, 3H, -OCH_3), 3.09 (t, 2H, *J* =7.0 Hz, H-3, 3'), 3.01 (t, 2H, *J* =7.0 Hz, H-2, 2'). $^{13}C\{^1H\}$ NMR (125 MHz, $CDCl_3$): δ 175.8, 168.3, 156.0, 137.6, 137.4, 133.5, 132.7, 129.4 (2C), 116.5 (2C), 112.6, 93.6, 71.6, 67.6, 59.0, 33.5, 29.5. HRMS (ESI-TOF) *m*/*z*: $[M + H]^+$ Calcd for $C_{18}H_{22}NO_5S_2$ 364.1219; Found 364.1215.

3-[4-(Methanesulfonyloxy)phenyl]propionic acid, 2-thioxopyridinyl ester (1e).

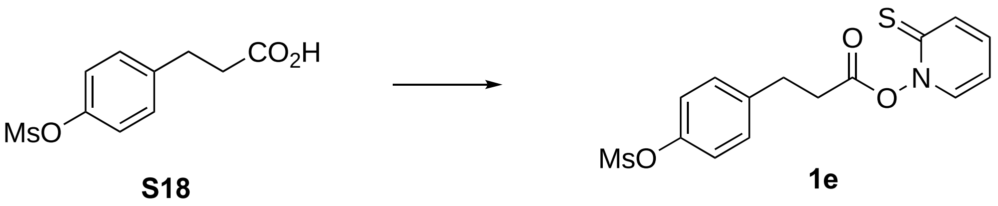

As described for the preparation of **1b**, compound **S18** (49.6 mg, 0.203 mmol) was converted to 55.1 mg (76%) of **1e**. Compound **1e** was obtained as yellow crystals. mp 64-68 °C. TLC R_f 0.52 (EtOAc/hexane, 4:1). IR (KBr): 2931, 1807 cm^{-1}. ^1H NMR (400 MHz, $CDCl_3$): δ 7.68 (dd, 1H, *J* =9.0, 1.6 Hz, H-6 of 2-thioxopyridinyl), 7.44 (dd, 1H, *J* =7.2, 1.2 Hz, H-3 of 2-thioxopyridinyl), 7.32 (d, 2H, *J* =8.8 Hz, H-2, 6 of Ar), 7.25-7.18 (m, 3H, H-5 of 2-thioxopyridinyl, H-3, 5 of Ar), 6.61 (td, 1H, *J* = 7.2, 1.6 Hz, H-4 of 2-thioxopyridinyl), 3.18-3.12 (m, 2H, H-3, 3'), 3.15 (s, 3H, -Me), 2.69 (t, 2H, *J* =7.4 Hz, H-2, 2'). $^{13}C\{^1H\}$ NMR (125 MHz, $CDCl_3$): δ 175.7, 168.0, 147.9, 138.7, 137.5, 137.3, 133.6, 130.1 (2C), 122.3 (2C), 112.7, 37.4, 33.1, 29.6. HRMS (ESI-TOF) *m*/*z*: $[M + H]^+$ Calcd for $C_{15}H_{16}NO_5S_2$ 354.0470; Found 354.0461.

3-[4-(Benzyloxy)phenyl]propionic acid, 2-thioxopyridinyl ester (1f).

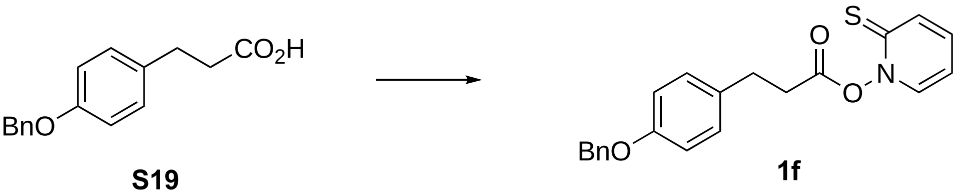

As described for the preparation of **1b**, compound **S19** (49.6 mg, 0.194 mmol) was converted to 36.7 mg (52%) of **1f**. Compound **1f** was obtained as yellow crystals. mp 101-104 °C. TLC R_f 0.47 (EtOAc/hexane, 1:1). IR (KBr): 3068, 1806 cm^{-1}. ^1H NMR (500 MHz, $CDCl_3$): δ 7.68 (dd, 1H, *J* =8.5, 1.9 Hz, H-6 of 2-thioxopyridinyl), 7.43 (d, 2H, *J* =7.4 Hz, H-2, 6 of Ph), 7.42-7.36 (m, 3H, H-3 of 2-thioxopyridinyl, H-3, 5 of Ph), 7.33 (t, 1H, *J* =7.4 Hz, H-4 of Ph), 7.21-7.16 (m, 3H, H-5 of 2-thioxopyridinyl, H-2, 6 of Ar), 6.94 (d, 2H, *J* =9.0 Hz, H-3, 5 of Ar), 6.59 (td, 1H, *J* = 6.9, 1.9 Hz, H-4 of 2-thioxopyridinyl), 5.06 (s, 2H, Bn), 3.08 (t, 2H, *J* =6.9 Hz, H-3, 3'), 3.01 (t, 2H, *J* =6.9 Hz, H-2, 2'). $^{13}C\{^1H\}$ NMR (125 MHz, $CDCl_3$): δ 175.8, 168.3, 157.6, 137.6, 137.4, 137.0, 133.5, 131.6, 129.4 (2C), 128.6 (2C), 127.9, 127.4 (2C), 115.0 (2C), 112.5, 70.0, 33.6, 29.5. HRMS (ESI-TOF) *m*/*z*: $[M + H]^+$

Calcd for $C_{21}H_{20}NO_3S$ 366.1164; Found 366.1168.

3-[4-(*p*-Methoxybenzyloxy)phenyl]propionic acid, 2-thioxopyridinyl ester (1g).

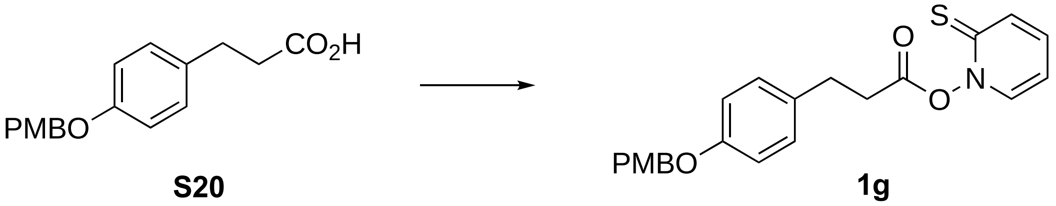

As described for the preparation of **1b**, compound **S20** (85.5 mg, 0.299 mmol) was converted to 52.2 mg (44%) of **1g**. Compound **1g** was obtained as yellow crystals. mp 105-109 °C. TLC R_f 0.45 (EtOAc/hexane, 1:1). IR (KBr): 2931, 1808 cm^{-1}. ^1H NMR (500 MHz, $CDCl_3$): δ 7.68 (dd, 1H, *J* =8.5, 1.9 Hz, H-6 of 2-thioxopyridinyl), 7.40 (dd, 1H, *J* =7.1, 1.8 Hz, H-3 of 2-thioxopyridinyl), 7.36 (d, 2H, *J* =9.0 Hz, H-2, 6 of PMB), 7.22-7.16 (m, 3H, H-5 of 2-thioxopyridinyl, H-2, 6 of Ar), 6.94-6.90 (m, 4H, H-3, 5 of Ar, H-3, 5 of PMB), 6.59 (td, 1H, *J* = 6.1, 1.9 Hz, H-4 of 2-thioxopyridinyl), 4.98 (s, 2H, PMB), 3.82 (s, 3H, -OMe), 3.08 (t, 2H, *J* =7.6 Hz, H-3, 3’), 3.01 (t, 2H, *J* =7.6 Hz, H-2, 2’). $^{13}C\{^1H\}$ NMR (125 MHz, $CDCl_3$): δ 175.8, 168.3, 159.4, 157.6, 137.6, 137.4, 133.5, 131.5, 129.4 (2C), 129.2 (2C), 129.0, 115.0 (2C), 114.0 (2C), 112.6, 69.8, 55.3, 33.6, 29.5. HRMS (ESI-TOF) *m*/*z*: $[M + H]^+$ Calcd for $C_{22}H_{22}NO_4S$ 396.1270; Found 396.1276.

3-[4-(*o*-Nitrobenzyloxy)phenyl]propionic acid, 2-thioxopyridinyl ester (1h).

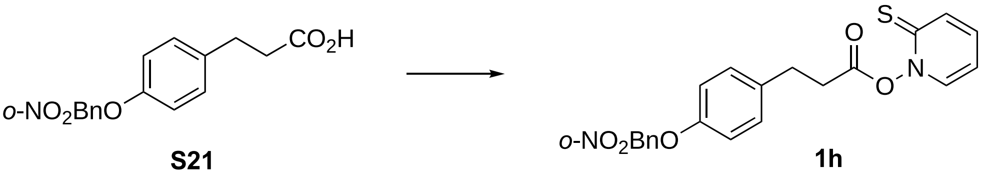

As described for the preparation of **1b**, compound **S21** (50.4 mg, 0.167 mmol) was converted to 51.7 mg (76%) of **1h**. Compound **1h** was obtained as yellow crystals. mp 99-101 °C. TLC R_f 0.55 (EtOAc/hexane, 1:1). IR (KBr): 3025, 1807 cm^{-1}. ^1H NMR (500 MHz, $CDCl_3$): δ 8.17 (d, 1H, *J* =8.0 Hz, H-3 of *o*-NO_2Bn), 7.89 (d, 1H, *J* =8.0 Hz, H-6 of *o*-NO_2Bn), 7.71-7.66 (m, 2H, H-6 of 2-thioxopyridinyl, H-5 of *o*-NO_2Bn), 7.49 (t, 1H, *J* =8.0 Hz, H-4 of *o*-NO_2Bn), 7.43 (dd, 1H, *J* =7.1, 1.3 Hz, H-3 of 2-thioxopyridinyl), 7.22-7.16 (m, 3H, H-5 of 2-thioxopyridinyl, H-2, 6 of Ar), 6.94 (d, 2H, *J* =8.5 Hz, H-3, 5 of Ar), 6.59 (td, 1H, *J* =7.1, 1.8 Hz, H-4 of 2-thioxopyridinyl), 5.47 (s, 2H, *o*-NO_2Bn), 3.09 (t, 2H, *J* =7.1 Hz, H-3, 3’), 3.01 (t, 2H, *J* =7.1 Hz, H-2, 2’). $^{13}C\{^1H\}$ NMR (125 MHz, $CDCl_3$): δ 175.8, 168.3, 156.9, 146.9, 137.6, 137.4, 134.0, 133.8, 133.5, 132.3, 129.6 (2C), 128.6, 128.3, 125.0, 115.1 (2C), 112.6, 66.9, 33.5, 29.5. HRMS (ESI-TOF) *m*/*z*: $[M + H]^+$ Calcd for $C_{21}H_{19}N_2O_5S$ 411.1015; Found 411.1002.

3-[4-(4-Pentenyloxy)phenyl]propionic acid, 2-thioxopyridinyl ester (1i).

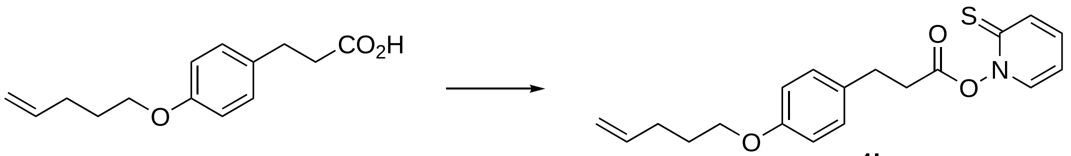

S26 **1i**

As described for the preparation of **1b**, compound **S26** (97.9 mg, 0.418 mmol) was converted to 124 mg (87%) of **1i**. Compound **1i** was obtained as yellow crystals. mp 68-71 °C. TLC R_f 0.50 (EtOAc/hexane, 1:1). IR (KBr): 2940, 1809 cm^{-1}. ^{1}H NMR (400 MHz, $CDCl_3$): δ 7.69 (dd, 1H, *J* =8.8, 1.8 Hz, H-6 of 2-thioxopyridinyl), 7.14 (dd, 1H, *J* =7.0, 1.8 Hz, H-3 of 2-thioxopyridinyl), 7.22-7.15 (m, 3H, H-5 of 2-thioxopyridinyl, H-2, 6 of Ar), 6.85 (d, 2H, *J* =8.8 Hz, H-3, 5 of Ar), 6.60 (td, 1H, *J* =7.0, 1.8 Hz, H-4 of 2-thioxopyridinyl), 5.85 (m, 1H, H-4 of 4-pentenyloxy), 5.08-4.97 (m, 2H, H-5, 5' of 4-pentenyloxy), 3.96 (t, 2H, *J* =6.9 Hz, H-1, 1' of 4-pentenyloxy), 3.11-2.97 (m, 4H, H-2, 2', 3, 3'), 2.23 (q, 2H, *J* =6.9 Hz, H-3, 3' of 4-pentenyloxy), 1.87 (quin, 2H, *J* =6.9 Hz, H-2, 2' of 4-pentenyloxy). ^{13}C{^{1}H} NMR (100 MHz, $CDCl_3$): δ 175.8, 168.3, 157.8, 137.8, 137.6, 137.4, 133.5, 131.2, 129.4 (2C), 115.2, 114.6 (2C), 112.6, 67.2, 33.6, 30.1, 29.5, 28.4. HRMS (ESI-TOF) *m*/*z*: [M + H]$^+$ Calcd for $C_{19}H_{22}NO_3S$ 344.1320; Found 344.1327.

3-[4-(3-Phenylpropioloxy)phenyl]propionic acid, 2-thioxopyridinyl ester (1j).

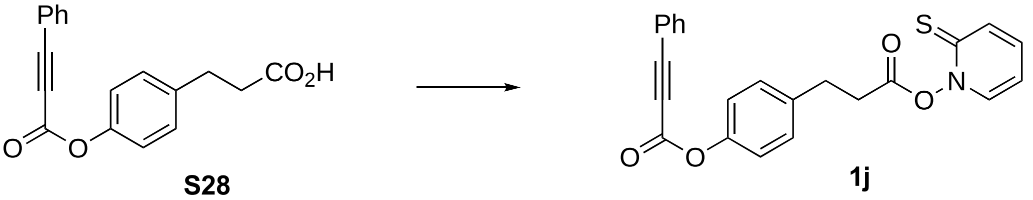

S28 **1j**

As described for the preparation of **1b**, compound **S28** (78.9 mg, 0.268 mmol) was converted to 74.5 mg (69%) of **1j**. Compound **1j** was obtained as yellow crystals. mp 85-88 °C. TLC R_f 0.55 (EtOAc/hexane, 2:1). IR (KBr): 3098, 2205, 1808, 1722 cm^{-1}. ^{1}H NMR (400 MHz, $CDCl_3$): δ 7.69 (dd, 1H, *J* =8.8, 1.9 Hz, H-6 of 2-thioxopyridinyl), 7.64 (d, 2H, *J* =7.2 Hz, H-2, 6 of Ph), 7.50 (t, 1H, *J* =7.2 Hz, H-4 of Ph), 7.41 (t, 2H, *J* =7.2 Hz, H-3, 5 of Ph), 7.37 (dd, 1H, *J* =6.9, 1.6 Hz, H-3 of 2-thioxopyridinyl), 7.32 (d, 2H, *J* =8.2 Hz, H-2, 6 of Ar), 7.20 (ddd, 1H, *J* =8.8, 6.9, 1.6 Hz, H-5 of 2-thioxopyridinyl), 7.15 (d, 2H, *J* =8.2 Hz, H-3, 5 of Ar), 6.62 (td, 1H, *J* =6.9, 1.9 Hz, H-4 of 2-thioxopyridinyl), 3.16 (t, 2H, *J* =7.1 Hz, H-3, 3'), 3.06 (t, 2H, *J* =7.1 Hz, H-2, 2'). ^{13}C{^{1}H} NMR (125 MHz, $CDCl_3$): δ 175.7, 168.0, 152.4, 148.8, 137.6, 137.5, 137.3, 133.6, 133.2 (2C), 131.1, 129.7 (2C), 128.7 (2C), 121.7 (2C), 119.1, 112.7, 88.8, 80.1, 33.2, 29.7. HRMS (ESI-TOF) *m*/*z*: [M + H]$^+$ Calcd for $C_{23}H_{18}NO_4S$ 404.0957; Found 404.0950.

3,3-Diphenylpropionic acid, 2-thioxopyridinyl ester (1k).

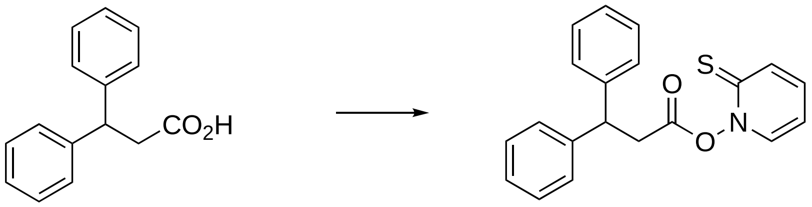

S40 **1k**

As described for the preparation of **1b**, compound **S40** (100 mg, 0.442 mmol) was converted to 88.2 mg (59%) of **1k**. Compound **1k** was obtained as yellow crystals. mp 108-111 °C. TLC R_f 0.43 (EtOAc/hexane, 1:1). IR (KBr): 3023, 1795 cm^{-1}. ^{1}H NMR (500 MHz, $CDCl_3$): δ 7.63 (dd, 1H, J =8.7, 1.8 Hz, H-6 of 2-thioxopyridinyl), 7.35-7.22 (m, 10H, -Ph), 7.14 (ddd, 1H, J =8.7, 6.9, 1.8 Hz, H-5 of 2-thioxopyridinyl), 6.94 (dd, 1H, J = 6.9, 1.8 Hz, H-3 of 2-thioxopyridinyl), 6.49 (td, 1H, J =6.9, 1.8 Hz, H-4 of 2-thioxopyridinyl), 4.67 (t, 1H, J = 8.2 Hz, H-3), 3.50 (d, 2H, J = 8.2 Hz, H-2, 2′). ^{13}C{^{1}H} NMR (125 MHz, $CDCl_3$): δ 175.8, 167.3, 142.3 (2C), 137.4, 137.3, 133.4, 128.8 (4C), 127.7 (4C), 127.0 (2C), 112.5, 46.7, 38.2. HRMS (ESI-TOF) *m*/*z*: [M + H]$^+$ Calcd for $C_{20}H_{18}NO_2S$ 336.1058; Found 336.1058.

Gemfibrozil, 2-thioxopyridinyl ester (1l).

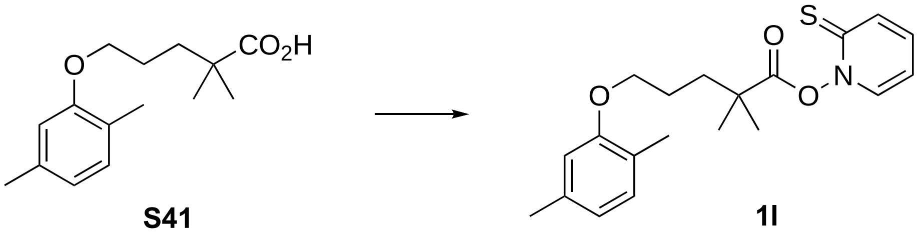

S41 **1l**

As described for the preparation of **1b**, gemfibrozil **(S41)** (102 mg, 0.406 mmol) was converted to 101 mg (69%) of **1l**. Compound **1l** was obtained as yellow crystals. mp 66-69 °C. TLC R_f 0.67 (EtOAc/hexane, 1:1). IR (KBr): 2924, 1793 cm^{-1}. ^{1}H NMR (500 MHz, $CDCl_3$): δ 7.68 (dd, 1H, J =8.8, 1.6 Hz, H-6 of 2-thioxopyridinyl), 7.34 (dd, 1H, J =6.9, 1.5 Hz, H-3 of 2-thioxopyridinyl), 7.17 (ddd, 1H, J = 8.8, 6.9, 1.5 Hz, H-5 of 2-thioxopyridinyl), 7.00 (d, 1H, J = 7.5 Hz, H-3 of dimethylphenoxy), 6.67 (d, 1H, J = 7.5 Hz, H-4 of dimethylphenoxy), 6.63 (s, 1H, H-6 of dimethylphenoxy), 6.57 (td, 1H, J = 6.9, 1.6 Hz, H-4 of 2-thioxopyridinyl), 4.00 (t, 2H, J = 5.8 Hz, H-5, 5’), 2.31 (s, 3H, $-CH_3$ of dimethylphenoxy), 2.18 (s, 3H, $-CH_3$ of dimethylphenoxy), 2.01-1.89 (m, 4H, H-3, 3’, 4, 4’), 1.49 (s, 6H, $-CH_3\times2$). ^{13}C{^{1}H} NMR (125 MHz, $CDCl_3$): δ 176.1, 173.0, 156.7, 137.6 (2C), 136.5, 133.3, 130.4, 123.6, 120.8, 112.5, 112.0, 67.5, 42.3, 37.0, 25.1 (2C), 25.0, 21.4, 15.8. HRMS (ESI-TOF) *m*/*z*: [M + H]$^+$ Calcd for $C_{20}H_{26}NO_3S$ 360.1633; Found 360.1631.

Ketoprofen, 2-thioxopyridinyl ester (1m).

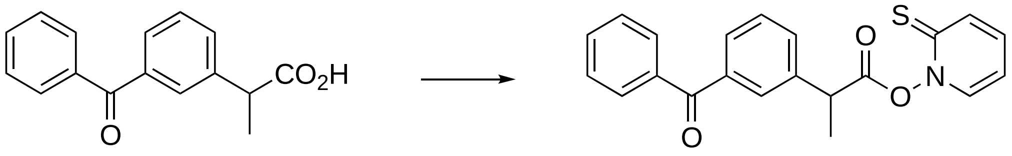

S42 **1m**

As described for the preparation of **1b**, ketoprofen **(S42)** (101 mg, 0.397 mmol) was converted to 56.8 mg (39%) of **1m**. Compound **1m** was obtained as a yellow oil. TLC R_f 0.44 (EtOAc/hexane, 1:1). IR (neat): 3028, 1800, 1734 cm^{-1}. ^{1}H NMR (500 MHz, $CDCl_3$): δ 7.85 (s, 1H, H-2 of 3-benzoylphenyl), 7.81 (d, 2H, *J* =7.0 Hz, H-2, 6 of Bz), 7.74 (d, 1H, *J* =7.5 Hz, H-4 of 3-benzoylphenyl), 7.70-7.66 (m, 2H, H-6 of 2-thioxopyridinyl, H-6 of 3-benzoylphenyl), 7.60 (t, 1H, *J* =7.3 Hz, H-4 of Bz), 7.53-7.47 (m, 3H, H-3, 5 of Bz, H-5 of 3-benzoylphenyl), 7.45 (dd, 1H, *J* =7.1, 1.6 Hz, H-3 of 2-thioxopyridinyl), 7.19 (ddd, 1H, *J* = 8.9, 7.1, 1.6 Hz, H-5 of 2-thioxopyridinyl), 6.61 (td, 1H, *J* = 7.1, 1.7 Hz, H-4 of 2-thioxopyridinyl), 4.25 (q, 1H, *J* = 7.4 Hz, H-2), 1.77 (d, 3H, *J* = 7.4 Hz, $-CH_3$). $^{13}C\{^1H\}$ NMR (125 MHz, $CDCl_3$): δ 196.2, 175.5, 169.8, 138.5, 138.2, 137.4, 137.3, 137.2, 133.4, 132.7, 131.9, 130.1 (2C), 129.7, 129.2, 128.9, 128.4 (2C), 112.6, 43.5, 18.7. HRMS (ESI-TOF) *m*/*z*: $[M + H]^+$ Calcd for $C_{21}H_{18}NO_3S$ 364.1007; Found 364.1019.

3-(3,4,5,6-Tetrachlorophthalimidyloxycarbonyl)adamantanecarboxylic acid, 2-thioxopyridinyl ester (1n).

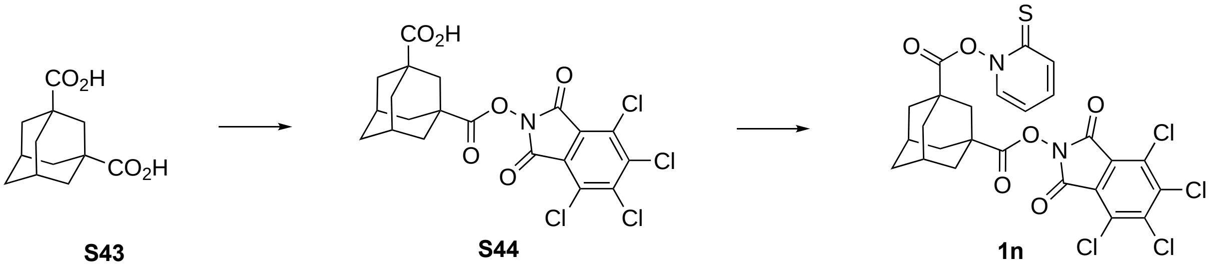

S43 **S44** **1n**

The following reaction was carried out under Ar and in the flask protected from light with aluminum foil. To a stirred solution of *N*-hydroxytetrachlorophthalimide (205 mg, 0.680 mmol) and EDCI·HCl (232 mg, 1.21 mmol) in CH_2Cl_2 (0.4 mL) was added a solution of **S43** (205 mg, 0.666 mmol) in DMF (3 mL). The mixture was stirred at room temperature for 1 h. The precipitated solids were removed by filtration through a pad of Celite and washed well with EtOAc. The combined filtrate and washings were concentrated under reduced pressure. The residue was diluted with EtOAc (20 mL) and washed with H_2O (20 mL×3) and saturated brine (10 mL), sequentially. The organic layer was dried and concentrated under reduced pressure to provide crude tetrachlorophthalimidyl ester **S44**, which was used in the next step without further purification.

To a stirred solution of crude tetrachlorophthalimidyl ester **S44** obtained above in CH_2Cl_2 (5 mL) was added 2-mercaptopyridine *N*-oxide (73.4 mg, 0.577 mmol) and EDCI·HCl (162 mg, 0.845 mmol). After being stirred at room temperature for 1 h, the mixture was diluted with CH_2Cl_2 (20 mL) and washed with 5wt% aqueous $NaHCO_3$ (10 mL×3), H_2O (10 mL×3) and saturated brine (10 mL), sequentially. The organic layer was dried and concentrated under

reduced pressure. The residue was purified by column chromatography on silica gel (CH_2Cl_2) to provide 72.2 mg (18%) of **1n** as yellow crystals. mp 115-120 °C. TLC R_f 0.50 (EtOAc/hexane, 1:1). IR (KBr): 2860, 1787, 1747 cm^{-1}. ^{1}H NMR (500 MHz, $CDCl_3$): δ 7.68 (dd, 1H, J =8.8, 1.6 Hz, H-6 of 2-thioxopyridinyl), 7.56 (dd, 1H, J =6.8, 1.5 Hz, H-3 of 2-thioxopyridinyl), 7.19 (ddd, 1H, J = 8.8, 6.8, 1.5 Hz, H-5 of 2-thioxopyridinyl), 6.61 (td, 1H, J = 6.8, 1.6 Hz, H-4 of 2-thioxopyridinyl), 2.51 (s, 2H, H-2, 2' of adamantane), 2.34 (s, 2H), 2.30-2.11 (m, 6H), 1.83 (s, 2H). ^{13}C{^{1}H} NMR (125 MHz, $CDCl_3$): δ 175.8, 171.7, 171.4, 157.6 (2C), 141.0 (2C), 137.6, 137.5, 133.4 (2C), 130.4, 124.7 (2C), 112.6, 41.0, 40.7, 38.6, 37.5 (2C), 37.4 (2C), 34.7, 27.3 (2C). HRMS (ESI-TOF) m/z: $[M + H]^+$ Calcd for $C_{25}H_{18}Cl_4N_2O_6S$ 614.9718; Found 614.9731.

3-[4-(5-(Phthalimidyloxycarbonyl)pentyloxy)phenyl]propionic acid, 2-thioxopyridinyl ester (1o).

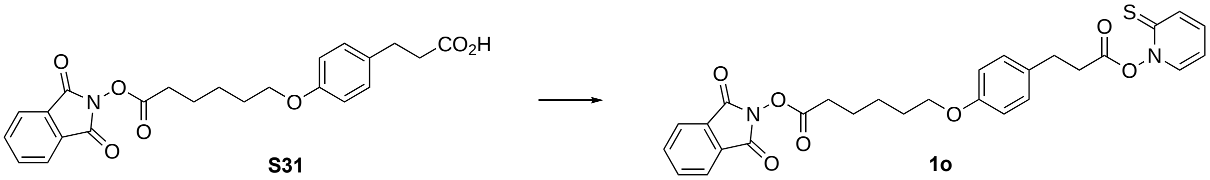

As described for the preparation of **1a**, compound **S31** (256 mg, 0. 603 mmol) was converted to 204 mg (63%) of **1o**. Compound **1o** was obtained as yellow crystals. mp 108-110 °C. TLC R_f 0.49 (EtOAc/hexane, 2:1). IR (KBr): 2936, 1811, 1788, 1738 cm^{-1}. ^{1}H NMR (500 MHz, $CDCl_3$): δ 7.91-7.87 (m, 2H, H-3, 6 of phthalimidyl), 7.82-7.77 (m, 2H, H-4, 5 of phthalimidyl), 7.68 (dd, 1H, J =8.8, 1.8 Hz, H-6 of 2-thioxopyridinyl), 7.41 (dd, 1H, J =7.0, 1.0 Hz, H-3 of 2-thioxopyridinyl), 7.21-7.15 (m, 3H, H-2, 6 of Ar, H-5 of 2-thioxopyridinyl), 6.86 (d, 2H, J =8.5 Hz, H-3, 5 of Ar), 6.60 (td, 1H, J = 7.0, 1.8 Hz, H-4 of 2-thioxopyridinyl), 3.98 (t, 2H, J =6.3 Hz, H-1, 1' of pentyloxy), 3.08 (t, 2H, J =7.4 Hz, H-3, 3'), 3.01 (t, 2H, J =7.4 Hz, H-2, 2'), 2.71 (t, 2H, J =7.4 Hz, H-5, 5' of pentyloxy), 1.91-1.81 (m, 4H, H-2, 2', 4, 4' of pentyloxy), 1.68-1.60 (m, 2H, H-3, 3' of pentyloxy). ^{13}C{^{1}H} NMR (125 MHz, $CDCl_3$): δ 175.9, 169.5, 168.3, 162.0 (2C), 157.8, 137.6, 137.4, 134.8 (2C), 133.5, 131.2, 129.4 (2C), 128.9 (2C), 124.0 (2C), 114.7 (2C), 112.5, 67.5, 33.6, 30.9, 29.5, 28.8, 25.4, 24.4. HRMS (ESI-TOF) m/z: $[M + H]^+$ Calcd for $C_{28}H_{27}N_2O_7S$ 535.1539; Found 535.1538.

3-[4-(5-(3,4,5,6-Tetrachlorophthalimidyloxycarbonyl)pentyloxy)phenyl]propionic acid, 2-thioxopyridinyl ester (1p).

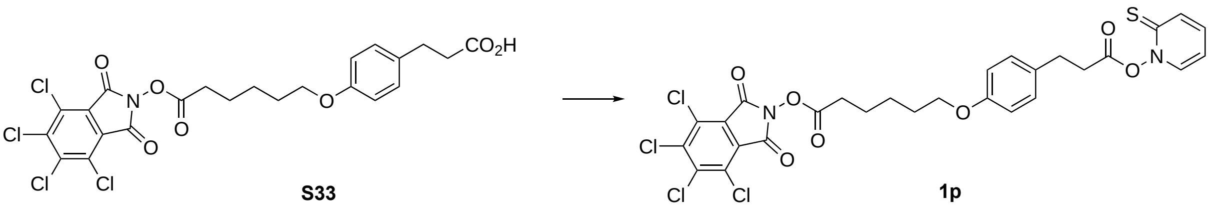

As described for the preparation of **1a**, compound **S33** (150 mg, 0.267 mmol) was converted to 62.3 mg (35%) of **1p**. Compound **1p** was obtained as a yellow oil. TLC R_f 0.72

(EtOAc/hexane, 2:1). IR (neat): 2934, 1815, 1793, 1748 cm^{-1}. ^1H NMR (400 MHz, $CDCl_3$): δ 7.67 (d, 1H, *J* =8.8 Hz, H-6 of 2-thioxopyridinyl), 7.41 (d, 1H, *J* =6.8 Hz, H-3 of 2-thioxopyridinyl), 7.22-7.13 (m, 3H, H-2, 6 of Ar, H-5 of 2-thioxopyridinyl), 6.84 (d, 2H, *J* =7.6 Hz, H-3, 5 of Ar), 6.60 (t, 1H, *J* = 6.8 Hz, H-4 of 2-thioxopyridinyl), 3.97 (t, 2H, *J* =6.0 Hz, H-1, 1' of pentyloxy), 3.07 (t, 2H, *J* =7.1 Hz, H-3, 3'), 3.00 (t, 2H, *J* =7.1 Hz, H-2, 2'), 2.71 (t, 2H, *J* =7.2 Hz, H-5, 5' of pentyloxy), 1.92-1.80 (m, 4H, H-2, 2', 4, 4' of pentyloxy), 1.68-1.58 (m, 2H, H-3, 3' of pentyloxy). $^{13}C\{^1H\}$ NMR (100 MHz, $CDCl_3$): δ 175.8, 169.0, 168.3, 157.7, 157.5 (2C), 141.0 (2C), 137.6, 137.4, 133.5 (2C), 131.2, 130.4, 129.4 (2C), 124.6 (2C), 114.6 (2C), 112.6, 67.4, 33.6, 30.8, 29.5, 28.7, 25.3, 24.4. HRMS (ESI-TOF) *m*/*z*: $[M + H]^+$ Calcd for $C_{28}H_{23}Cl_4N_2O_7S$ 670.9980; Found 670.9955.

Abietic acid, 2-thioxopyridinyl ester (1q).

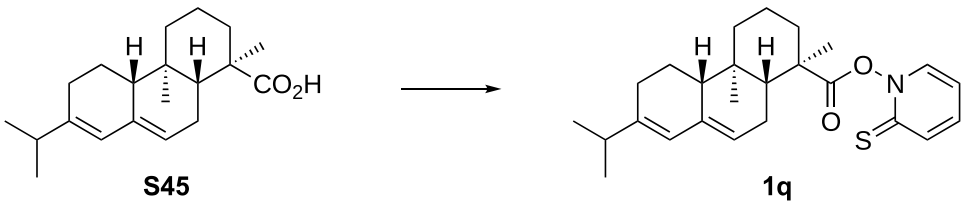

S45 1q

As described for the preparation of **1b**, abietic acid **(S45)** (99.4 mg, 0.329 mmol) was converted to 71.1 mg (53%) of **1q**. Compound **1q** was obtained as yellow crystals. TLC R_f 0.67 (EtOAc/hexane, 1:1). $[\alpha]^{25}_D$ -193 (*c* 0.615, $CHCl_3$). IR (neat): 2930, 1786 cm^{-1}. ^1H NMR (500 MHz, $CDCl_3$): δ 7.67 (dd, 1H, *J* =8.9, 1.8 Hz, H-6 of 2-thioxopyridinyl), 7.41 (dd, 1H, *J* =6.9, 1.5 Hz, H-3 of 2-thioxopyridinyl), 7.17 (ddd, 1H, *J* = 8.9, 6.9, 1.5 Hz, H-5 of 2-thioxopyridinyl), 6.57 (td, 1H, *J* = 6.9, 1.8 Hz, H-4 of 2-thioxopyridinyl), 5.78 (s, 1H, H-8), 5.40 (m, 1H, H-9), 2.27-2.21 (m, 2H), 2.18 (m, 1H), 2.11-2.02 (m, 5H), 1.98 (m, 1H), 1.92 (m, 1H), 1.83 (m, 1H), 1.69-1.62 (m, 2H), 1.50 (s, 3H, $-CH_3$), 1.32-1.16 (m, 3H), 1.20 (d, 3H, *J* = 4.5 Hz, *i*-Pr), 1.00 (d, 3H, *J* = 4.5 Hz, *i*-Pr), 0.88 (s, 3H, $-CH_3$). $^{13}C\{^1H\}$ NMR (125 MHz, $CDCl_3$): δ 176.1, 173.8, 145.6, 137.6 (2C), 135.6, 133.3, 122.2, 119.9, 112.6, 50.8, 46.9, 45.1, 37.9, 37.4, 34.8, 34.7, 27.3, 25.9, 22.4, 21.4, 20.8, 17.8, 17.0, 14.2. HRMS (ESI-TOF) *m*/*z*: $[M + H]^+$ Calcd for $C_{25}H_{34}NO_2S$ 412.2310; Found 412.2295.

3-*O*-Benzoyl-lithocholic acid, 2-thioxopyridinyl ester (1r).

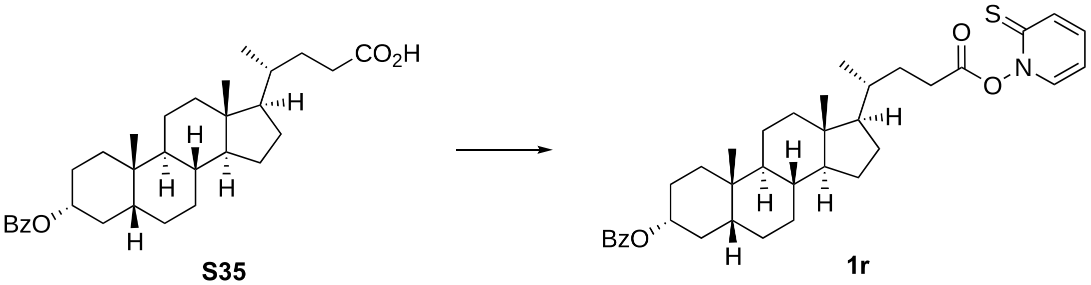

S35 1r

As described for the preparation of **1b**, **S35** (50.8 mg, 0.354 mmol) was converted to 42.6 mg (69%) of **1r**. Compound **1r** was obtained as yellow crystals. mp 70-73 °C. TLC R_f 0.60

(EtOAc/hexane, 1:1). $[\alpha]^{26}_D$ +105 (*c* 1.25, $CHCl_3$). IR (KBr): 2937, 1808, 1713 cm^{-1}. ^1H NMR (500 MHz, $CDCl_3$): δ 8.05 (d, 2H, *J* =7.8 Hz, H-2, 6 of Bz), 7.69 (dd, 1H, *J* =8.5, 1.6 Hz, H-6 of 2-thioxopyridinyl), 7.58-7.52 (m, 2H, H-4 of Bz, H-3 of 2-thioxopyridinyl), 7.43 (t, 2H, *J* =7.8 Hz, H-3, 5 of Bz), 7.20 (ddd, 1H, *J* =8.5, 6.9, 1.5 Hz, H-5 of 2-thioxopyridinyl), 6.63 (td, 1H, *J* =6.9, 1.6 Hz, H-4 of 2-thioxopyridinyl), 4.97 (m, 1H, H-3), 2.76 (m, 1H), 2.63 (m, 1H), 2.03-1.95 (m, 3H), 1.92-1.79 (m, 4H), 1.67 (m, 1H), 1.63-1.50 (m, 5H) , 1.49-1.39 (m, 4H), 1.35-1.20 (m, 4H), 1.19-1.04 (m, 5H), 0.98 (d, 3H, *J* =6.5 Hz, H-21), 0.96 (s, 3H, H-19), 0.67 (s, 3H, H-18). $^{13}C\{^1H\}$ NMR (125 MHz, $CDCl_3$): δ 175.9, 169.6, 166.1, 137.6, 137.4, 133.4, 132.6, 130.9, 129.5 (2C), 128.2 (2C), 112.5, 75.0, 56.4, 55.8, 42.8, 41.9, 40.4, 40.1, 35.8, 35.2, 35.0, 34.6, 32.3, 30.3, 28.6, 28.2, 27.0, 26.7, 26.3, 24.1, 23.3, 20.8, 18.3, 12.1. HRMS (ESI-TOF) *m*/*z*: $[M + H]^+$ Calcd for $C_{36}H_{48}NO_4S$ 590.3304; Found 590.3297.

Linoleic acid, 2-thioxopyridinyl ester (1s).

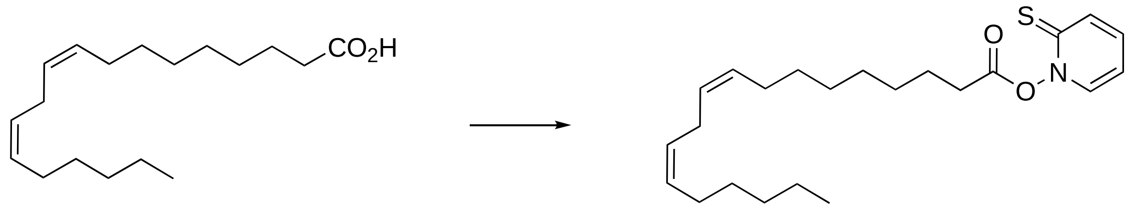

S46 **1s**

As described for the preparation of **1b**, linoleic acid **(S46)** (0.110 mL, 0.354 mmol) was converted to 121 mg (88%) of **1s**. Compound **1s** was obtained as a yellow oil. TLC R_f 0.72 (EtOAc/hexane, 1:1). IR (neat): 2927, 1808 cm^{-1}. ^1H NMR (500 MHz, $CDCl_3$): δ 7.68 (dd, 1H, *J* =9.1, 1.6 Hz, H-6 of 2-thioxopyridinyl), 7.55 (dd, 1H, *J* =7.0, 1.5 Hz, H-3 of 2-thioxopyridinyl), 7.17 (ddd, 1H, *J* = 9.1, 7.0, 1.5 Hz, H-5 of 2-thioxopyridinyl), 6.57 (td, 1H, *J* = 7.0, 1.6 Hz, H-4 of 2-thioxopyridinyl), 5.41-5.29 (m, 4H, H-9, 10, 12, 13), 2.77 (t, 2H, *J* = 6.8 Hz, H-11, 11’), 2.71 (t, 2H, *J* = 7.4 Hz, H-2, 2’), 2.08-1.98 (m, 4H, H-8, 8’, 14, 14’), 1.81 (quin, 2H, *J* = 7.4 Hz, H-3, 3’), 1.46-1.40 (m, 2H), 1.39-1.24 (m, 12H), 0.88 (t, 3H, *J* = 7.0 Hz, $-CH_3$). $^{13}C\{^1H\}$ NMR (125 MHz, $CDCl_3$): δ 175.9, 169.0, 137.6, 137.4, 133.5, 130.2, 130.0, 128.1, 127.9, 112.5, 31.6, 31.5, 29.5, 29.3, 29.0 (2C), 28.9, 27.2, 27.1, 25.6, 24.3, 22.5, 14.1. HRMS (ESI-TOF) *m*/*z*: $[M + H]^+$ Calcd for $C_{23}H_{36}NO_2S$ 390.2467; Found 390.2471.

***O*-Acetyl-mycophenolic acid, 2-thioxopyridinyl ester (1t).**

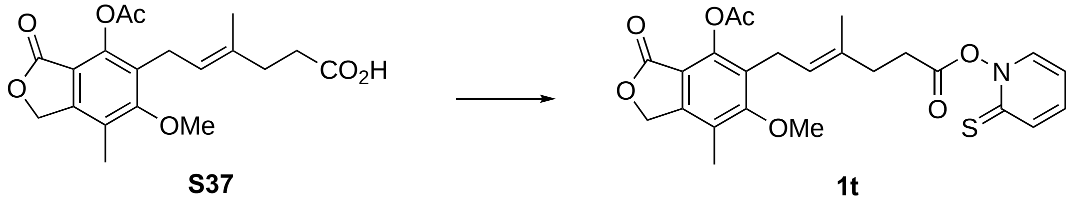

S37 **1t**

As described for the preparation of **1b**, compound **S37** (70.2 mg, 0.194 mmol) was converted to 65.2 mg (71%) of **1t**. Compound **1t** was obtained as yellow crystals. mp 48-50 °C. TLC R_f 0.43 (EtOAc/hexane, 1:1). IR (KBr): 2937, 1809, 1760 cm^{-1}. ^1H NMR (500

MHz, $CDCl_3$): δ 7.66 (dd, 1H, *J* =8.7, 1.8 Hz, H-6 of 2-thioxopyridinyl), 7.56 (dd, 1H, *J* =6.9, 1.5 Hz, H-3 of 2-thioxopyridinyl), 7.19 (ddd, 1H, *J* = 8.7, 6.9, 1.5 Hz, H-5 of 2-thioxopyridinyl), 6.61 (td, 1H, *J* = 6.9, 1.8 Hz, H-4 of 2-thioxopyridinyl), 5.20-5.13 (m, 3H, C=C*H*, CO_2CH_2), 3.80 (s, 3H, OMe), 3.38 (d, 2H, *J* =7.0 Hz, C=CH-CH_2), 2.81 (t, 2H, *J* = 7.5 Hz, -C=O-CH_2), 2.48 (t, 2H, *J* =7.5 Hz, -C=O-CH_2-CH_2), 2.39 (s, 3H, OAc), 2.23 (s, 3H, Ar-CH_3), 1.84 (s, 3H, CH_3-C=CH). $^{13}C\{^1H\}$ NMR (125 MHz, $CDCl_3$): δ 175.8, 169.0, 168.4, 168.3, 162.6, 146.3, 145.9, 137.8, 137.3, 133.6 (2C), 129.1, 123.1, 122.9, 113.5, 112.6, 68.4, 61.3, 33.3, 30.1, 23.5, 20.6, 16.4, 11.8. HRMS (ESI-TOF) *m*/*z*: $[M + H]^+$ Calcd for $C_{24}H_{26}NO_7S$ 472.1430; Found 472.1417.

2,7-Di-*O*-acetyl-gibberellic acid, 2-thioxopyridinyl ester (1u).

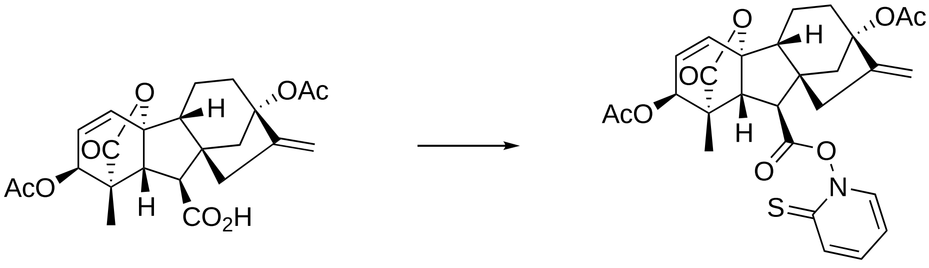

S39 **1u**

As described for the preparation of **1b**, compound **S39** (80.3 mg, 0.187 mmol) was converted to 53.9 mg (54%) of **1u**. Compound **1u** was obtained as yellow solids. TLC R_f 0.43 (EtOAc/hexane, 1:1). $[\alpha]^{24}_D$ +284 (*c* 1.18, $CHCl_3$). IR (KBr): 2939, 1780, 1738 cm^{-1}. ^1H NMR (400 MHz, $CDCl_3$): δ 7.68 (dd, 1H, *J* =8.8, 1.8 Hz, H-6 of 2-thioxopyridinyl), 7.56 (dd, 1H, *J* =6.8, 1.4 Hz, H-3 of 2-thioxopyridinyl), 7.21 (ddd, 1H, *J* = 8.8, 6.8, 1.4 Hz, H-5 of 2-thioxopyridinyl), 6.66 (td, 1H, *J* = 6.8, 1.8 Hz, H-4 of 2-thioxopyridinyl), 6.38 (d, 1H, *J* =9.1 Hz, H-4), 5.87 (dd, 1H, *J* =9.1, 3.9 Hz, H-3), 5.36 (d, 1H, *J* =3.9 Hz, H-2), 5.18 (d, 1H, *J* =2.2 Hz, H-12), 5.02 (d, 1H, *J* =2.2 Hz, H-12'), 3.44 (d, 1H, *J* =10.4 Hz, H-10a), 3.21 (d, 1H, *J* =10.4 Hz, H-10), 2.76-2.64 (m, 2H), 2.40-2.23 (m, 3H), 2.10 (s, 3H, OAc), 2.07-2.00 (m, 2H) 2.03 (s, 3H, OAc), 1.90-1.73 (m, 2H), 1.41 (s, 3H, H-14). $^{13}C\{^1H\}$ NMR (125 MHz, $CDCl_3$): δ 176.5 (2C), 170.0 (3C), 152.3, 137.6, 137.2, 133.8, 133.4, 129.5, 113.0, 108.6, 89.6, 83.8, 70.3, 54.1, 52.2, 51.8, 50.8, 47.8, 43.1, 39.4, 36.6, 22.0, 20.8, 16.8, 15.3. HRMS (ESI-TOF) *m*/*z*: $[M + H]^+$ Calcd for $C_{28}H_{30}NO_8S$ 540.1692; Found 540.1696.

Zaltoprofen, 2-thioxopyridinyl ester (1z).

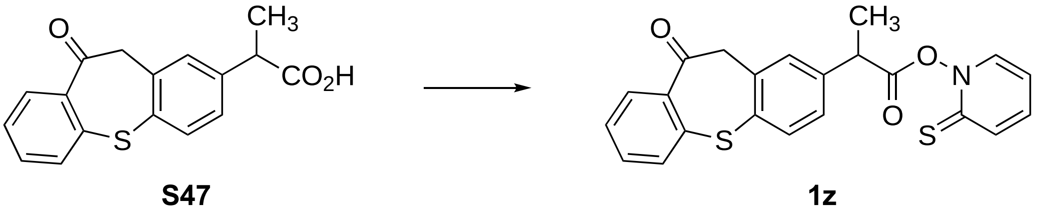

S47 **1z**

As described for the preparation of **1b**, zaltoprofen **(S47)** (100 mg, 0.336 mmol) was converted to 69.0 mg (50%) of **1z**. Compound **1z** was obtained as yellow crystals. mp 85-

89 °C. TLC R_f 0.41 (EtOAc/hexane, 1:1). IR (KBr): 2977, 1799, 1729, 1671 cm^{-1}. ^{1}H NMR (400 MHz, $CDCl_3$): δ 8.20 (dd, 1H, *J* =7.8, 1.6 Hz, H-9 of dihydrodibenzo[b,f]thiepin), 7.68 (dd, 1H, *J* =8.7, 1.9 Hz, H-6 of 2-thioxopyridinyl), 7.66 (d, 1H, *J* =8.1 Hz, H-4 of dihydrodibenzo[b,f]thiepin), 7.61 (dd, 1H, *J* =7.8, 1.4 Hz, H-6 of dihydrodibenzo[b,f]thiepin), 7.49 (d, 1H, *J* =1.8 Hz, H-1 of dihydrodibenzo[b,f]thiepin), 7.45 (td, 1H, *J* =7.8, 1.6 Hz, H-7 of dihydrodibenzo[b,f]thiepin), 7.40 (dd, 1H, *J* =6.9, 1.4 Hz, H-3 of 2-thioxopyridinyl), 7.33 (td, 1H, *J* =7.8, 1.4 Hz, H-8 of dihydrodibenzo[b,f]thiepin), 7.29 (dd, 1H, *J* =8.1, 1.8 Hz, H-3 of dihydrodibenzo[b,f]thiepin), 7.18 (ddd, 1H, *J* = 8.7, 6.9, 1.4 Hz, H-5 of 2-thioxopyridinyl), 6.58 (td, 1H, *J* = 6.9, 1.9 Hz, H-4 of 2-thioxopyridinyl), 4.39 (s, 2H, -CH_2-), 4.17 (q, 1H, *J* = 7.2 Hz, H-2), 1.72 (d, 3H, *J* = 7.2 Hz, -CH_3). $^{13}C\{^1H\}$ NMR (125 MHz, $CDCl_3$): δ 191.2, 175.5, 169.7, 140.2, 139.9, 138.4, 137.3 (2C), 136.1, 134.2, 133.4, 132.6, 131.8, 131.5, 130.9, 128.7, 126.9, 126.8, 112.6, 51.0, 43.3, 18.7. HRMS (ESI-TOF) *m*/*z*: $[M + H]^+$ Calcd for $C_{22}H_{18}NO_3S_2$ 408.0728; Found 408.0726.

Xanthene-9-carboxylic acid, 2-thioxopyridinyl ester (1aa).

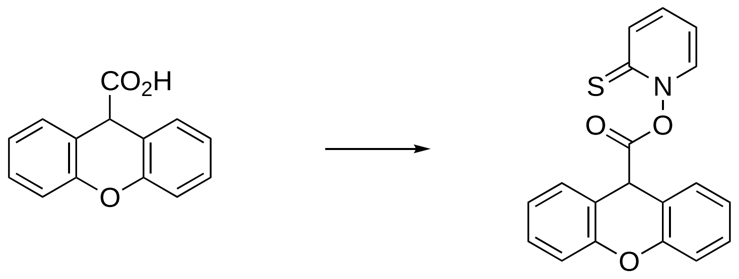

S48 **1aa**

As described for the preparation of **1a**, compound **S48** (101 mg, 0.444 mmol) was converted to 29.2 mg (20%) of **1aa**. Compound **1aa** was obtained as yellow crystals. mp 112-116 °C. TLC R_f 0.44 (EtOAc/hexane, 1:1). IR (KBr): 3017, 1799, 1713 cm^{-1}. ^{1}H NMR (500 MHz, $CDCl_3$): δ 7.66 (dd, 1H, *J* =9.0, 1.7 Hz, H-6 of 2-thioxopyridinyl), 7.59 (dd, 2H, *J* =7.9, 1.3 Hz, H-1, 8 of xanthene), 7.41 (dd, 1H, *J* =7.0, 1.0 Hz, H-3 of 2-thioxopyridinyl), 7.36 (td, 2H, *J* =7.9, 1.3 Hz, H-3, 6 of xanthene), 7.20-7.13 (m, 5H, H-2, 4, 5, 7 of xanthene, H-5 of 2-thioxopyridinyl), 6.56 (td, 1H, J = 7.0, 1.7 Hz, H-4 of 2-thioxopyridinyl), 5.51 (s, 1H, H-9). $^{13}C\{^1H\}$ NMR (125 MHz, $CDCl_3$): δ 174.3, 168.2, 151.5 (2C), 137.4, 137.0, 133.1, 129.9 (4C), 123.7 (2C), 117.2 (2C), 116.0 (2C), 113.0, 43.6. HRMS (ESI-TOF) *m*/*z*: $[M + H]^+$ Calcd for $C_{19}H_{14}NO_3S$ 336.0694; Found 336.0707.

4-Phenoxybenzoic acid, 2-thioxopyridinyl ester (1ac).

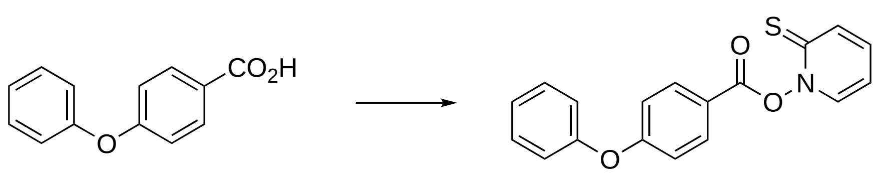

S49 **1ac**

As described for the preparation of **1b**, compound **S49** (103 mg, 0.480 mmol) was converted

to 10.0 mg (6%) of **1ac**. Compound **1ac** was obtained as yellow solids. TLC R_f 0.33 (EtOAc/hexane, 1:2). IR (neat): 3066, 1772, 1716 cm^{-1}. ^1H NMR (400 MHz, $CDCl_3$): δ 8.21 (d, 2H, J =8.8 Hz, H-2, 6 of 4-phenoxyphenyl), 7.73 (dd, 1H, J =8.8, 1.9 Hz, H-6 of 2-thioxopyridinyl), 7.68 (dd, 1H, J =7.1, 1.6 Hz, H-3 of 2-thioxopyridinyl), 7.43 (t, 2H, J =7.8 Hz, H-3, 5 of Ph), 7.26-7.20 (m, 2H, H-4 of Ph, H-5 of 2-thioxopyridinyl), 7.10 (d, 2H, J = 7.8 Hz, H-2, 6 of Ph), 7.06 (d, 2H, J = 8.8 Hz, H-3, 5 of 4-phenoxyphenyl), 6.61 (td, 1H, J = 7.1, 1.9 Hz, H-4 of 2-thioxopyridinyl). $^{13}C\{^1H\}$ NMR (125 MHz, $CDCl_3$): δ 176.1, 163.7, 154.9, 138.1, 137.4, 133.5, 133.1 (2C), 132.8, 130.2 (2C), 125.1, 120.5 (2C), 119.3, 117.4 (2C), 112.6. HRMS (ESI-TOF) m/z: $[M + H]^+$ Calcd for $C_{18}H_{14}NO_3S$ 324.0694; Found 324.0679.

Benzyloxycarbonylaminocyclohexane (2a).

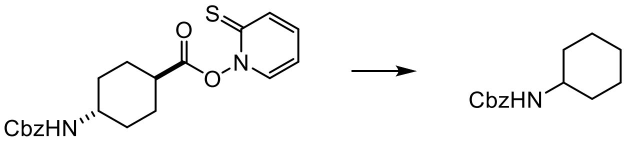

1a **2a**

The following reaction was carried out under Ar. To a stirred solution of **1a** (250 mg, 0.648 mmol) and zinc(II) tetraphenylporphyrin (0.4 mg, 0.6 µmol) in MeCN (3 mL) was added *tert*-dodecanethiol (610 µL, 2.59 mmol). The stirred mixture was irradiated by red LEDs at 25 °C for 15 min, and concentrated under reduced pressure. The residue was purified by column chromatography on silica gel (EtOAc/hexane, 1:10) to provide 124 mg (82%) of **2a** as white crystals. mp 88-90 °C. TLC R_f 0.83 (EtOAc/hexane, 1:4). IR (KBr): 3320, 2932, 1687 cm^{-1}. ^1H NMR (500 MHz, $CDCl_3$): δ 7.38-7.29 (m, 5H, -Ph of Cbz), 5.08 (s, 2H, $-CH_2-$ of Cbz), 4.63 (br s, 1H, -NH-), 3.51 (br s, 1H, H-1), 1.98-1.89 (m, 2H, H_{eq}-2, 6), 1.73-1.66 (m, 2H, H_{eq}-3, 5), 1.59 (m, 1H, H_{eq}-4), 1.40-1.30 (m, 2H, H_{ax}-2, 6), 1.21-0.99 (m, 3H, H_{ax}-3, 4, 5). $^{13}C\{^1H\}$ NMR (125 MHz, $CDCl_3$): δ 155.5, 136.7, 128.5 (2C), 128.1 (3C), 66.5, 49.9, 33.4 (2C), 25.5, 24.8 (2C). HRMS (ESI-TOF) m/z: $[M + H]^+$ Calcd for $C_{14}H_{20}NO_2$ 234.1494; Found 234.1505.

1-(*tert*-Butyldimethylsilyloxy)-4-ethylbenzene (2b).

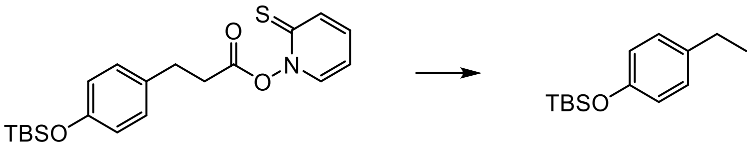

1b **2b**

As described for the preparation of **2a**, compound **1b** (10.0 mg, 25.7 µmol) was converted to 5.4 mg (89%) of **2b**. Compound **2b** was obtained as a colorless oil. TLC R_f 0.36 (hexane). IR (neat): 2961, 1609 cm^{-1}. ^1H NMR (500 MHz, $CDCl_3$): δ 7.04 (d, 2H, J =8.3 Hz, H-3, 5 of Ar), 6.75 (d, 2H, J =8.3 Hz, H-2, 6 of Ar), 2.58 (q, 2H, J =7.8 Hz, $-CH_2-$ of ethyl), 1.20 (t, 3H, J =7.8 Hz, $-CH_3$ of ethyl), 0.98 (s, 9H, t-Bu), 0.18 (s, 6H, -Me). $^{13}C\{^1H\}$ NMR (125 MHz,

CDCl3): δ 153.4, 136.9, 128.6 (2C), 119.8 (2C), 28.0, 25.7 (3C), 18.2, 15.7, -4.4 (2C). HRMS (ESI-TOF) *m/z*: $[M + H]^+$ Calcd for $C_{14}H_{25}OSi$ 237.1675; Found 237.1673.

1-Ethyl-4-(pivaloyloxy)benzene (2c).

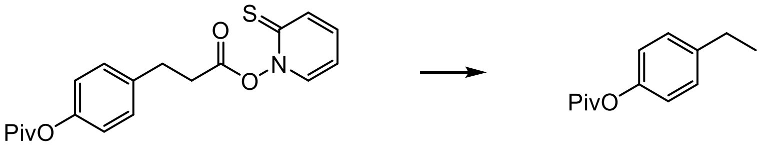

1c **2c**

As described for the preparation of **2a**, compound **1c** (10.0 mg, 27.8 μmol) was converted to 4.6 mg (82%) of **2c**. Compound **2c** was obtained as a colorless oil. TLC R_f 0.38 (EtOAc/hexane, 1:20). IR (neat): 2967, 1754 cm^{-1}. ^1H NMR (500 MHz, $CDCl_3$): δ 7.18 (d, 2H, *J* =8.5 Hz, H-2, 6 of Ar), 6.96 (d, 2H, *J* =8.5 Hz, H-3, 5 of Ar), 2.62 (q, 2H, *J* =7.6 Hz, $-CH_2-$ of ethyl), 1.35 (s, 9H, *t*-Bu), 1.20 (t, 3H, *J* =7.6 Hz, $-CH_3$ of ethyl). $^{13}C\{^1H\}$ NMR (125 MHz, $CDCl_3$): δ 177.3, 149.0, 141.5, 128.7 (2C), 121.2 (2C), 39.0, 28.3, 27.1 (3C), 15.6. HRMS (ESI-TOF) *m/z*: $[M + H]^+$ Calcd for $C_{13}H_{19}O_2$ 207.1385; Found 207.1389.

1-Ethyl-4-(2-methoxyethoxymethoxy)benzene (2d).

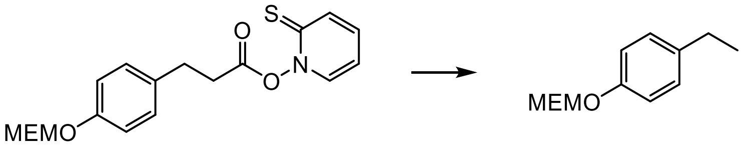

1d **2d**

As described for the preparation of **2a**, compound **1d** (10.0 mg, 27.5 μmol) was converted to 5.3 mg (92%) of **2d**. Compound **2d** was obtained as a colorless oil. TLC R_f 0.72 (EtOAc/hexane, 1:2). IR (neat): 2963 cm^{-1}. ^1H NMR (500 MHz, $CDCl_3$): δ 7.10 (d, 2H, *J* =7.3 Hz, H-2, 6 of Ar), 6.98 (d, 2H, *J* =7.3 Hz, H-3, 5 of Ar), 5.24 (s, 2H, $-OCH_2O-$), 3.85-3.78 (m, 2H, $-OCH_2CH_2O-$), 3.60-3.52 (m, 2H, $-OCH_2CH_2O-$), 3.39 (s, 3H, $-OCH_3$), 2.59 (q, 2H, *J* =7.5 Hz, $-CH_2-$ of ethyl), 1.21 (t, 3H, *J* =7.5 Hz, $-CH_3$ of ethyl). $^{13}C\{^1H\}$ NMR (125 MHz, $CDCl_3$): δ 155.3, 137.7, 128.7 (2C), 116.2 (2C), 93.7, 71.6, 67.5, 59.0, 28.0, 15.8. HRMS (ESI-TOF) *m/z*: $[M + H]^+$ Calcd for $C_{12}H_{19}O_3$ 211.1334; Found 211.1341.

1-Ethyl-4-(methanesulfonyloxy)benzene (2e).

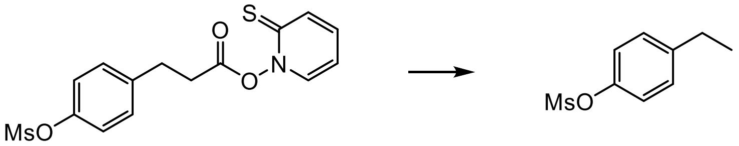

1e **2e**

As described for the preparation of **2a**, compound **1e** (10.0 mg, 28.3 μmol) was converted to 5.0 mg (88%) of **2e**. Compound **2e** was obtained as a colorless oil. TLC R_f 0.51 (EtOAc/hexane, 1:4). IR (neat): 2968 cm^{-1}. ^1H NMR (500 MHz, $CDCl_3$): δ 7.22 (d, 2H, *J* =8.5 Hz, H-2, 6 of Ar), 7.19 (d, 2H, *J* =8.5 Hz, H-3, 5 of Ar), 3.12 (s, 3H, -Me), 2.66 (q, 2H, *J* =7.6 Hz, $-CH_2-$ of ethyl), 1.24 (t, 3H, *J* =7.6 Hz, $-CH_3$ of ethyl). $^{13}C\{^1H\}$ NMR (125 MHz, $CDCl_3$):

δ 147.2, 143.6, 129.3 (2C), 121.8 (2C), 37.2, 28.3, 15.5. HRMS (ESI-TOF) *m*/*z*: $[M + K]^+$ Calcd for $C_9H_{12}KO_3S$ 239.0144; Found 239.0150.

1-(Benzyloxy)-4-ethylbenzene (2f).

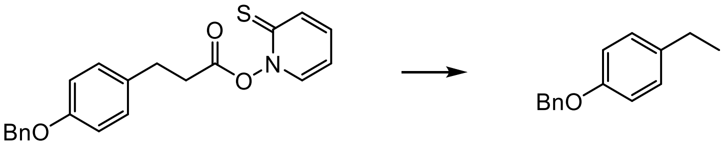

1f **2f**

As described for the preparation of **2a**, compound **1f** (11.9 mg, 32.6 µmol) was converted to 4.8 mg (70%) of **2f**. Compound **2f** was obtained as a colorless oil. TLC R_f 0.77 (EtOAc/hexane, 1:4). IR (neat): 2963, 1724 cm^{-1}. ^1H NMR (500 MHz, $CDCl_3$): δ 7.44 (d, 2H, *J* =7.4 Hz, H-2, 6 of Ph), 7.38 (t, 2H, *J* =7.4 Hz, H-3, 5 of Ph), 7.31 (t, 1H, *J* =7.4 Hz, H-4 of Ph), 7.12 (d, 2H, *J* =8.5 Hz, H-3, 5 of Ar), 6.91 (d, 2H, *J* =8.5 Hz, H-2, 6 of Ar), 5.05 (s, 2H, Bn), 2.60 (q, 2H, *J* =7.6 Hz, $-CH_2-$ of ethyl), 1.21 (t, 3H, *J* =7.6 Hz, $-CH_3$ of ethyl). $^{13}C\{^1H\}$ NMR (125 MHz, $CDCl_3$): δ 156.8, 137.3, 136.7, 128.7 (2C), 128.5 (2C), 127.9, 127.5 (2C), 114.7 (2C), 70.1, 28.0, 15.8. HRMS (ESI-TOF) *m*/*z*: $[M + H]^+$ Calcd for $C_{15}H_{17}O$ 213.1279; Found 213.1270.

1-Ethyl-4-(*p*-methoxybenzyloxy)benzene (2g).

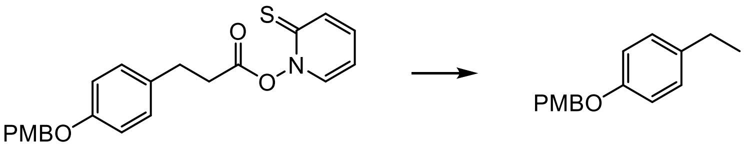

1g **2g**

As described for the preparation of **2a**, compound **1g** (13.4 mg, 33.9 µmol) was converted to 5.0 mg (61%) of **2g**. Compound **2g** was obtained as white crystals. mp 68-71 °C. TLC R_f 0.65 (toluene). IR (KBr): 2930 cm^{-1}. ^1H NMR (500 MHz, $CDCl_3$): δ 7.35 (d, 2H, *J* =9.0 Hz, H-2, 6 of PMB), 7.11 (d, 2H, *J* =8.5 Hz, H-2, 6 of Ar), 6.93-6.88 (m, 4H, H-3, 5 of Ar and H-3, 5 of PMB), 4.97 (s, 2H, Bn), 3.82 (s, 3H, -Me), 2.59 (q, 2H, *J* =7.7 Hz, $-CH_2-$ of ethyl), 1.21 (t, 3H, *J* =7.7 Hz, $-CH_3$ of ethyl). $^{13}C\{^1H\}$ NMR (125 MHz, $CDCl_3$): δ 159.4, 156.9, 136.6, 129.3, 129.2 (2C), 128.7 (2C), 114.7 (2C), 114.0 (2C), 69.8, 55.3, 28.0, 15.9. HRMS (ESI-TOF) *m*/*z*: $[M + H]^+$ Calcd for $C_{16}H_{19}O_2$ 248.1385; Found 248.1389.

1-Ethyl-4-(*o*-nitrobenzyloxy)benzene (2h).

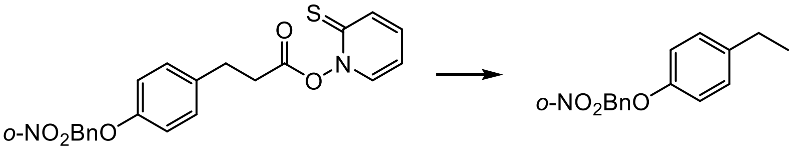

1h **2h**

As described for the preparation of **2a**, compound **1h** (9.9 mg, 24 µmol) was converted to 5.2 mg (84%) of **2h**. Compound **2h** was obtained as a beige oil. TLC R_f 0.78 (toluene). IR

(neat): 3032 cm^{-1}. ^{1}H NMR (400 MHz, $CDCl_3$): δ 8.17 (d, 1H, *J* =7.8 Hz, H-3 of *o*-NO_2Bn), 7.91 (d, 1H, *J* =7.8 Hz, H-6 of *o*-NO_2Bn), 7.68 (t, 1H, *J* =7.8 Hz, H-5 of *o*-NO_2Bn), 7.48 (t, 1H, *J* =7.8 Hz, H-4 of *o*-NO_2Bn), 7.13 (d, 2H, *J* =8.4 Hz, H-2, 6 of Ar), 6.91 (d, 2H, *J* =8.4 Hz, H-3, 5 of Ar), 5.48 (s, 2H, *o*-NO_2Bn), 2.60 (q, 2H, *J* =7.6 Hz, -CH_2- of ethyl), 1.21 (t, 3H, *J* =7.6 Hz, -CH_3 of ethyl). ^{13}C{^{1}H} NMR (125 MHz, $CDCl_3$): δ 156.1, 146.9, 137.3, 134.3, 134.0, 128.9 (2C), 128.5, 128.2, 124.9, 114.7 (2C), 66.9, 28.0, 15.8. HRMS (ESI-TOF) *m*/*z*: [M + H]$^+$ Calcd for $C_{15}H_{16}NO_3$ 258.1130; Found 258.1131.

1-Ethyl-4-(4-pentenyloxy)benzene (2i).

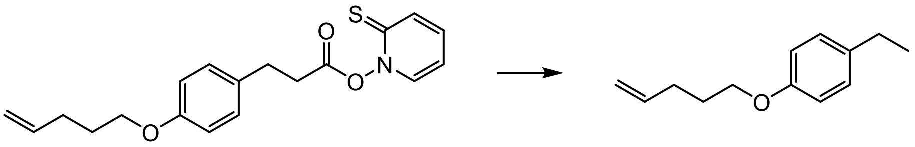

1i **2i**

As described for the preparation of **2a**, compound **1i** (29.9 mg, 87.0 µmol) was converted with 0.5 mol% of the catalyst to 14.2 mg (86%) of **2i**. Compound **2i** was obtained as a colorless oil. TLC R_f 0.48 (EtOAc/hexane, 1:4). IR (neat): 2928 cm^{-1}. ^{1}H NMR (500 MHz, $CDCl_3$): δ 7.10 (d, 2H, *J* =8.5 Hz, H-2, 6 of Ar), 6.82 (d, 2H, *J* =8.5 Hz, H-3, 5 of Ar), 5.86 (m, 1H, H-4 of 4-pentenyloxy), 5.09-4.98 (m, 2H, H-5, 5' of 4-pentenyloxy), 3.95 (t, 2H, *J* =6.9 Hz, H-1, 1' of 4-pentenyloxy), 2.59 (q, 2H, *J* =7.6 Hz, -CH_2- of ethyl), 2.24 (q, 2H, *J* =6.9 Hz, H-3, 3' of 4-pentenyloxy), 1.87 (quin, 2H, *J* =6.9 Hz, H-2, 2' of 4-pentenyloxy), 1.21 (t, 3H, *J* =7.6 Hz, -CH_3 of ethyl). ^{13}C{^{1}H} NMR (125 MHz, $CDCl_3$): δ 157.1, 137.9, 136.3, 128.6 (2C), 115.1, 114.4 (2C), 67.2, 30.1, 28.5, 28.0, 15.9. HRMS (ESI-TOF) *m*/*z*: [M + H]$^+$ Calcd for $C_{13}H_{19}O$ 191.1436; Found 191.1442.

1-Ethyl-4-(3-phenylpropioloxy)benzene (2j).

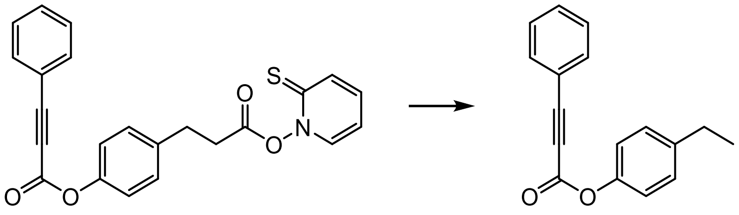

1j **2j**

As described for the preparation of **2a**, compound **1j** (28.4 mg, 70.4 µmol) was converted to 15.2 mg (86%) of **2j**. Compound **2j** was obtained as a colorless oil. TLC R_f 0.71 (toluene). IR (neat): 2921, 2234, 1808, 1722 cm^{-1}. ^{1}H NMR (500 MHz, $CDCl_3$): δ 7.63 (d, 2H, *J* =7.8 Hz, H-2, 6 of Ph), 7.49 (t, 1H, *J* =7.8 Hz, H-4 of Ph), 7.41 (t, 2H, *J* =7.8 Hz, H-3, 5 of Ph), 7.24 (d, 2H, *J* =8.8 Hz, H-2, 6 of Ar), 7.10 (d, 2H, *J* =8.8 Hz, H-3, 5 of Ar), 2.67 (q, 2H, *J* =7.6 Hz, -CH_2- of ethyl), 1.25 (t, 3H, *J* =7.6 Hz, -CH_3 of ethyl). ^{13}C{^{1}H} NMR (125 MHz, $CDCl_3$): δ 152.6, 148.0, 142.4, 133.2 (2C), 131.0, 128.9 (2C), 128.7 (2C), 121.2 (2C), 119.3, 88.5, 80.5, 28.3, 15.5. HRMS (ESI-TOF) *m*/*z*: [M + H]$^+$ Calcd for $C_{17}H_{15}O_2$ 251.1072; Found 251.1080.

1,1-Diphenylethane (2k).

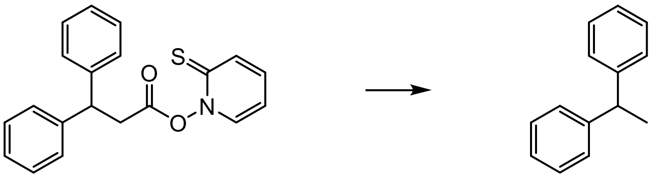

1k **2k**

As described for the preparation of **2a**, compound **1k** (9.9 mg, 30 µmol) was converted to 5.0 mg (93%) of **2k**. Compound **2k** was obtained as a colorless oil. TLC R_f 0.52 (hexane). IR (neat): 2967 cm^{-1}. ^{1}H NMR (500 MHz, $CDCl_3$): δ 7.30 (t, 4H, *J* =7.3 Hz, H-3, 5 of Ph), 7.24 (d, 4H, *J* =7.3 Hz, H-2, 6 of Ph), 7.20 (t, 2H, *J* =7.3 Hz, H-4 of Ph), 4.17 (q, 1H, *J* =7.4 Hz, H-1), 1.66 (d, 3H, *J* =7.4 Hz, -CH_3). ^{13}C{^{1}H} NMR (125 MHz, $CDCl_3$): δ 146.3 (2C), 128.3 (4C), 127.6 (4C), 126.0 (2C), 44.7, 21.8. HRMS (ESI-TOF) *m*/*z*: [M + H]$^+$ Calcd for $C_{14}H_{15}$ 183.1174; Found 183.1174.

1,4-Dimethyl-2-(4-methylpentyloxy)benzene (2l).

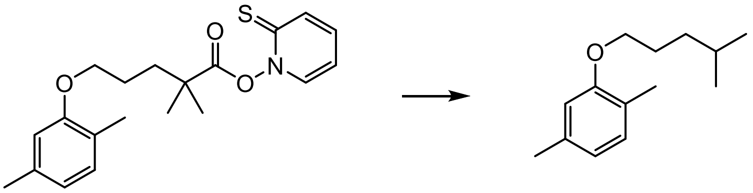

1l **2l**

As described for the preparation of **2a**, compound **1l** (10.0 mg, 27.8 µmol) was converted to 4.1 mg (71%) of **2l**. Compound **2l** was obtained as a colorless oil. TLC R_f 0.88 (EtOAc/hexane, 1:6). IR (KBr): 2955 cm^{-1}. ^{1}H NMR (500 MHz, $CDCl_3$): δ 7.01 (d, 1H, *J* = 7.5 Hz, H-6), 6.66 (d, 1H, *J* = 7.5 Hz, H-5), 6.64 (s, 1H, H-3), 3.93 (t, 2H, *J* = 6.8 Hz, H-1, 1′ of 4-methylpentyloxy), 2.32 (s, 3H, -CH_3 of dimethylphenoxy), 2.19 (s, 3H, -CH_3 of dimethylphenoxy), 1.84-1.77 (m, 2H, H-2, 2′ of 4-methylpentyloxy), 1.63 (m, 1H, H-4 of 4-methylpentyloxy), 1.40-1.34 (m, 2H, H-3, 3′ of 4-methylpentyloxy), 0.93 (d, 6H, *J* = 6.5 Hz, -CH_3×2). ^{13}C{^{1}H} NMR (125 MHz, $CDCl_3$): δ 157.1, 136.4, 130.2, 123.6, 120.5, 112.0, 68.2, 35.3, 27.8, 27.3, 22.6 (2C), 21.4, 15.8. HRMS (ESI-TOF) *m*/*z*: [M + H]$^+$ Calcd for $C_{14}H_{23}O$ 207.1749; Found 207.1756.

3-Ethylbenzophenone (2m).

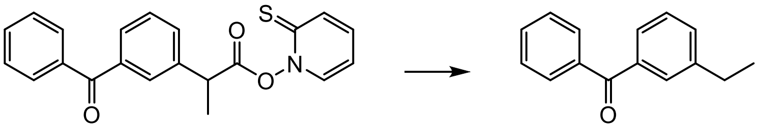

1m **2m**

As described for the preparation of **2a**, compound **1m** (31.2 mg, 85.9 µmol) was converted to 8.8 mg (49%) of **2m**. Compound **2m** was obtained as a colorless oil. TLC R_f 0.79

(EtOAc/hexane, 1:4). IR (KBr): 2965, 1660 cm^{-1}. ^1H NMR (500 MHz, $CDCl_3$): δ 7.81 (d, 2H, J =8.1 Hz, H-2, 6 of Bz), 7.66 (s, 1H, H-2 of 3-benzoylphenyl), 7.61-7.57 (m, 2H, H-4 of Bz, H-4 of 3-benzoylphenyl), 7.48 (t, 2H, J =8.1 Hz, H-3, 5 of Bz), 7.43 (d, 1H, J =7.8 Hz, H-6 of 3-benzoylphenyl), 7.39 (t, 1H, J =7.8 Hz, H-5 of 3-benzoylphenyl), 2.72 (q, 2H, J =7.7 Hz, -CH_2- of ethyl), 1.27 (t, 3H, J =7.7 Hz, -CH_3 of ethyl). $^{13}C\{^1H\}$ NMR (125 MHz, $CDCl_3$): δ 197.0, 144.5, 137.8, 137.7, 132.3, 132.0, 130.0 (2C), 129.3, 128.2 (2C), 128.1, 127.6, 28.7, 15.5. HRMS (ESI-TOF) m/z: $[M + H]^+$ Calcd for $C_{15}H_{15}O$ 211.1123; Found 211.1129.

(3,4,5,6-Tetrachlorophthalimidyloxycarbonyl)adamantane (2n).

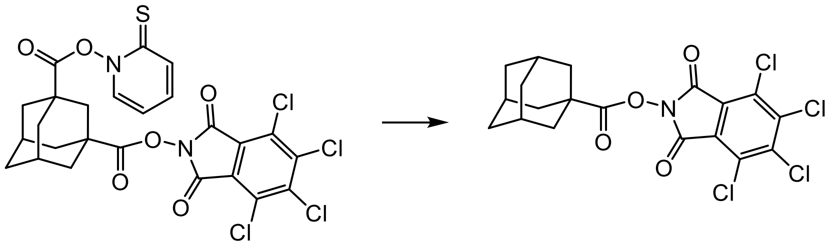

1n **2n**

As described for the preparation of **2a**, compound **1n** (38.6 mg, 62.6 μmol) was converted using CH_2Cl_2 instead of MeCN to 16.7 mg (58%) of **2n**. Compound **2n** was obtained as white crystals. mp 198-201 °C. TLC R_f 0.65 (EtOAc/hexane, 1:4). IR (KBr): 2914, 1809, 1783, 1747 cm^{-1}. ^1H NMR (500 MHz, $CDCl_3$): δ 2.12 (s, 9H), 1.78 (s, 6H). $^{13}C\{^1H\}$ NMR (125 MHz, $CDCl_3$): δ 172.7, 157.8 (2C), 140.9 (2C), 130.3 (2C), 124.8 (2C), 40.5, 38.4 (3C), 36.1 (3C), 27.6 (3C). HRMS (ESI-TOF) m/z: $[M + Na]^+$ Calcd for $C_{19}H_{15}Cl_4NNaO_4$ 483.9653; Found 483.9672.

1-Ethyl-4-[5-(phthalimidyloxycarbonyl)pentyloxy]benzene (2o).

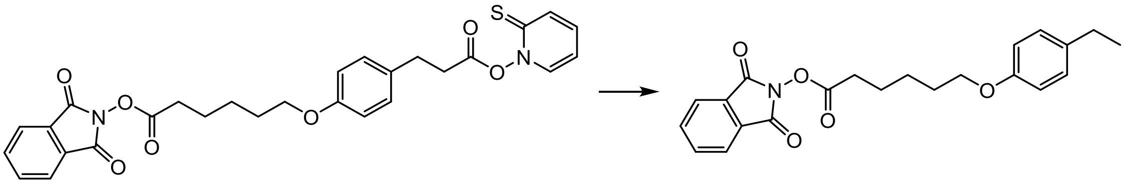

1o **2o**

As described for the preparation of **2a**, compound **1o** (89.7 mg, 0.168 mmol) was converted to 45.7 mg (71%) of **2o**. Compound **2o** was obtained as a colorless oil. TLC R_f 0.39 (EtOAc/hexane, 1:4). IR (neat): 2931, 1816, 1789, 1745 cm^{-1}. ^1H NMR (500 MHz, $CDCl_3$): δ 7.91-7.87 (m, 2H, H-3, 6 of phthalimidyl), 7.81-7.78 (m, 2H, H-4, 5 of phthalimidyl), 7.10 (d, 2H, J =8.8 Hz, H-2, 6), 6.83 (d, 2H, J =8.8 Hz, H-3, 5), 3.97 (t, 2H, J =6.3 Hz, H-1, 1' of pentyloxy), 2.71 (t, 2H, J =7.3 Hz, H-5, 5' of pentyloxy), 2.58 (q, J =7.8 Hz, 2H, -CH_2- of ethyl), 1.91-1.80 (m, 4H, H-2, 2', 4, 4' of pentyloxy), 1.68-1.60 (m, 2H, H-3, 3' of pentyloxy), 1.21 (t, J =7.8 Hz, 3H, -CH_3 of ethyl). $^{13}C\{^1H\}$ NMR (125 MHz, $CDCl_3$): δ 169.5, 162.0 (2C), 157.0, 136.3, 134.7 (2C), 128.9 (2C), 128.7 (2C), 124.0 (2C), 114.4 (2C), 67.5, 30.9, 28.8, 28.0, 25.4, 24.5, 15.9. HRMS (ESI-TOF) m/z: $[M + H]^+$ Calcd for $C_{22}H_{24}NO_5$ 382.1666;

Found 382.1654.

1-Ethyl-4-[5-(3,4,5,6-tetrachlorophthalimidyloxycarbonyl)pentyloxy]benzene (2p).

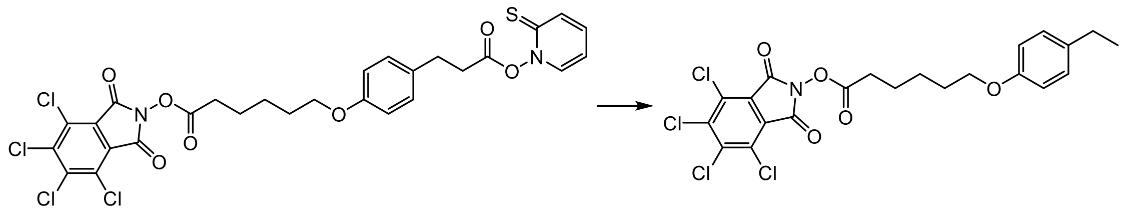

1p **2p**

As described for the preparation of **2a**, compound **1p** (62.3 mg, 92.7 µmol) was converted to 25.5 mg (53%) of **2p**. Compound **2p** was obtained as white crystals. mp 89-93 °C. TLC R_f 0.63 (EtOAc/hexane, 1:4). IR (KBr): 2934, 1816, 1790, 1748 cm^{-1}. ^{1}H NMR (400 MHz, $CDCl_3$): δ 7.10 (d, 2H, *J* =8.8 Hz, H-2, 6 of Ar), 6.82 (d, 2H, *J* =8.8 Hz, H-3, 5 of Ar), 3.96 (t, 2H, *J* =6.4 Hz, H-1, 1' of pentyloxy), 2.71 (t, 2H, *J* =7.6 Hz, H-5, 5' of pentyloxy), 2.58 (q, *J* =7.6 Hz, 2H, -CH_2- of ethyl), 1.92-1.79 (m, 4H, H-2, 2', 4, 4' of pentyloxy), 1.68-1.59 (m, 2H, H-3, 3' of pentyloxy), 1.21 (t, *J* =7.6 Hz, 3H, -CH_3 of ethyl). ^{13}C{^{1}H} NMR (100 MHz, $CDCl_3$): δ 177.6, 169.9 (2C), 154.9, 134.7, 128.9, 106.9, 91.6, 84.9, 70.4, 52.3, 51.0, 50.4, 48.2, 46.7, 39.8, 36.8, 34.9, 22.1, 20.8, 16.5, 14.6. HRMS (ESI-TOF) *m*/*z*: [M + H]$^+$ Calcd for $C_{22}H_{20}Cl_4NO_5$ 518.0096; Found 518.0092.

(1*R*,4a*S*,4b*R*,10a*S*)-7-Isopropyl-1,4a-dimethyl-1,2,3,4,4a,4b,5,6,10,10a-decahydrophenanthrene (2qA) and (1*S*,4a*S*,4b*R*,10a*S*)-7-Isopropyl-1,4a-dimethyl-1,2,3,4,4a,4b,5,6,10,10a-decahydrophenanthrene (2qB).

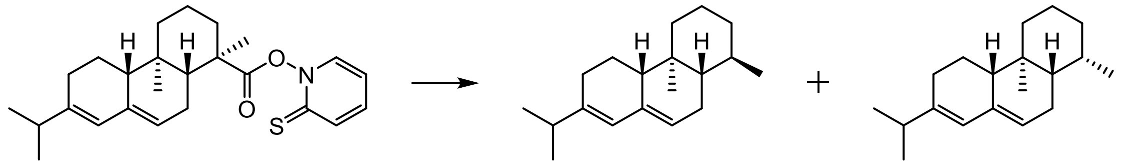

1q **2qA** **2qB**

As described for the preparation of **2a**, compound **1q** (10.0 mg, 26.6 µmol) was converted to a mixture of **2qA** and **2qB** (5.0 mg, 80%, dr = 3:2). A mixture of **2qA** and **2qB** was obtained as a colorless oil. TLC R_f 0.88 (hexane). IR (neat): 2923 cm^{-1}. ^{1}H NMR (500 MHz, $CDCl_3$): δ 5.78 (s, 1H, H-8), 5.44 (m, 1H×3/5, H-9), 5.40 (m, 1H×2/5, H-9), 2.30-2.12 (m, 2H), 2.11-2.04 (m, 2H), 1.89-1.76 (m, 4H), 1.68-1.40 (m, 5H), 1.39-1.12 (m, 2H), 1.03-0.99 (m, 7H), 0.98 (d, 3H×3/5, *J* = 7.5 Hz, -CH_3), 0.83 (d, 3H×2/5, *J* = 6.5 Hz, -CH_3), 0.78 (s, 3H×3/5, -CH_3), 0.72 (s, 3H×2/5, -CH_3). ^{13}C{^{1}H} NMR (125 MHz, $CDCl_3$) for major isomer: δ 145.1, 135.7, 122.6, 121.5, 50.4, 48.2, 44.0, 39.2, 34.9, 33.8, 32.1, 28.3, 27.5, 22.4, 21.4, 20.8, 17.4, 15.5, 14.4. HRMS (ESI-TOF) *m*/*z*: [M + H]$^+$ Calcd for $C_{19}H_{31}$ 259.2426; Found 259.2419.

(3*R*,5*R*,8*R*,9*S*,10*S*,13*R*,14*S*,17*R*)-3-Benzoyloxy-17-[(2*R*)-2-butyl)]-10,13-dimethyl-hexadecahydro-1*H*-cyclopenta[*a*]phenanthrene (2r).

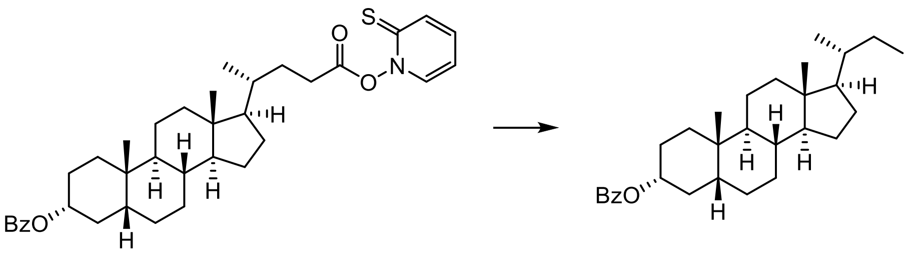

1r **2r**

As described for the preparation of **2a**, compound **1r** (10.2 mg, 17.3 μmol) was converted using benzene instead of MeCN to 5.6 mg (74%) of **2r**. Compound **2r** was obtained as white solids. TLC R_f 0.61 (EtOAc/hexane, 1:8). $[\alpha]^{25}_D$ +62.7 (*c* 0.665, $CHCl_3$). IR (neat): 2936, 1716 cm^{-1}. ^1H NMR (500 MHz, $CDCl_3$): δ 8.05 (d, 2H, *J* =7.5 Hz, H-2, 6 of Bz), 7.54 (t, 1H, *J* =7.5 Hz, H-4 of Bz), 7.43 (t, 2H, *J* =7.5 Hz, H-3, 5 of Bz), 4.98 (m, 1H, H-3), 2.02-1.96 (m, 2H), 1.92-1.77 (m, 4H), 1.68 (m, 1H), 1.61-1.38 (m, 8H) 1.35-1.18 (m, 5H), 1.16-1.00 (m, 6H), 0.96 (s, 3H, H-19), 0.90 (d, 3H, *J* =6.5 Hz, H-21), 0.82 (t, 3H, *J* =6.5 Hz, $-CH_2CH_3$), 0.66 (s, 3H, H-18). $^{13}C\{^1H\}$ NMR (125 MHz, $CDCl_3$): δ 166.1, 132.7, 130.9, 129.5 (2C), 128.2 (2C), 75.0, 56.5, 55.8, 42.6, 42.0, 40.5, 40.2, 37.0, 35.8, 35.1, 34.6, 32.4, 28.3, 28.2, 27.1, 26.7, 26.4, 24.2, 23.4, 20.9, 18.0, 12.0, 10.3. HRMS (ESI-TOF) *m*/*z*: $[M + H]^+$ Calcd for $C_{30}H_{45}O_2$ 437.3420; Found 437.3424.

(6*Z*,9*Z*)-6,9-Heptadecadiene (2s).

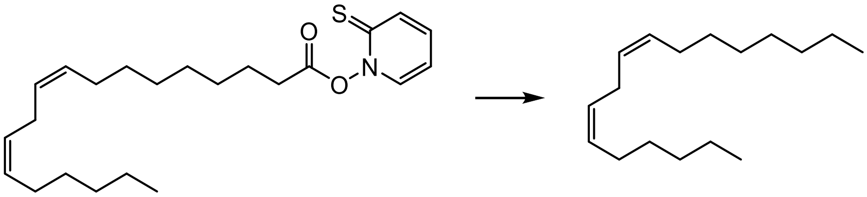

1s **2s**

As described for the preparation of **2a**, compound **1s** (10.0 mg, 26.6 μmol) was converted to 4.8 mg (81%) of **2s**. Compound **2s** was obtained as a colorless oil. TLC R_f 0.85 (hexane). IR (KBr): 2926 cm^{-1}. ^1H NMR (500 MHz, $CDCl_3$): δ 5.42-5.30 (m, 4H, H-6, 7, 9, 10), 2.78 (t, 2H, *J* = 6.8 Hz, H-8, 8'), 2.05 (q, 4H, *J* = 7.0 Hz, H-5, 5', 11, 11'), 1.39-1.24 (m, 16H), 0.92-0.86 (m, 6H, $-CH_3$). $^{13}C\{^1H\}$ NMR (125 MHz, $CDCl_3$): δ 130.2 (2C), 127.9 (2C), 31.9, 31.5, 29.7, 29.4, 29.3, 29.2, 27.2 (2C), 25.6, 22.7, 22.6, 14.1 (2C). HRMS (ESI-TOF) *m*/*z*: $[M + H]^+$ Calcd for $C_{17}H_{33}$ 237.2582; Found 237.2580.

7-Acetoxy-5-methoxy-4-methyl-6-[(2*E*)-3-methylpent-2-enyl]isobenzofuran-1(3*H*)-one (2t).

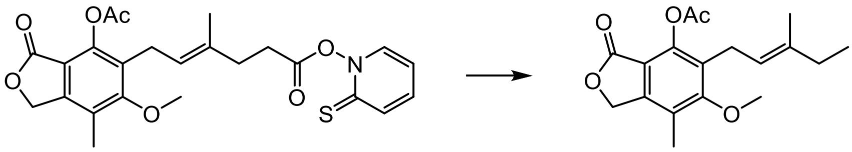

1t **2t**

As described for the preparation of **2a**, compound **1t** (15.8 mg, 33.5 μmol) was converted to 7.7 mg (72%) of **2t**. Compound **2t** was obtained as white crystals. mp 97-100 °C. TLC R_f 0.38 (EtOAc/hexane, 1:3). IR (KBr): 2966, 1777, 1762 cm^{-1}. ^{1}H NMR (400 MHz, $CDCl_3$): δ 5.15 (s, 2H, H-3, 3'), 5.05 (t, 1H, *J* =6.5 Hz, C=C*H*), 3.79 (s, 3H, OMe), 3.36 (d, 2H, *J* =6.5 Hz, C=CH-C*H*$_2$), 2.39 (s, 3H, OAc), 2.22 (s, 3H, C*H*$_3$ at C-4), 1.97 (q, 2H, *J* =7.3 Hz, -C*H*$_2$-CH$_3$), 1.77 (s, 3H, C*H*$_3$-C=CH), 0.95 (t, 3H, *J* =7.3 Hz, -CH$_2$-C*H*$_3$). ^{13}C{^{1}H} NMR (125 MHz, $CDCl_3$): δ 169.0, 168.3, 162.7, 146.0, 138.0, 129.6, 122.9, 120.0 (2C), 113.5, 68.3, 61.2, 32.2, 23.5, 20.5, 16.1, 12.5, 11.8. HRMS (ESI-TOF) *m*/*z*: [M + H]$^+$ Calcd for $C_{18}H_{23}O_5$ 319.1545; Found 319.1538.

(1*S*,2*S*,4a*R*,4b*R*,7*S*,9a*R*,10a*R*)-2,7-Diacetoxy-1-methyl-8-methylene-13-oxo-1,2,4,4b,5,6,7,8,9,10,10a-decahydro-4a,1-(epoxymethano)-7,9a-methanobenzo[*a*]azulene (2u).

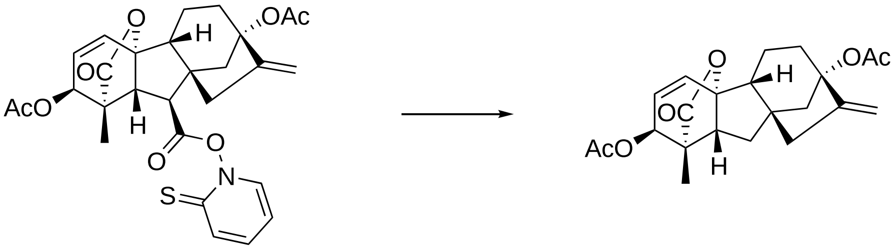

1u **2u**

As described for the preparation of **2a**, compound **1u** (19.8 mg, 36.7 μmol) was converted with 0.5 mol% of the catalyst to 7.6 mg (54%) of **2u**. Compound **2u** was obtained as white crystals. mp 155-159 °C. TLC R_f 0.48 (EtOAc/hexane, 1:1). $[\alpha]^{25}_{D}$ +157 (*c* 0.500, $CHCl_3$). IR (KBr): 2935, 1777, 1739 cm^{-1}. ^{1}H NMR (500 MHz, $CDCl_3$): δ 6.38 (d, 1H, *J* =9.4 Hz, H-4), 5.84 (dd, 1H, *J* =9.4, 3.7 Hz, H-3), 5.33 (d, 1H, *J* =3.7 Hz, H-2), 5.11 (s, 1H, H-12), 4.95 (s, 1H, H-12'), 2.83 (dd, 1H, *J* =11.0, 8.0 Hz, H-10a), 2.52 (dt, 1H, *J* =15.6, 3.0 Hz), 2.36 (dd, 1H, *J* =12.5, 8.0 Hz), 2.28 (d, 1H, *J* =16.0 Hz), 2.18 (dd, 1H, *J* =10.8, 2.3 Hz), 2.12 (d, 1H, *J* =7.0 Hz), 2.10 (s, 3H, OAc), 2.02 (s, 3H, OAc), 1.95 (dd, 2H, *J* =13.8, 7.3 Hz), 1.82-1.74 (m, 2H), 1.71-1.63 (m, 2H), 1.20 (s, 3H, H-14). ^{13}C{^{1}H} NMR (125 MHz, $CDCl_3$): δ 177.6, 169.9 (2C), 154.9, 134.7, 128.9, 106.9, 91.6, 84.9, 70.4, 52.3, 51.0, 50.4, 48.2, 46.7, 39.8, 36.8, 34.9, 22.1, 20.8, 16.5, 14.6. HRMS (ESI-TOF) *m*/*z*: [M + H]$^+$ Calcd for $C_{22}H_{27}O_6$ 387.1808; Found 387.1803.

(3a*S*,4*S*,6a*R*)-4-Butyltetrahydro-1*H*-thieno[3,4-d]imidazol-2(3*H*)-one (2v).

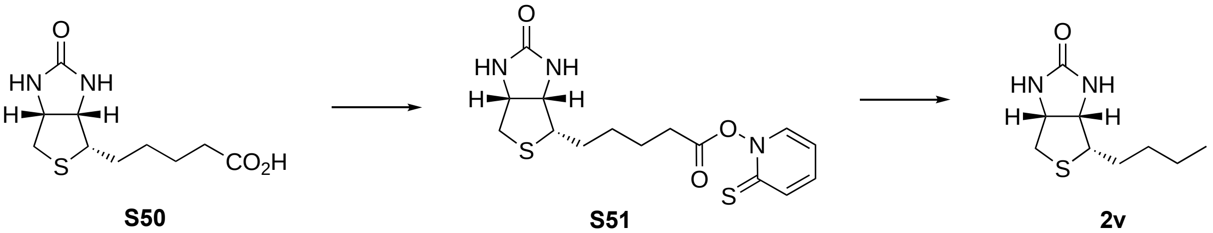

The following reaction was carried out under Ar and in the flask protected from light with aluminum foil. To a stirred solution of 2-mercaptopyridine *N*-oxide (20.7 mg, 0.163 mmol) and EDCI·HCl (31.2 mg, 0.163 mmol) in DMF (0.6 mL) was added **S50** (29.3 mg, 0.120 mmol). The mixture was stirred at room temperature for 1 h, and zinc(II) tetraphenylporphyrin (0.1 mg, 0.1 µmol) and *tert*-dodecanethiol (116 µL, 0.491 mmol) were added. After being stirred at 25 °C and irradiated by red LEDs for 30 min, the mixture was purified by column chromatography on silica gel (MeOH/CH_2Cl_2, 1:20) to provide 11.7 mg (49%) of **2v** as white crystals. mp 190-194 °C. TLC R_f 0.27 (MeOH/CH_2Cl_2, 1:12). $[\alpha]^{26}_{D}$ +66.7 (*c* 0.435, $CHCl_3$). IR (KBr): 3264, 2955, 1710 cm^{-1}. ^1H NMR (500 MHz, $CDCl_3$): δ 5.15 (br s, 1H, -NH-), 5.08 (br s, 1H, -NH-), 4.51 (m, 1H, H-3a), 4.31 (m, 1H, H-6a), 3.17 (m, 1H, H-4), 2.92 (dd, 1H, *J* =12.8, 5.2 Hz, H-6), 2.73 (d, 1H, *J* =12.8 Hz, H-6'), 1.72-1.62 (m, 2H, H-1, 1' of butyl), 1.46-1.30 (m, 4H, H-2, 2', 3, 3' of butyl), 0.91 (t, 3H, *J* =7.0 Hz, $-CH_3$). $^{13}C\{^1H\}$ NMR (125 MHz, $CDCl_3$): δ 163.1, 61.9, 60.1, 55.5, 40.6, 31.2, 28.3, 22.6, 13.9. HRMS (ESI-TOF) *m*/*z*: $[M + H]^+$ Calcd for $C_9H_{17}N_2OS$ 201.1062; Found 201.1070.

***N*-[(9*H*-Fluoren-9-yl)methoxycarbonyl]pyrrolidine (2w).**

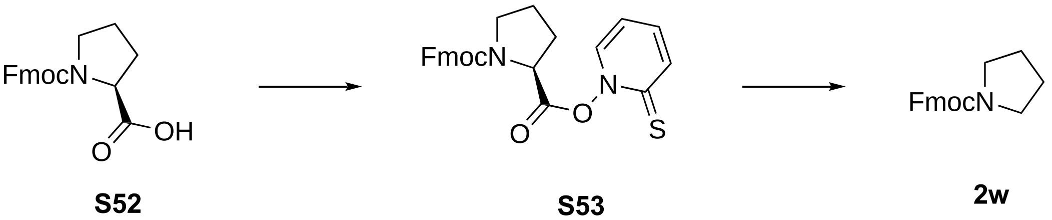

The following reaction was carried out under Ar and in the flask protected from light with aluminum foil. To a stirred solution of 2-mercaptopyridine *N*-oxide (21.5 mg, 0.169 mmol) and EDCI·HCl (32.0 mg, 0.167 mmol) in DMF (0.7 mL) was added **S52** (50.1 mg, 0.142 mmol). The mixture was stirred at room temperature for 1 h, and zinc(II) tetraphenylporphyrin (0.1 mg, 0.1 µmol) and *tert*-dodecanethiol (133 µL, 0.566 mmol) were added. After being stirred at 25 °C and irradiated by red LEDs for 30 min, the mixture was diluted with EtOAc (20 mL) and washed with H_2O (10 mL×3) and saturated brine (10 mL), sequentially. The organic layer was dried and concentrated under reduced pressure. The residue was purified by column chromatography on silica gel (EtOAc/hexane, 1:10) to provide 35.6 mg (86%) of **2w** as white crystals. mp 71-74 °C. TLC R_f 0.74 (EtOAc/hexane, 2:1). IR (KBr): 2875, 1710, 1694 cm^{-1}. ^1H NMR (500 MHz, $CDCl_3$): δ 7.77 (d, 2H, *J* =7.5 Hz, H-4, 5 of Fmoc), 7.62 (d, 2H, *J* =7.5 Hz, H-1, 8 of Fmoc), 7.40 (t, 2H, *J* =7.5 Hz, H-3, 6 of Fmoc), 7.32 (t, 2H, *J* =7.5 Hz, H-2, 7 of Fmoc), 4.38 (d, 2H, *J* =7.1 Hz, $-CH_2-$ of Fmoc), 4.25 (t, 1H, *J* =7.1 Hz, H-9 of Fmoc), 3.43 (t, 4H, *J* =6.5 Hz, H-2, 2', 5, 5'), 1.96-1.84 (m, 4H, H-3,

3’, 4, 4’). $^{13}C\{^1H\}$ NMR (125 MHz, $CDCl_3$): δ 154.9, 144.2 (2C), 141.3 (2C), 127.6 (2C), 127.0 (2C), 125.1 (2C), 119.9 (2C), 67.0, 47.4, 46.2, 45.8, 25.8, 24.9. HRMS (ESI-TOF) *m*/*z*: $[M + H]^+$ Calcd for $C_{19}H_{20}N_2O_2$ 294.1494; Found 294.1484.

***tert*-Butyl (9*H*-fluoren-9-yl)methyl pentane-1,5-diyldicarbamate (2x).**

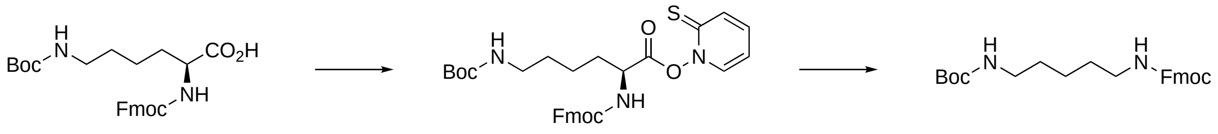

S54 **S55** **2x**

As described for the preparation of **2w**, compound **S54** (59.2 mg, 0.126 mmol) was converted to 43.7 mg (82%) of **2x**. Compound **2x** was obtained as white crystals. mp 110-112 °C. TLC R_f 0.35 (EtOAc/hexane, 1:2). IR (KBr): 2963, 1695, 1682 cm^{-1}. ^1H NMR (500 MHz, $CDCl_3$): δ 7.77 (d, 2H, *J* =7.6 Hz, H-4, 5 of Fmoc), 7.59 (d, 2H, *J* =7.6 Hz, H-1, 8 of Fmoc), 7.40 (t, 2H, *J* =7.6 Hz, H-3, 6 of Fmoc), 7.31 (t, 2H, *J* =7.6 Hz, H-2, 7 of Fmoc), 4.79 (br s, 1H, -NH-), 4.53 (br s, 1H, -NH-), 4.40 (d, 2H, *J* =6.8 Hz, $-CH_2-$ of Fmoc), 4.21 (t, 1H, *J* =6.8 Hz, H-9 of Fmoc), 3.19 (t, 2H, *J* =6.5 Hz, H-5, 5’), 3.15-3.00 (m, 2H, H-1, 1’), 1.57-1.46 (m, 4H, H-2, 2’, 4, 4’), 1.44 (s, 9H, *t*-Bu), 1.39-1.30 (m, 2H, H-3, 3’). $^{13}C\{^1H\}$ NMR (125 MHz, $CDCl_3$): δ 156.6, 156.2, 144.1 (2C), 141.4 (2C), 127.7 (2C), 127.1 (2C), 125.1 (2C), 120.1 (2C), 79.2, 66.6, 47.4, 40.9, 40.4, 29.8, 29.7, 28.5 (3C), 23.9. HRMS (ESI-TOF) *m*/*z*: $[M + H]^+$ Calcd for $C_{25}H_{33}N_2O_4$ 425.2440; Found 425.2444.

***tert*-Butyl 3-[(9*H*-fluoren-9-yl)methoxycarbonylamino]propanoate (2y).**

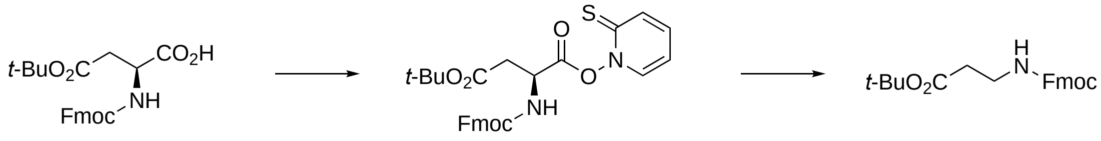

S56 **S57** **2y**

As described for the preparation of **2w**, compound **S56** (59.3 mg, 0.144 mmol) was converted to 45.3 mg (85%) of **2y**. Compound **2y** was obtained as white crystals. mp 39-42 °C. TLC R_f 0.53 (EtOAc/hexane, 1:9). IR (KBr): 2977, 1731, 1692 cm^{-1}. ^1H NMR (500 MHz, $CDCl_3$): δ 7.76 (d, 2H, *J* =7.5 Hz, H-4, 5 of Fmoc), 7.59 (d, 2H, *J* =7.5 Hz, H-1, 8 of Fmoc), 7.40 (t, 2H, *J* =7.5 Hz, H-3, 6 of Fmoc), 7.31 (t, 2H, *J* =7.5 Hz, H-2, 7 of Fmoc), 5.32 (br s, 1H, -NH-), 4.38 (d, 2H, *J* =7.1 Hz, $-CH_2-$ of Fmoc), 4.22 (t, 1H, *J* =7.1 Hz, H-9 of Fmoc), 3.44 (t, 2H, *J* =6.0 Hz, H-3, 3’), 2.47 (t, 2H, *J* =6.0 Hz, H-2, 2’), 1.47 (s, 9H, *t*-Bu). $^{13}C\{^1H\}$ NMR (125 MHz, $CDCl_3$): δ 171.8, 156.3, 143.9 (2C), 141.3 (2C), 127.6 (2C), 127.0 (2C), 125.0 (2C), 119.9 (2C), 81.1, 66.7, 47.2, 36.7, 35.5, 28.1 (3C). HRMS (ESI-TOF) *m*/*z*: $[M + H]^+$ Calcd for $C_{22}H_{26}NO_4$ 368.1862; Found 368.1871.

2-thioxopyridin-1(2*H*)-yl 5-phenylpentanoate (3).

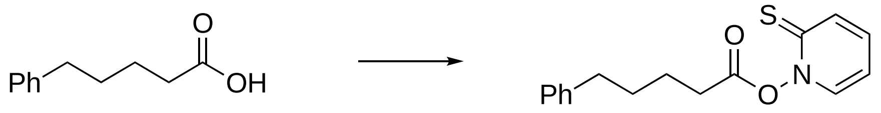

S3 **3**

As described for the preparation of **1b**, valeric acid **S3** (507 mg, 2.81 mmol) was converted to 619 mg (77%) of **3**. Compound **3** was obtained as a yellow oil. TLC R_f 0.24 (EtOAc/hexane, 1:2). IR (neat): 2942, 2847, 1800 cm^{-1}. ^{1}H NMR (400 MHz, $CDCl_3$): δ 7.69 (dd, 1H, J = 8.8, 1.3 Hz, H-6 of 2-pyridinethione), 7.53 (dd, 1H, J = 6.8, 1.3, H-3 of 2-pyridinethione), 7.31-7.26 (m, 2H, phenyl), 7.23-7.16 (m, 4H, phenyl, H-5 of 2-pyridinethione), 6.62 (td, 1H, J = 6.8, 1.3 Hz, H-4 of 2-pyridinethione), 2.74 (t, 2H, J = 8.0 Hz, H-2, 2'), 2.68 (t, 2H, J = 8.0 Hz, H-5, 5'), 1.91-1.74 (m, 4H, H-3, 3', 4, 4'). ^{13}C{^{1}H} NMR (100 MHz, $CDCl_3$): δ 175.7, 168.8, 141.7, 137.6, 137.2, 133.5, 128.3 (4C), 125.8, 112.5, 35.3, 31.3, 30.5, 23.8. HRMS (ESI-TOF) *m/z*: [M + H]$^+$ Calcd for $C_{16}H_{18}NO_2S$ 288.1057; Found 288.1058.

(4-Chlorobutyl)benzene (4a).

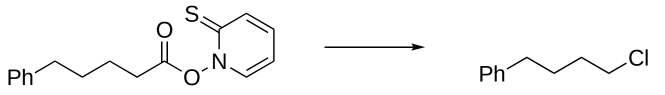

3 **4a**

The following reaction was carried out under Ar. To a stirred solution of **3** (20.0 mg, 0.0696 mmol), zinc(II) tetraphenylporphyrin (1.4 mg, 2.1 µmol) in carbon tetrachloride (1.4 mL) was added hexachloroethane (49.4 mg, 0.208 mmol). The stirred mixture was irradiated by red LEDs at 25 °C for 1 h, the mixture concentrated under reduced pressure. The residue was purified by PTLC (EtOAc/hexane, 1:10) to provide 8.5 mg (73%) of **4a** as a colorless oil. TLC R_f 0.78 (EtOAc/hexane, 1:10). IR (neat): 2939 cm^{-1}. ^{1}H NMR (400 MHz, $CDCl_3$): *δ* 7.31-7.25 (m, 2H, -Ph), 7.22-7.16 (m, 3H, -Ph), 3.55 (t, 2H, J = 6.4 Hz, H-4, 4' of butyl), 2.65 (t, 2H, J = 8.0 Hz, H-1, 1' of butyl), 1.86-1.73 (m, 4H, H-2, 2', 3, 3' of butyl). ^{13}C{^{1}H} NMR (125 MHz, $CDCl_3$): δ 141.8, 128.4 (4C), 125.9, 44.9, 35.1, 32.1, 28.5. HRMS (ESI-TOF) *m/z*: [M + H]$^+$ Calcd for $C_{10}H_{14}Cl$ 169.0784; Found 169.0781.

(4-Bromobutyl)benzene (4b).

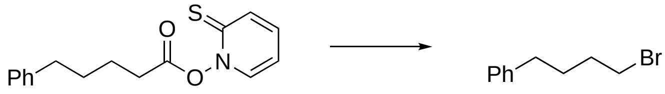

3 **4b**

The following reaction was carried out under Ar. To a stirred solution of **3** (20.0 mg, 0.0696 mmol), zinc(II) tetraphenylporphyrin (1.4 mg, 2.1 µmol) in toluene (1.4 mL) was added bromotrichloromethane (21 mL, 0.21 mmol). The stirred mixture was irradiated by red LEDs at 25 °C for 1 h, the mixture concentrated under reduced pressure. The residue was purified by PTLC (EtOAc/hexane, 1:10) to provide 8.1 mg (55%) of **4b** as a yellow oil. TLC R_f 0.78 (EtOAc/hexane, 1:10). IR (neat): 2936 cm^{-1}. ^{1}H NMR (400 MHz, $CDCl_3$): δ 7.32-7.26 (m, 2H, -Ph), 7.22-7.16 (m, 3H, -Ph), 3.42 (t, 2H, J = 6.8 Hz, H-4, 4' of butyl), 2.64 (t, 2H, J = 7.6 Hz,

H-1, 1' of butyl), 1.94-1.73 (m, 4H, H-2, 2', 3, 3' of butyl). $^{13}C\{^1H\}$ NMR (125 MHz, $CDCl_3$): δ 142.9, 128.4 (2C), 128.2 (2C), 125.5, 82.9, 35.8, 34.2, 24.8, 23.8. HRMS (ESI-TOF) *m/z*: $[M + H]^+$ Calcd for $C_{10}H_{14}Br$ 213.0279; Found 213.0275.

(4-Iodobutyl)benzene (4c).

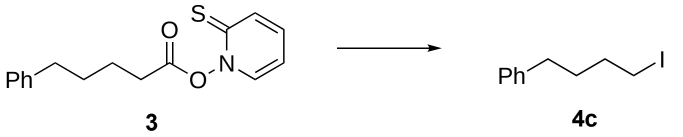

The following reaction was carried out under Ar. To a stirred solution of **3** (20.0 mg, 0.0696 mmol), zinc(II) tetraphenylporphyrin (1.4 mg, 2.1 µmol) in toluene (1.4 mL) was added diiodomethane (17 mL, 0.21 mmol). The stirred mixture was irradiated by red LEDs at 25 °C for 1 h, the mixture concentrated under reduced pressure. The residue was purified by PTLC (EtOAc/hexane, 1:10) to provide 12.6 mg (70%) of **4c** as a yellow oil. TLC R_f 0.78 (EtOAc/hexane, 1:10). IR (neat): 2933 cm^{-1}. ^1H NMR (400 MHz, $CDCl_3$): δ 7.32-7.26 (m, 2H, -Ph), 7.21-7.16 (m, 3H, -Ph), 3.20 (t, 2H, *J* = 7.6 Hz, H-4, 4' of butyl), 2.64 (t, 2H, *J* = 8.8 Hz, H-1, 1' of butyl), 1.89-1.71 (m, 4H, H-2, 2', 3, 3' of butyl). $^{13}C\{^1H\}$ NMR (125 MHz, $CDCl_3$): δ 141.8, 128.4 (4C), 125.9, 34.7, 32.9, 32.2, 6.7. HRMS (ESI-TOF) *m/z*: $[M + H]^+$ Calcd for $C_{10}H_{14}I$ 261.0140; Found 261.0152.

4-Phenyl-1-butanol (4d).

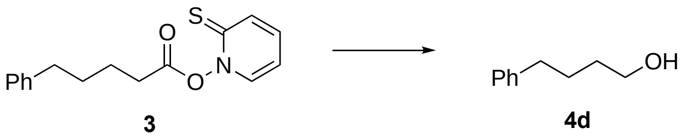

The following reaction was carried out under O_2. To a stirred solution of **3** (10.2 mg, 0.0355 mmol) in ethanol (0.7 mL) was added zinc(II) tetraphenylporphyrin (0.71 mg, 0.001 mmol) and *t*-butyl mercaptan (35 µL, 0.31 mmol). The stirred mixture was irradiated by red LEDs at 25 °C for 30 min. The reaction flask was replaced to Ar, and trimethyl phosphite (9 µL, 0.076 mmol) was added and stirred for additional 2 h. The mixture was diluted with H_2O (10 mL) and extracted with EtOAc (3 mL×3). The combined organic layer was dried and concentrated under reduced pressure. The residue was purified by PTLC (EtOAc/hexane, 1:2) to provide 4.0 mg (75%) of **4d** as a yellow oil. TLC R_f 0.40 (EtOAc/hexane, 1:2). ^1H NMR (400 MHz, $CDCl_3$): δ 7.30-7.26 (m, 2H, -Ph), 7.19-7.15 (m, 3H, -Ph), 3.66 (t, 2H, *J* = 6.4 Hz, H-1, 1'), 2.64 (t, 2H, *J* = 7.6 Hz, H-4, 4'), 1.74-1.58 (m, 4H, H-2, 2', 3, 3'). **4d** is a commercially available compound.

Dimer of (1-nitroso-4-phenylbutane) (4e).

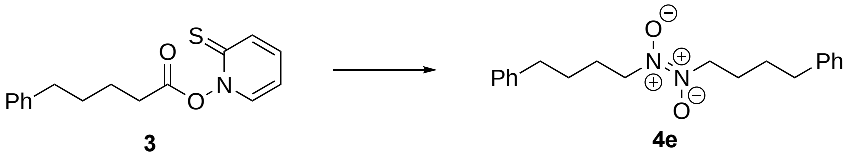

The following reaction was carried out under Ar. To a stirred solution of **3** (10.1 mg, 0.0352 mmol), zinc(II) tetraphenylporphyrin (0.71 mg, 0.001 μmol) in dichloromethane/toluene (2:1, 0.7 mL) was added *S*-trityl nitrothioite (17 mL, 0.21 mmol).[S1] The stirred mixture was irradiated by red LEDs at 25 °C for 1.5 h, The mixture was diluted with H_2O (10 mL) and extracted with EtOAc (3 mL×3). The combined organic layer was dried and concentrated under reduced pressure. The residue was purified by PTLC (EtOAc/hexane, 1:4) to provide 3.5 mg (61%) of **4e** as a yellow oil. TLC R_f 0.20 (EtOAc/hexane, 1:4). IR (neat): 2927, 1454, 1221 cm^{-1}. ^{1}H NMR (400 MHz, $CDCl_3$): δ 7.30-7.14 (m, 10H, -Ph×2), 4.27 (t, 4H, *J* = 7.2 Hz, H-1, 1'×2), 2.66 (t, 4H, *J* = 7.6 Hz, H-4, 4'×2), 1.91 (tt, 4H, *J* = 7.2, 7.2 Hz, H-2, 2'×2), 1.70 (tt, 4H, *J* = 7.6, 7.2 Hz, H-3, 3'×2). ^{13}C{^{1}H} NMR (100 MHz, $CDCl_3$): δ 141.5 (2C), 128.40 (4C), 128.37 (4C), 126.0 (2C), 58.6 (2C), 35.2(2C), 28.3 (2C), 24.7 (2C). HRMS (ESI-TOF) *m/z*: [M + Na]$^+$ Calcd for $C_{20}H_{26}N_2NaO_2$ 349.1892; Found 349.1885.

Diethyl 1-(4-phenylbutyl)hydrazine-1,2-dicarboxylate (4f).

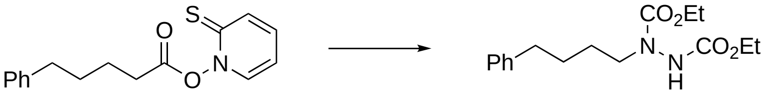

3 **4f**

The following reaction was carried out under Ar. To a stirred solution of **3** (10.5 mg, 0.0365 mmol), zinc(II) tetraphenylporphyrin (0.71 mg, 0.001 μmol) in toluene (0.35 mL) was added diethyl azodicarboxylate in toluene (16 μL, 0.035 mmol) and tris(trimethylsilyl)silane (43 μL, 0.035 mmol). The stirred mixture was irradiated by red LEDs at 25 °C for 1.5 h, The mixture was diluted with H_2O (10 mL) and extracted with EtOAc (3 mL×3). The combined organic layer was dried and concentrated under reduced pressure. The residue was purified by PTLC (EtOAc/hexane, 1:2) to provide 6.4 mg (57%) of **4f** as a yellow oil. TLC R_f 0.40 (EtOAc/hexane, 1:2). IR (neat): 2936, 1712 cm^{-1}. ^{1}H NMR (400 MHz, $CDCl_3$): δ 7.30-7.25 (m, 2H, -Ph), 7.20-7.15 (m, 3H, -Ph), 6.44 (br, 1H, NH), 4.22-4.14 (m, 4H, CH_2 of CO_2Et×2), 3.52 (br s, 2H, H-1,1'), 2.63 (t, 2H, *J* = 6.8 Hz, H-4, 4'), 1.64-1.60 (m, 4H, H-2, 2', 3, 3'), 1.29-1.23 (m, 6H, CH_3 of CO_2Et×2). ^{13}C{^{1}H} NMR (100 MHz, $CDCl_3$): δ 156.3 (br, 2C), 142.1, 128.4 (2C), 128.3 (2C), 125.8, 62.4, 62.0, 49.7 (br), 35.5, 28.3, 26.9, 14.5, 14.4. HRMS (ESI-TOF) *m/z*: [M + Na]$^+$ Calcd for $C_{16}H_{24}N_2NaO_4$ 331.1634; Found 331.1628.

Phenyl(4-phenylbutyl)sulfane (4g)

[S1] van Zwet, H.; Kooyman, E. C. *Recl. Trav. Chim. Pays-bas* **1968**, *87*, 45-48.

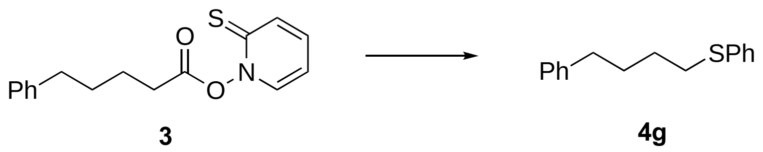

The following reaction was carried out under Ar. To a stirred solution of **3** (11.0 mg, 0.0383 mmol) in DMSO (0.8 mL) were added zinc(II) tetraphenylporphyrin (0.78 mg, 0.001 mmol) and diphenyl disulfide (17.0 mg, 0.0779 mmol). The stirred mixture was irradiated by red LEDs at 25 °C for 1.5 h, diluted with H_2O (10 mL) and extracted with EtOAc (3 mL × 3). The combined extracts were dried and concentrated under reduced pressure. The residue was purified by PTLC (EtOAC/hexane, 1:40) to provide 7.7 mg (84%) of **4g** as a colorless oil. TLC R_f 0.38 (EtOAc/hexane, 1:40). IR (neat): 2933, 1480 cm^{-1}. ^1H NMR (400 MHz, $CDCl_3$): δ 7.32-7.23 (m, 6H, phenyl), 7.20-7.14 (m, 4H, phenyl), 2.93 (t, 2H, J = 7.2 Hz, H-1,1'), 2.62 (t, 2H, J = 7.2 Hz, H-4,4'), 1.81-1.64 (m, 4H, H-2,2',3,3'). $^{13}C\{^1H\}$ NMR (100 MHz, $CDCl_3$): δ 142.1, 136.8, 129.0 (2C), 128.8 (2C), 128.4 (2C), 128.3 (2C), 125.8, 125.7, 35.4, 33.5, 30.4, 28.6. HRMS (ESI-TOF) *m/z*: $[M + Na]^+$ Calcd for $C_{16}H_{18}NaS$ 265.1027; Found 265.1028.

(4-Phenylbutyl)seleno benzene (4h)

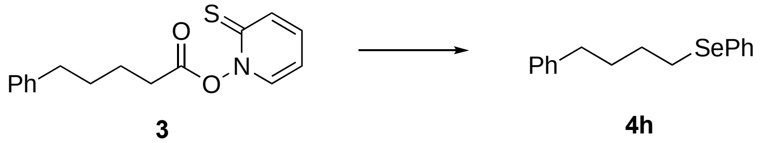

The following reaction was carried out under Ar. To a stirred solution of **3** (20.0 mg, 0.0696 mmol), zinc(II) tetraphenylporphyrin (1.4 mg, 2.1 μmol) in CH_2Cl_2 (1.4 mL) was added diphenyl diselenide (21.7 mg, 0.0696 mmol). The stirred mixture was irradiated by red LEDs at 25 °C for 1 h, the mixture concentrated under reduced pressure. The residue was purified by PTLC (EtOAc/hexane, 1:8) to provide 18.3 mg (91%) of **4h** as a yellow oil. TLC R_f 0.75 (toluene/hexane, 1:3). IR (neat): 2999 cm^{-1}. ^1H NMR (400 MHz, $CDCl_3$): δ 7.50-7.44 (m, 2H, -Ph), 7.30-7.21 (m, 5H, -Ph), 7.20-7.12 (m, 3H, -Ph), 2.92 (t, 2H, J = 8.0 Hz, H-4, 4' of butyl), 2.61 (t, 2H, J = 8.8 Hz, H-1, 1' of butyl), 1.80-1.70 (m, 4H, H-2, 2', 3, 3' of butyl). $^{13}C\{^1H\}$ NMR (125 MHz, $CDCl_3$): δ 142.1, 132.5 (2C), 130.5, 129.0 (2C), 128.4 (2C), 128.3 (2C), 126.7, 125.7, 35.3, 31.4, 29.6, 27.7. HRMS (ESI-TOF) *m/z*: $[M + H]^+$ Calcd for $C_{16}H_{19}Se$ 291.0652; Found 291.0642.

4,4,5,5-Tetramethyl-2-(4-phenylbutyl)-1,3,2-dioxaborolane (4i)

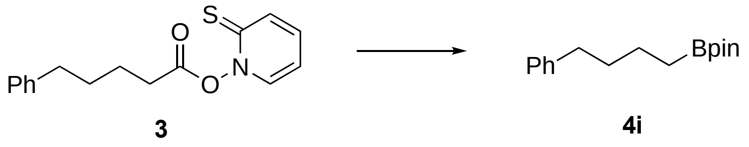

The following reaction was carried out under Ar. To a stirred solution of **3** (20.0 mg, 0.0696 mmol), zinc(II) tetraphenylporphyrin (1.4 mg, 2.1 μmol) in DMF (2.8 mL) was added bis(catecholato)diboron (33.1 mg, 0.139 mmol). The stirred mixture was irradiated by red LEDs at 25 °C for 1 h, and pinacol (32.9

mg, 0.278 mmol) in triethylamine (0.1 mL) were added. After being stirred at room temperature for 1 h, the mixture was diluted with EtOAc (15 mL) and washed with saturated brine (10 mL×2) and H_2O (10 mL×2) saturated brine (10 mL×2), sequentially. The organic layer was dried and concentrated under reduced pressure. The residue was purified by columchlomatography (toluene) to provide 12.1 mg (67%) of **4i** as a brown oil. TLC R_f 0.65 (EtOAc/hexane, 1:10). IR (neat): 2928 cm^{-1}. ^1H NMR (400 MHz, $CDCl_3$): δ 7.29-7.24 (m, 2H, -Ph), 7.20-7.16 (m, 3H, -Ph), 2.60 (t, 2H, J = 9.6 Hz, H-1, 1' of butyl), 1.67-1.57 (m, 2H, H-2, 2' of butyl), 1.52-1.43 (m, 2H, -3, 3' of butyl), 1.24 (s, 12H, -Me×4 of dioxaborolane), 0.81 (t, 2H, J = 8.0 Hz, H-4, 4' of butyl). $^{13}C\{^1H\}$ NMR (125 MHz, $CDCl_3$): δ 142.9, 128.4 (2C), 128.2 (2C), 125.5, 82.9 (2C), 35.8, 34.2, 24.8 (4C), 23.8. HRMS (ESI-TOF) *m/z*: $[M + H]^+$ Calcd for $C_{16}H_{26}BO_2$ 261.2026; Found 261.2038.

[4-(Methylseleno)butyl]benzene (S7)

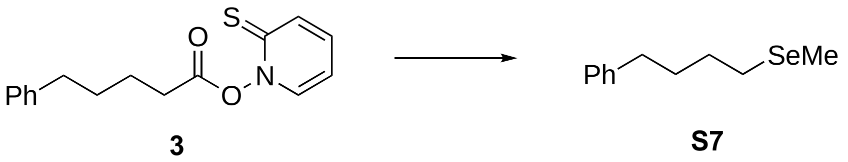

The following reaction was carried out under Ar. To a stirred solution of **3** (20.0 mg, 0.0696 mmol), zinc(II) tetraphenylporphyrin (1.4 mg, 2.1 μmol) in CH_2Cl_2 (1.4 mL) was added dimetyl diselenide (7 mL, 0.0696 mmol). The stirred mixture was irradiated by red LEDs at 25 °C for 1 h, the mixture concentrated under reduced pressure. The residue was purified by PTLC (EtOAc/hexane, 1:10) to provide 14.1 mg (89%) of **S7** as a yellow oil. TLC R_f 0.71 (EtOAc/hexane, 1:10). IR (neat): 2925 cm^{-1}. ^1H NMR (400 MHz, $CDCl_3$): δ 7.30-7.25 (m, 2H, -Ph), 7.20-7.15 (m, 3H, -Ph), 2.63 (t, 2H, J = 7.0 Hz, H-1, 1' of butyl), 2.56 (t, 2H, J = 7.0 Hz, H-4, 4' of butyl), 1.97 (s, 3H, Me), 1.78-1.67 (m, 4H, H-2, 2', 3, 3' of butyl). $^{13}C\{^1H\}$ NMR (125 MHz, $CDCl_3$): δ 142.2, 128.4 (2C), 128.3 (2C), 125.7, 35.4, 31.5, 29.7, 25.2, 4.0. HRMS (ESI-TOF) *m/z*: $[M + H]^+$ Calcd for $C_{11}H_{17}Se$ 229.0495; Found 229.0497.

1-Chloro-4-[(4-phenylbutyl)seleno]benzene (S8)

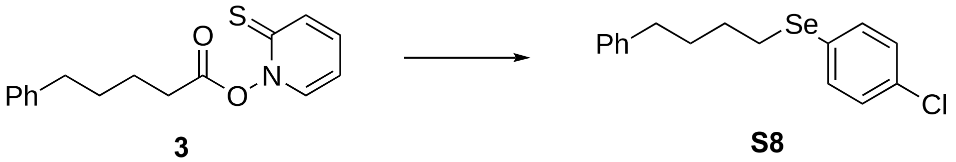

The following reaction was carried out under Ar. To a stirred solution of **3** (20.2 mg, 0.0696 mmol), zinc(II) tetraphenylporphyrin (1.4 mg, 2.1 μmol) in CH_2Cl_2 (1.4 mL) was added bis(4-chlorophenyl) diselenide (26.5 mg, 0.0696 mmol). The stirred mixture was irradiated by red LEDs at 25 °C for 1 h, the mixture concentrated under reduced pressure. The residue was purified by PTLC (toluene/hexane, 1:3) to provide 19.1 mg (85%) of **S8** as a yellow oil. TLC R_f 0.40 (toluene/hexane, 1:10). IR (neat): 2932 cm^{-1}. ^1H NMR (400 MHz, $CDCl_3$): δ 7.42-7.36 (m, 2H, -Ar), 7.30-7.25 (m, 2H, -Ph), 7.24-7.18 (m, 3H, -Ph), 7.17-7.12 (m, 2H, -Ar), 2.89 (t, 2H, J = 7.2 Hz, H-1, 1' of butyl), 2.61 (t, 2H, J = 7.0 Hz, H-4, 4' of butyl), 1.77-1.71 (m, 4H,

H-2, 2', 3, 3' of butyl). $^{13}C\{^1H\}$ NMR (100 MHz, $CDCl_3$): δ 142.0, 134.0 (2C), 132.9, 129.1 (2C), 128.5, 128.4 (2C), 128.3 (2C), 125.8, 35.4, 31.5, 29.7, 28.3. HRMS (ESI-TOF) *m/z*: $[M + H]^+$ Calcd for $C_{16}H_{18}ClSe$ 325.0262; Found 325.0264.

2-Thioxopyridin-1(2*H*)-yl 2-phenethyl-4-phenylbutanoate (5)

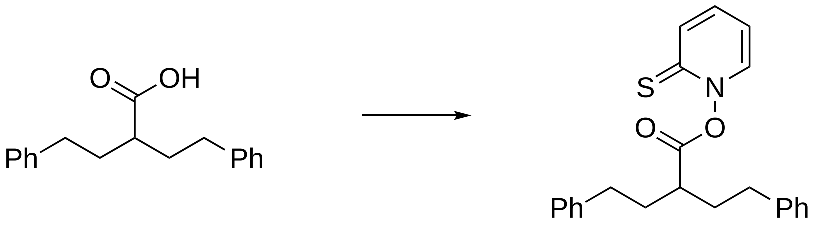

S58 **5**

As described for the preparation of **1b**, compound **S58**[S2] (100 mg, 0.420 mmol) was converted to 113 mg (80%) of **5**. Compound **5** was obtained as a yellow oil. TLC R_f 0.33 (EtOAc/hexane, 1:2). IR (neat): 1798, 1527, 1448 cm^{-1}. ^1H NMR (400 MHz, $CDCl_3$): δ 7.70 (m, 1H, 2-pyridinethione), 7.32-7.25 (m, 4H, 2-pyridinethione, phenyl), 7.24-7.17 (m, 8H, 2-pyridinethione, phenyl), 6.59 (m, 1H, 2-pyridinethione), 2.88-2.76 (m, 5H), 2.36-2.25 (m, 2H), 2.08-1.98 (m, 2H). $^{13}C\{^1H\}$ NMR (100 MHz, $CDCl_3$): δ 176.0, 171.2, 141.0 (2C), 137.60, 137.56, 133.2, 128.6 (4C), 128.5 (4C), 126.1 (2C), 112.4, 42.2, 33.3 (4C). HRMS (ESI-TOF) *m/z*: $[M + Na]^+$ Calcd for $C_{23}H_{23}NNaO_2S$ 400.1347; Found 400.1338.

Methyl 4-phenethyl-6-phenyl-2-(pyridin-2-ylthio)hexanoate (7a)

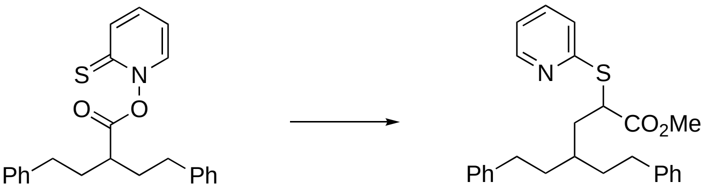

5 **7a**

The following reaction was carried out under Ar. To a stirred solution of **5** (10.4 mg, 0.0276 mmol) and zinc(II) tetraphenylporphyrin (0.6 mg, 0.9 μmol) in toluene (0.3 mL) was added methyl acrylate **6a** (5.0 μL, 0.073 mmol). The mixture was stirred and irradiated by red LEDs at 25 °C for 1 h, diluted with H_2O (10 mL) and extracted with CH_2Cl_2 (5 mL × 3). The combined extracts were dried and concentrated under reduced pressure. The residue was purified by PTLC (EtOAC/hexane, 1:2) to provide 7.7 mg (67%) of **7a** as a yellow oil. TLC R_f 0.76 (EtOAc/hexane, 1:2). IR (neat): 2927, 1736, 1454 cm^{-1}. ^1H NMR (400 MHz, $CDCl_3$): δ 8.39 (m, 1H), 7.49 (m, 1H), 7.30-7.22 (m, 4H), 7.20-7.13 (m, 7H), 7.00 (m, 1H), 4.75 (t, 1H, *J* = 8.0 Hz), 3.68 (s, 3H), 2.71-2.56 (m, 4H), 2.10 (m, 1H), 1.93 (m, 1H), 1.79-1.62 (m, 5H). $^{13}C\{^1H\}$ NMR (100 MHz, $CDCl_3$): δ 173.3, 157.1, 149.4, 142.6, 142.5, 136.1, 128.39 (2C),

[S2] Blankson, G. A.; Parhi, A. K.; Kaul, M.; Pilch, D. S.; LaVoie, E. J. *Bioorg. Med. Chem.* **2019**, *27,* 3254-3278.

128.35 (2C), 128.32 (2C), 128.29 (2C), 125.7 (2C), 122.2, 119.9, 52.4, 44.2, 36.2, 35.3, 35.1, 35.0, 32.7, 32.5. HRMS (ESI-TOF) *m/z*: [M + Na]$^+$ Calcd for $C_{26}H_{29}NNaO_2S$ 442.1817; Found 442.1806.

***tert*-Butyl 4-phenethyl-6-phenyl-2-(pyridin-2-ylthio)hexanoate (7b)**

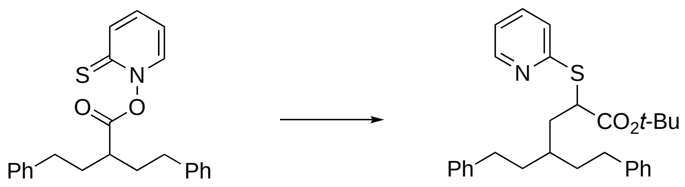

5 **7b**

As described for the preparation of **7a**, compound **5** (10.8mg, 0.0286mmol) was converted by using *t*-butyl acrylate **6b** instead of methyl acrylate to 6.1 mg (50%) of **7b**. Compound **7b** was obtained as a yellow oil. TLC R_f 0.86 (EtOAc/hexane, 1:2). IR (neat): 2931, 1726, 1146 cm^{-1}. ^{1}H NMR (400 MHz, $CDCl_3$): δ 8.39 (m, 1H), 7.48 (m, 1H), 7.31-7.24 (m, 4H), 7.22-7.14 (m, 7H), 6.98 (m, 1H), 4.56 (m, 1H), 2.74-2.57 (m, 4H), 2.07 (m, 1H), 1.88 (m, 1H), 1.82-1.66 (m, 5H), 1.39 (s, 9H). ^{13}C{^{1}H} NMR (100 MHz, $CDCl_3$): δ 171.7, 157.7, 149.3, 142.7, 142.6, 136.0, 128.38 (2C), 128.36 (2C), 128.33 (2C), 128.30 (2C), 125.7 (2C), 122.4, 119.8, 81.3, 45.8, 36.3, 35.4, 35.3, 35.0, 32.8, 32.5, 27.9 (3C). HRMS (ESI-TOF) *m/z*: [M + Na]$^+$ Calcd for $C_{29}H_{35}NNaO_2S$ 484.2286; Found 484.2275.

5-Phenethyl-7-phenyl-3-(pyridin-2-ylthio)heptan-2-one (7c)

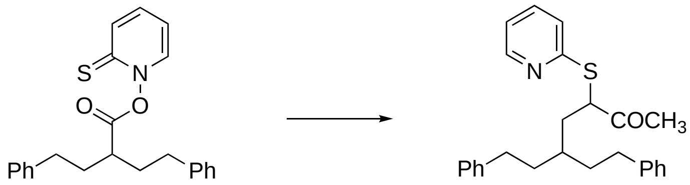

5 **7c**

As described for the preparation of **7a**, compound **5** (10.4mg, 0.0276mmol) was converted by using methyl vinyl ketone **6c** instead of methyl acrylate to 7.2 mg (65%) of **7c**. Compound **7c** was obtained as a yellow oil. TLC R_f 0.75 (EtOAc/hexane, 1:2). IR (neat): 2925, 1712, 1453 cm^{-1}. ^{1}H NMR (400 MHz, $CDCl_3$): δ 8.40 (m, 1H), 7.50 (m, 1H), 7.30-7.22 (m, 4H), 7.21-7.11 (m, 7H), 7.02 (m, 1H), 4.79 (m, 1H), 2.67-2.50 (m, 4H), 2.25 (s, 3H), 2.03 (m, 1H), 1.85-1.60 (m, 6H). ^{13}C{^{1}H} NMR (100 MHz, $CDCl_3$): δ 206.5, 157.1, 149.3, 142.5 (2C), 136.2, 128.4 (2C), 128.34 (4C), 128.29 (2C), 125.71, 125.67, 122.3, 120.0, 50.1, 35.5, 35.3, 34.8, 34.2, 32.7 (2C), 27.8. HRMS (ESI-TOF) *m/z*: [M + Na]$^+$ Calcd for $C_{26}H_{29}NNaOS$ 426.1868; Found 426.1855.

4-Phenethyl-6-phenyl-2-(pyridin-2-ylthio)hexanenitrile (7d)

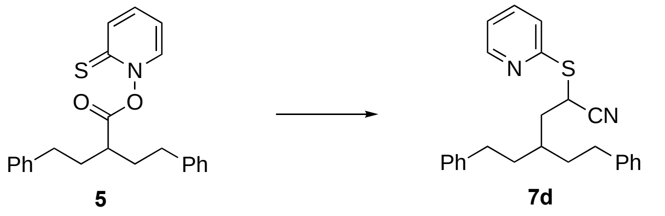

As described for the preparation of **7a**, compound **5** (10.0 mg, 0.0265 mmol) was converted by using acrylonitrile **6d** instead of methyl acrylate to 5.6 mg (55%) of **7d**. Compound **7d** was obtained as a yellow oil. TLC R_f 0.76 (EtOAc/hexane, 1:2). IR (neat): 2926, 2237, 1454 cm^{-1}. ^{1}H NMR (400 MHz, $CDCl_3$): δ 8.46 (m, 1H), 7.56 (m, 1H), 7.33-7.24 (m, 4H), 7.23-7.14 (m, 7H), 7.08 (m, 1H), 4.96 (m, 1H), 2.74-2.58 (m, 4H), 2.13 (m, 1H), 1.98 (m, 1H), 1.90 (m, 1H), 1.84-1.69 (m, 4H). ^{13}C{^{1}H} NMR (100 MHz, $CDCl_3$): δ 154.7, 149.7, 142.1, 142.0, 136.6, 128.5 (2C), 128.4 (2C), 128.34 (2C), 128.32 (2C), 125.90, 125.87, 122.3, 120.7, 119.9, 36.5, 35.4, 35.3, 34.6, 32.8, 32.3, 29.3. HRMS (ESI-TOF) *m/z*: [M + Na]$^+$ Calcd for $C_{25}H_{26}N_2NaS$ 409.1714; Found 409.1702.

4-Phenethyl-6-phenyl-2-(pyridin-2-ylthio)hexanoic acid (7e)

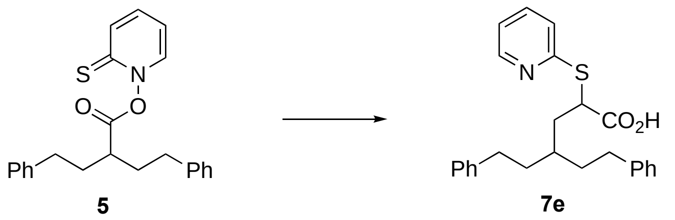

As described for the preparation of **7a**, compound **5** (100 mg, 0.265 mmol) was converted by using acrylic acid **6e** instead of methyl acrylate to 92.4 mg (86%) of **7e**. Compound **7e** was obtained as a yellow oil. TLC R_f 0.19 (EtOAc/hexane, 1:2). IR (neat): 2928, 1729, 1454 cm^{-1}. ^{1}H NMR (400 MHz, $CDCl_3$): δ 8.42 (m, 1H), 7.67 (m, 1H), 7.32 (m, 1H), 7.30-7.06 (m, 11H), 3.90 (m, 1H), 2.70-2.51 (m, 4H), 2.21 (m, 1H), 1.83 (m, 1H), 1.78-1.60 (m, 5H). ^{13}C{^{1}H} NMR (100 MHz, $CDCl_3$): δ 172.8, 158.1, 147.8, 142.34, 142.28, 138.0, 128.34 (4C), 128.28 (2C), 128.2 (2C), 125.8, 125.7, 124.1, 121.2, 45.8, 35.4, 35.2, 34.3, 33.9, 32.8, 32.4. HRMS (ESI-TOF) *m/z*: [M + Na]$^+$ Calcd for $C_{25}H_{27}NNaO_2S$ 428.1660; Found 428.1651.

Methyl 2-methyl-4-phenethyl-6-phenyl-2-(pyridin-2-ylthio)hexanoate (7g)

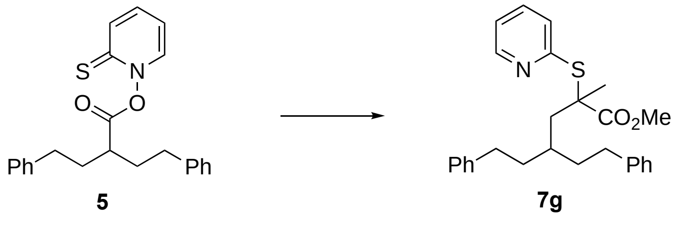

As described for the preparation of **7a**, compound **5** (10.8 mg, 0.0286mmol) was converted by using methyl methacrylate **6g** instead of methyl acrylate to 4.3 mg (35%) of **7g**. Compound **7g** was obtained as a yellow oil. TLC R_f 0.76 (EtOAc/hexane, 1:2). IR (neat): 2930, 1732 cm^{-1}. ^{1}H NMR (400 MHz, $CDCl_3$): δ 8.38 (m, 1H), 7.48 (m, 1H), 7.31-7.24 (m,

4H), 7.21-7.11 (m, 7H), 7.00 (m, 1H), 3.64 (s, 3H), 2.63-2.55 (m, 4H), 2.13 (m, 1H), 2.01 (m, 1H), 1.79 (m, 1H), 1.71-1.46 (m, 7H). $^{13}C\{^1H\}$ NMR (100 MHz, $CDCl_3$): δ 174.7, 149.3, 142.53, 142.51, 136.0, 128.3 (8C), 125.7 (2C), 123.8, 120.2, 55.0, 52.4, 42.5, 37.1, 36.7, 33.8, 32.9, 32.7, 24.1. HRMS (ESI-TOF) *m/z*: $[M + Na]^+$ Calcd for $C_{27}H_{31}NNaO_2S$ 456.1973; Found 456.1960.

Dimethyl 2-(1,5-diphenylpentan-3-yl)-3-(pyridin-2-ylthio)succinate (6i)

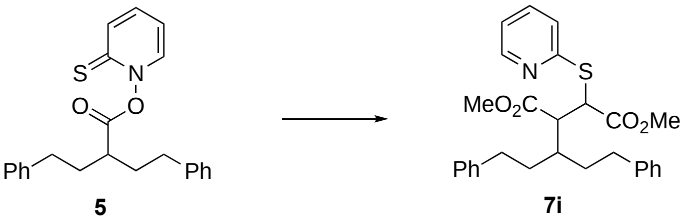

As described for the preparation of **7a**, compound **5** (9.5 mg, 0.0252mmol) was converted by using dimethyl fumarate **6i** instead of to methyl acrylate to 6.0 mg (50%, dr 2:1) of **7i**. Compound **7i** was obtained as a yellow oil. TLC R_f 0.55 (EtOAc/hexane, 1:2). IR (neat): 2949, 1733 cm^{-1}. ^1H NMR (400 MHz, $CDCl_3$): δ 8.45 (m, 2/3H), 8.42 (m, 1/3H), 7.51 (m, 1H), 7.30-7.09 (m, 10H), 7.07-6.99 (m, 2H), 5.28 (d, 1/3H, J = 8.0 Hz), 5.19 (d, 2/3H, J = 7.6 Hz), 3.704 (s, 3×2/3H), 3.698 (s, 3×2/3H), 3.67 (s, 3×1/3H), 3.61 (s, 3×1/3H), 3.34 (m, 1H), 2.89 (m, 2/3H), 2.76-2.45 (m, 3H), 2.19 (m, 2/3H), 2.08 (m, 2/3H), 2.01-1.67 (m, 10/3H), 1.56 (m, 2/3H). $^{13}C\{^1H\}$ NMR (100 MHz, $CDCl_3$) for major isomer: δ 173.1, 172.8, 155.9, 149.5, 142.4, 142.1, 136.4, 128.5 (2C), 128.4 (2C), 128.3 (4C), 125.8, 125.7, 122.5, 120.4, 52.7, 51.7, 48.2, 44.8, 36.9, 34.0, 33.6, 33.4, 32.7. HRMS (ESI-TOF) *m/z*: $[M + Na]^+$ Calcd for $C_{28}H_{31}NNaO_4S$ 500.1871; Found 500.1861.

1-ethyl-4-(hexyloxy)benzene (9).

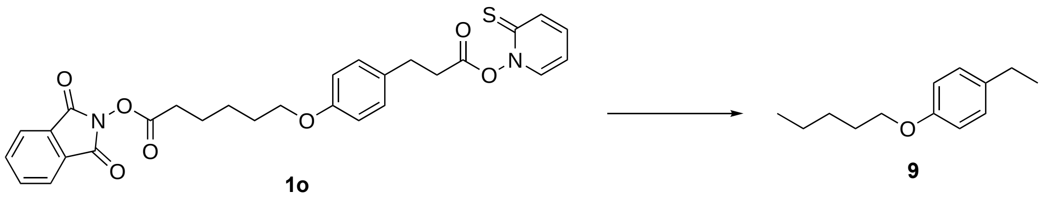

The following reaction was carried out under Ar. To a stirred solution of **1o** (19.6 mg, 0.0367 mmol) and zinc(II) tetraphenylporphyrin (0.03 mg, 0.04 μmol) in DMA (0.4 mL) was added *tert*-butylthiol (16.9 μL, 0.150 mmol). The stirred mixture was irradiated by red LEDs at 25 °C for 1 h, and BNAH (8.2 mg, 0.038 mmol) and $Ru(bpy)_3Cl_2{\cdot}H_2O$ (1.4 mg, 1.9 μmol) were added. After being stirred at 25 °C and irradiated by blue LEDs for 6 h, the mixture was diluted with EtOAc/hexane (1:3, 10 mL) and washed with H_2O (10 mL×3) and saturated brine (10 mL), sequentially. The organic layer was dried and concentrated under reduced pressure.

The residue was purified by PTLC (EtOAc/hexane, 1:8) to provide 5.2 mg (73%) of **9**[S3] as a colorless oil.

1-[2-(4-Methoxyphenylthio)ethyl]-4-pentyloxybenzene (11).

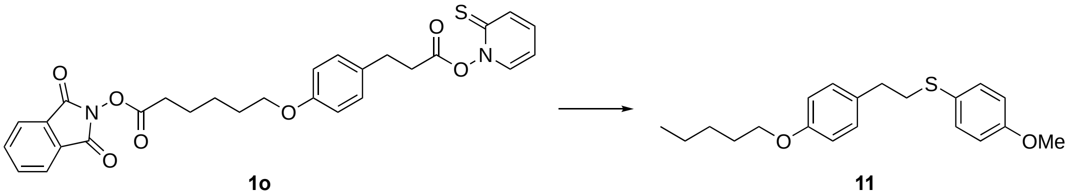

The following reaction was carried out under Ar. To a stirred solution of **1o** (14.5 mg, 0.0271 mmol) and zinc(II) tetraphenylporphyrin (0.6 mg, 0.9 µmol) in DMA (0.6 mL) was added bis(4-methoxyphenyl) disulfide (15.2 mg, 0.0546 mmol). The stirred mixture was irradiated by red LEDs at 25 °C for 1 h, and BNAH (6.3 mg, 0.029 mmol), *tert*-butylthiol (13.0 µL, 0.115 mmol) and Ru(bpy)$_3$Cl$_2$·H$_2$O (0.7 mg, 0.9 µmol) were added. After being stirred at 25 °C and irradiated by blue LEDs for 4 h, the mixture was diluted with EtOAc (20 mL) and washed with H_2O (10 mL×3) and saturated brine (10 mL), sequentially. The organic layer was dried and concentrated under reduced pressure. The residue was purified by PTLC (EtOAc/hexane, 1:12) to provide 6.1 mg (68%) of **11** as a colorless oil. TLC R_f 0.70 (EtOAc/hexane, 1:12). IR (neat): 2930 cm^{-1}. ^{1}H NMR (400 MHz, CDCl$_3$): δ 7.36 (d, 2H, *J* =7.8 Hz, H-2, 6 of 4-methoxyphenyl), 7.06 (d, 2H, *J* =8.2 Hz, H-2, 6 of Ar), 6.86 (d, 2H, *J* =7.8 Hz, H-3, 5 of 4-methoxyphenyl), 6.81 (d, 2H, *J* =8.2 Hz, H-3, 5 of Ar), 3.92 (t, 2H, *J* =6.7 Hz, H-1, 1' of pentyloxy), 3.81 (s, 3H, -CH$_3$ of 4-methoxyphenyl), 3.03 (t, 2H, *J* =7.7 Hz, H-2, 2' of 2-(4-methoxyphenylthio)ethyl), 2.80 (t, 2H, *J* =7.7 Hz, H-1, 1' of 2-(4-methoxyphenylthio)ethyl), 1.77 (quin, 2H, *J* =6.7 Hz, H-2, 2' of pentyloxy), 1.47-1.33 (m, 4H, H-3, 3', 4, 4' of pentyloxy), 0.92 (t, 3H, *J* =6.8 Hz, -CH$_3$ of pentyloxy). ^{13}C{^{1}H} NMR (125 MHz, CDCl$_3$): δ 158.9, 157.7, 133.2 (2C), 132.2, 129.4 (2C), 126.4, 114.6 (2C), 114.2 (2C), 68.0, 55.3, 37.5, 35.0, 29.0, 28.2, 22.5, 14.0. HRMS (ESI-TOF) *m/z*: [M + H]$^+$ Calcd for $C_{20}H_{27}O_2S$ 331.1732; Found 331.1731.

1-[4-(Methoxycarbonyl)-4-(pyridinylthio)butyl]-4-pentyloxybenzene (13).

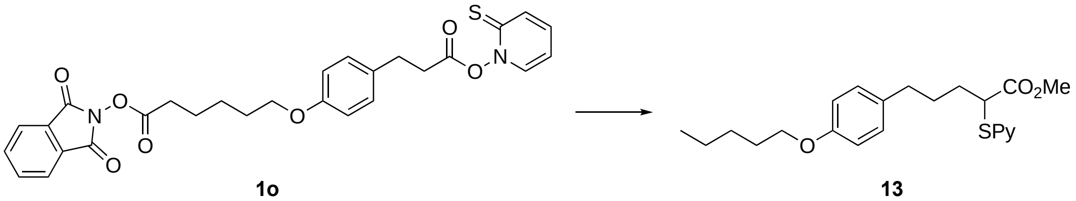

The following reaction was carried out under Ar. To a stirred solution of **1o** (14.3 mg, 0.0267 mmol) and zinc(II) tetraphenylporphyrin (0.5 mg, 0.7 µmol) in DMA (0.6 mL) was added methyl acrylate (3.8 µL, 0.042 mmol). The stirred mixture was irradiated by red LEDs at 25 °C for 1 h, and BNAH (6.0 mg, 0.028 mmol), *tert*-butylthiol (13.0 µL, 0.115 mmol) and

[S3] Li, F.; Zhang, G.; Liu, Y.; Zhu, B.; Leng, Y.; Wu, J. *Org. Lett.* **2020**, *22*, 8791-8795.

Ru(bpy)$_3$Cl$_2$·H$_2$O (0.6 mg, 0.8 μmol) were added. After being stirred at 25 °C and irradiated by blue LEDs for 4 h, the mixture was diluted with EtOAc (20 mL) and washed with H_2O (10 mL×3) and saturated brine (10 mL), sequentially. The organic layer was dried and concentrated under reduced pressure. The residue was purified by PTLC (EtOAc/hexane, 1:6) to provide 7.4 mg (72%) of **13** as a colorless oil. TLC R_f 0.69 (EtOAc/hexane, 1:3). IR (neat): 2952, 1735 cm^{-1}. ^{1}H NMR (400 MHz, CDCl$_3$): δ 8.40 (m, 1H, H-6 of pyridinyl), 7.48 (td, 1H, *J* =7.6, 1.8 Hz, H-4 of pyridinyl), 7.18 (d, 1H, *J* =7.6 Hz, H-3 of pyridinyl), 7.06 (d, 2H, *J* =8.8 Hz, H-2, 6 of Ar), 6.99 (dd, 1H, *J* =7.6, 4.9 Hz, H-5 of pyridinyl), 6.80 (d, 2H, *J* =8.8 Hz, H-3, 5 of Ar), 4.62 (t, 1H, *J* =7.5 Hz, H-4 of butyl), 3.92 (t, 2H, *J* =6.5 Hz, H-1, 1' of pentyloxy), 3.71 (s, 3H, CO$_2$Me), 2.59 (t, 2H, *J* =7.8 Hz, H-1, 1' of butyl), 2.06-1.86 (m, 2H, H-3, 3' of butyl), 1.84-1.70 (m, 4H, H-2, 2' of butyl, H-2, 2' of pentyloxy), 1.46-1.33 (m, 4H, H-3, 3', 4, 4' of pentyloxy), 0.93 (t, 3H, *J* =7.3 Hz, -CH$_3$ of pentyloxy). ^{13}C{^{1}H} NMR (125 MHz, CDCl$_3$): δ 173.2, 157.3 (2C), 149.4, 136.1, 133.6, 129.2 (2C), 122.2, 119.9, 114.3 (2C), 67.9, 52.4, 46.1, 34.4, 31.4, 29.1, 29.0, 28.2, 22.5, 14.0. HRMS (ESI-TOF) *m/z*: [M + H]$^+$ Calcd for $C_{22}H_{30}NO_3S$ 388.1946; Found 388.1934.

1-[4-(Methoxycarbonyl)-4-(pyridinylthio)butyl]-4-[5-(phenylthio)pentyloxy]benzene (14).

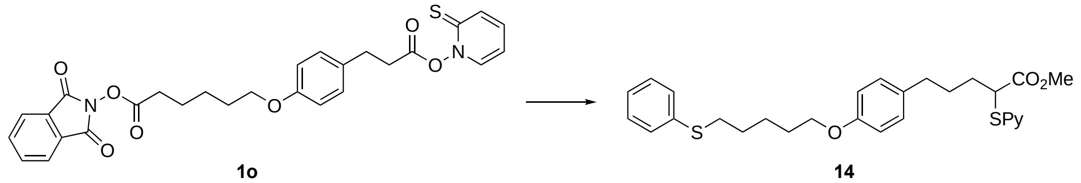

1o 14

The following reaction was carried out under Ar. To a stirred solution of **1o** (14.6 mg, 0.0273 mmol) and zinc(II) tetraphenylporphyrin (0.6 mg, 0.9 μmol) in DMA (0.6 mL) was added methyl acrylate (3.8 μL, 0.042 mmol). The stirred mixture was irradiated by red LEDs at 25 °C for 1 h, and diphenyl disulfide (24.2 mg, 0.0281 mmol), DIPEA (10.0 μL, 0.0574 mmol) and Ru(bpy)$_3$Cl$_2$·H$_2$O (0.7 mg, 0.9 μmol) were added. After being stirred at 25 °C and irradiated by blue LEDs for 4 h, the mixture was diluted with EtOAc (20 mL) and washed with H_2O (10 mL×3) and saturated brine (10 mL), sequentially. The organic layer was dried and concentrated under reduced pressure. The residue was purified by PTLC (EtOAc/hexane, 1:3) to provide 7.2 mg (53%) of **14** as a colorless oil. TLC R_f 0.69 (EtOAc/hexane, 1:3). IR (neat): 2937, 1736 cm^{-1}. ^{1}H NMR (400 MHz, CDCl$_3$): δ 8.40 (m, 1H, H-6 of pyridinyl), 7.48 (td, 1H, *J* =7.5, 1.7 Hz, H-4 of pyridinyl), 7.33 (d, 2H, *J* =8.1 Hz, H-2, 6 of phenyl), 7.27 (t, 2H, *J* =8.1 Hz, H-3, 5 of phenyl), 7.20-7.14 (m, 2H, H-4 of phenyl, H-3 of pyridinyl), 7.06 (d, 2H, *J* =8.8 Hz, H-3, 5 of Ar), 6.99 (dd, 1H, *J* =7.5, 5.0 Hz, H-5 of pyridinyl), 6.78 (d, 2H, *J* =8.8 Hz, H-2, 6 of Ar), 4.62 (t, 1H, *J* =7.5 Hz, H-4 of butyl), 3.91 (t, 2H, *J* =6.5 Hz, H-1, 1' of pentyloxy), 3.71 (s, 3H, CO$_2$Me), 2.95 (t, 2H, *J* =7.3 Hz, H-5, 5' of pentyloxy), 2.59 (t, 2H, *J* =7.5 Hz, H-1, 1' of butyl), 2.07-1.86 (m, 2H, H-3, 3' of butyl), 1.84-1.68 (m, 6H, H-2, 2' of butyl, H-2, 2', 4, 4' of pentyloxy), 1.64-1.57 (m, 2H, H-3, 3' of pentyloxy). ^{13}C{^{1}H} NMR (125 MHz, CDCl$_3$): δ 173.2, 157.2 (2C), 149.4, 136.7, 136.1, 133.7, 129.2 (2C), 129.0 (2C), 128.8 (2C), 125.8, 122.2, 119.9, 114.3 (2C), 67.6, 52.4, 46.1, 34.4, 33.5, 31.4, 29.1, 28.9 (2C),

25.3. HRMS (ESI-TOF) *m/z*: [M + H]$^+$ Calcd for $C_{28}H_{34}NO_3S_2$ 496.1980; Found 496.1980.

Benzyl 8-(4-(5-methoxy-5-oxo-4-(pyridin-2-ylthio)pentyl)phenoxy)octanoate (15).

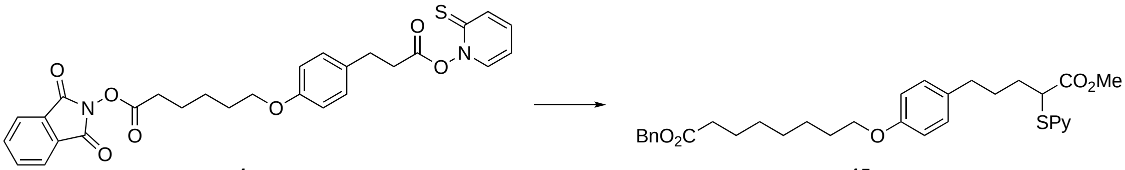

The following reaction was carried out under Ar. To a stirred solution of **1o** (14.7 mg, 0.0275 mmol) and zinc(II) tetraphenylporphyrin (0.6 mg, 0.9 μmol) in DMA (0.6 mL) was added methyl acrylate (3.8 μL, 0.042 mmol). The stirred mixture was irradiated by red LEDs at 25 °C for 1 h, and benzyl acrylate (17 μL, 0.11 mmol), BNAH (12.1 mg, 0.0564 mmol) and $Ru(bpy)_3Cl_2·H_2O$ (0.5 mg, 0.7 μmol) were added. After being stirred at 25 ℃ and irradiated by blue LEDs for 4 h, the mixture was diluted with EtOAc (20 mL) and washed with H_2O (10 mL×3) and saturated brine (10 mL), sequentially. The organic layer was dried and concentrated under reduced pressure. The residue was purified by PTLC (EtOAc/hexane, 1:3) to provide 7.6 mg (50%) of **15** as a yellow oil. TLC R_f 0.70 (EtOAc/hexane, 1:3). IR (neat): 2934, 1736 cm^{-1}. ^{1}H NMR (400 MHz, $CDCl_3$): δ 8.40 (m, 1H, H-6 of pyridinyl), 7.48 (t, 1H, *J* = 7.8 Hz, H-4 of pyridinyl), 7.40-7.31 (m, 5H , -Ph), 7.18 (d, 1H, *J* = 7.8 Hz, H-3 of pyridinyl), 7.06 (d, 2H, *J* = 8.6 Hz, H-3, 5 of Ar), 6.99 (dd, 1H, *J* = 7.8, 4.9 Hz, H-5 of pyridinyl), 6.79 (d, 2H, *J* = 8.6 Hz, H-2, 6 of Ar), 5.11 (s, 2H, Bn), 4.62 (t, 1H, *J* = 7.2 Hz, H-4 of butyl), 3.90 (t, 2H, *J* = 6.6 Hz, H-1, 1' of heptyloxy), 3.71 (s, 3H, CO_2Me), 2.59 (t, 2H, *J* = 7.6 Hz, H-1, 1' of butyl), 2.36 (t, 2H, *J* = 7.4 Hz, H-7,7' of heptyloxy), 2.08-1.84 (m, 2H, H-3, 3' of butyl), 1.83-1.60 (m, 6H, H-2, 2' of butyl, H-2, 2', 6, 6' of heptyloxy), 1.48-1.31 (m, 6H, H-3, 3', 4, 4', 5, 5' of heptyloxy). $^{13}C\{^1H\}$ NMR (100 MHz, $CDCl_3$): δ 173.6, 173.2, 157.3 (2C), 149.4, 136.1 (2C), 133.6, 129.2 (2C), 128.5 (2C), 128.2 (3C), 122.2, 119.9, 114.3 (2C), 67.8, 66.1, 52.4, 46.1, 34.4, 34.3, 31.4, 29.2, 29.1, 29.01, 28.99, 25.9, 24.9. HRMS (ESI-TOF) *m/z*: [M + H]$^+$ Calcd for $C_{32}H_{40}NO_5S$ 550.2627; Found 550.2631.

1-Pentyloxy-4-[2-(phenylthio)ethyl]benzene (S10).

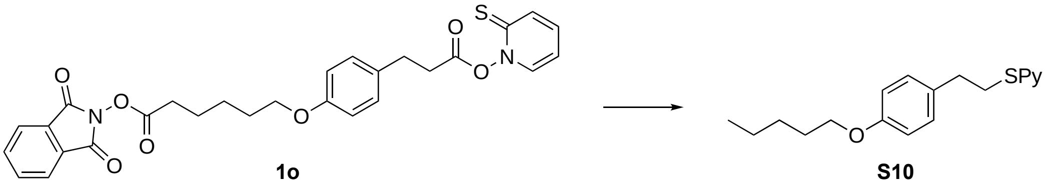

The following reaction was carried out under Ar. To a stirred solution of **1o** (14.7 mg, 0.0275 mmol) and zinc(II) tetraphenylporphyrin (0.5 mg, 0.7 μmol) and MS 4Å powder (26.2 mg) in DMA (0.6 mL) was added 2,2'-dipyridyl disulfide (13.5 mg, 0.0613 mmol). The stirred mixture was irradiated by red LEDs at 25 °C for 1 h, and BNAH (6.1 mg, 0.029 mmol), *tert*-butylthiol (12.7 μL, 0.112 mmol) and $Ru(bpy)_3Cl_2·H_2O$ (0.7 mg, 0.9 μmol) were added. After being stirred at 25 ℃ and irradiated by blue LEDs for 4 h, the mixture was diluted with EtOAc (20 mL) and washed with H_2O (5 mL×3) and saturated brine (10 mL), sequentially. The organic layer was dried and concentrated under reduced pressure. The residue was purified by PTLC (EtOAc/hexane, 1:8) to provide 4.6 mg (55%) of **S10** as a colorless oil. TLC R_f 0.71 (EtOAc/hexane, 1:4). IR (neat): 2927 cm^{-1}. ^{1}H NMR (400 MHz, $CDCl_3$): δ 8.45 (m, 1H, H-6 of pyridinyl),

7.47 (m, 1H, H-4 of pyridinyl), 7.17 (m, 3H, H-3, 5 of Ar, H-3 of pyridinyl), 6.97 (m, 1H, H-5 of pyridinyl), 6.84 (d, 2H, *J* =8.4 Hz, H-2, 6 of Ar), 3.93 (t, 2H, *J* =6.7 Hz, H-1, 1' of pentyloxy), 3.38 (t, 2H, *J* =7.8 Hz, H-2, 2' of 2-(pyridinylthio)ethyl), 2.94 (t, 2H, *J* =7.8 Hz, H-1, 1' of 2-(pyridinylthio)ethyl), 1.78 (quin, 2H, *J* =6.7 Hz, H-2, 2' of pentyloxy), 1.48-1.33 (m, 4H, H-3, 3', 4, 4' of pentyloxy), 0.93 (t, 3H, *J* =7.0 Hz, -CH_3 of pentyloxy). $^{13}C\{^1H\}$ NMR (100 MHz, $CDCl_3$): δ 159.0, 157.7, 149.5, 135.8, 132.4, 129.5 (2C), 122.3, 119.3, 114.4 (2C), 68.0, 34.9, 31.7, 29.0, 28.2, 22.5, 14.0. HRMS (ESI-TOF) *m/z*: $[M + H]^+$ Calcd for $C_{18}H_{24}NOS$ 302.1579; Found 302.1574.

1-[5-Oxo-4-(pyridinylthio)hexyl]-4-pentyloxybenzene (S11).

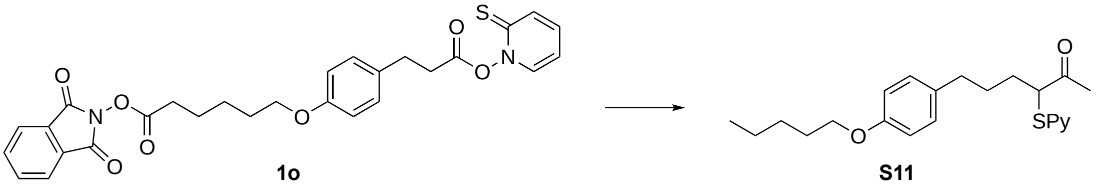

As described for the preparation of **13**, compound **1o** (14.3 mg, 0.0267 mmol) was converted using methyl vinyl ketone instead of methyl acrylate to 6.7 mg (67%) of **S11**. Compound **S11** was obtained as a colorless oil. TLC R_f 0.62 (EtOAc/hexane, 1:3). IR (neat): 2930, 1711 cm^{-1}. ^1H NMR (400 MHz, $CDCl_3$): δ 8.38 (m, 1H, H-6 of pyridinyl), 7.49 (t, 1H, *J* =7.7 Hz, H-4 of pyridinyl), 7.19 (d, 1H, *J* =7.7 Hz, H-3 of pyridinyl), 7.05 (d, 2H, *J* =8.4 Hz, H-2, 6 of Ar), 7.00 (dd, 1H, *J* =7.7, 4.9 Hz, H-5 of pyridinyl), 6.80 (d, 2H, *J* =8.4 Hz, H-3, 5 of Ar), 4.64 (t, 1H, *J* =7.0 Hz, H-4 of hexyl), 3.92 (t, 2H, *J* =6.6 Hz, H-1, 1' of pentyloxy), 2.65-2.53 (m, 2H, H-1, 1' of hexyl), 2.27 (s, 3H, -CH_3 of hexyl), 2.02-1.92 (m, 2H, H-3 of hexyl), 1.85-1.65 (m, 5H, H-2, 2', 3' of hexyl, H-2, 2' of pentyloxy), 1.48-1.33 (m, 4H, H-3, 3', 4, 4' of pentyloxy), 0.92 (t, 3H, *J* =7.0 Hz, -CH_3 of pentyloxy). $^{13}C\{^1H\}$ NMR (125 MHz, $CDCl_3$): δ 206.6, 157.3, 157.2, 149.3, 136.2, 133.6, 129.2 (2C), 122.3, 119.9, 114.4 (2C), 68.0, 52.4, 34.6, 29.6, 29.1, 29.0, 28.2, 22.5, 14.0. HRMS (ESI-TOF) *m/z*: $[M + H]^+$ Calcd for $C_{22}H_{30}NO_2S$ 372.1997; Found 372.1998.

1-[3,4-Bis(methoxycarbonyl)-4-(pyridinylthio)butyl]-4-pentyloxybenzene (S12).

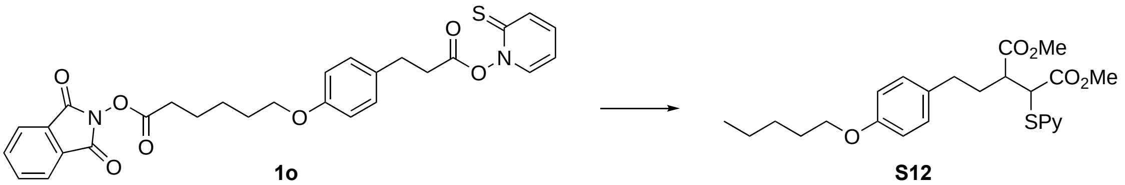

As described for the preparation of **13**, compound **1o** (14.3 mg, 0.0269 mmol) was converted using dimethyl fumarate instead of methyl acrylate to 7.9 mg (66%, dr = 2:1) of **S12**. Compound **S12** was obtained as a colorless oil. TLC R_f 0.47 (EtOAc/hexane, 1:3). ^1H NMR (400 MHz, $CDCl_3$): δ 8.42 (m, 1H, H-6 of pyridinyl), 7.50 (t, 1H, *J* =7.7 Hz, H-4 of pyridinyl), 7.21 (m, 1H, H-3 of pyridinyl), 7.04-6.97 (m, 3H, H-2, 6 of Ar, H-5 of pyridinyl), 6.77 (m, 2H, H-3, 5 of Ar), 5.26 (d, 1H×2/3, *J* =6.4 Hz, H-4 of butyl×2/3), 5.11 (d, 1H×1/3, *J* =9.6 Hz, H-4 of butyl×1/3), 3.94-3.86 (m, 2H, H-1, 1' of pentyloxy), 3.71 (s, 3H×1/3, CO_2Me×1/3), 3.70 (s, 3H×2/3, CO_2Me×2/3), 3.69 (s, 3H×1/3, CO_2Me×1/3), 3.69 (s, 3H×2/3, CO_2Me×2/3), 3.20 (m, 1H×2/3, H-3 of butyl×2/3), 3.10 (m, 1H×1/3, H-3 of butyl×1/3), 2.71-2.43 (m, 2H, H-1, 1' of butyl), 2.18-2.07 (m, 2H×2/3, H-2, 2' of butyl×2/3), 1.96-1.86 (m, 2H×1/3, H-2, 2' of butyl×1/3), 1.81-1.71 (m, 2H, H-2, 2' of pentyloxy), 1.48-1.32 (m, 4H, H-3, 3', 4, 4' of pentyloxy), 0.92 (t, 3H, *J* =7.0

Hz, -CH_3 of pentyloxy). $^{13}C\{^1H\}$ NMR (100 MHz, $CDCl_3$), major isomer: δ 173.6, 171.8, 157.5, 156.6, 149.3, 136.2, 132.8, 129.3 (2C), 122.3, 120.1, 114.4 (2C), 68.0, 52.8, 52.0, 46.38, 46.36, 32.6, 31.9, 29.0, 28.2, 22.5, 14.0. minor isomer: δ 174.0, 172.1, 157.4, 156.0, 149.4, 136.3, 133.0, 129.3 (2C), 122.5, 120.3, 114.3 (2C), 68.0, 52.7, 52.0, 46.5, 46.2, 31.9, 31.3, 29.0, 28.2, 22.5, 14.0. HRMS (ESI-TOF) *m/z*: $[M + H]^+$ Calcd for $C_{24}H_{32}NO_5S$ 446.2001; Found 446.1995.

1-Pentyloxy-4-[4-(phenylsulfonyl)-4-(pyridinylthio)butyl]benzene (S13).

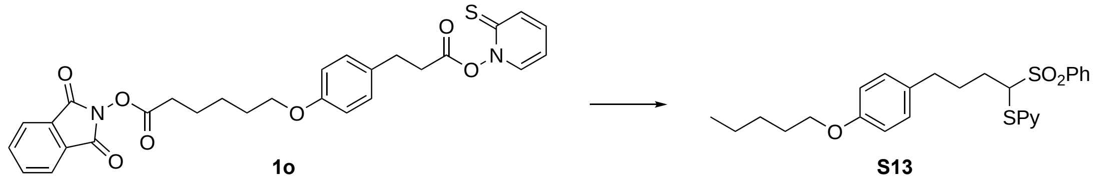

1o **S13**

As described for the preparation of **13**, compound **1o** (15.0 mg, 0.0281 mmol) was converted using phenyl vinyl sulfone instead of methyl acrylate to 6.8 mg (52%) of **S13**. Compound **S13** was obtained as a colorless oil. TLC R_f 0.41 (EtOAc/hexane, 1:3). IR (neat): 2926 cm^{-1}. ^1H NMR (400 MHz, $CDCl_3$): δ 8.20 (m, 1H, H-6 of pyridinyl), 7.87 (d, 2H, *J* =6.4 Hz, H-2, 6 of -SO_2Ph), 7.42 (t, 1H, *J* =7.6 Hz, H-4 of pyridinyl), 7.37-7.28 (m, 3H, H-3, 4, 5 of -SO_2Ph), 7.02 (d, 2H, *J* =8.4 Hz, H-3, 5 of Ar), 6.96 (d, 1H, *J* =7.6 Hz, H-3 of pyridinyl), 6.90 (dd, 1H, *J* =7.6, 5.0 Hz, H-5 of pyridinyl), 6.77 (d, 2H, *J* =8.4 Hz, H-2, 6 of Ar), 5.75 (dd, 1H, *J* =6.8, 3.2 Hz, H-4 of butyl), 3.91 (t, 2H, *J* = 6.4 Hz, H-1, 1' of pentyloxy), 2.69-2.51 (m, 2H, H-1, 1' of butyl), 2.43 (m, 1H, H-3 of butyl), 2.04-1.72 (m, 5H, H-2, 2', 3' of butyl, H-2, 2' of pentyloxy), 1.48-1.32 (m, 4H, H-3, 3', 4, 4' of pentyloxy), 0.92 (t, 3H, *J* =7.0 Hz, -CH_3 of pentyloxy). $^{13}C\{^1H\}$ NMR (100 MHz, $CDCl_3$): δ 157.4, 154.7, 149.0, 137.1, 136.2, 133.4, 133.3, 129.6 (2C), 129.2 (2C), 128.2 (2C), 122.4, 120.4, 114.4 (2C), 68.0, 65.3, 34.2, 29.0, 28.5, 28.2, 26.6, 22.5, 14.0. HRMS (ESI-TOF) *m/z*: $[M + H]^+$ Calcd for $C_{26}H_{32}NO_3S_2$ 470.1824; Found 470.1816. HRMS (ESI-TOF) *m/z*: $[M + H]^+$ Calcd for $C_{26}H_{32}NO_3S_2$ 470.1824; Found 470.1816.

1-[6,7-Bis(methoxycarbonyl)heptyloxy]-4-[4-(methoxycarbonyl)-4-(pyridinylthio)butyl]benzene (S14).

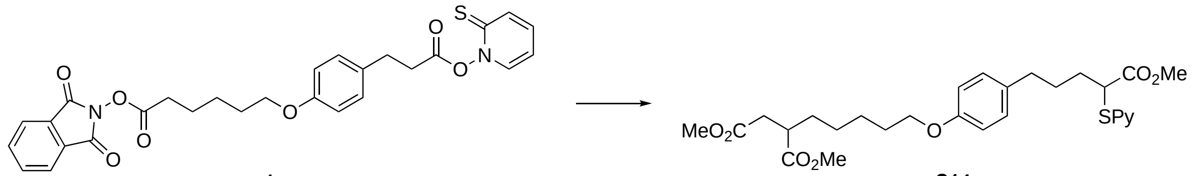

1o **S14**

As described for the preparation of **15**, compound **1o** (14.6 mg, 0.0273 mmol) was converted using dimethyl fumarate instead of benzyl acrylate to 8.7 mg (60%) of **S14**. Compound **S14** was obtained as yellow solids. TLC R_f 0.42 (EtOAc/hexane, 1:2). IR (neat): 1732, 2950 cm^{-1}. ^1H NMR (400 MHz, $CDCl_3$): δ 8.40 (m, 1H, H-6 of pyridinyl), 7.48 (t, 1H, *J* =7.7 Hz, H-4 of pyridinyl), 7.18 (d, 1H, *J* =7.7 Hz, H-3 of pyridinyl), 7.06 (d, 2H, *J* =8.6 Hz, H-3, 5 of Ar), 6.99 (dd, 1H, *J* =7.7, 5.1 Hz, H-5 of pyridinyl), 6.78 (d, 2H, *J* =8.6 Hz, H-2, 6 of Ar), 4.62 (t, 1H, *J* =7.4 Hz, H-4 of butyl), 3.90 (t, 2H, *J* =6.4 Hz, H-1, 1' of heptyloxy), 3.71 (s, 3H, CO_2Me), 3.69 (s, 3H, CO_2Me), 3.67 (s, 3H, CO_2Me), 2.86 (m, 1H, H-6 of heptyloxy), 2.73 (dd, 1H, *J* =16.7, 9.4 Hz, H-7 of heptyloxy), 2.59 (t, 2H, *J* =7.6 Hz, H-1, 1' of butyl), 2.44 (dd, 1H, *J* =16.7, 5.2 Hz, H-7' of heptyloxy), 2.08-1.85 (m, 2H, H-3, 3' of butyl), 1.83-1.72 (m, 4H, H-2, 2' of butyl, H-2, 2' of heptyloxy), 1.66 (m, 1H, H-5 of heptyloxy), 1.55 (m, 1H, H-5' of heptyloxy), 1.51-1.31

(m, 4H, H-3, 3', 4, 4' of heptyloxy). $^{13}C\{^1H\}$ NMR (100 MHz, $CDCl_3$): δ 175.4, 173.2, 172.4, 157.3, 157.2, 149.4, 136.1, 133.7, 129.2 (2C), 122.3, 119.9, 114.3 (2C), 67.7, 52.4, 51.84, 51.78, 46.1, 41.1, 35.8, 34.4, 31.8, 31.4, 29.1 (2C), 26.7, 25.9. HRMS (ESI-TOF) *m/z*: $[M + H]^+$ Calcd for $C_{27}H_{36}NO_7S$ 518.2212; Found 518.2214.

2-Thioxopyridin-1(2*H*)-yl 2-methylbutanoate (S60)

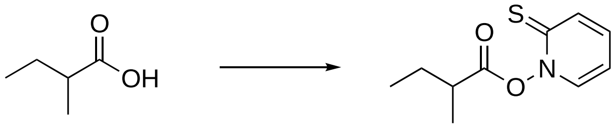

S59 **S60**

As described for the preparation of **1b**, compound **S59** (200 mg, 1.96 mmol) was converted to 413 mg (quant.) of **S60**.[S4] Compound **S60** was obtained as a yellow oil. TLC R_f 0.32 (EtOAc/hexane, 1:2). ^1H NMR (400 MHz, $CDCl_3$): δ 7.69 (m, 1H, 2-pyridinethione), 7.51 (m, 1H, 2-pyridinethione), 7.20 (m, 1H, 2-pyridinethione), 6.62 (m, 1H, 2-pyridinethione), 2.81 (m, 1H), 1.95 (m, 1H), 1.70 (m, 1H), 1.40 (d, 3H, J = 7.6 Hz), 1.05 (t, 3H, J = 7.2 Hz).

2-Thioxopyridin-1(2*H*)-yl 2-methyl-3-phenylpropanoate (S62)

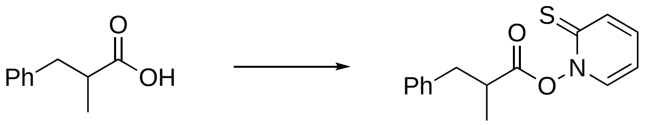

S61 **S62**

As described for the preparation of **1b**, compound **S61** (50.1 mg, 0.305 mmol) was converted to 58.5 mg (70%) of **S62**. Compound **S62** was obtained as a yellow oil. TLC R_f 0.35 (EtOAc/hexane, 1:2). IR (neat): 1801, 1527, 1448 cm^{-1}. ^1H NMR (400 MHz, $CDCl_3$): δ 7.65 (m, 1H, 2-pyridinethione), 7.37-7.22 (m, 4H, 2-pyridinethione, phenyl), 7.16 (m, 1H, 2-pyridinethione), 7.07 (d, 1H, phenyl, J = 6.8 Hz), 6.54 (m, 1H, 2-pyridinethione), 3.23-3.13 (m, 2H), 2.92 (m, 1H), 1.43 (m, 3H). $^{13}C\{^1H\}$ NMR (100 MHz, $CDCl_3$): δ 175.8, 171.3, 138.2, 137.4, 137.3, 133.4, 129.1 (2C), 128.5 (2C), 126.8, 112.5, 39.7, 39.6, 16.7. HRMS (ESI-TOF) *m/z*: $[M + Na]^+$ Calcd for $C_{15}H_{15}NNaO_2S$ 296.0721; Found 296.0715.

2-Thioxopyridin-1(2*H*)-yl cyclohexanecarboxylate (S64)

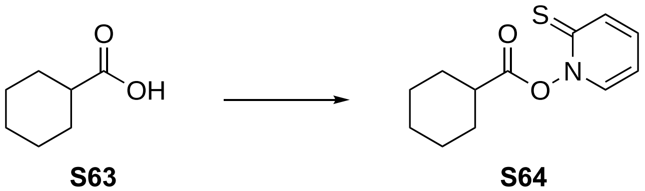

S63 **S64**

As described for the preparation of **1b**, compound **S63** (200 mg, 1.56 mmol) was converted to 368 mg (quant.) of **S64**.[S5] Compound **S64** was obtained as a yellow oil. TLC R_f 0.41(EtOAc/hexane, 1:2). ^1H NMR (400 MHz, $CDCl_3$): δ 7.69 (m, 1H, 2-pyridinethione), 7.52 (m, 1H, 2-pyridinethione), 7.19 (m, 1H, 2-

[S4] D. H. R. Barton, N. Ozbalik, B. Vacher *Tetrahedron* **1988**, *44*, 3501-3512.

[S5] D. H. R. Barton, D. Crich, G. Kretzschmar *Tetrahedron Lett.* **1984**, *25*, 1055-1058.

pyridinethione), 6.62 (m, 1H, 2-pyridinethione), 2.76 (tt, 1H, J = 10.8, 3.6 Hz), 2.22-2.14 (m, 2H), 1.89-1.80 (m, 2H), 1.73-1.61 (m, 3H), 1.43-1.24 (m, 3H).

2-Thioxopyridin-1(2*H*)-yl cyclobutanecarboxylate (S66)

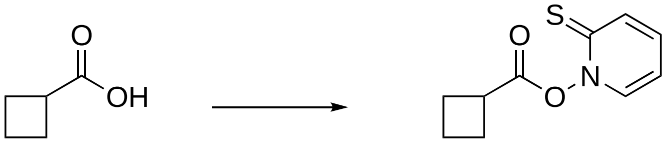

S65 **S66**

As described for the preparation of **1b**, compound **S65** (200 mg, 2.00 mmol) was converted to 418 mg (quant.) of **S66**.[S6] Compound **S66** was obtained as a yellow oil. TLC R_f 0.25 (EtOAc/hexane, 1:2). ^{1}H NMR (400 MHz, $CDCl_3$): δ 7.70 (m, 1H, 2-pyridinethione), 7.55 (m, 1H, 2-pyridinethione), 7.21(m, 1H, 2-pyridinethione), 6.63 (m, 1H, 2-pyridinethione), 3.52 (m, 1H,), 2.68-2.57 (m, 2H), 2.45-2.35 (m, 2H), 2.14-2.01 (m, 2H).

1-(*tert*-Butyl) 4-[2-thioxopyridin-1(2*H*)-yl] piperidine-1,4-dicarboxylate (S68)

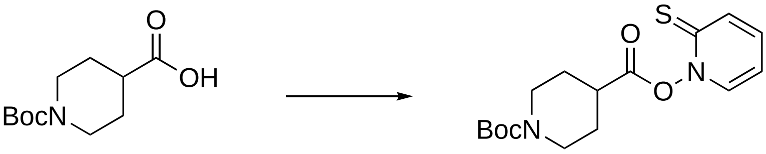

S67 **S68**

As described for the preparation of **S68**, compound **S67** (100 mg, 0.436 mmol) was converted to 122 mg (83%) of **S68**. Compound **S68** was obtained as a yellow oil. TLC *Rf* 0.25 (EtOAc/hexane, 4:1). IR (neat): 2977, 1684 cm^{-1}. ^{1}H NMR (400 MHz, $CDCl_3$): δ 7.65 (m, 1H, 2-pyridinethione), 7.54 (m, 1H, 2-pyridinethione), 7.19 (m, 1H, 2-pyridinethione), 6.62 (m, 1H, 2-pyridinethione), 4.07 (br, 2H), 2.99-2.86 (m, 3H), 2.14-2.06 (m, 2H), 1.90-1.78 (m, 2H), 1.44 (s, 9H). ^{13}C{^{1}H} NMR (100 MHz, $CDCl_3$): δ 175.7, 169.9, 154.5, 137.5, 137.4, 133.5, 112.6, 79.8, 42.5 (2C, br), 39.2, 28.3 (3C), 27.7 (2C). HRMS (ESI-TOF) *m/z*: [M + Na]$^+$ Calcd for $C_{16}H_{22}N_2NaO_4S$ 361.1198; Found 361.1191.

1-Benzyl 4-[2-thioxopyridin-1(2*H*)-yl] piperidine-1,4-dicarboxylate (S70)

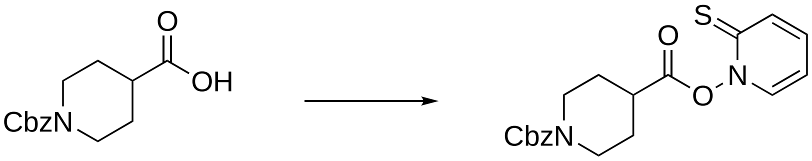

S69 **S70**

As described for the preparation of **1b**, compound **S69** (100 mg, 0.380 mmol) was converted to 117 mg (83%) of **S70**. Compound **S70** was obtained as a yellow oil. TLC R_f 0.19 (EtOAc/hexane, 4:1). IR (neat): 2953, 1695, 1449 cm^{-1}. ^{1}H NMR (400 MHz, $CDCl_3$): δ 7.69 (m, 1H, 2-pyridinethione), 7.54 (m, 1H, 2-

[S6] D. H. R. Barton, C. Tachdjian *Tetrahedron* **1992**, *48*, 7109-7120.

pyridinethione), 7.40-7.29 (m, 5H, phenyl), 7.21 (m, 1H, 2-pyridinethione), 6.64 (m, 1H, 2-pyridinethione), 5.13 (s, 2H), 4.18 (br, 2H), 3.11-2.91 (m, 3H), 2.20-2.11 (m, 2H), 1.96-1.84 (m, 2H). $^{13}C\{^{1}H\}$ NMR (100 MHz, $CDCl_3$): δ 175.6, 169.8, 155.0, 137.5, 137.4, 136.5, 133.5, 128.5 (2C), 128.0, 127.8 (2C), 112.6, 67.2, 42.8 (2C), 39.0, 27.6 (2C, br). HRMS (ESI-TOF) *m/z*: $[M + Na]^+$ Calcd for $C_{19}H_{20}N_2NaO_4S$ 395.1041; Found 395.1034.

2-Thioxopyridin-1(2*H*)-yl 2,2-dimethyl-3-phenylpropanoate (S72)

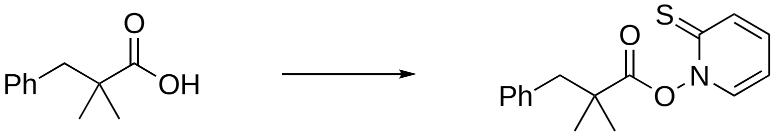

S71 **S72**

As described for the preparation of **1b**, compound **S71** (30.6 mg, 0.172 mmol) was converted to 34.5 mg (70%) of **S72**. Compound **S72** was obtained as a yellow oil. TLC R_f 0.41 (EtOAc/hexane, 1:2). IR (neat): 2977, 1791, 1527 cm^{-1}. ^{1}H NMR (400 MHz, $CDCl_3$): δ 7.65 (m, 1H, 2-pyridinethione), 7.36-7.27 (m, 3H, 2-pyridinethione, phenyl), 7.25-7.13 (m, 4H, 2-pyridinethione, phenyl), 6.57 (m, 1H, 2-pyridinethione), 3.07 (s, 2H), 1.47 (s, 6H). $^{13}C\{^{1}H\}$ NMR (100 MHz, $CDCl_3$): δ 175.9, 172.8, 137.5 137.4, 136.7, 133.3, 130.5 (2C), 128.1 (2C), 126.9, 112.5, 46.1, 43.7, 24.8 (2C). HRMS (ESI-TOF) *m/z*: $[M + Na]^+$ Calcd for $C_{16}H_{17}NNaO_2S$ 310.0878; Found 310.0872.

2-Thioxopyridin-1(2*H*)-yl 3-[(benzyloxy)methoxy]- 2,2-dimethylpropanoate (S74)

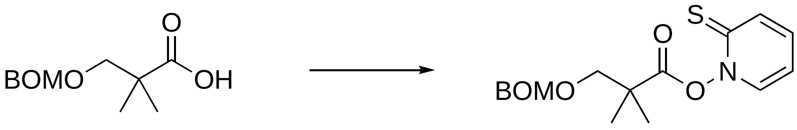

S73 **S74**

As described for the preparation of **1b**, compound **S73** (100 mg, 0.420 mmol) was converted to 101 mg (69%) of **S74**. Compound **S74** was obtained as a yellow oil. TLC R_f 0.17 (EtOAc/hexane, 1:2). IR (neat): 2937, 1798, 1528 cm^{-1}. ^{1}H NMR (400 MHz, $CDCl_3$): δ 7.67 (m, 1H, 2-pyridinethione), 7.48 (m, 1H, 2-pyridinethione), 7.38-7.28 (m, 5H, phenyl), 7.17 (m, 1H, 2-pyridinethione), 6.54 (m, 1H, 2-pyridinethione), 4.81 (s, 2H), 4.62 (s, 2H), 3.78 (s, 2H), 1.50 (s, 6H). $^{13}C\{^{1}H\}$ NMR (100 MHz, $CDCl_3$): δ 176.0, 171.6, 137.8, 137.4, 133.3, 128.4 (2C), 127.8 (2C), 112.4, 95.1, 92.3, 91.2, 74.7, 69.7, 42.7, 22.4 (2C). HRMS (ESI-TOF) *m/z*: $[M + Na]^+$ Calcd for $C_{18}H_{21}NNaO_4S$ 370.1089; Found 370.1083.

2-[(3-Phenethyl-5-phenylpentyl)thio]pyridine (16a)

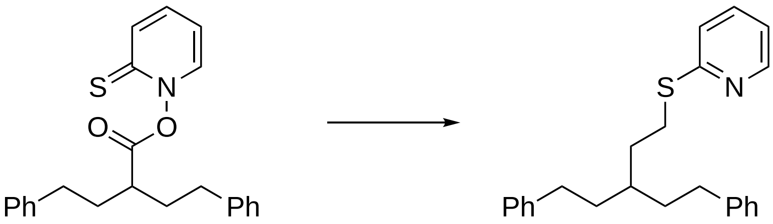

5 **16a**

The following reaction was carried out under Ar. To a stirred solution of **5** (19.8mg, 0.0525 mmol) and zinc(II) tetraphenylporphyrin (1.1 mg, 1.6 μmol) in toluene (0.5 mL) was added acrylic acid (9.0 μL, 0.133 mmol). The mixture was stirred and irradiated by red LEDs at 25 °C for 1 h, and diphenyl disulfide (12.7 mg, 0.0580 mmol), DIPEA (32 μL, 0.19 mmol), TBAI (5.5mg, 0.015mmol), [Ir(dF(CF$_3$)ppy)$_2$(dtbbpy)] PF$_6$ (0.7 mg, 0.7 μmol), toluene (0.5 mL) and H_2O (110 μL) were added. After being stirred and irradiated by blue LEDs at 25 ℃ for 2 d, the mixture was diluted with H_2O (10 mL) and extracted with CH_2Cl_2 (5 mL × 3). The combined extracts were dried and concentrated under reduced pressure. The residue was purified by PTLC (EtOAc/hexane, 1:12) to provide 12.7 mg (67%) of **16a** as a colorless oil. TLC R_f 0.58 (EtOAc/hexane, 1:10). IR (neat): 2925, 1454 cm^{-1}. ^{1}H NMR (400 MHz, CDCl$_3$): δ 8.42 (m, 1H, H-6 of pyridinyl), 7.46 (m, 1H, H-4 of pyridinyl), 7.31-7.25 (m, 4H, Ar), 7.22-7.16 (m, 6H, Ar), 7.15 (m, 1H, H-3 of pyridinyl), 6.96 (m, 1H, H-5 of pyridinyl), 3.22-3.16 (m, 2H, H-1, 1'), 2.67-2.60 (m, 4H), 1.85-1.78 (m, 2H), 1.75-1.67 (m, 4H), 1.65 (m, 1H). ^{13}C{^{1}H} NMR (100 MHz, CDCl$_3$): δ 159.2, 149.4, 142.8 (2C), 135.8, 128.4 (4C), 128.3 (4C), 125.7 (2C), 122.3, 119.2, 36.6, 35.3 (2C), 33.1, 32.9 (2C), 27.4. HRMS (ESI-TOF) *m/z*: [M + H]$^+$ Calcd for $C_{24}H_{28}NS$ 362.1942; Found 362.1935.

2-[(3-Methylpentyl)thio]pyridine (16b)

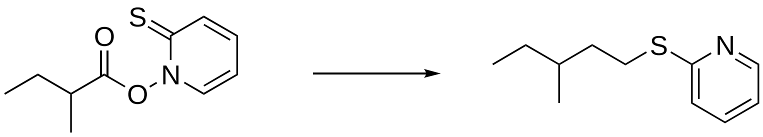

S60 **16b**

As described for the preparation of **16a**, compound **S60** (9.6 mg, 0.0454 mmol) was converted to 4.2 mg (45%) of **16b**. Compound **16b** was obtained as a yellow oil. TLC R_f 0.56 (EtOAc/hexane, 1:10). IR (neat): 2960, 2926, 1579 cm^{-1}. ^{1}H NMR (400 MHz, CDCl$_3$): δ 8.42 (m, 1H), 7.46 (m, 1H), 7.16 (m, 1H), 6.96 (m, 1H), 3.25-3.08 (m, 2H), 1.71 (m, 1H), 1.64-1.47 (m, 2H), 1.38 (m, 1H), 1.20 (m, 1H), 0.93 (d, 3H, J = 6.0 Hz), 0.88 (t, 3H, J = 7.2 Hz). ^{13}C{^{1}H} NMR (100 MHz, CDCl$_3$): δ 159.6, 149.4, 135.8, 122.1, 119.1, 35.9, 34.0, 29.1, 28.1, 18.9, 11.2. HRMS (ESI-TOF) *m/z*: [M + H]$^+$ Calcd for $C_{11}H_{18}NS$ 196.1160; Found 196.1152.

2-[(3-Methyl-4-phenylbutyl)thio]pyridine (16c)

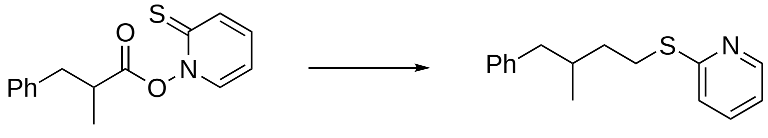

S62 **16c**

As described for the preparation of **16a**, compound **S62** (9.3 mg, 0.0340 mmol) was converted to 3.3 mg (38%) of **16c**. Compound **16c** was obtained as a yellow oil. TLC R_f 0.54 (EtOAc/hexane, 1:10). IR (neat): 2925, 1454 cm^{-1}. ^{1}H NMR (400 MHz, CDCl$_3$): δ 8.41 (m, 1H), 7.45 (m, 1H), 7.30-7.23 (m, 2H, phenyl), 7.20-7.12 (m, 4H), 6.96 (m, 1H), 3.28 (m, 1H), 3.15 (m, 1H), 2.71 (m, 1H), 2.42 (m, 1H), 1.94 (m, 1H), 1.77 (m, 1H), 1.59 (m, 1H), 0.93 (d, 3H, J = 6.8 Hz). ^{13}C{^{1}H} NMR (100 MHz, CDCl$_3$): δ 159.4, 149.4, 141.0, 135.8, 129.2 (2C), 128.1 (2C), 125.7, 121.9, 119.2, 43.2, 36.0, 34.5, 28.0, 19.1. HRMS (ESI-TOF) *m/z*: [M + Na]$^+$ Calcd for $C_{16}H_{19}NNaS$ 280.1136; Found 280.1125.

2-[(3-Methyl-4-phenylbutyl)thio]pyridine (16d)

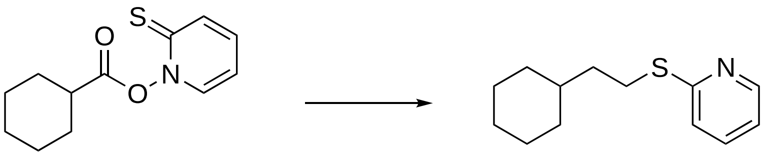

As described for the preparation of **16a**, compound **S64** (9.3 mg, 0.0340 mmol) was converted to 3.3 mg (38%) of **16d**.[S7] Compound **16d** was obtained as a yellow oil. TLC R_f 0.54 (EtOAc/hexane, 1:10). ^{1}H NMR (400 MHz, $CDCl_3$): δ 8.41 (m, 1H), 7.45 (m, 1H), 7.30-7.23 (m, 2H, phenyl), 7.20-7.12 (m, 4H), 6.96 (m, 1H), 3.28 (m, 1H), 3.14 (m, 1H), 2.70 (m, 1H), 2.42 (m, 1H), 1.94 (m, 1H), 1.77 (m, 1H), 1.60 (m, 1H), 0.93 (d, 3H, J = 6.8 Hz).

2-[(2-Cyclobutylethyl)thio]pyridine (16e)

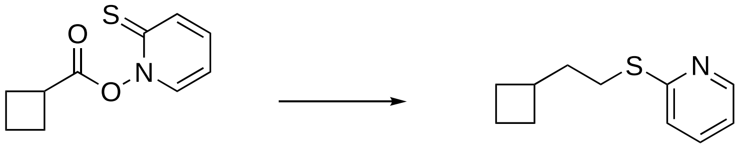

As described for the preparation of **16a**, compound **S66** (10.0 mg, 0.0478 mmol) was converted to 4.5 mg (49%) of **16e**. Compound **16e** was obtained as a yellow oil. TLC R_f 0.53 (EtOAc/hexane, 1:10). IR (neat): 2925, 2854 cm^{-1}. ^{1}H NMR (400 MHz, $CDCl_3$): δ 8.42 (m, 1H), 7.46 (m, 1H), 7.15 (m, 1H), 6.96 (m, 1H), 3.08-3.03 (m, 2H), 2.43 (m, 1H), 2.13-2.03 (m, 2H), 1.93-1.75 (m, 4H), 1.71-1.61 (m, 2H). ^{13}C{^{1}H} NMR (100 MHz, $CDCl_3$): δ 159.6, 149.4, 135.8, 122.1, 119.2, 36.3, 35.4, 28.1 (2C), 28.0, 18.4. HRMS (ESI-TOF) *m/z*: [M + H]$^+$ Calcd for $C_{11}H_{16}NS$ 194.1003; Found 194.0994.

***tert*-Butyl 4-[2-(pyridin-2-ylthio)ethyl]piperidine-1-carboxylate (16f)**

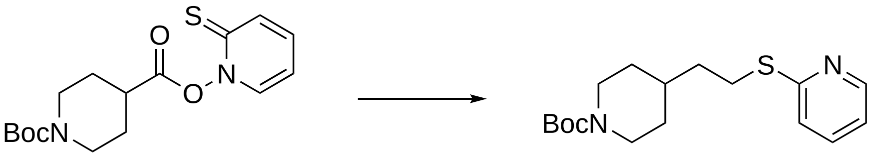

As described for the preparation of **16a**, compound **S68** (8.6 mg, 0.0255 mmol) was converted to 3.3 mg (40%) of **16f**. Compound **16f** was obtained as a yellow oil. TLC R_f 0.49 (EtOAc/hexane, 1:2). IR (neat): 2926, 1693 cm^{-1}. ^{1}H NMR (400 MHz, $CDCl_3$): δ 8.41 (m, 1H), 7.47 (m, 1H), 7.16 (m, 1H), 6.97 (m, 1H), 4.20-3.88 (br, 8/3H), 3.22-3.17 (m, 4/3H), 3.14-3.04 (m, 2/3H), 2.78-2.57 (br, 4/3H), 2.10-2.02 (br, 2/3H), 1.76-1.58 (m, 5H), 1.46 (s, 3H), 1.45 (s, 6H), 1.20-1.07 (m, 4/3H). ^{13}C{^{1}H} NMR (100 MHz, $CDCl_3$): δ

[S7] D. H. R. Barton, H. Togo, S. Z. Zard *Tetrahedron Lett.* **1985**, *26*, 6349-6352.

159.1, 154.9, 149.5, 135.8, 122.2, 119.3, 79.2, 40.4, 35.9, 35.2, 32.1, 31.9, 29.7, 28.5 (3C), 27.3. HRMS (ESI-TOF) *m/z*: $[M + Na]^+$ Calcd for $C_{17}H_{26}N_2NaO_2S$ 345.1613; Found 345.1606.

Benzyl 4-[2-(pyridin-2-ylthio)ethyl]piperidine-1-carboxylate (16g)

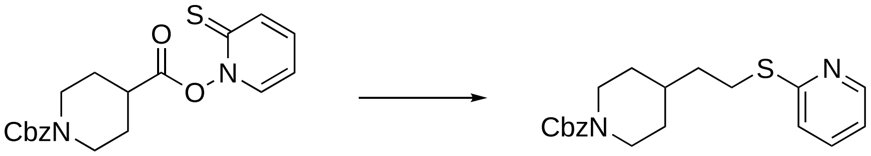

S70 **16g**

As described for the preparation of **16a**, compound **S70** (7.7 mg, 0.207 mmol) was converted to 2.0 mg (27%) of **16g**. Compound **16g** was obtained as a yellow oil. TLC R_f 0.34 (EtOAc/hexane, 1:2). IR (neat): 2924, 1698 cm^{-1}. ^1H NMR (400 MHz, $CDCl_3$): δ 8.41 (m, 1H), 7.47 (m, 1H), 7.40-7.28 (m, 5H, phenyl), 7.16 (m, 1H), 6.98 (m, 1H), 5.12 (s, 2H), 4.24-4.06 (br, 2H), 3.19 (t, 2H, J = 7.2 Hz), 2.87-2.70 (br, 2H), 2.07 (br, 1H), 1.81-1.60 (m, 4H), 1.23-1.08 (m, 2H). $^{13}C\{^1H\}$ NMR (100 MHz, $CDCl_3$): δ 159.0, 155.3, 149.4, 136.9, 135.9, 128.5 (2C), 127.9, 127.8 (2C), 122.3, 119.3, 66.9, 44.1 (2C), 35.8, 35.1, 31.7 (2C, br), 27.2. HRMS (ESI-TOF) *m/z*: $[M + Na]^+$ Calcd for $C_{20}H_{24}N_2NaO_2S$ 379.1456; Found 379.1447.

2-[(3,3-Dimethyl-4-phenylbutyl)thio]pyridine (16h)

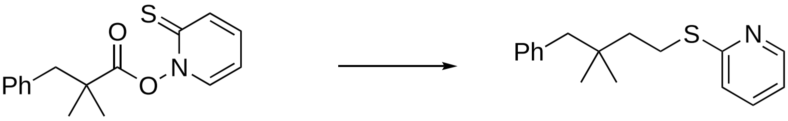

S72 **16h**

As described for the preparation of **16a**, compound **S72** (10.0 mg, 0.0348 mmol) was converted to 5.6 mg (59%) of **16h**. Compound **16h** was obtained as a yellow oil. TLC R_f 0.64 (EtOAc/hexane, 1:10). IR (neat): 2957, 2926, 1578 cm^{-1}. ^1H NMR (400 MHz, $CDCl_3$): δ 8.44 (m, 1H), 7.47 (m, 1H), 7.30-7.14 (m, 6H), 6.97 (m, 1H), 3.24-3.18 (m, 2H), 2.58 (s, 2H), 1.67-1.61 (m, 2H), 0.95 (s, 6H). $^{13}C\{^1H\}$ NMR (100 MHz, $CDCl_3$): δ 159.4, 149.5, 138.8, 135.8, 130.6 (2C), 127.7 (2C), 125.9, 122.1, 119.2, 48.1, 41.5, 34.8, 26.6 (2C), 25.7. HRMS (ESI-TOF) *m/z*: $[M + Na]^+$ Calcd for $C_{17}H_{21}NNaS$ 294.1292; Found 294.1282.

2-{[4-((Benzyloxy)methoxy)-3,3-dimethylbutyl]thio}pyridine (16i)

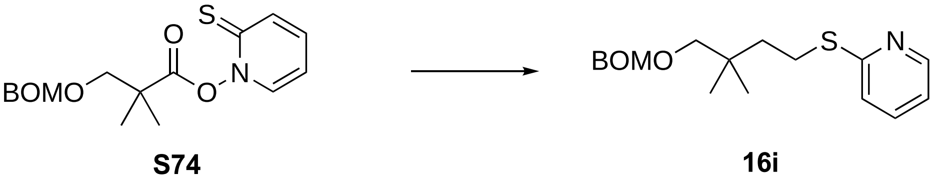

S74 **16i**

As described for the preparation of **16a**, compound **S74** (9.7 mg, 0.0279 mmol) was converted to 5.3 mg (57%) of **16i**. Compound **16i** was obtained as a yellow oil. TLC R_f 0.38 (EtOAc/hexane, 1:10). IR (neat): 2926, 1579 cm^{-1}. ^1H NMR (400 MHz, $CDCl_3$): δ 8.41 (m, 1H), 7.45 (m, 1H), 7.36-7.26 (m, 5H, phenyl), 7.16 (m, 1H), 6.95 (m, 1H), 4.76 (s, 2H), 4.60 (s, 2H), 3.34 (s, 2H), 3.18-3.12 (m, 2H), 1.74-1.68 (m, 2H), 1.00 (s, 6H). $^{13}C\{^1H\}$ NMR (100 MHz, $CDCl_3$): δ 159.5, 149.5, 137.9, 135.8, 128.4 (2C), 127.9 (2C), 127.7,

122.0, 119.2, 94.9, 69.3, 38.8, 34.8, 25.5, 24.4 (2C) (one carbon missing due to overlap). HRMS (ESI-TOF) *m/z*: [M + Na]+ Calcd for $C_{19}H_{25}NNaO_2S$ 354.1504; Found 354.1496.

2-{[6-(2,5-Dimethylphenoxy)-3,3-dimethylhexyl]thio}pyridine (16j)

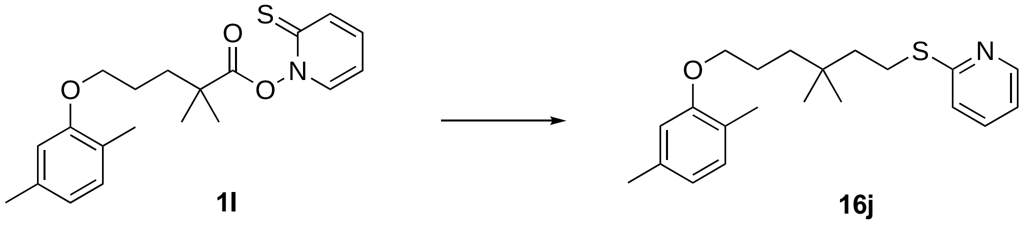

As described for the preparation of **16a**, compound **1l** (8.8 mg, 0.0245 mmol) was converted to 4.2 mg (53%) of **16j**. Compound **16j** was obtained as a yellow oil. TLC R_f 0.59 (EtOAc/hexane, 1:10). IR (neat): 2955, 1414 cm^{-1}. ^1H NMR (400 MHz, $CDCl_3$): δ 8.41 (m, 1H), 7.46 (m, 1H), 7.16 (m, 1H), 7.00 (d, 1H, *J* = 7.2 Hz , Ar), 6.95 (m, 1H), 6.65 (d, 1H, *J* = 7.2 Hz , Ar), 6.63 (s, 1H), 3.94 (t, 2H, *J* = 6.4 Hz) , 3.19-3.09 (m, 2H), 2.31 (s, 3H), 2.18 (s, 3H), 1.85-1.76 (m, 2H), 1.68-1.61 (m, 2H), 1.47-1.41 (m, 2H), 0.98 (s, 6H). $^{13}C\{^1H\}$ NMR (100 MHz, $CDCl_3$): δ 159.4, 157.1, 149.5, 136.4, 135.8, 130.2, 123.6, 122.1, 120.6, 119.2, 111.9, 68.5, 41.1, 37.7, 33.2, 29.7, 27.1, 25.5, 24.2, 21.4, 15.8. HRMS (ESI-TOF) *m/z*: [M + Na]+ Calcd for $C_{21}H_{29}NNaOS$ 366.1868; Found 366.1859.

3-Phenethyl-5-phenylpentan-1-ol (17a)

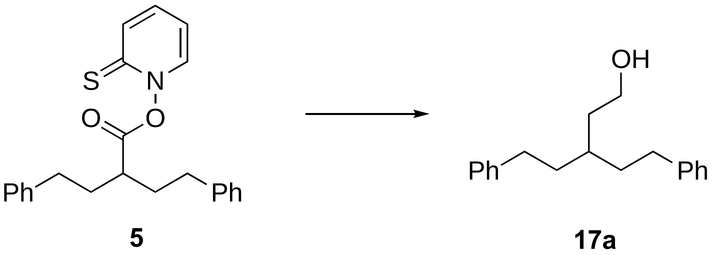

To a stirred solution of **5** (10.0 mg, 0.0265 mmol) and zinc(II) tetraphenylporphyrin (0.6 mg, 0.9 µmol) in toluene (0.3 mL) was added acrylic acid (8.0 µL, 0.012 mmol). The mixture was stirred and irradiated by red LEDs at 25 °C for 1 h under Ar, and Na_2CO_3 (16.9 mg, 0.159 mmol), TBAI (2.0 mg, 0.0052 mmol), $[Ir(dF(CF_3)ppy)_2(dtbbpy)]\ PF_6$ (0.3 mg, 0.3 µmol), toluene (1.0 mL) and H_2O (130 µL) were added. The mixture was stirred and irradiated by blue LEDs at 25 °C for 1 d under O_2, and $NaBH_4$ (20.0 mg, 0.528 mmol) was added. After being stirred for 30 min, the mixture was diluted with H_2O (10 mL) and extracted with CH_2Cl_2 (5 mL × 3). The combined extracts were dried and concentrated under reduced pressure. The residue was purified by PTLC (EtOAc/hexane, 1:2) to provide 4.2 mg (59%) of **17a** as a colorless oil. TLC R_f 0.47 (EtOAc/hexane, 1:2). IR (neat): 3356, 2927 cm^{-1}. ^1H NMR (400 MHz, $CDCl_3$): δ 7.31-7.26 (m, 4H, Ar), 7.21-7.15 (m, 6H, Ar), 3.70 (t, 2H, *J* = 7.2 Hz, H-1, 1'), 2.63 (t, 4H, *J* = 7.6 Hz), 1.74-1.61 (m, 6H), 1.59 (m, 1H). $^{13}C\{^1H\}$ NMR (100 MHz, $CDCl_3$): δ 142.7, 128.4, 128.3, 125.7, 60.9, 36.6, 35.6 (2C), 33.6, 32.9 (2C). HRMS (ESI-TOF) *m/z*: [M + Na]+ Calcd for $C_{19}H_{25}NaO$ 291.1725; Found 291.1714.

3-Methylpentan-1-ol (17b)

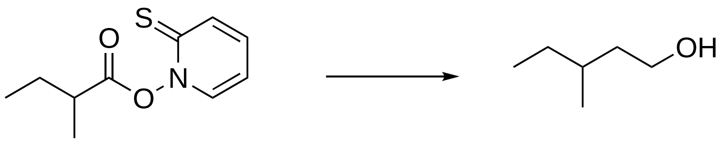

S60 **17b**

As described for the preparation of **17a**, compound **S60** (10.0 mg, 0.0473 mmol) was converted to **17b**. 46% yield of **17b** was calculated according to the GC results. **17b** is a commercially available compound.

3-Methyl-4-phenylbutan-1-ol (17c)

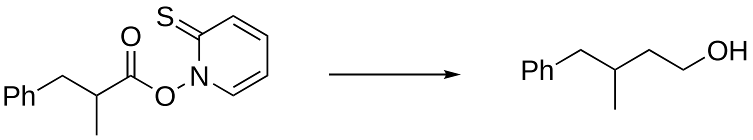

S62 **17c**

As described for the preparation of **17c**, compound **S62** (6.5 mg, 0.0238 mmol) was converted to 2.1 mg (53%) of **17c**.[S8] Compound **17c** was obtained as a yellow oil. TLC R_f 0.65 (EtOAc/hexane, 1:2). ^{1}H NMR (400 MHz, $CDCl_3$): δ 7.35–7.25 (m, 2H), 7.21–7.13 (m, 3H), 3.79–3.61 (m, 2H), 2.64 (m, 1H), 2.45 (m, 1H), 1.90 (m, 1H), 1.66 (m, 1H), 1.49–1.35 (m, 1H), 0.90 (d, 3H, J = 6.8 Hz).

2-Cyclohexylethan-1-ol (17d)

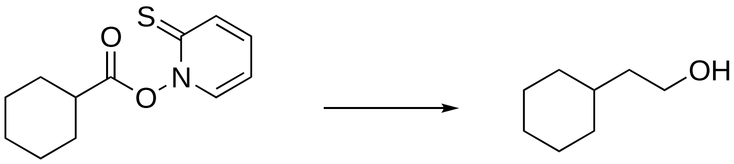

S64 **17d**

As described for the preparation of **17a**, compound **S64** (9.8 mg, 0.0413 mmol) was converted to 2.5 mg (46%) of **17d**. Compound **17d** was obtained as a yellow oil. TLC R_f 0.35 (EtOAc/hexane, 1:2). ^{1}H NMR (400 MHz, $CDCl_3$): δ 3.68 (t, 2H, J = 6.8 Hz), 1.75-1.61 (m, 5H), 1.50-1.43 (m, 2H),1.39 (m, 1H), 1.32-1.08 (m, 3H), 0.98-0.85 (m, 2H). **17d** is a commercially available compound.

***tert*-Butyl 4-(2-hydroxyethyl)piperidine-1-carboxylate (17f)**

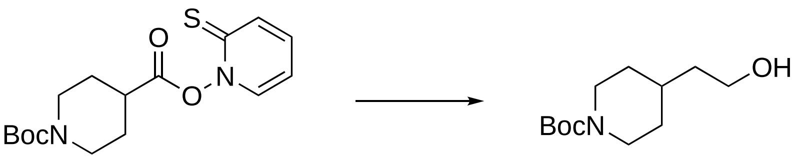

S68 **17f**

As described for the preparation of **17a**, compound **S68** (9.2 mg, 0.0272 mmol) was converted to 3.0 mg (48%) of **17f**.[S9] Compound **17f** was obtained as a yellow oil. TLC R_f 0.24 (EtOAc/hexane, 1:2). ^{1}H NMR

[S8] J. V. Braun, G. Kirschbaum *Ber. Dtsch. Chem. Ges.* **1914**, *47*, 262.

[S9] S. M. N. Efange, R. H. Michelson, B. Knusel, F. Hefti, R. J. Boudreau, J. R. Thomas, J. R.

(400 MHz, $CDCl_3$): δ 4.16-3.98 (br, 2H), 3.71 (t, 2H, J = 6.8 Hz), 2.78-2.60 (br, 2H), 1.71-1.48 (m, 5H), 1.45 (s, 9H), 1.18-1.06 (m, 2H).

Benzyl 4-(2-hydroxyethyl)piperidine-1-carboxylate (17g)

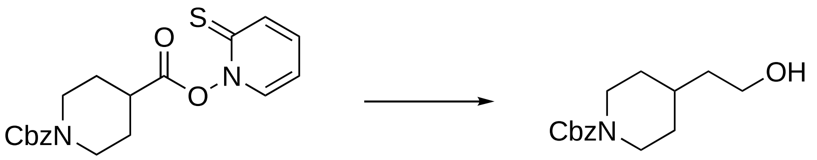

S70 **17g**

As described for the preparation of **17a**, compound **S70** (7.2 mg, 0.0193 mmol) was converted to 3.4 mg (66%) of **17g**.[S10] Compound **17g** was obtained as a yellow oil. TLC R_f 0.12 (EtOAc/hexane, 1:2). ^{1}H NMR (400 MHz, $CDCl_3$): δ 7.41-7.28 (m, 5H, phenyl), 4.29-4.04 (br, 2H), 3.71 (t, 2H, J = 6.8 Hz), 2.88-2.66 (br, 2H), 1.93-1.40 (m, 5H), 1.23-1.06 (m, 2H).

3,3-Dimethyl-4-phenylbutan-1-ol (17h)

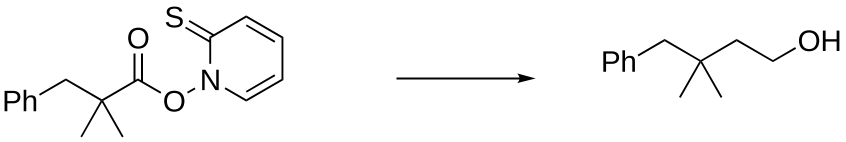

S72 **17h**

As described for the preparation of **17a**, compound **S72** (10.0 mg, 0.0348 mmol) was converted to 3.5 mg (56%) of **17h**.[S11] Compound **17h** was obtained as a yellow oil. TLC R_f 0.46 (EtOAc/hexane, 1:2). ^{1}H NMR (400 MHz, $CDCl_3$): δ 7.30–7.24 (m, 2H), 7.23–7.18 (m, 1H), 7.15–7.11 (m, 2H), 3.77 (t, 2H, J = 7.2 Hz), 2.54 (s, 2H), 1.55 (t, 2H, J = 7.2 Hz), 0.91 (s, 6H).

4-[(Benzyloxy)methoxy]-3,3-dimethylbutan-1-ol (17i)

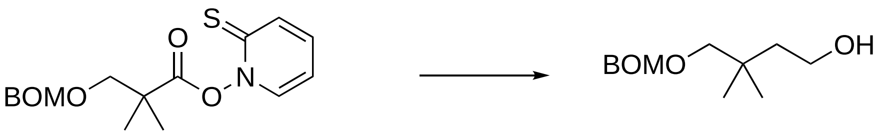

S74 **17i**

As described for the preparation of **17a**, compound **S74** (9.2 mg, 0.0265 mmol) was converted to 3.8 mg (65%) of **17i**. Compound **17i** was obtained as a yellow oil. TLC R_f 0.33 (EtOAc/hexane, 1:2). IR (neat): 3406, 2927 cm^{-1}. ^{1}H NMR (400 MHz, $CDCl_3$): δ 7.37-7.27 (m, 5H, phenyl), 4.77 (s, 2H), 4.61 (s, 2H), 3.70 (t, 2H, J = 6.8 Hz), 3.34 (s, 2H), 1.61 (t, 2H, J = 6.8 Hz), 0.96 (s, 6H). $^{13}C\{^1H\}$ NMR (100 MHz, $CDCl_3$): δ 137.7, 128.5 (2C), 127.9 (2C), 127.8, 94.9, 69.5, 59.5, 42.9, 33.8, 25.2 (2C). HRMS (ESI-TOF) *m/z*: [M + Na]$^+$ Calcd for $C_{14}H_{22}NaO_3$ 261.1467; Found 261.1456.

Tennison *Nucl. Med. Biol.* **1993**, *20,* 527.

[S10] R. Brehm, D. Ohnhäuser, H. Gerlach *Helv. Chim. Acta* **1987**, *70,* 1981.

[S11] G. L. Goerner *J. Org. Chem.* **1959**, *24,* 888-891.

6-(2,5-Dimethylphenoxy)-3,3-dimethylhexan-1-ol (17j)

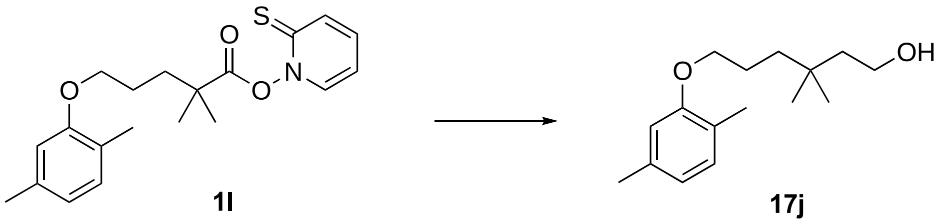

1l

17j

As described for the preparation of **17a**, compound **1l** (10.0 mg, 0.0278 mmol) was converted to 4.3 mg (62%) of **17j**. Compound **17j** was obtained as a yellow oil. TLC R_f 0.47 (EtOAc/hexane, 1:2). IR (neat): 3375, 2955 cm^{-1}. ^{1}H NMR (400 MHz, $CDCl_3$): δ 7.01 (d, 1H, J = 7.2 Hz), 6.68-6.61 (m, 2H), 3.92 (t, 2H, J = 6.4 Hz), 3.73 (t, 2H, J = 7.6 Hz), 2.31 (s, 3H), 2.18 (s, 3H), 1.81-1.72 (m, 2H), 1.57 (m, 1H) 1.41-1.36 (m, 2H), 0.95 (s, 6H). ^{13}C{^{1}H} NMR (100 MHz, $CDCl_3$): δ 157.0, 136.4, 130.3, 123.5, 120.6, 111.9, 68.4, 59.8, 44.2, 38.6, 32.0, 27.5 (2C), 24.2, 21.4, 15.8. HRMS (ESI-TOF) m/z: $[M + Na]^+$ Calcd for $C_{16}H_{26}NaO_2$ 273.1830; Found 273.1818.

5. Stern–Volmer emission quenching

Determination of photon flux:

Photon flux of the reaction equipment was determined by the method described by Brauer.[S12] Briefly, methylene blue (8×10^{-5} M) and *meso*-diphenylhelianthrene[S13] (1×10^{-4} M) was dissolved in air-saturated chloroform. The change in absorbance A_a was recorded at wavelength λ_a = 405 nm, and plotted against irradiation time using red light irradiation equipment. The slope of the plot was used to calculate the photon flow I_λ according to the following equation:

$$\frac{\Delta A_a}{\Delta t} = \frac{0.96 d Q_{\mathrm{PO}} \Delta\epsilon_a}{V} \times I_\lambda$$

Where d = 1 cm (optical path length), $Q_{\mathrm{PO}}\Delta\varepsilon_a = 1.930 \times 10^6$ cm^2 M^{-1} (actinometric factor), V = 3.0 mL (volume). Photon flow $I_\lambda = 1.03 \times 10^{-7}$ einstein min^{-1} was thus obtained.

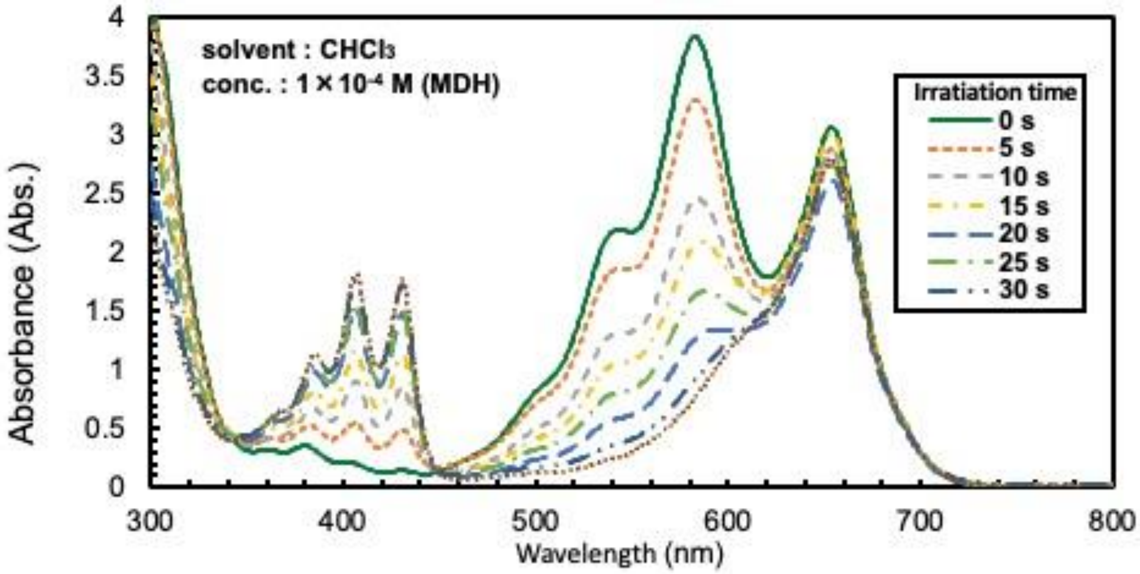

Figure S2 UV-vis spectra after red light irradiation.

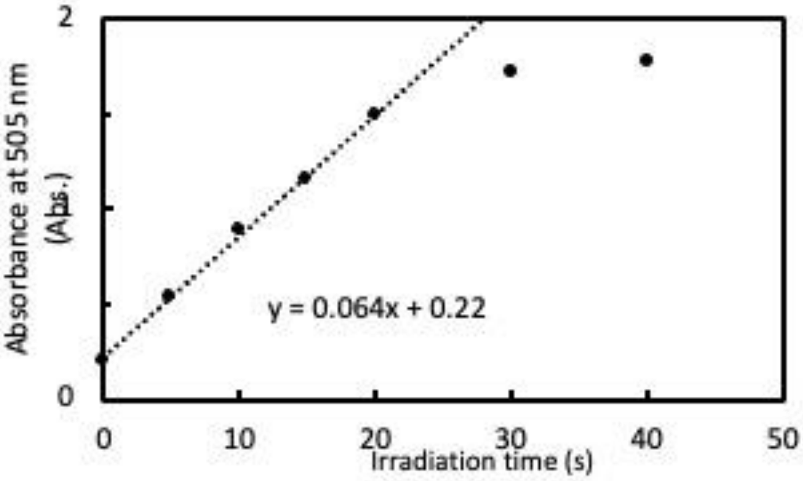

Figure S3 Absorption at 405 nm as a function of red light irradiation time.

[S12] H.-J. Adick, R. Schmidt, H.-D. Brauer *J. Photochem. Photobiol. A* **1989**, *49*, 311-316.

[S13] G. Sauvage *Ann. Chim.* **1947**, *2*, 844-852.

Determination of quantum yield:[S14]

A 4 mL vial was charged with Barton ester **1a** (195 mg, 0.50 mmol), *t*-dodecanethiol (0.47 mL, 2.0 mmol), ZnTPP (0.3 mg, 0.0045 mmol) and MeCN (2.5 mL). The resulting mixture was irradiated with red LED equipment shown in Experimental procedure.

The quantum yield ϕ was calculated according to the following equation:

$$\phi = \frac{n}{I_\lambda t(1-10^{-A})}$$

Where $n = 9.69\times10^{-5}$ mmol (product obtained), $t = 200$ s (reaction time), $A = 0.034$ (absorbance at 630 nm of the ZnTPP (MeCN)). The quantum yield $\phi = 62$ was thus obtained.

Stern–Volmer emission quenching:

A 4 mL vial was charged with ZnTPP, MeCN and indicated concentration of Barton ester **1a** or *t*-dodecanethiol. Fluorescence was measured at excitation wavelength of 630 nm. Stern–Volmer equation for **1a** is the following:

$$I_0/I = 1 + k_{q,\mathbf{1a}}\tau_0[\mathbf{1a}]$$

Where $k_{q,\mathbf{1a}}$ is quencher rate constant for **1a**, τ_0 is fluorescence lifetime of ZnTPP in MeCN (1.9 ns).[S15] $k_{q,\mathbf{1a}}$ is thus calculated to be 1.0×10^{12} ($M^{-1}s^{-1}$). Similarly, $k_{q,\text{thiol}}$ was calculated to be 1.4×10^{9} ($M^{-1}s^{-1}$).

Average chain length:

The average chain length can be estimated by calculating ϕ/Q.[S14] Q is the quenching fraction and is calculated according to the following equation:

$$Q = \frac{k_{q,\mathbf{1a}}[\mathbf{1a}]}{\tau_0^{-1} + k_{q,\mathbf{1a}}[\mathbf{1a}] + k_{q,\text{thiol}}[\text{thiol}]}$$

Where k_q = quenching rate ($M^{-1}s^{-1}$, vide supra), $\tau_0 = 1.9$ ns (life time of excited state of ZnTPP in MeCN).[S15] $Q = 0.99$ was thus obtained. Average chain length ϕ/Q was calculated to be 63.

[S14] M. A. Cismesia, T. P. Yoon *Chem. Sci.* **2015**, *6*, 5426-5434.

[S15] M. Ghosh, A. K. Mora, S. Nath, A. K. Chandra, A. Hajra, S. Sinha *Spectrochim. Acta A* **2013**, *116*, 466-472.

6. Differential pulse voltammetry

The electrochemical measurements were conducted in an Ar filled glove box with a continuous gas purified system at 298 K using a computerized electrochemical system (HZ-7000, Hokuto Denko). The dried **1a** was dissolved in super hydrated acetonitrile containing 0.1 M tetrabutylammonium perchlorate as a supporting electrolyte. Pt was used as a working and counter electrode, respectively. Reference electrode was an Ag wire immersed in acetonitrile containing 0.1 M TBAP and 10 mM $AgClO_4$. The inner electrolyte of the reference electrode was separated from the sample electrolyte by a porous glass (Vycor). The middle potential of ferrocene (Fc) | ferrocenium (Fc^+) in acetonitrile was 0.04 V vs. Ag|Ag(I).

Figure S2 shows the differential pulse voltammogram of a Pt electrode in 10 mM **1a** / acetonitrile. The cathodic current was observed around –2.01 V vs. Ag|Ag(I), e.g. -1.76 V vs SCE.

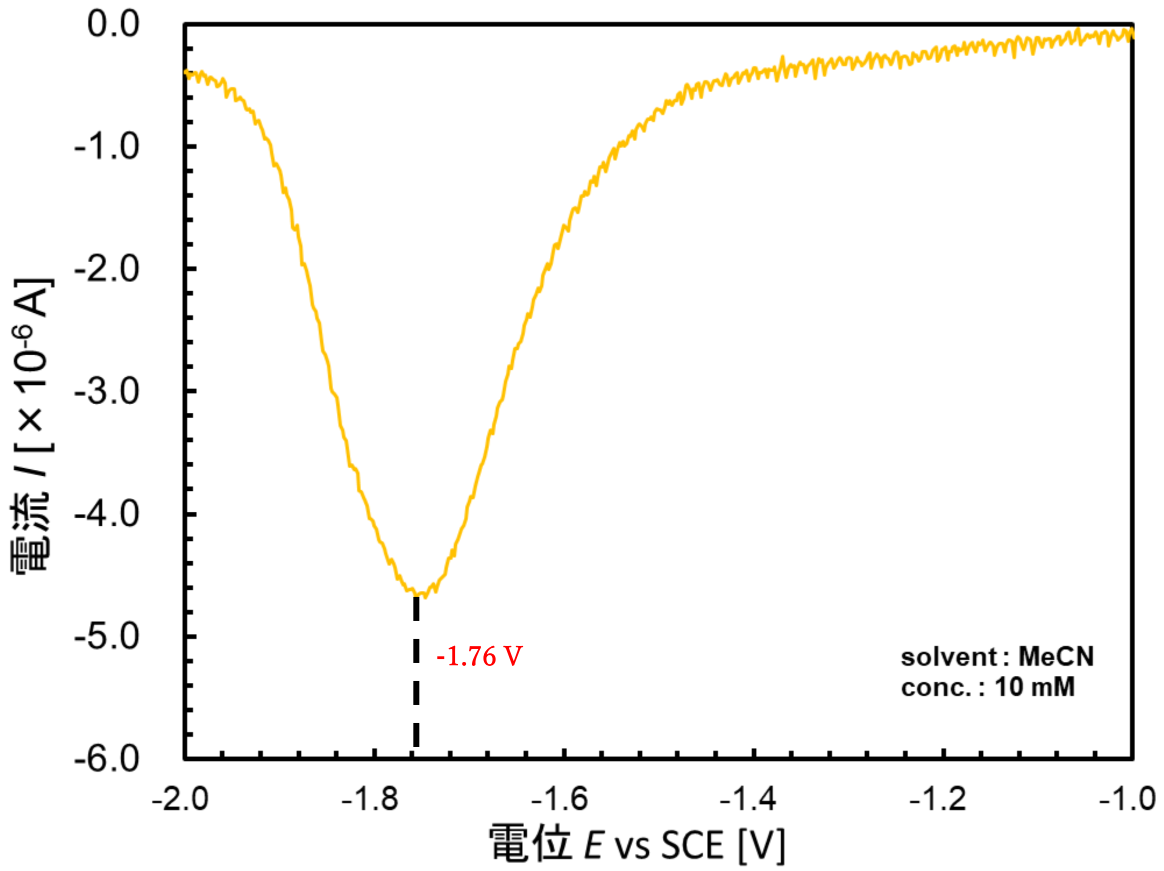

Figure S4 Differential pulse voltammogram of **1a**

7. HPLC profiles of ZnTPP before/after the reaction

A stirred solution of **1a** (30.0 mg), *tert*-dodecanethiol (73.1 μL) and zinc(II) tetraphenylporphyrin 1 M solution in MeCN (0.388 mL) was irradiated by red LEDs at 25 °C for 15 min. Aliquots (0.10 mL) before or after the reaction were taken and diluted with 0.40 mL of 0.025 M H_2TPP (reference) solution in MeCN. 10 μL of the resulting mixture was injected to HPLC and eluted with acetone/MeCN = 1:20 at flow rate of 1 mL/min.

before irradiation

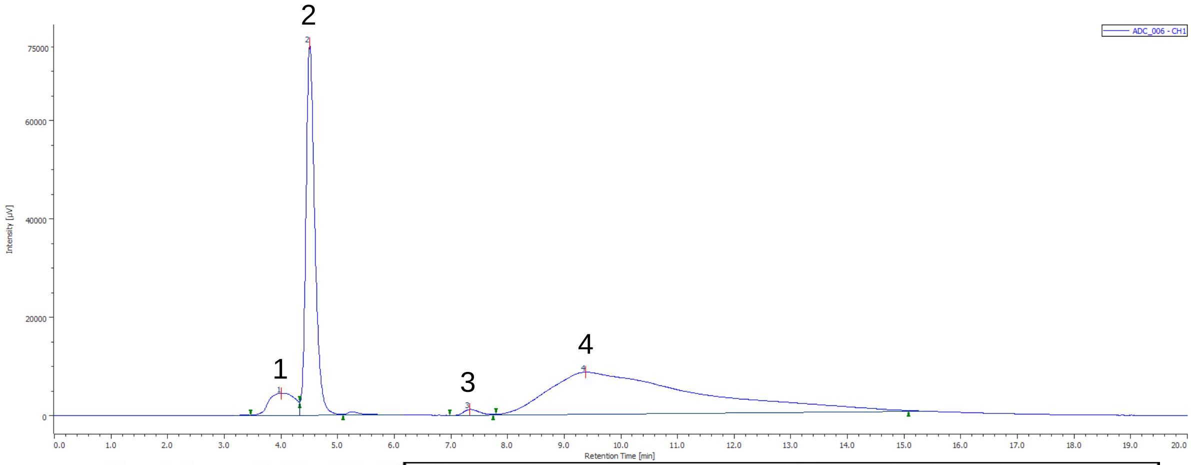

Concentration	ZnTPP	0.02 mM
	H2TPP	0.002 mM
Injection		10 μL
Detection		420 nm
Eluent	acetone/MeCN	1:20

Peak	tR	Area	Height	Area%	Height%	NTP	Resolution	Symmetry factor
1	4	141787	4455	5.588	4.97	N/A	N/A	N/A
2(**1a**)	4.508	821145	75497	32.36	84.231	4308	7.255	1.28
3(ZnTPP)	7.333	22103	1156	0.871	1.289	3359	0.835	N/A
4	9.375	1552468	8523	61.181	9.509	73	N/A	2.41

after irradiation

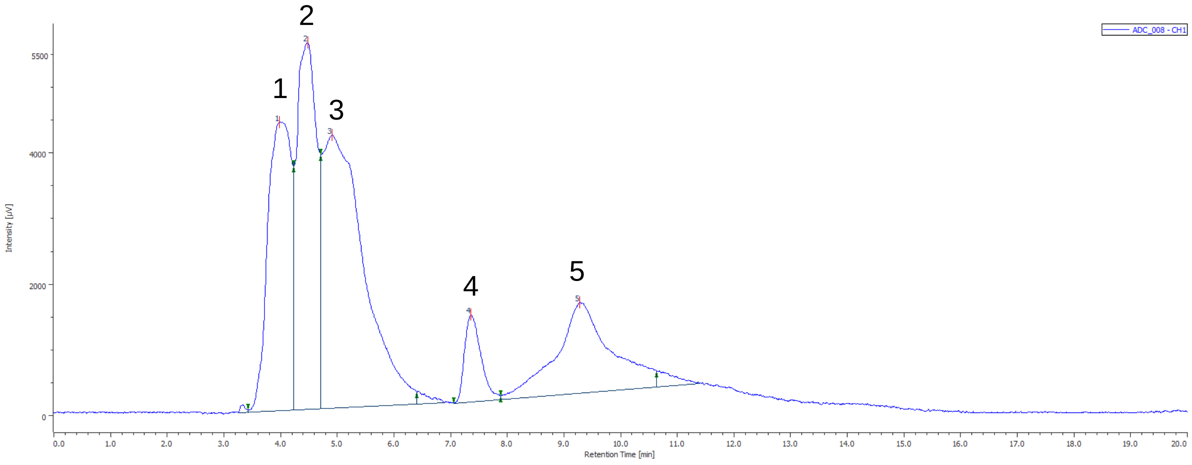

Concentration	ZnTPP	0.02 mM
	H2TPP	0.002 mM
Injection		10 μL
Detection		420 nm
Eluent	acetone/MeCN	1:20

Peak	tR	Area	Height	Area%	Height%	NTP	Resolution	Symmetry factor
1	3.983	127555	4395	21.486	26.108	N/A	N/A	N/A
2	4.475	136250	5581	22.951	33.158	N/A	N/A	N/A
3	4.908	211447	4155	35.618	24.682	N/A	N/A	N/A
4(ZnTPP)	7.358	26482	1324	4.461	7.863	3106	2.128	1.589
5	9.275	91922	1378	15.484	8.188	843	N/A	N/A

8. UV-vis spectra of ZnTPP and $Ru(bpy)_3Cl_2$

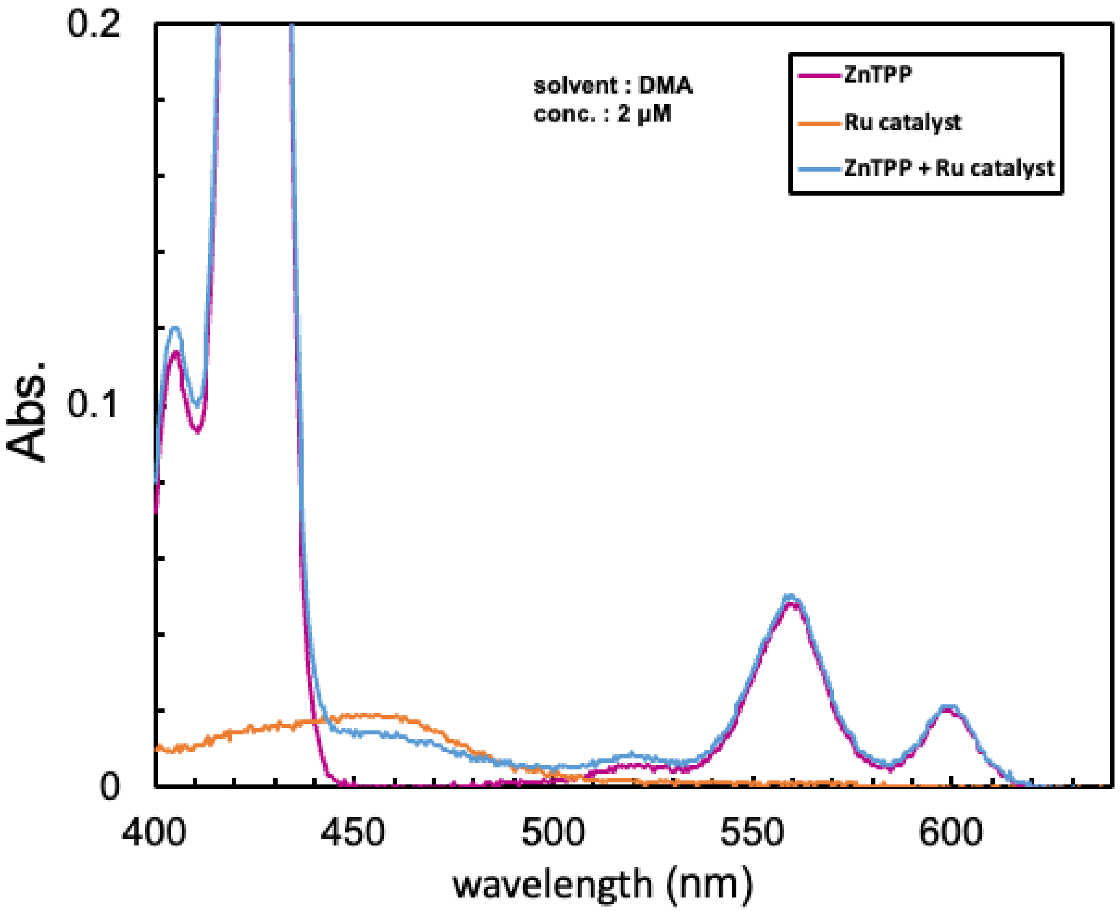

Figure S5 UV-vis spectra of ZnTPP and $Ru(bpy)_3Cl_2$

Several mechanistic possibilities can be postulated for 17% conversion of **12** to **13** when red-red light was used.

(I) Red-light activation of $Ru(bpy)_3Cl_2$: We believe this unlikely, because UV-vis spectra of $Ru(bpy)_3Cl_2$ has little absorption peak in the red light region compared to co-existing ZnTPP, as shown obove.

(II) Partial photoredox reaction by ZnTPP: Activated ZnTPP can convert phthalimidyl ester. However, this process is supposed to be slow, because photoredox is not a chain mechanism (photoredox catalyst requires activation every time for conversion of each substrate molecule), and difference in redox potentials is not so large.

(III) Activation of BNAH-phthalimidyl EDA complex by ZnTPP.

(IV) Reduction of activated ZnTPP by BNAH to form $ZnTPP^{\bullet -}$.

9. Computational methods

Compound **19** was subjected to a conformational search using the OPLS3 force field as implemented in MacroModel. Both singlet and triplet states for each conformers were further optimized using B3LYP/6-311+G(d,p) with the CPCM MeCN model in Jaguar. Free energies at B3LYP/6-311+G(3df,3dp) were calculated respectively using Jaguar. The S–T gap was computed as the difference between the two free energies.

Complex of compound **19** and ZnTPP was subjected to a conformational search using the OPLS3 force field as implemented in MacroModel. The obtained structure was further optimized using LANL2TZ for Zn and B3LYP-D3/6-31G(d,p) for other atoms in vacuum in Jaguar. Free energies were also calculated at the same level of theory.

References for computational methods (also, see ref. 72 of the main article)

B3LYP: a) A. D. Becke, *J. Chem. Phys*. **1993**, *98*, 1372; b) A. D. Becke, *J. Chem. Phys*. **1993**, *98*, 5648; c) C. Lee,; W. Yang, R. G. Parr, *Phys. Rev. B*. **1988**, *37*, 785; d) P. J. Stephens, F. J. Devlin, C. F. Chabalowski, M. J. Frisch, *J. Phys. Chem*. **1994**, *98*, 11623; e) J. Tirado-Rives, W. L. Jorgensen, *J. Chem. Theory Comput*. **2008**, *4*, 297.

Macromodel: Schrödinger Release 2020-3, Schrödinger, LLC, New York, NY, 2020.

Jaguar: a) Schrödinger Release 2020-3, Schrödinger, LLC, New York, NY, 2020; b) Bochevarov, A.D.; Harder, E.; Hughes, T.F.; Greenwood, J.R.; Braden, D.A.; Philipp, D.M.; Rinaldo, D.; Halls, M.D.; Zhang, J.; Friesner, R.A., "Jaguar: A high-performance quantum chemistry software program with strengths in life and materials sciences," *Int. J. Quantum Chem.*, **2013**, *113(18)*, 2110-2142

Computed coordinates and energies for **19**

Coordinates of S_0:

C -4.43250 -1.95450 0.54360
C -4.63010 -1.99310 -0.85570
C -3.72130 -1.40460 -1.70240
C -2.55770 -0.73850 -1.22450
N -2.43520 -0.76240 0.15260
C -3.31880 -1.32520 1.02680
S -1.41000 0.02420 -2.22070
O -1.37800 -0.06990 0.73590
C -0.17370 -0.79780 0.81430
C 0.91990 0.09560 1.35360
O -0.11190 -1.94870 0.49330
C 1.76110 0.64640 0.17970
C 1.78510 -0.68440 2.35510
H -5.13510 -2.40480 1.23380
H -5.50600 -2.48490 -1.26800

H -3.86520 -1.42170 -2.77520
H -3.06920 -1.22570 2.07710
H 0.43780 0.93630 1.86020
H 1.15310 1.22830 -0.51470
H 2.54380 1.29530 0.58140
H 2.23860 -0.16710 -0.37350
H 2.28060 -1.52830 1.86810
H 2.55370 -0.02210 2.76160
H 1.18870 -1.06700 3.18740
solution phase energy = -953.170893 Hartree

Coordinates of T_1 :
C -3.93460 -3.01390 -0.23670
C -4.88210 -2.29470 -0.96130
C -4.59540 -0.98490 -1.32860
C -3.34900 -0.40500 -0.95920
N -2.45020 -1.14140 -0.24980
C -2.71900 -2.38670 0.09920
S -2.97920 1.21110 -1.40600
O -0.44540 -0.05570 0.37320
C 0.43470 -0.76500 0.99500
C 1.68500 0.12400 1.28200
O 0.36090 -1.94350 1.32990
C 2.89560 -0.46710 0.54510
C 1.89750 0.21910 2.79940
H -4.11040 -4.03860 0.07270
H -5.82960 -2.74620 -1.23800
H -5.30020 -0.38650 -1.89510
H -1.95170 -2.90820 0.66190
H 1.48650 1.12450 0.88910
H 2.72620 -0.50870 -0.53440
H 3.77190 0.16380 0.72430
H 3.11750 -1.47640 0.90160
H 2.09010 -0.76620 3.23120
H 2.75940 0.86180 3.00430
H 1.02810 0.65470 3.29940
solution phase energy = -953.116722 Hartree

triplet energy = 0.054171 Hartree = 142.23 kJ/mol

Computed coordinates and energies for **19** and ZnTPP

C 13.41467 -1.56237 3.11929

C 14.84358 -1.10137 3.21325

C 12.48014 -0.41945 3.56886

C 13.21878 -2.83768 3.94381

O 15.15513 -0.29172 2.07951

O 15.66854 -1.33641 4.04594

N 16.39490 0.31903 2.18849

C 17.44589 -0.39775 1.70192

C 16.44693 1.57391 2.79046

C 18.70396 0.12416 1.76709

C 17.77974 2.10520 2.81731

S 15.08885 2.33303 3.40792

C 18.86267 1.41142 2.34310

C 16.61969 2.25282 -0.95121

N 15.36277 2.24456 -0.39160

C 15.04731 3.54104 -0.07123

C 13.82278 3.99514 0.45898

C 16.14848 4.40581 -0.43860

C 17.10838 3.61668 -0.99649

C 17.32093 1.11960 -1.41033

C 12.66694 3.21383 0.64913

C 13.75627 5.43840 0.84438

C 11.39179 3.71961 1.11391

N 12.55526 1.86784 0.39913

C 11.25928 1.50368 0.67468

C 10.73179 0.19923 0.57453

C 10.51952 2.67379 1.10670

C 9.29654 0.00293 0.94548

C 11.46008 -0.95095 0.19324

N 12.78418 -0.98050 -0.17661

C 13.12747 -2.29815 -0.38276

C 14.40644 -2.77209 -0.74255

C 11.96880 -3.13168 -0.14444

C 10.94515 -2.30411 0.21411

C 15.53145 -1.96998 -1.03677

C 16.83795 -2.47219 -1.40477

N 15.56859 -0.59483 -1.01259

C 16.83445 -0.20852 -1.38022
C 17.63480 -1.38710 -1.63317
C 18.69874 1.32214 -1.94718
C 14.58514 -4.25560 -0.81384
C 12.97580 6.35117 0.12054
C 12.93183 7.69625 0.48924
C 13.66762 8.14583 1.58756
C 14.44698 7.24314 2.31440
C 14.49404 5.89829 1.94602
C 8.84890 0.25377 2.25265
C 7.51195 0.05790 2.59957
C 6.59805 -0.39195 1.64458
C 7.03002 -0.64245 0.34038
C 8.36710 -0.44653 -0.00574
C 14.84654 -4.89588 -2.03481
C 15.01555 -6.27975 -2.09151
C 14.92568 -7.04621 -0.92761
C 14.66441 -6.42084 0.29344
C 14.49535 -5.03726 0.34867
C 19.71118 1.89868 -1.16297
C 20.99840 2.07213 -1.67102
C 21.29972 1.67243 -2.97462
C 20.30210 1.10011 -3.76682
C 19.01543 0.92697 -3.25744
Zn 14.10742 0.61589 -0.14525
H 13.21474 -1.76530 2.06583
H 12.63336 0.47768 2.96712
H 11.44501 -0.75217 3.45614
H 12.65939 -0.16394 4.61749
H 13.88408 -3.63916 3.60758
H 13.42598 -2.65324 5.00154
H 12.18615 -3.18224 3.84095
H 17.19002 -1.34894 1.25558
H 19.53700 -0.43096 1.35687
H 17.88697 3.09006 3.25456
H 19.85259 1.85404 2.40358
H 16.16384 5.47841 -0.31491
H 18.05094 3.93009 -1.41863
H 11.19228 4.74279 1.39478

H 9.47242 2.69012 1.36869
H 11.94195 -4.20673 -0.24186
H 9.93536 -2.58421 0.47396
H 17.10917 -3.51465 -1.48187
H 18.67591 -1.38644 -1.91898
H 12.40862 5.99913 -0.73637
H 12.32630 8.39295 -0.08384
H 13.63303 9.19292 1.87535
H 15.01681 7.58489 3.17412
H 15.08496 5.18575 2.51461
H 9.55859 0.60601 2.99471
H 7.18581 0.25396 3.61714
H 5.55690 -0.54481 1.91405
H 6.32448 -0.98667 -0.41061
H 8.70312 -0.63774 -1.02057
H 14.91155 -4.29846 -2.93930
H 15.21271 -6.75963 -3.04601
H 15.05729 -8.12352 -0.97193
H 14.59554 -7.00928 1.20406
H 14.29188 -4.54918 1.29762
H 19.47706 2.20782 -0.15059
H 21.76816 2.51738 -1.04649
H 22.30222 1.80777 -3.37042
H 20.52384 0.79271 -4.78495
H 18.23887 0.48666 -3.87540

HOMO energy: -0.17951 hartrees
LUMO energy: -0.08108 hartrees

10. ^{1}H and ^{13}C NMR spectra of new compounds

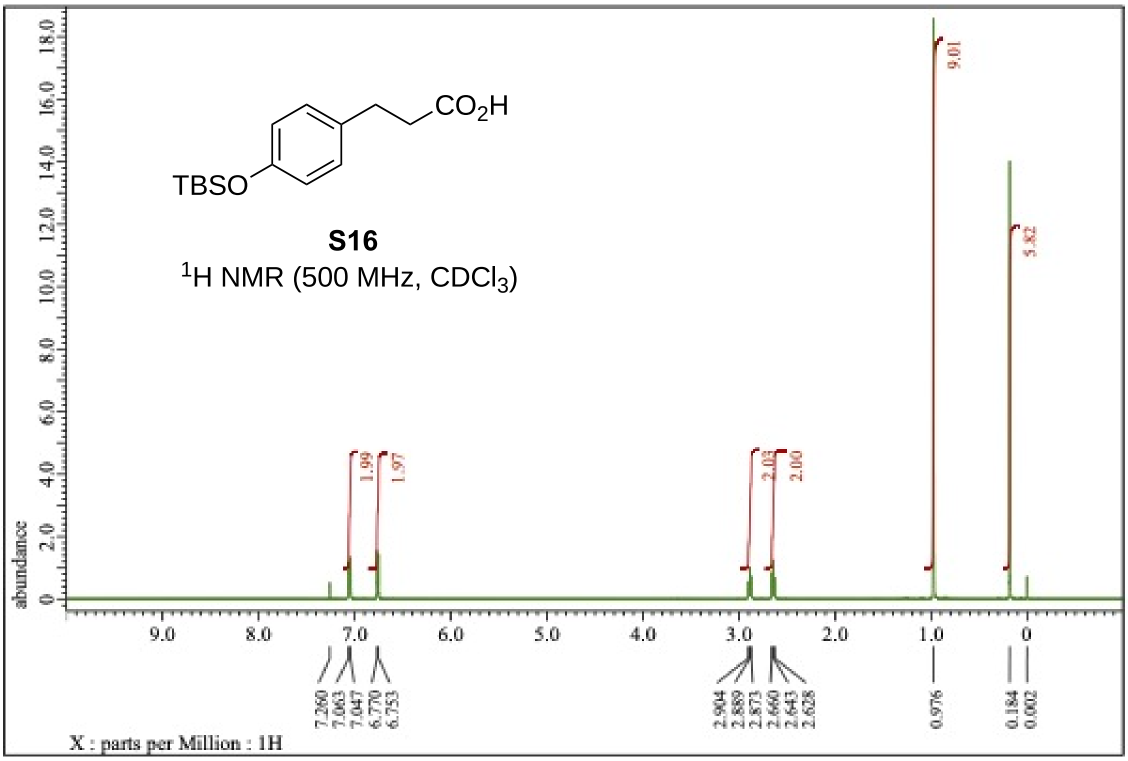

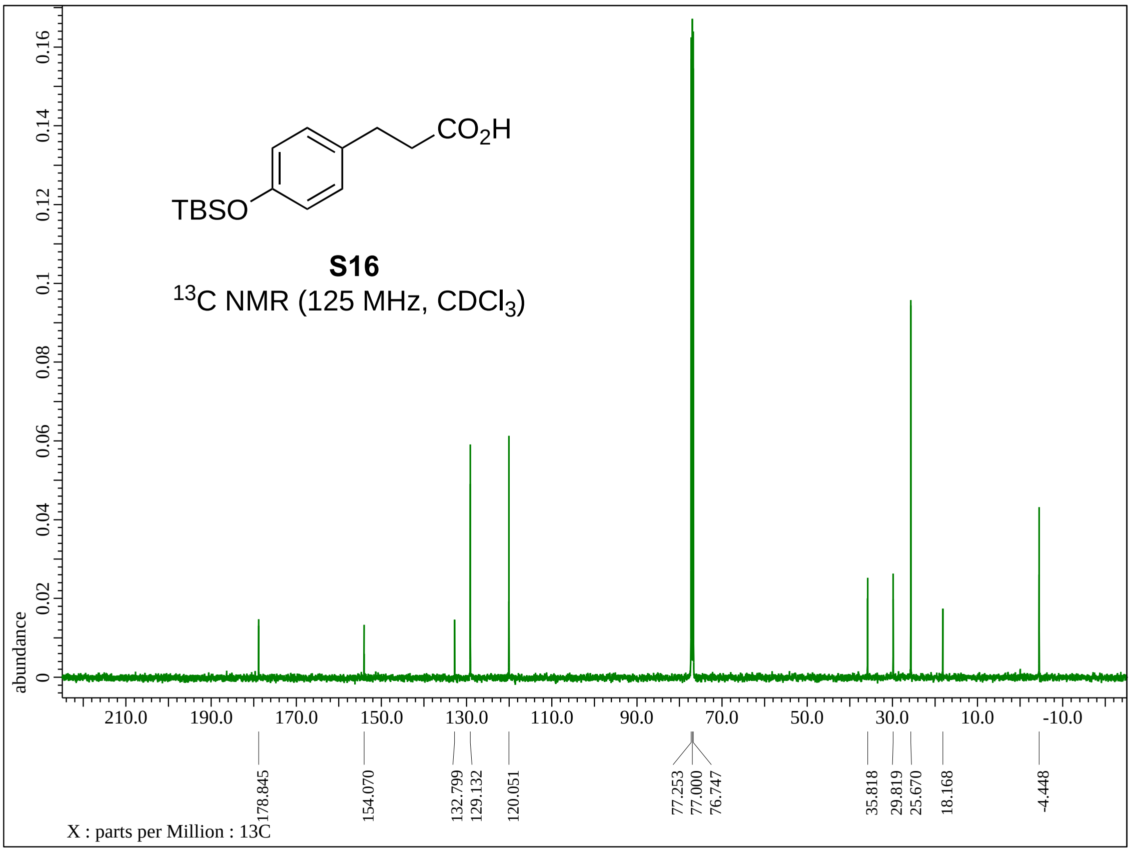

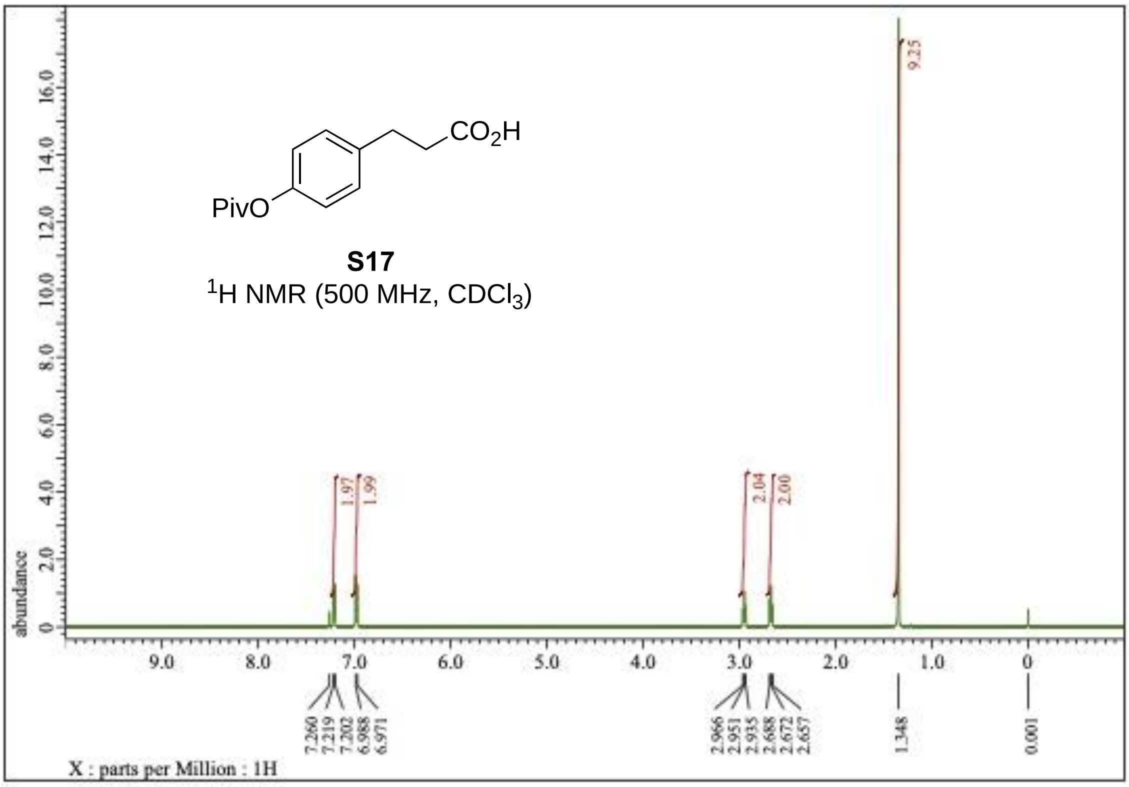

PivO
CO2H
S17
1H NMR (500 MHz, CDCl3)
abundance
X : parts per Million : 1H

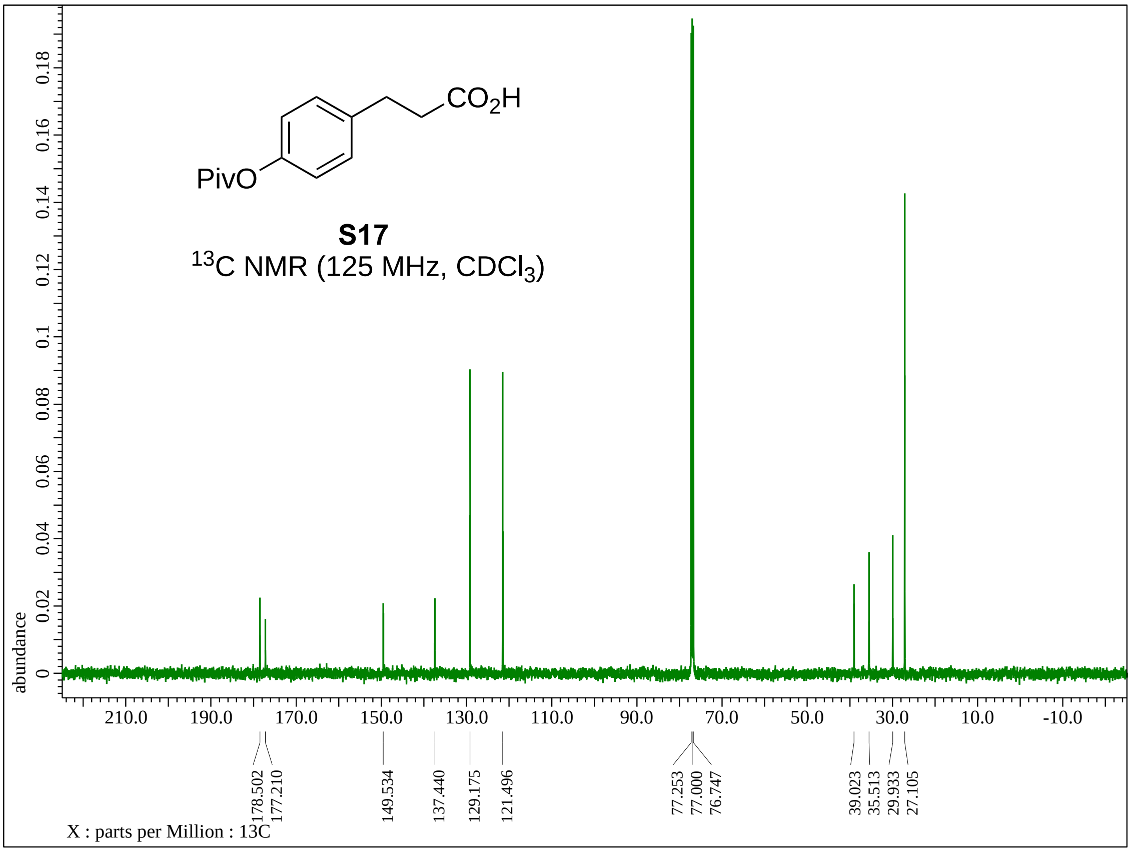

PivO
CO2H
S17
13C NMR (125 MHz, CDCl3)
abundance
X : parts per Million : 13C

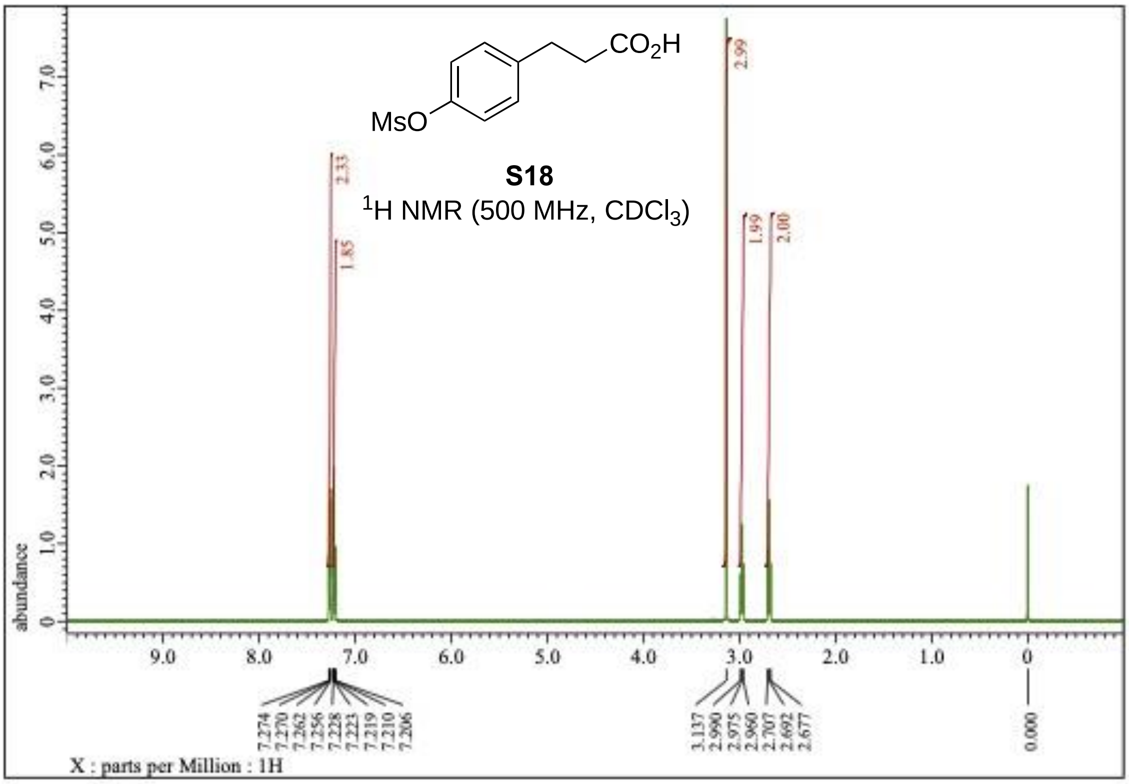
CO2H
MsO
S18
1H NMR (500 MHz, CDCl3)
abundance
X : parts per Million : 1H

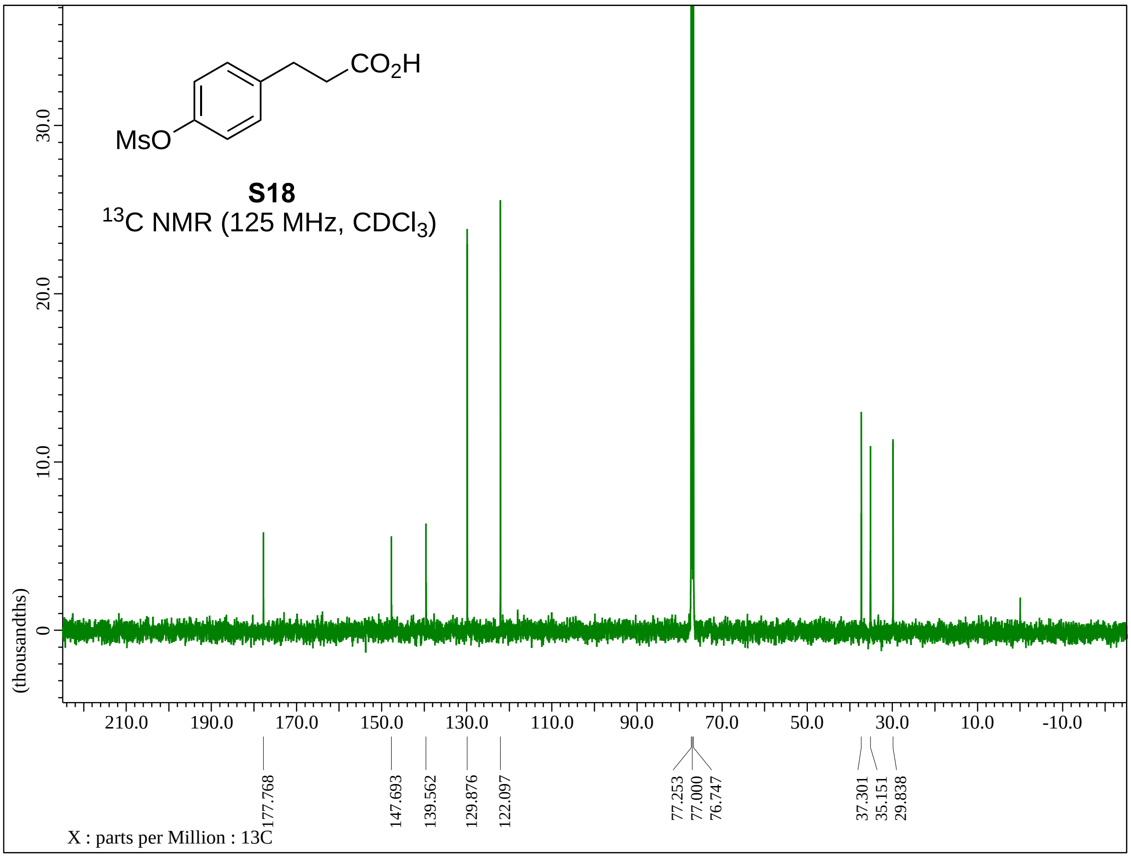
CO2H
MsO
S18
13C NMR (125 MHz, CDCl3)
(thousandths)
177.768
147.693
139.562
129.876
122.097
77.253
77.000
76.747
37.301
35.151
29.838
X : parts per Million : 13C

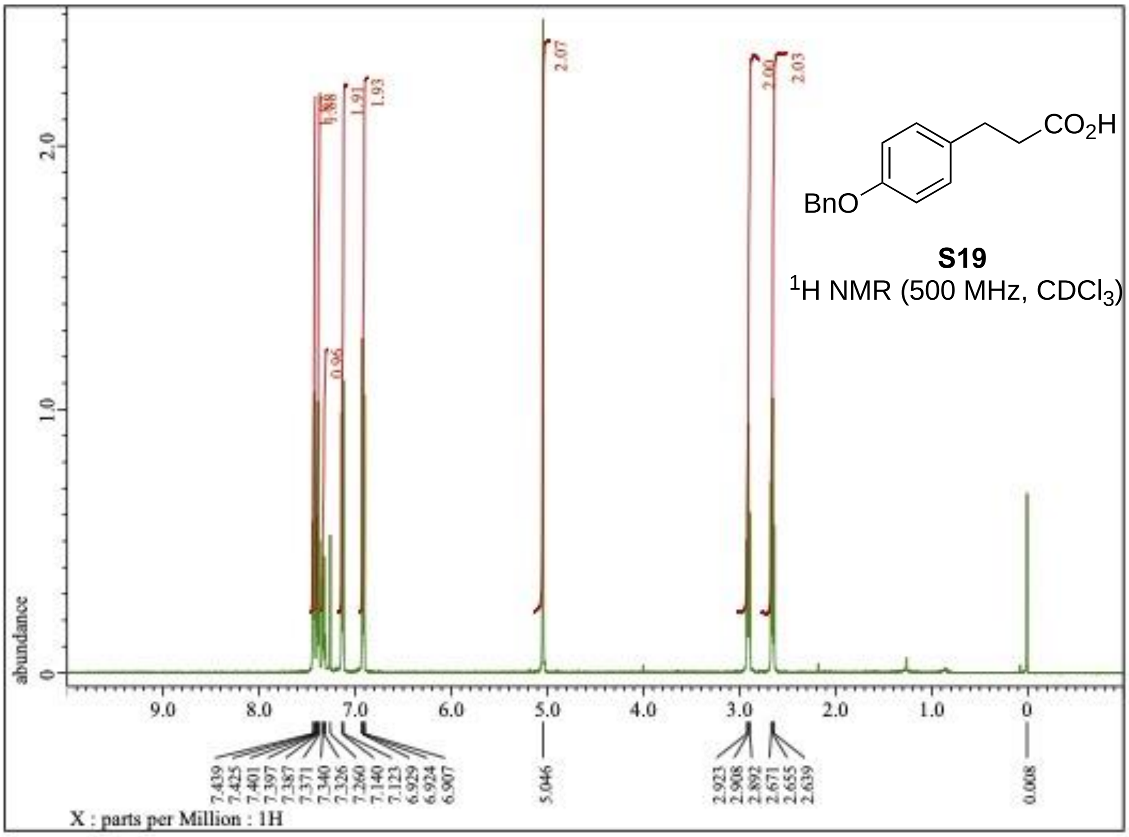
CO2H
BnO
S19
1H NMR (500 MHz, CDCl3)
abundance
X : parts per Million : 1H
7.439
7.425
7.401
7.397
7.387
7.371
7.340
7.326
7.260
7.140
7.123
6.929
6.924
6.907
5.046
2.923
2.908
2.892
2.671
2.655
2.639
0.008

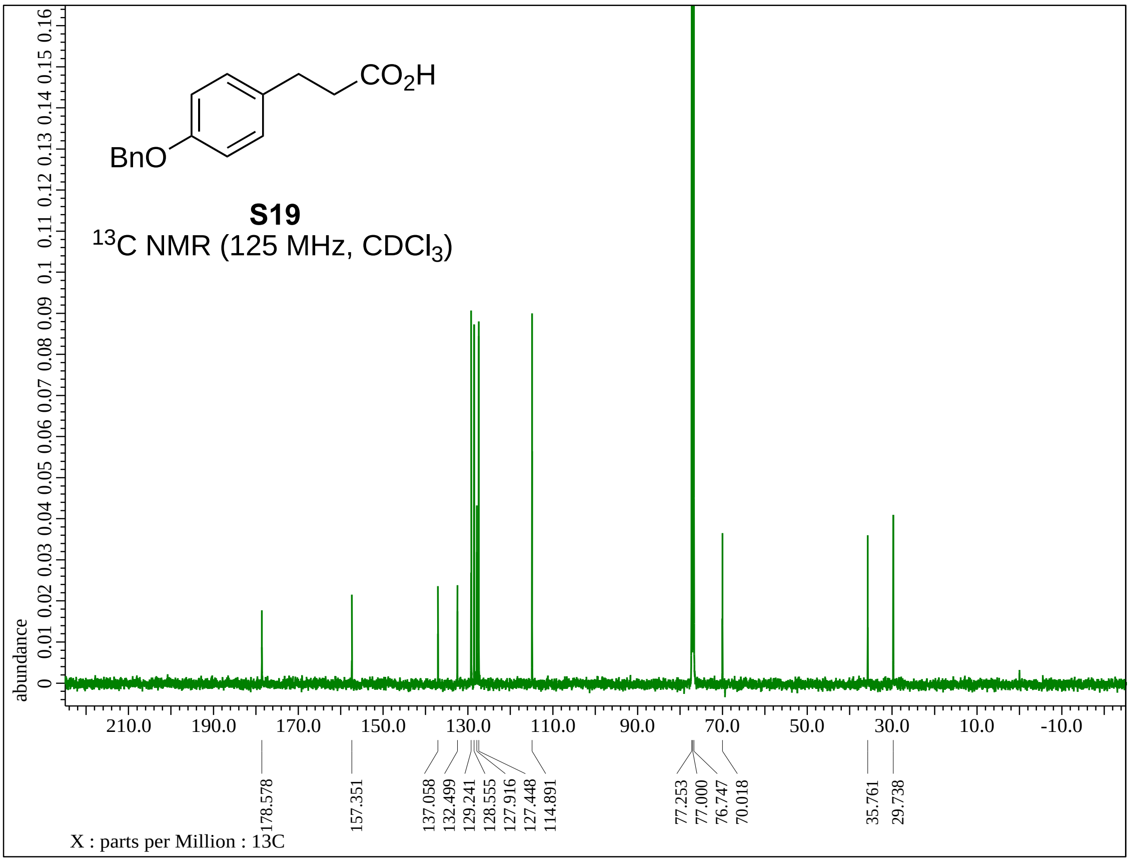
CO2H
BnO
S19
13C NMR (125 MHz, CDCl3)
abundance
X : parts per Million : 13C
178.578
157.351
137.058
132.499
129.241
128.555
127.916
127.448
114.891
77.253
77.000
76.747
70.018
35.761
29.738

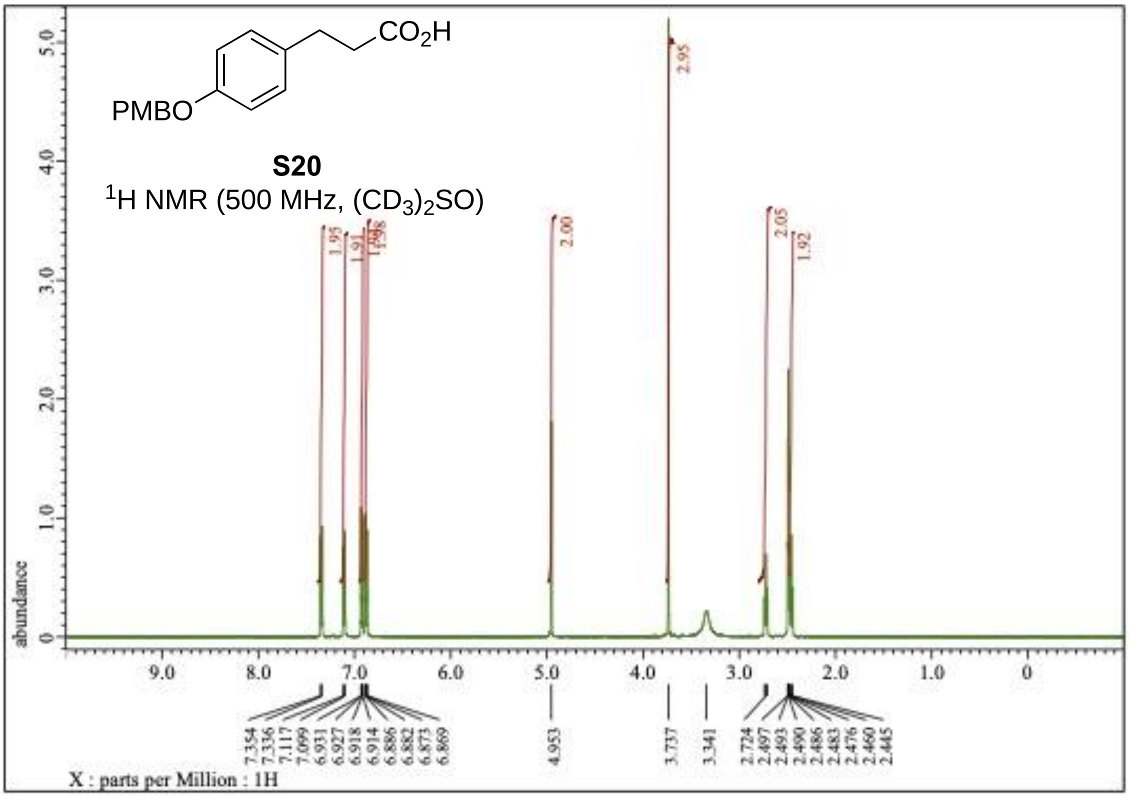
CO2H
PMBO
S20
1H NMR (500 MHz, (CD3)2SO)
abundance
X : parts per Million : 1H
7.354
7.336
7.117
7.099
6.931
6.927
6.918
6.914
6.886
6.882
6.873
6.869
4.953
3.737
3.341
2.724
2.497
2.493
2.490
2.486
2.483
2.476
2.460
2.445

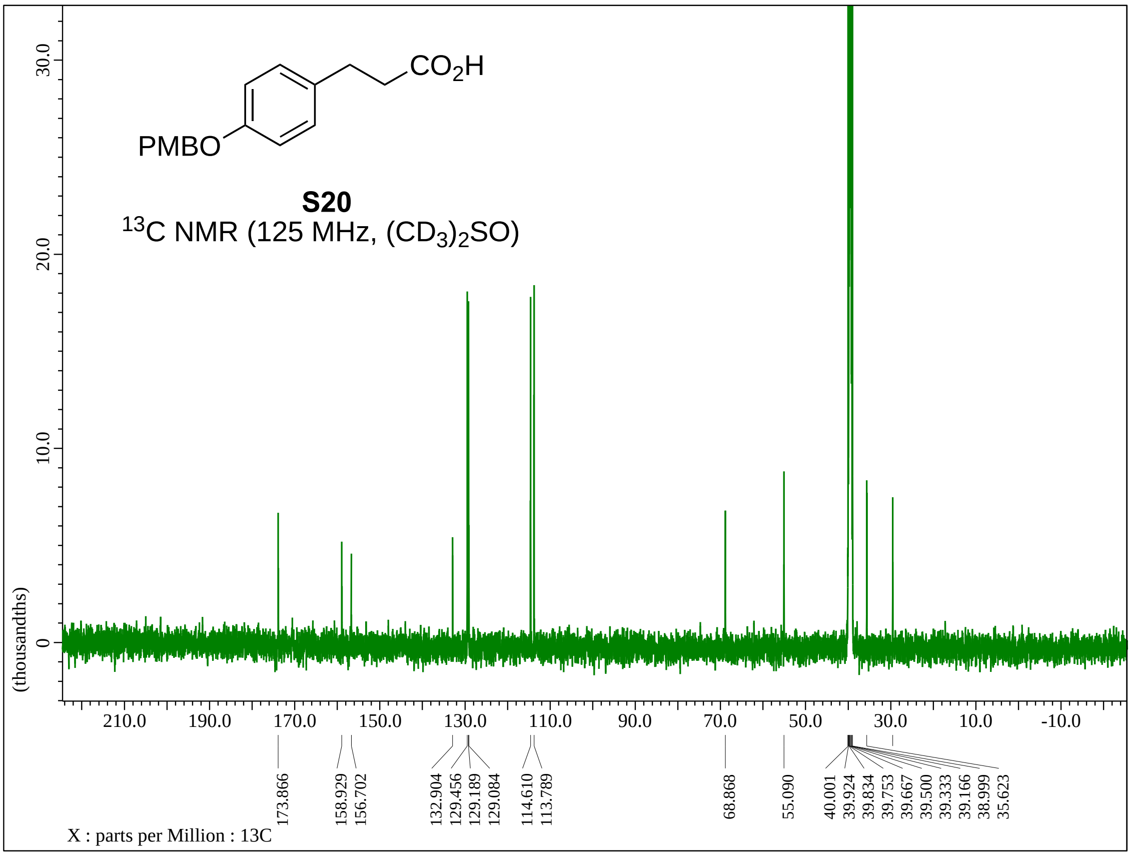
CO2H
PMBO
S20
13C NMR (125 MHz, (CD3)2SO)
(thousandths)
X : parts per Million : 13C
173.866
158.929
156.702
132.904
129.456
129.189
129.084
114.610
113.789
68.868
55.090
40.001
39.924
39.834
39.753
39.667
39.500
39.333
39.166
38.999
35.623

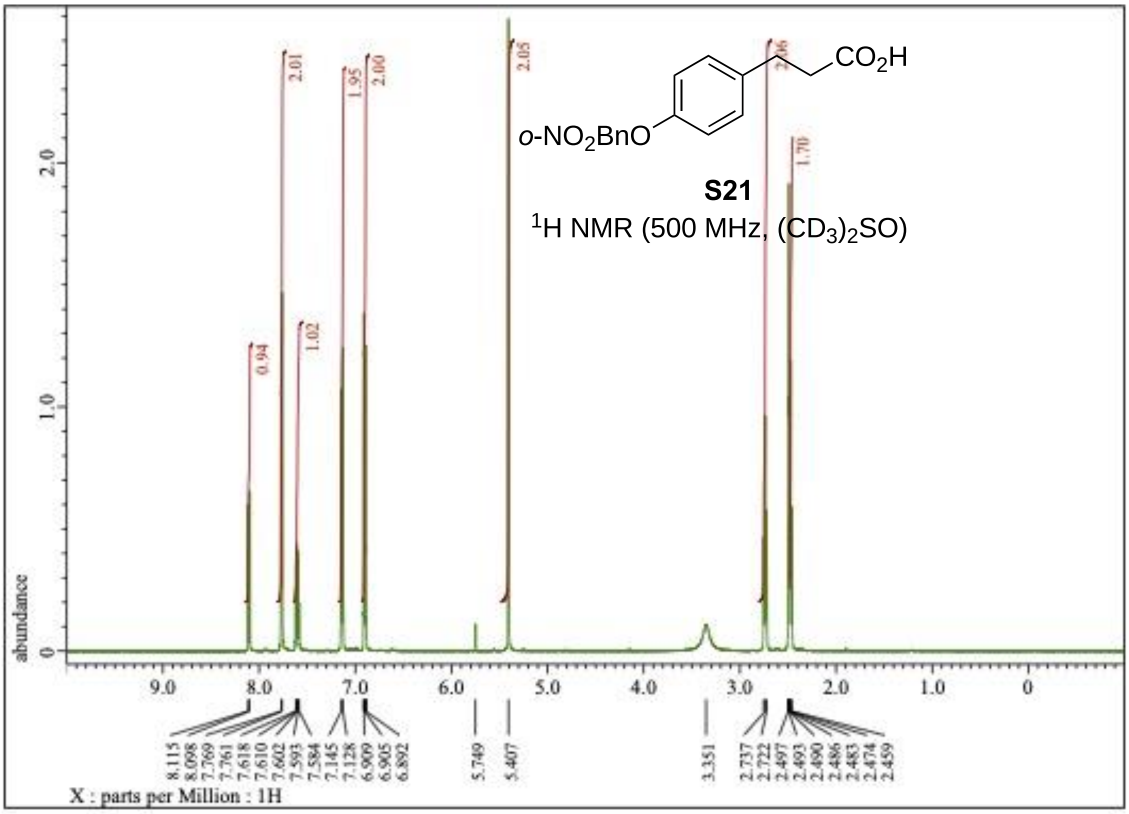
CO2H
o-NO2BnO
S21
1H NMR (500 MHz, (CD3)2SO)
X : parts per Million : 1H
abundance

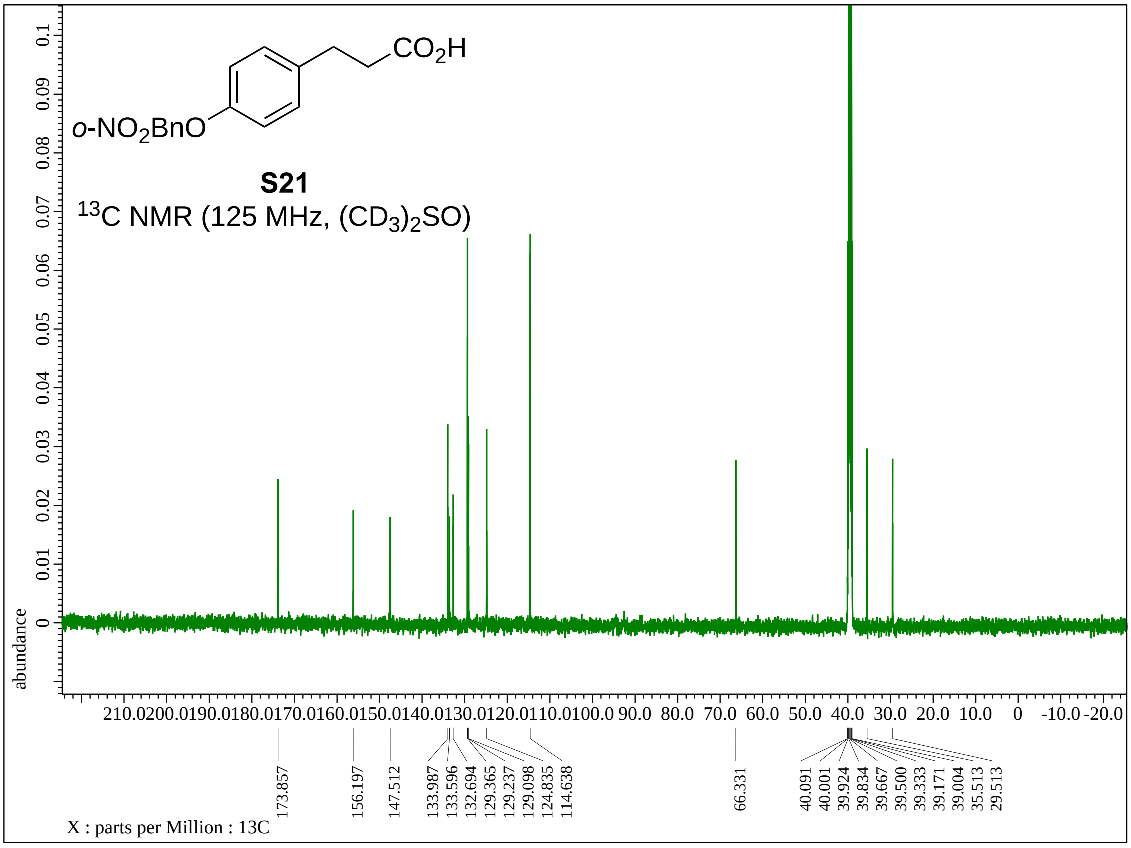
CO2H
o-NO2BnO
S21
13C NMR (125 MHz, (CD3)2SO)
X : parts per Million : 13C
abundance

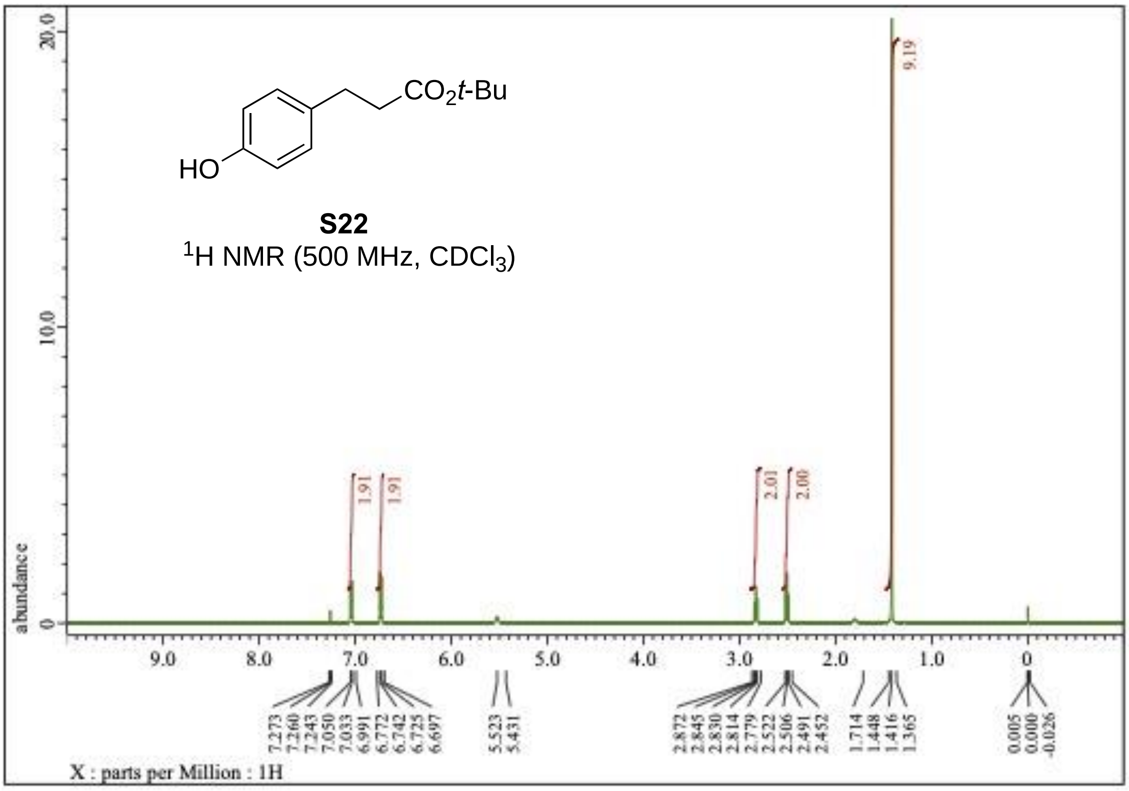
CO2t-Bu
HO
S22
1H NMR (500 MHz, CDCl3)
abundance
X : parts per Million : 1H
9.19
1.91
1.91
2.01
2.00

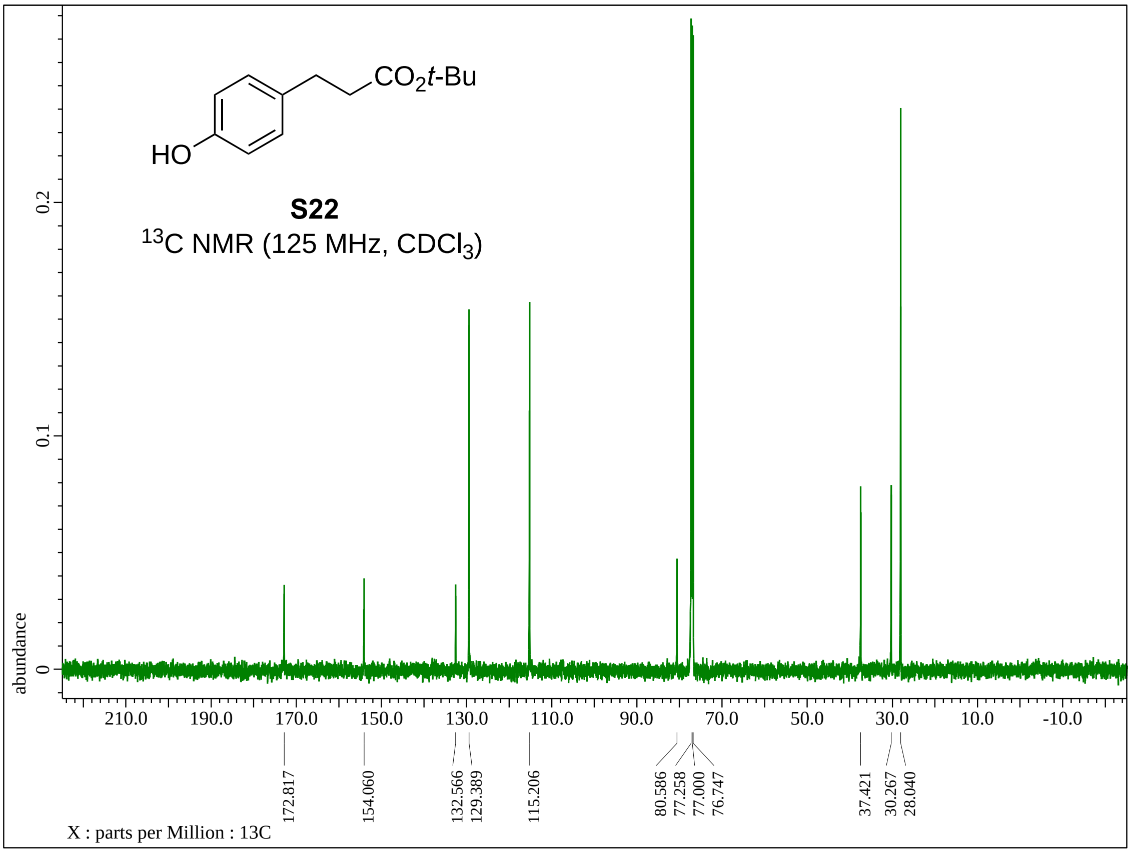
CO2t-Bu
HO
S22
13C NMR (125 MHz, CDCl3)
abundance
X : parts per Million : 13C
172.817
154.060
132.566
129.389
115.206
80.586
77.258
77.000
76.747
37.421
30.267
28.040

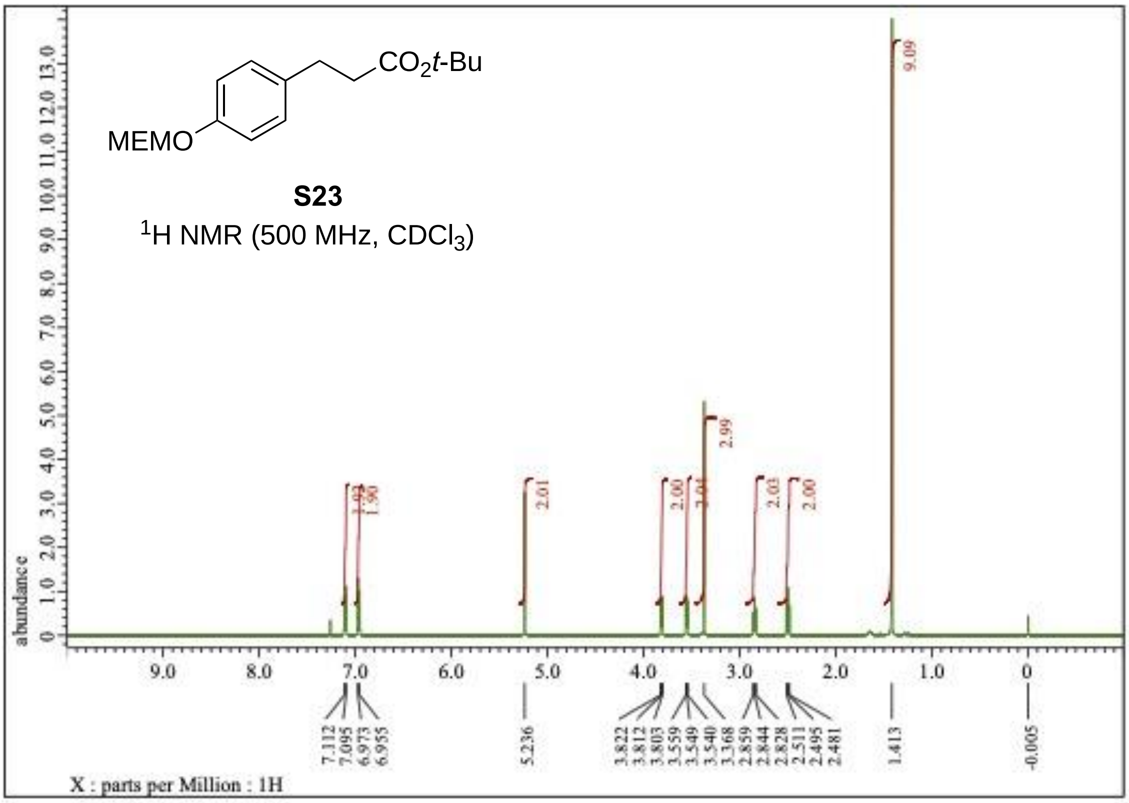
CO2t-Bu
MEMO
S23
1H NMR (500 MHz, CDCl3)
abundance
9.09
2.99
2.01
2.00
2.03
2.00
1.90
7.112
7.095
6.973
6.955
5.236
3.822
3.812
3.803
3.559
3.549
3.540
3.368
2.859
2.844
2.828
2.511
2.495
2.481
1.413
-0.005
X : parts per Million : 1H

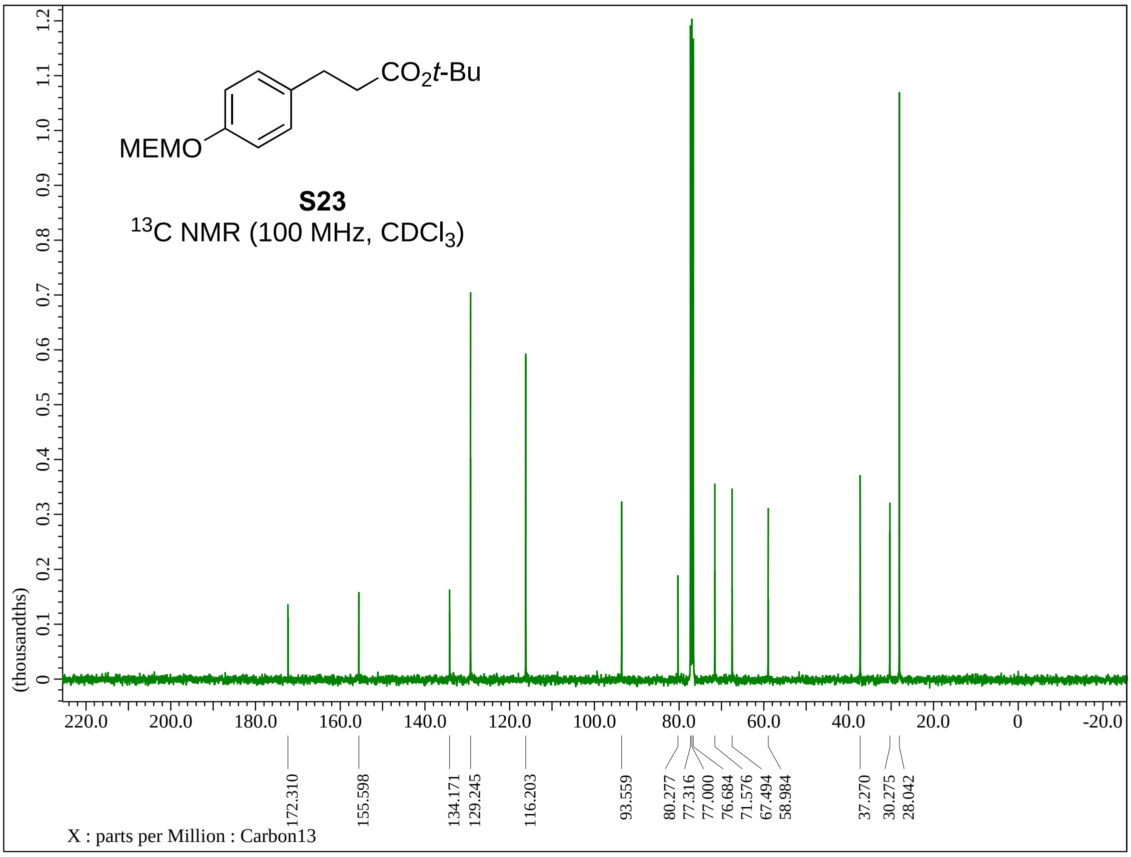
CO2t-Bu
MEMO
S23
13C NMR (100 MHz, CDCl3)
(thousandths)
172.310
155.598
134.171
129.245
116.203
93.559
80.277
77.316
77.000
76.684
71.576
67.494
58.984
37.270
30.275
28.042
X : parts per Million : Carbon13

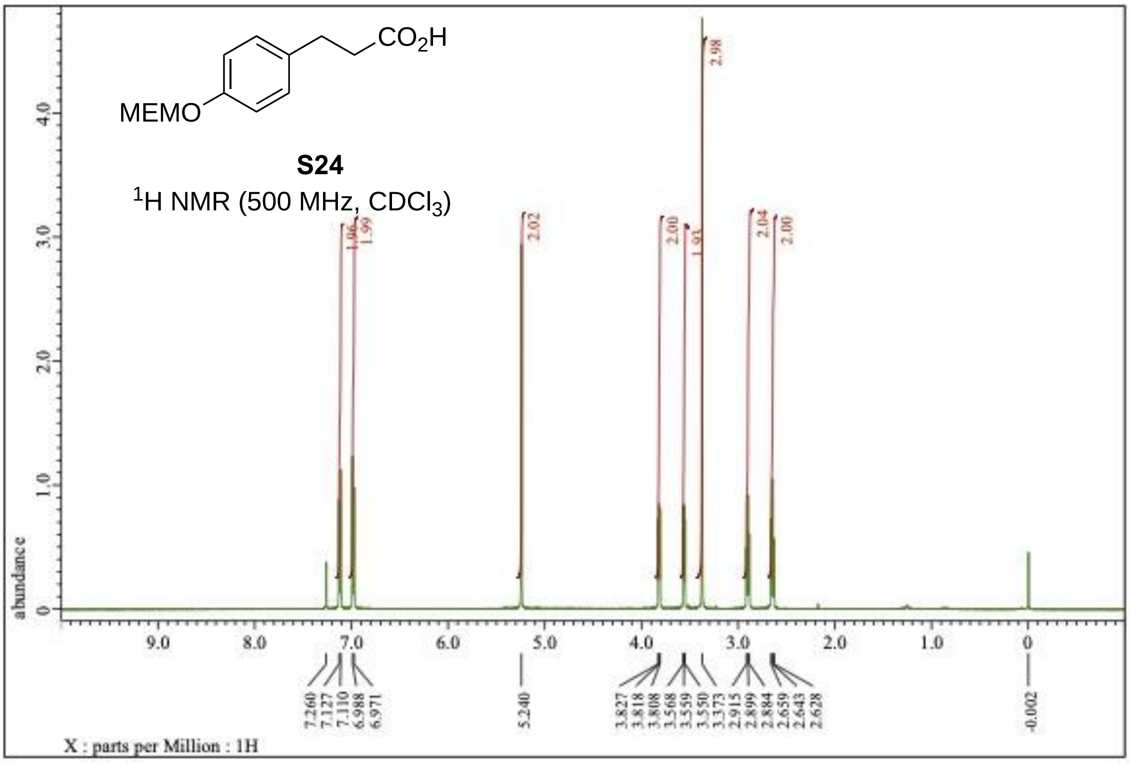

CO2H
MEMO
S24
1H NMR (500 MHz, CDCl3)
abundance
X : parts per Million : 1H
7.260
7.127
7.110
6.988
6.971
5.240
3.827
3.818
3.808
3.568
3.559
3.550
3.373
2.915
2.899
2.884
2.659
2.643
2.628
-0.002
1.96
1.99
2.02
2.00
1.93
2.98
2.04
2.00

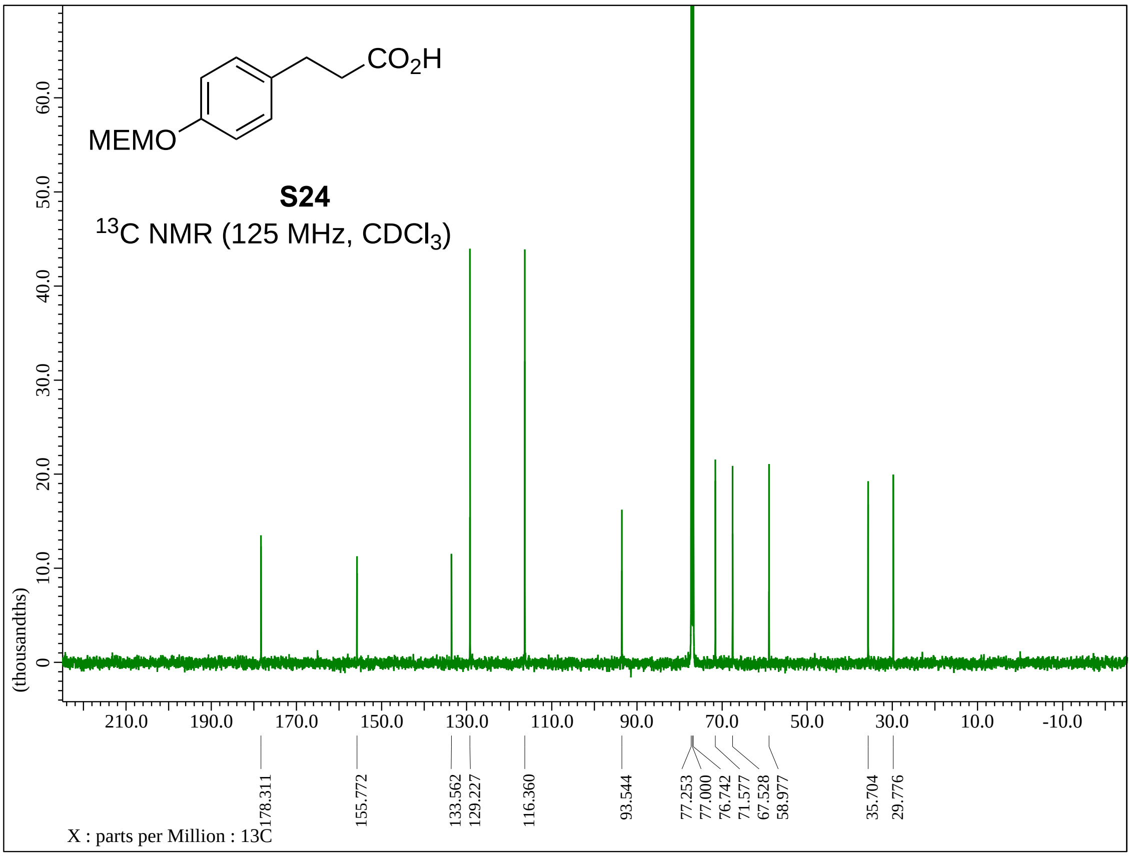

CO2H
MEMO
S24
13C NMR (125 MHz, CDCl3)
(thousandths)
X : parts per Million : 13C
178.311
155.772
133.562
129.227
116.360
93.544
77.253
77.000
76.742
71.577
67.528
58.977
35.704
29.776

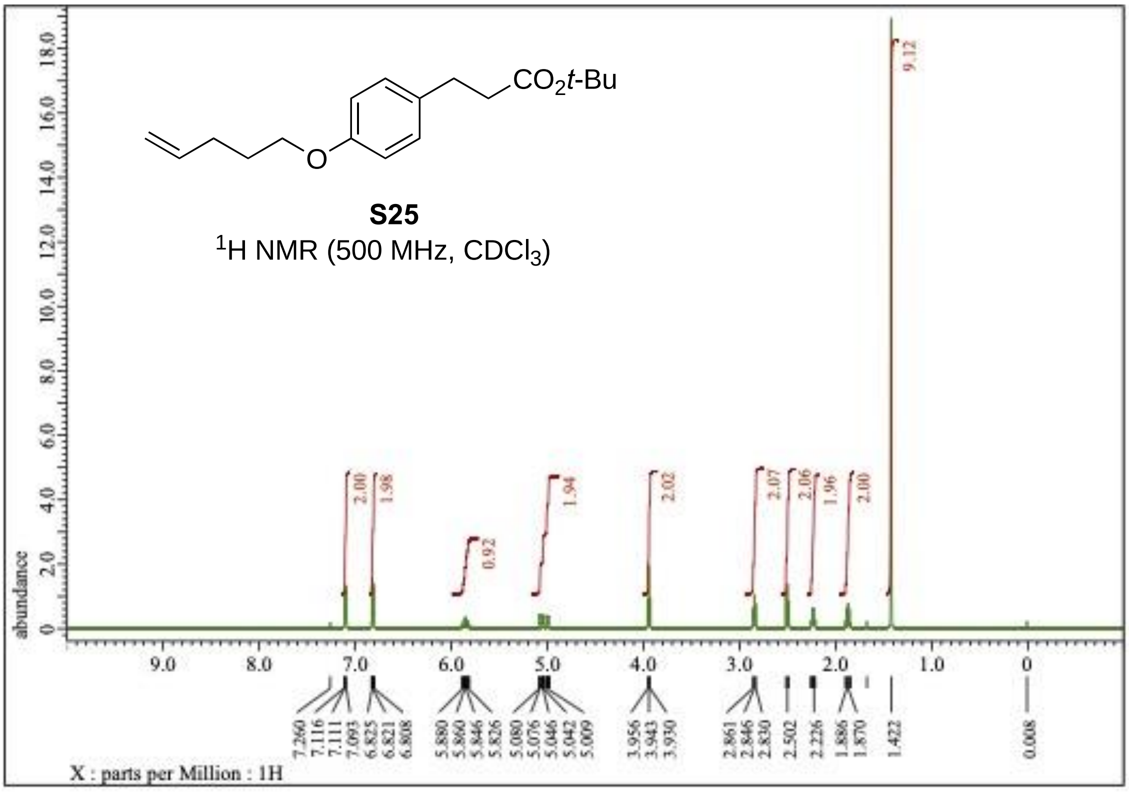
CO2t-Bu
O
S25
1H NMR (500 MHz, CDCl3)
abundance
X : parts per Million : 1H
2.00
1.98
0.92
1.94
2.02
2.07
2.06
1.96
2.00
9.12
7.260
7.116
7.111
7.093
6.825
6.821
6.808
5.880
5.860
5.846
5.826
5.080
5.076
5.046
5.042
5.009
3.956
3.943
3.930
2.861
2.846
2.830
2.502
2.226
1.886
1.870
1.422
0.008

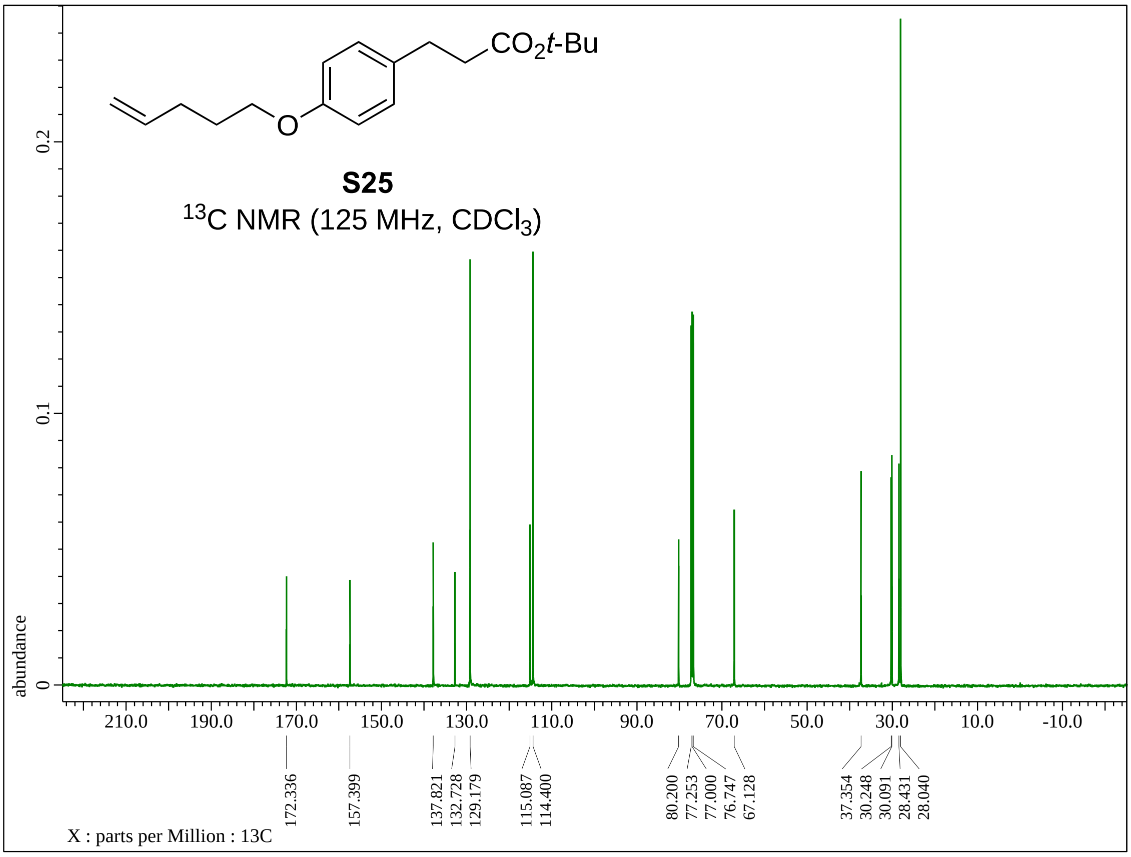
CO2t-Bu
O
S25
13C NMR (125 MHz, CDCl3)
abundance
X : parts per Million : 13C
172.336
157.399
137.821
132.728
129.179
115.087
114.400
80.200
77.253
77.000
76.747
67.128
37.354
30.248
30.091
28.431
28.040

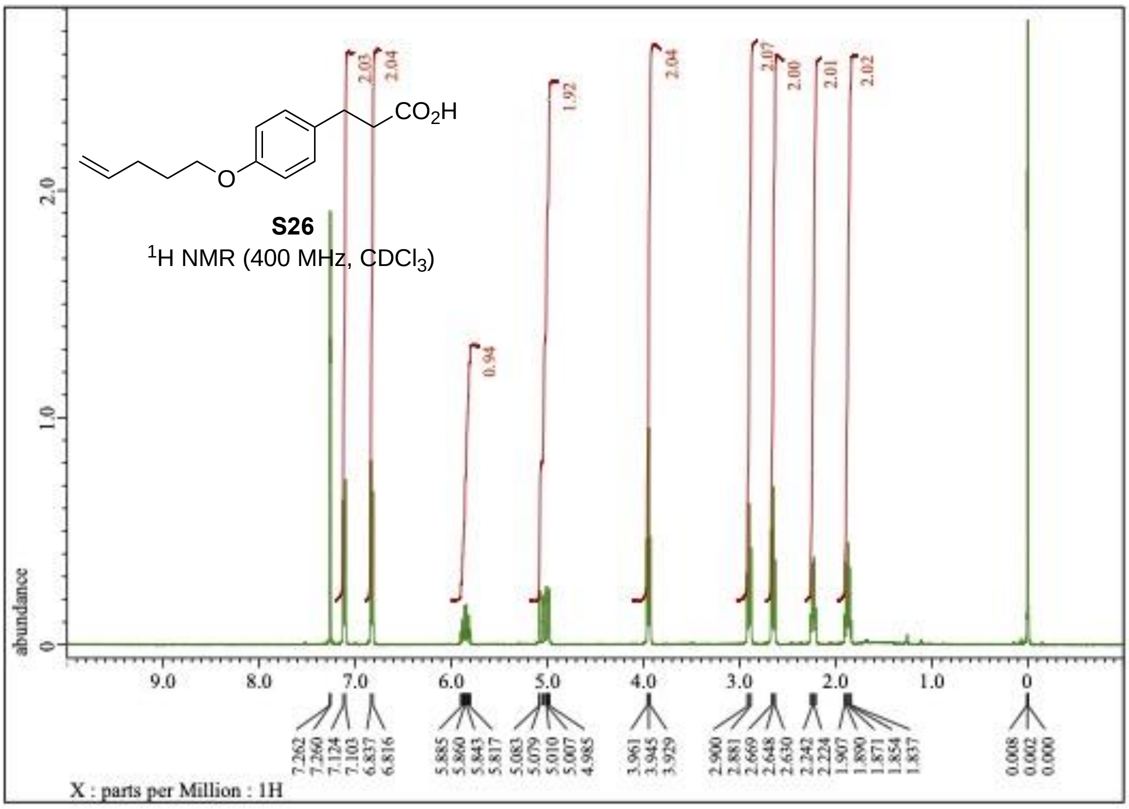
CO2H
O
S26
1H NMR (400 MHz, CDCl3)
abundance
X : parts per Million : 1H

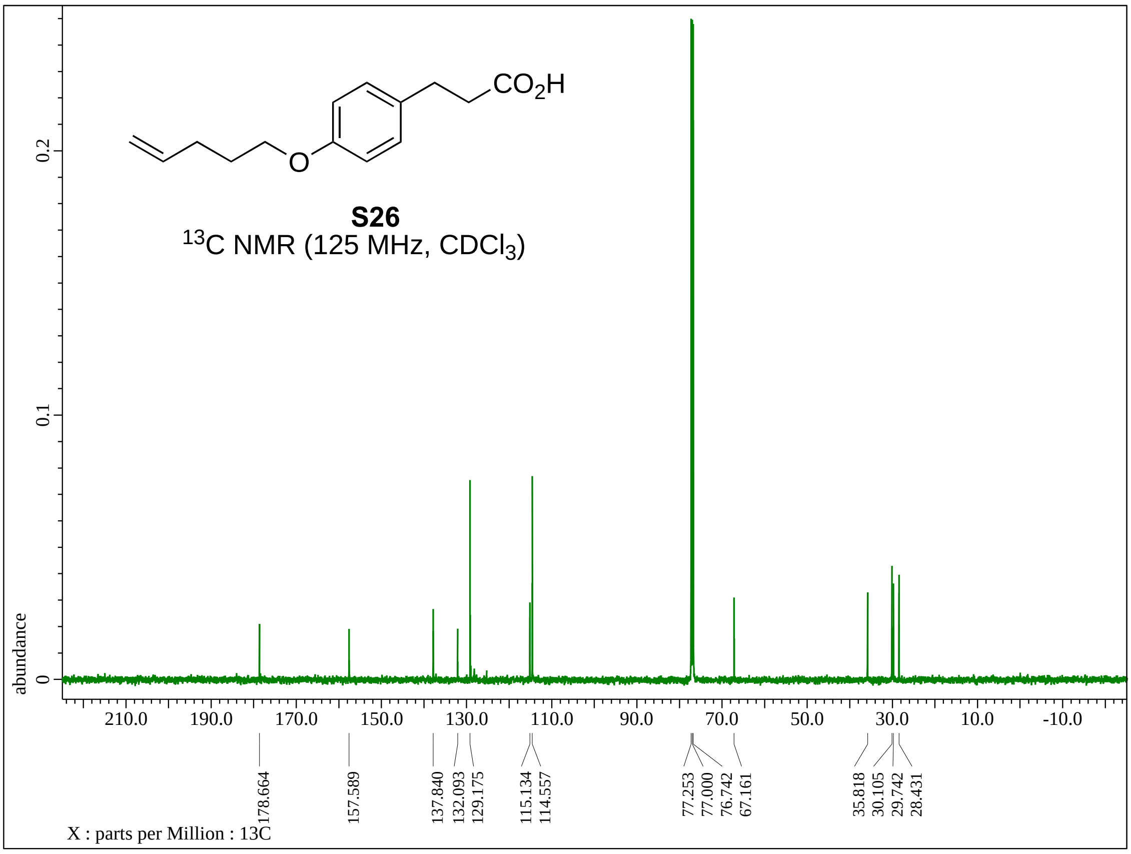
CO2H
O
S26
13C NMR (125 MHz, CDCl3)
abundance
178.664
157.589
137.840
132.093
129.175
115.134
114.557
77.253
77.000
76.742
67.161
35.818
30.105
29.742
28.431
X : parts per Million : 13C

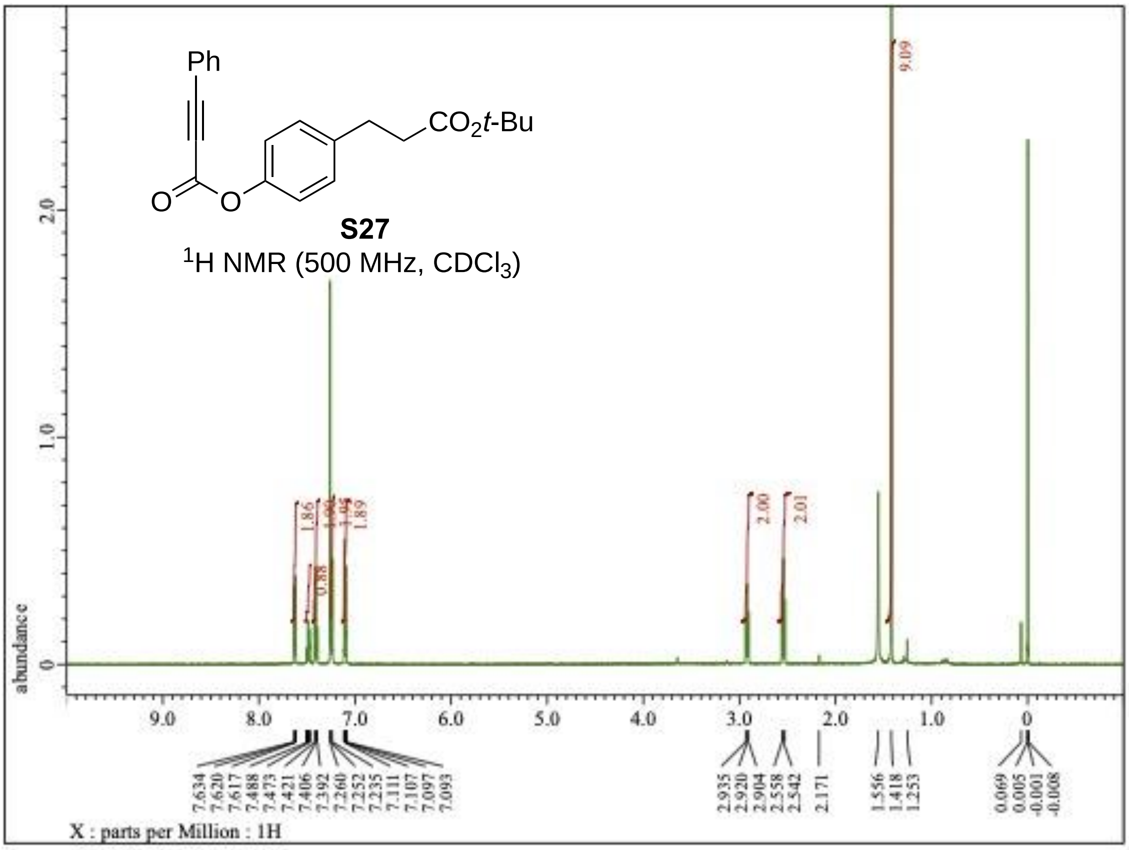
Ph
CO_2t-Bu
O
O
S27
^1H NMR (500 MHz, $CDCl_3$)
X : parts per Million : 1H
abundance

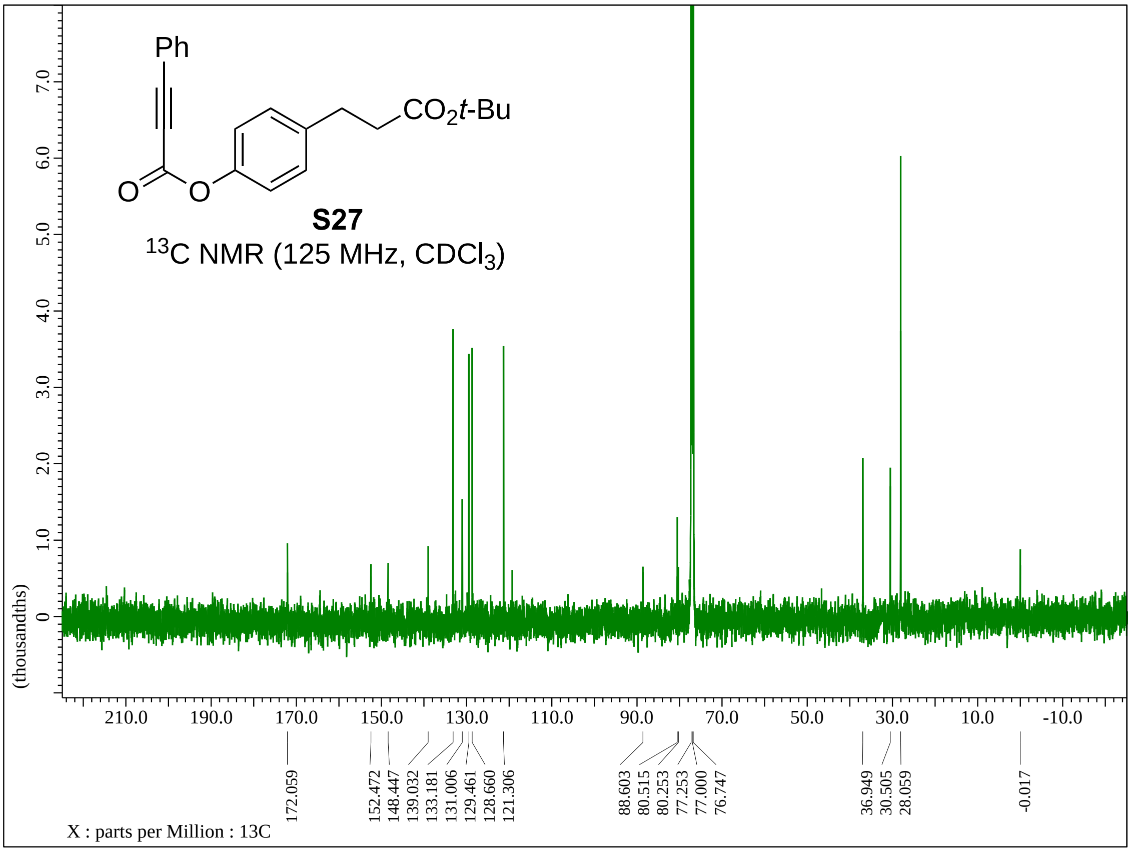
Ph
CO_2t-Bu
O
O
S27
^{13}C NMR (125 MHz, $CDCl_3$)
X : parts per Million : 13C
(thousandths)

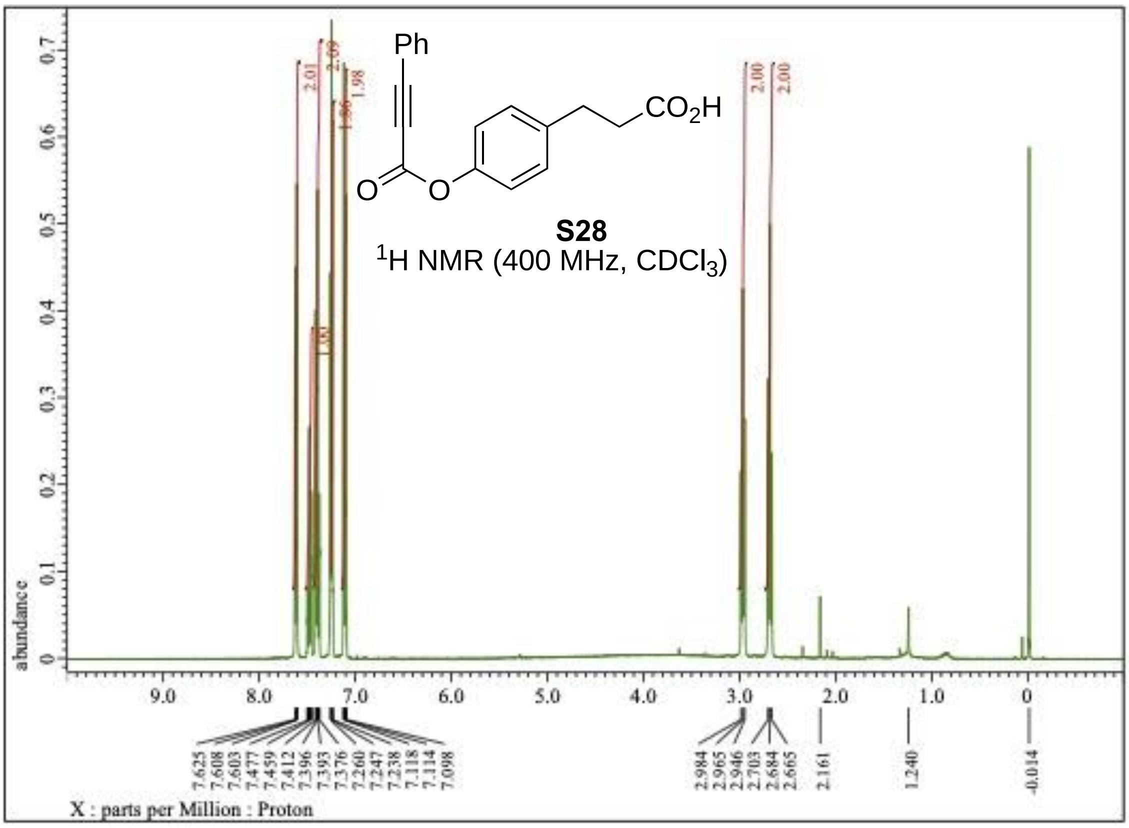
Ph
CO2H
O
O
S28
1H NMR (400 MHz, CDCl3)
abundance
X : parts per Million : Proton
7.625
7.608
7.603
7.477
7.459
7.412
7.396
7.393
7.376
7.260
7.247
7.238
7.118
7.114
7.098
2.984
2.965
2.946
2.703
2.684
2.665
2.161
1.240
-0.014

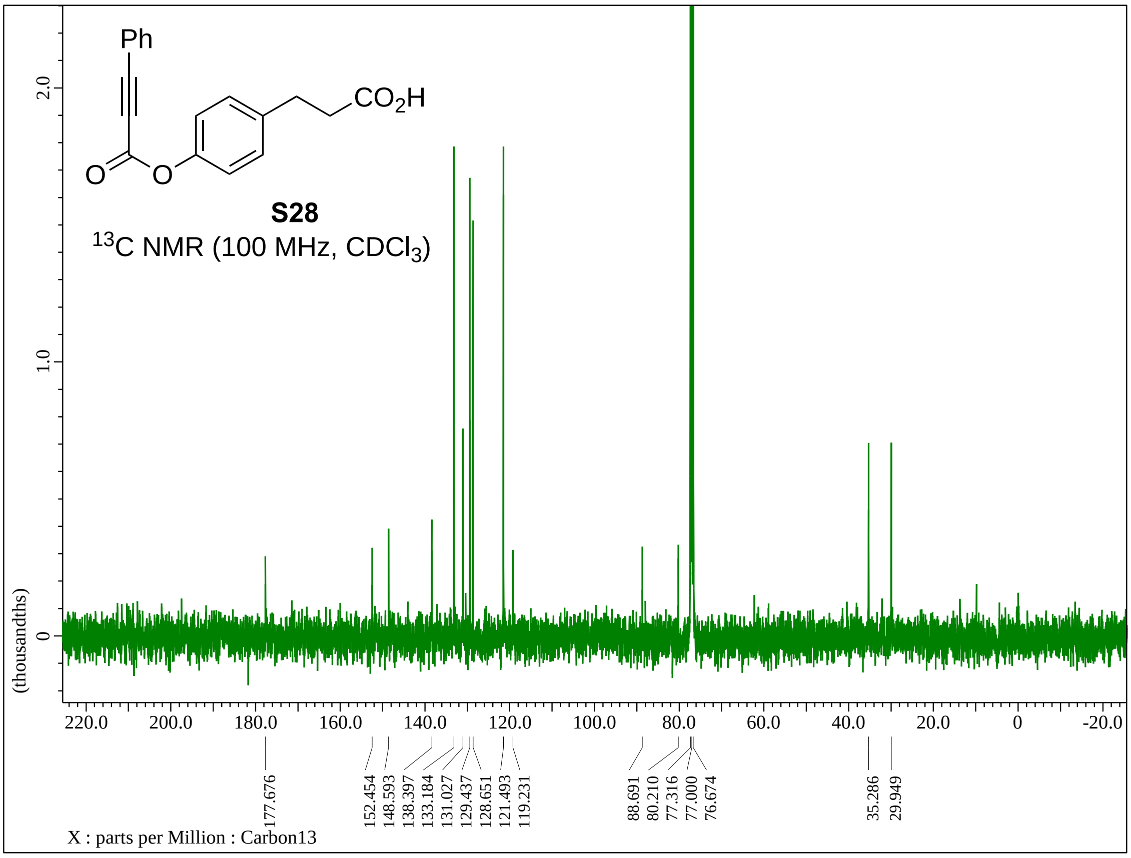
Ph
CO2H
O
O
S28
13C NMR (100 MHz, CDCl3)
(thousandths)
X : parts per Million : Carbon13
177.676
152.454
148.593
138.397
133.184
131.027
129.437
128.651
121.493
119.231
88.691
80.210
77.316
77.000
76.674
35.286
29.949

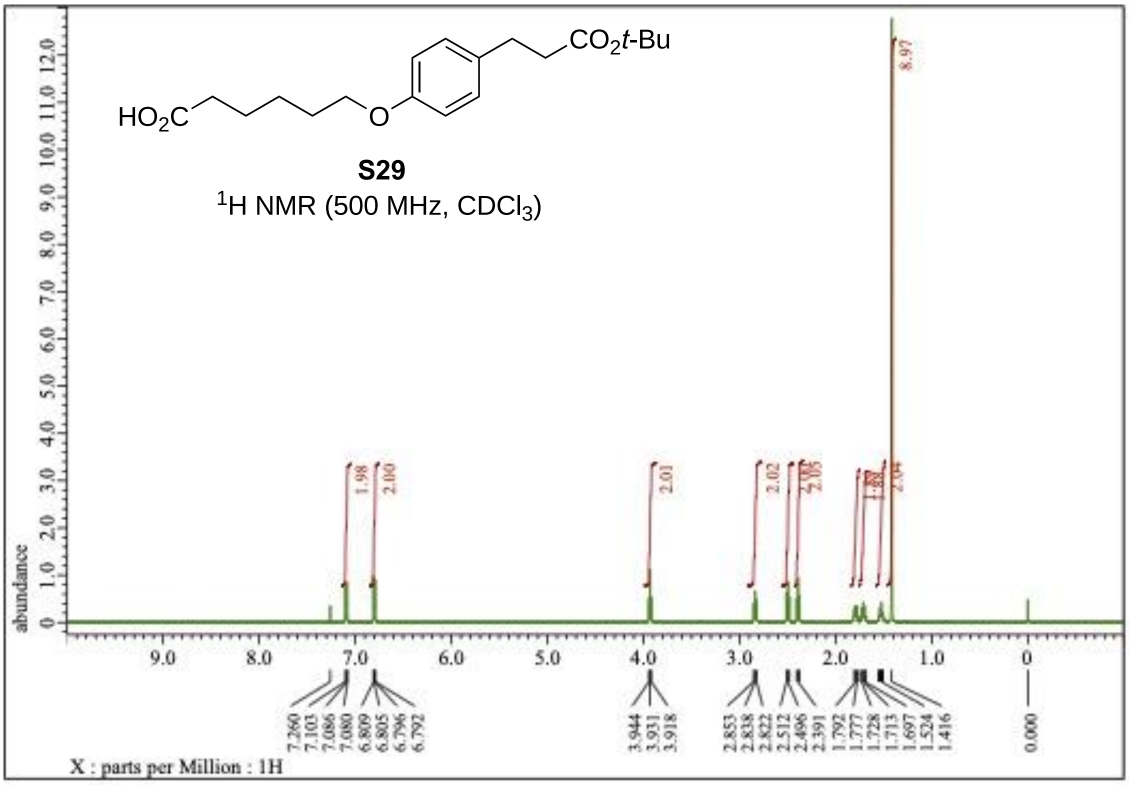
CO2t-Bu
HO2C
O
S29
1H NMR (500 MHz, CDCl3)
abundance
X : parts per Million : 1H

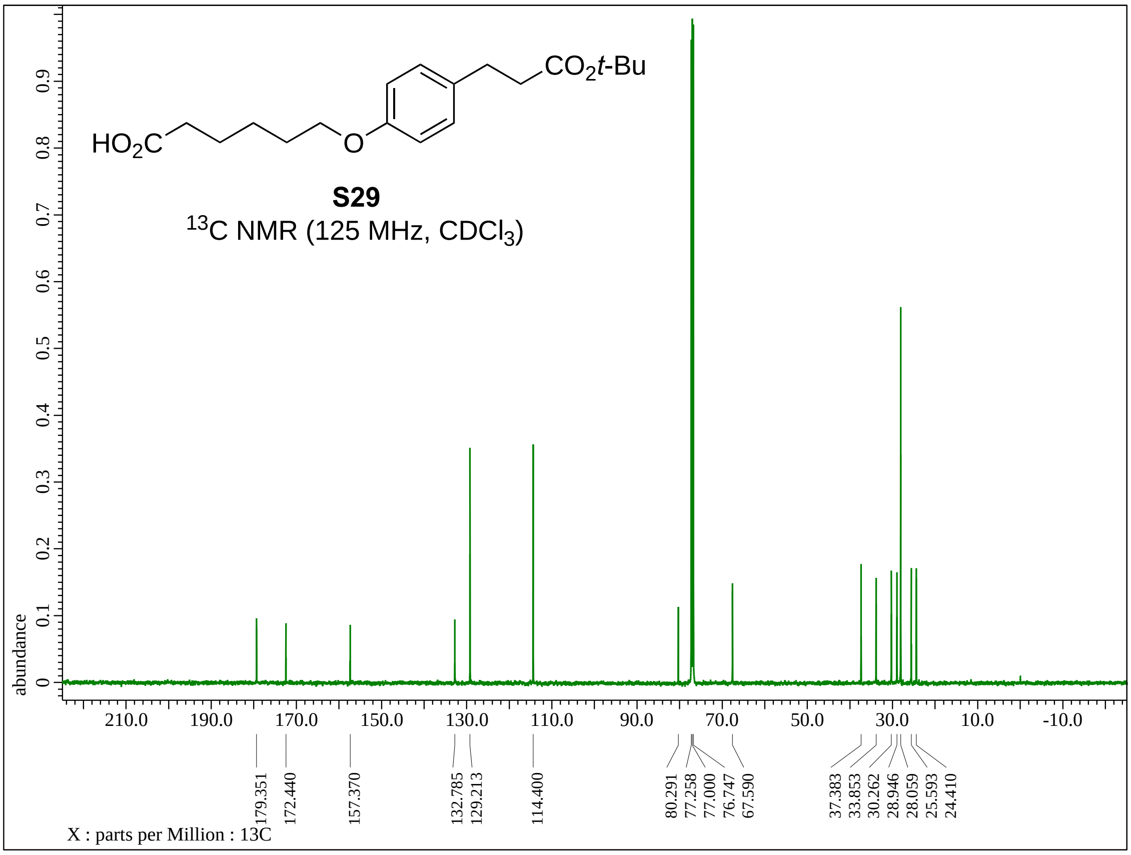
CO2t-Bu
HO2C
O
S29
13C NMR (125 MHz, CDCl3)
abundance
179.351
172.440
157.370
132.785
129.213
114.400
80.291
77.258
77.000
76.747
67.590
37.383
33.853
30.262
28.946
28.059
25.593
24.410
X : parts per Million : 13C

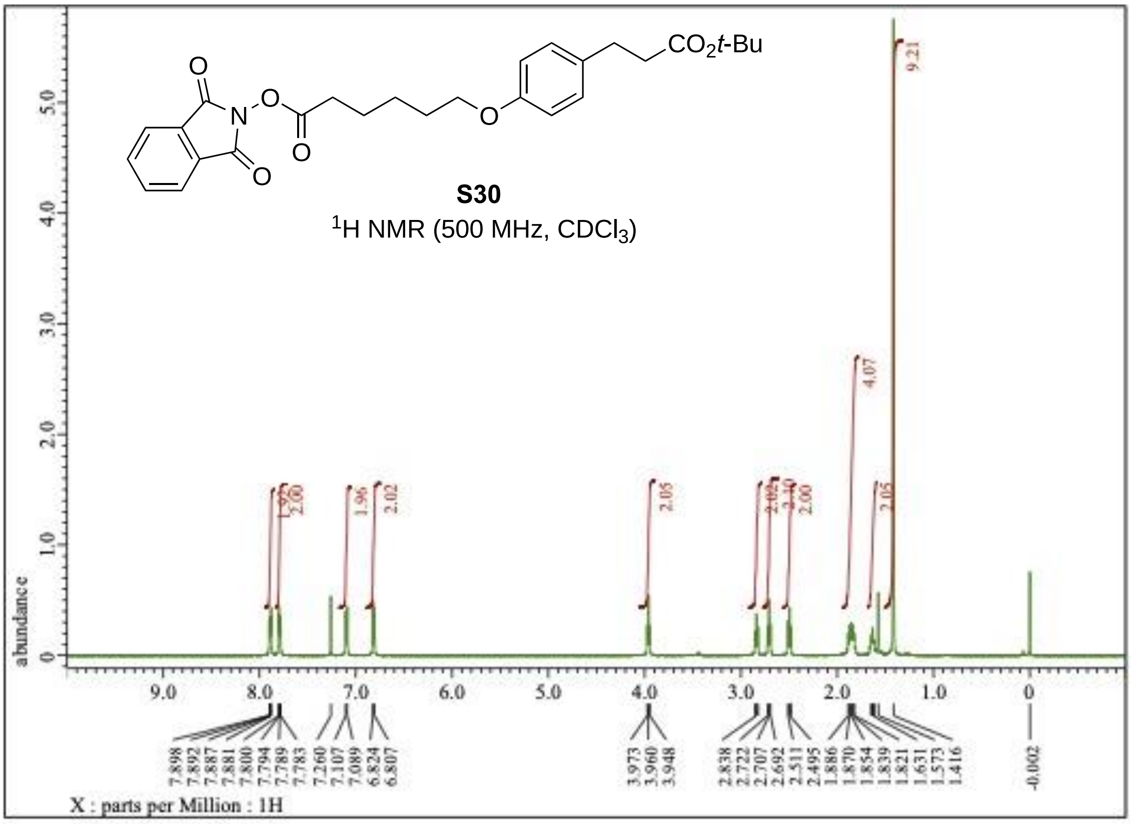
S30
^{1}H NMR (500 MHz, CDCl$_3$)
X : parts per Million : 1H
abundance

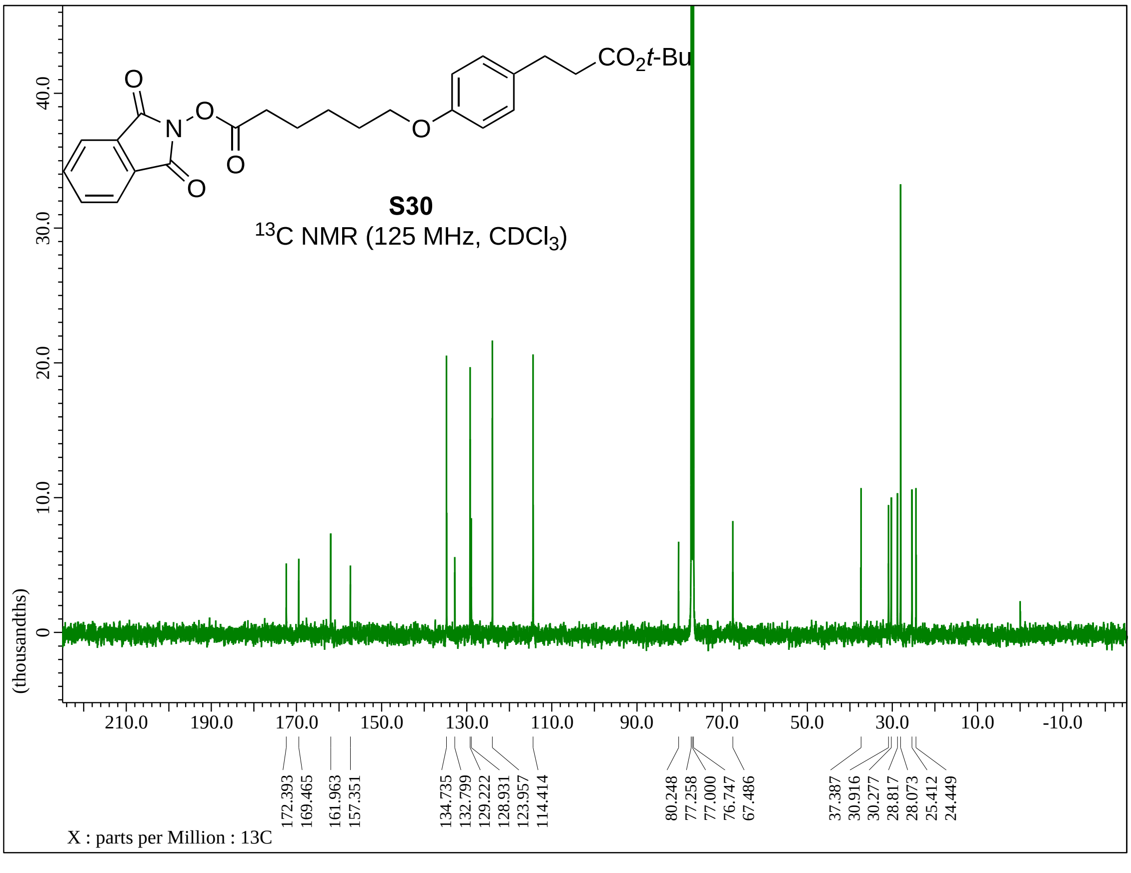
S30
^{13}C NMR (125 MHz, CDCl$_3$)
X : parts per Million : 13C
(thousandths)

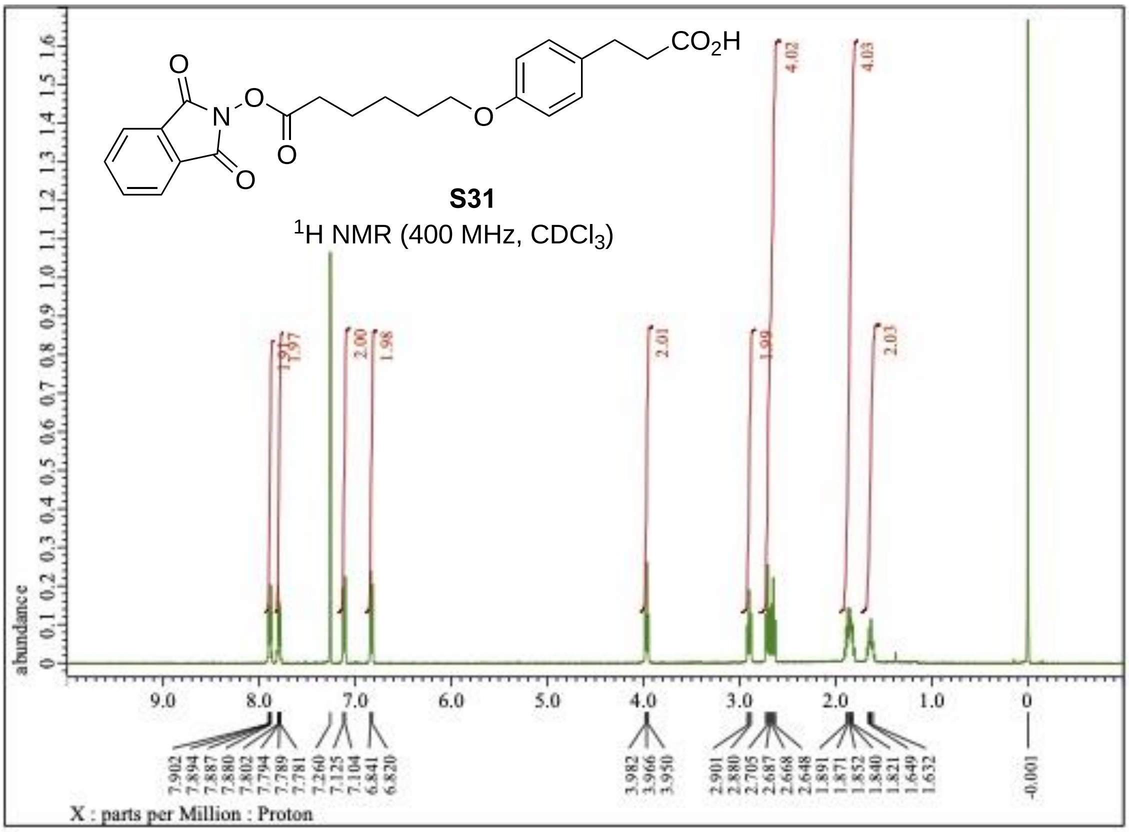

CO2H
S31
1H NMR (400 MHz, CDCl3)
abundance
X : parts per Million : Proton

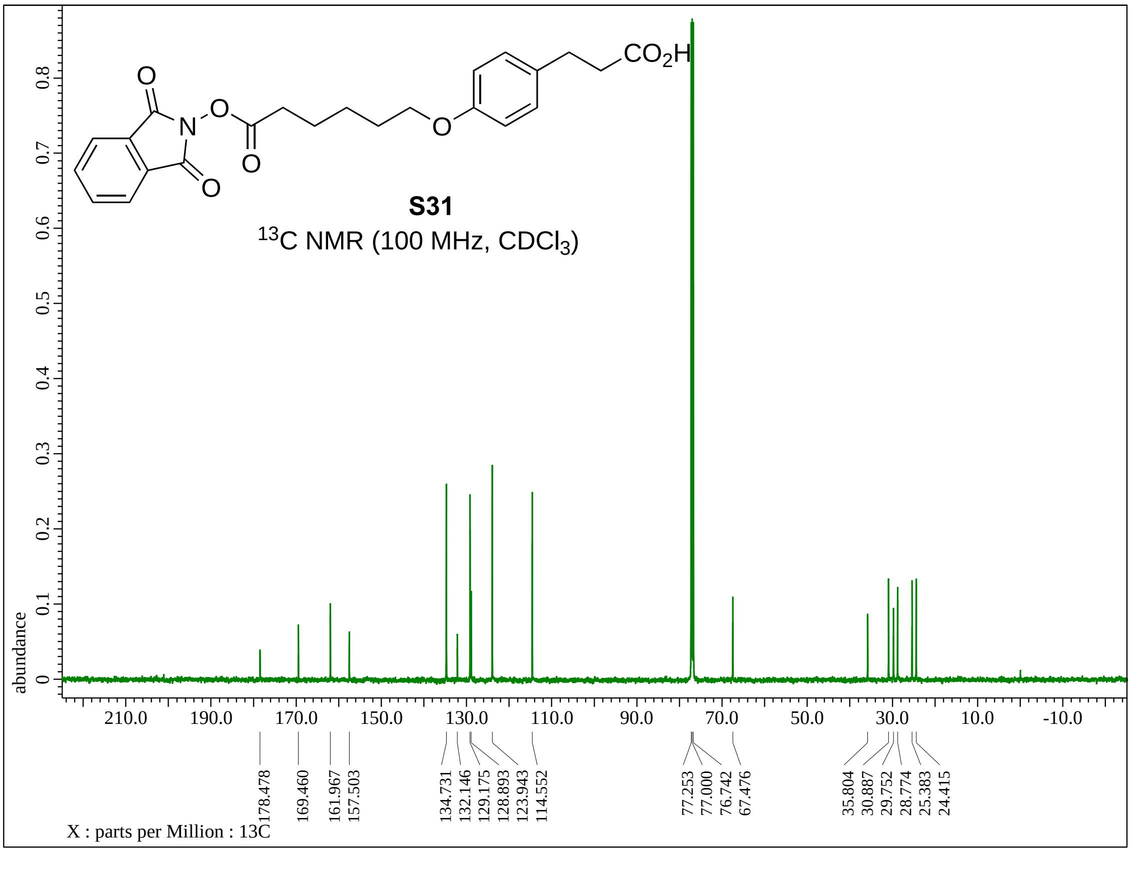

CO2H
S31
13C NMR (100 MHz, CDCl3)
abundance
178.478
169.460
161.967
157.503
134.731
132.146
129.175
128.893
123.943
114.552
77.253
77.000
76.742
67.476
35.804
30.887
29.752
28.774
25.383
24.415
X : parts per Million : 13C

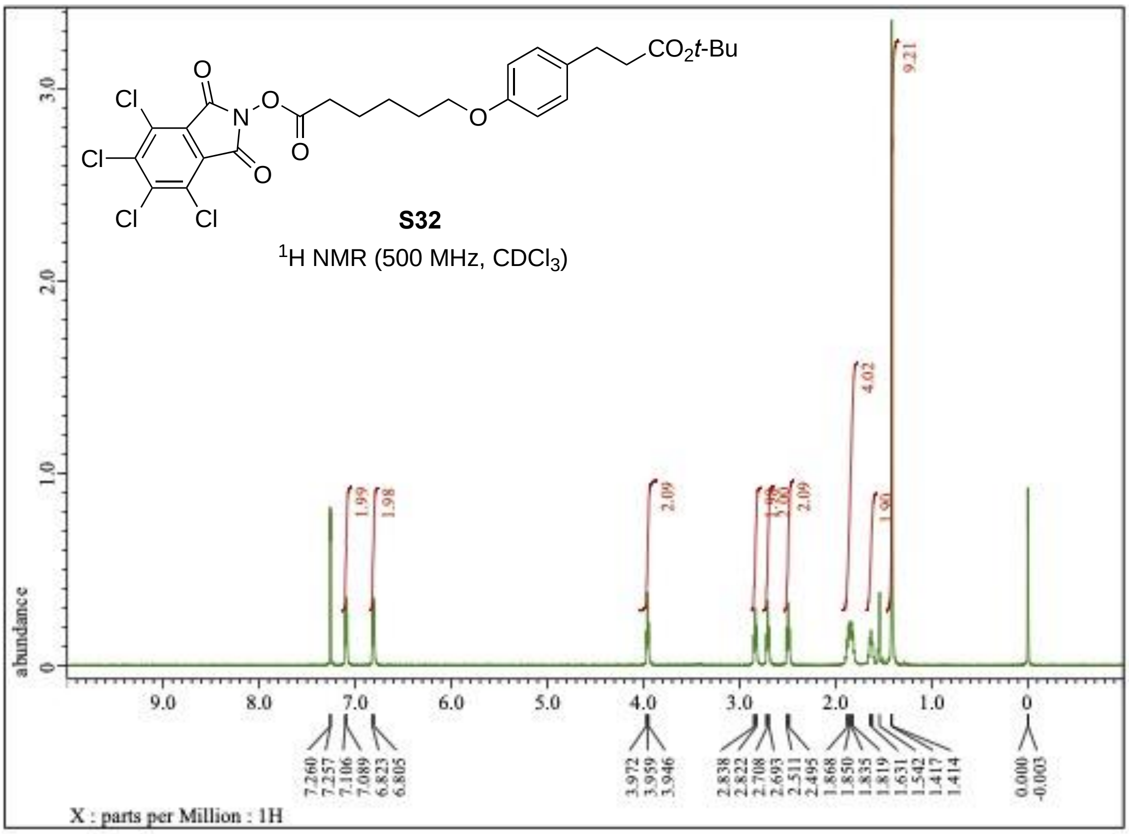
CO2t-Bu
S32
1H NMR (500 MHz, CDCl3)
abundance
X : parts per Million : 1H
7.260 7.257 7.106 7.089 6.823 6.805
3.972 3.959 3.946
2.838 2.822 2.708 2.693 2.511 2.495 1.868 1.850 1.835 1.819 1.631 1.542 1.417 1.414
0.000 -0.003
1.99 1.98 2.09 2.09 4.02 9.21

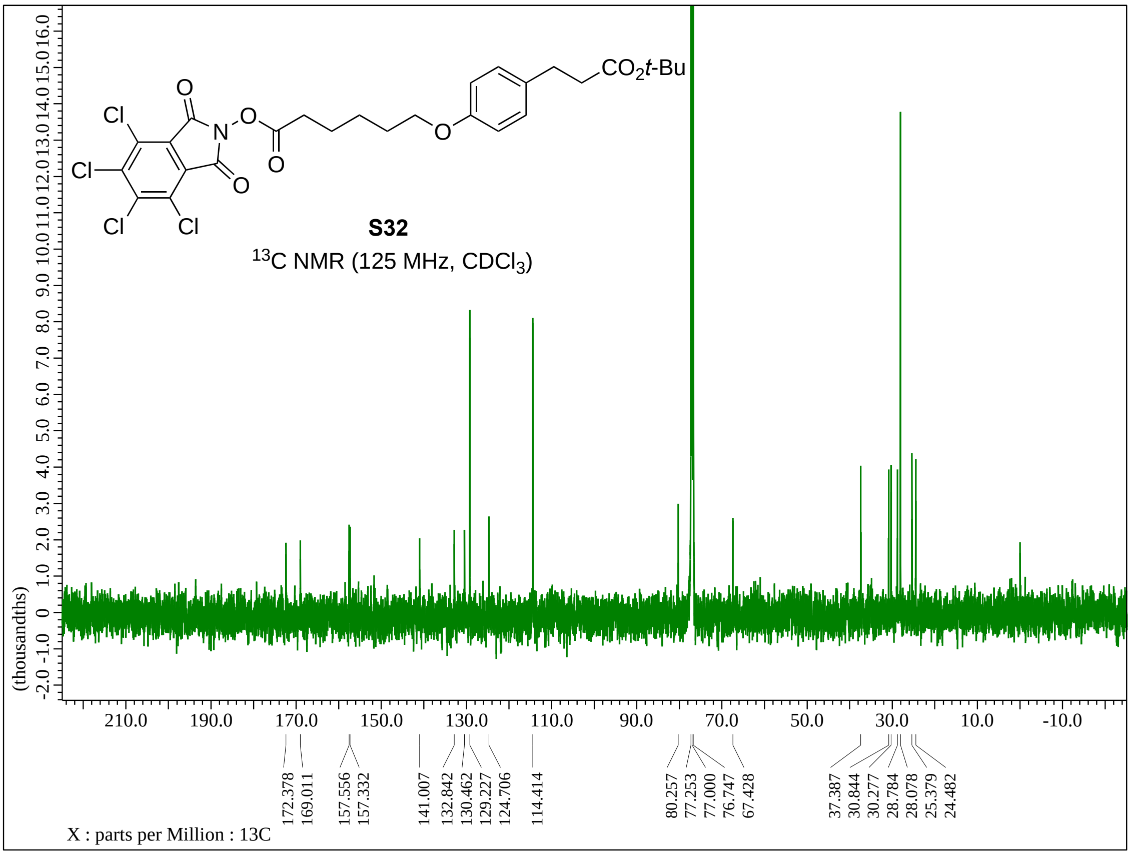
CO2t-Bu
S32
13C NMR (125 MHz, CDCl3)
(thousandths)
X : parts per Million : 13C
172.378 169.011 157.556 157.332 141.007 132.842 130.462 129.227 124.706 114.414
80.257 77.253 77.000 76.747 67.428
37.387 30.844 30.277 28.784 28.078 25.379 24.482

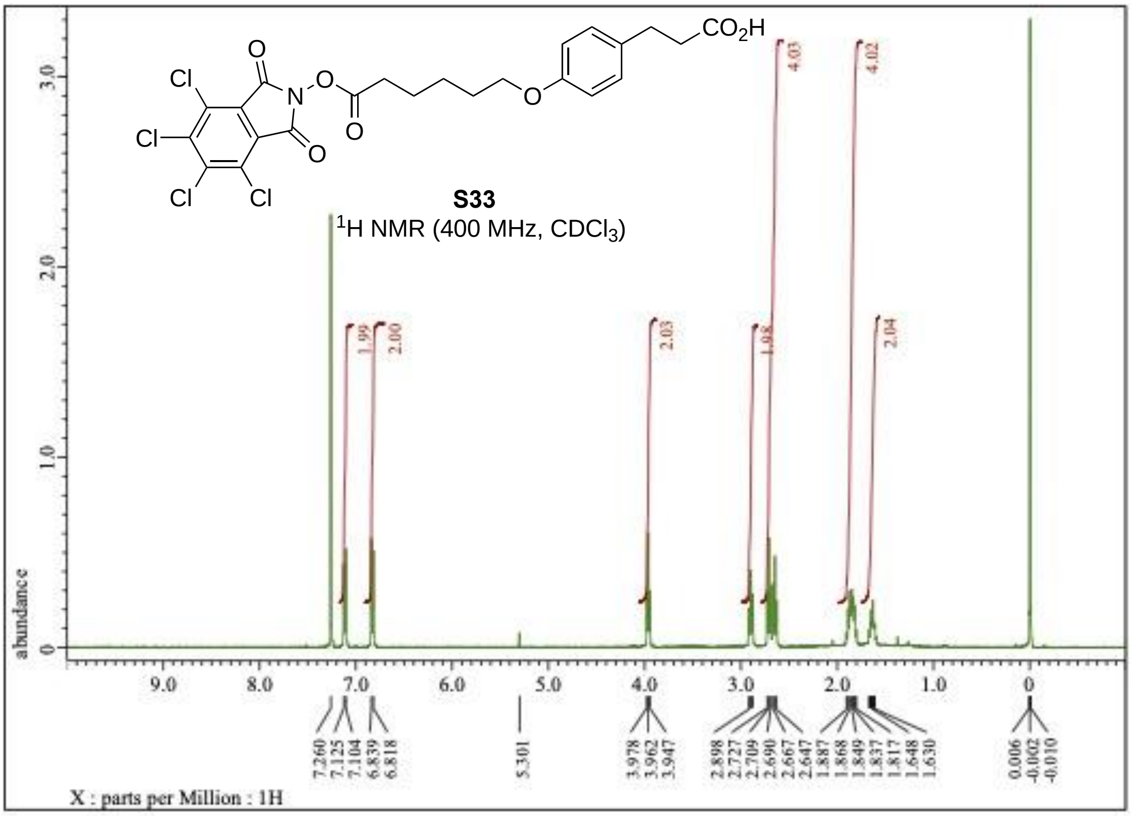
CO2H
Cl
Cl
Cl
Cl
O
O
O
O
N
O
O
S33
1H NMR (400 MHz, CDCl3)
abundance
X : parts per Million : 1H
7.260
7.125
7.104
6.839
6.818
5.301
3.978
3.962
3.947
2.898
2.727
2.709
2.690
2.667
2.647
1.887
1.868
1.849
1.837
1.817
1.648
1.630
0.006
-0.002
-0.010

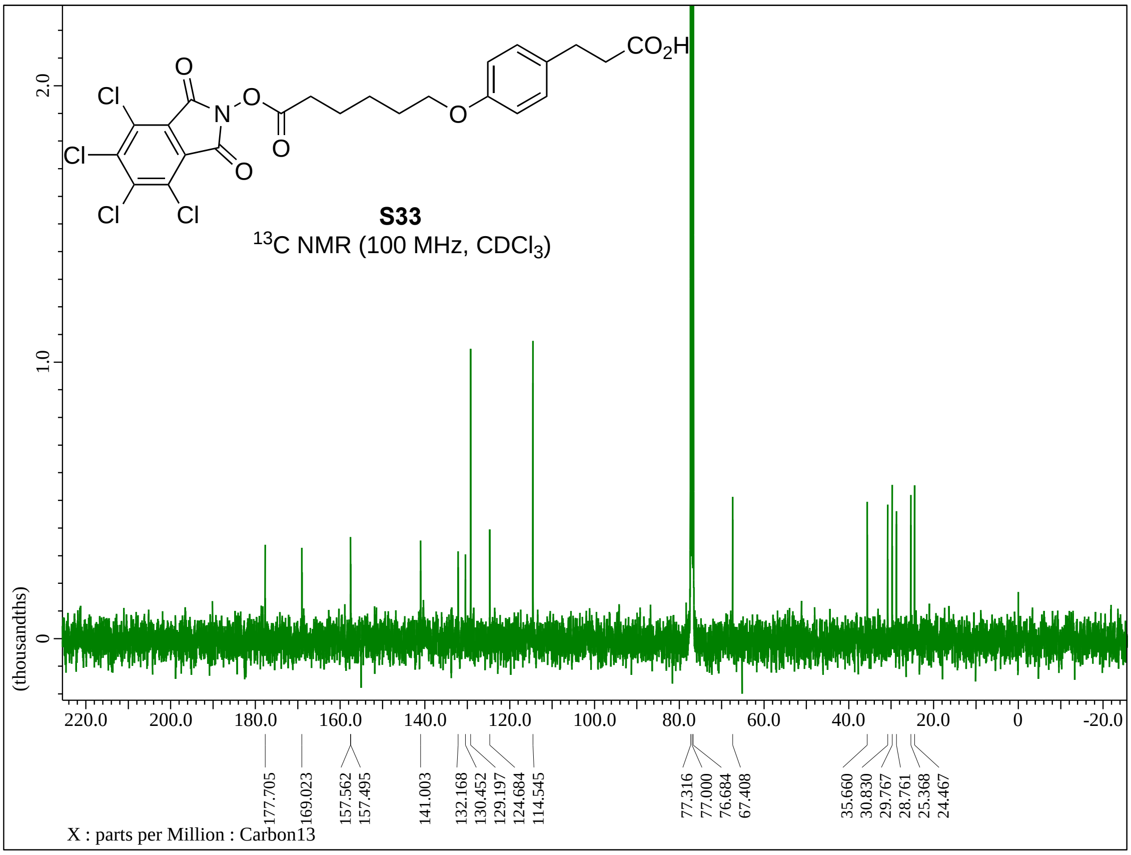
CO2H
S33
13C NMR (100 MHz, CDCl3)
(thousandths)
X : parts per Million : Carbon13
177.705
169.023
157.562
157.495
141.003
132.168
130.452
129.197
124.684
114.545
77.316
77.000
76.684
67.408
35.660
30.830
29.767
28.761
25.368
24.467

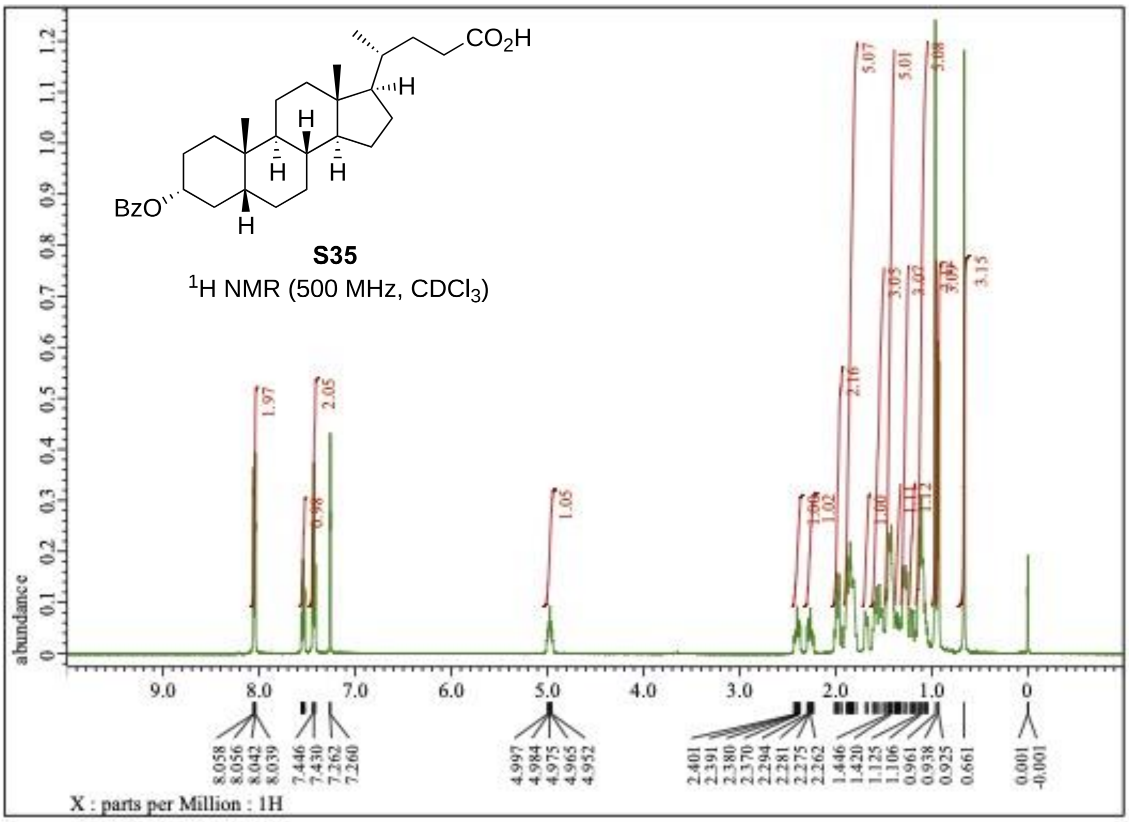
CO2H
BzO
S35
1H NMR (500 MHz, CDCl3)
abundance
X : parts per Million : 1H

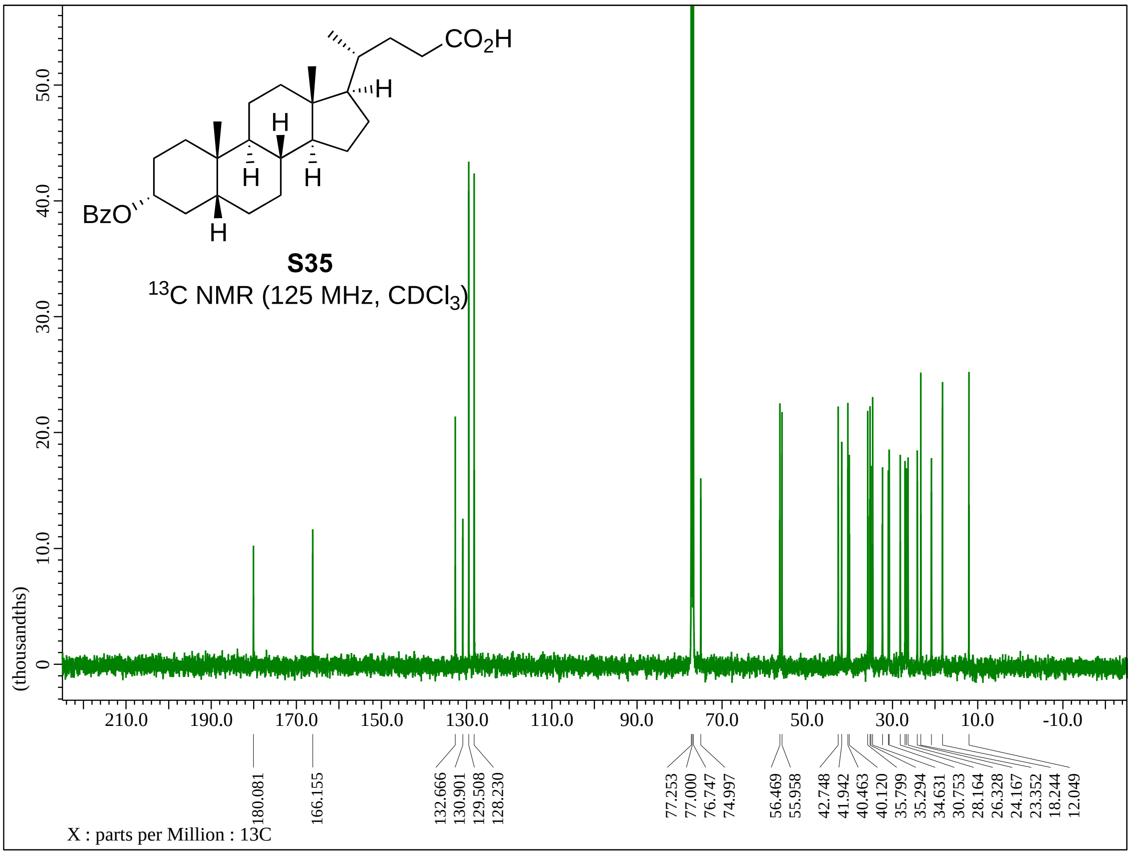
CO2H
BzO
S35
13C NMR (125 MHz, CDCl3)
(thousandths)
X : parts per Million : 13C

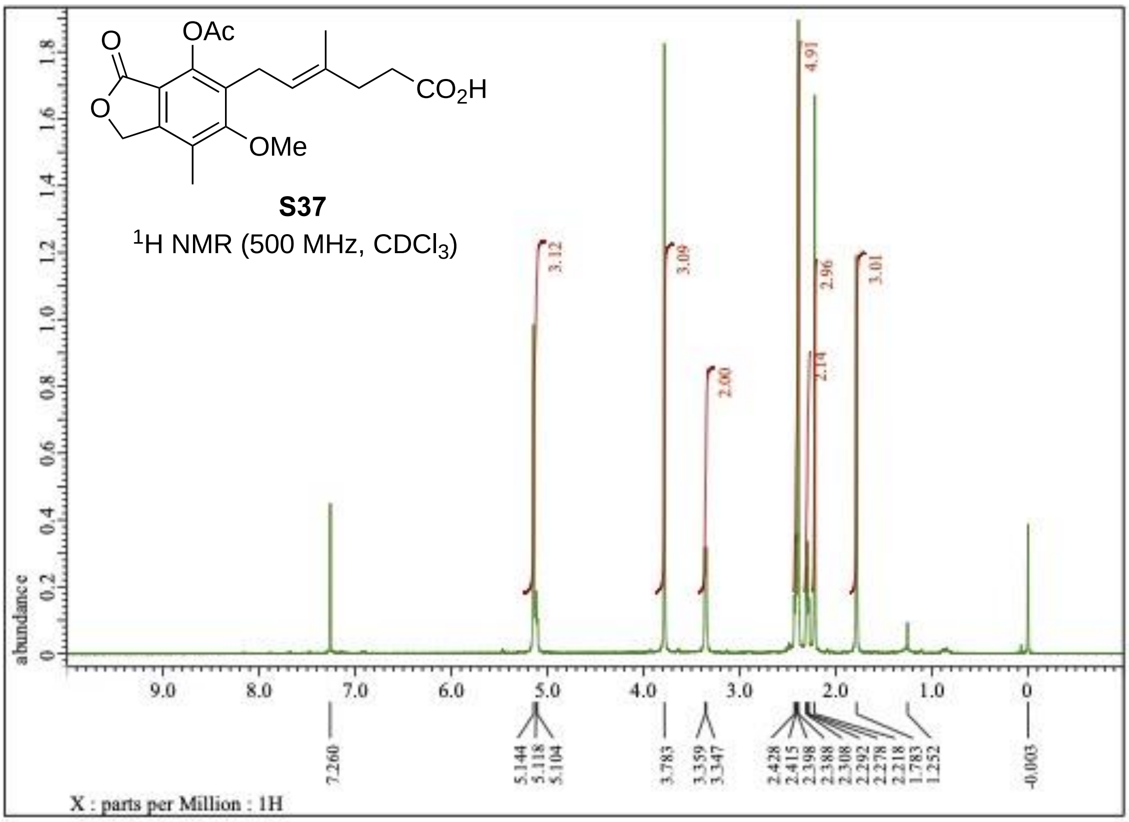
OAc
O
O
CO2H
OMe
S37
1H NMR (500 MHz, CDCl3)
abundance
3.12
3.09
2.00
4.91
2.14
2.96
3.01
9.0 8.0 7.0 6.0 5.0 4.0 3.0 2.0 1.0 0
7.260
5.144 5.118 5.104
3.783
3.359 3.347
2.428 2.415 2.398 2.388 2.308 2.292 2.278 2.218 1.783 1.252
-0.003
X : parts per Million : 1H

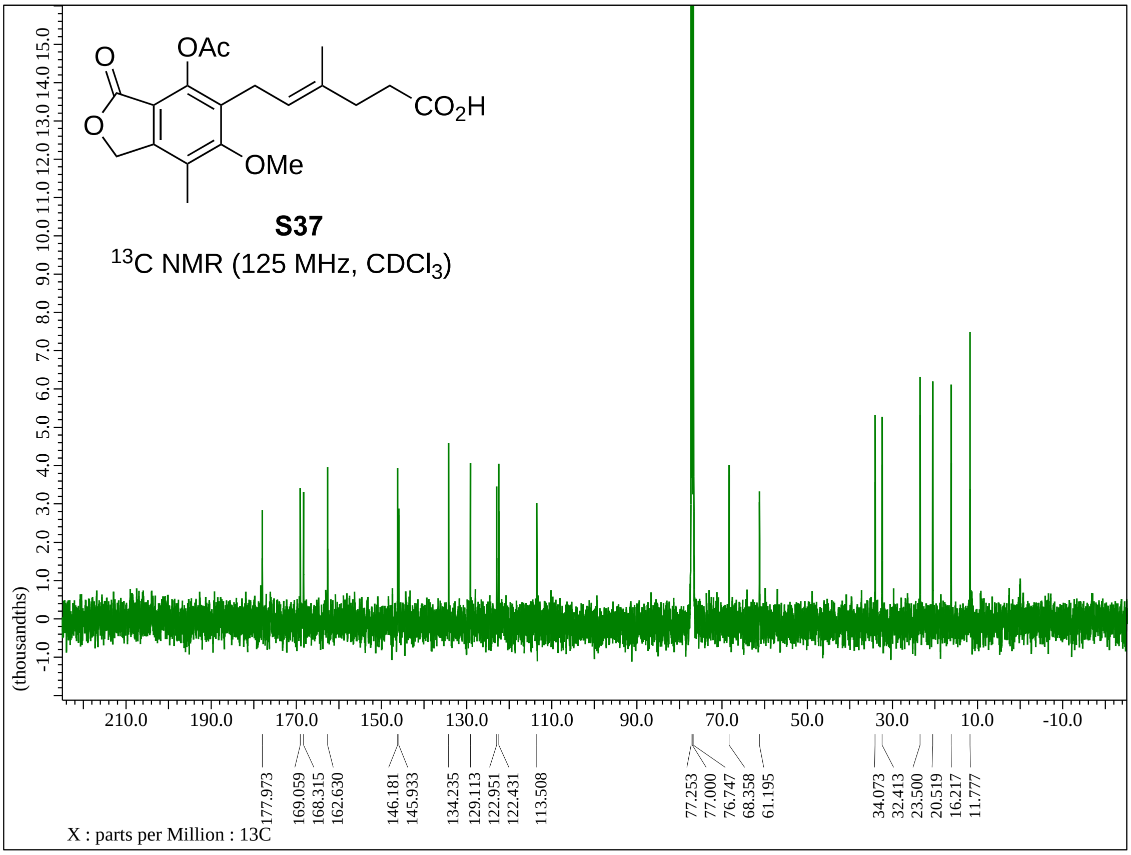
OAc
O
O
CO2H
OMe
S37
13C NMR (125 MHz, CDCl3)
(thousandths)
210.0 190.0 170.0 150.0 130.0 110.0 90.0 70.0 50.0 30.0 10.0 -10.0
177.973
169.059 168.315 162.630
146.181 145.933
134.235 129.113 122.951 122.431
113.508
77.253 77.000 76.747 68.358 61.195
34.073 32.413 23.500 20.519 16.217 11.777
X : parts per Million : 13C

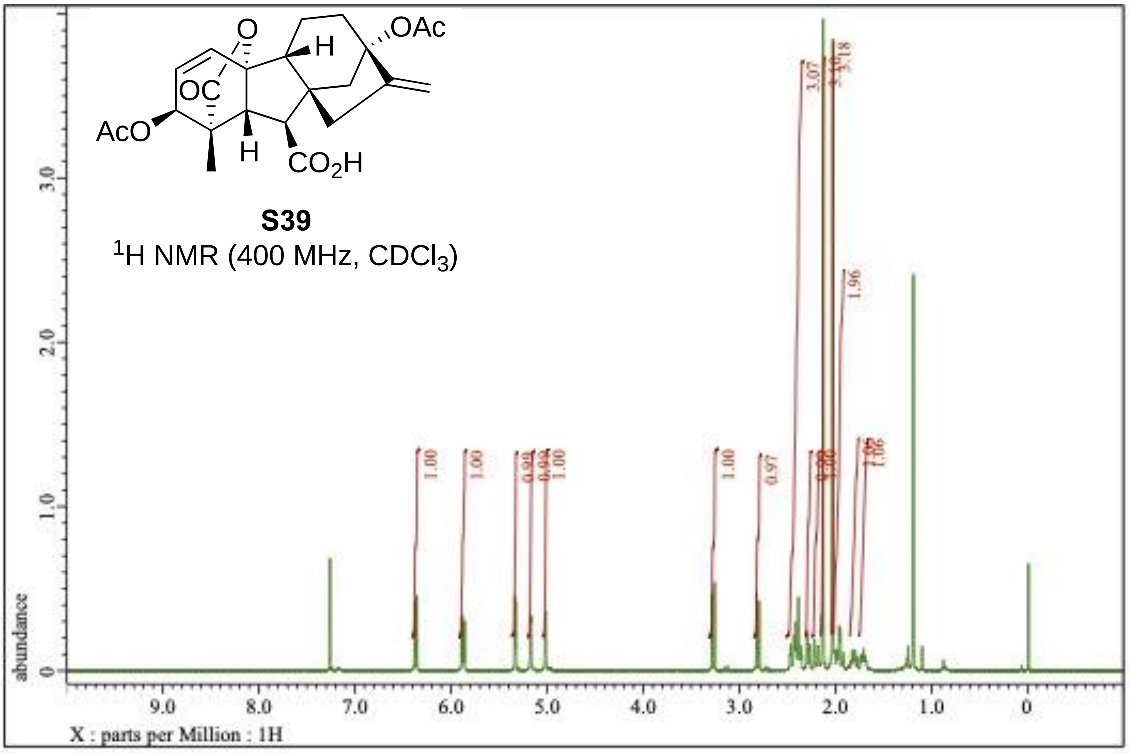

OAc
AcO
OC
H
CO2H
S39
1H NMR (400 MHz, CDCl3)
abundance
X : parts per Million : 1H

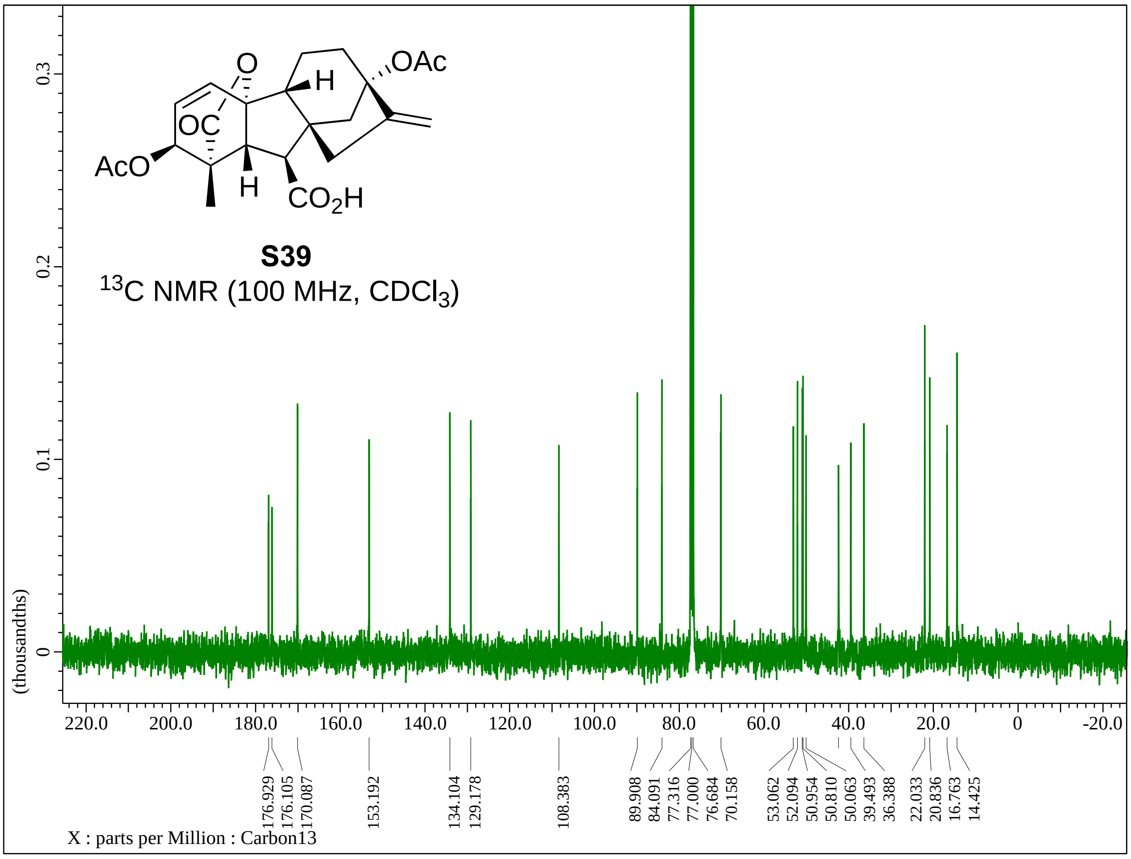

OAc
AcO
OC
H
CO2H
S39
13C NMR (100 MHz, CDCl3)
(thousandths)
176.929
176.105
170.087
153.192
134.104
129.178
108.383
89.908
84.091
77.316
77.000
76.684
70.158
53.062
52.094
50.954
50.810
50.063
39.493
36.388
22.033
20.836
16.763
14.425
X : parts per Million : Carbon13

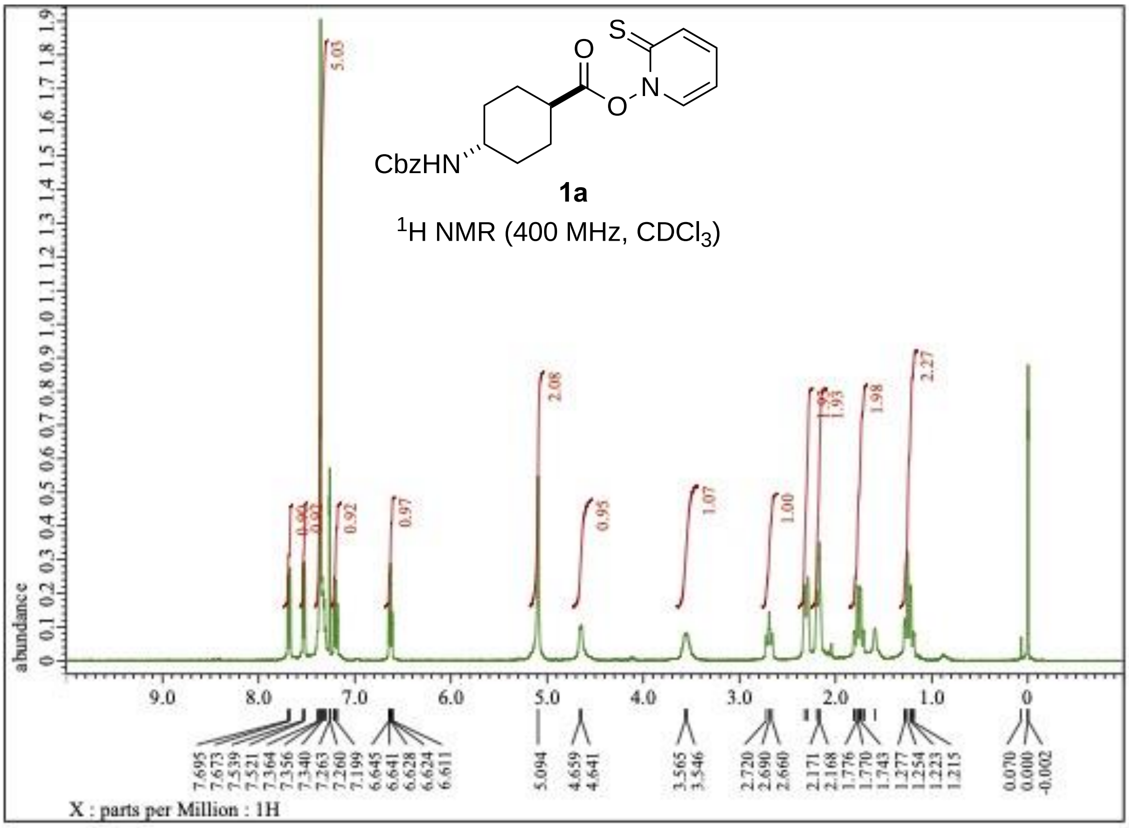
CbzHN
1a
^{1}H NMR (400 MHz, CDCl$_3$)
abundance
X : parts per Million : 1H

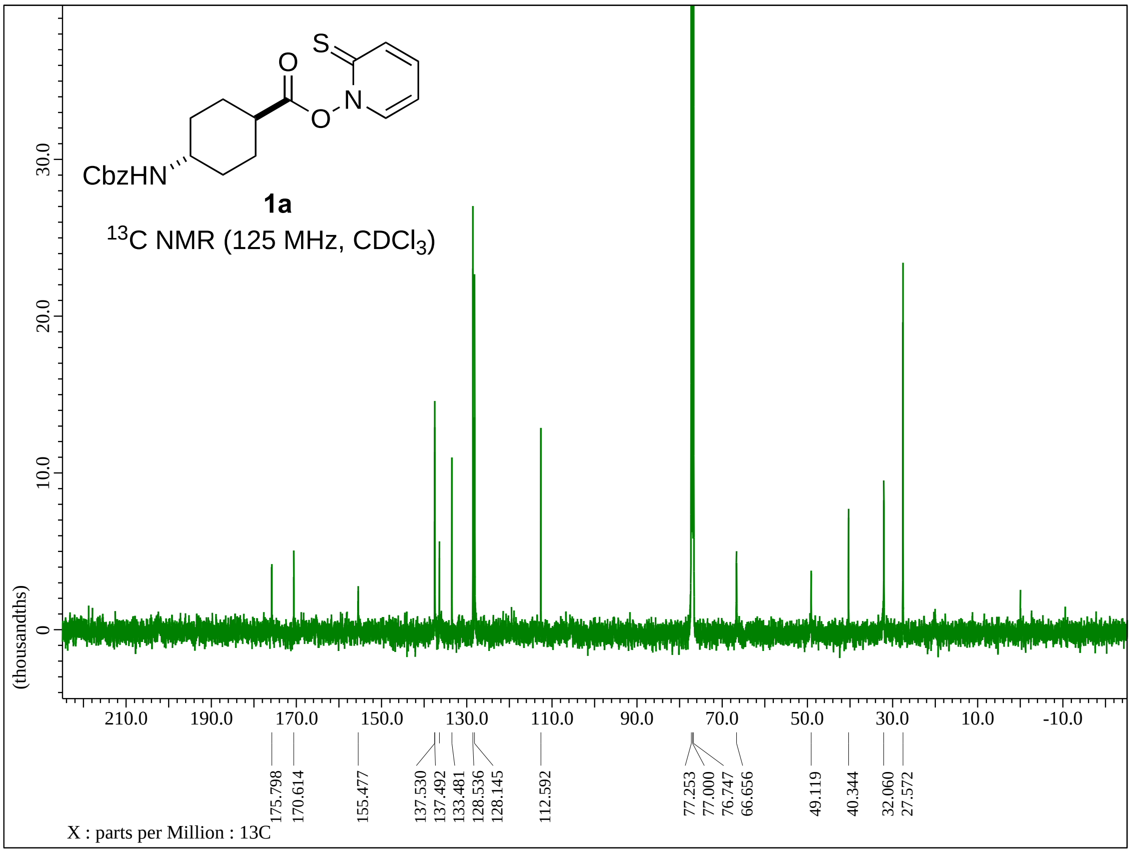
CbzHN
1a
^{13}C NMR (125 MHz, CDCl$_3$)
(thousandths)
X : parts per Million : 13C

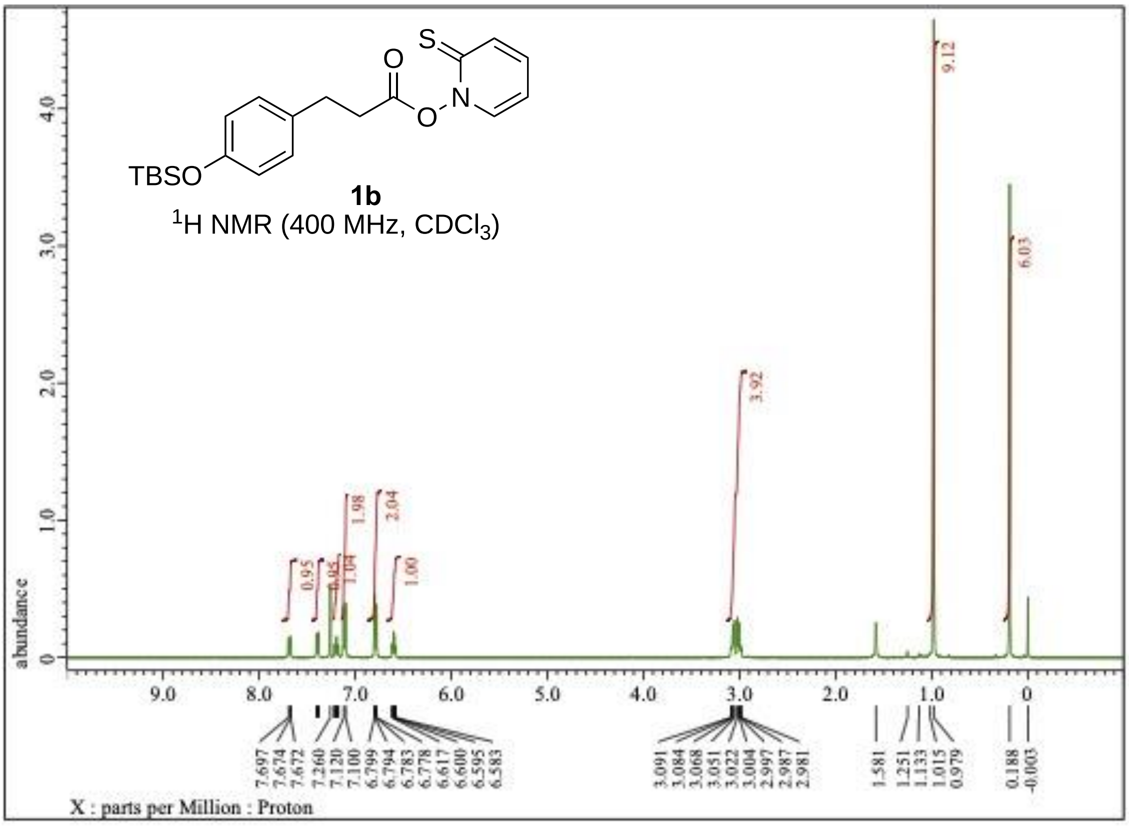
TBSO
1b
1H NMR (400 MHz, CDCl3)
abundance
X : parts per Million : Proton
9.12
6.03
3.92
2.04
1.98
1.00
0.95
1.04
7.697 7.674 7.672 7.260 7.120 7.100 6.799 6.794 6.783 6.778 6.617 6.600 6.595 6.583
3.091 3.084 3.068 3.051 3.022 3.004 2.997 2.987 2.981
1.581 1.251 1.133 1.015 0.979
0.188 -0.003

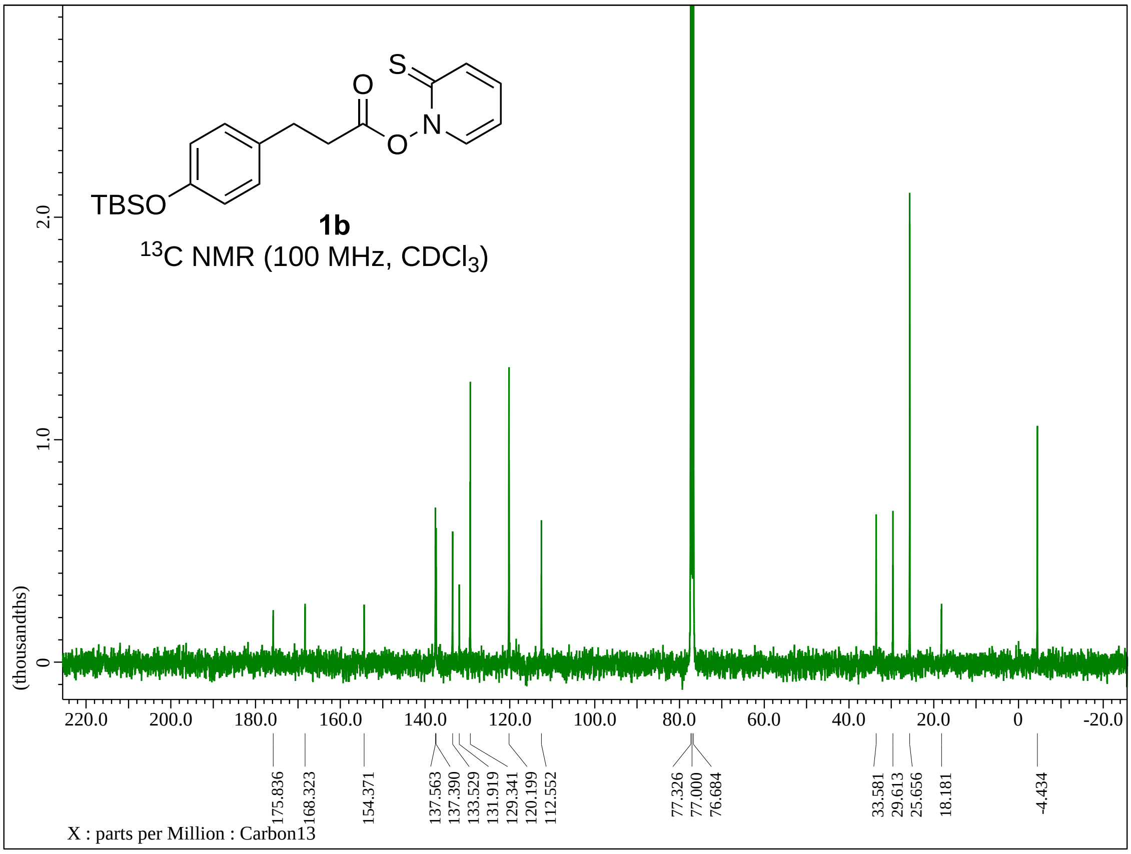
TBSO
1b
13C NMR (100 MHz, CDCl3)
(thousandths)
X : parts per Million : Carbon13
175.836 168.323 154.371 137.563 137.390 133.529 131.919 129.341 120.199 112.552
77.326 77.000 76.684
33.581 29.613 25.656 18.181 -4.434

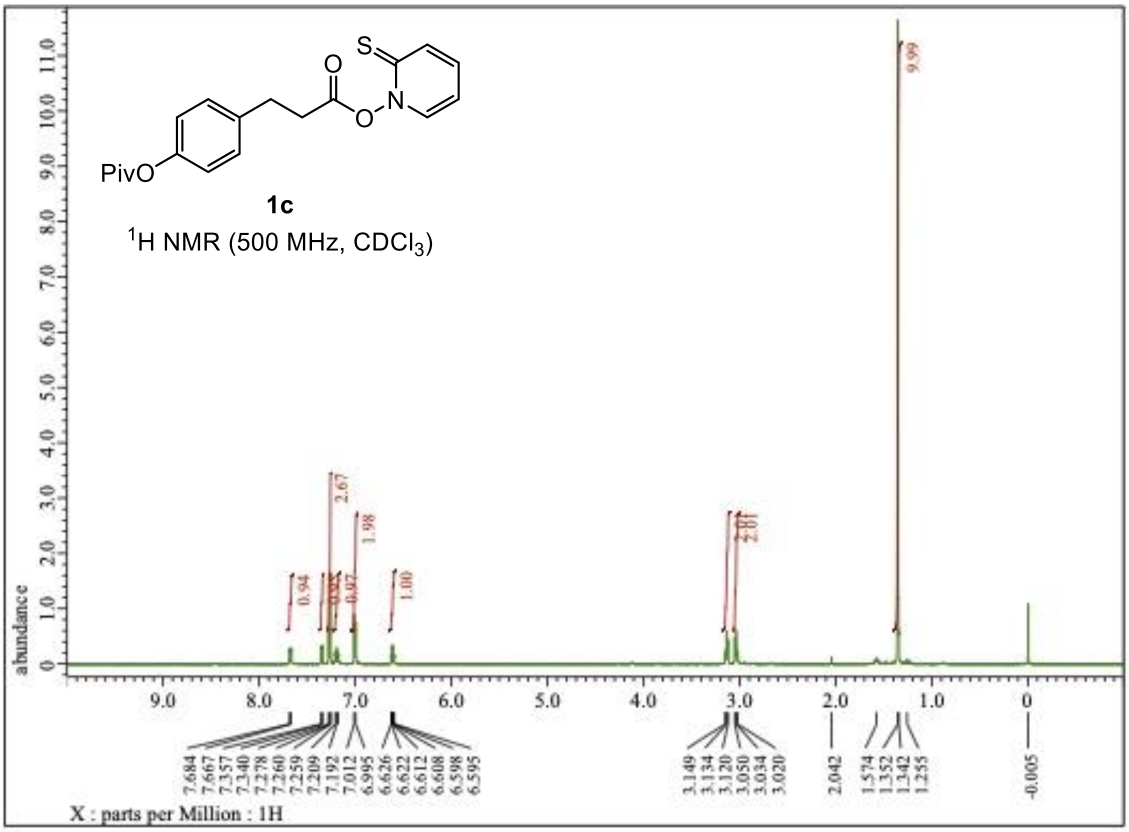
1c
^{1}H NMR (500 MHz, CDCl$_3$)
PivO
X : parts per Million : 1H
abundance
0.94
2.67
1.98
1.00
2.01
2.01
9.99
7.684
7.667
7.357
7.340
7.278
7.260
7.259
7.209
7.192
7.012
6.995
6.626
6.622
6.612
6.608
6.598
6.595
3.149
3.134
3.120
3.050
3.034
3.020
2.042
1.574
1.352
1.342
1.255
-0.005

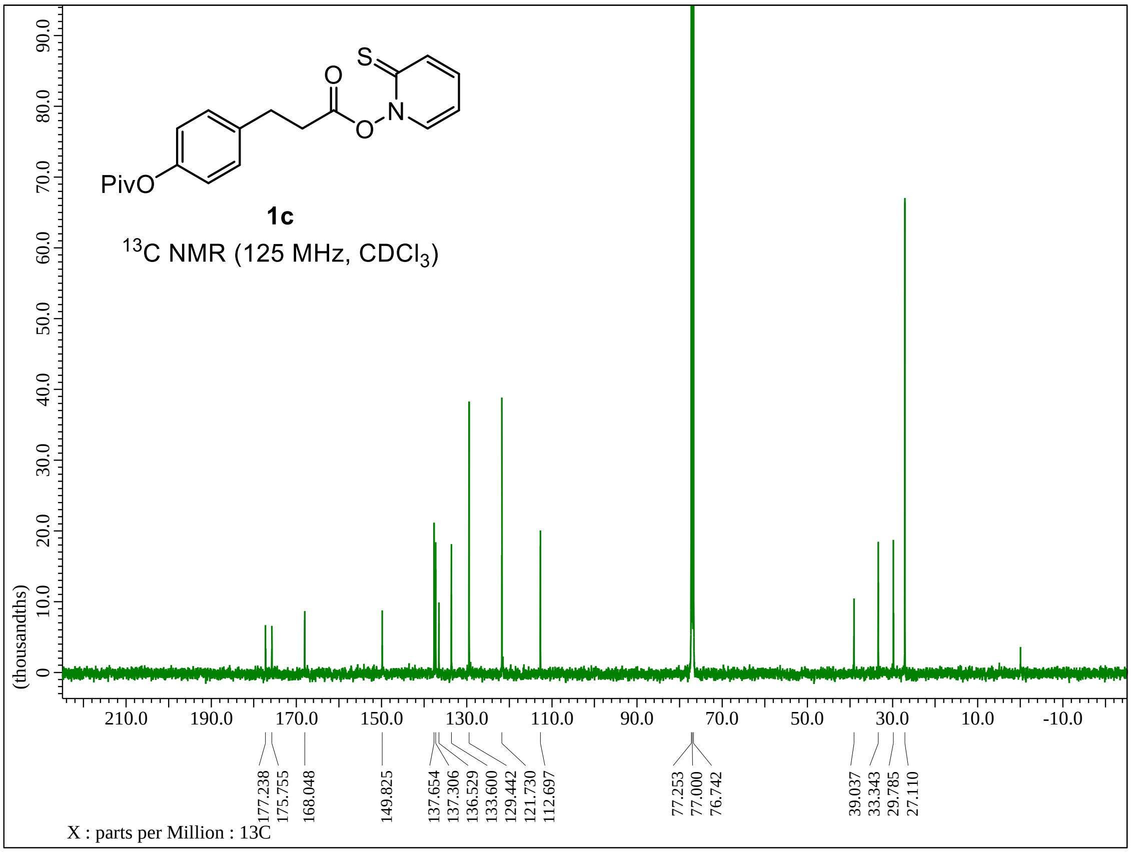
1c
^{13}C NMR (125 MHz, CDCl$_3$)
PivO
X : parts per Million : 13C
(thousandths)
177.238
175.755
168.048
149.825
137.654
137.306
136.529
133.600
129.442
121.730
112.697
77.253
77.000
76.742
39.037
33.343
29.785
27.110

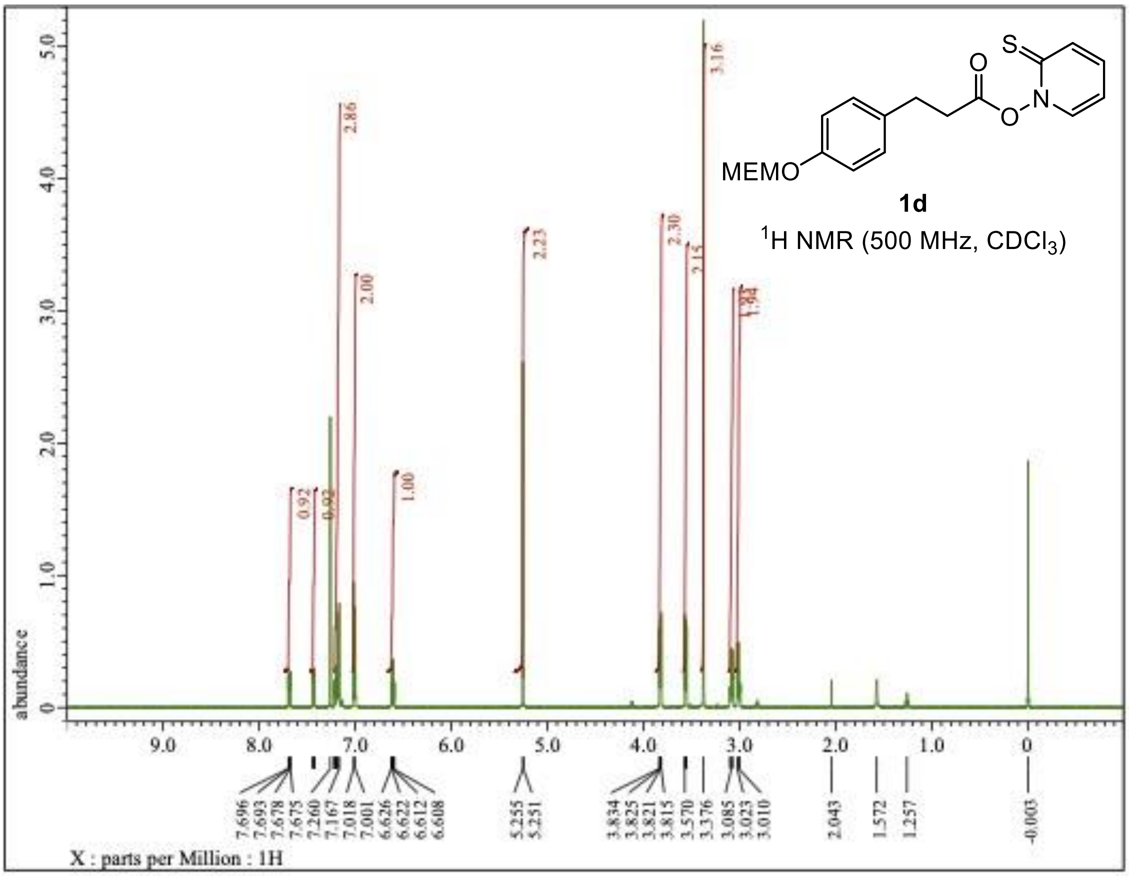
MEMO
1d
¹H NMR (500 MHz, CDCl₃)
abundance
X : parts per Million : 1H

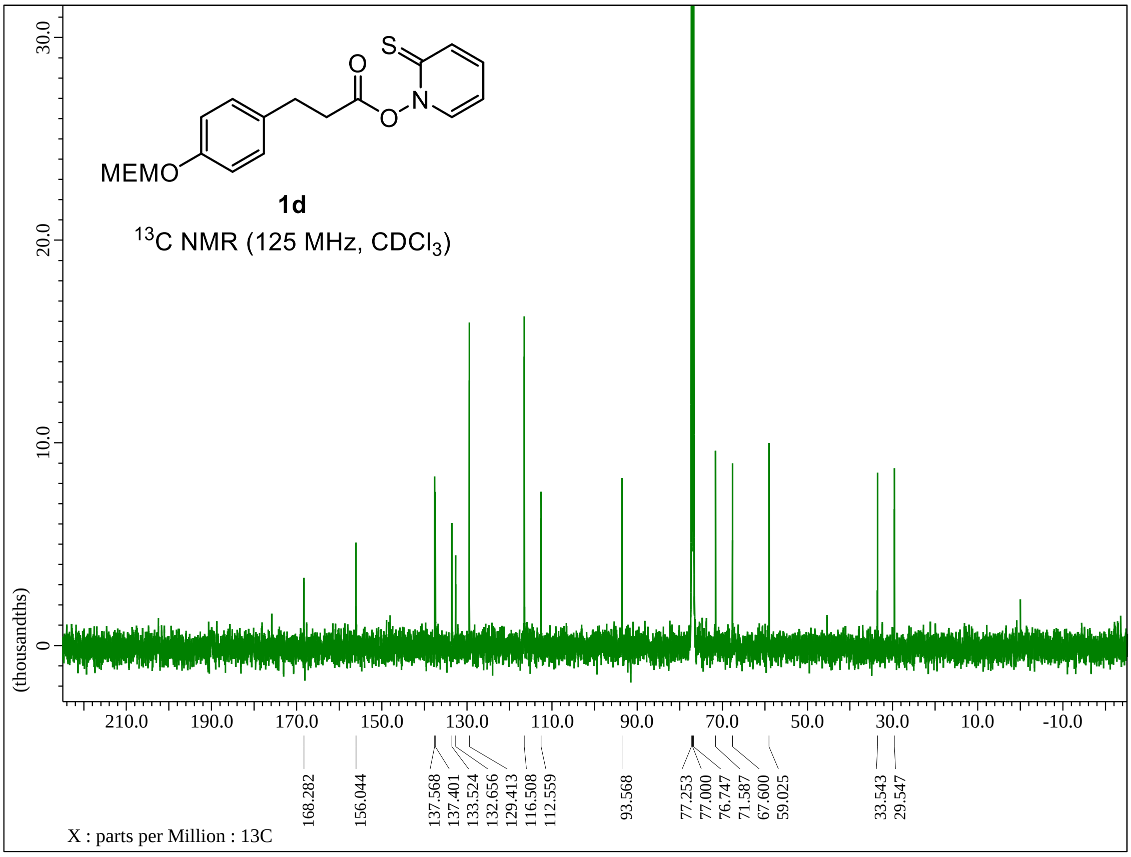
MEMO
1d
¹³C NMR (125 MHz, CDCl₃)
(thousandths)
X : parts per Million : 13C

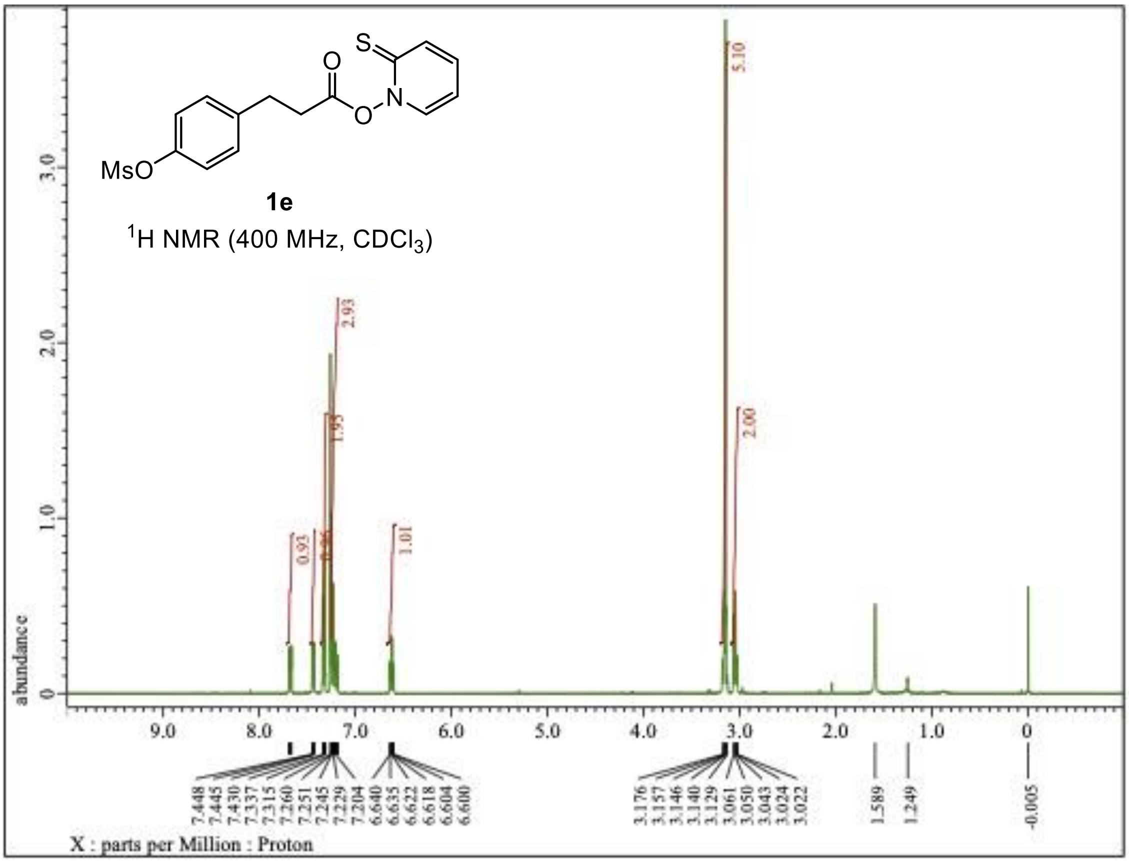
MsO
1e
¹H NMR (400 MHz, CDCl3)
X : parts per Million : Proton

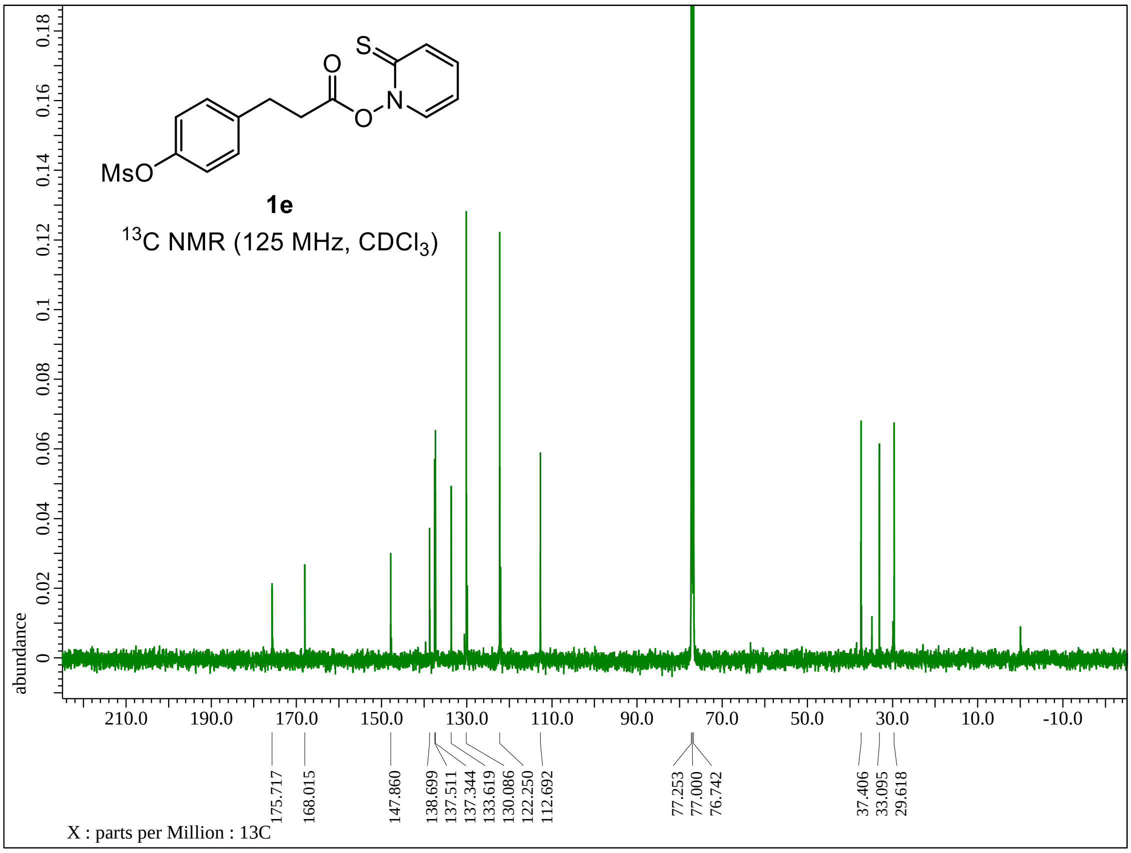
MsO
1e
¹³C NMR (125 MHz, CDCl3)
X : parts per Million : 13C

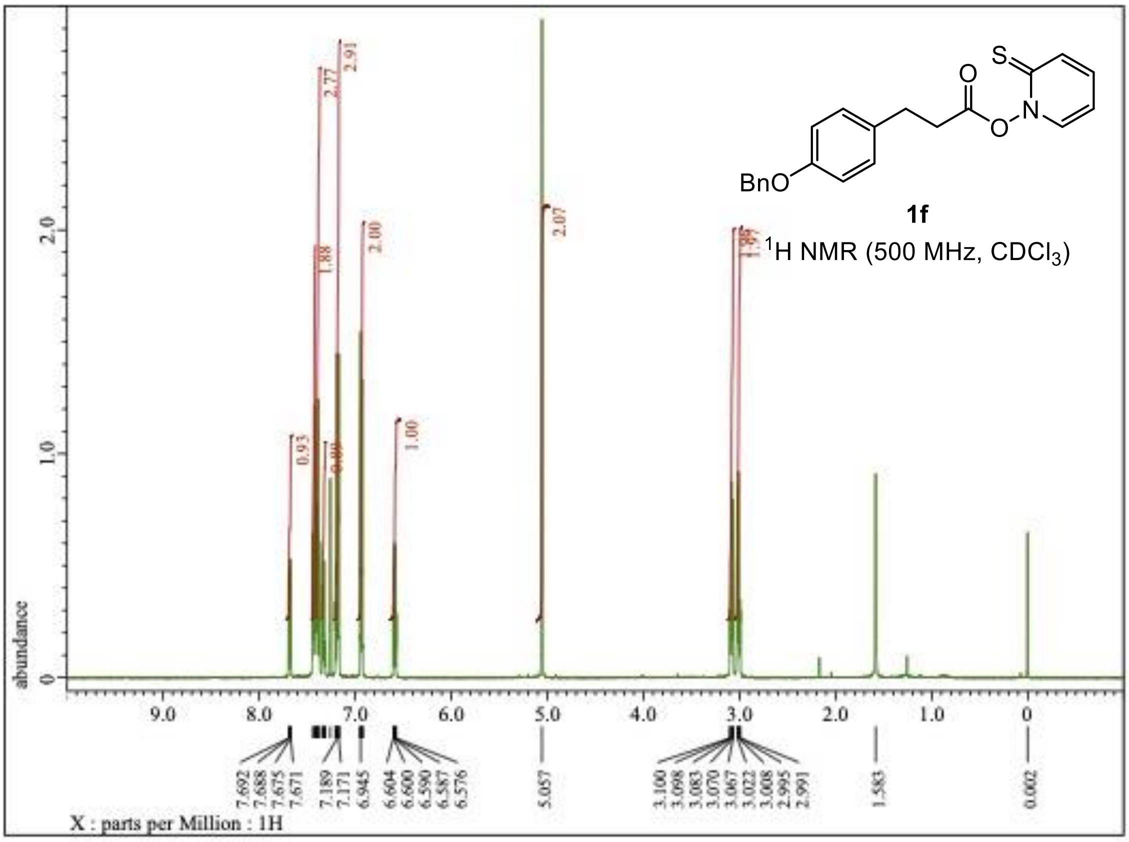
BnO
1f
¹H NMR (500 MHz, CDCl₃)
abundance
X : parts per Million : 1H
9.0
8.0
7.0
6.0
5.0
4.0
3.0
2.0
1.0
0
7.692
7.688
7.675
7.671
7.189
7.171
6.945
6.604
6.600
6.590
6.587
6.576
5.057
3.100
3.098
3.083
3.070
3.067
3.022
3.008
2.995
2.991
1.583
0.002
0.93
2.77
2.91
1.88
2.00
1.00
2.07

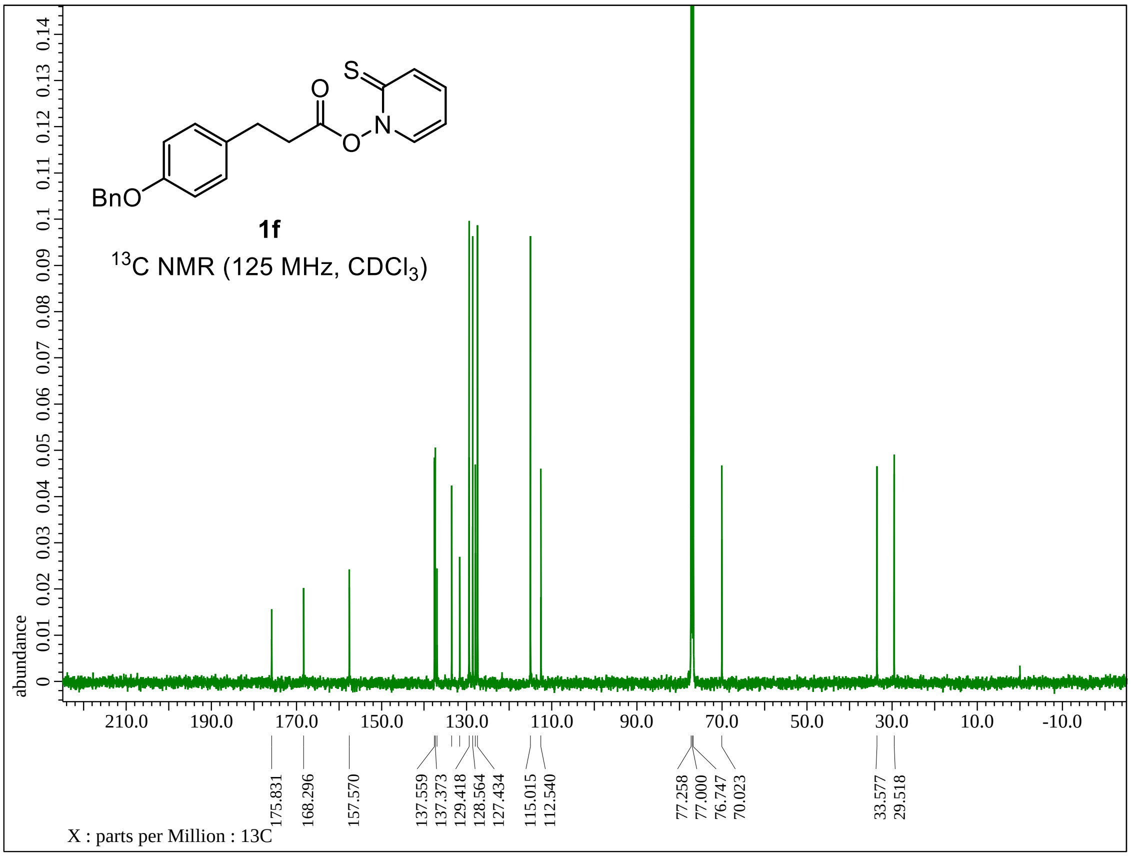
BnO
1f
¹³C NMR (125 MHz, CDCl₃)
abundance
X : parts per Million : 13C
210.0
190.0
170.0
150.0
130.0
110.0
90.0
70.0
50.0
30.0
10.0
-10.0
175.831
168.296
157.570
137.559
137.373
129.418
128.564
127.434
115.015
112.540
77.258
77.000
76.747
70.023
33.577
29.518

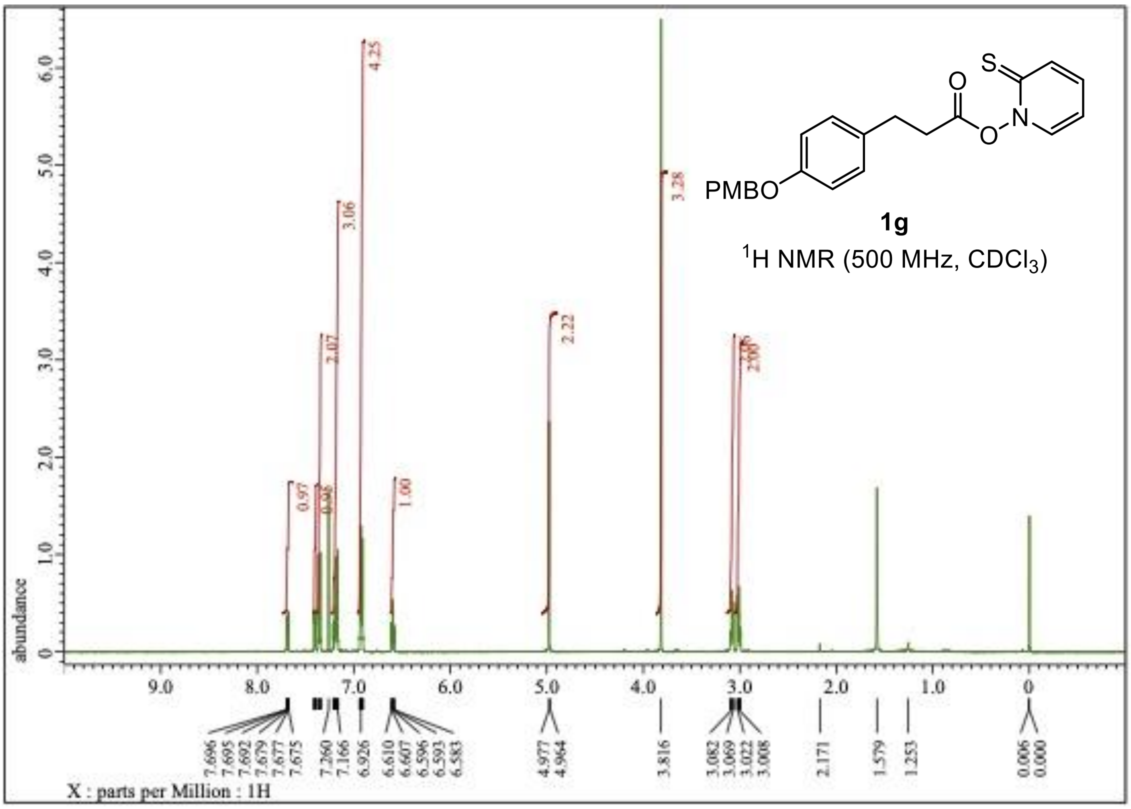
PMBO
1g
1H NMR (500 MHz, CDCl3)
abundance
X : parts per Million : 1H
7.696
7.695
7.692
7.679
7.677
7.675
7.260
7.166
6.926
6.610
6.607
6.596
6.593
6.583
4.977
4.964
3.816
3.082
3.069
3.022
3.008
2.171
1.579
1.253
0.006
0.000

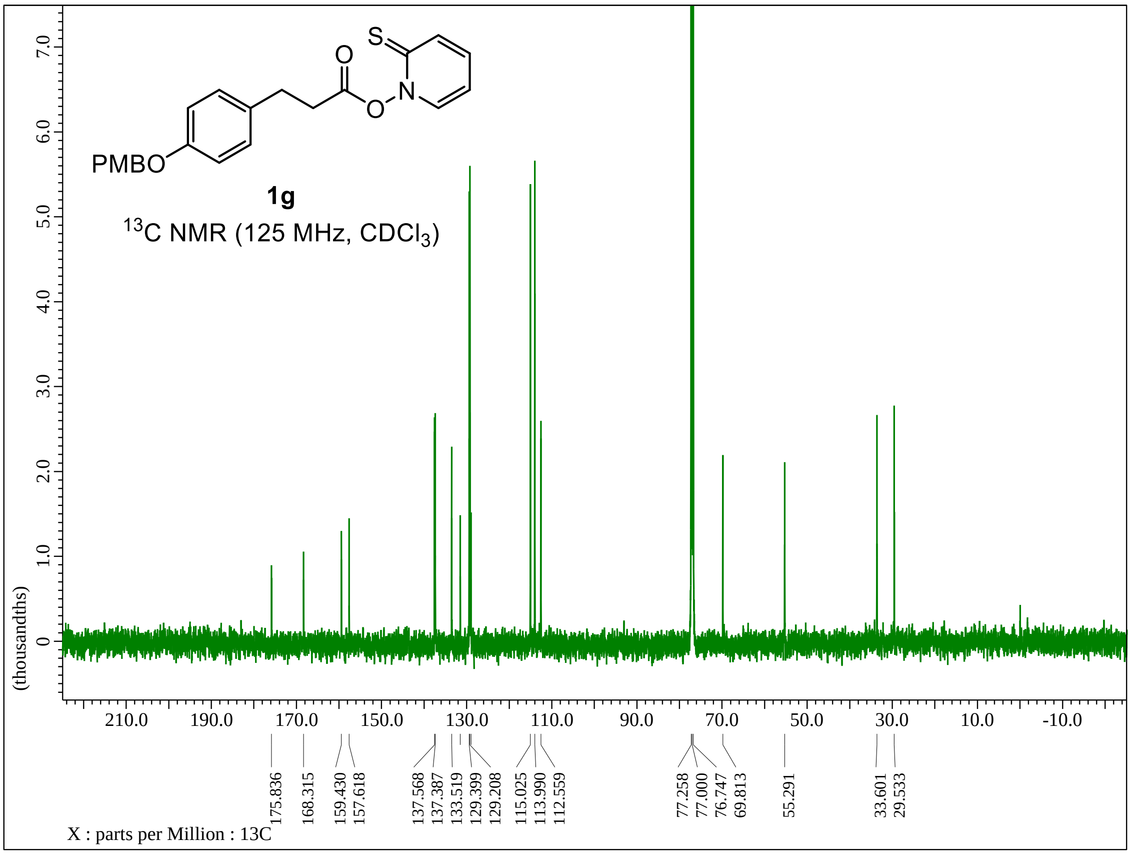
PMBO
1g
13C NMR (125 MHz, CDCl3)
(thousandths)
X : parts per Million : 13C
175.836
168.315
159.430
157.618
137.568
137.387
133.519
129.399
129.208
115.025
113.990
112.559
77.258
77.000
76.747
69.813
55.291
33.601
29.533

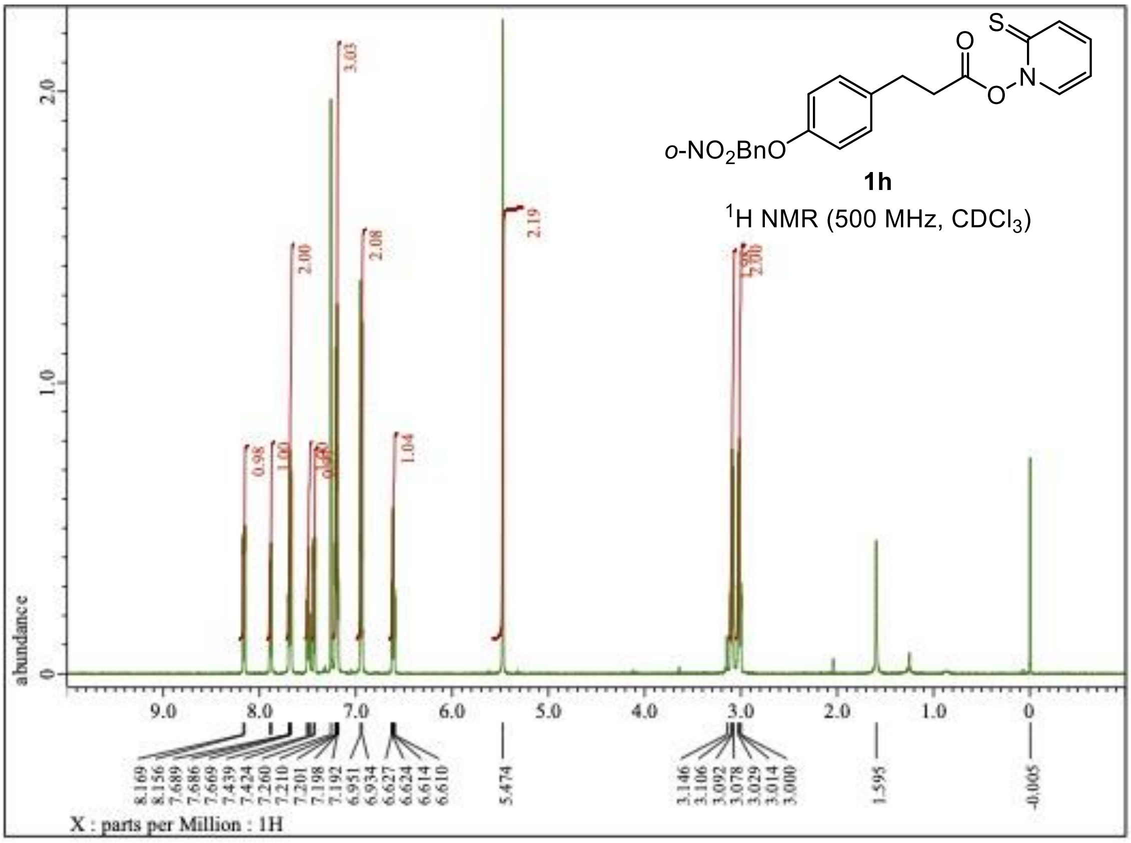
o-NO2BnO
1h
1H NMR (500 MHz, CDCl3)
X : parts per Million : 1H

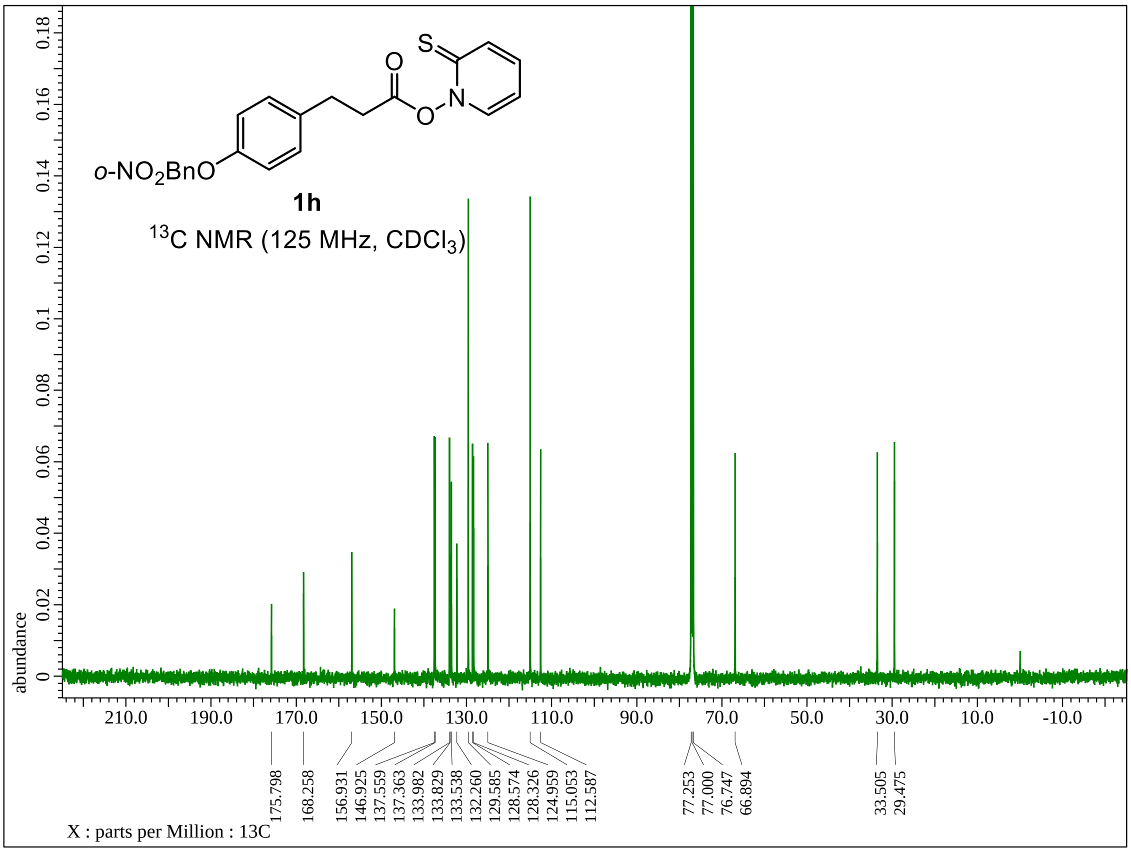
o-NO2BnO
1h
13C NMR (125 MHz, CDCl3)
X : parts per Million : 13C

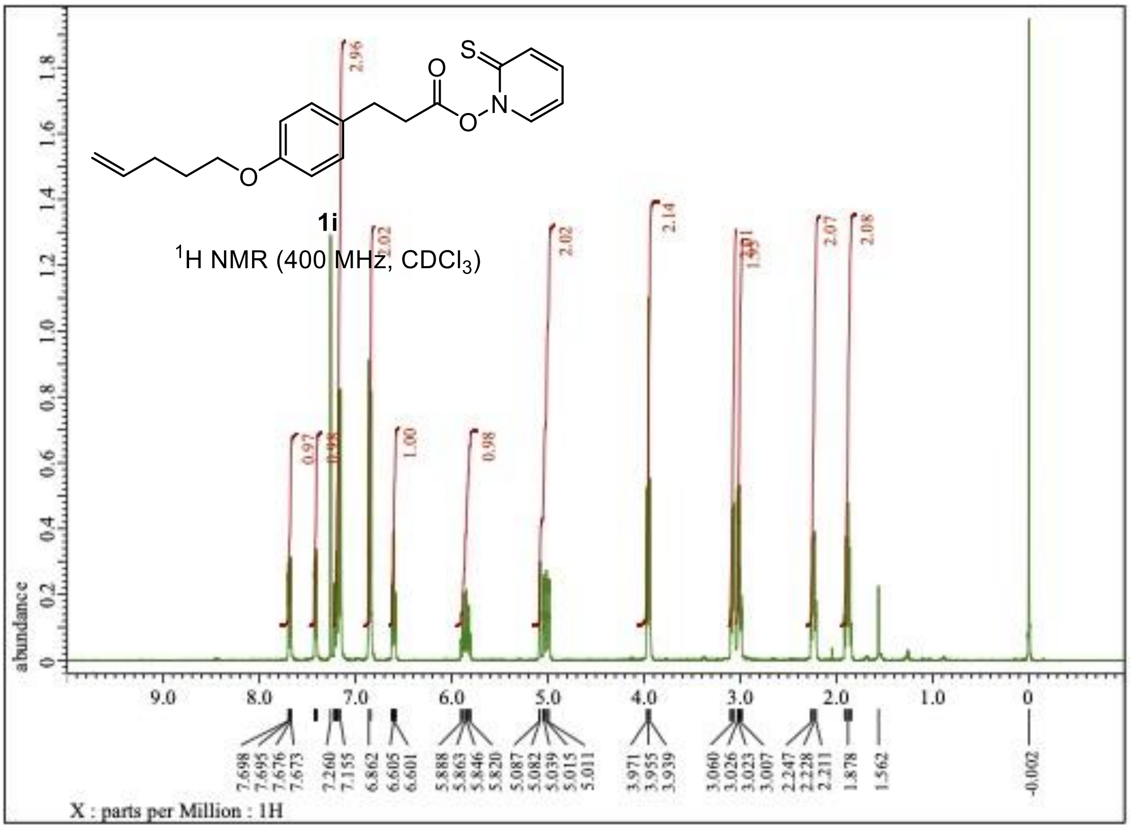
1i
^{1}H NMR (400 MHz, CDCl$_3$)
X : parts per Million : 1H
abundance

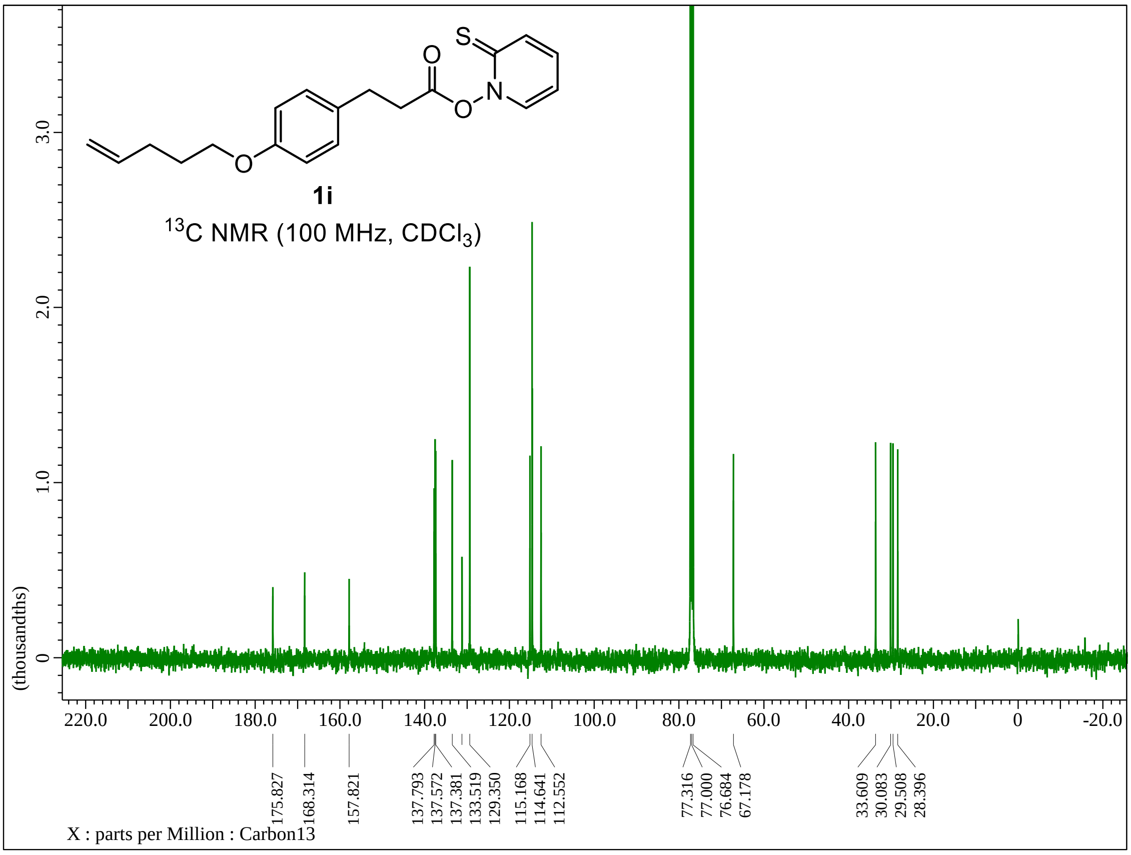
1i
^{13}C NMR (100 MHz, CDCl$_3$)
X : parts per Million : Carbon13
(thousandths)
175.827
168.314
157.821
137.793
137.572
137.381
133.519
129.350
115.168
114.641
112.552
77.316
77.000
76.684
67.178
33.609
30.083
29.508
28.396

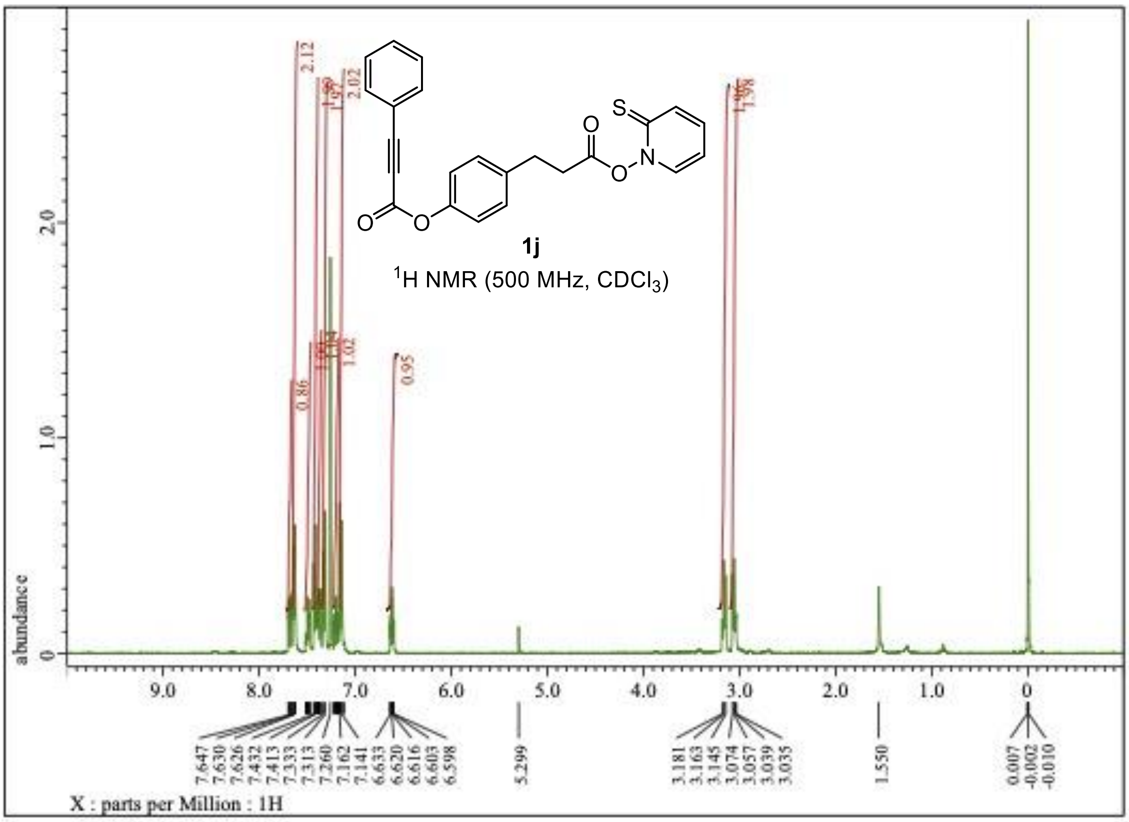

1j
^{1}H NMR (500 MHz, $CDCl_3$)
abundance
X : parts per Million : 1H

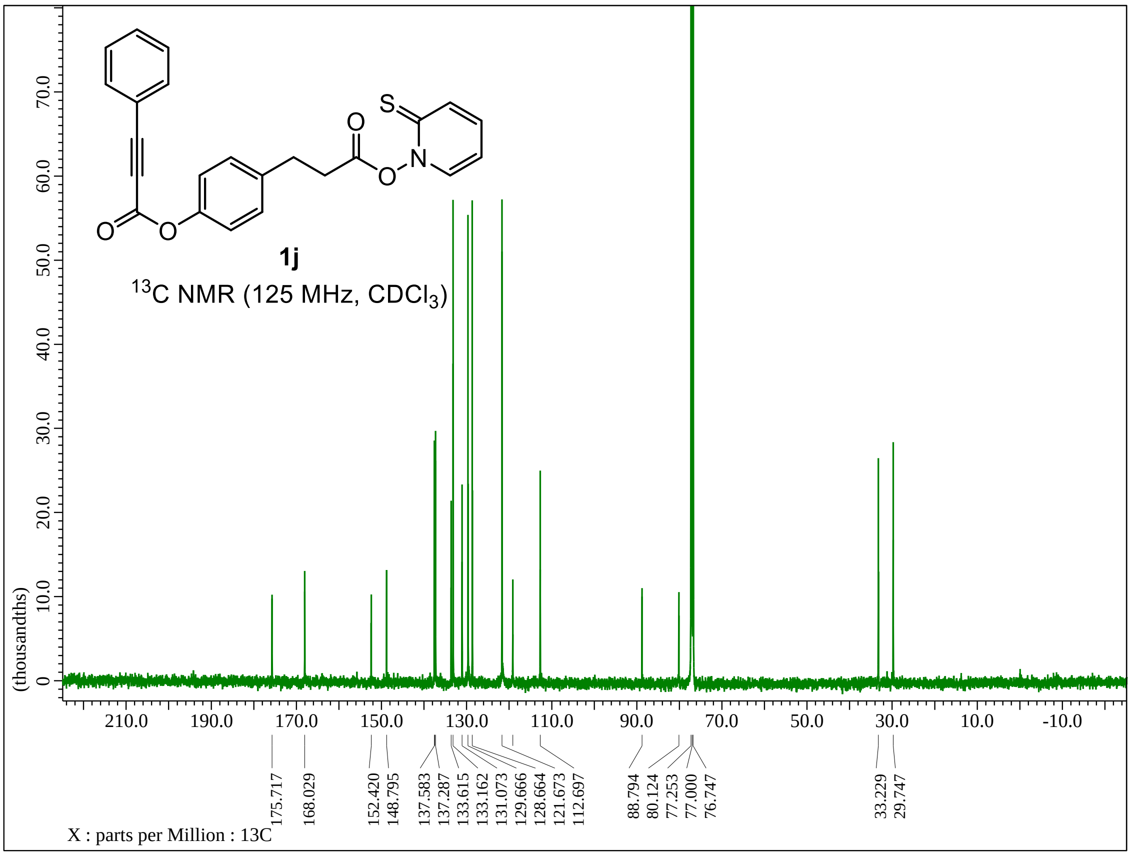

1j
^{13}C NMR (125 MHz, $CDCl_3$)
(thousandths)
X : parts per Million : 13C

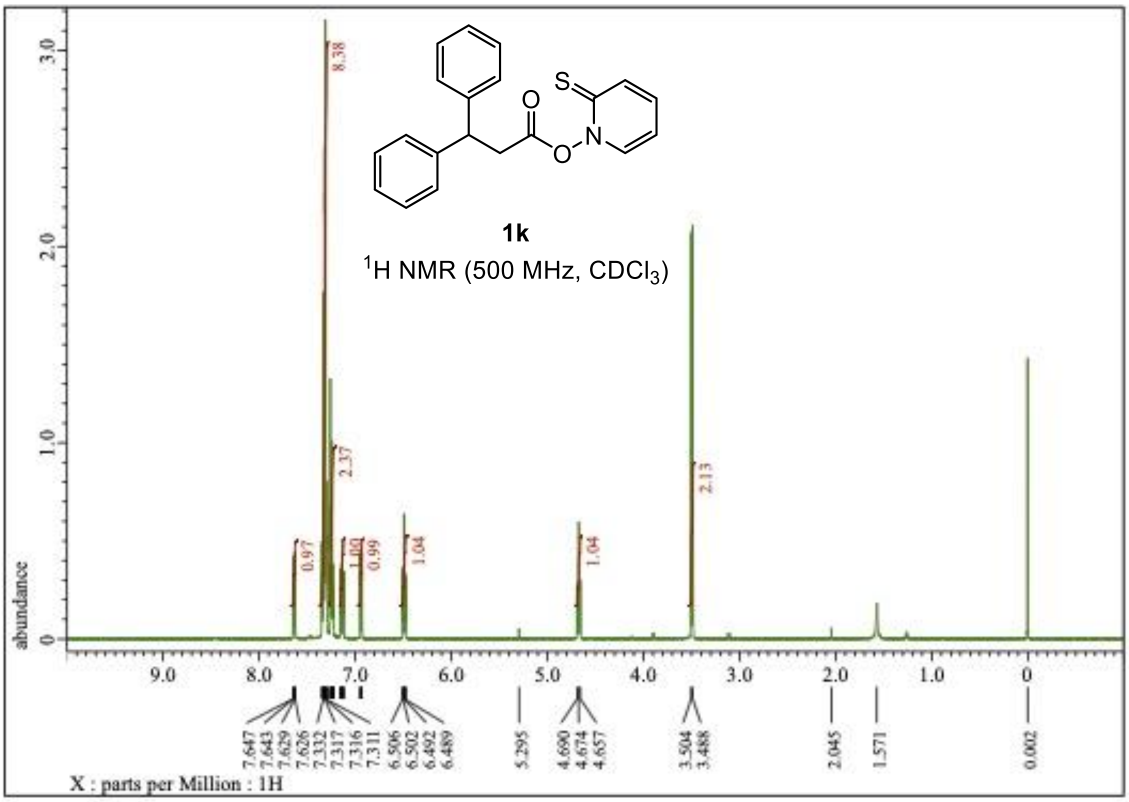

S
O
N
O
1k
^{1}H NMR (500 MHz, CDCl$_3$)
abundance
X : parts per Million : 1H
7.647
7.643
7.629
7.626
7.332
7.317
7.316
7.311
6.506
6.502
6.492
6.489
5.295
4.690
4.674
4.657
3.504
3.488
2.045
1.571
0.002

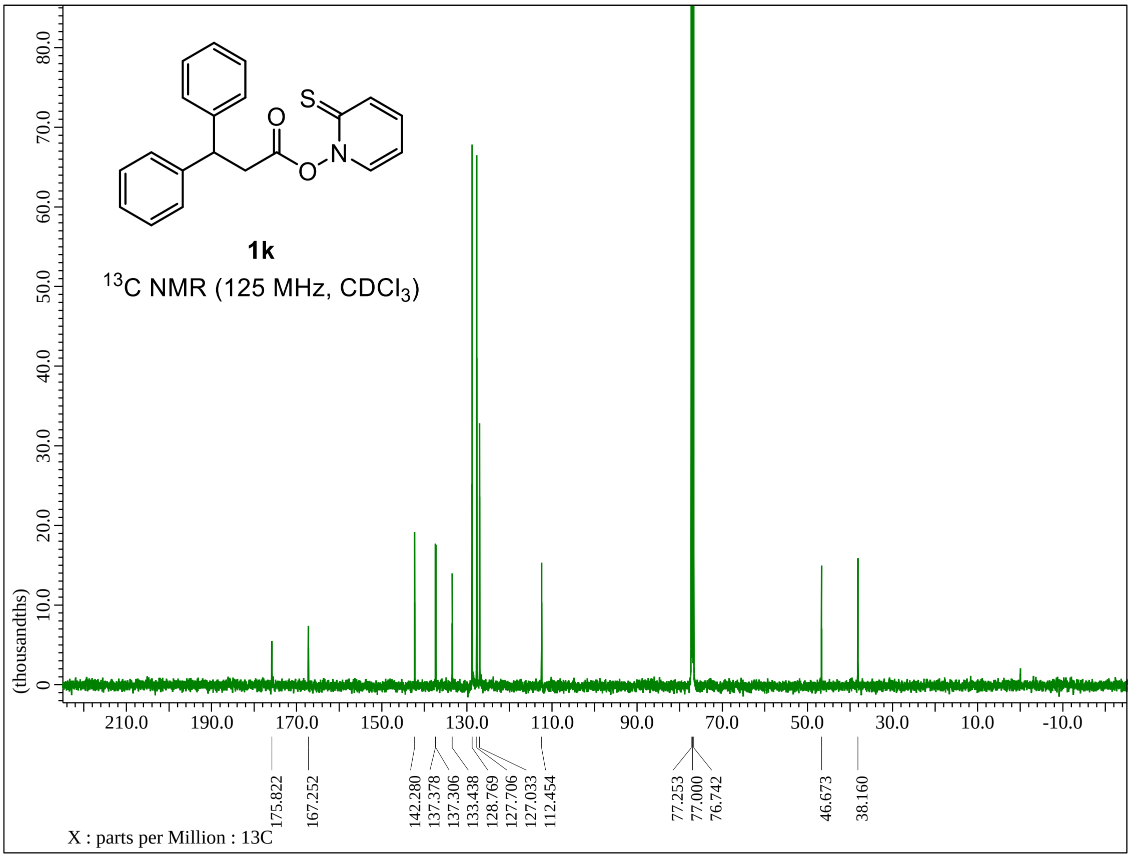

S
O
N
O
1k
^{13}C NMR (125 MHz, CDCl$_3$)
(thousandths)
X : parts per Million : 13C
175.822
167.252
142.280
137.378
137.306
133.438
128.769
127.706
127.033
112.454
77.253
77.000
76.742
46.673
38.160

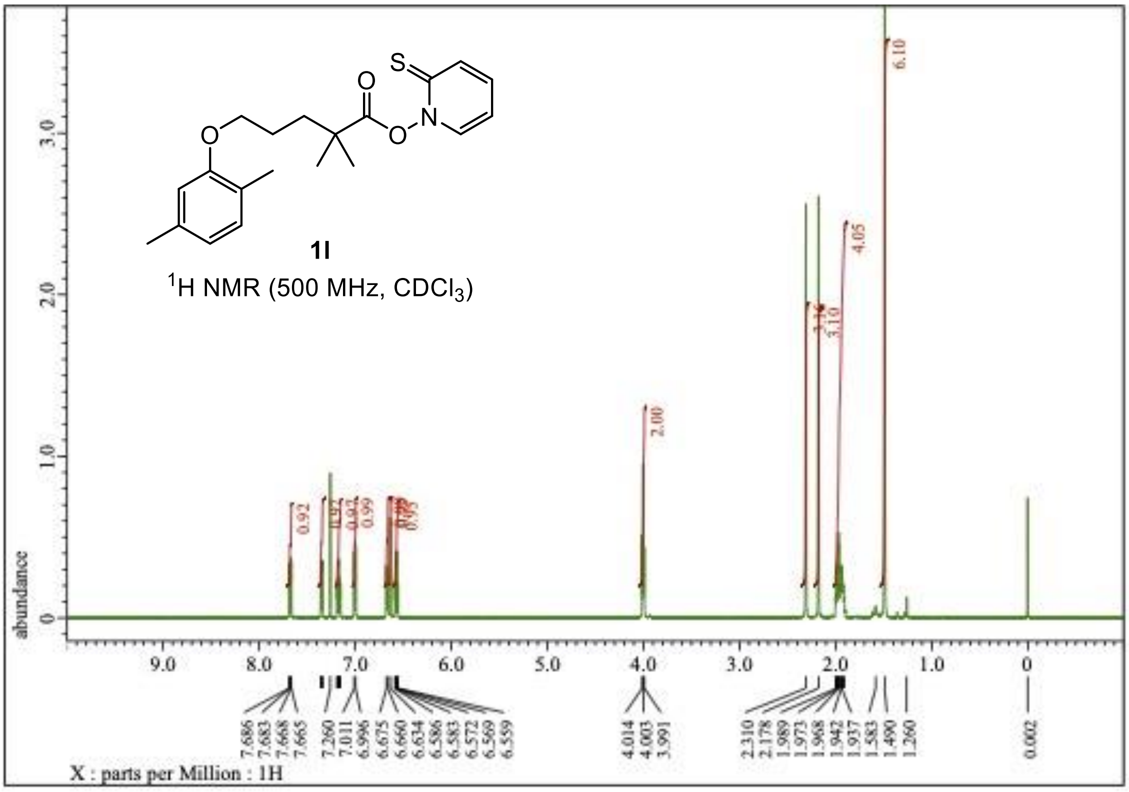
1l
^{1}H NMR (500 MHz, CDCl$_3$)
abundance
X : parts per Million : 1H

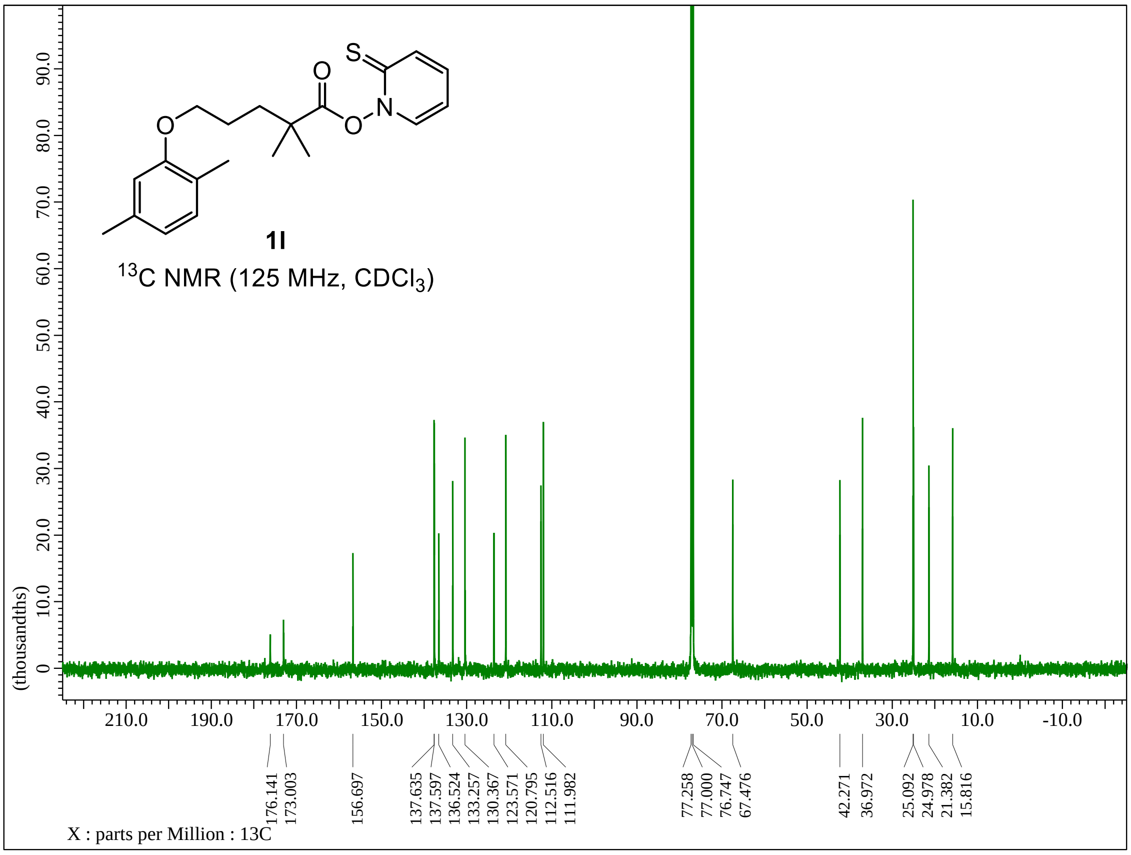
1l
^{13}C NMR (125 MHz, CDCl$_3$)
(thousandths)
X : parts per Million : 13C

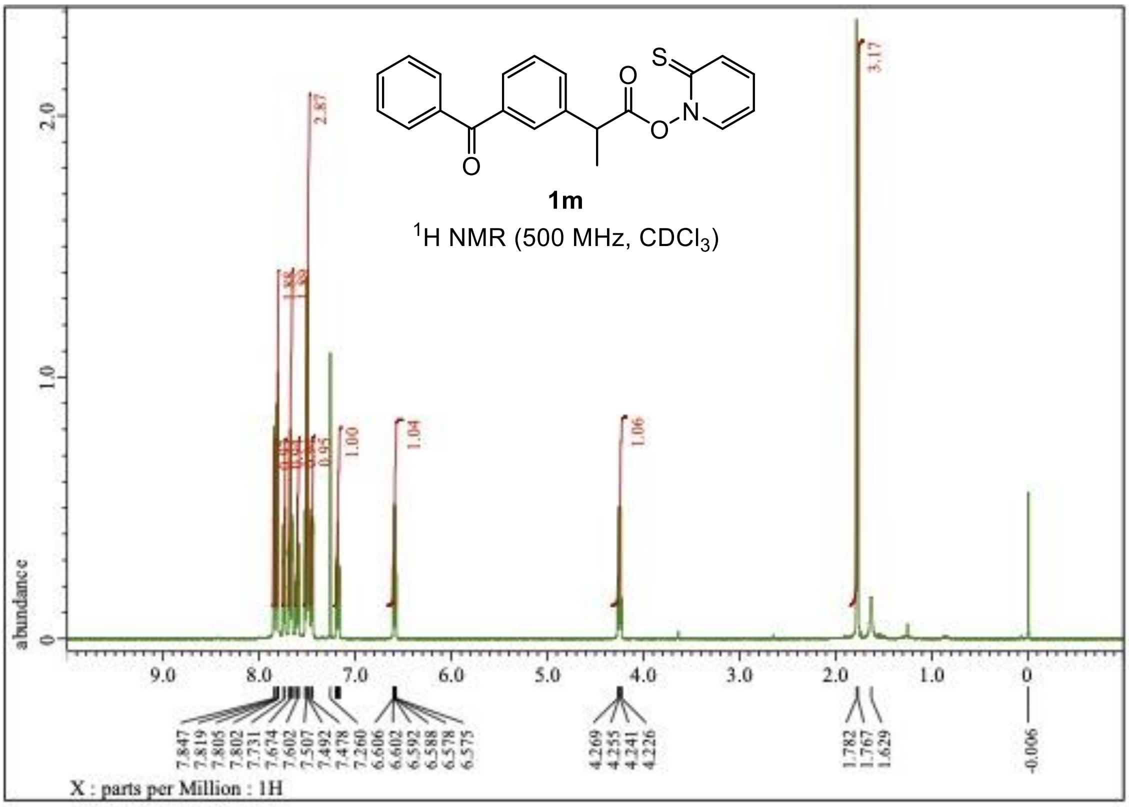
S
O
N
O
O
1m
1H NMR (500 MHz, CDCl3)
abundance
X : parts per Million : 1H
7.847
7.819
7.805
7.802
7.731
7.674
7.602
7.507
7.492
7.478
7.260
6.606
6.602
6.592
6.588
6.578
6.575
4.269
4.255
4.241
4.226
1.782
1.767
1.629
-0.006

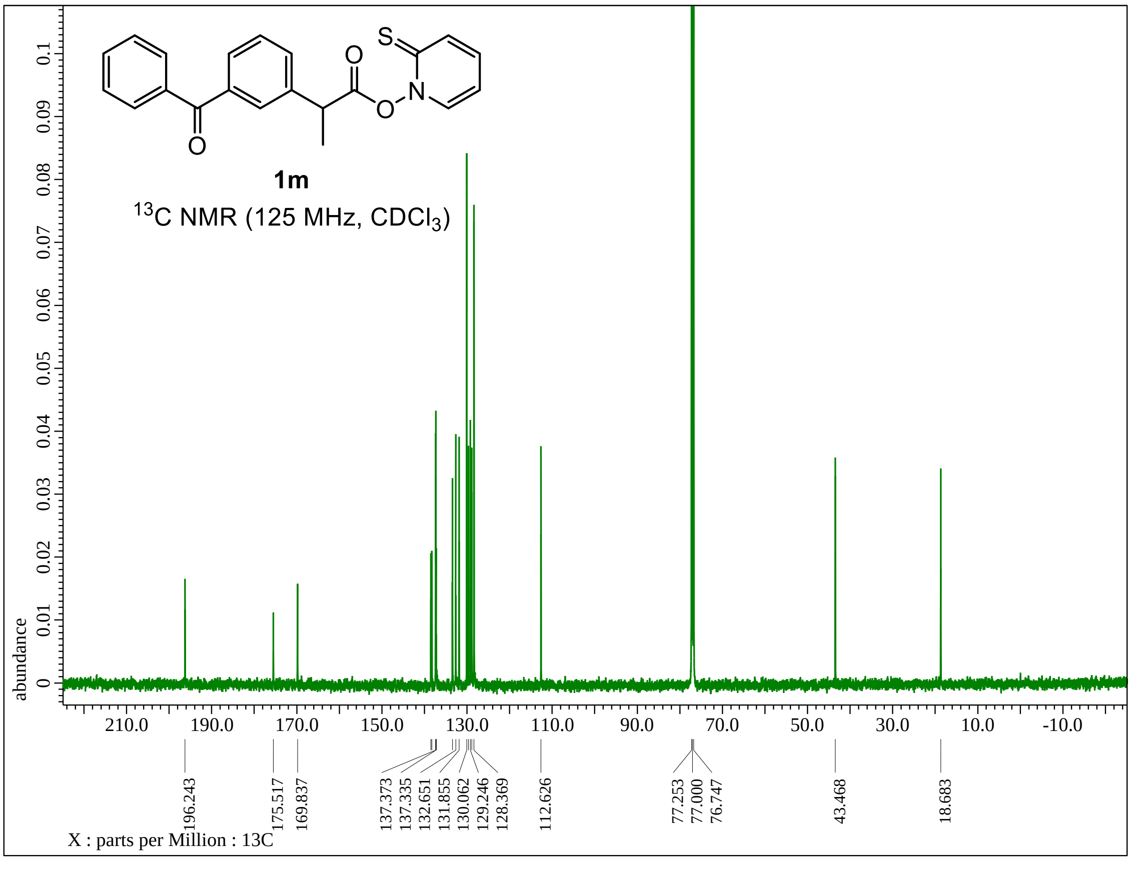
S
O
N
O
O
1m
13C NMR (125 MHz, CDCl3)
abundance
X : parts per Million : 13C
196.243
175.517
169.837
137.373
137.335
132.651
131.855
130.062
129.246
128.369
112.626
77.253
77.000
76.747
43.468
18.683

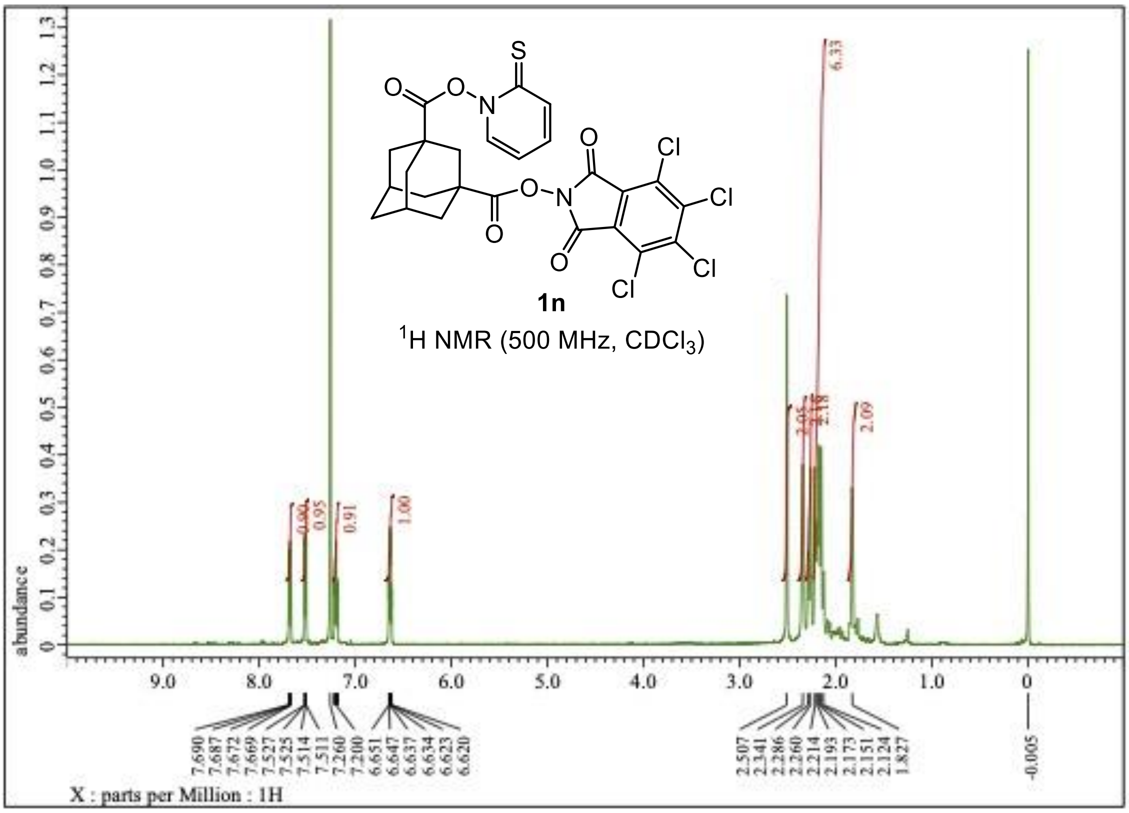

1n
^{1}H NMR (500 MHz, CDCl$_3$)
abundance
X : parts per Million : 1H

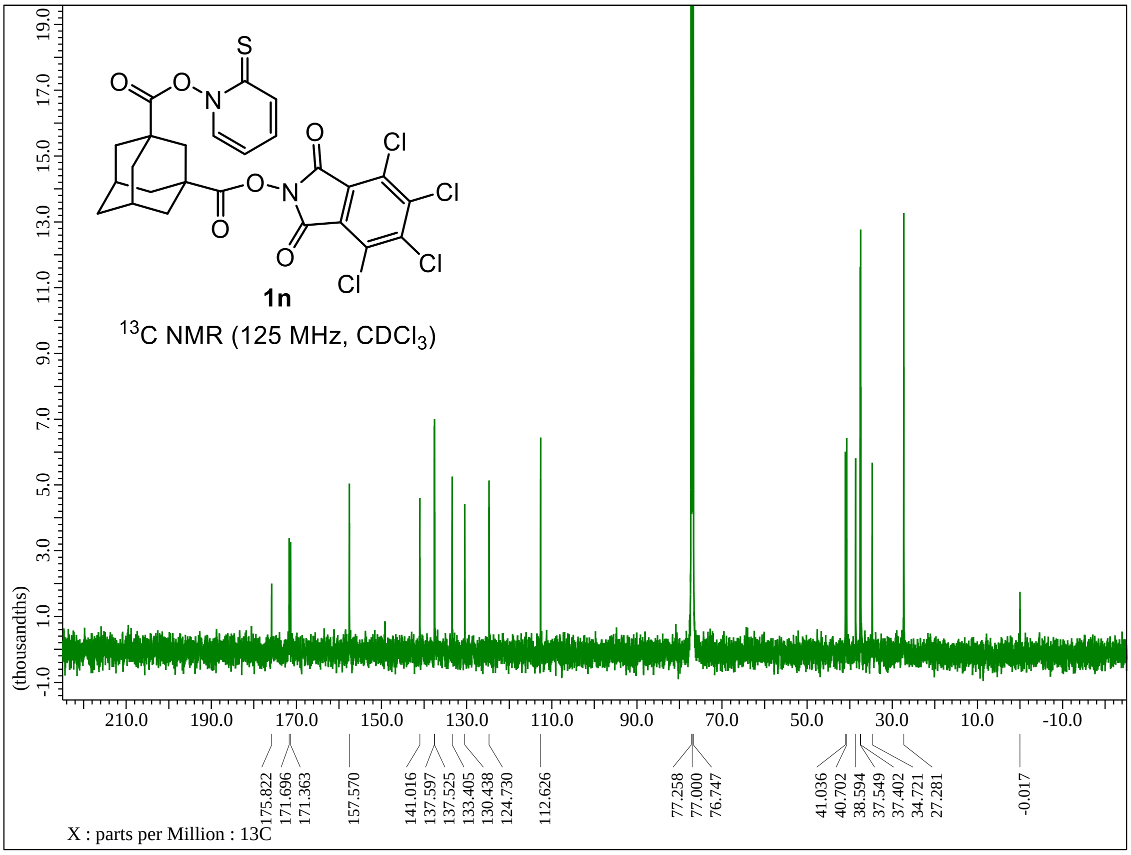

1n
^{13}C NMR (125 MHz, CDCl$_3$)
(thousandths)
X : parts per Million : 13C

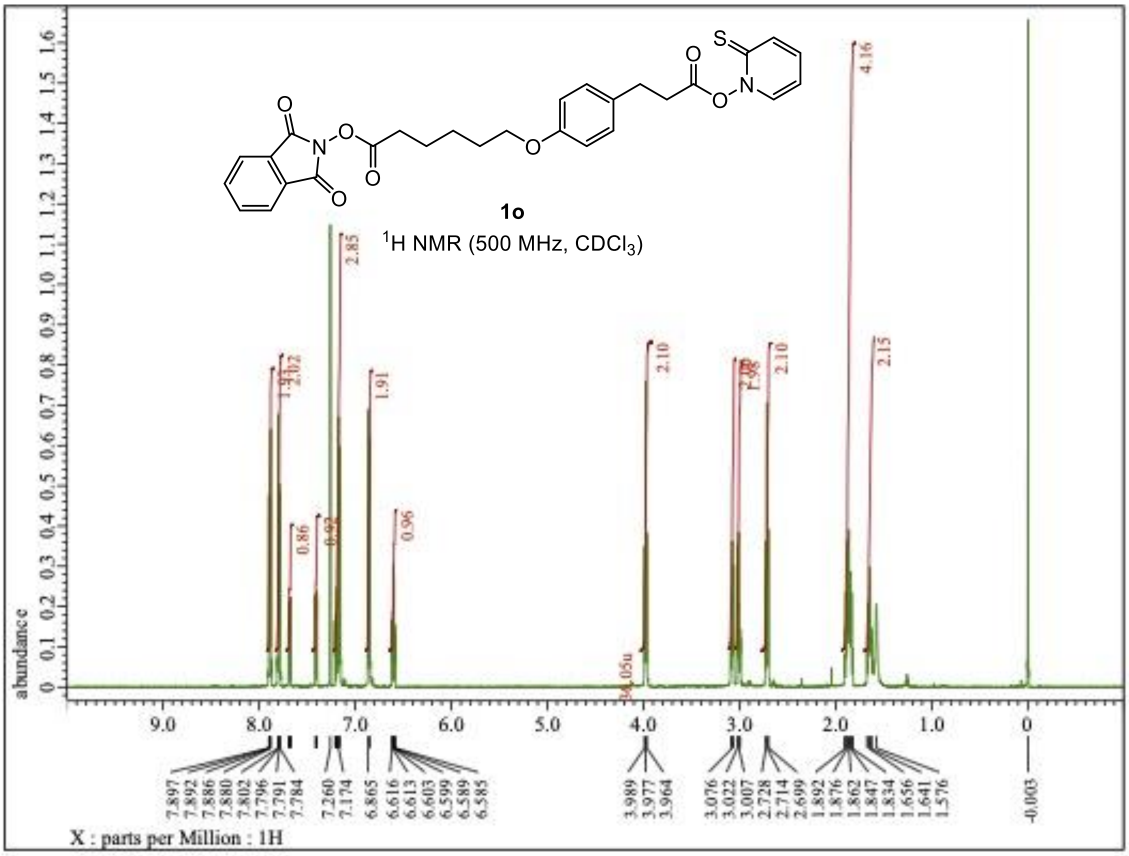
1o
^{1}H NMR (500 MHz, CDCl$_3$)
X : parts per Million : 1H
abundance

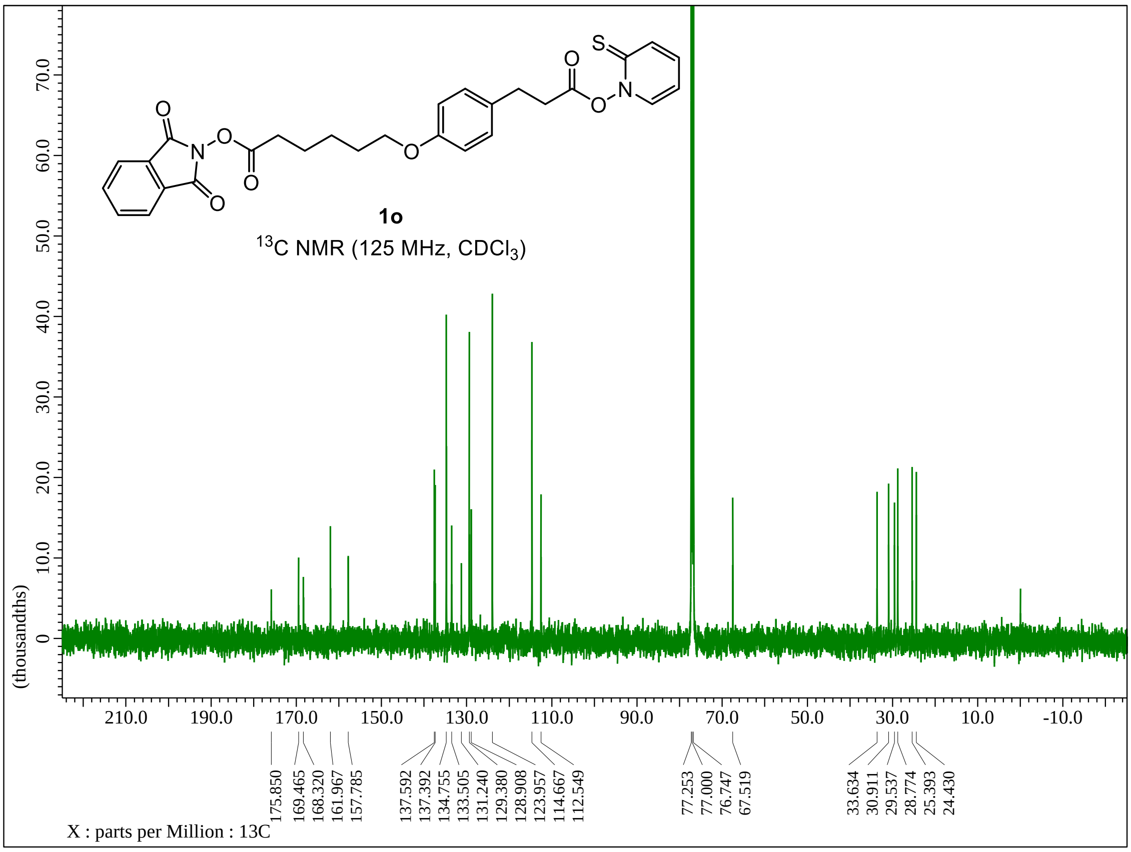
1o
^{13}C NMR (125 MHz, CDCl$_3$)
X : parts per Million : 13C
(thousandths)

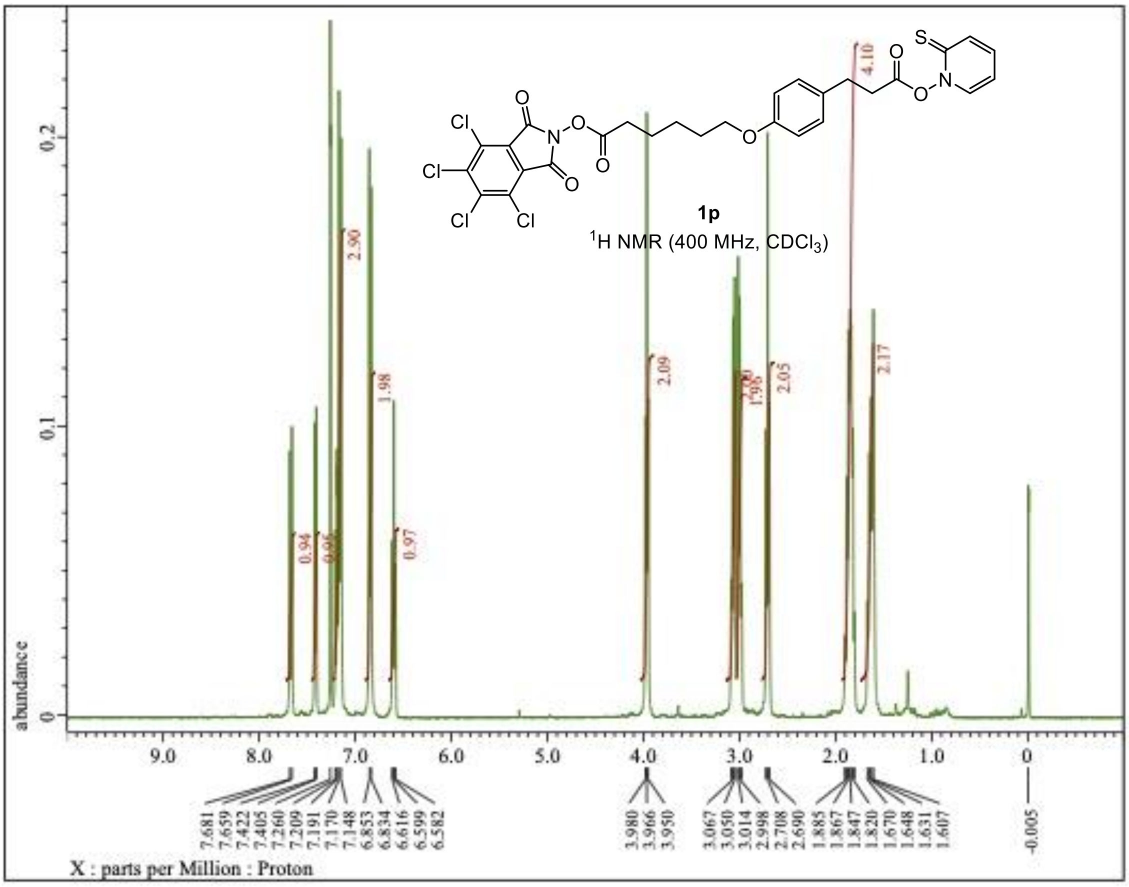
1p
1H NMR (400 MHz, CDCl3)
X : parts per Million : Proton
abundance

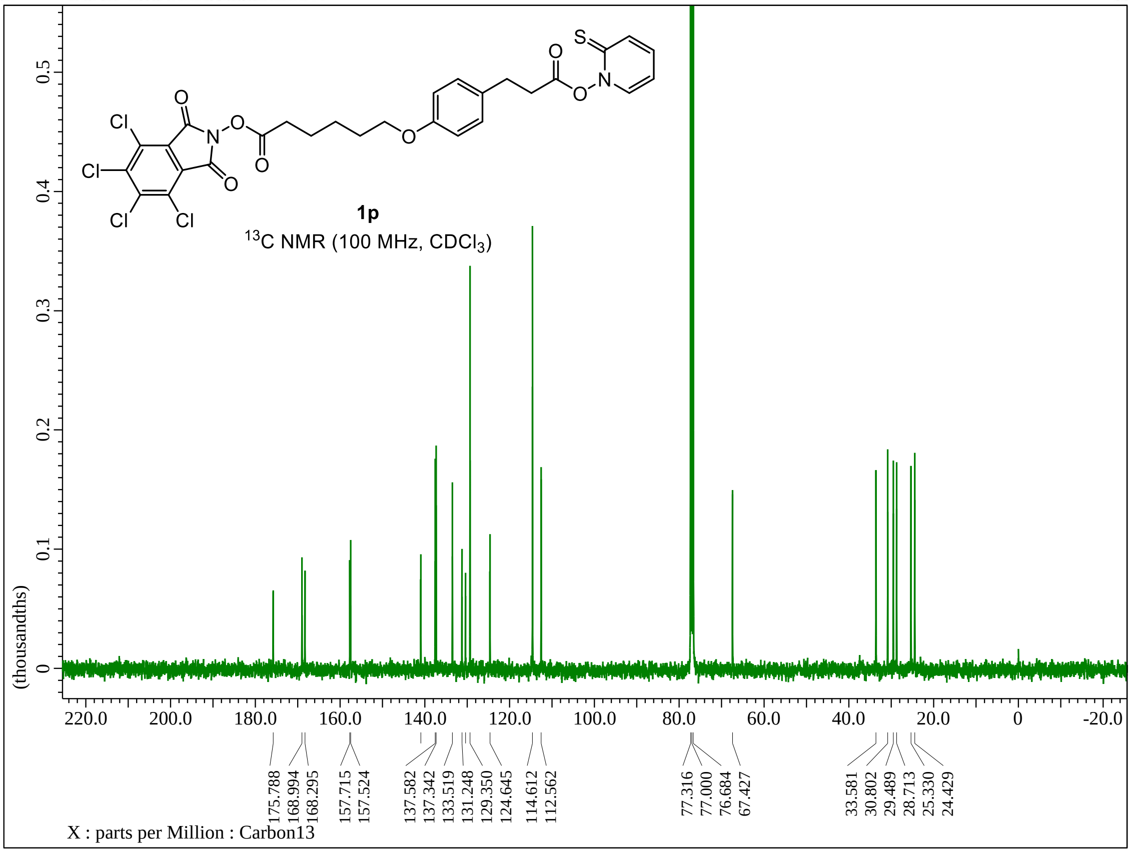
1p
13C NMR (100 MHz, CDCl3)
X : parts per Million : Carbon13
(thousandths)

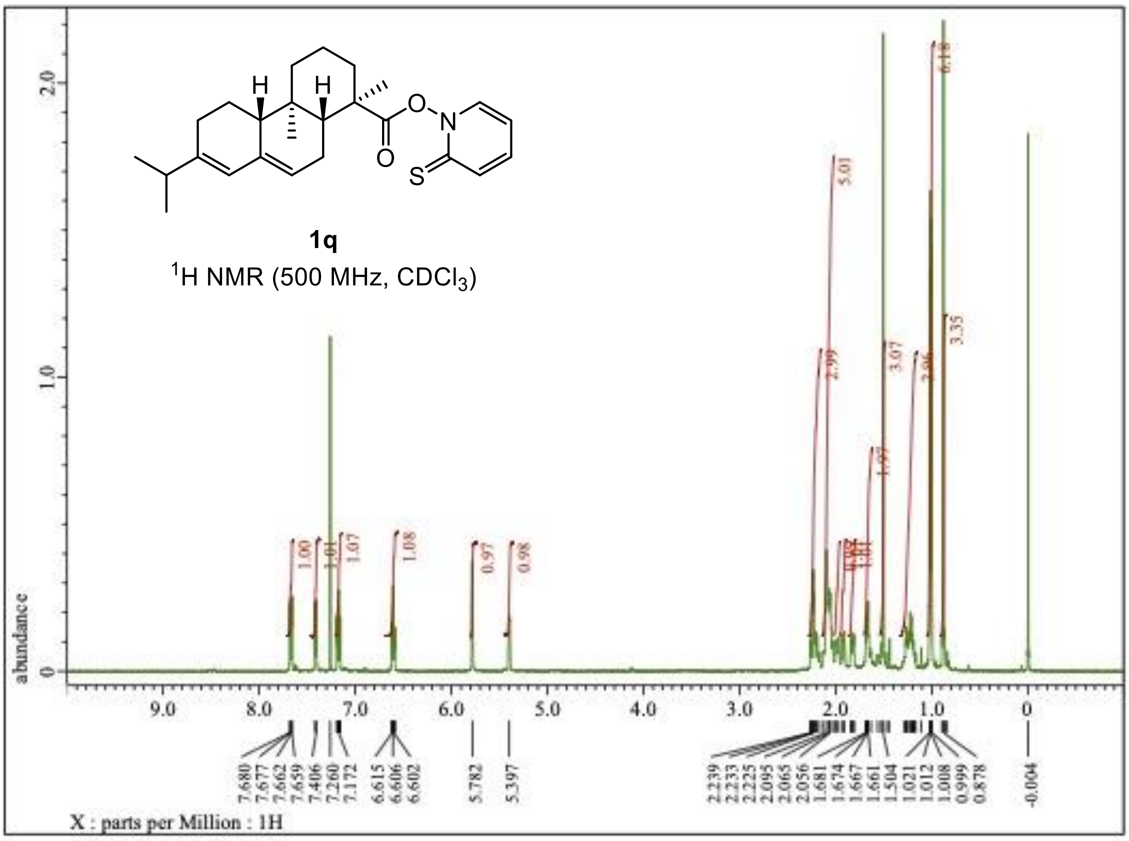
1q
¹H NMR (500 MHz, CDCl3)
abundance
X : parts per Million : 1H
7.680
7.677
7.662
7.659
7.406
7.260
7.172
6.615
6.606
6.602
5.782
5.397
2.239
2.233
2.225
2.095
2.065
2.056
1.681
1.674
1.667
1.661
1.504
1.021
1.012
1.008
0.999
0.878
-0.004

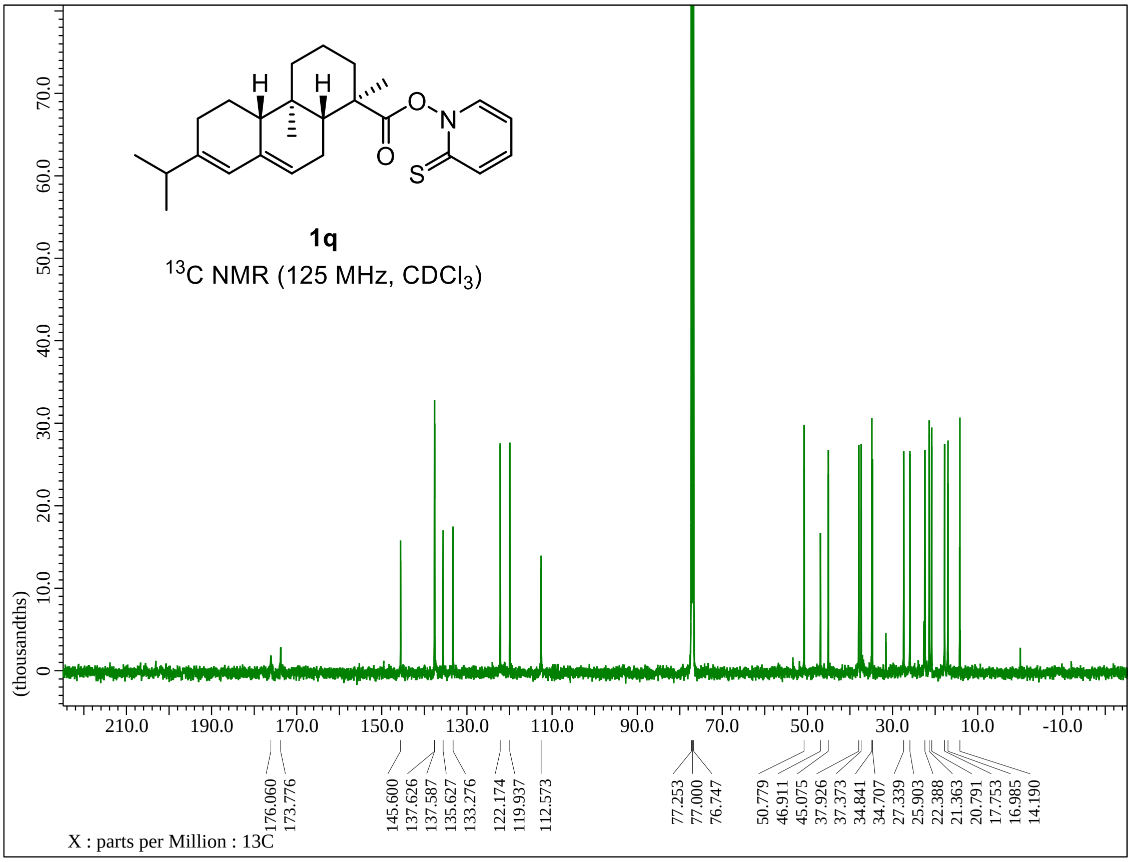
1q
¹³C NMR (125 MHz, CDCl3)
(thousandths)
X : parts per Million : 13C
176.060
173.776
145.600
137.626
137.587
135.627
133.276
122.174
119.937
112.573
77.253
77.000
76.747
50.779
46.911
45.075
37.926
37.373
34.841
34.707
27.339
25.903
22.388
21.363
20.791
17.753
16.985
14.190

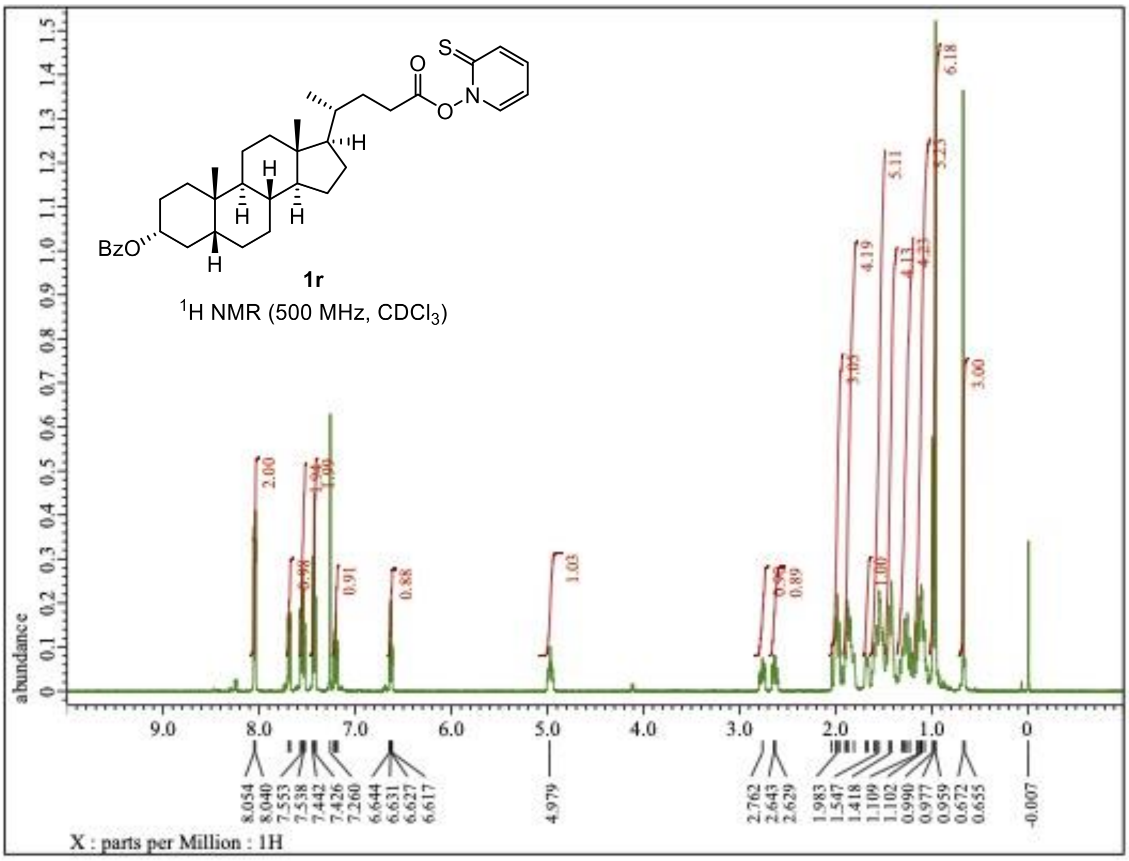
BzO
1r
^{1}H NMR (500 MHz, CDCl$_3$)
abundance
X : parts per Million : 1H

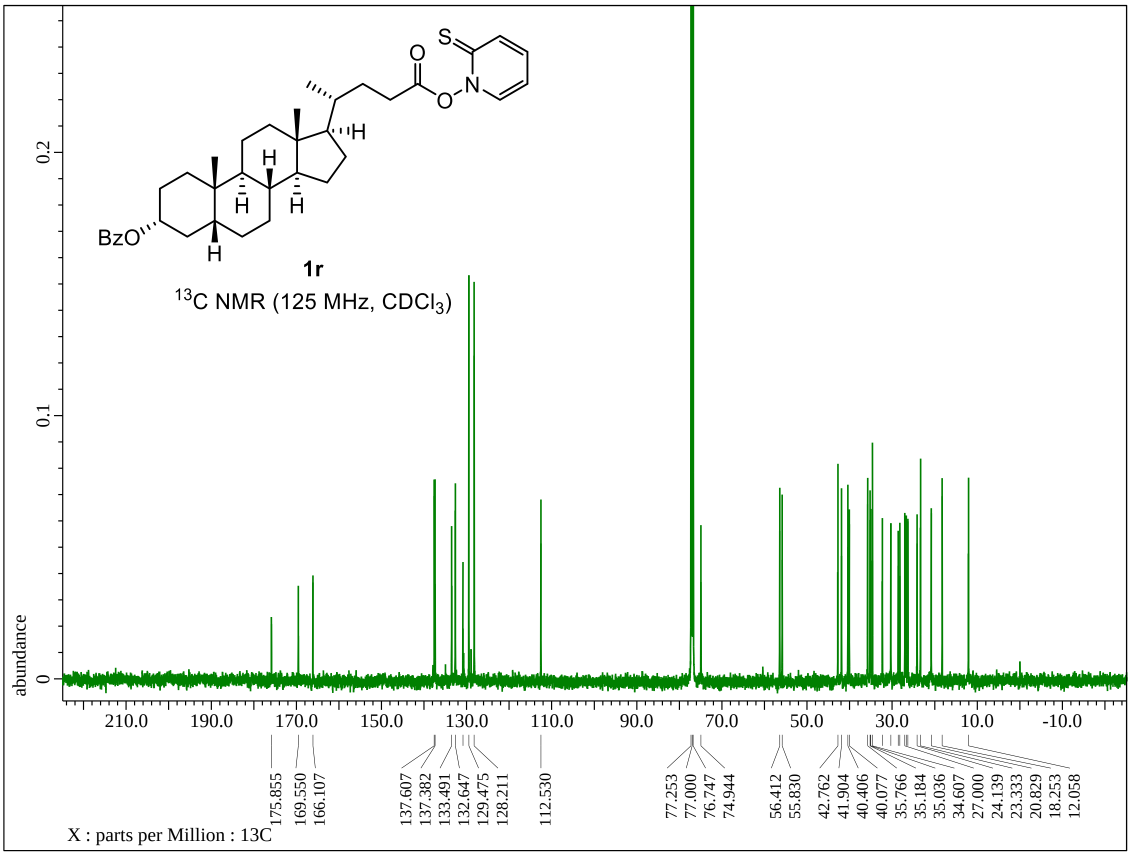
BzO
1r
^{13}C NMR (125 MHz, CDCl$_3$)
abundance
X : parts per Million : 13C

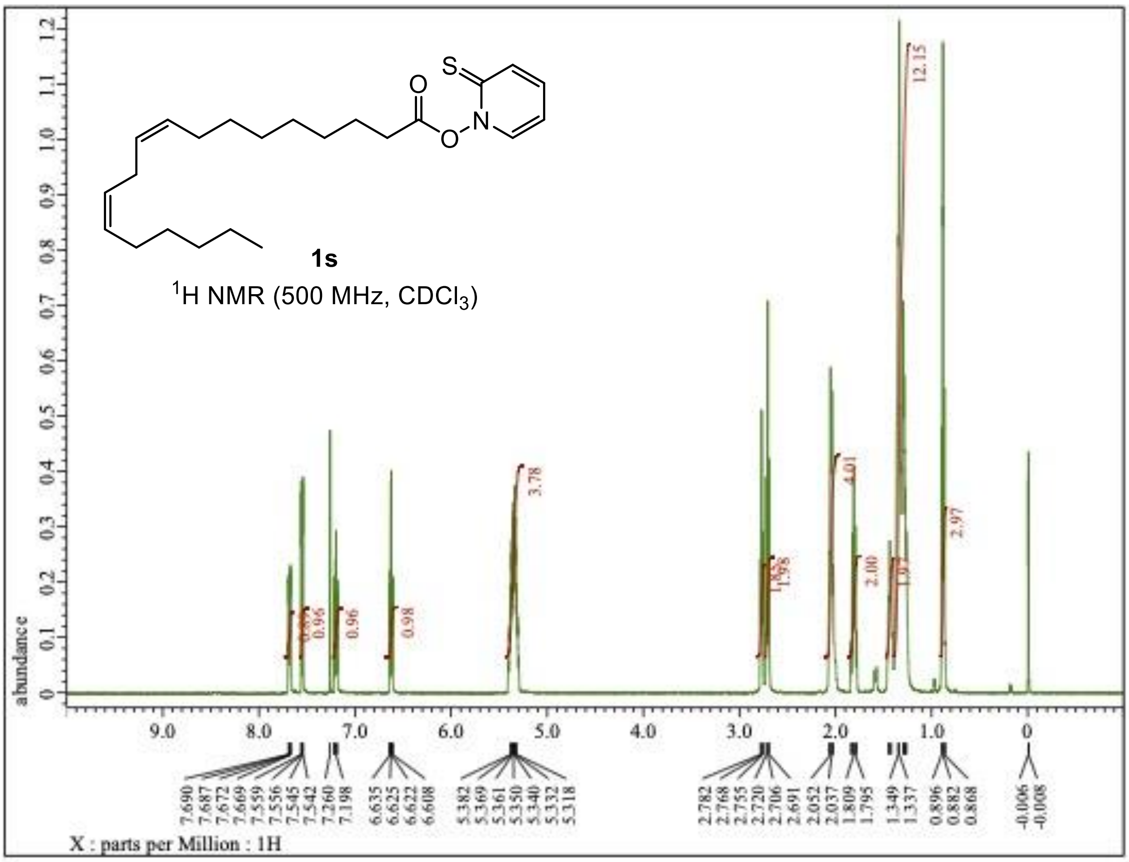
1s
¹H NMR (500 MHz, CDCl₃)
X : parts per Million : 1H
abundance

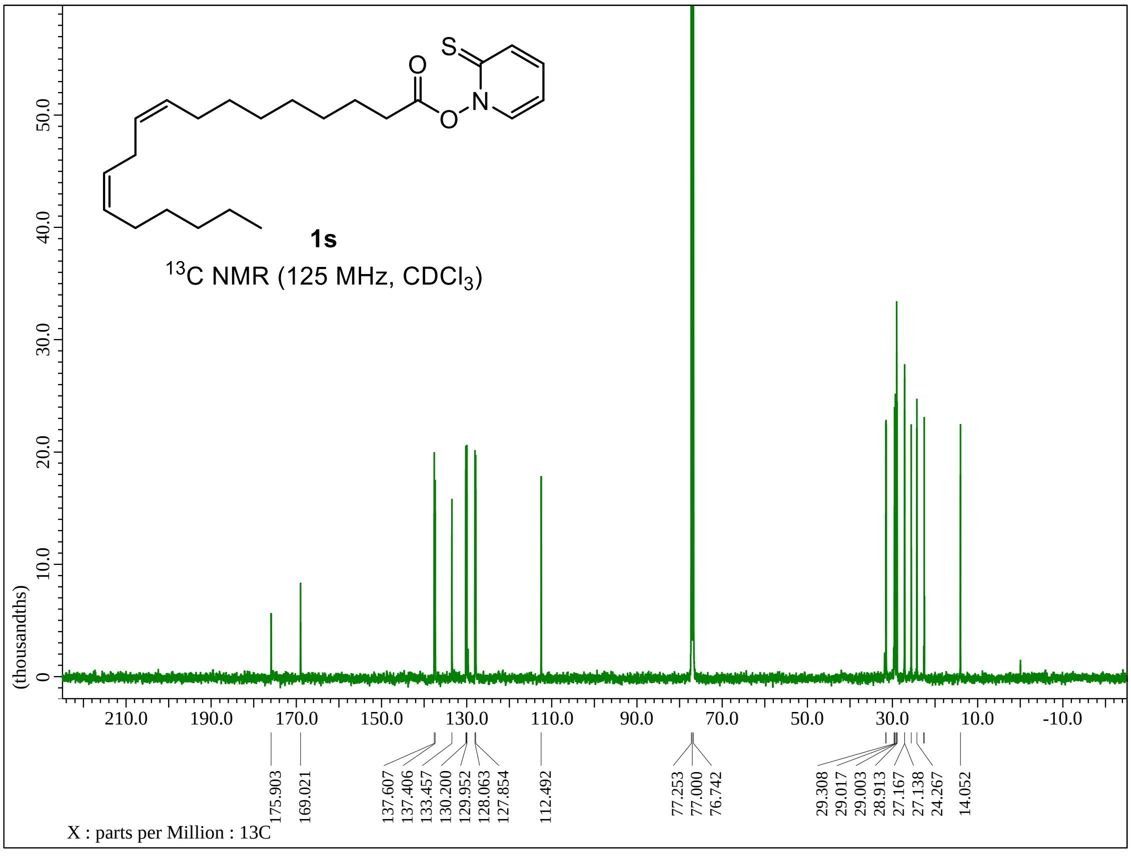
1s
¹³C NMR (125 MHz, CDCl₃)
X : parts per Million : 13C
(thousandths)

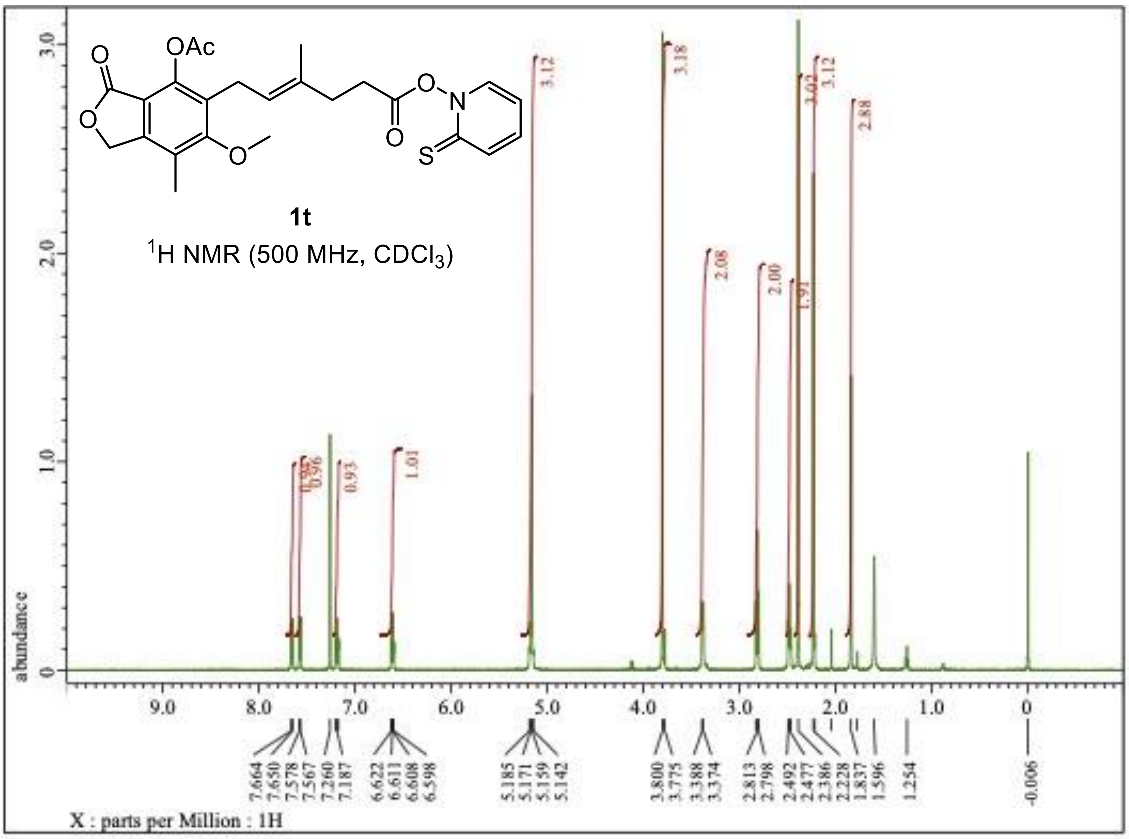
OAc
1t
1H NMR (500 MHz, CDCl3)
abundance
X : parts per Million : 1H
7.664 7.650 7.578 7.567 7.260 7.187 6.622 6.611 6.608 6.598 5.185 5.171 5.159 5.142 3.800 3.775 3.388 3.374 2.813 2.798 2.492 2.477 2.386 2.228 1.837 1.596 1.254 -0.006

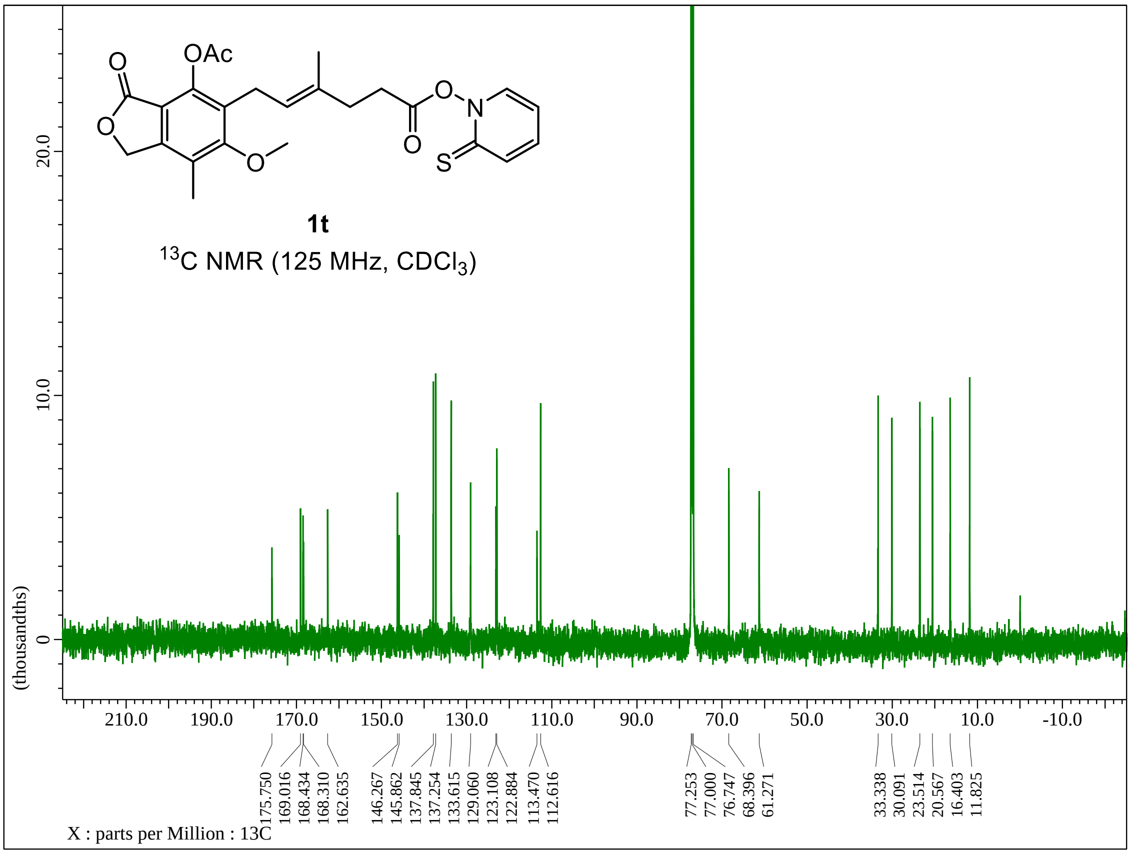
OAc
1t
13C NMR (125 MHz, CDCl3)
(thousandths)
X : parts per Million : 13C
175.750 169.016 168.434 168.310 162.635 146.267 145.862 137.845 137.254 133.615 129.060 123.108 122.884 113.470 112.616 77.253 77.000 76.747 68.396 61.271 33.338 30.091 23.514 20.567 16.403 11.825

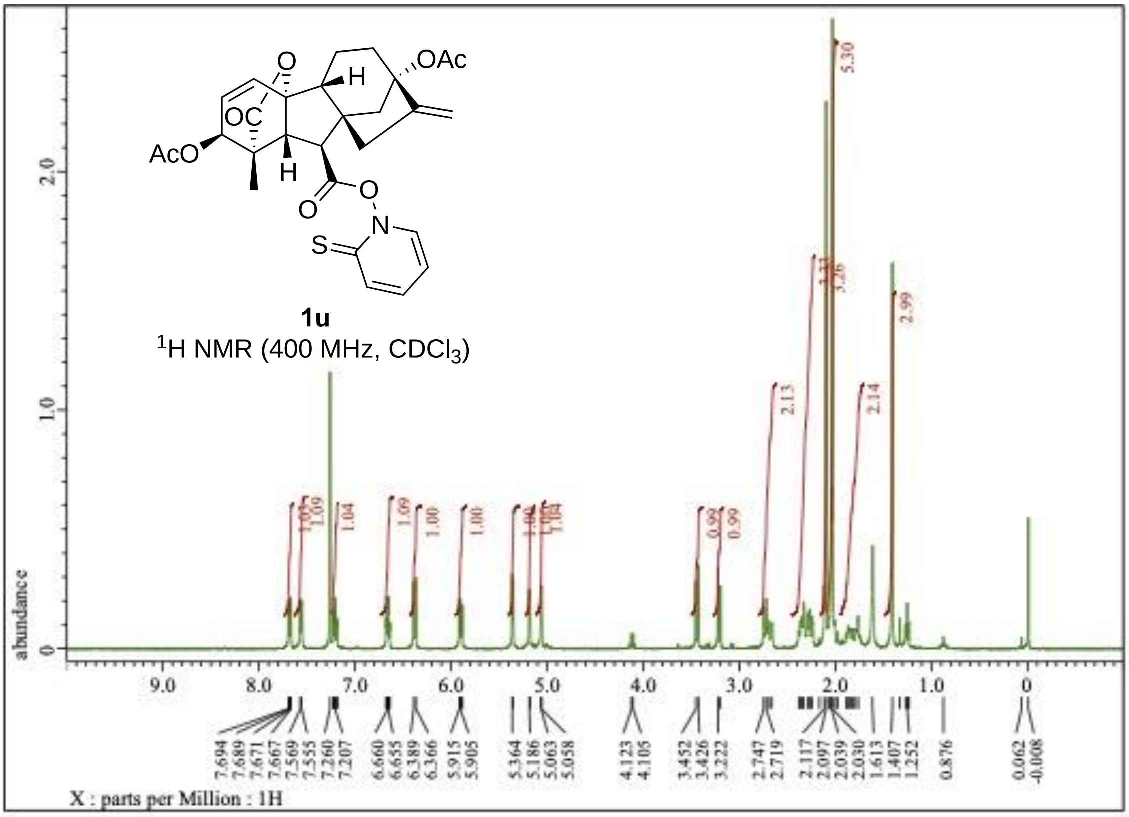
1u
^{1}H NMR (400 MHz, CDCl$_3$)
abundance
X : parts per Million : 1H

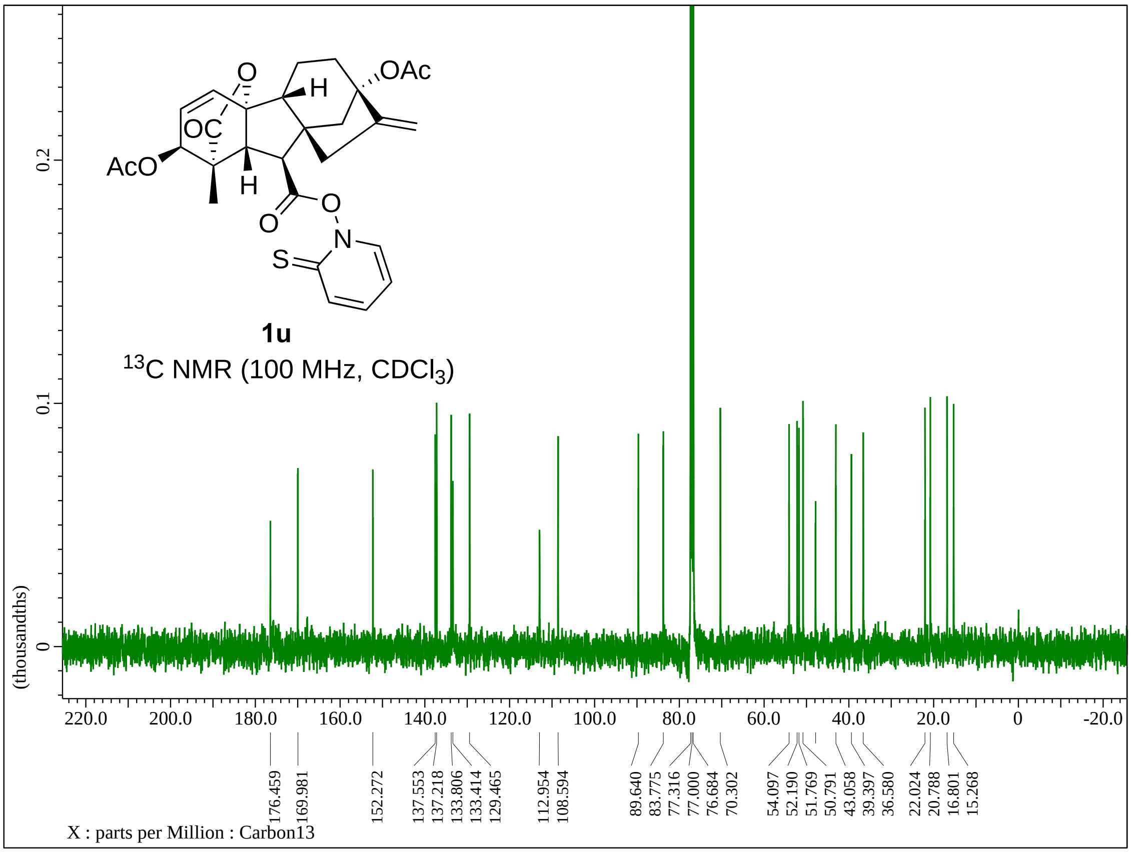
1u
^{13}C NMR (100 MHz, CDCl$_3$)
(thousandths)
X : parts per Million : Carbon13

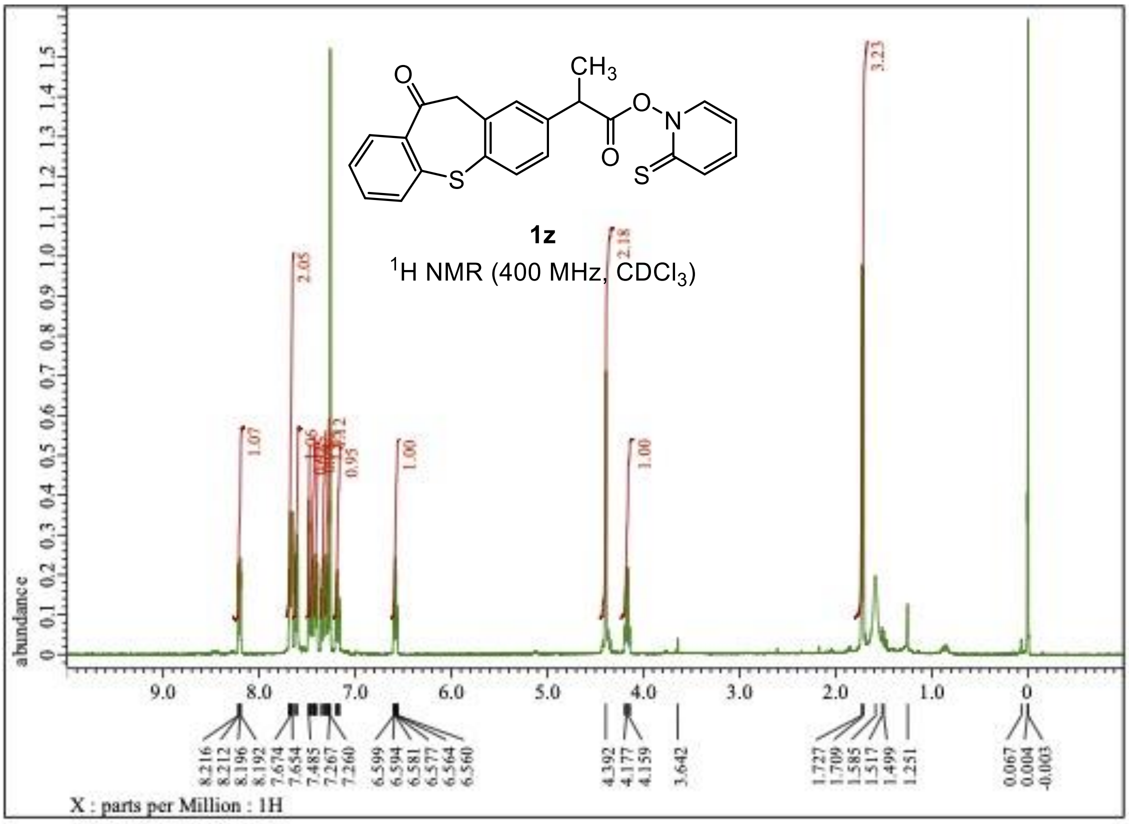
1z
1H NMR (400 MHz, CDCl3)
abundance
X : parts per Million : 1H

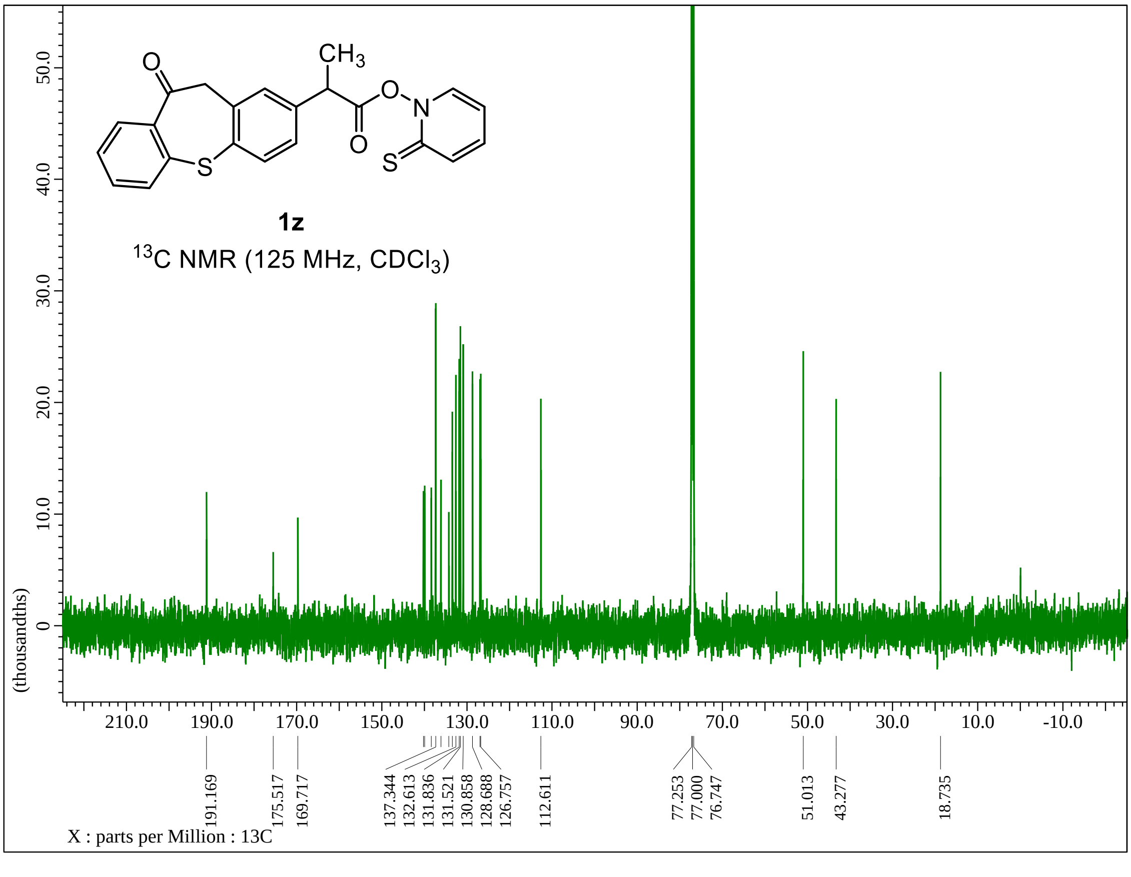
1z
13C NMR (125 MHz, CDCl3)
(thousandths)
X : parts per Million : 13C

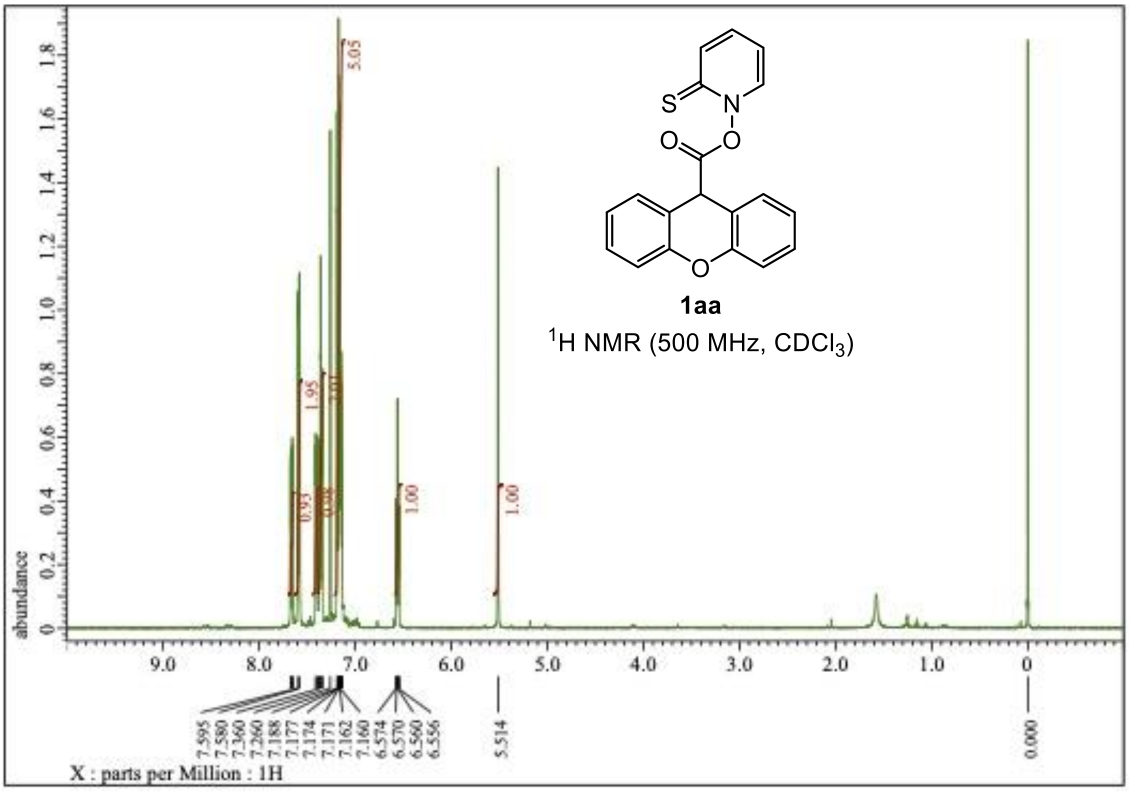
1aa
^{1}H NMR (500 MHz, CDCl$_3$)
X : parts per Million : 1H
abundance

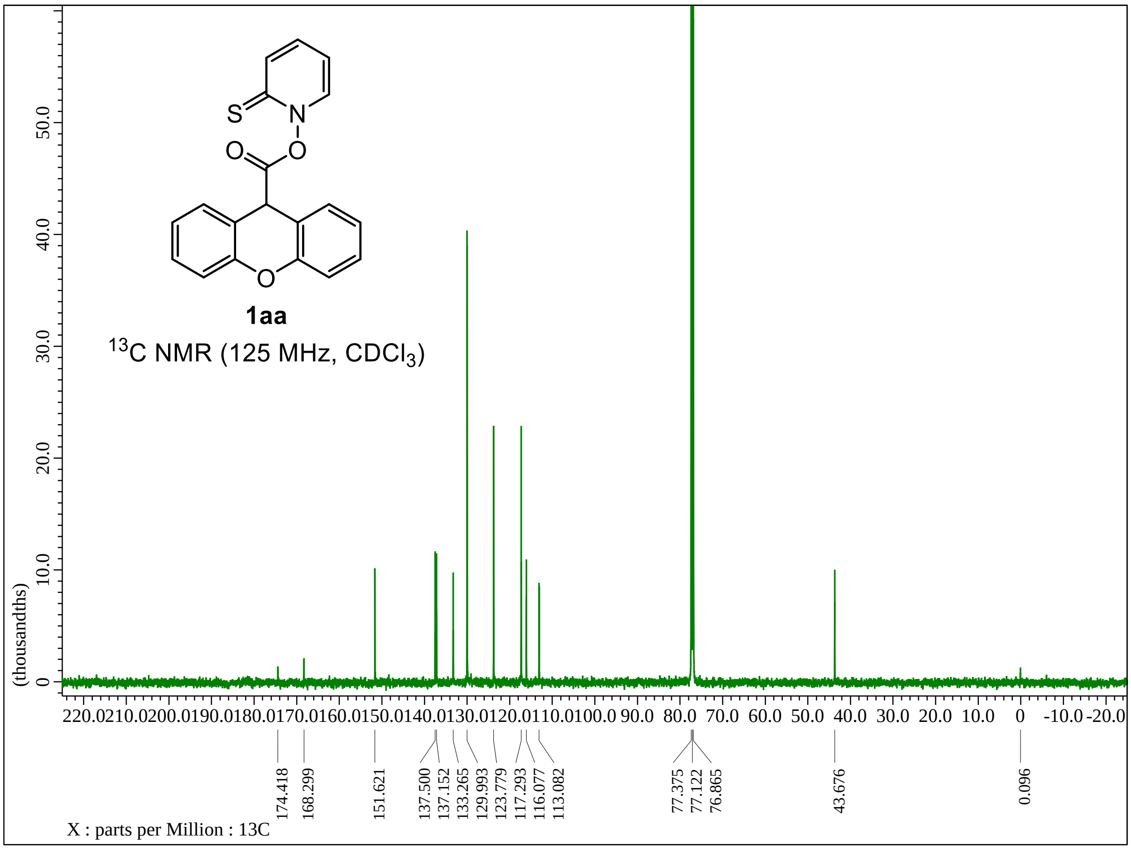
1aa
^{13}C NMR (125 MHz, CDCl$_3$)
X : parts per Million : 13C
(thousandths)

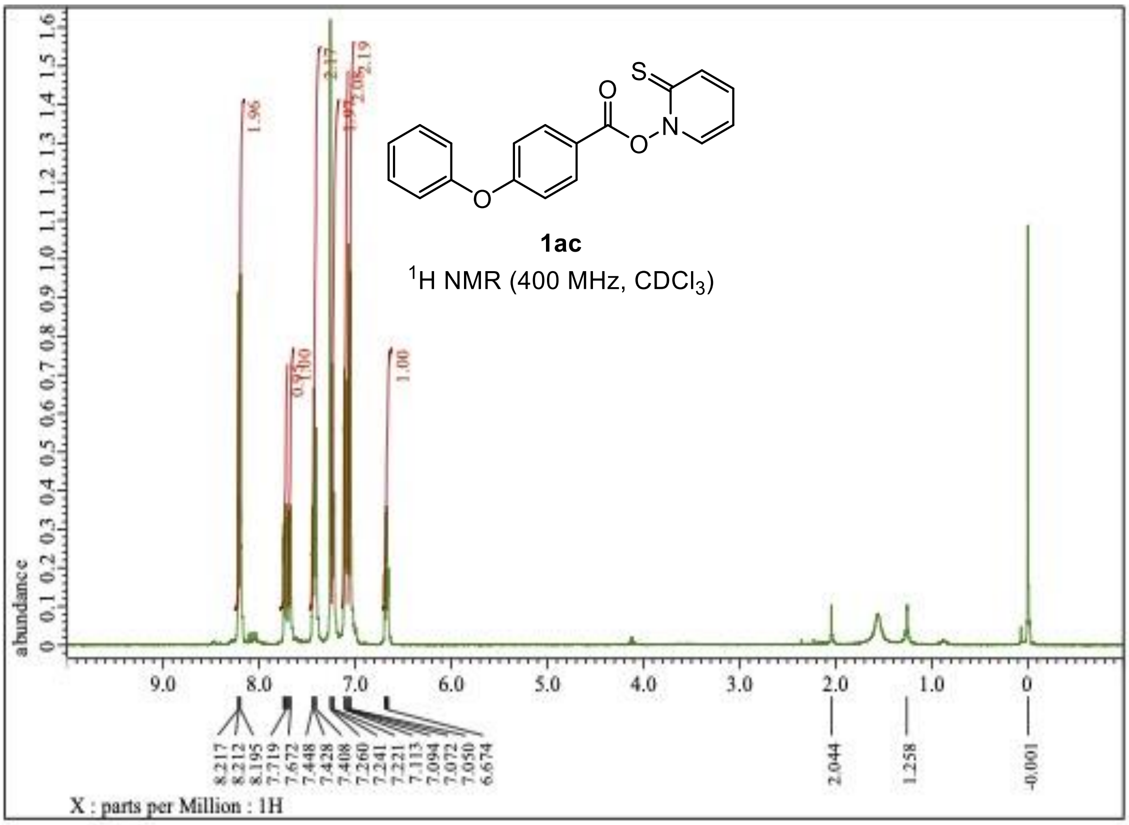

1ac
^{1}H NMR (400 MHz, CDCl$_3$)
abundance
X : parts per Million : 1H
8.217
8.212
8.195
7.719
7.672
7.448
7.428
7.408
7.260
7.241
7.221
7.113
7.094
7.072
7.050
6.674
2.044
1.258
-0.001

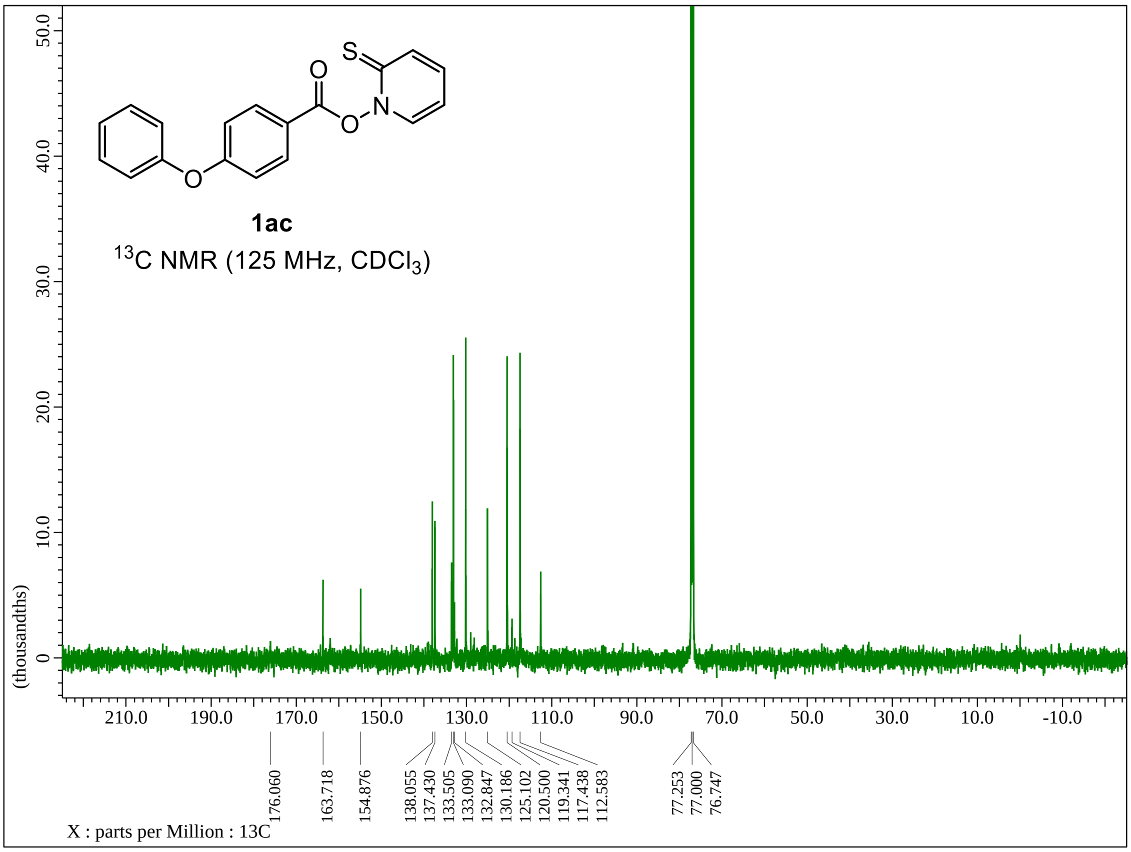

1ac
^{13}C NMR (125 MHz, CDCl$_3$)
(thousandths)
X : parts per Million : 13C
176.060
163.718
154.876
138.055
137.430
133.505
133.090
132.847
130.186
125.102
120.500
119.341
117.438
112.583
77.253
77.000
76.747

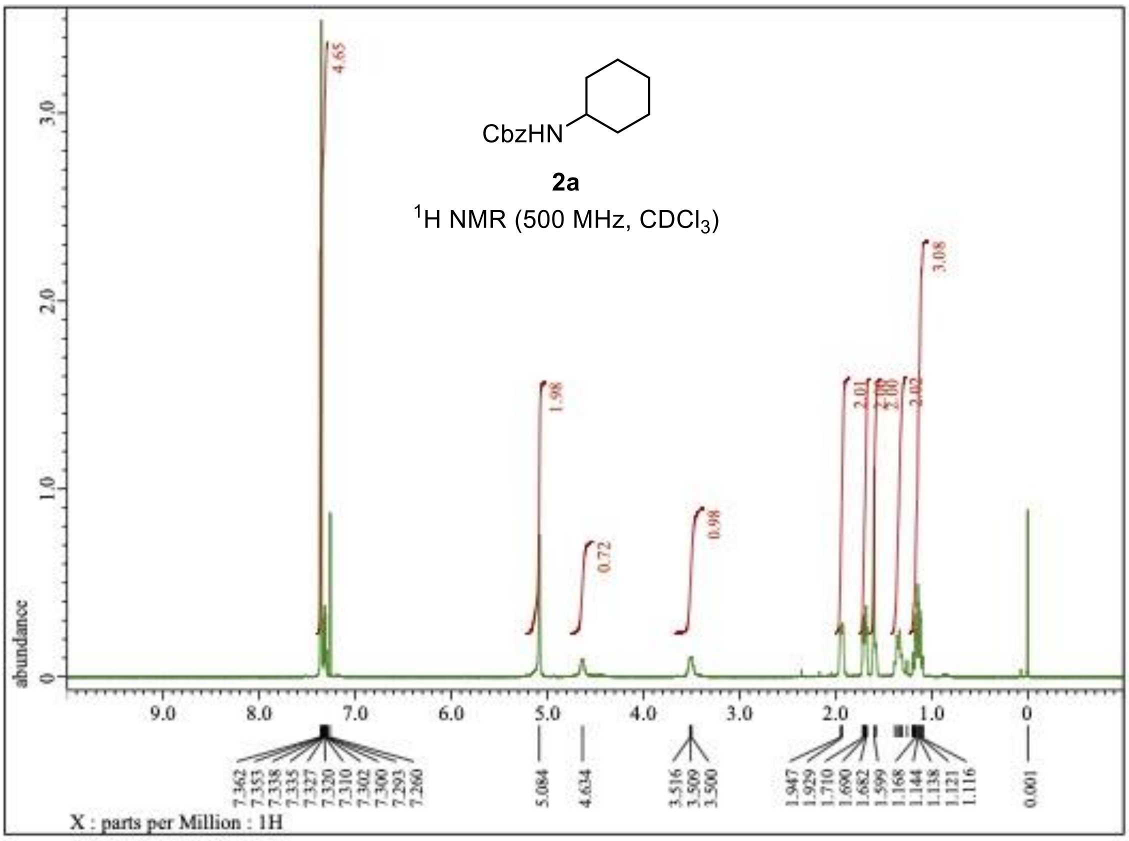
CbzHN
2a
1H NMR (500 MHz, CDCl3)
abundance
X : parts per Million : 1H

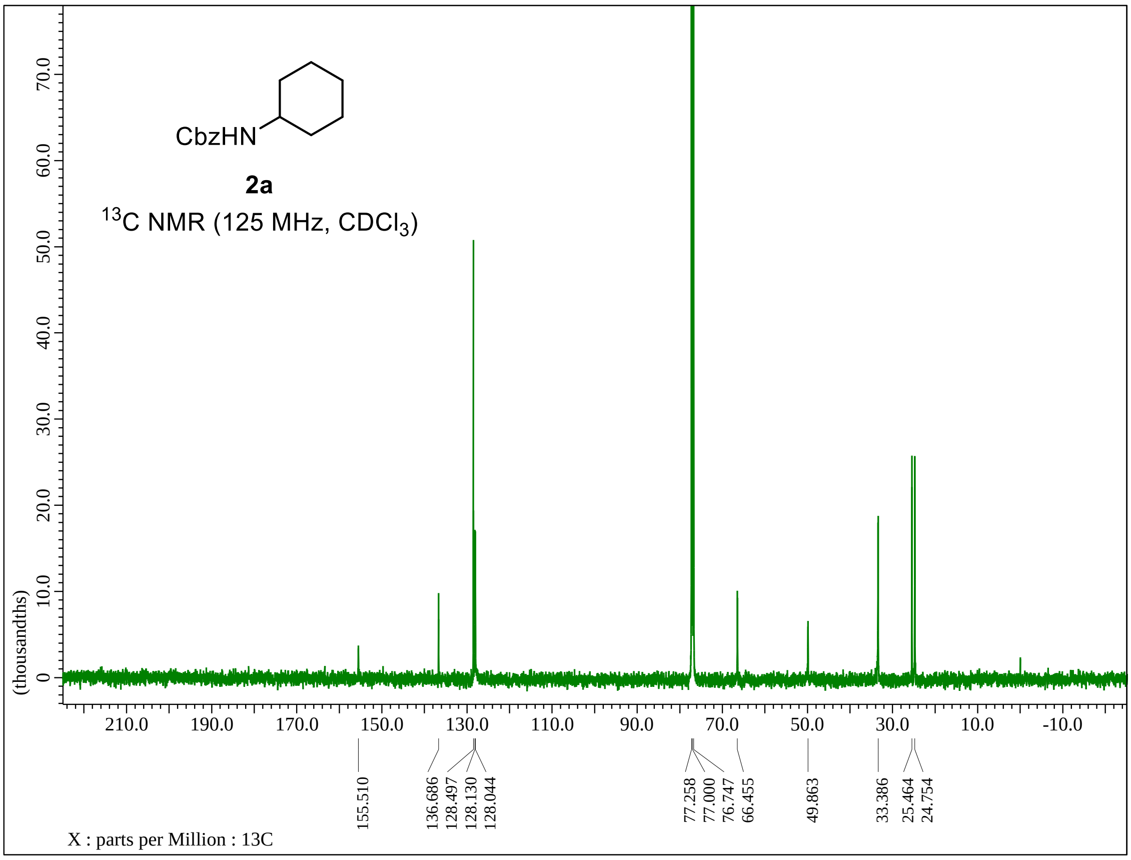
CbzHN
2a
13C NMR (125 MHz, CDCl3)
(thousandths)
X : parts per Million : 13C

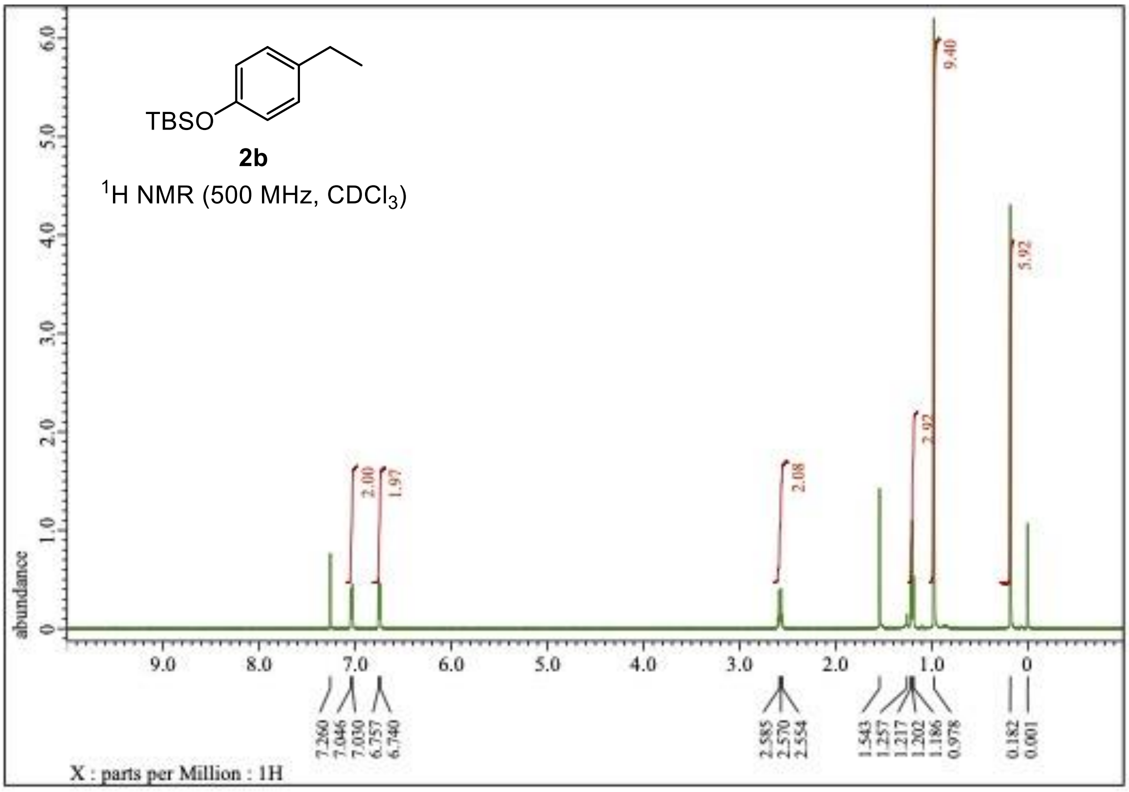
TBSO
2b
1H NMR (500 MHz, CDCl3)
abundance
X : parts per Million : 1H
9.40
5.92
2.92
2.08
2.00
1.97
7.260
7.046
7.030
6.757
6.740
2.585
2.570
2.554
1.543
1.257
1.217
1.202
1.186
0.978
0.182
0.001

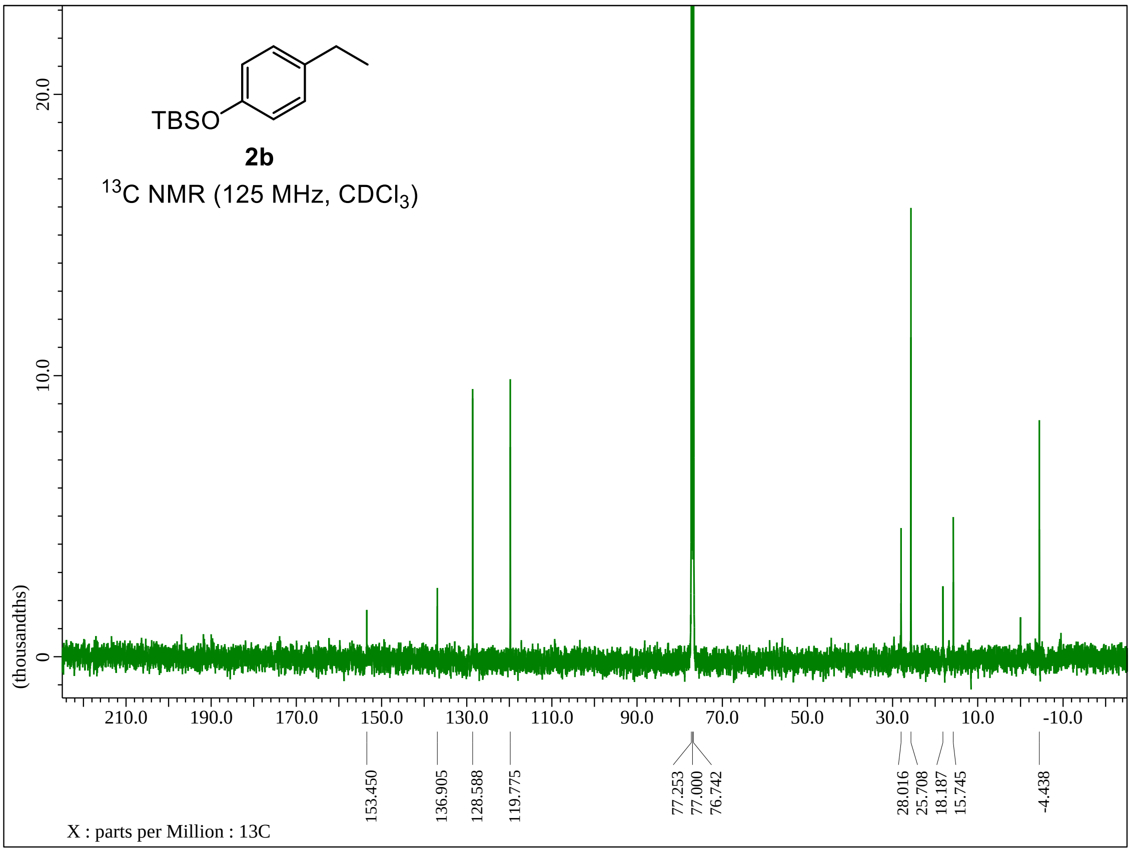
TBSO
2b
13C NMR (125 MHz, CDCl3)
(thousandths)
X : parts per Million : 13C
153.450
136.905
128.588
119.775
77.253
77.000
76.742
28.016
25.708
18.187
15.745
-4.438

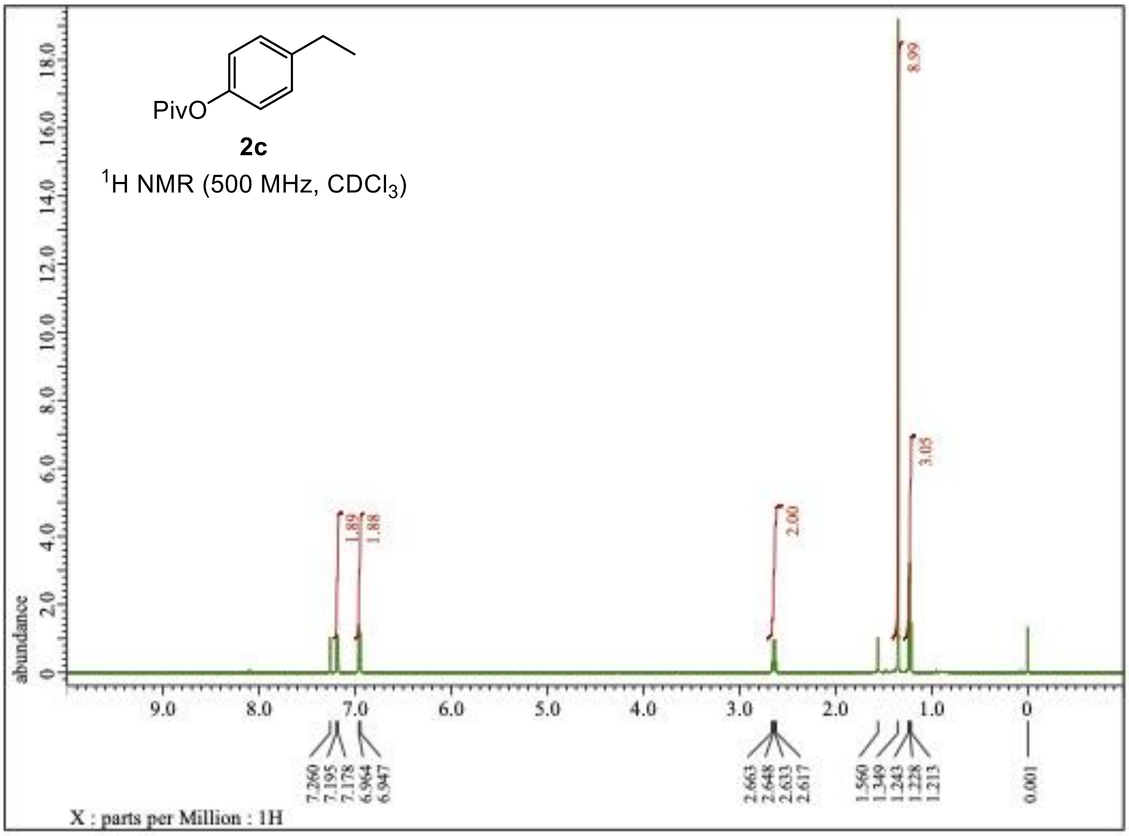
PivO
2c
¹H NMR (500 MHz, CDCl₃)
abundance
X : parts per Million : 1H
7.260
7.195
7.178
6.964
6.947
2.663
2.648
2.633
2.617
1.560
1.349
1.243
1.228
1.213
0.001
1.89
1.88
2.00
8.99
3.05

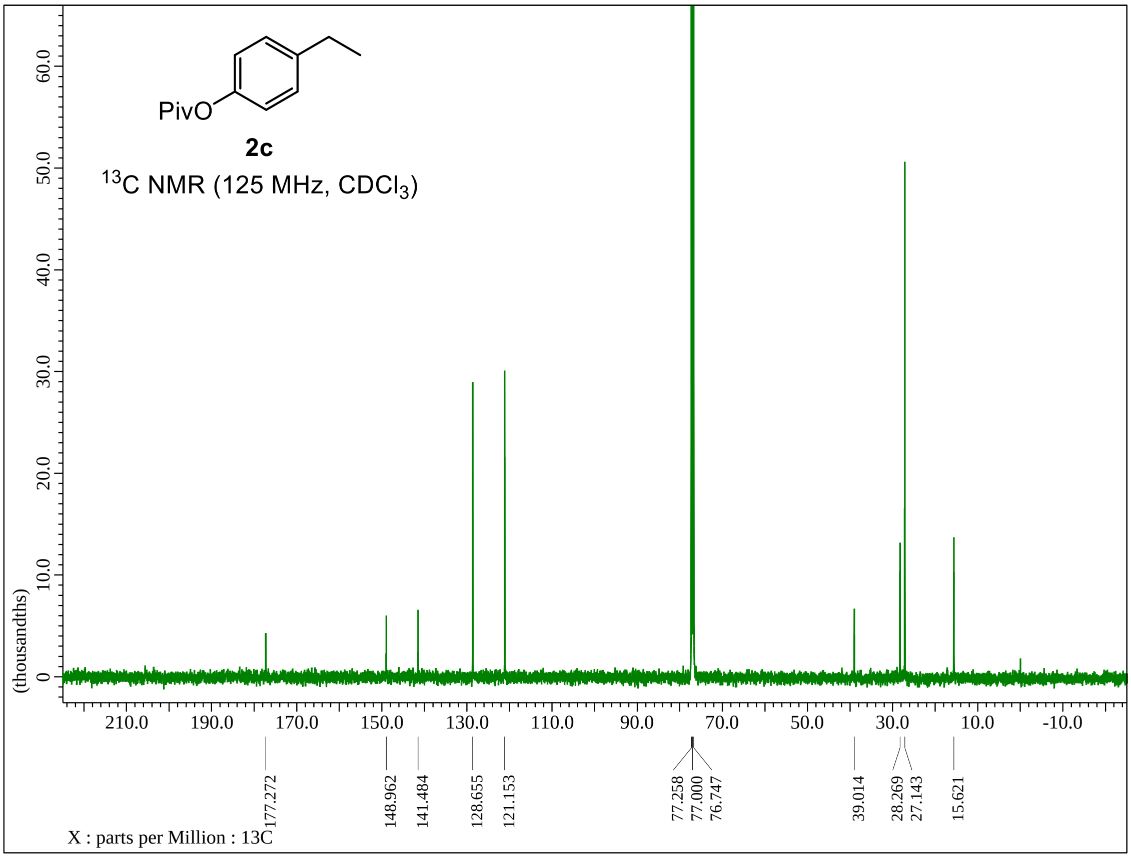
PivO
2c
¹³C NMR (125 MHz, CDCl₃)
(thousandths)
X : parts per Million : 13C
177.272
148.962
141.484
128.655
121.153
77.258
77.000
76.747
39.014
28.269
27.143
15.621

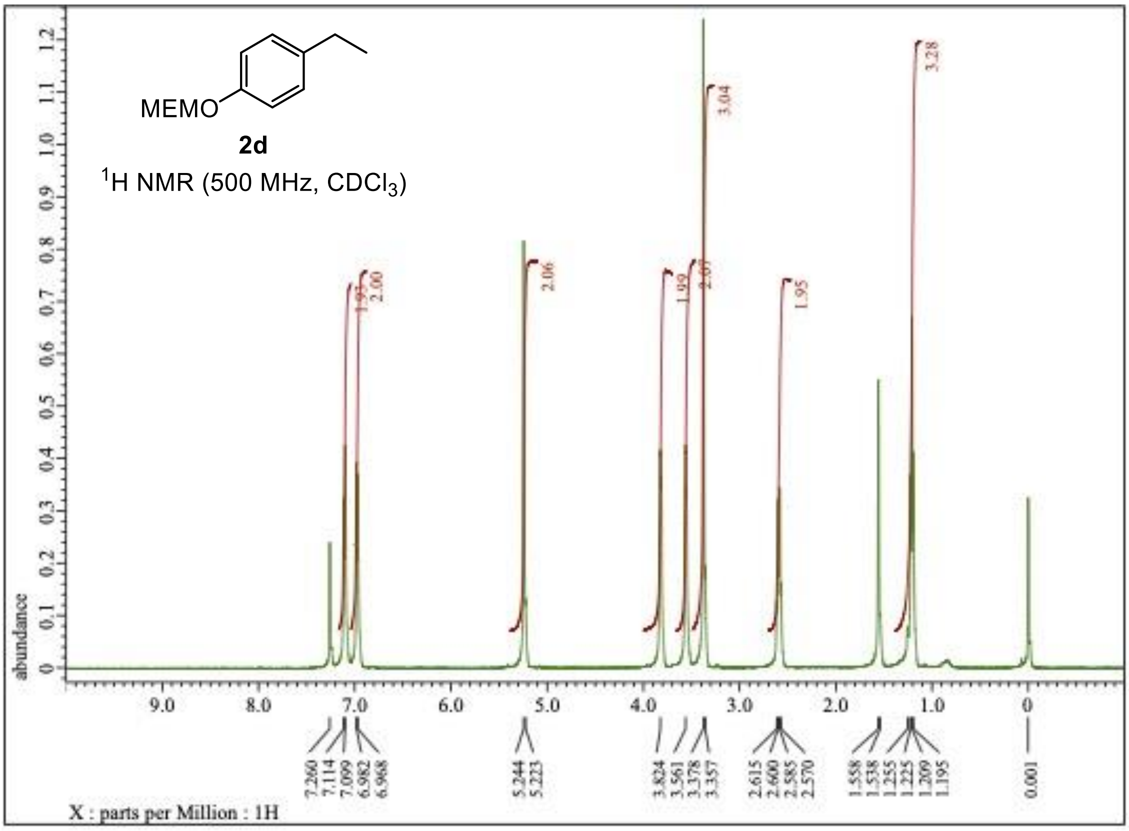
MEMO
2d
¹H NMR (500 MHz, CDCl₃)
X : parts per Million : 1H
abundance

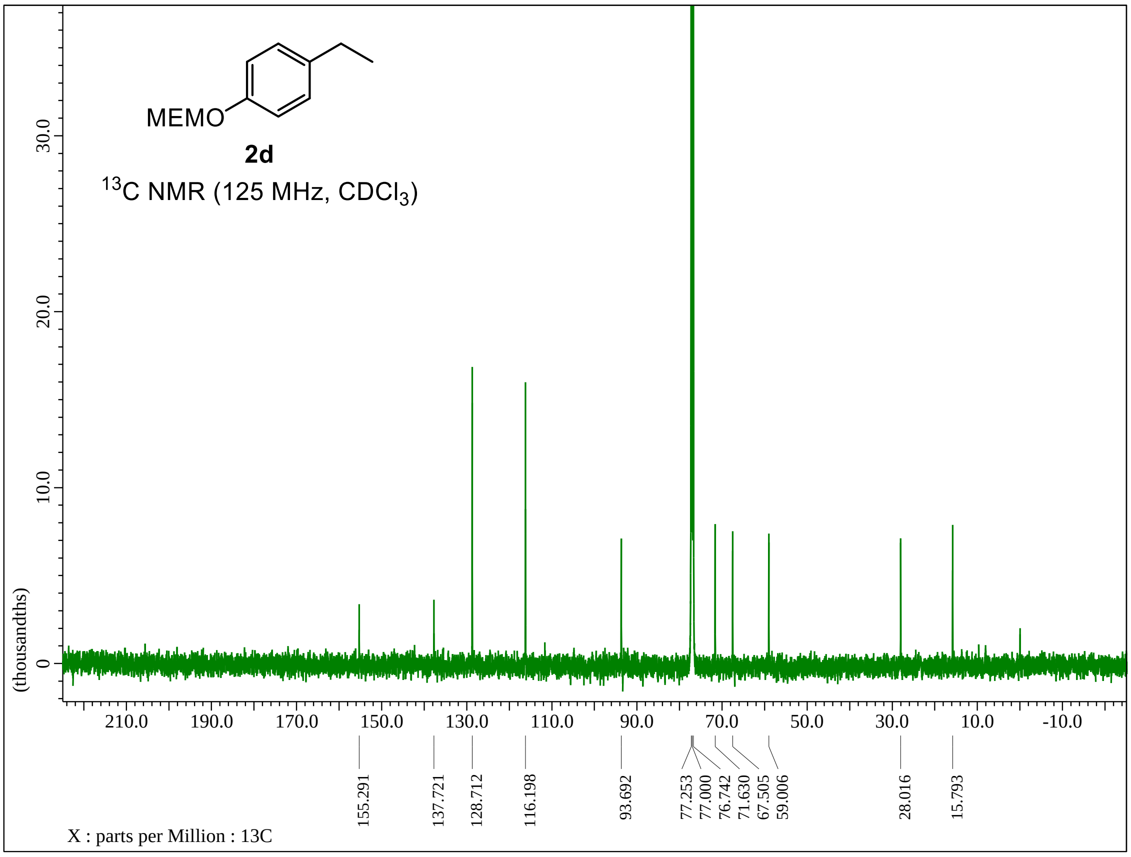
MEMO
2d
¹³C NMR (125 MHz, CDCl₃)
X : parts per Million : 13C
(thousandths)

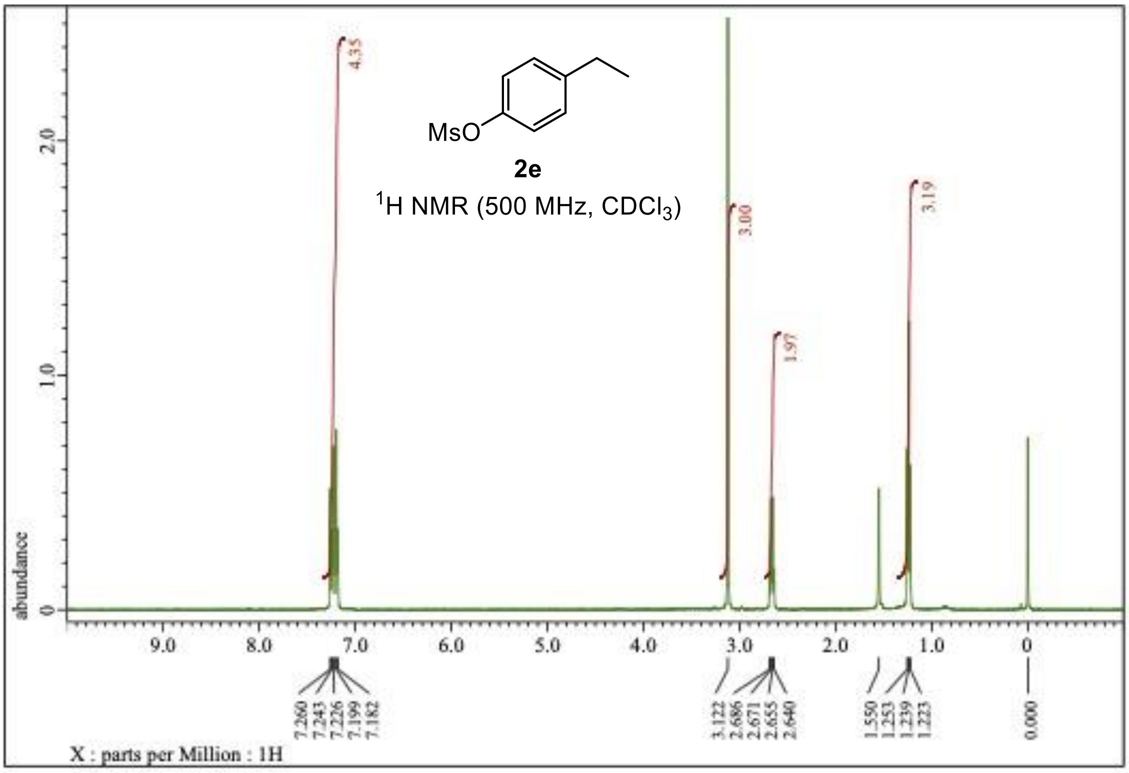
MsO
2e
1H NMR (500 MHz, CDCl3)
4.35
3.00
1.97
3.19
abundance
2.0
1.0
0
9.0
8.0
7.0
6.0
5.0
4.0
3.0
2.0
1.0
0
7.260
7.243
7.226
7.199
7.182
3.122
2.686
2.671
2.655
2.640
1.550
1.253
1.239
1.223
0.000
X : parts per Million : 1H

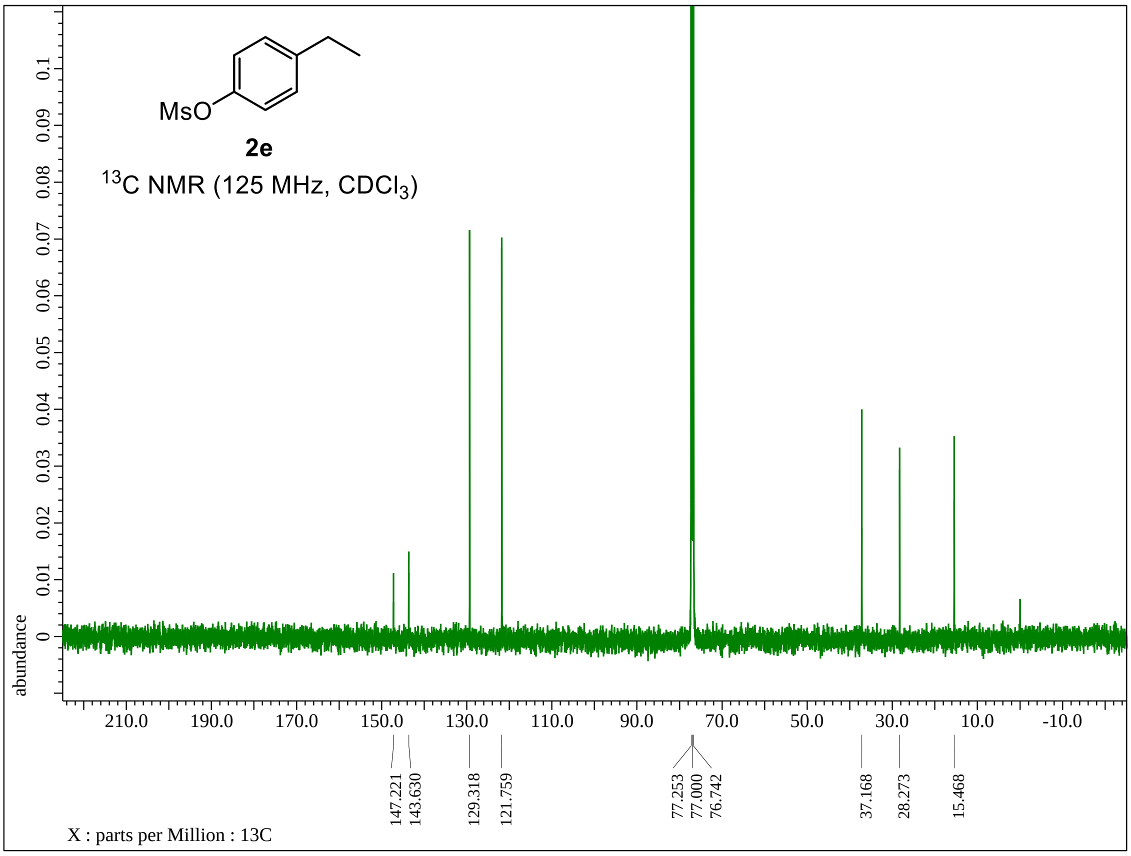
MsO
2e
13C NMR (125 MHz, CDCl3)
abundance
0
0.01
0.02
0.03
0.04
0.05
0.06
0.07
0.08
0.09
0.1
210.0
190.0
170.0
150.0
130.0
110.0
90.0
70.0
50.0
30.0
10.0
-10.0
147.221
143.630
129.318
121.759
77.253
77.000
76.742
37.168
28.273
15.468
X : parts per Million : 13C

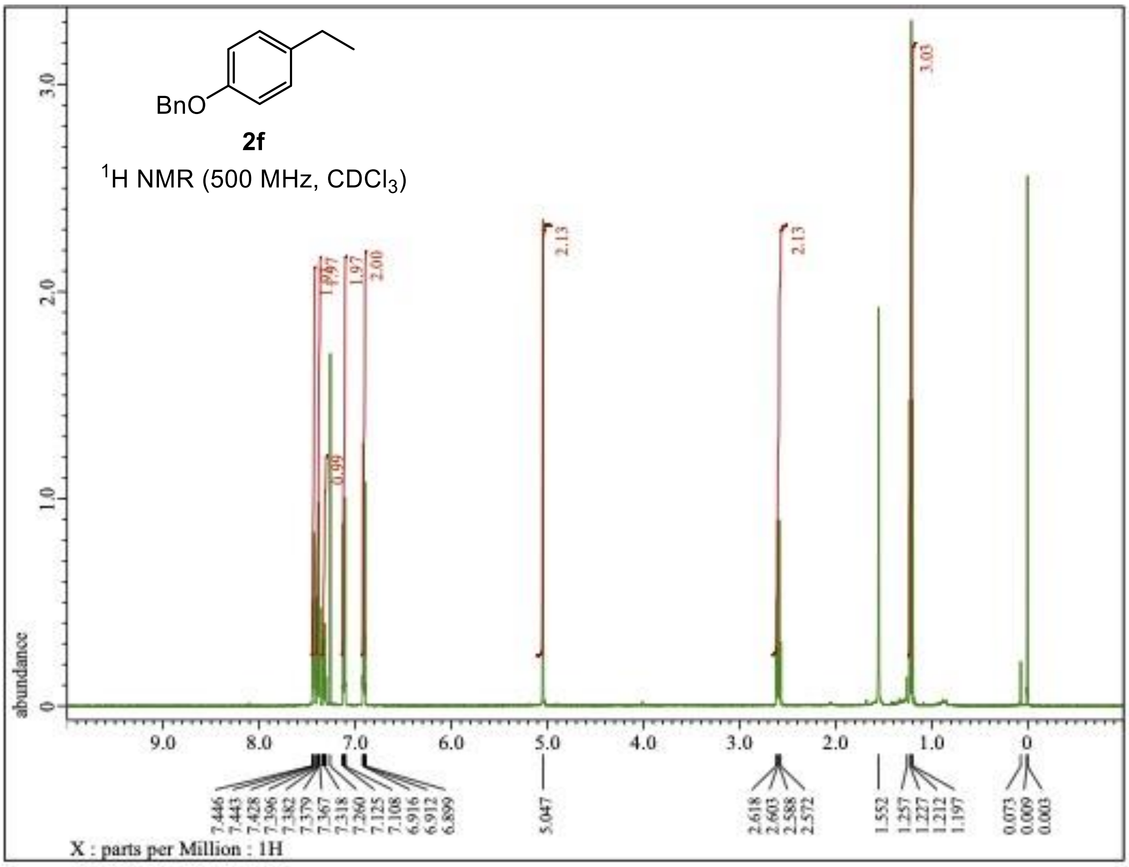

BnO
2f
1H NMR (500 MHz, CDCl3)
abundance
X : parts per Million : 1H

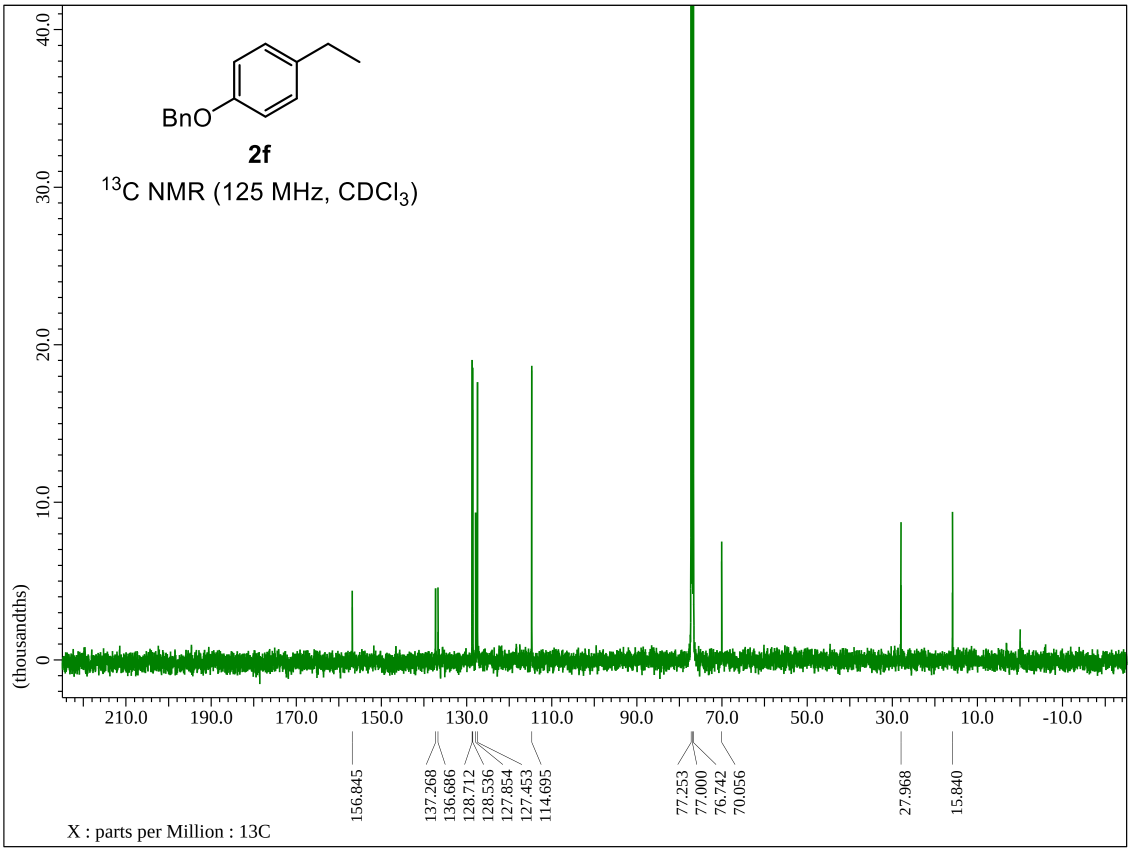

BnO
2f
13C NMR (125 MHz, CDCl3)
(thousandths)
X : parts per Million : 13C
156.845
137.268
136.686
128.712
128.536
127.854
127.453
114.695
77.253
77.000
76.742
70.056
27.968
15.840

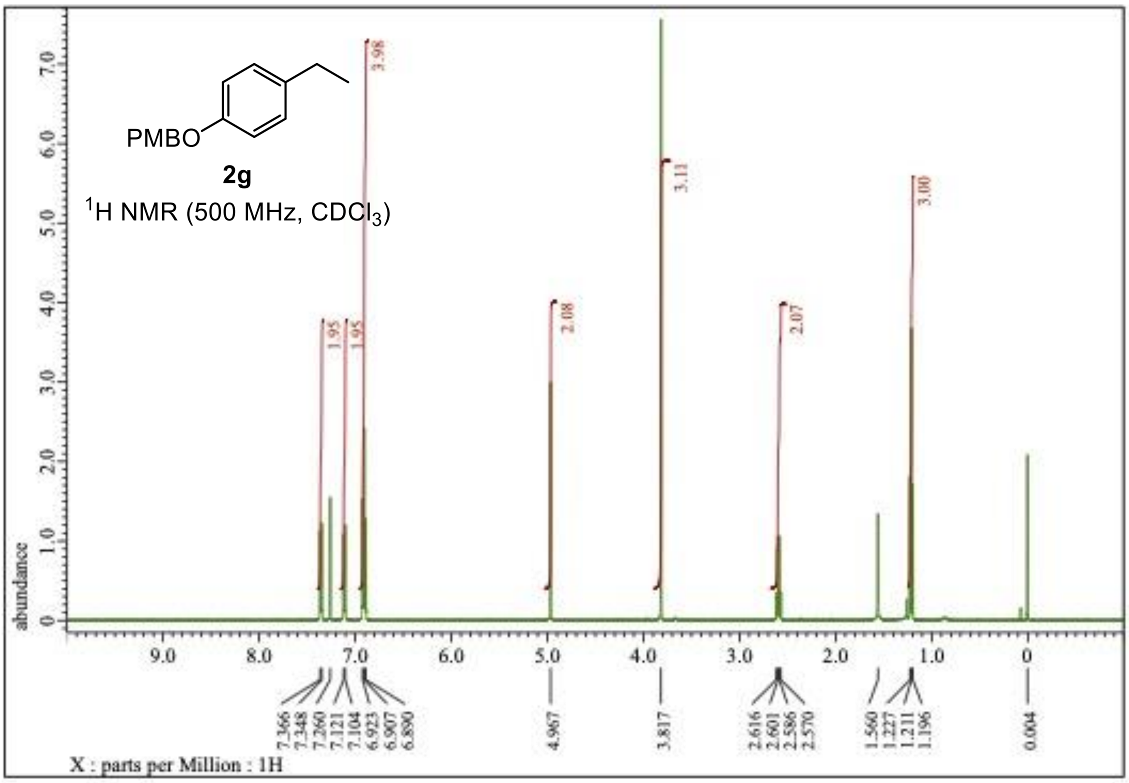
PMBO
2g
1H NMR (500 MHz, CDCl3)
abundance
X : parts per Million : 1H
7.366
7.348
7.260
7.121
7.104
6.923
6.907
6.890
4.967
3.817
2.616
2.601
2.586
2.570
1.560
1.227
1.211
1.196
0.004
1.95
1.95
3.98
2.08
3.11
2.07
3.00

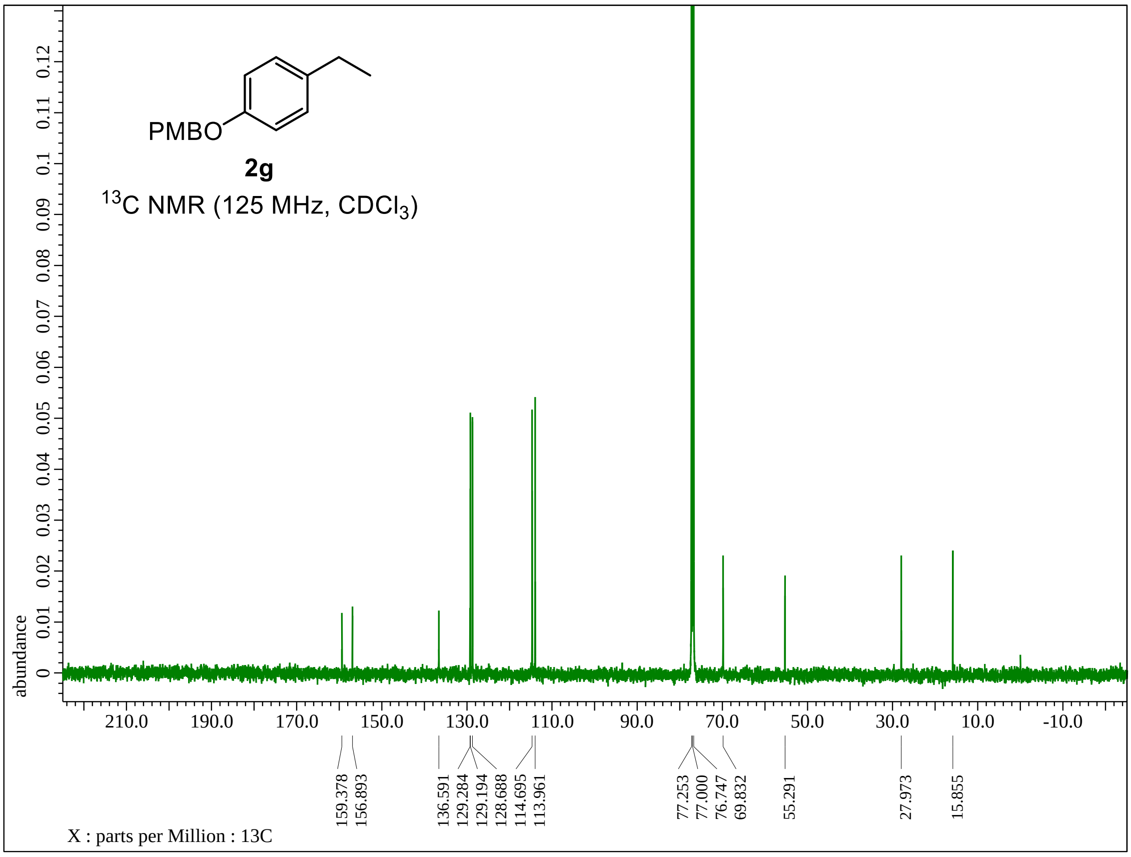
PMBO
2g
13C NMR (125 MHz, CDCl3)
abundance
X : parts per Million : 13C
159.378
156.893
136.591
129.284
129.194
128.688
114.695
113.961
77.253
77.000
76.747
69.832
55.291
27.973
15.855

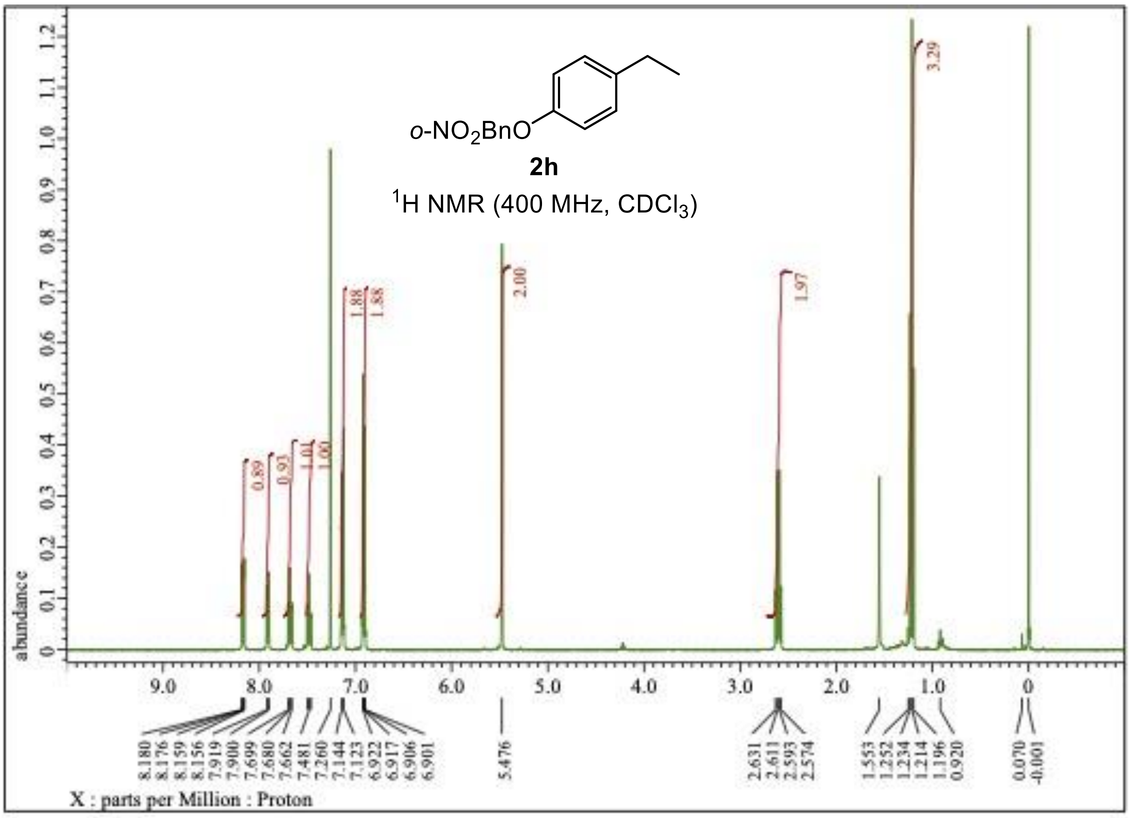
o-NO2BnO
2h
1H NMR (400 MHz, CDCl3)
X : parts per Million : Proton
abundance

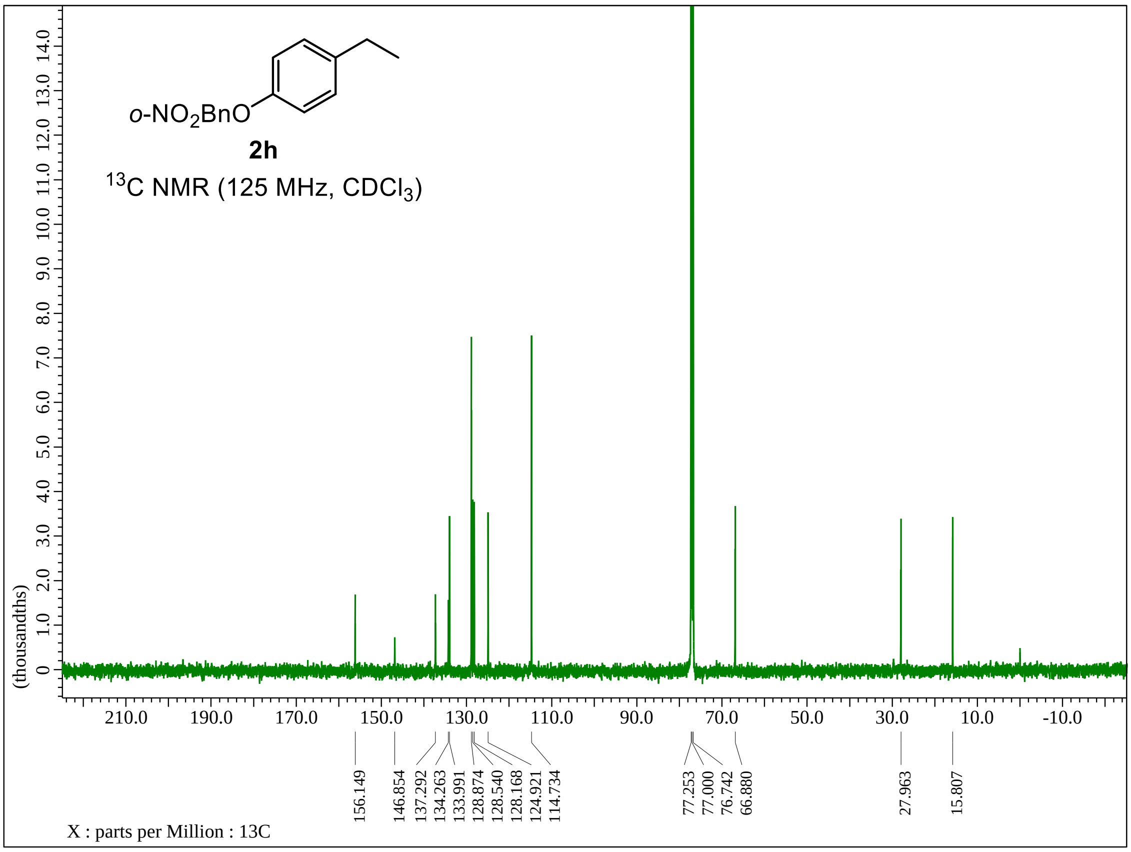
o-NO2BnO
2h
13C NMR (125 MHz, CDCl3)
X : parts per Million : 13C
(thousandths)

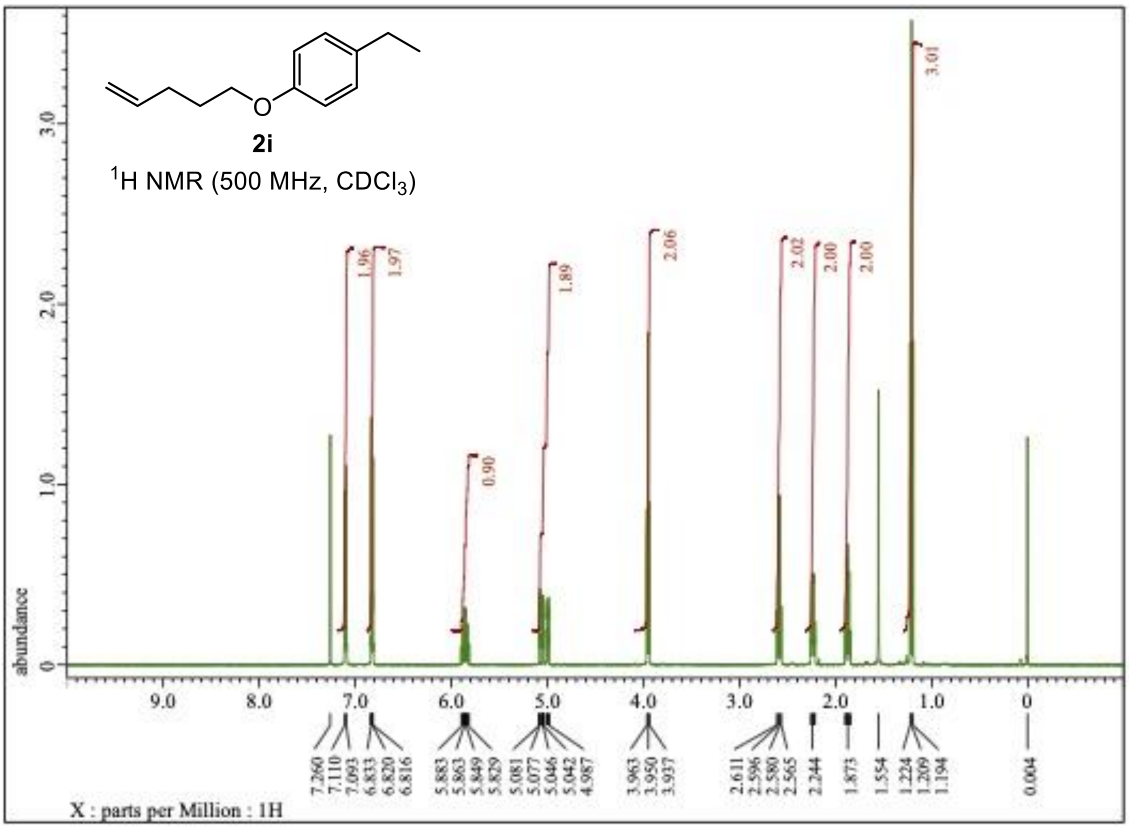

2i
1H NMR (500 MHz, CDCl3)
X : parts per Million : 1H
abundance

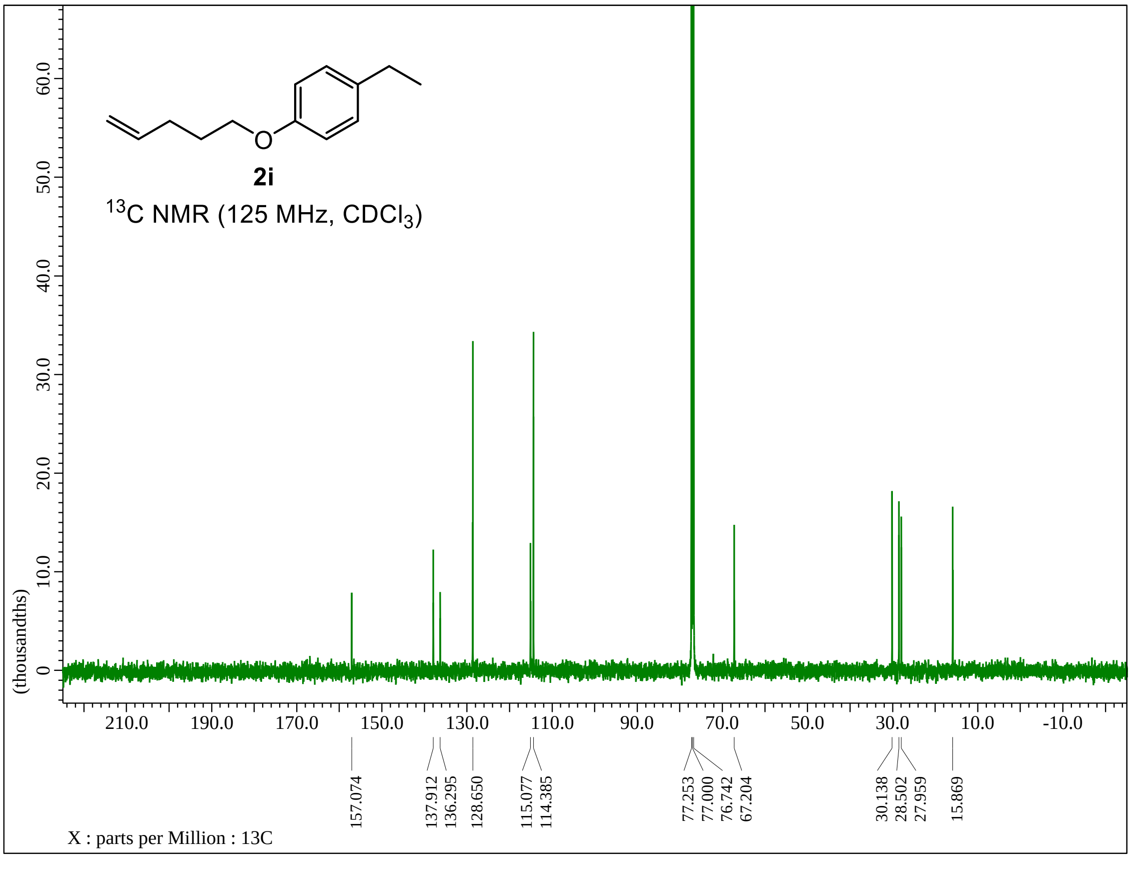

2i
13C NMR (125 MHz, CDCl3)
X : parts per Million : 13C
(thousandths)

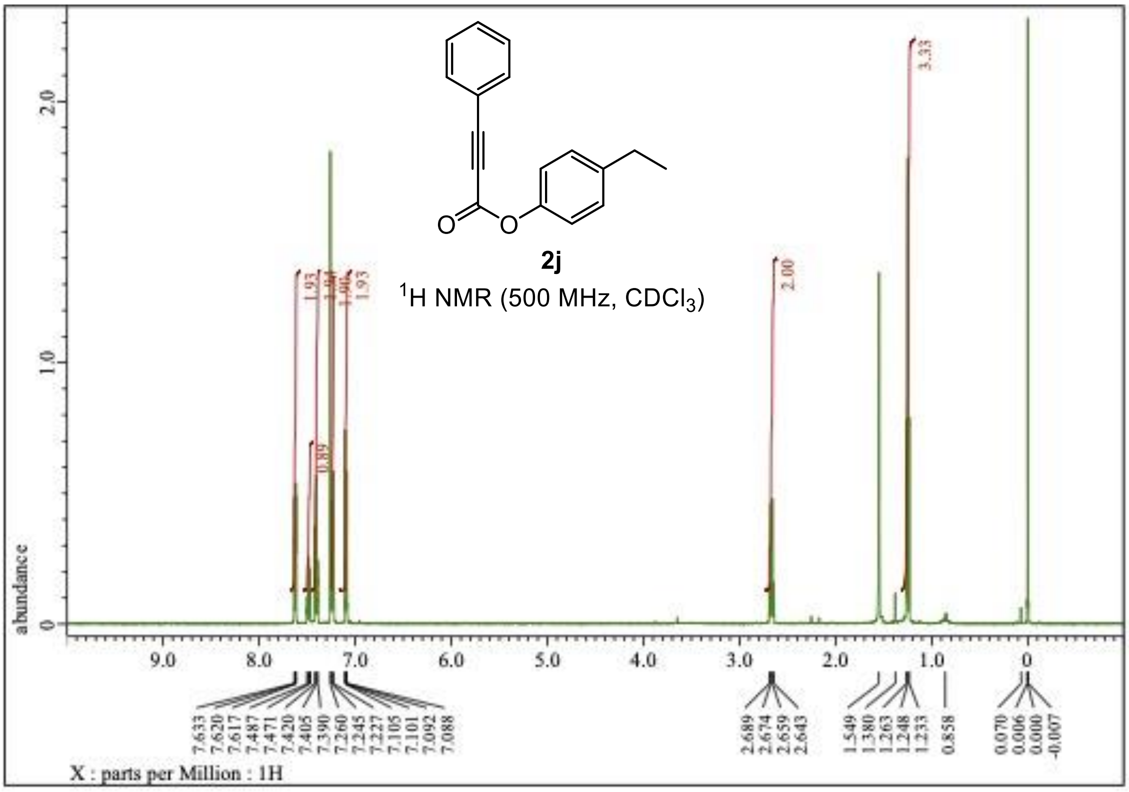

2j
^{1}H NMR (500 MHz, CDCl$_3$)
abundance
X : parts per Million : 1H
7.633 7.620 7.617 7.487 7.471 7.420 7.405 7.390 7.260 7.245 7.227 7.105 7.101 7.092 7.088
2.689 2.674 2.659 2.643
1.549 1.380 1.263 1.248 1.233
0.858
0.070 0.006 0.000 -0.007
1.93 0.89 1.94 1.96 1.93 2.00 3.33

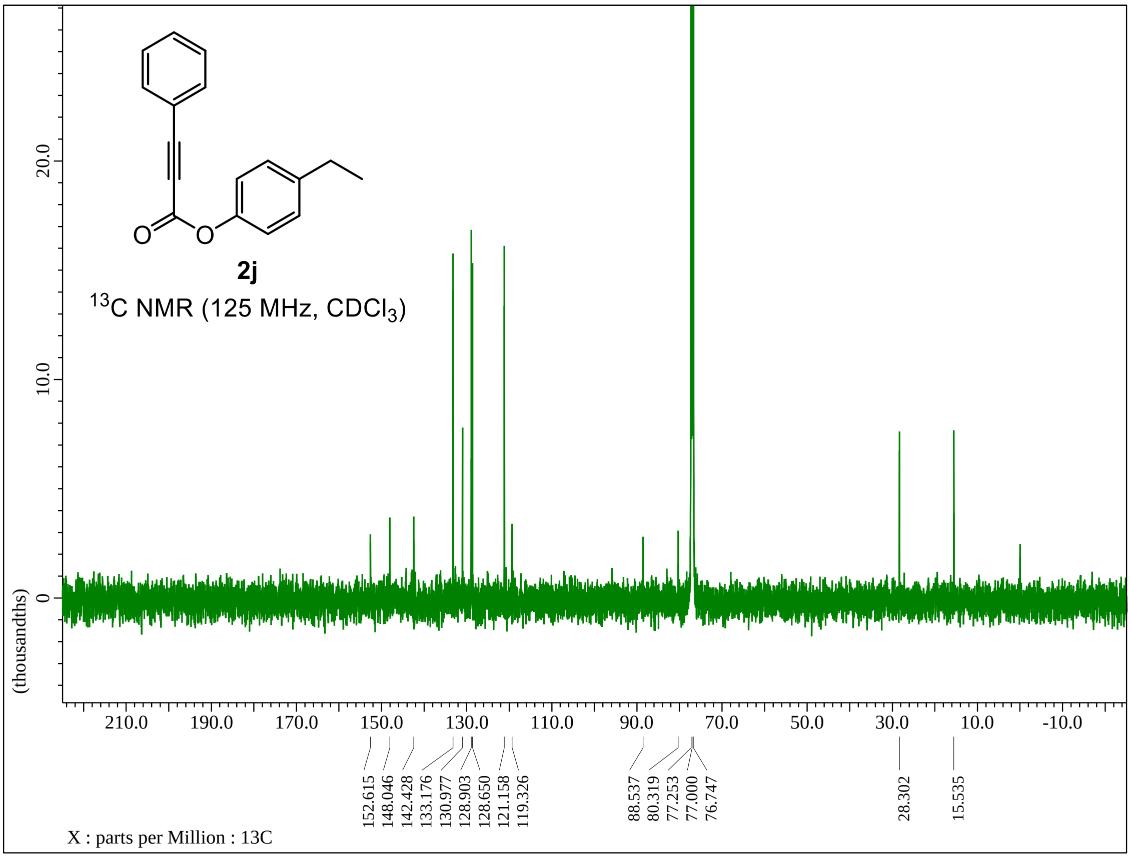

2j
^{13}C NMR (125 MHz, CDCl$_3$)
(thousandths)
X : parts per Million : 13C
152.615 148.046 142.428 133.176 130.977 128.903 128.650 121.158 119.326
88.537 80.319 77.253 77.000 76.747
28.302
15.535

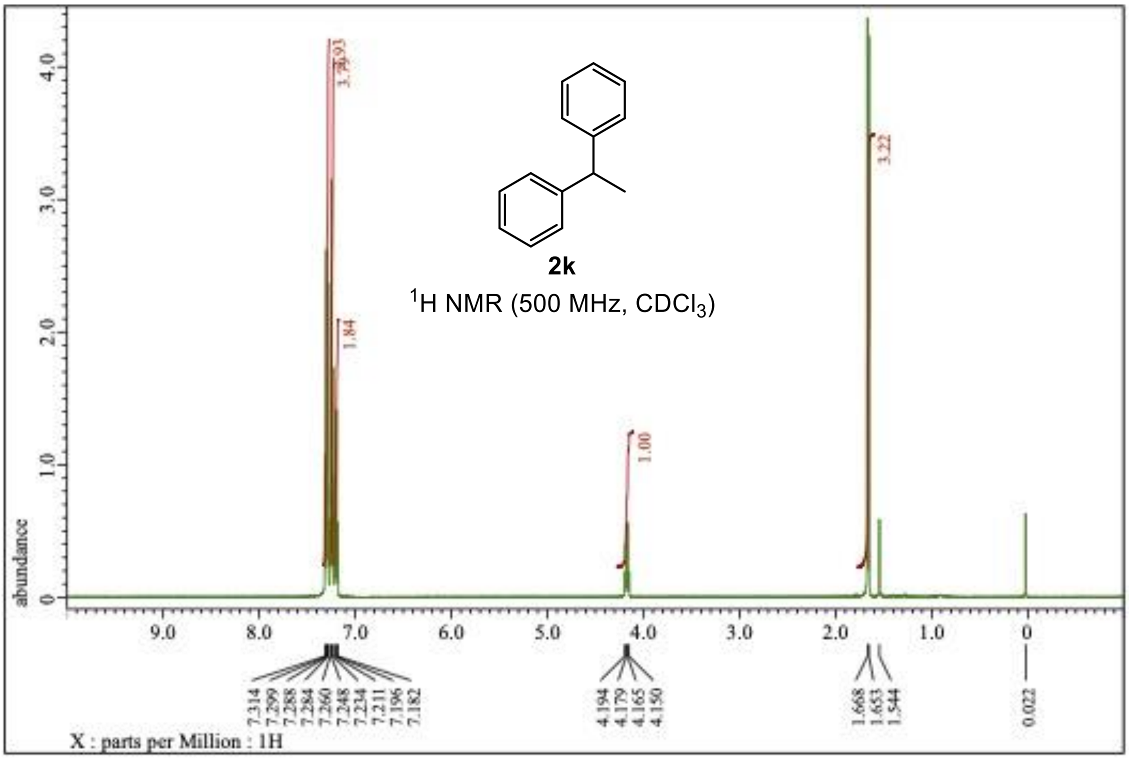
2k
1H NMR (500 MHz, CDCl3)
abundance
X : parts per Million : 1H
7.314 7.299 7.288 7.284 7.260 7.248 7.234 7.211 7.196 7.182
4.194 4.179 4.165 4.150
1.668 1.653 1.544
0.022
1.84
1.00
3.22

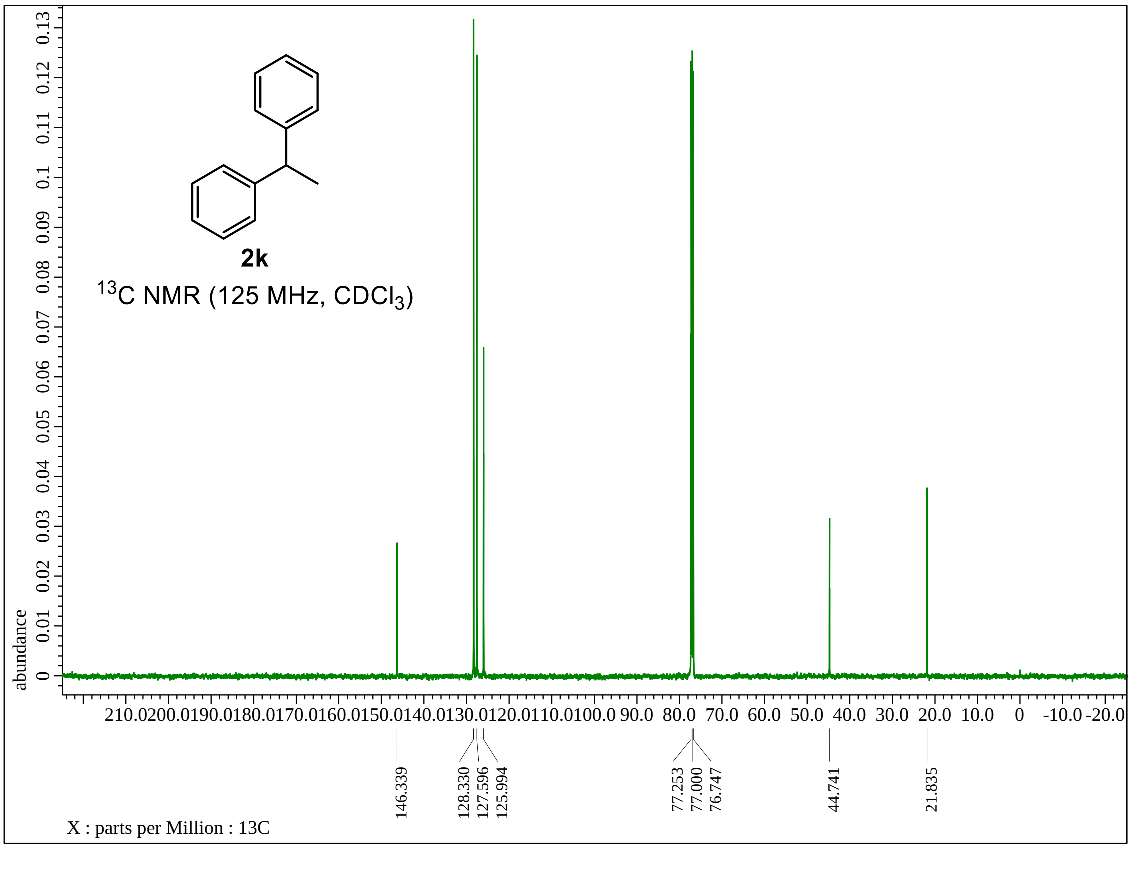
2k
13C NMR (125 MHz, CDCl3)
abundance
X : parts per Million : 13C
146.339
128.330 127.596 125.994
77.253 77.000 76.747
44.741
21.835

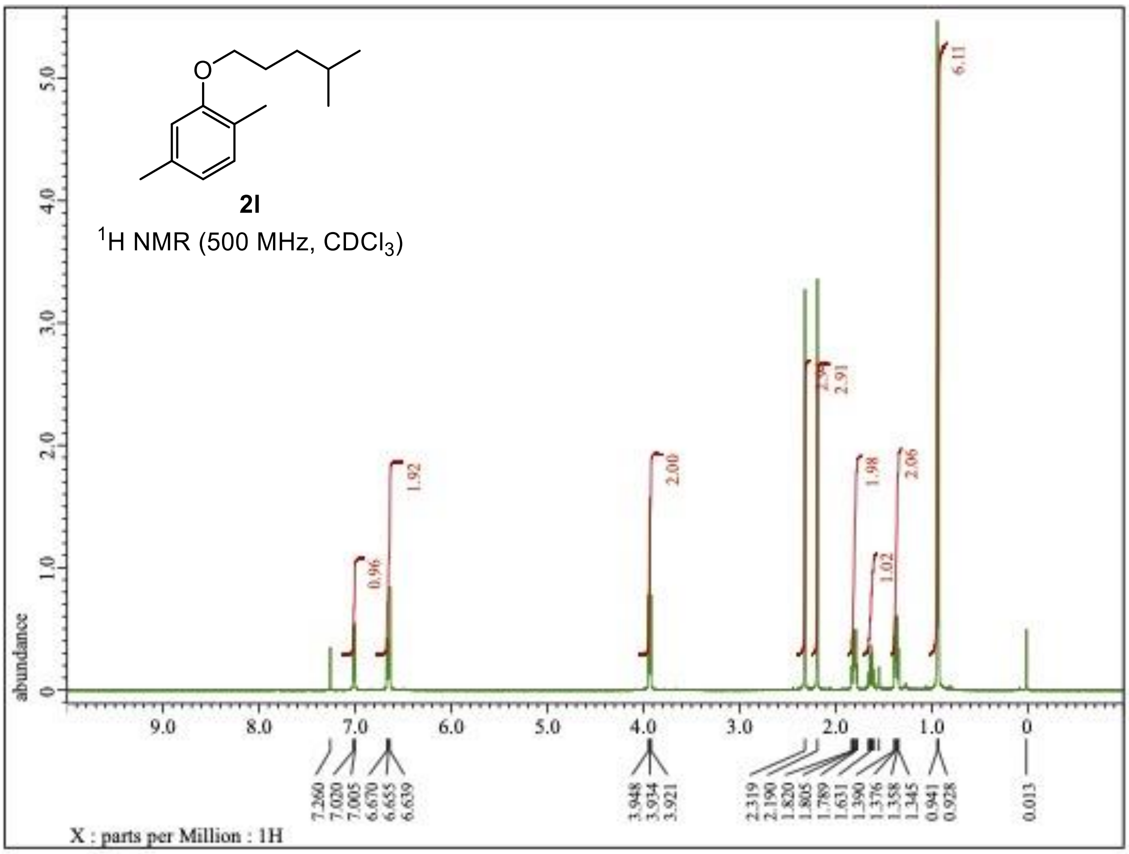
2l
^{1}H NMR (500 MHz, CDCl$_3$)
X : parts per Million : 1H
abundance

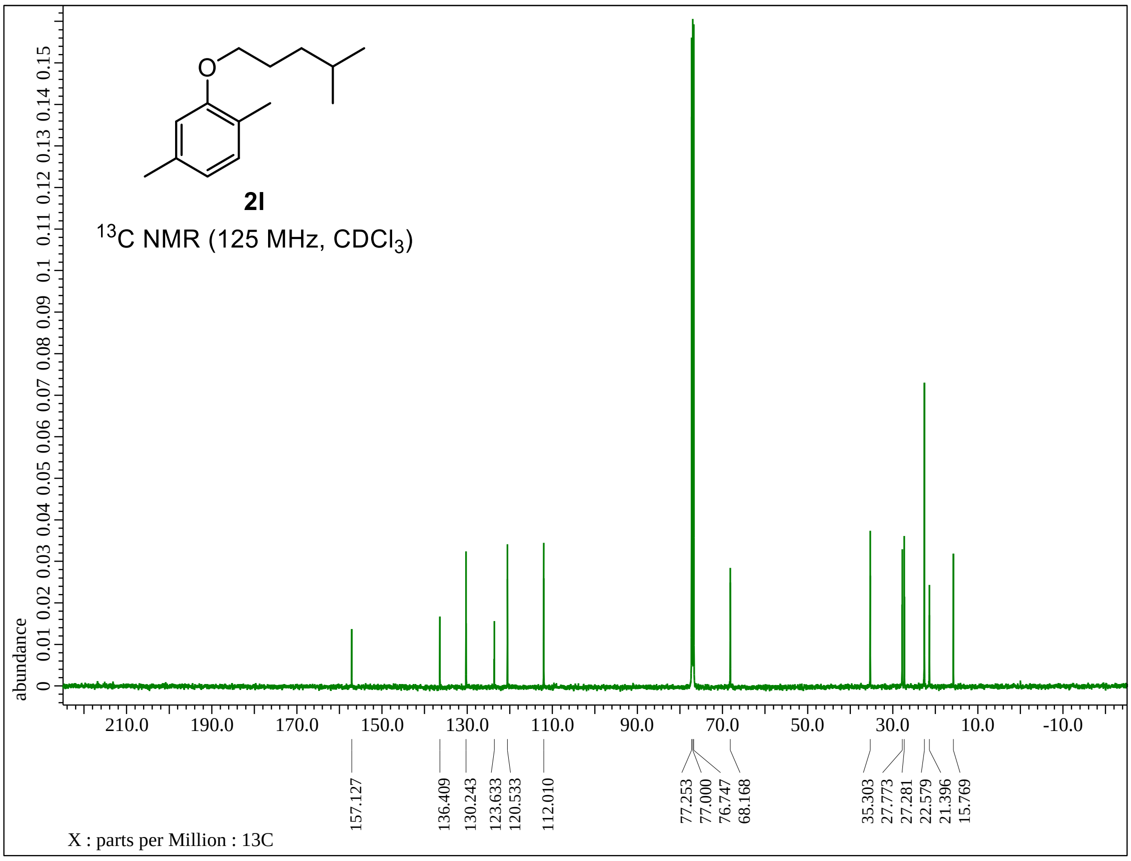
2l
^{13}C NMR (125 MHz, CDCl$_3$)
X : parts per Million : 13C
abundance
157.127
136.409
130.243
123.633
120.533
112.010
77.253
77.000
76.747
68.168
35.303
27.773
27.281
22.579
21.396
15.769

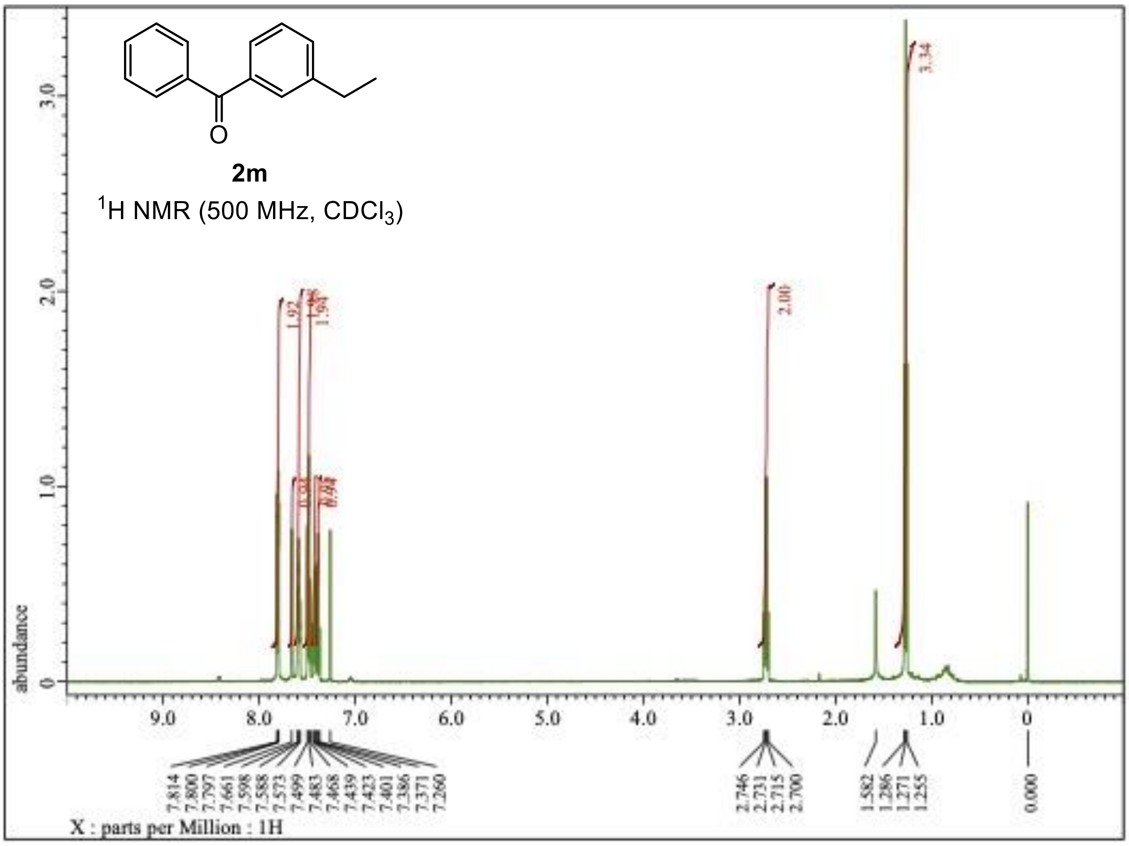
O
2m
¹H NMR (500 MHz, CDCl3)
abundance
X : parts per Million : 1H

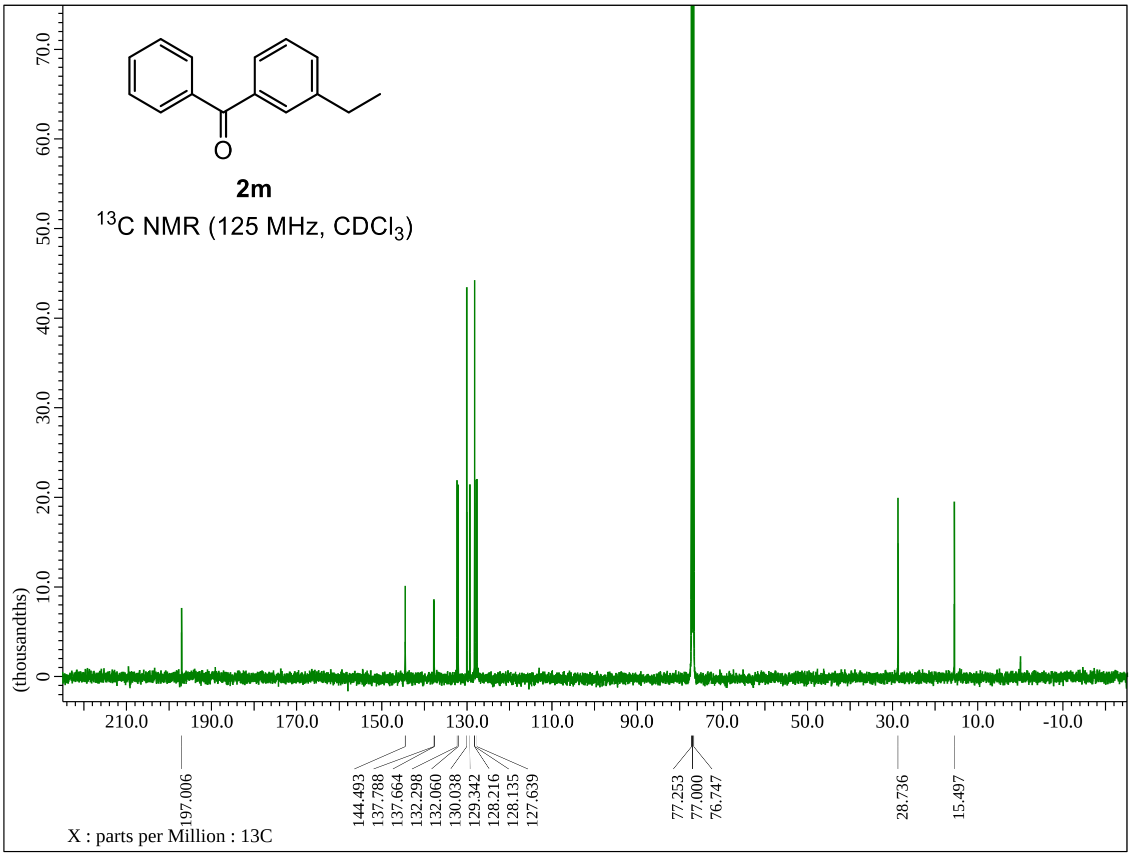
O
2m
¹³C NMR (125 MHz, CDCl3)
(thousandths)
X : parts per Million : 13C

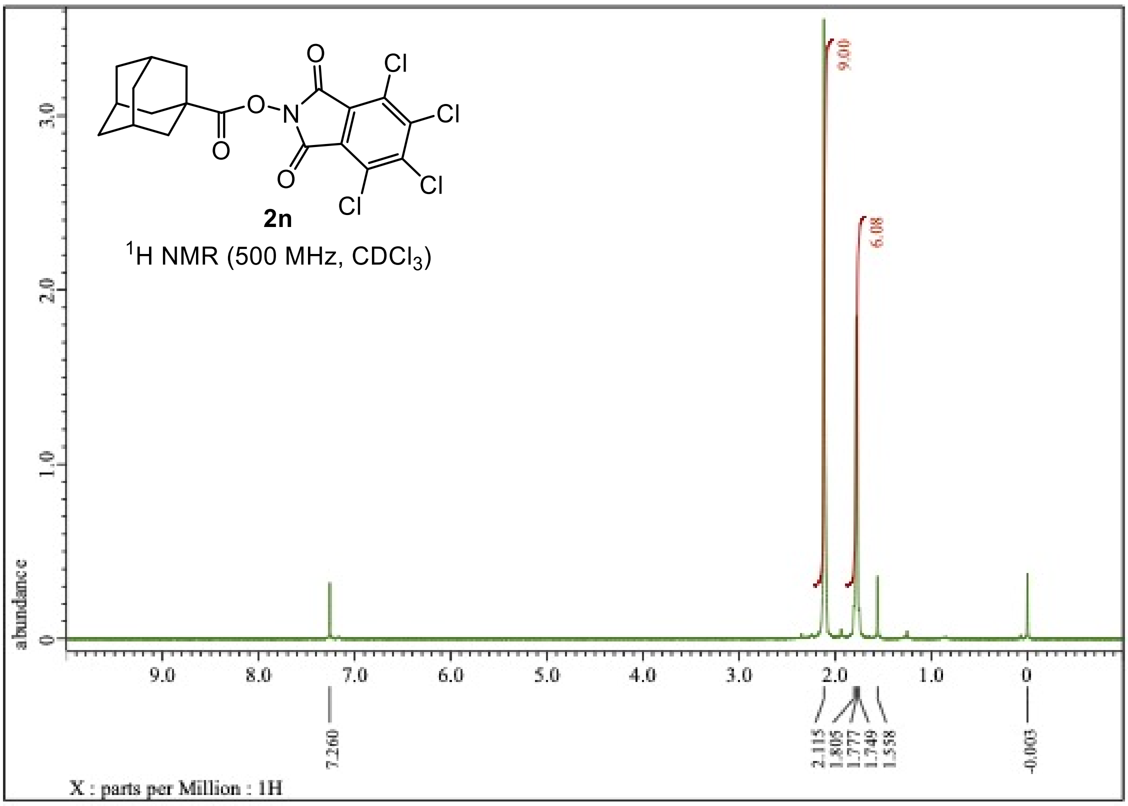
2n
^{1}H NMR (500 MHz, CDCl$_3$)
9.00
6.08
abundance
7.260
2.115
1.805
1.777
1.749
1.558
-0.003
X : parts per Million : 1H

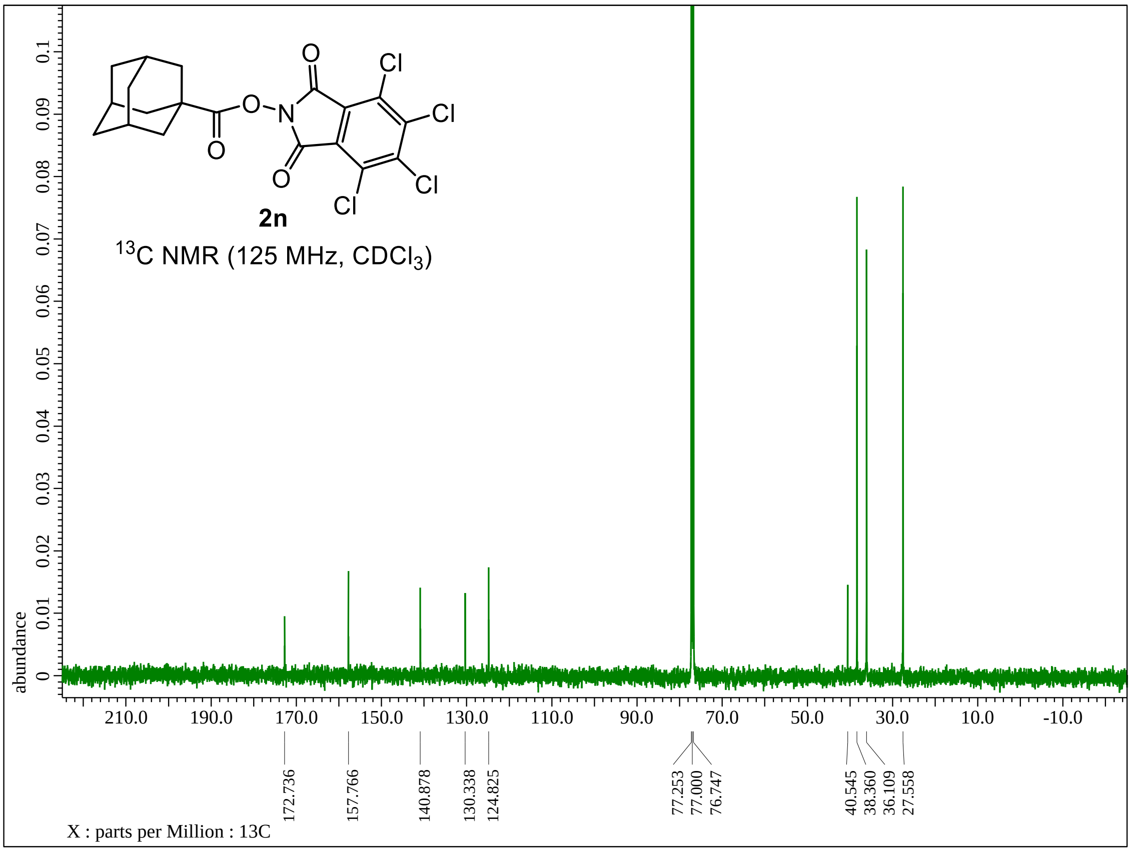
2n
^{13}C NMR (125 MHz, CDCl$_3$)
abundance
172.736
157.766
140.878
130.338
124.825
77.253
77.000
76.747
40.545
38.360
36.109
27.558
X : parts per Million : 13C

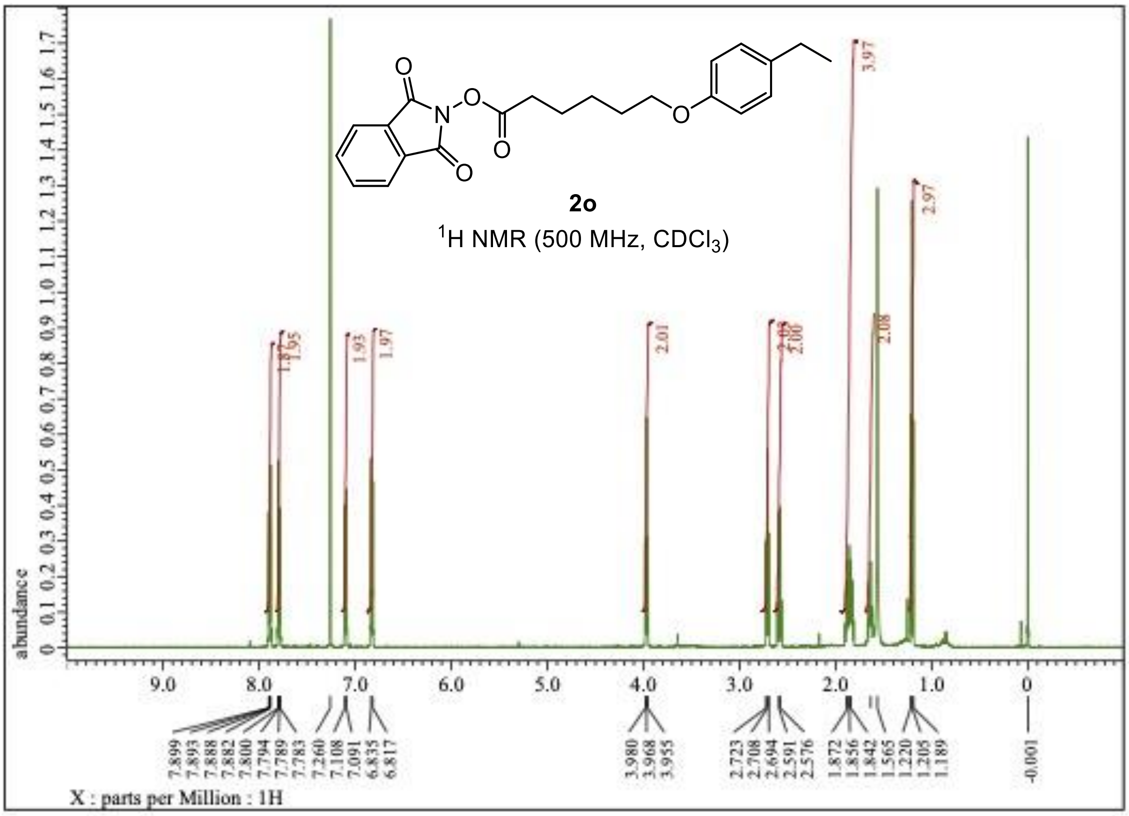
2o
1H NMR (500 MHz, CDCl3)
abundance
X : parts per Million : 1H

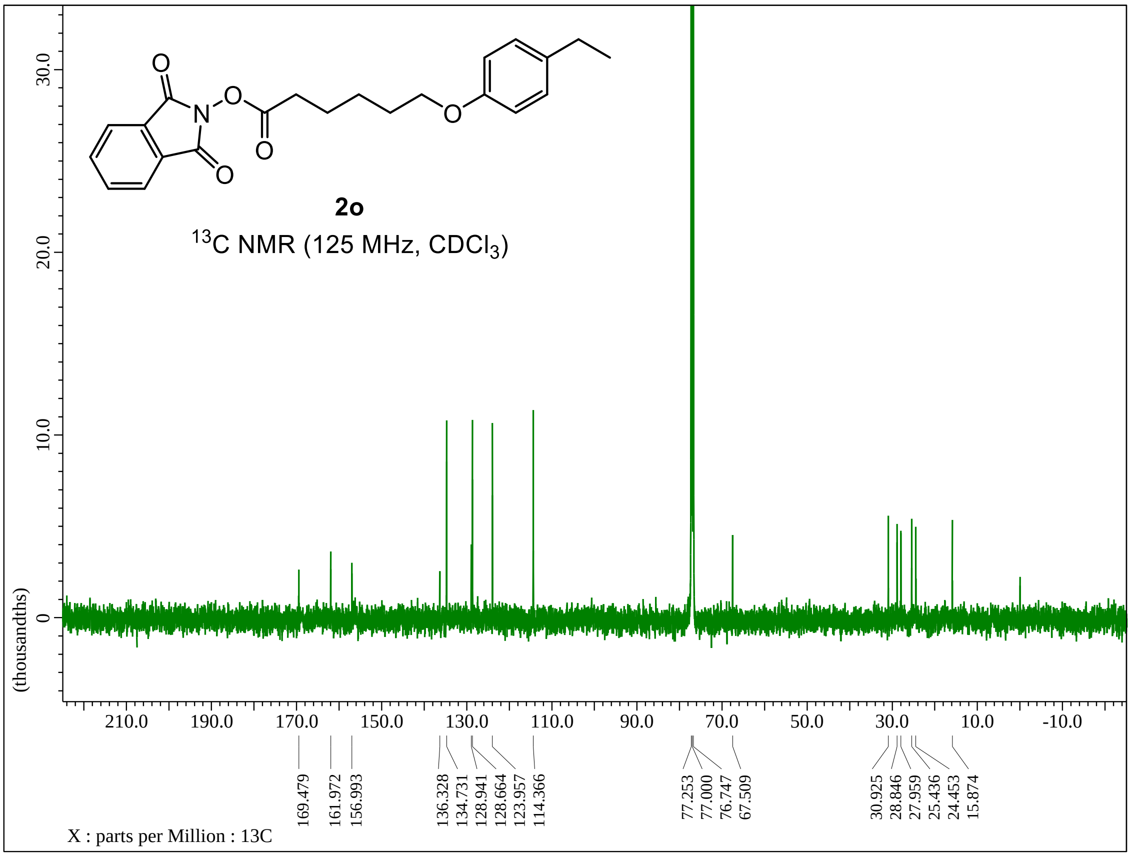
2o
13C NMR (125 MHz, CDCl3)
(thousandths)
X : parts per Million : 13C

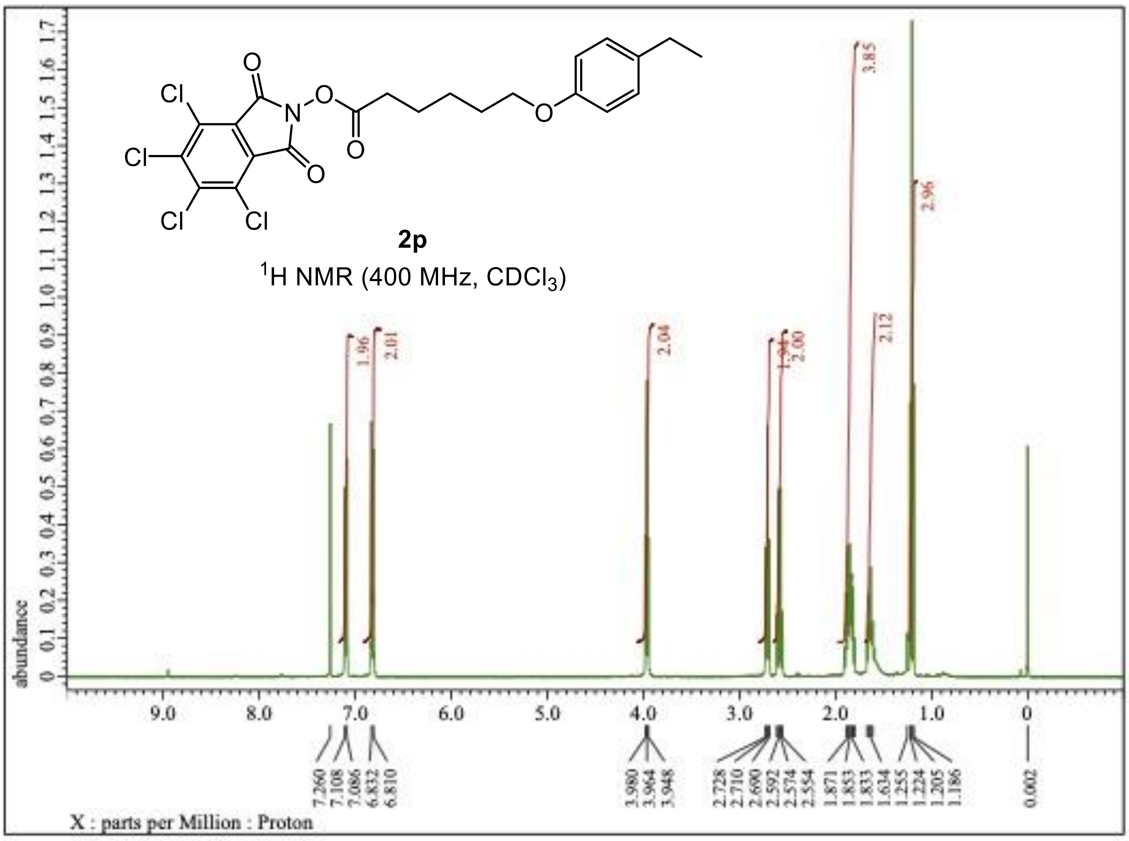
2p
^{1}H NMR (400 MHz, $CDCl_3$)
abundance
X : parts per Million : Proton

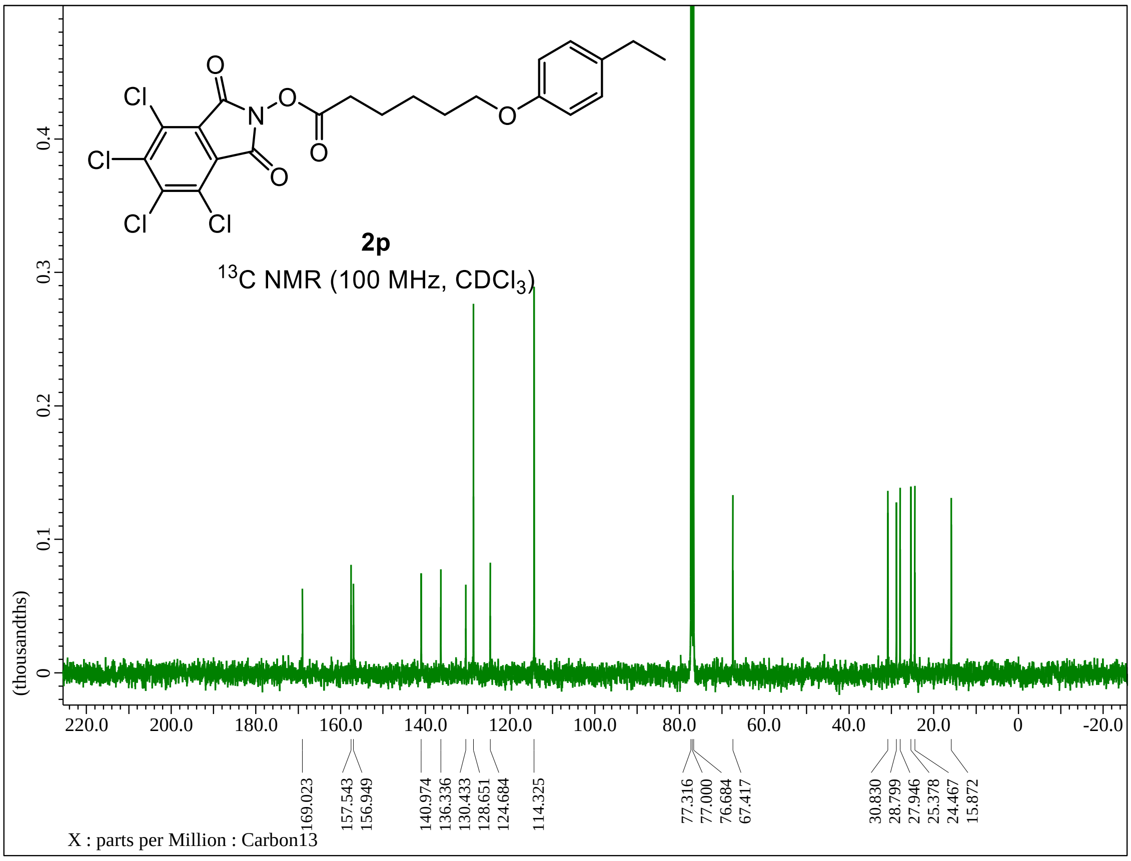
2p
^{13}C NMR (100 MHz, $CDCl_3$)
(thousandths)
X : parts per Million : Carbon13

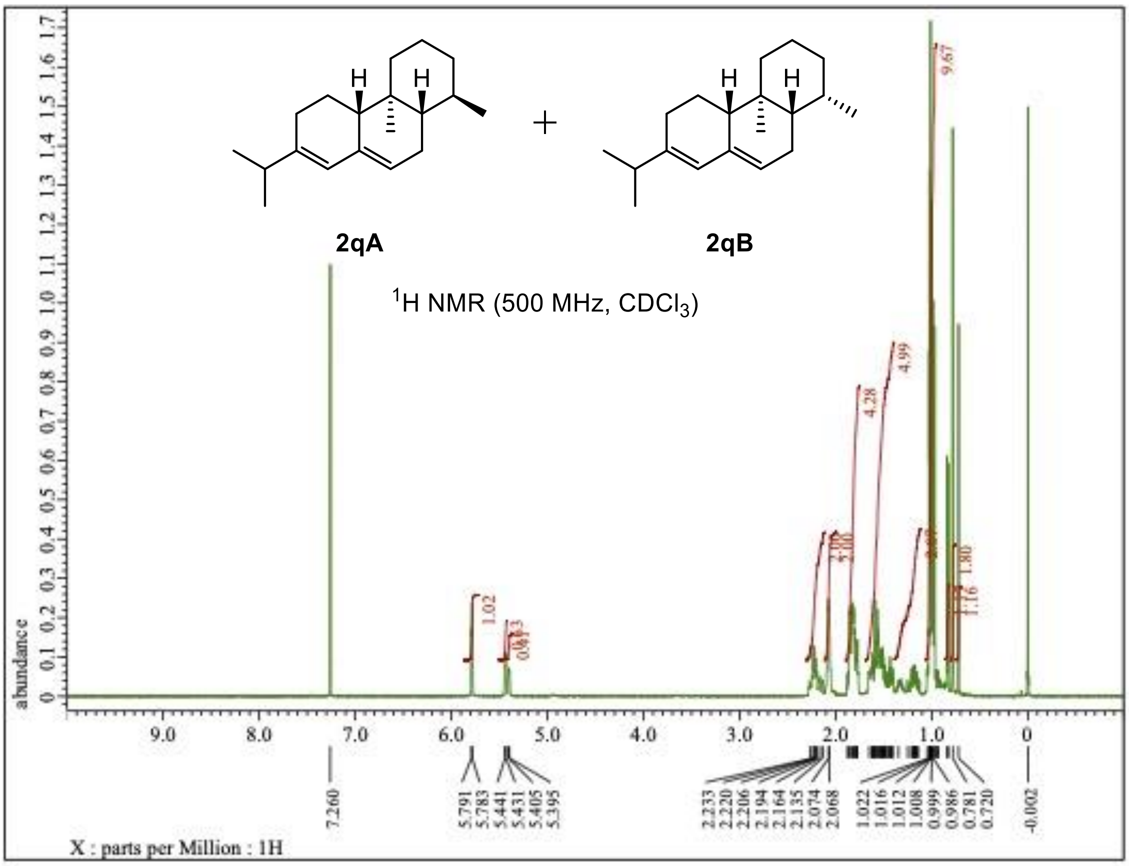
2qA
2qB
^{1}H NMR (500 MHz, CDCl$_3$)
abundance
X : parts per Million : 1H

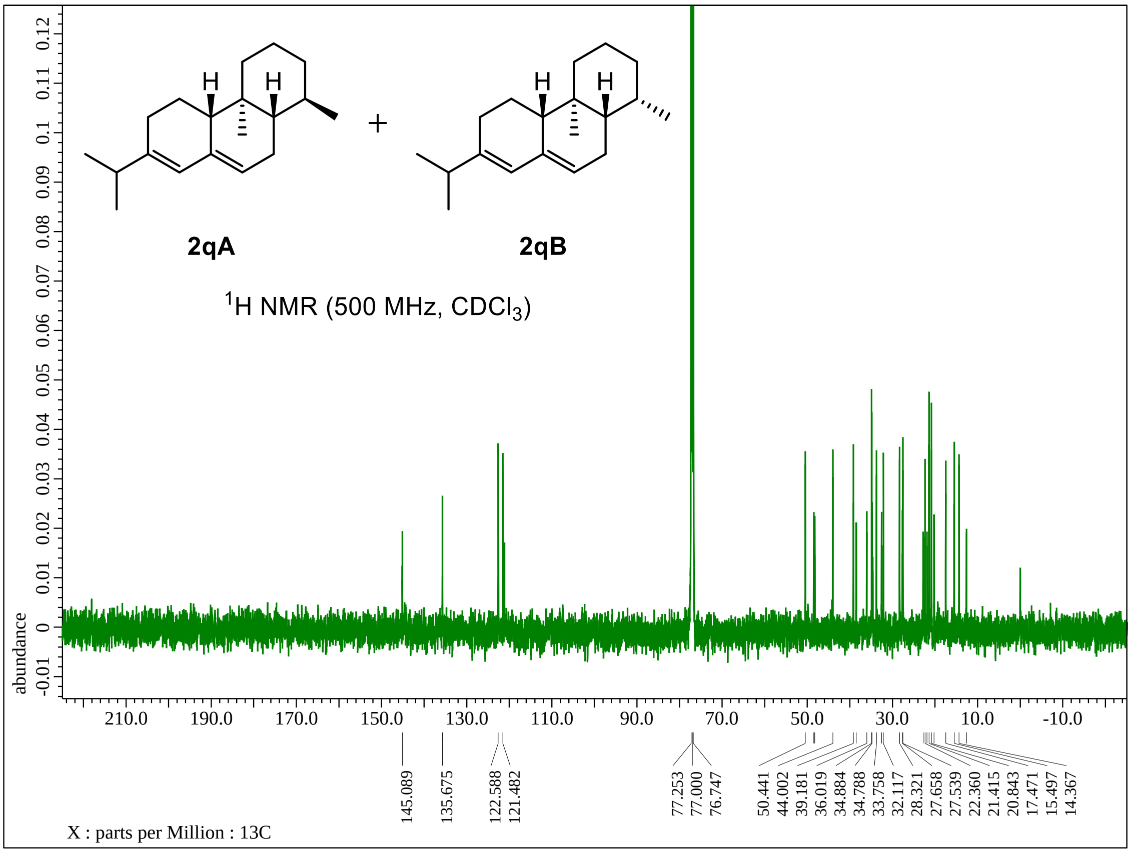
2qA
2qB
^{1}H NMR (500 MHz, CDCl$_3$)
abundance
145.089
135.675
122.588
121.482
77.253
77.000
76.747
50.441
44.002
39.181
36.019
34.884
34.788
33.758
32.117
28.321
27.658
27.539
22.360
21.415
20.843
17.471
15.497
14.367
X : parts per Million : 13C

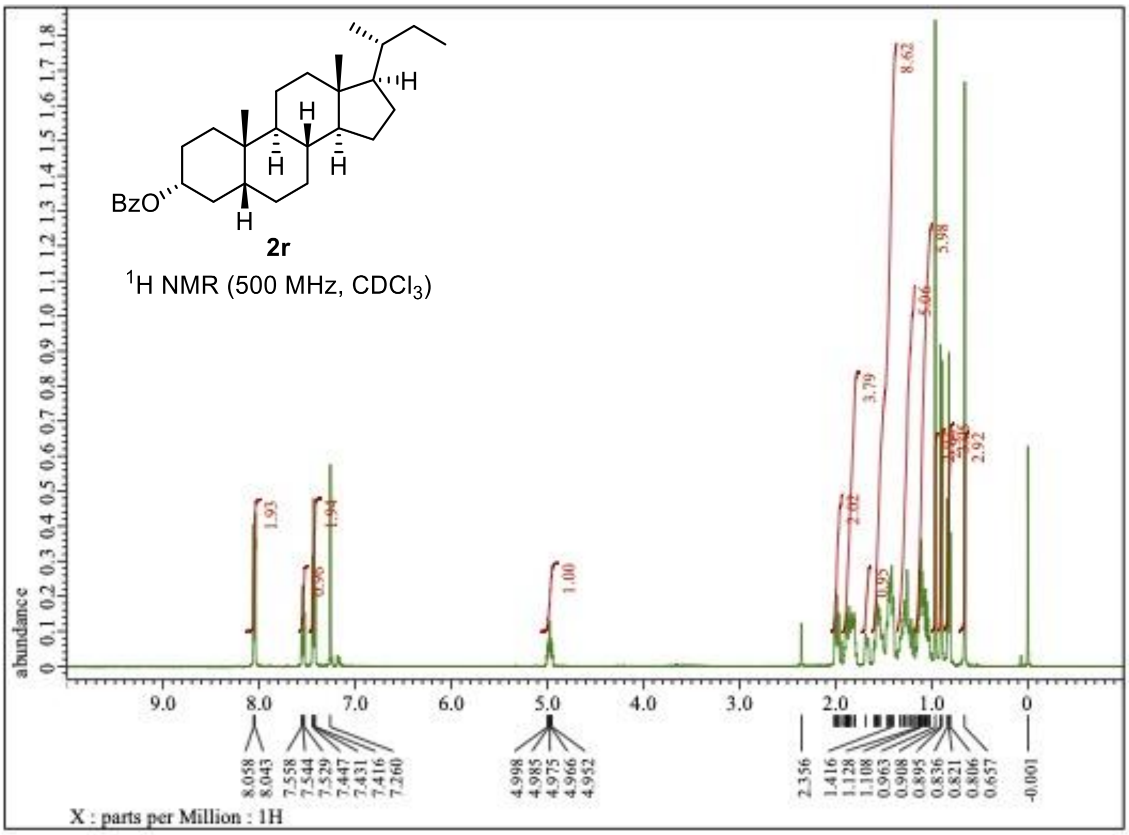

BzO
H
2r
^{1}H NMR (500 MHz, CDCl$_3$)
abundance
X : parts per Million : 1H
8.058
8.043
7.558
7.544
7.529
7.447
7.431
7.416
7.260
4.998
4.985
4.975
4.966
4.952
2.356
1.416
1.128
1.108
0.963
0.908
0.895
0.836
0.821
0.806
0.657
-0.001
1.93
1.94
1.00
2.02
3.79
8.62
5.06
5.98
2.92

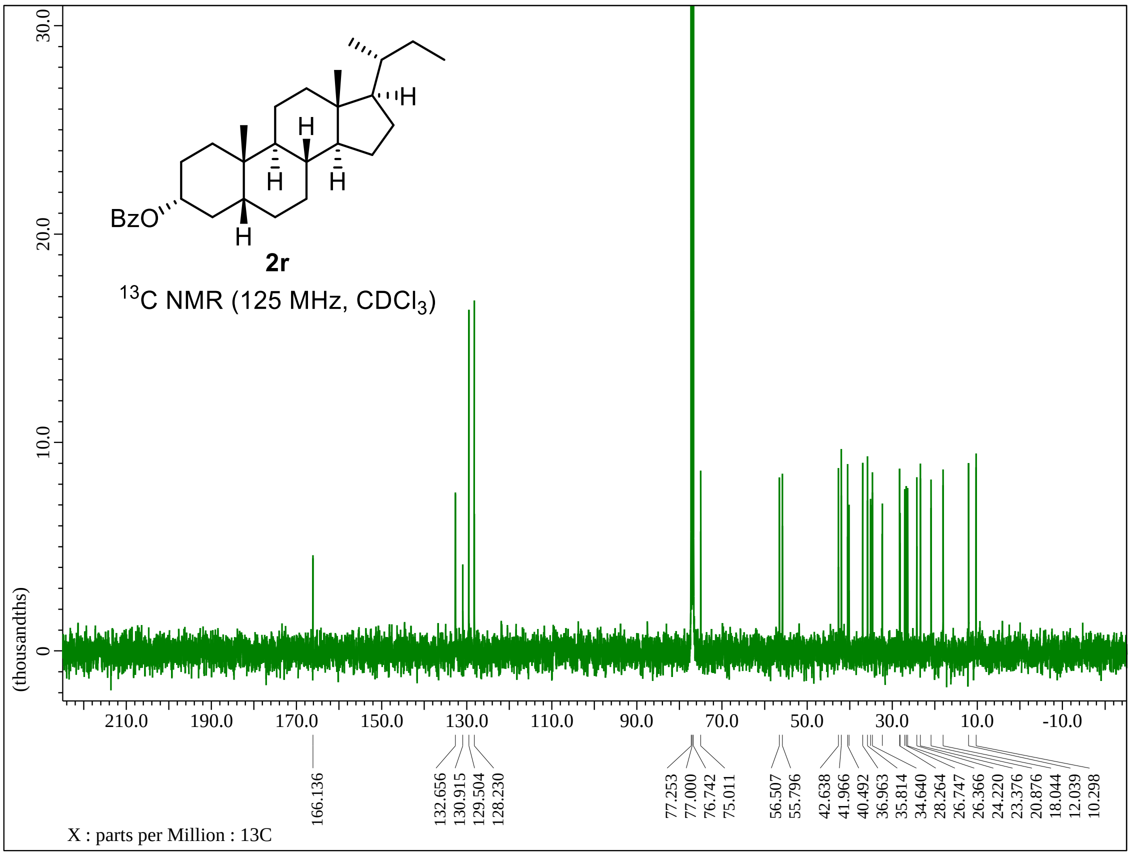

BzO
H
2r
^{13}C NMR (125 MHz, CDCl$_3$)
(thousandths)
X : parts per Million : 13C
166.136
132.656
130.915
129.504
128.230
77.253
77.000
76.742
75.011
56.507
55.796
42.638
41.966
40.492
36.963
35.814
34.640
28.264
26.747
26.366
24.220
23.376
20.876
18.044
12.039
10.298

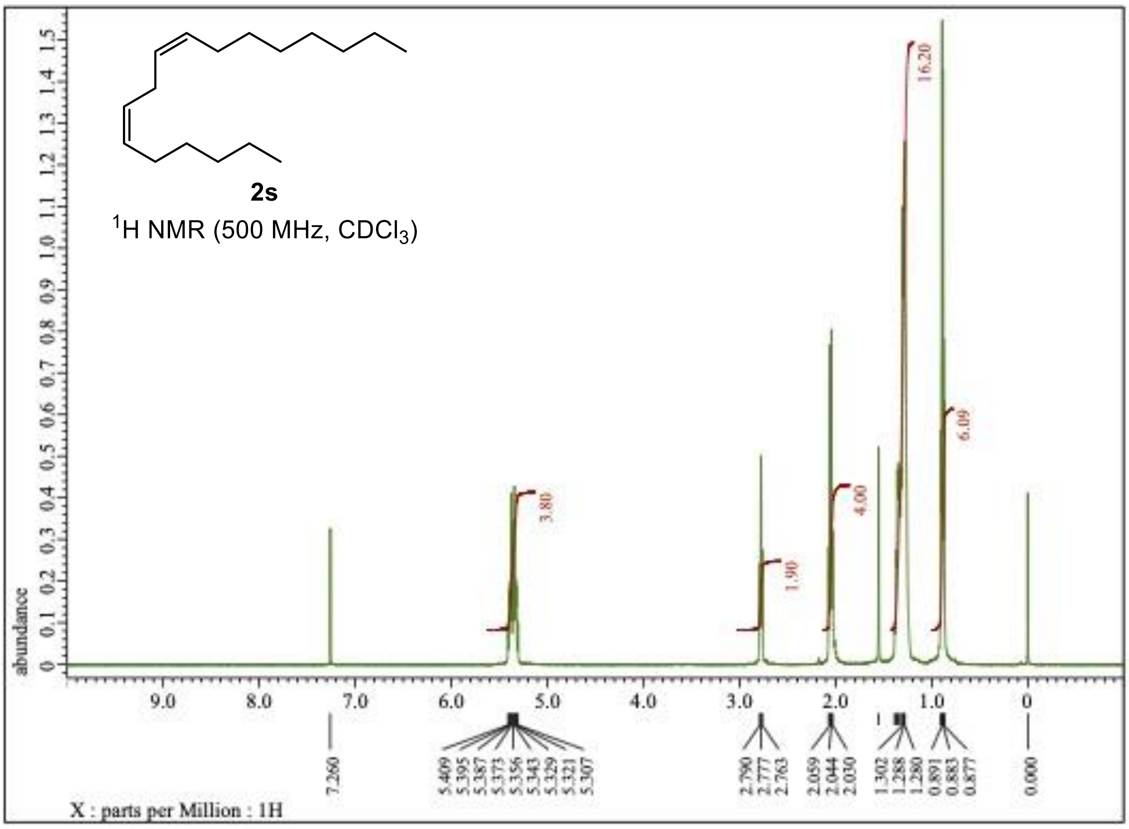
2s
^{1}H NMR (500 MHz, CDCl$_3$)
X : parts per Million : 1H
abundance
3.80
1.90
4.00
16.20
6.09
7.260
5.409
5.395
5.387
5.373
5.356
5.343
5.329
5.321
5.307
2.790
2.777
2.763
2.059
2.044
2.030
1.302
1.288
1.280
0.891
0.883
0.877
0.000

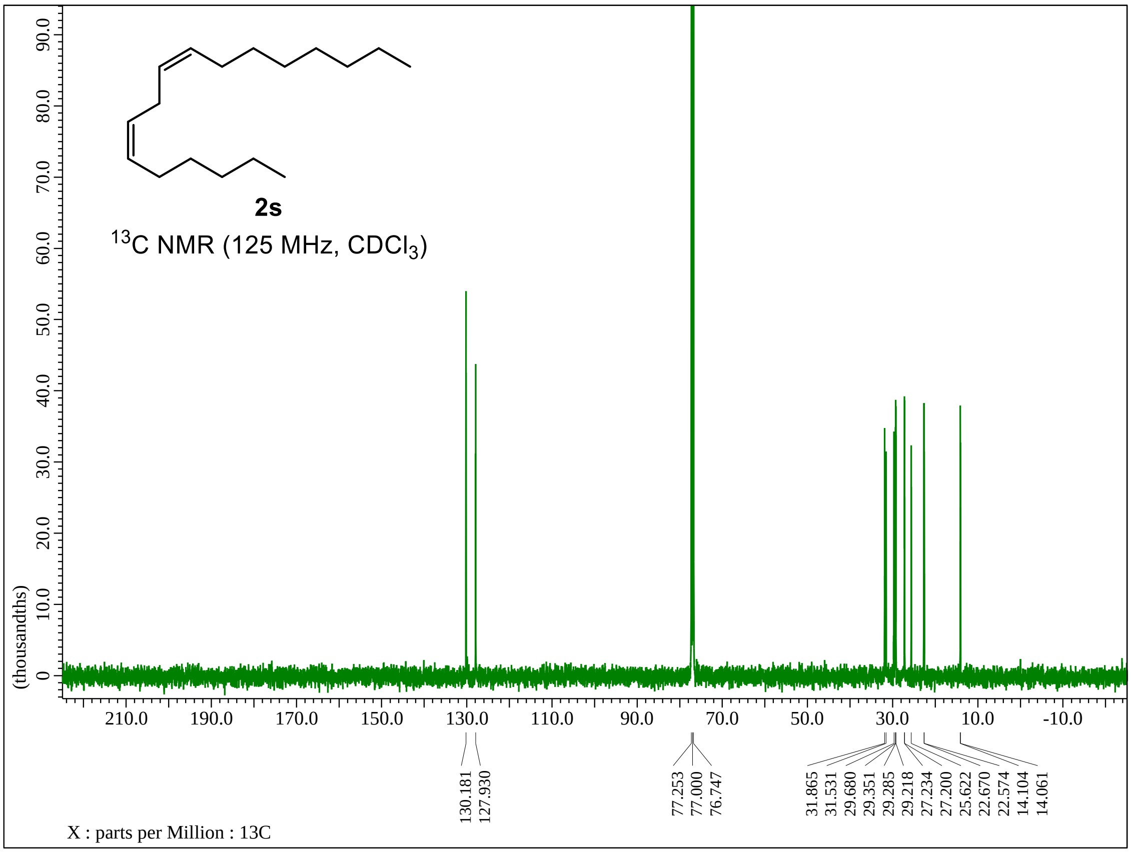
2s
^{13}C NMR (125 MHz, CDCl$_3$)
X : parts per Million : 13C
(thousandths)
130.181
127.930
77.253
77.000
76.747
31.865
31.531
29.680
29.351
29.285
29.218
27.234
27.200
25.622
22.670
22.574
14.104
14.061

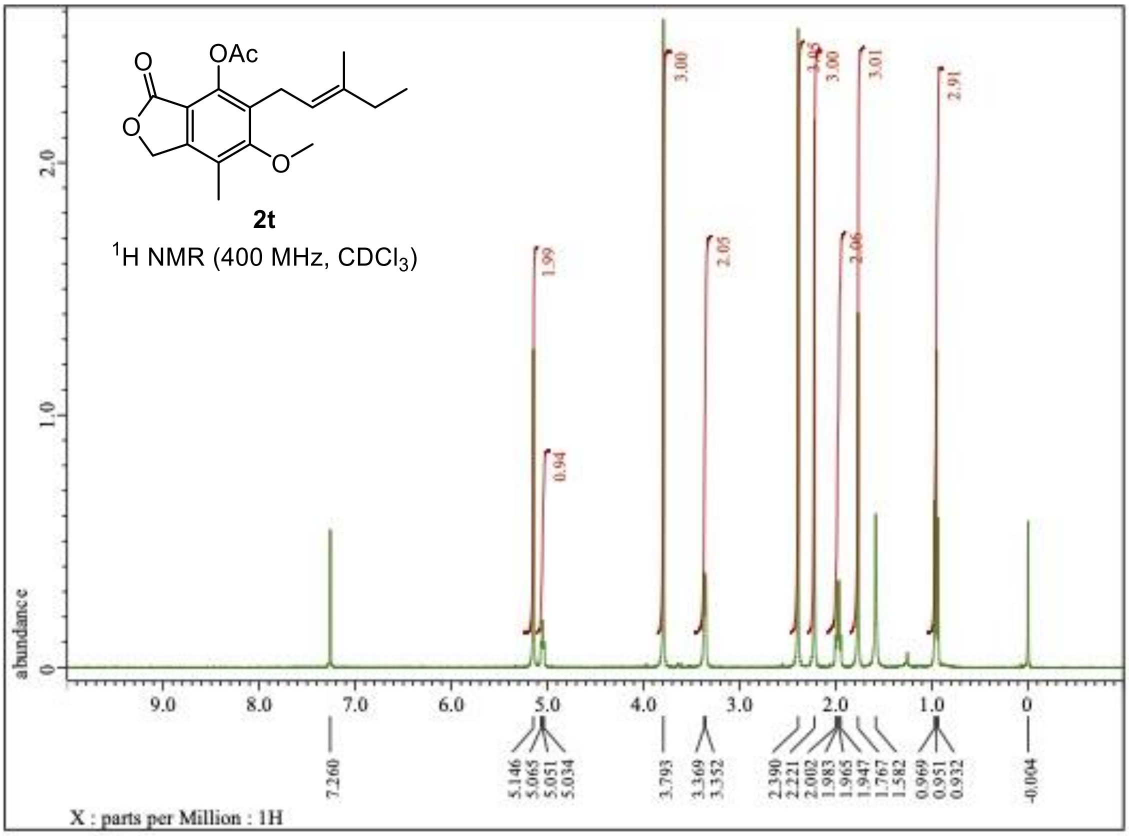
OAc
O
O
O
2t
^{1}H NMR (400 MHz, CDCl$_3$)
1.99
0.94
3.00
2.05
3.05
3.00
2.06
3.01
2.91
abundance
2.0
1.0
0
9.0
8.0
7.0
6.0
5.0
4.0
3.0
2.0
1.0
0
7.260
5.146
5.065
5.051
5.034
3.793
3.369
3.352
2.390
2.221
2.002
1.983
1.965
1.947
1.767
1.582
0.969
0.951
0.932
-0.004
X : parts per Million : 1H

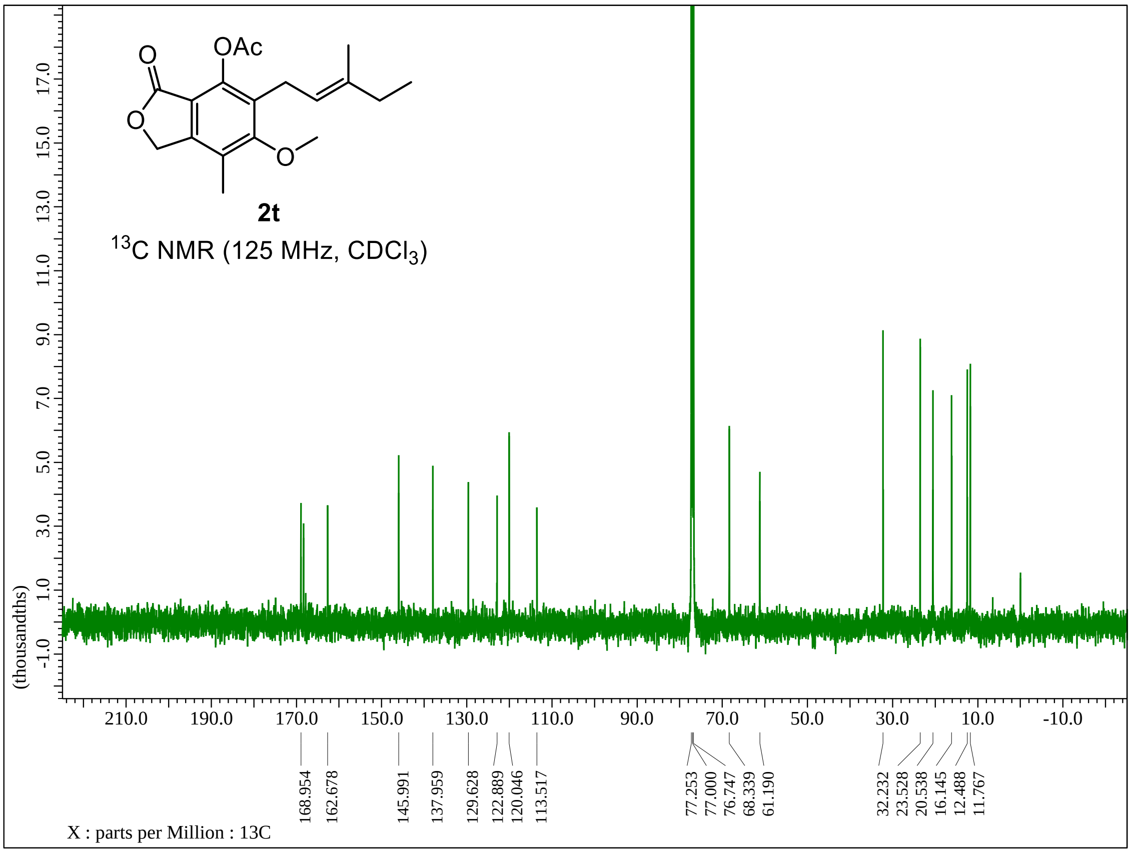
OAc
O
O
O
2t
^{13}C NMR (125 MHz, CDCl$_3$)
(thousandths)
17.0
15.0
13.0
11.0
9.0
7.0
5.0
3.0
1.0
-1.0
210.0
190.0
170.0
150.0
130.0
110.0
90.0
70.0
50.0
30.0
10.0
-10.0
168.954
162.678
145.991
137.959
129.628
122.889
120.046
113.517
77.253
77.000
76.747
68.339
61.190
32.232
23.528
20.538
16.145
12.488
11.767
X : parts per Million : 13C

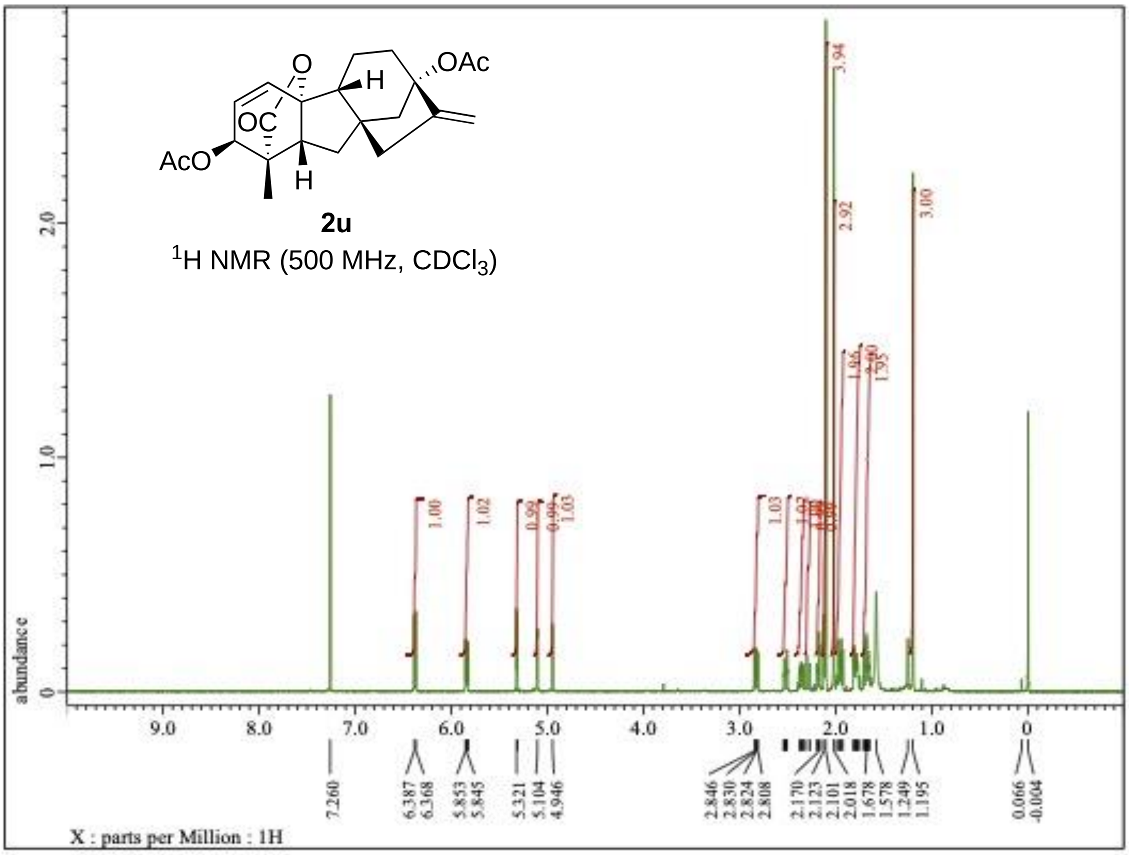
OAc
O
H
OC
AcO
H
2u
1H NMR (500 MHz, CDCl3)
abundance
X : parts per Million : 1H
7.260
6.387
6.368
5.853
5.845
5.321
5.104
4.946
2.846
2.830
2.824
2.808
2.170
2.123
2.101
2.018
1.678
1.578
1.249
1.195
0.066
-0.004

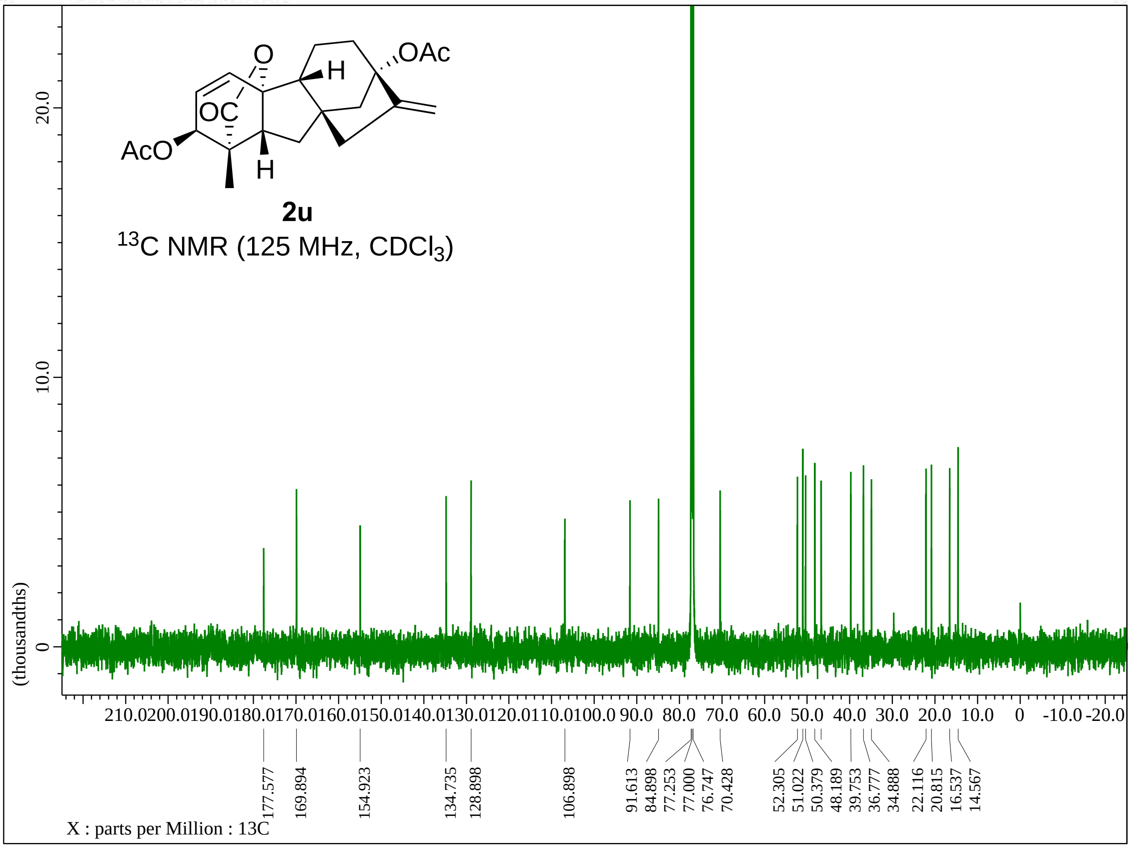
OAc
O
H
OC
AcO
H
2u
13C NMR (125 MHz, CDCl3)
(thousandths)
X : parts per Million : 13C
177.577
169.894
154.923
134.735
128.898
106.898
91.613
84.898
77.253
77.000
76.747
70.428
52.305
51.022
50.379
48.189
39.753
36.777
34.888
22.116
20.815
16.537
14.567

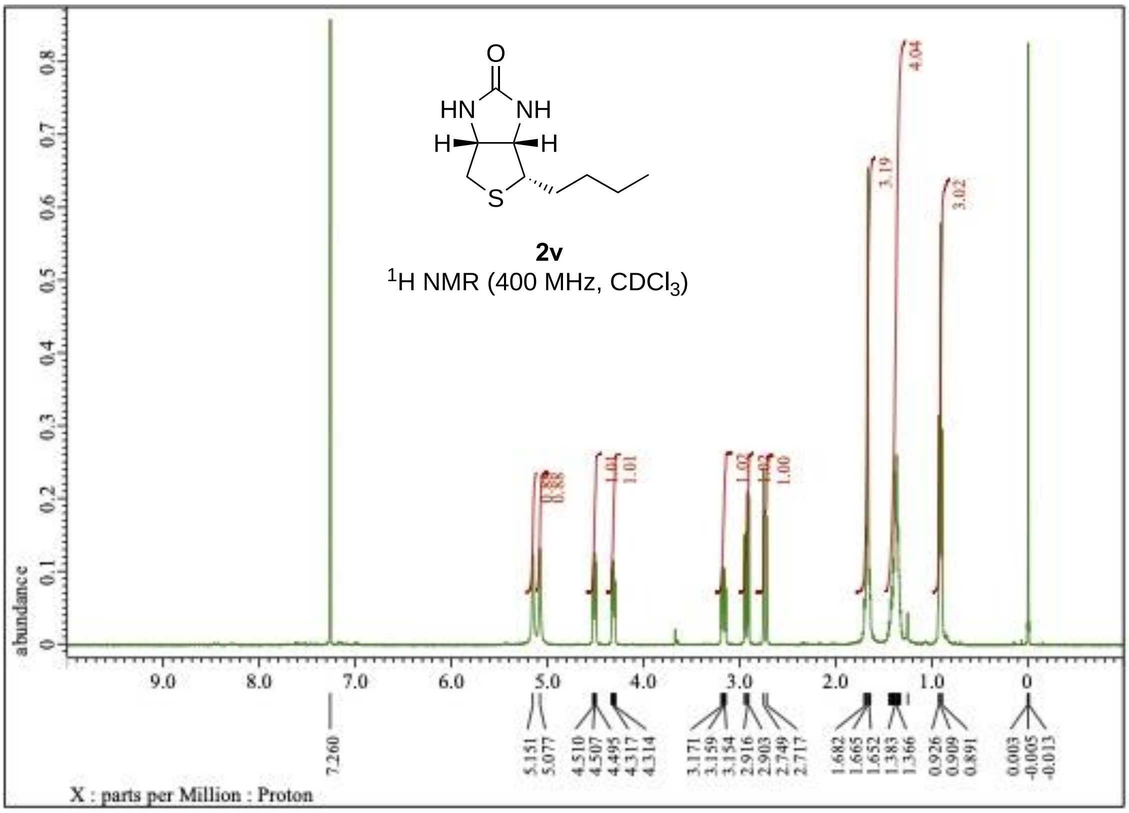
O
HN
NH
H
H
S
2v
1H NMR (400 MHz, CDCl3)
abundance
X : parts per Million : Proton
7.260
5.151
5.077
4.510
4.507
4.495
4.317
4.314
3.171
3.159
3.154
2.916
2.903
2.749
2.717
1.682
1.665
1.652
1.383
1.366
0.926
0.909
0.891
0.003
-0.005
-0.013
4.04
3.19
3.02
1.01
1.01
1.02
1.00

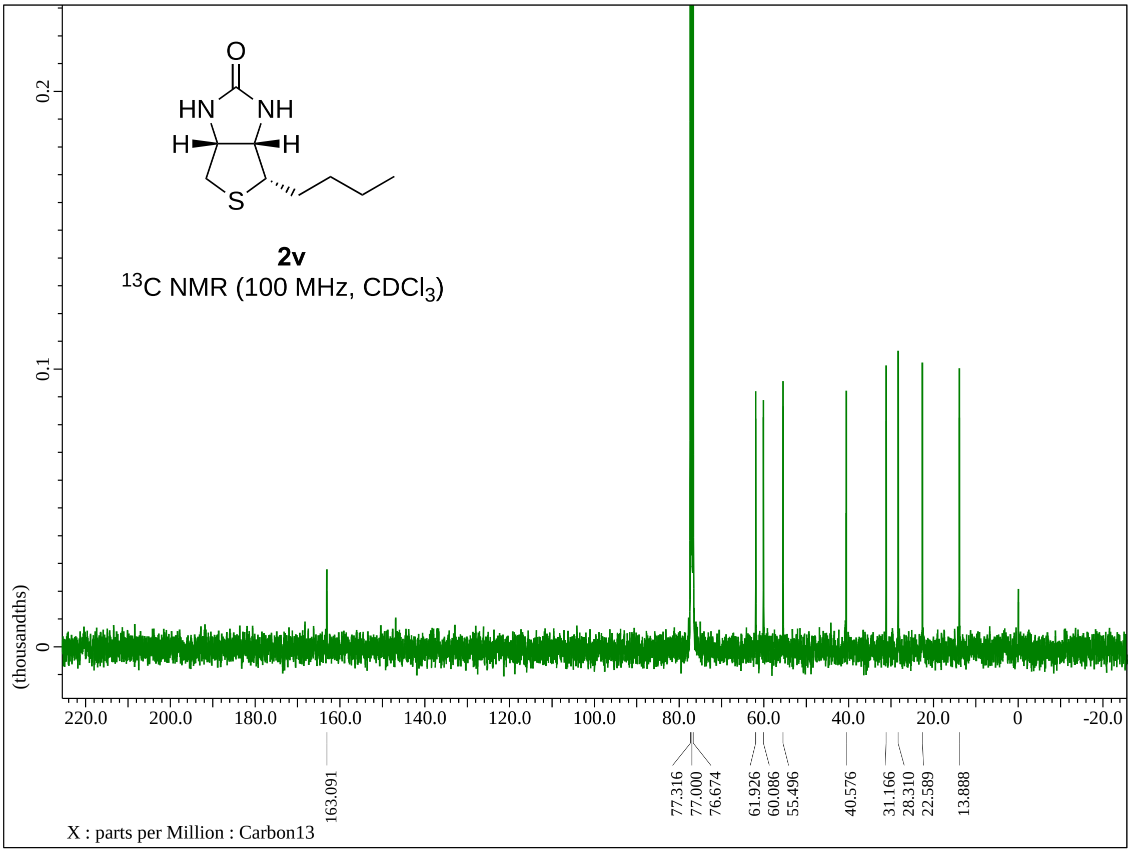
O
HN
NH
H
H
S
2v
13C NMR (100 MHz, CDCl3)
(thousandths)
X : parts per Million : Carbon13
163.091
77.316
77.000
76.674
61.926
60.086
55.496
40.576
31.166
28.310
22.589
13.888

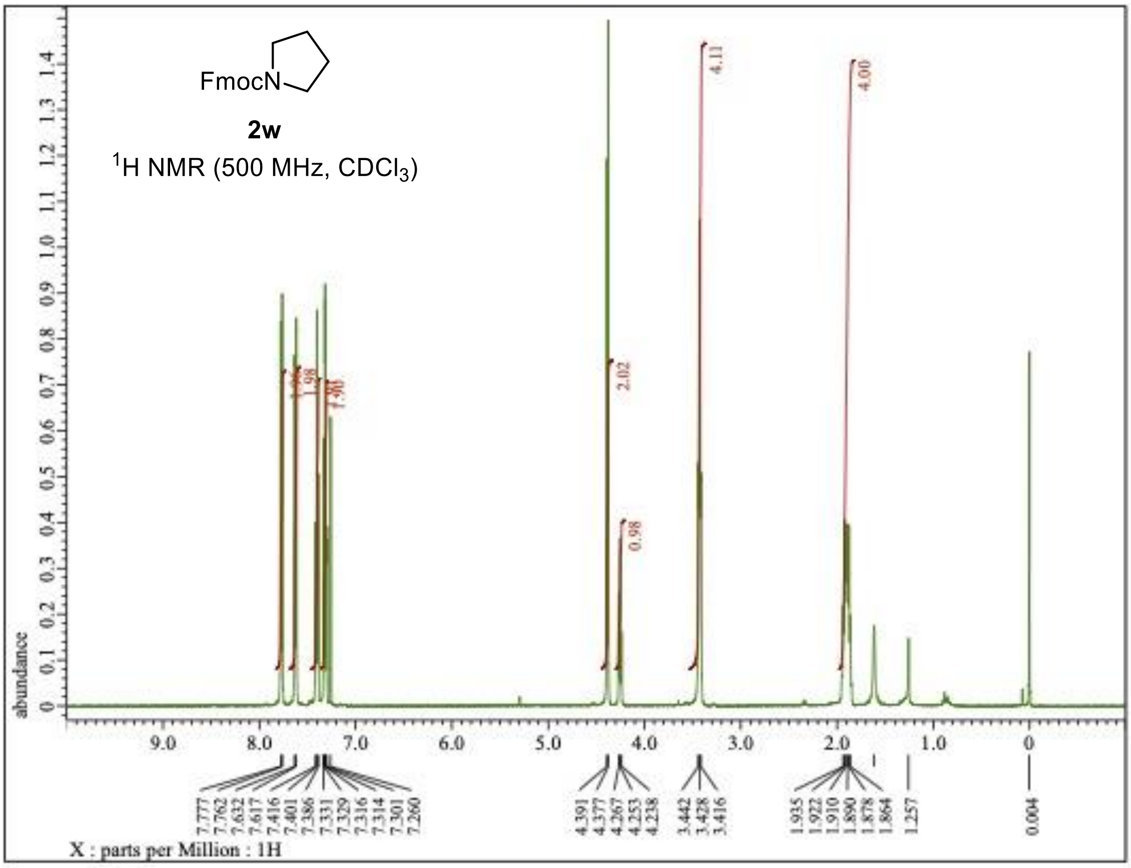
FmocN
2w
1H NMR (500 MHz, CDCl3)
abundance
X : parts per Million : 1H
7.777
7.762
7.632
7.617
7.416
7.401
7.386
7.331
7.329
7.316
7.314
7.301
7.260
4.391
4.377
4.267
4.253
4.238
3.442
3.428
3.416
1.935
1.922
1.910
1.890
1.878
1.864
1.257
0.004
2.02
0.98
4.11
4.00

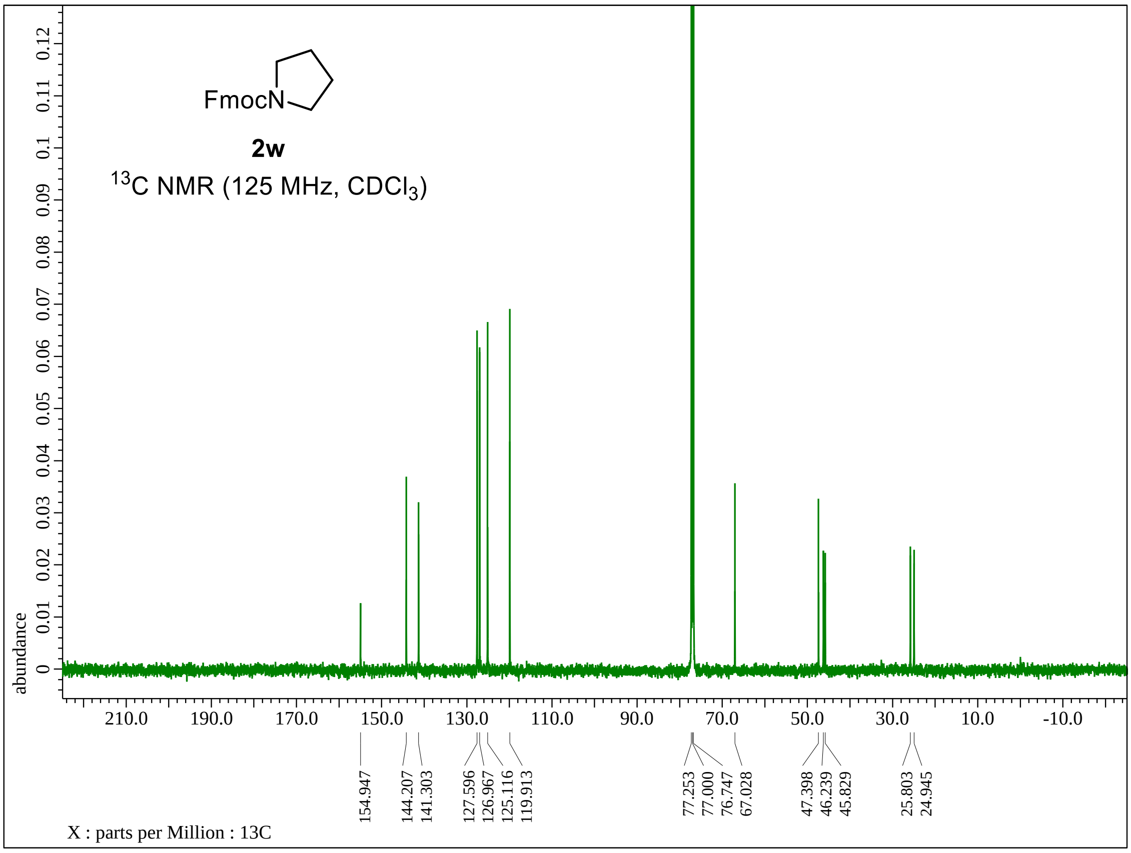
FmocN
2w
13C NMR (125 MHz, CDCl3)
abundance
X : parts per Million : 13C
154.947
144.207
141.303
127.596
126.967
125.116
119.913
77.253
77.000
76.747
67.028
47.398
46.239
45.829
25.803
24.945

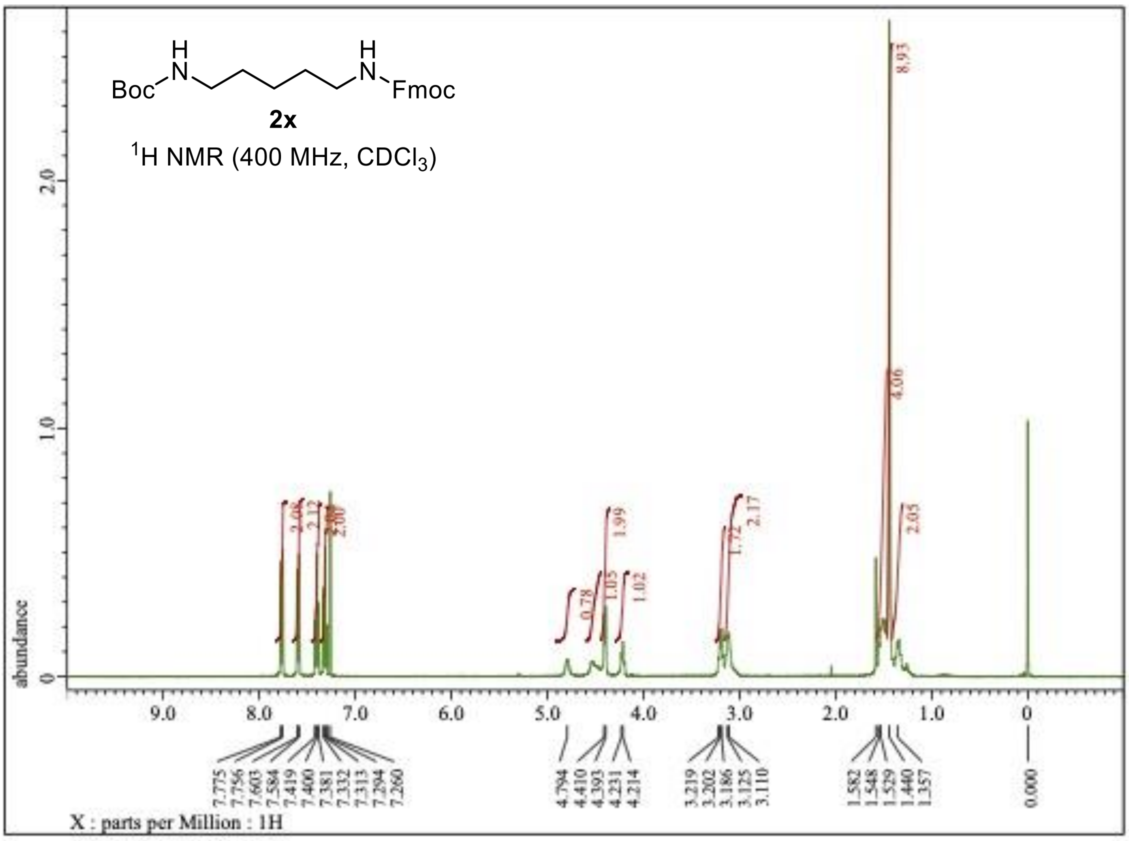
Boc
N
H
N
H
Fmoc
2x
¹H NMR (400 MHz, CDCl₃)
abundance
X : parts per Million : 1H

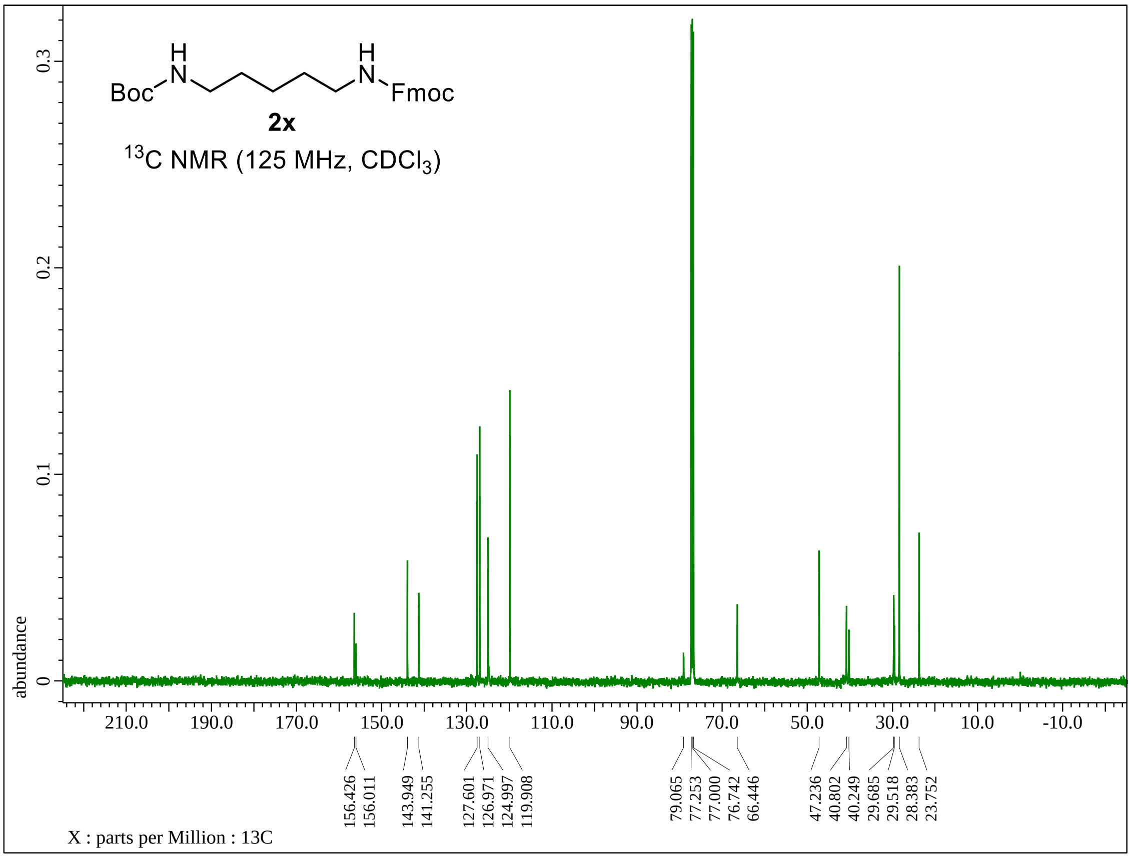
Boc
N
H
N
H
Fmoc
2x
¹³C NMR (125 MHz, CDCl₃)
abundance
X : parts per Million : 13C

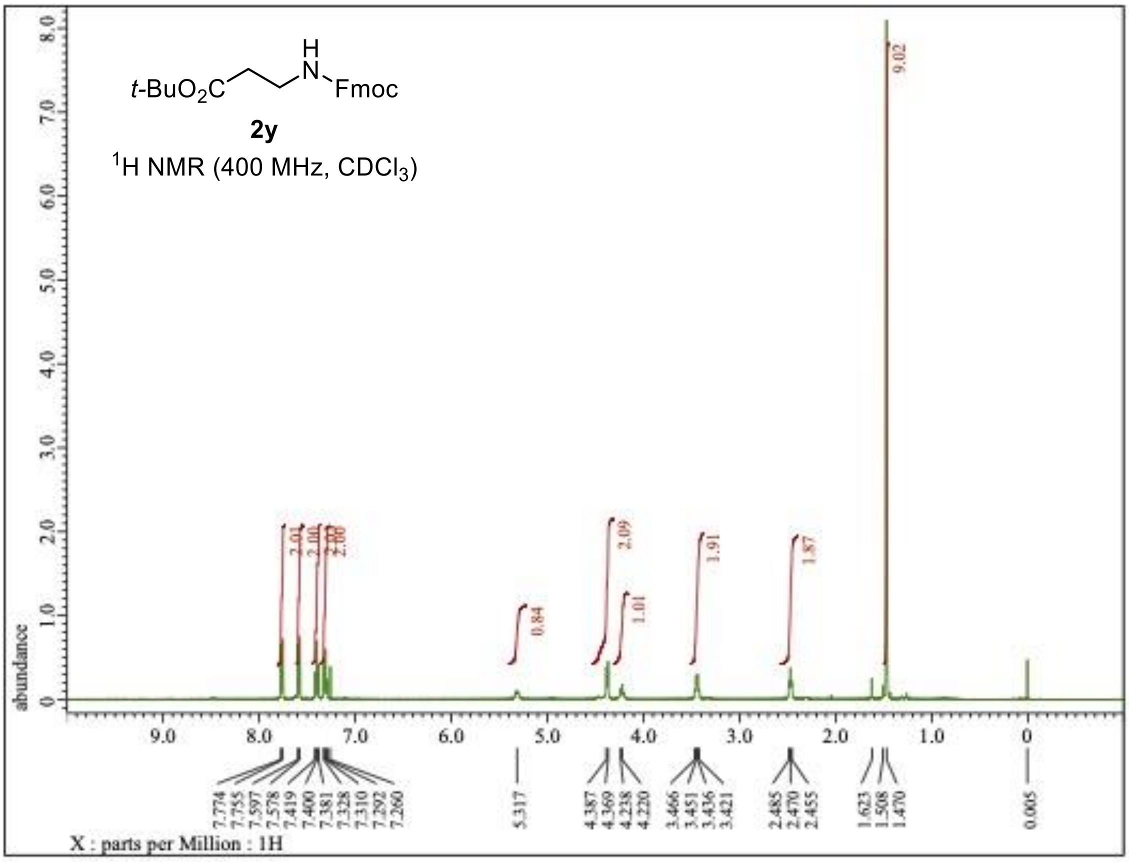
t-BuO2C
H
N
Fmoc
2y
1H NMR (400 MHz, CDCl3)
X : parts per Million : 1H
abundance

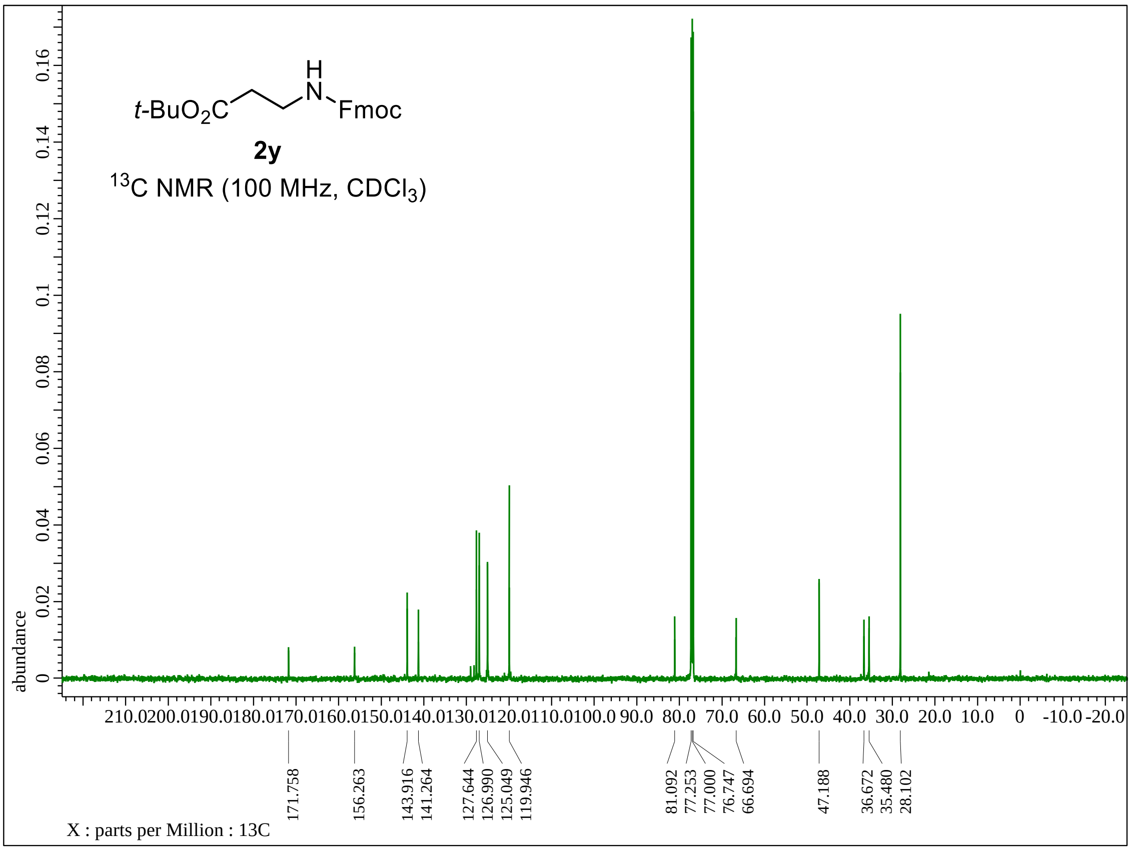
t-BuO2C
H
N
Fmoc
2y
13C NMR (100 MHz, CDCl3)
X : parts per Million : 13C
abundance
171.758
156.263
143.916
141.264
127.644
126.990
125.049
119.946
81.092
77.253
77.000
76.747
66.694
47.188
36.672
35.480
28.102

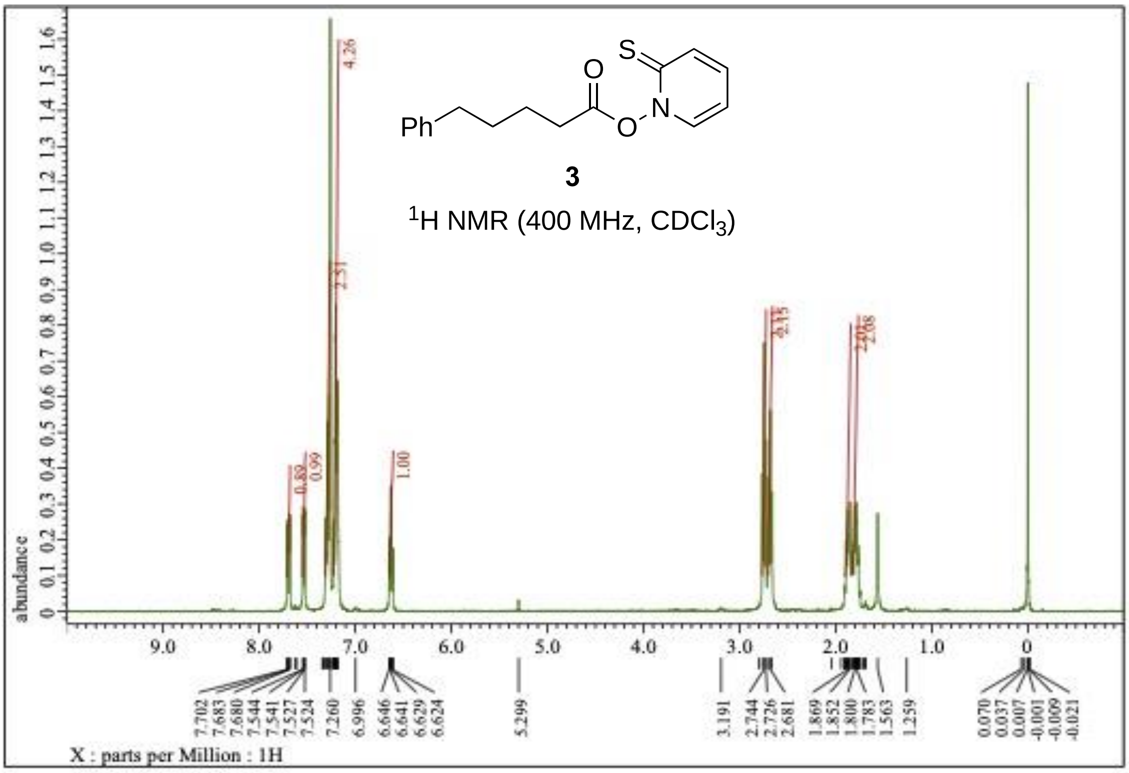
Ph
O
S
N
O
3
1H NMR (400 MHz, CDCl3)
abundance
0.89
0.99
4.26
2.31
1.00
2.13
2.01
2.08
7.702
7.683
7.680
7.544
7.541
7.527
7.524
7.260
6.996
6.646
6.641
6.629
6.624
5.299
3.191
2.744
2.726
2.681
1.869
1.852
1.800
1.783
1.563
1.259
0.070
0.037
0.007
-0.001
-0.009
-0.021
X : parts per Million : 1H

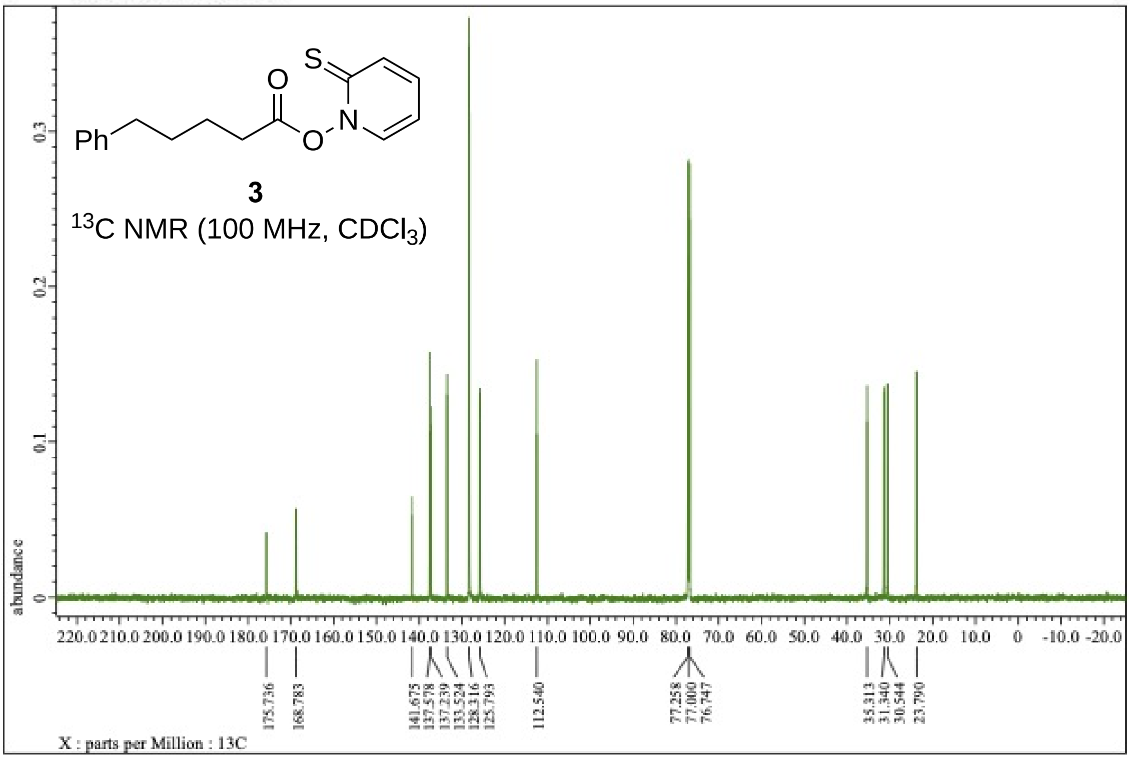
Ph
O
S
N
O
3
13C NMR (100 MHz, CDCl3)
abundance
175.736
168.783
141.675
137.578
137.239
133.524
128.316
125.793
112.540
77.258
77.000
76.747
35.313
31.340
30.544
23.790
X : parts per Million : 13C

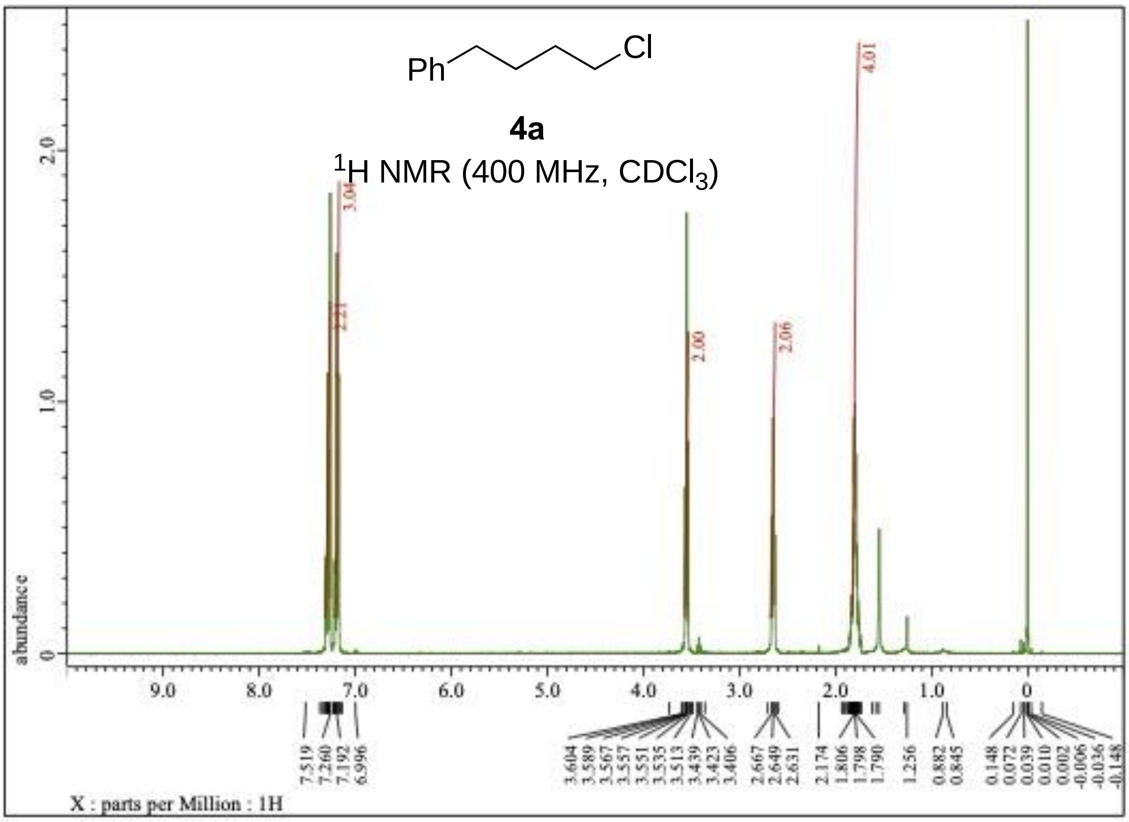
Ph
Cl
4a
1H NMR (400 MHz, CDCl3)
abundance
X : parts per Million : 1H

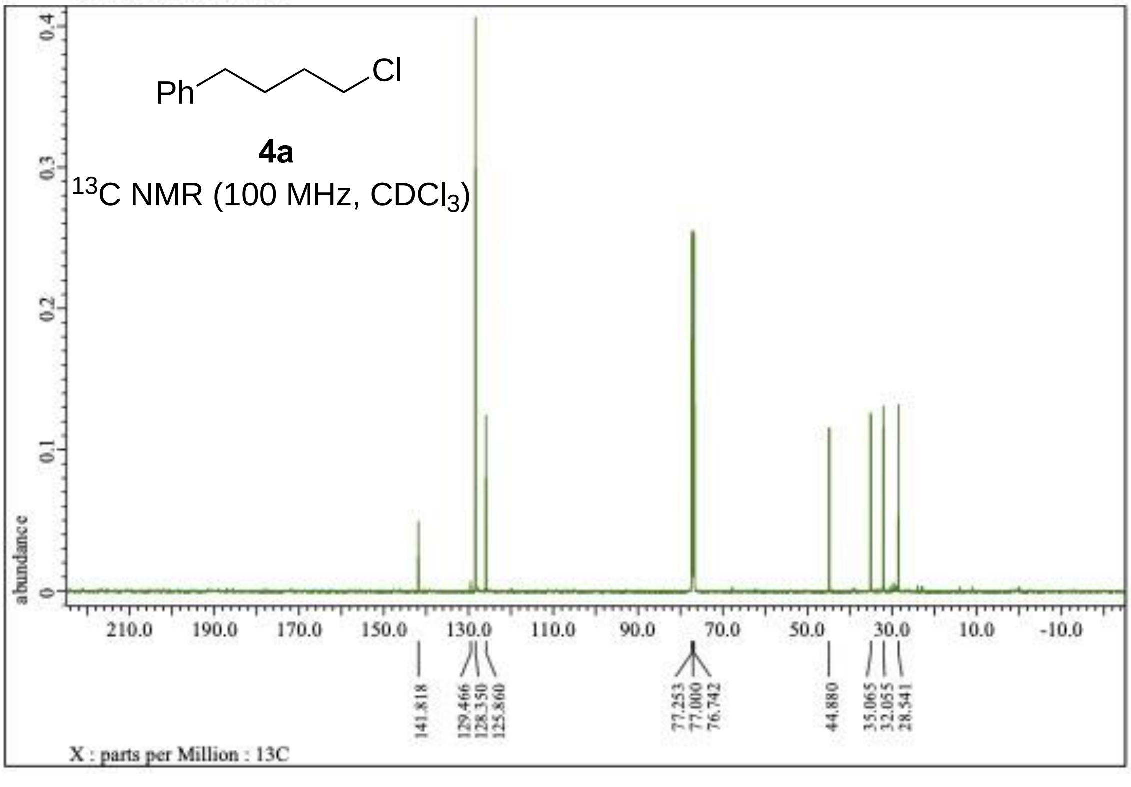
Ph
Cl
4a
13C NMR (100 MHz, CDCl3)
abundance
141.818
129.466
128.350
125.860
77.253
77.000
76.742
44.880
35.065
32.055
28.541
X : parts per Million : 13C

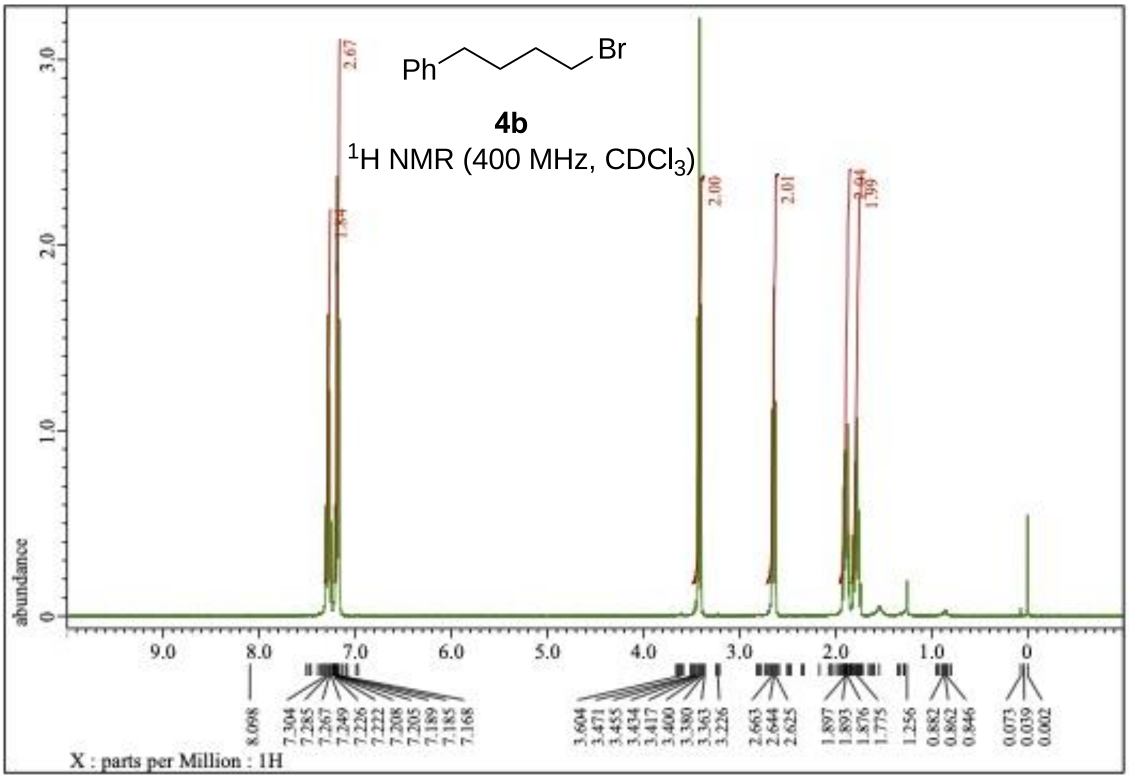
Ph
Br
4b
1H NMR (400 MHz, CDCl3)
abundance
X : parts per Million : 1H

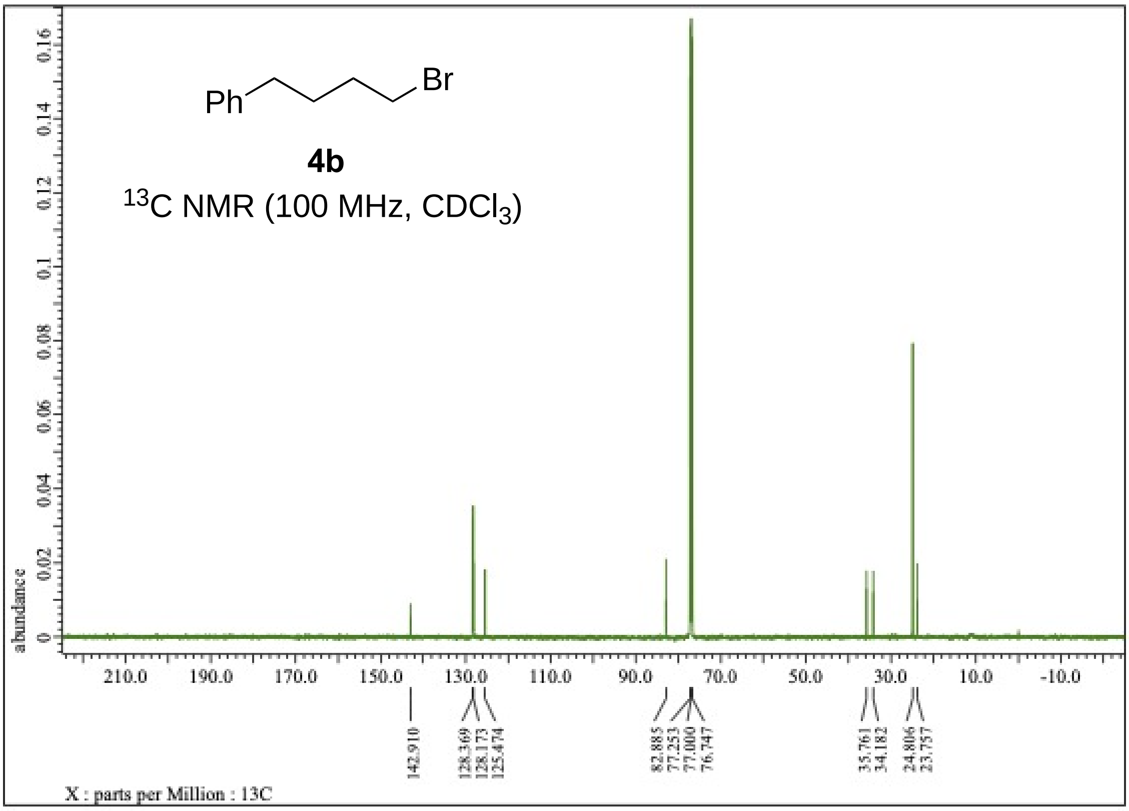
Ph
Br
4b
13C NMR (100 MHz, CDCl3)
abundance
142.910
128.369
128.173
125.474
82.885
77.253
77.000
76.747
35.761
34.182
24.806
23.757
X : parts per Million : 13C

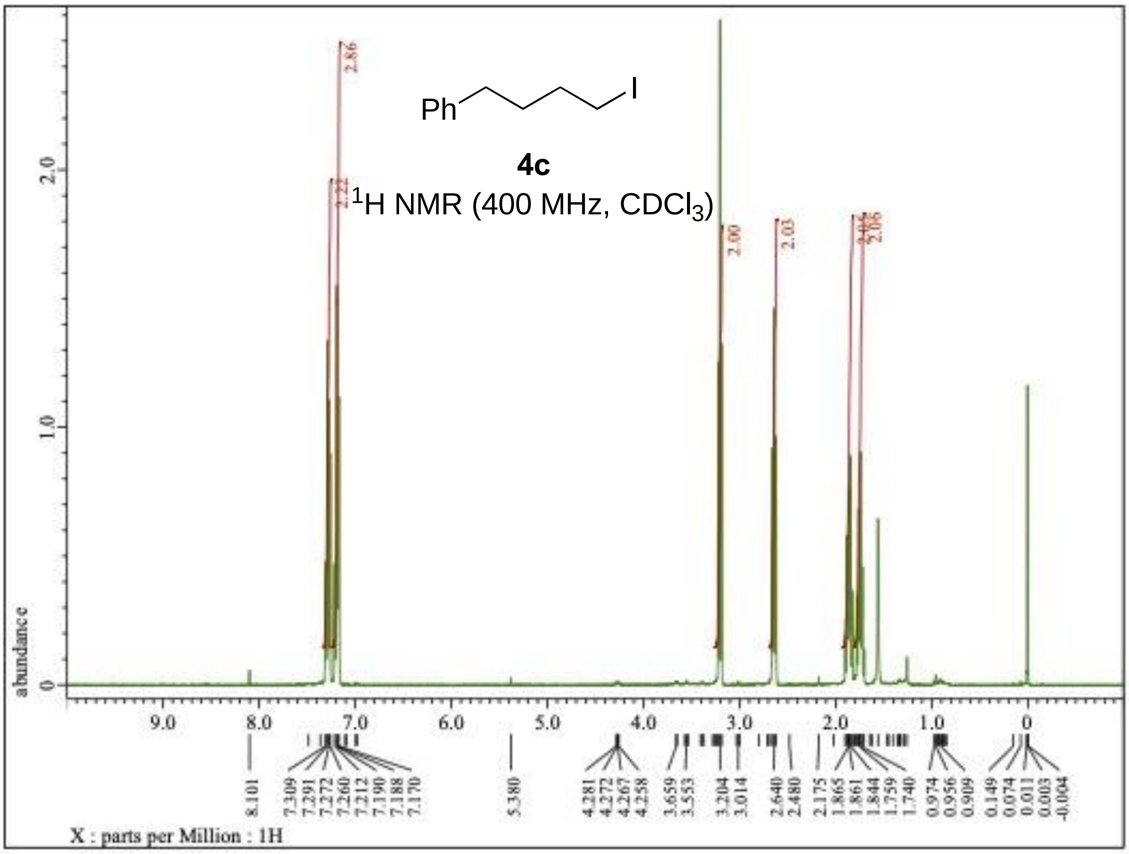
Ph
I
4c
1H NMR (400 MHz, CDCl3)
abundance
X : parts per Million : 1H
8.101
7.309 7.291 7.272 7.260 7.212 7.190 7.188 7.170
5.380
4.281 4.272 4.267 4.258
3.659 3.553
3.204 3.014
2.640 2.480
2.175 1.865 1.861 1.844 1.759 1.740
0.974 0.956 0.909
0.149 0.074 0.011 0.003 -0.004
2.86
2.22
2.00
2.03
2.06

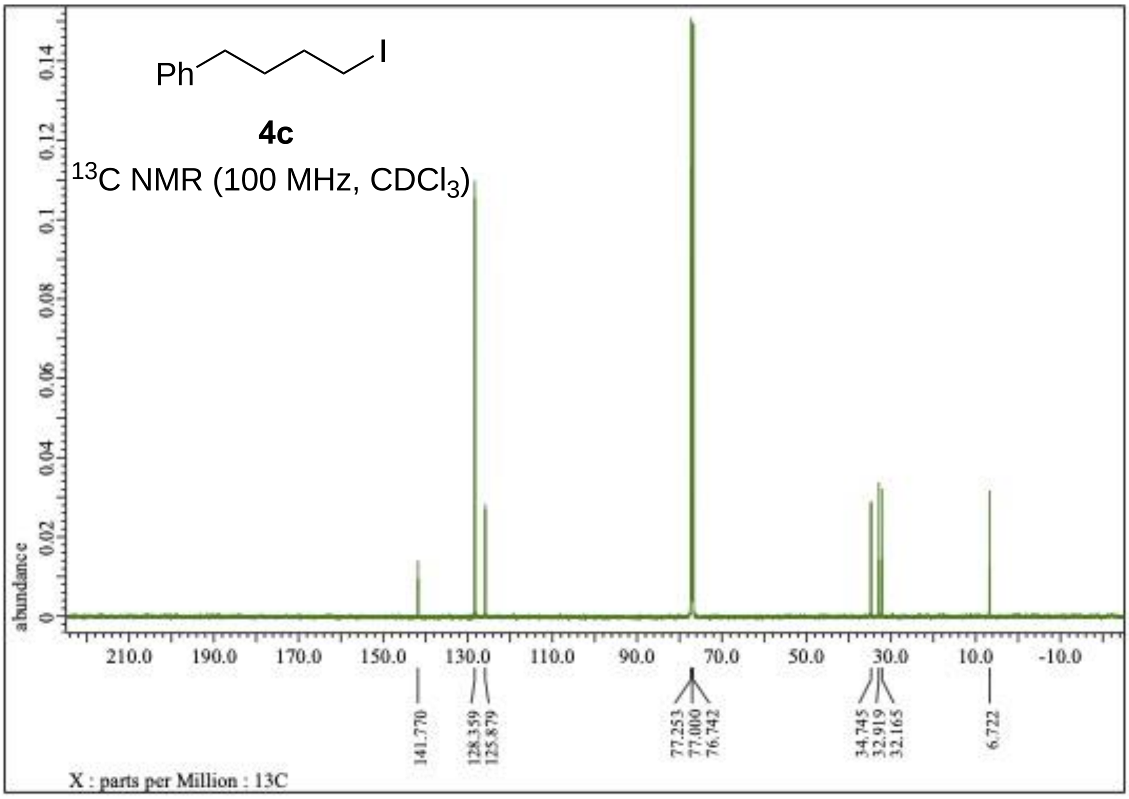
Ph
I
4c
13C NMR (100 MHz, CDCl3)
abundance
X : parts per Million : 13C
141.770
128.359 125.879
77.253 77.000 76.742
34.745 32.919 32.165
6.722

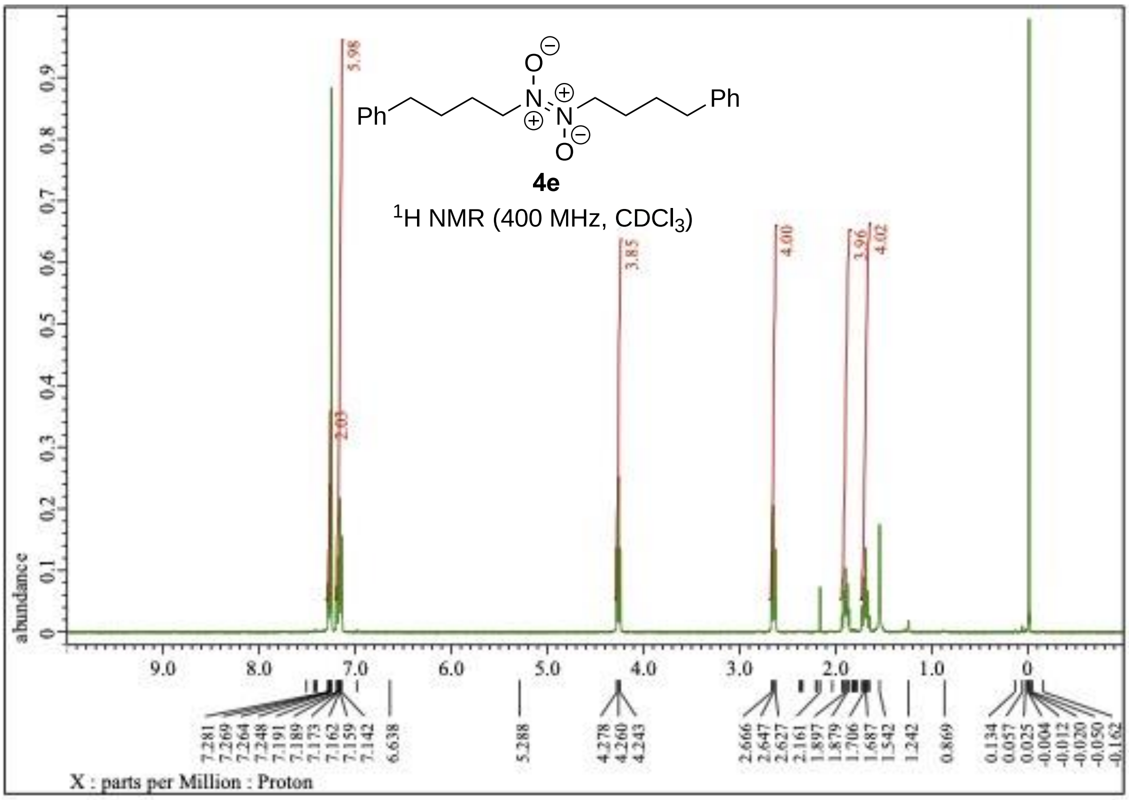
4e
^{1}H NMR (400 MHz, CDCl$_3$)
X : parts per Million : Proton
abundance

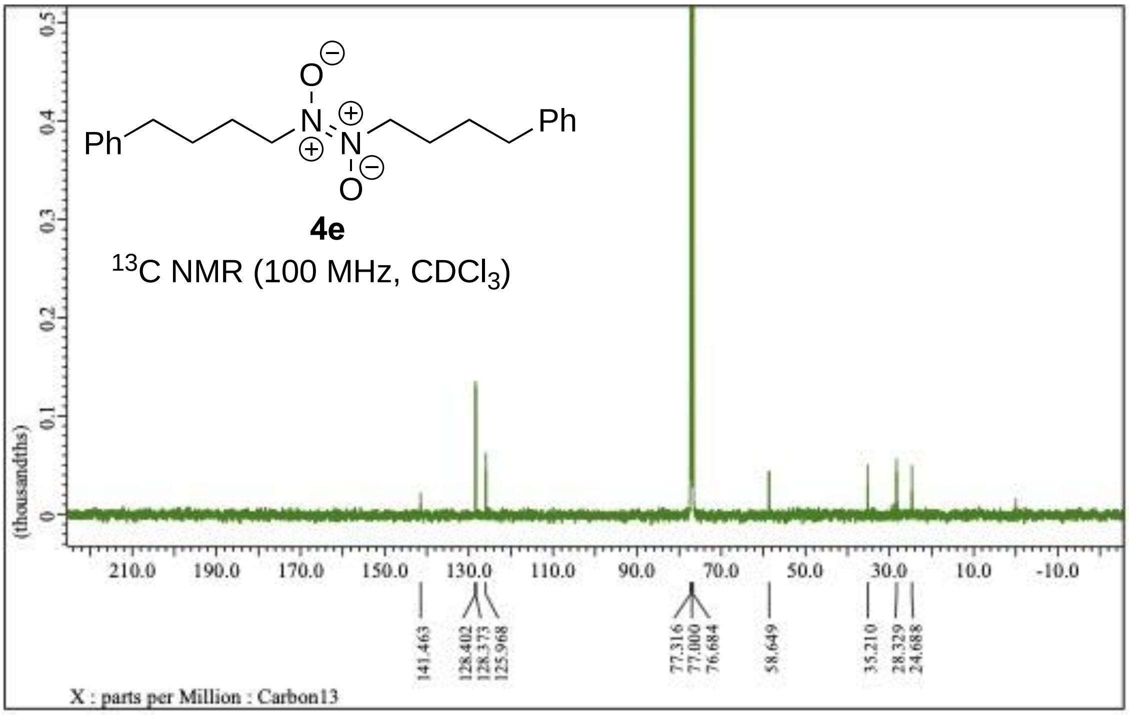
4e
^{13}C NMR (100 MHz, CDCl$_3$)
X : parts per Million : Carbon13
(thousandths)

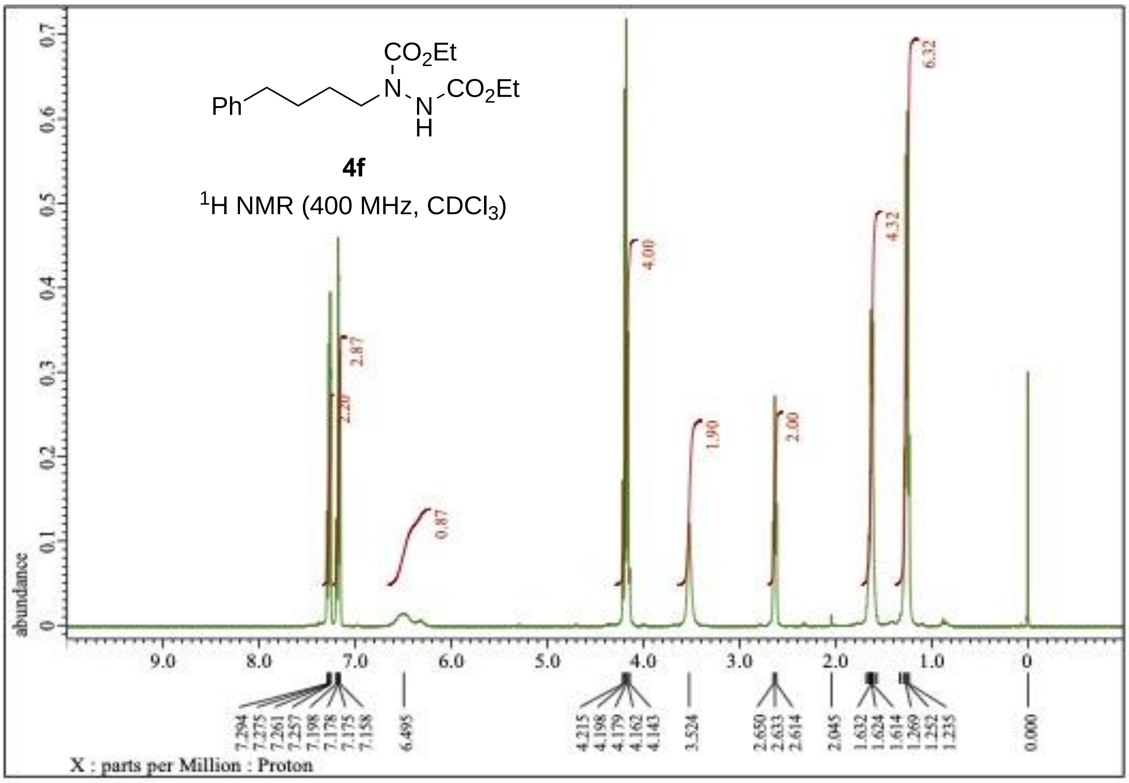
CO2Et
Ph
N
N
CO2Et
H
4f
1H NMR (400 MHz, CDCl3)
X : parts per Million : Proton
abundance
7.294
7.275
7.261
7.257
7.198
7.178
7.175
7.158
6.495
4.215
4.198
4.179
4.162
4.143
3.524
2.650
2.633
2.614
2.045
1.632
1.624
1.614
1.269
1.252
1.235
0.000
2.20
2.87
0.87
4.00
1.90
2.00
4.32
6.32

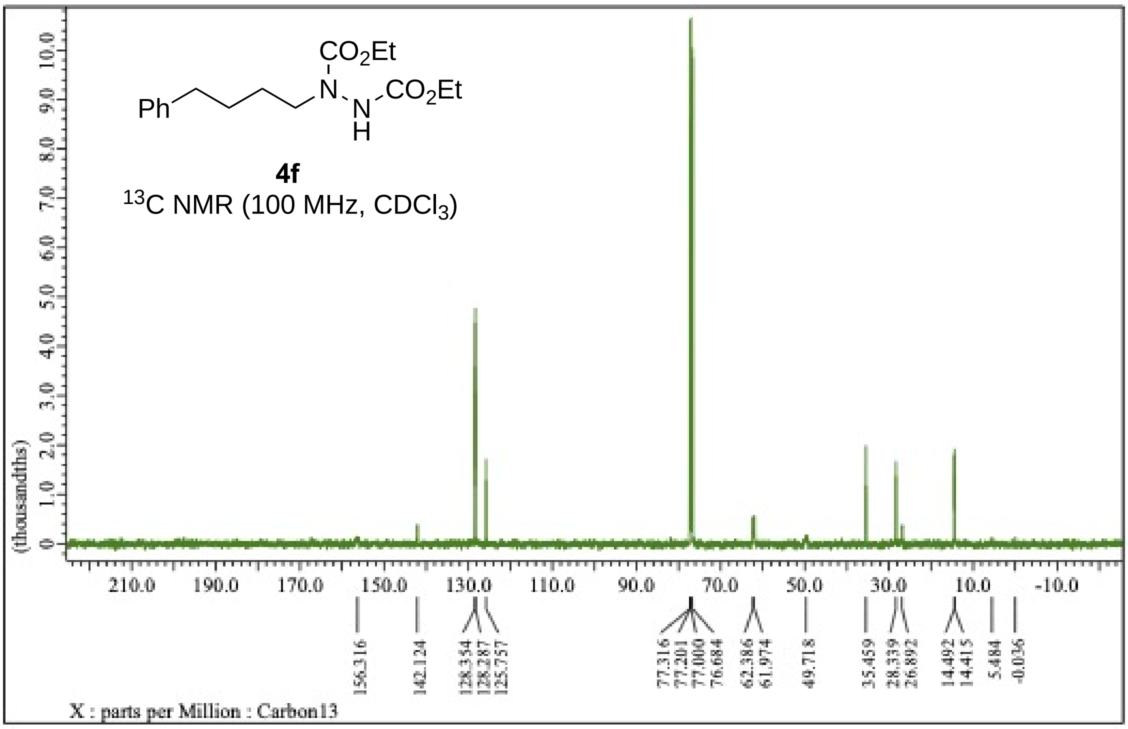
CO2Et
Ph
N
N
CO2Et
H
4f
13C NMR (100 MHz, CDCl3)
X : parts per Million : Carbon13
(thousandths)
156.316
142.124
128.354
128.287
125.757
77.316
77.201
77.000
76.684
62.386
61.974
49.718
35.459
28.339
26.892
14.492
14.415
5.484
-0.036

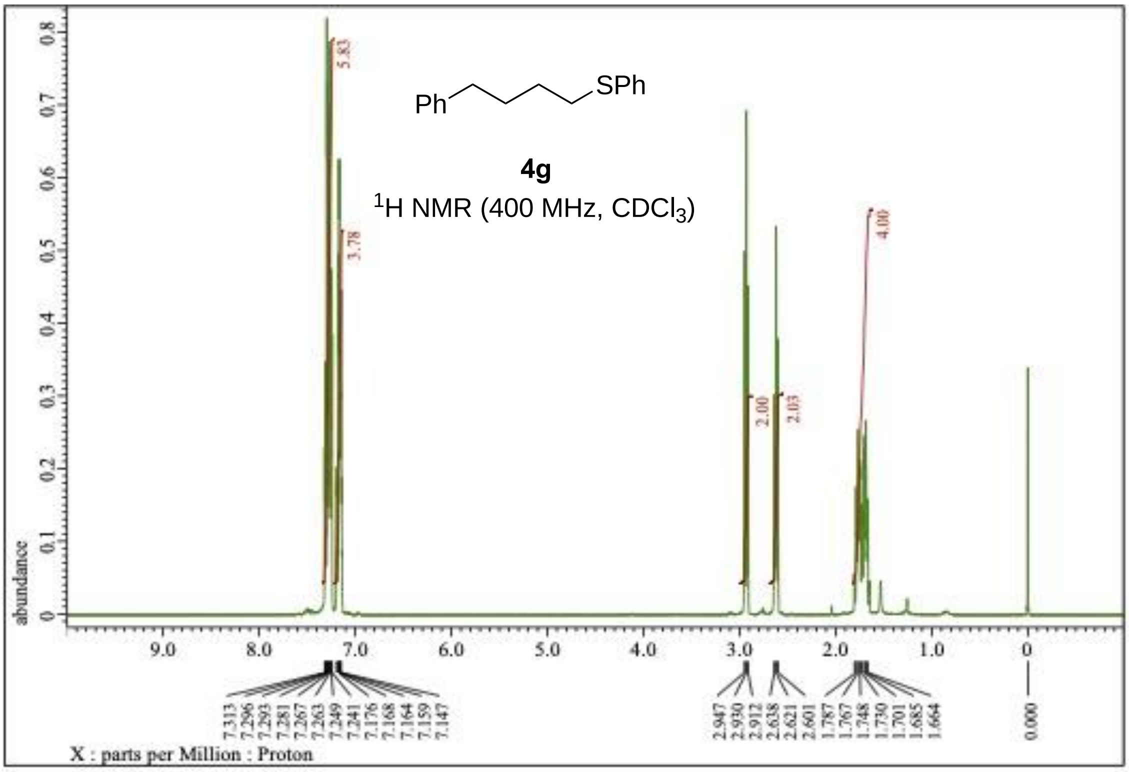
Ph SPh
4g
^{1}H NMR (400 MHz, CDCl$_3$)
5.83
3.78
2.00
2.03
4.00
abundance
9.0 8.0 7.0 6.0 5.0 4.0 3.0 2.0 1.0 0
7.313 7.296 7.293 7.281 7.267 7.263 7.249 7.241 7.176 7.168 7.164 7.159 7.147
2.947 2.930 2.912 2.638 2.621 2.601 1.787 1.767 1.748 1.730 1.701 1.685 1.664
0.000
X : parts per Million : Proton

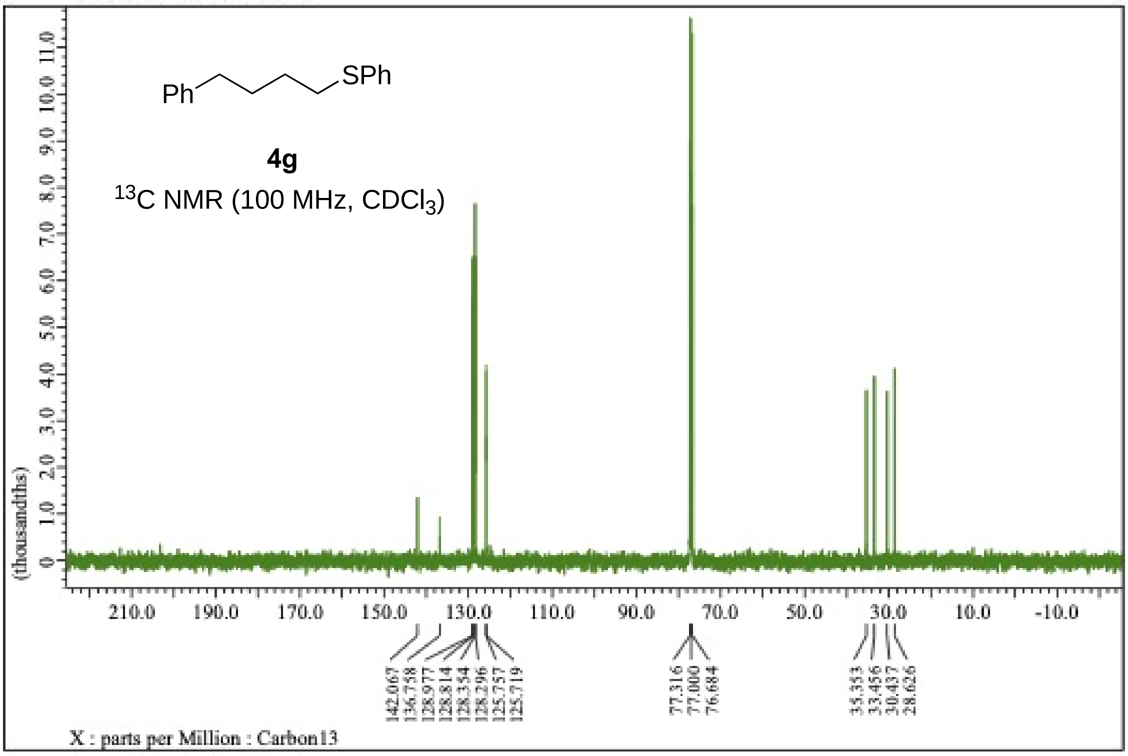
Ph SPh
4g
^{13}C NMR (100 MHz, CDCl$_3$)
(thousandths)
210.0 190.0 170.0 150.0 130.0 110.0 90.0 70.0 50.0 30.0 10.0 -10.0
142.067 136.758 128.977 128.814 128.354 128.296 125.757 125.719
77.316 77.000 76.684
35.353 33.456 30.437 28.626
X : parts per Million : Carbon13

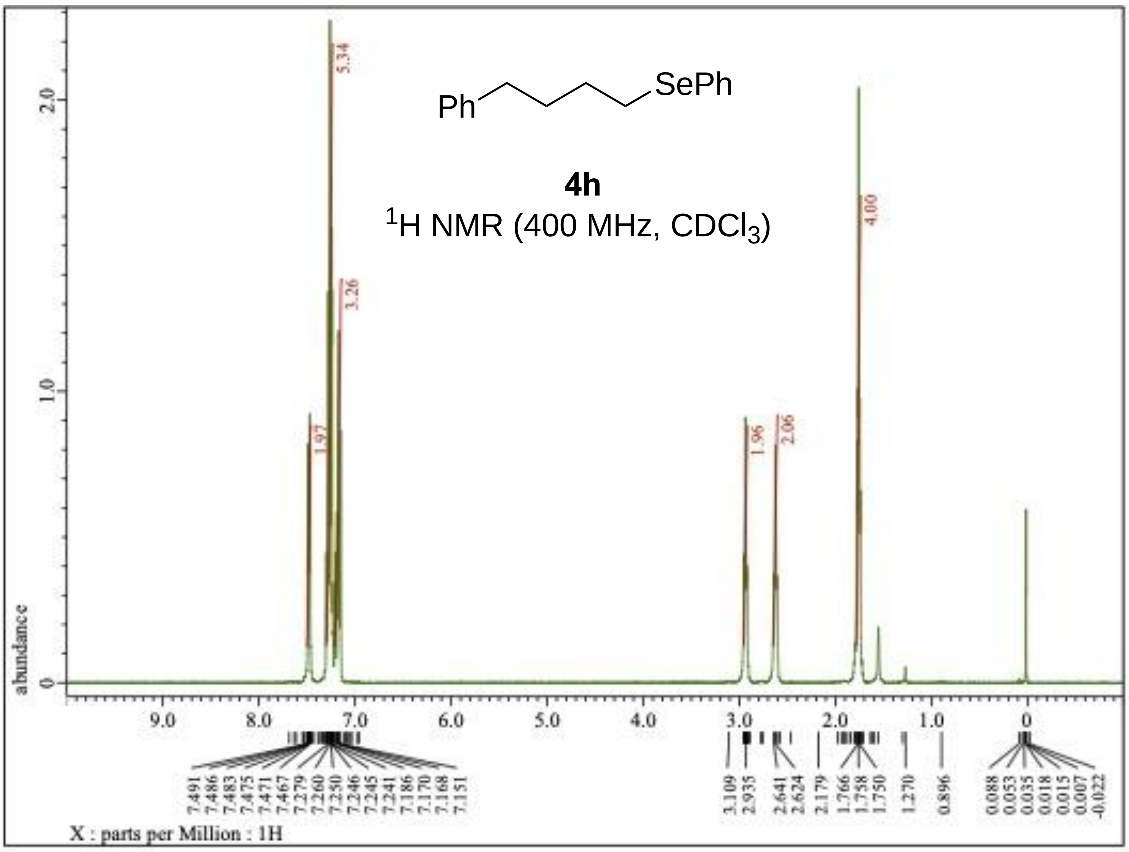
Ph SePh
4h
1H NMR (400 MHz, CDCl3)
X : parts per Million : 1H
abundance

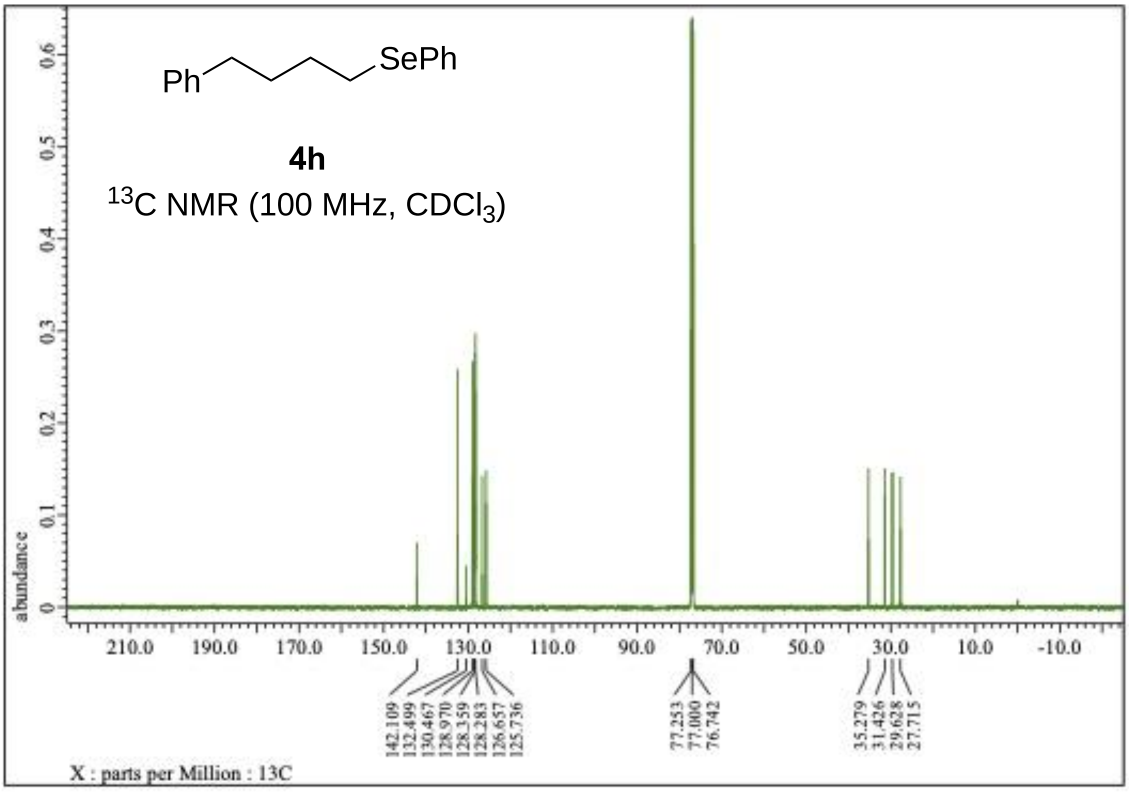
Ph SePh
4h
13C NMR (100 MHz, CDCl3)
X : parts per Million : 13C
abundance

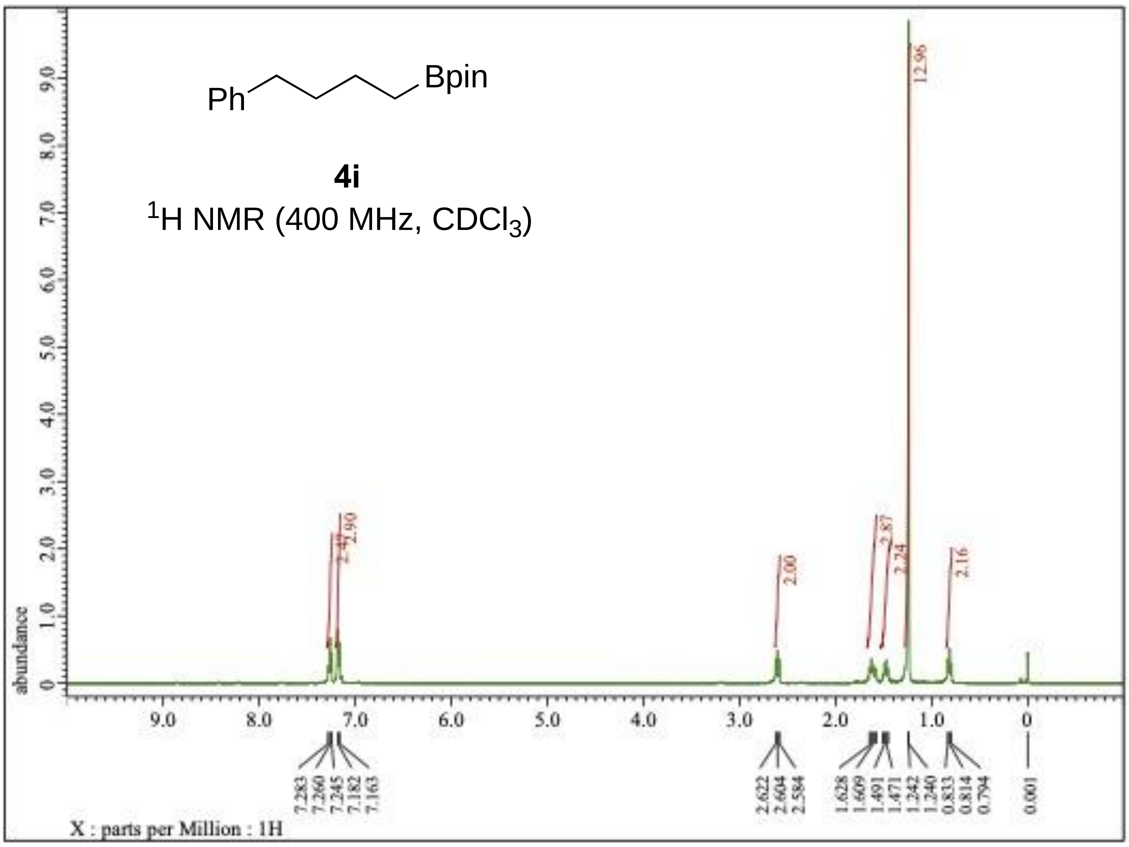
Ph
Bpin
4i
1H NMR (400 MHz, CDCl3)
abundance
X : parts per Million : 1H
7.283
7.260
7.245
7.182
7.163
2.622
2.604
2.584
1.628
1.609
1.491
1.471
1.342
1.240
0.833
0.814
0.794
0.001
12.96
2.00
2.87
2.24
2.16
2.90

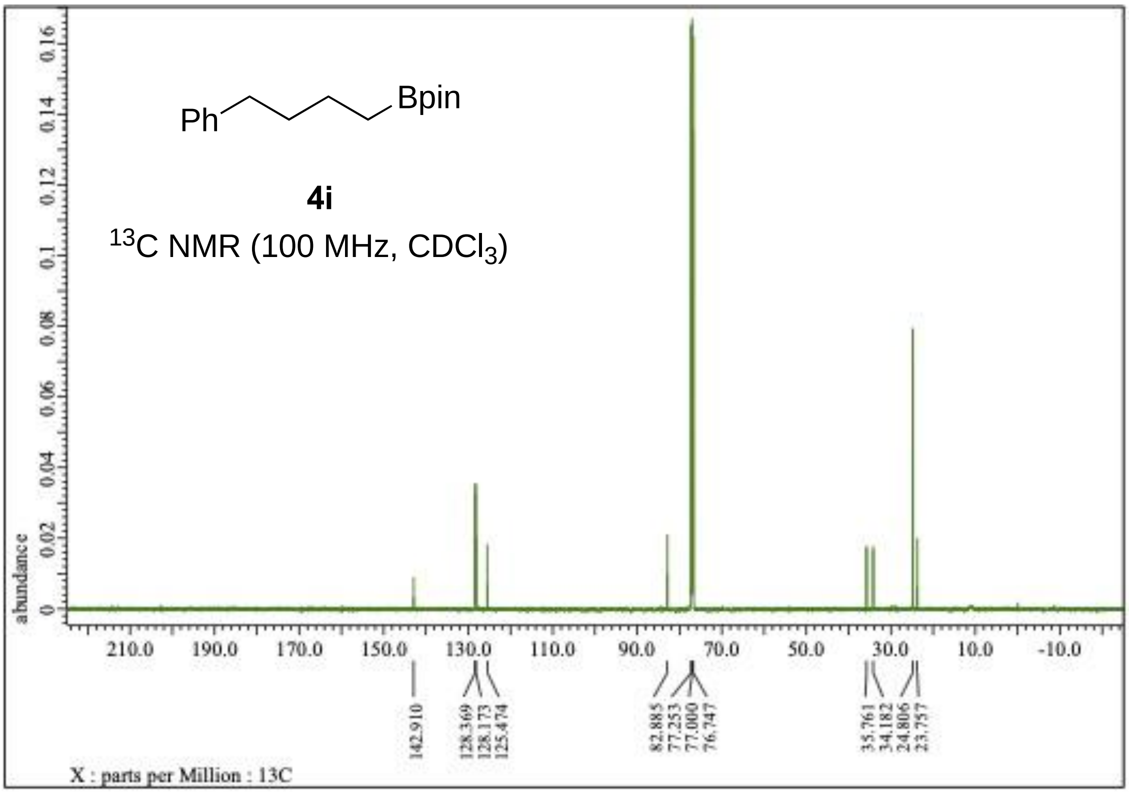
Ph
Bpin
4i
13C NMR (100 MHz, CDCl3)
abundance
X : parts per Million : 13C
142.910
128.369
128.173
125.474
82.885
77.253
77.000
76.747
35.761
34.182
24.806
23.757

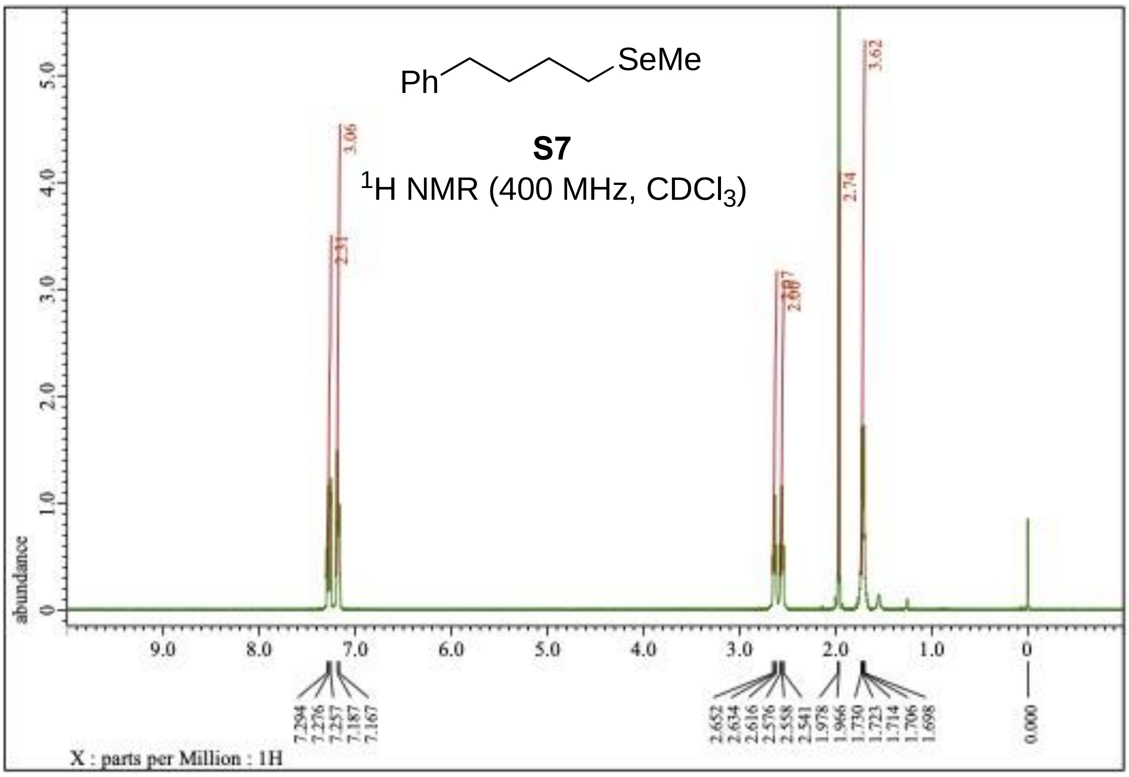
Ph SeMe
S7
1H NMR (400 MHz, CDCl3)
abundance
X : parts per Million : 1H
7.294
7.276
7.257
7.187
7.167
2.652
2.634
2.616
2.576
2.558
2.541
1.978
1.966
1.730
1.723
1.714
1.706
1.698
0.000

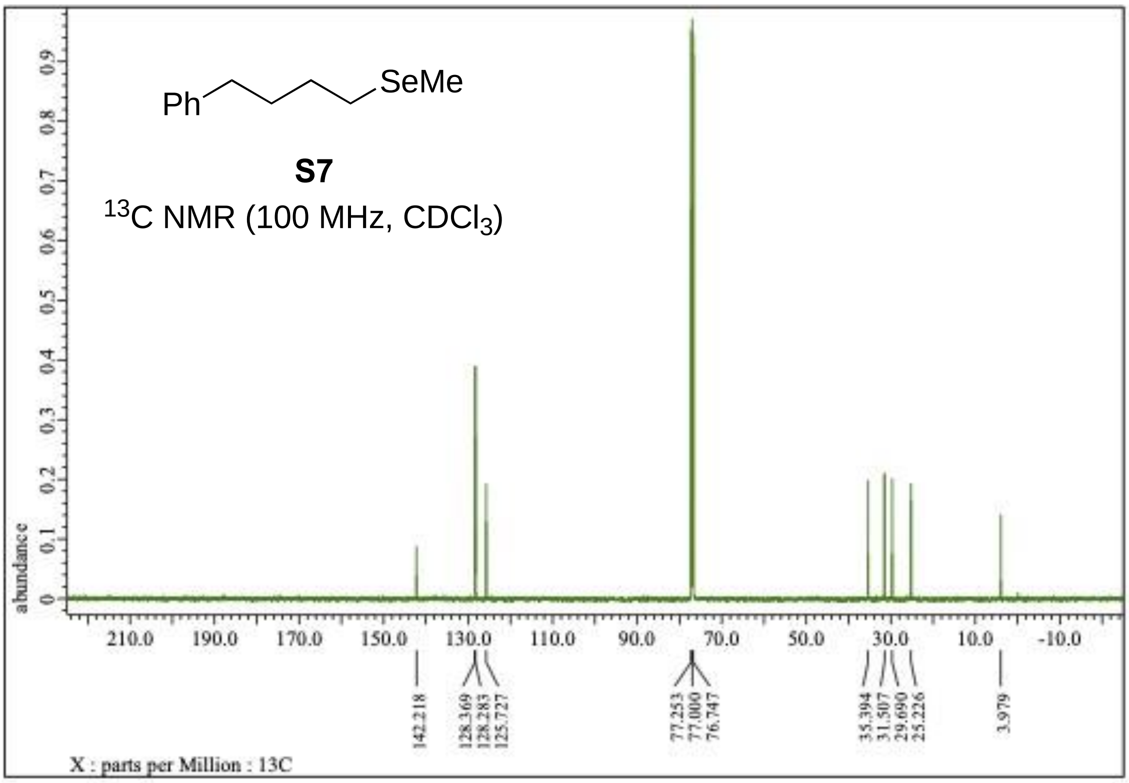
Ph SeMe
S7
13C NMR (100 MHz, CDCl3)
abundance
X : parts per Million : 13C
142.218
128.369
128.283
125.727
77.253
77.000
76.747
35.394
31.507
29.690
25.226
3.979

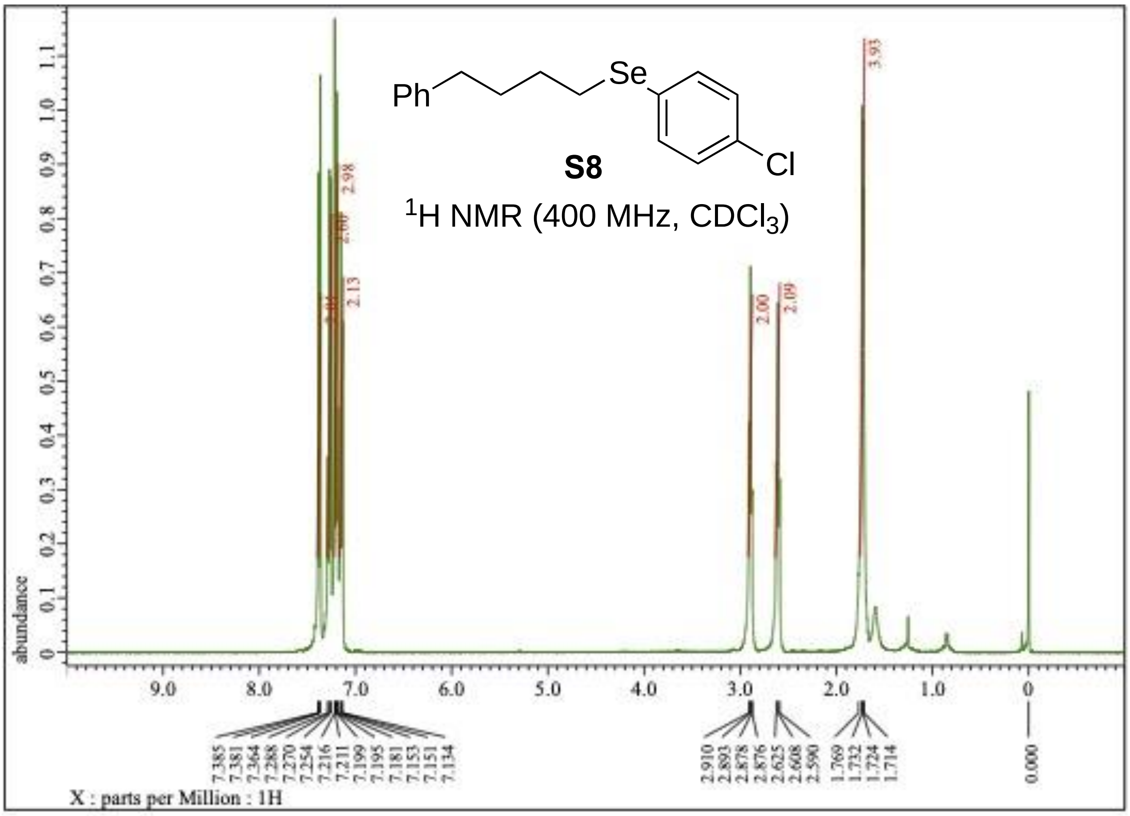
Ph
Se
Cl
S8
1H NMR (400 MHz, CDCl3)
abundance
X : parts per Million : 1H

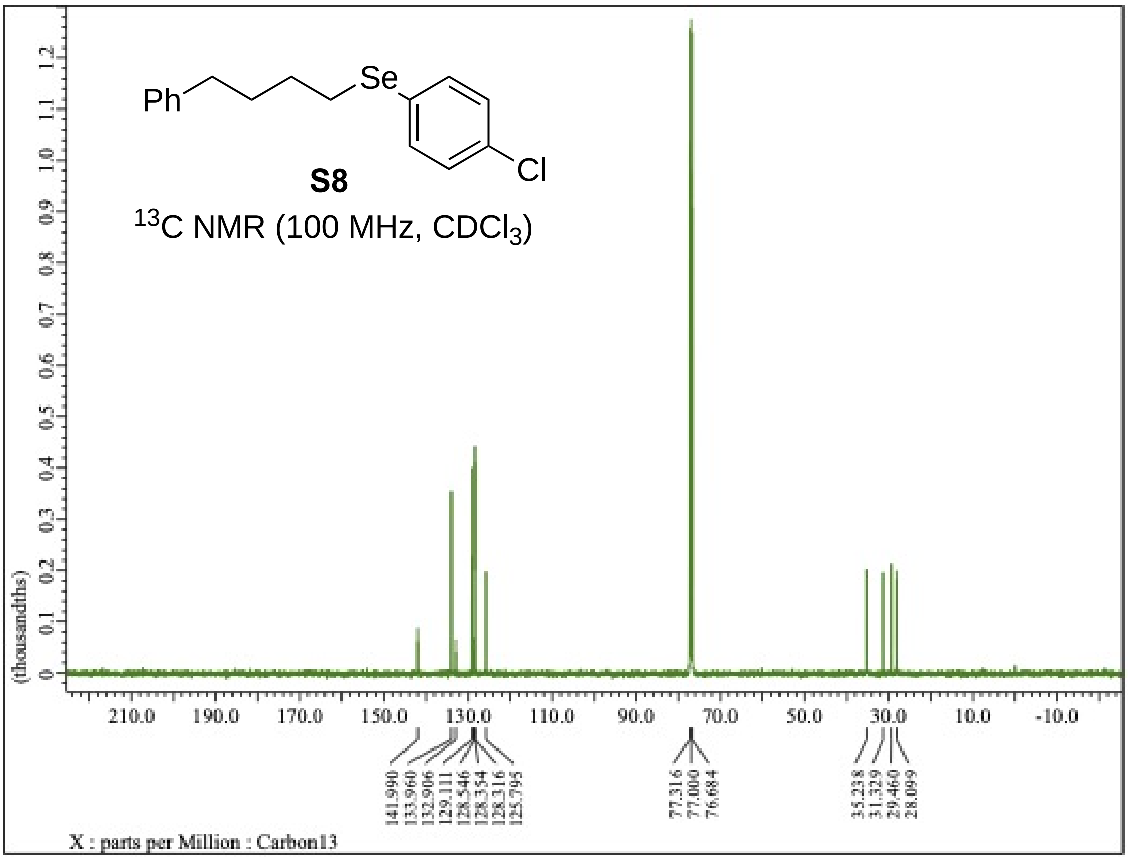
Ph
Se
Cl
S8
13C NMR (100 MHz, CDCl3)
(thousandths)
X : parts per Million : Carbon13

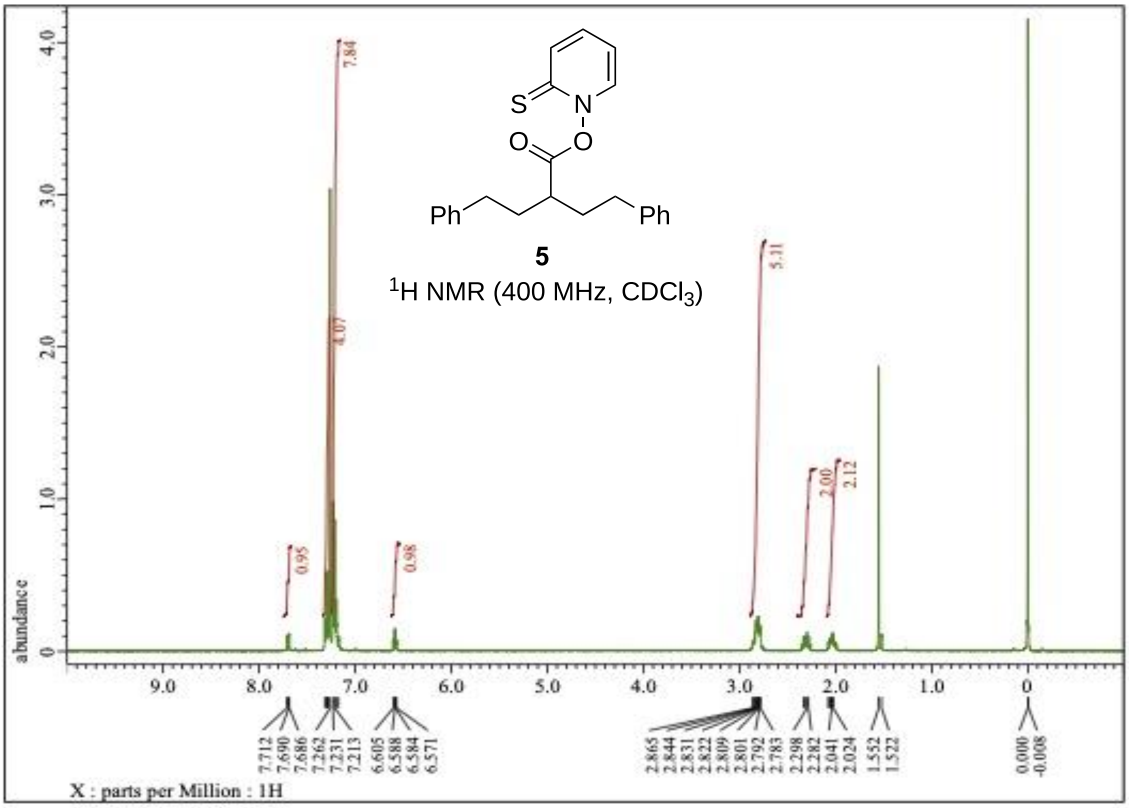
S
N
O
O
Ph
Ph
5
1H NMR (400 MHz, CDCl3)
abundance
X : parts per Million : 1H
7.712
7.690
7.686
7.262
7.231
7.213
6.605
6.588
6.584
6.571
2.865
2.844
2.831
2.822
2.809
2.801
2.792
2.783
2.298
2.282
2.041
2.024
1.552
1.522
0.000
-0.008
0.95
7.84
4.07
0.98
5.31
2.00
2.12

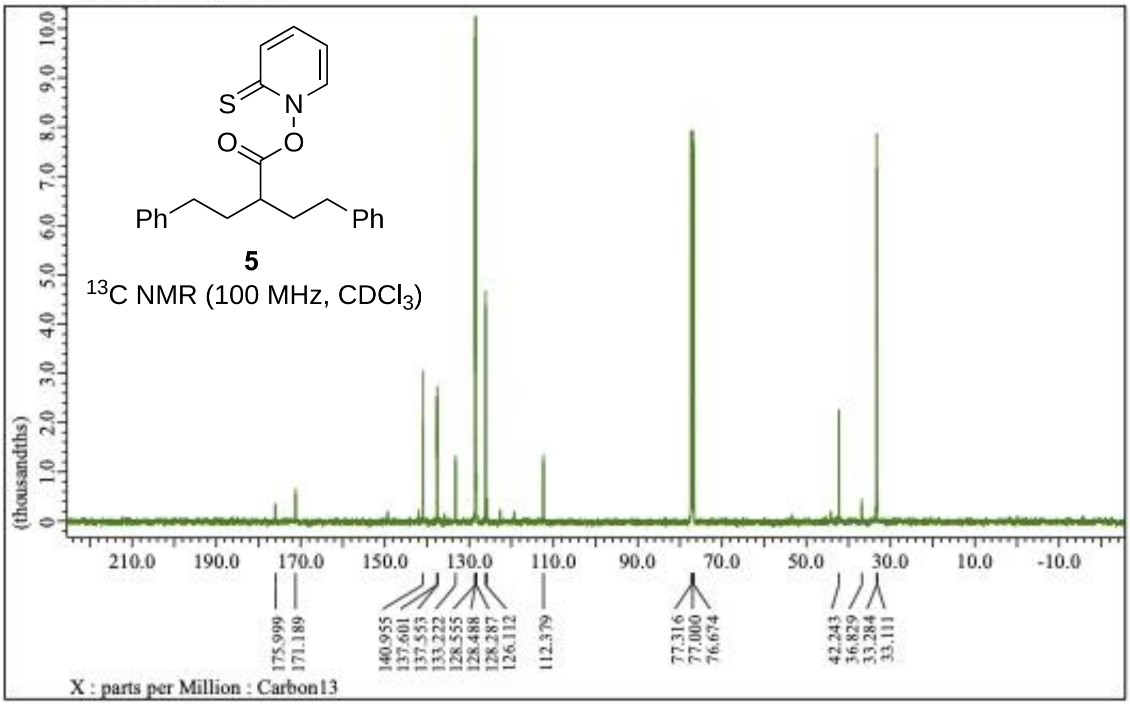
S
N
O
O
Ph
Ph
5
13C NMR (100 MHz, CDCl3)
(thousandths)
X : parts per Million : Carbon13
175.999
171.189
140.955
137.601
137.553
133.222
128.555
128.488
128.287
126.112
112.379
77.316
77.000
76.674
42.243
36.829
33.284
33.111

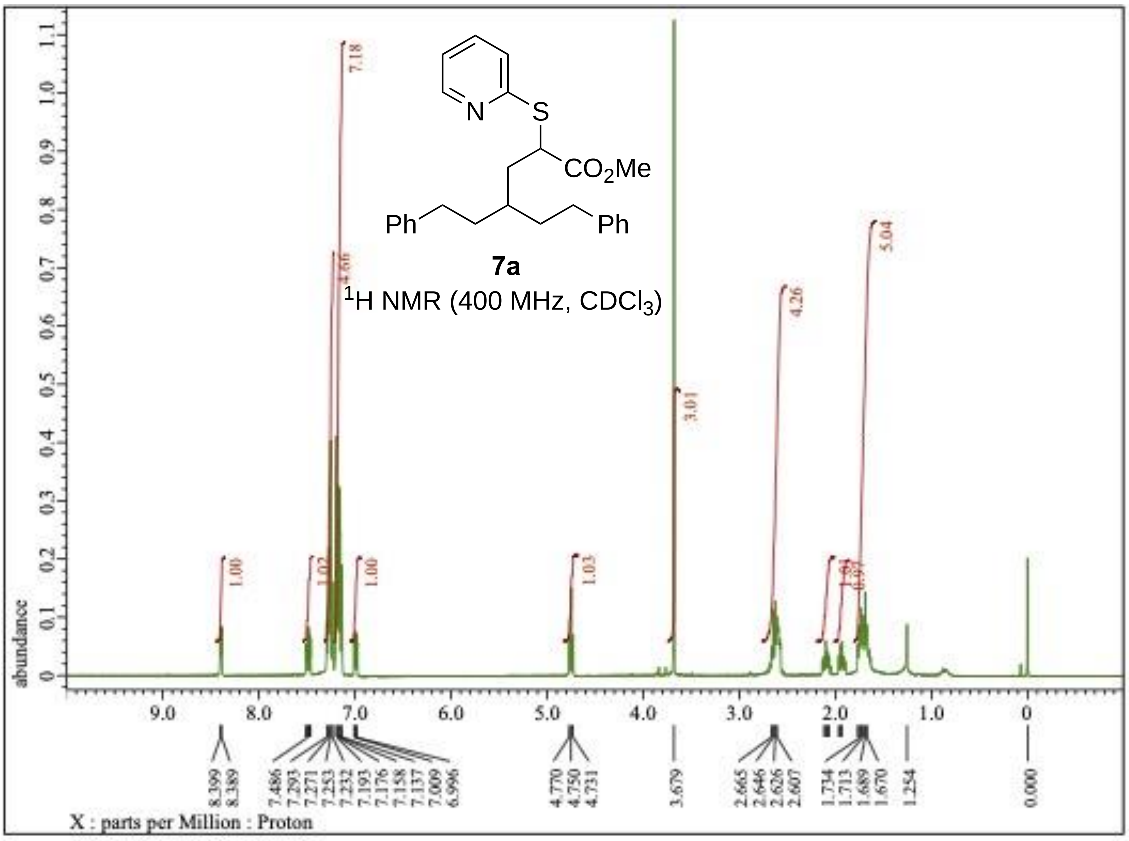

7a
1H NMR (400 MHz, CDCl3)
abundance
X : parts per Million : Proton
8.399 8.389
7.486 7.293 7.271 7.253 7.232 7.193 7.176 7.158 7.137 7.009 6.996
4.770 4.750 4.731
3.679
2.665 2.646 2.626 2.607
1.734 1.713 1.689 1.670
1.254
0.000

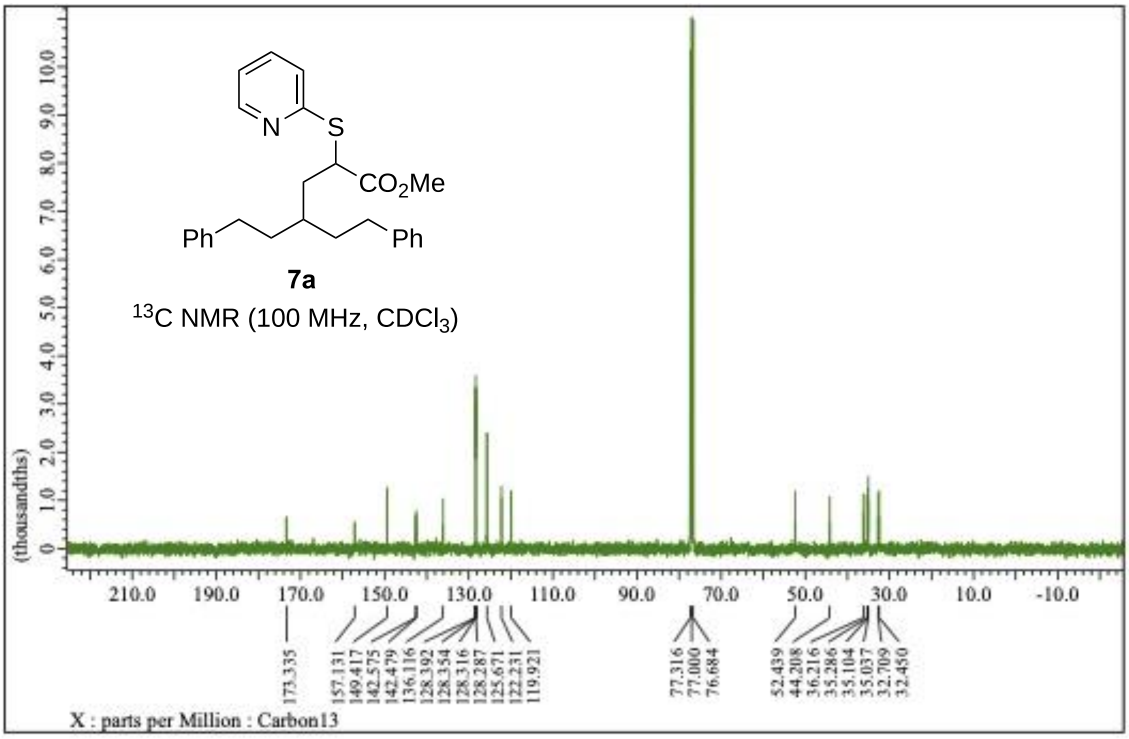

7a
13C NMR (100 MHz, CDCl3)
(thousandths)
X : parts per Million : Carbon13
173.335
157.131 149.417 142.575 142.479 136.116 128.392 128.354 128.316 128.287 125.671 122.231 119.921
77.316 77.000 76.684
52.439 44.208 36.216 35.286 35.104 35.037 32.709 32.450

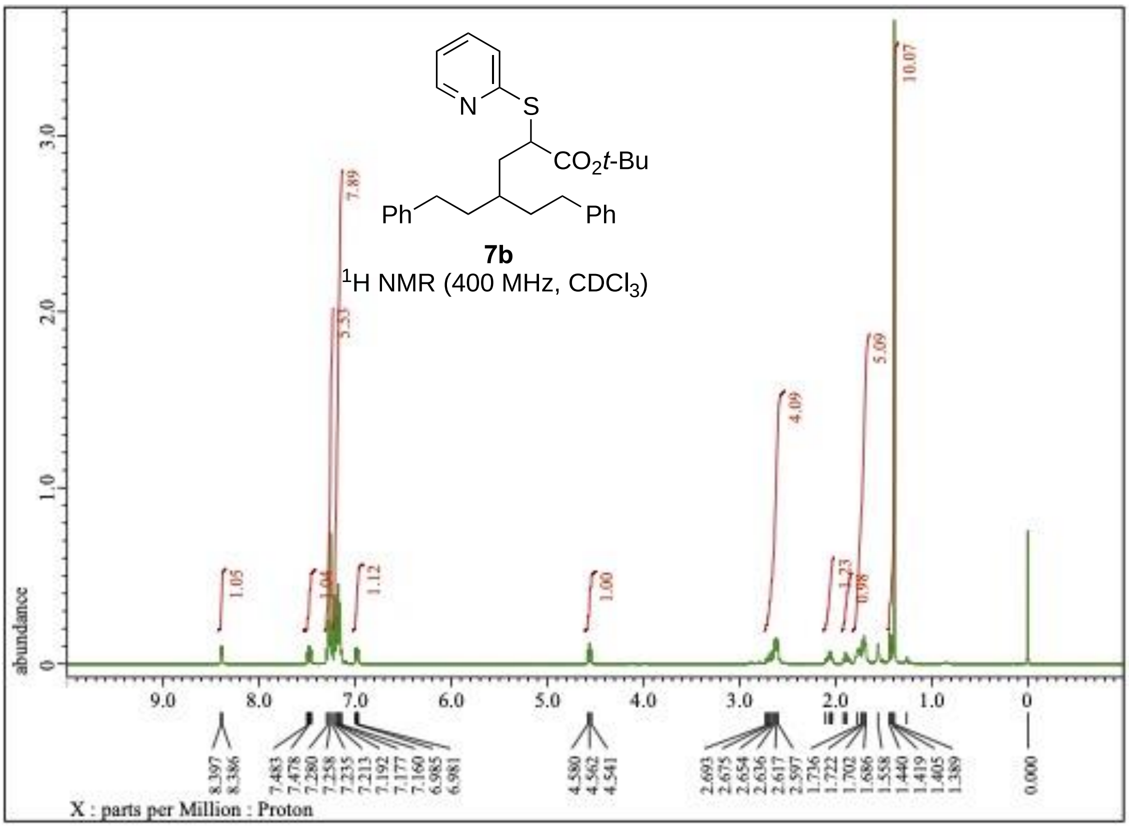
7b
^{1}H NMR (400 MHz, CDCl$_3$)
X : parts per Million : Proton
abundance

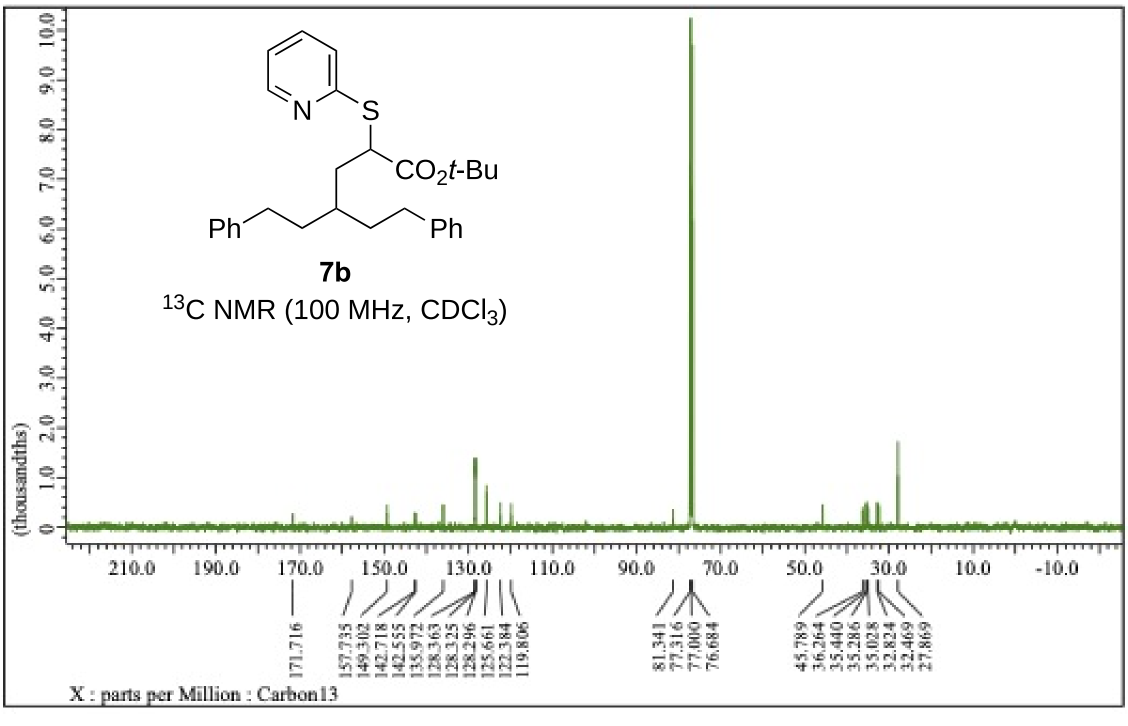
7b
^{13}C NMR (100 MHz, CDCl$_3$)
X : parts per Million : Carbon13
(thousandths)

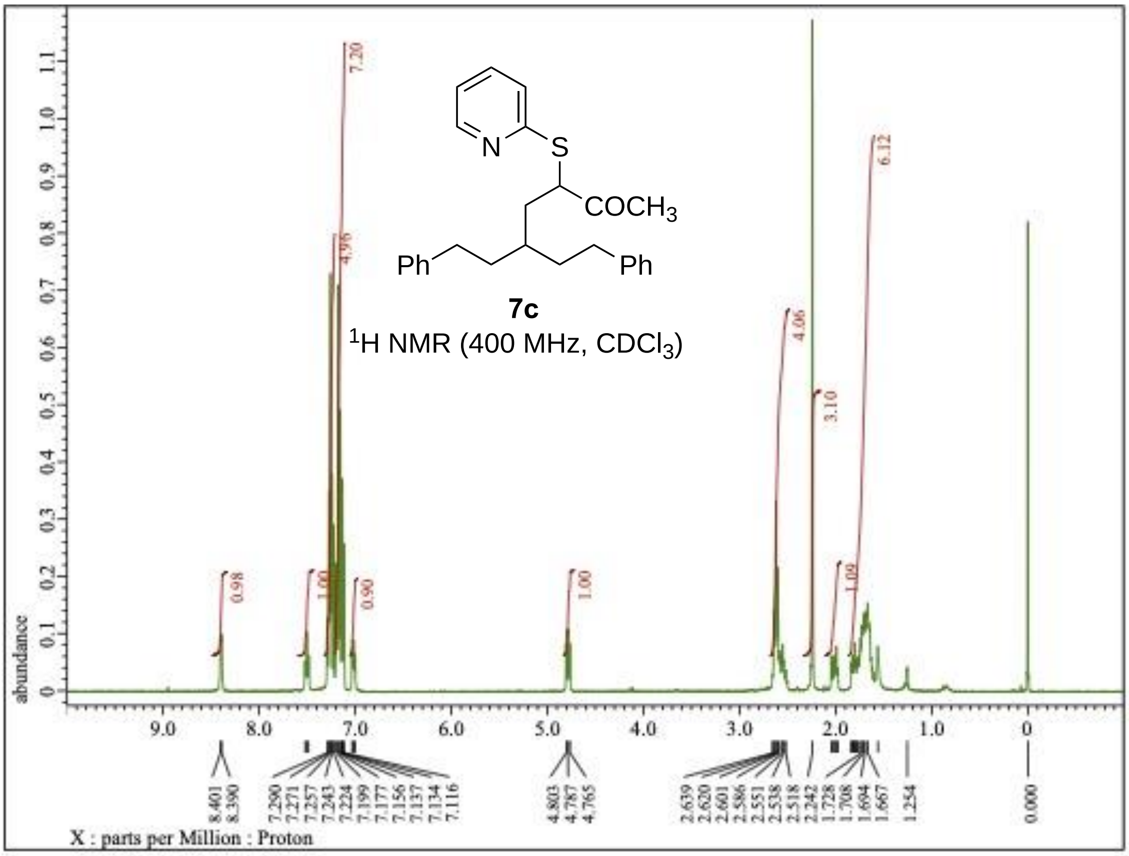
N
S
COCH3
Ph
Ph
7c
1H NMR (400 MHz, CDCl3)
abundance
X : parts per Million : Proton
0.98
1.00
4.96
7.20
0.90
1.00
4.06
3.10
1.09
6.12
8.401
8.390
7.290
7.271
7.257
7.243
7.224
7.199
7.177
7.156
7.137
7.134
7.116
4.803
4.787
4.765
2.639
2.620
2.601
2.586
2.551
2.538
2.518
2.242
1.728
1.708
1.694
1.667
1.254
0.000

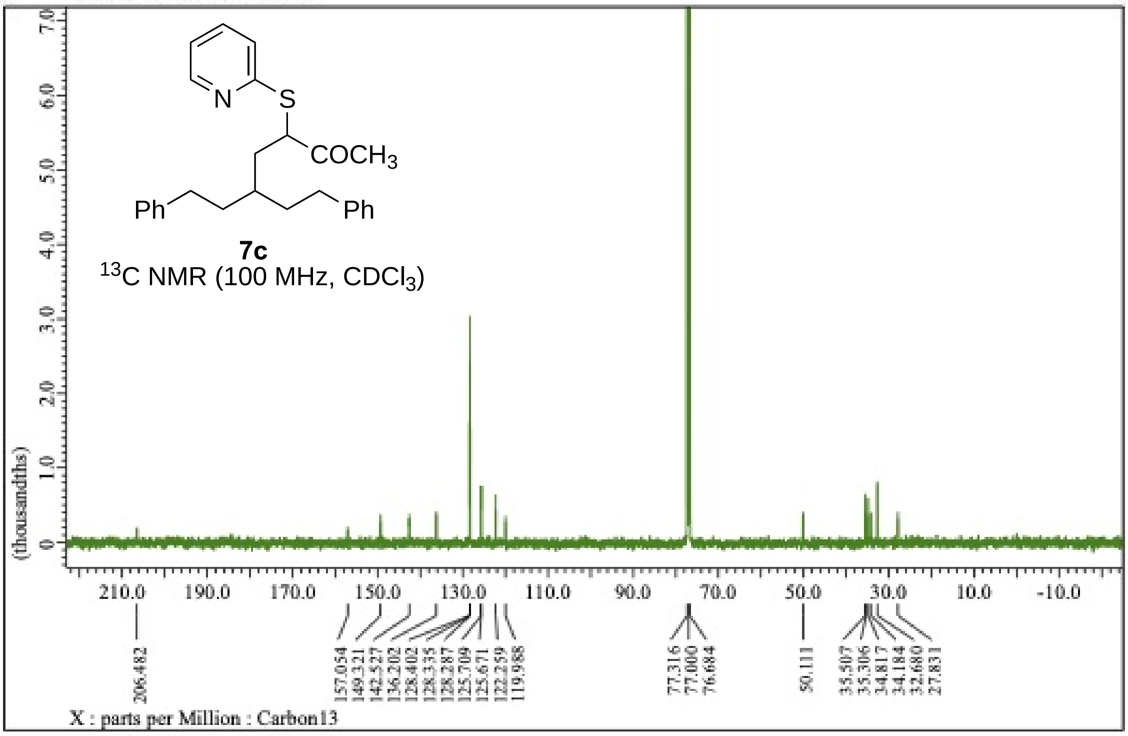
N
S
COCH3
Ph
Ph
7c
13C NMR (100 MHz, CDCl3)
(thousandths)
X : parts per Million : Carbon13
206.482
157.054
149.321
142.527
136.202
128.402
128.335
128.287
125.709
125.671
122.259
119.988
77.316
77.000
76.684
50.111
35.507
35.306
34.817
34.184
32.680
27.831

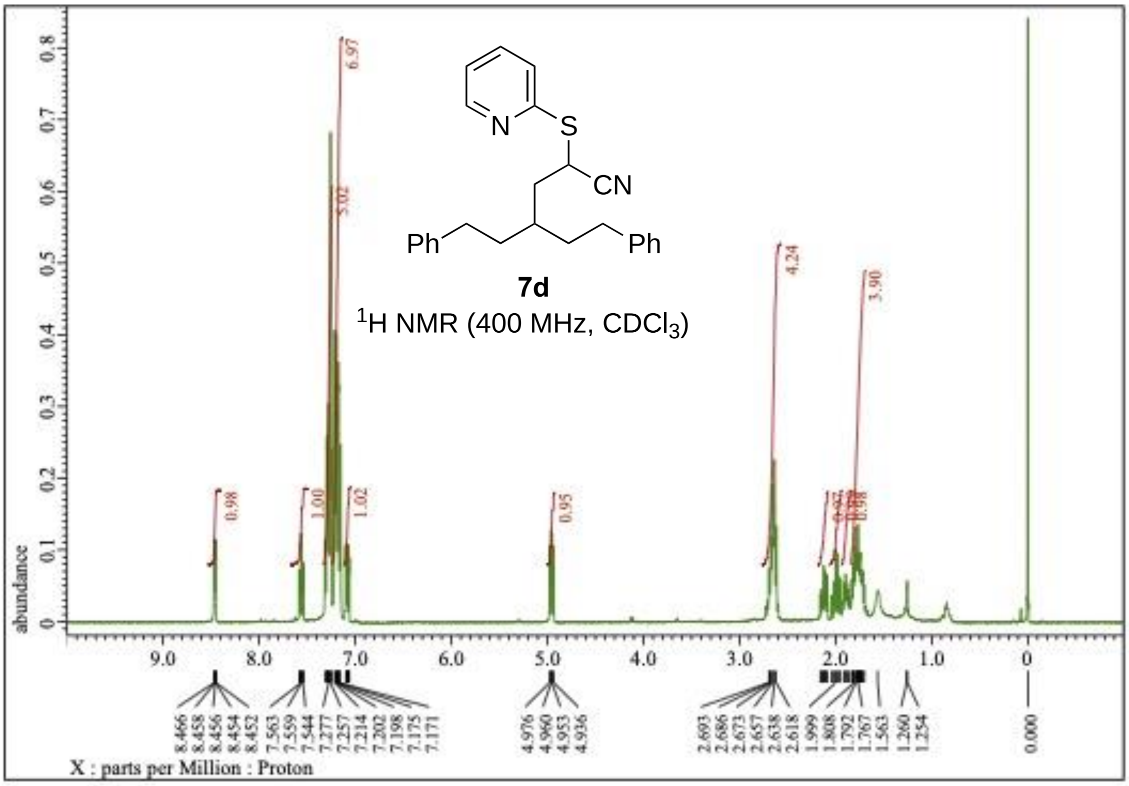
N
S
CN
Ph
Ph
7d
1H NMR (400 MHz, CDCl3)
abundance
X : parts per Million : Proton

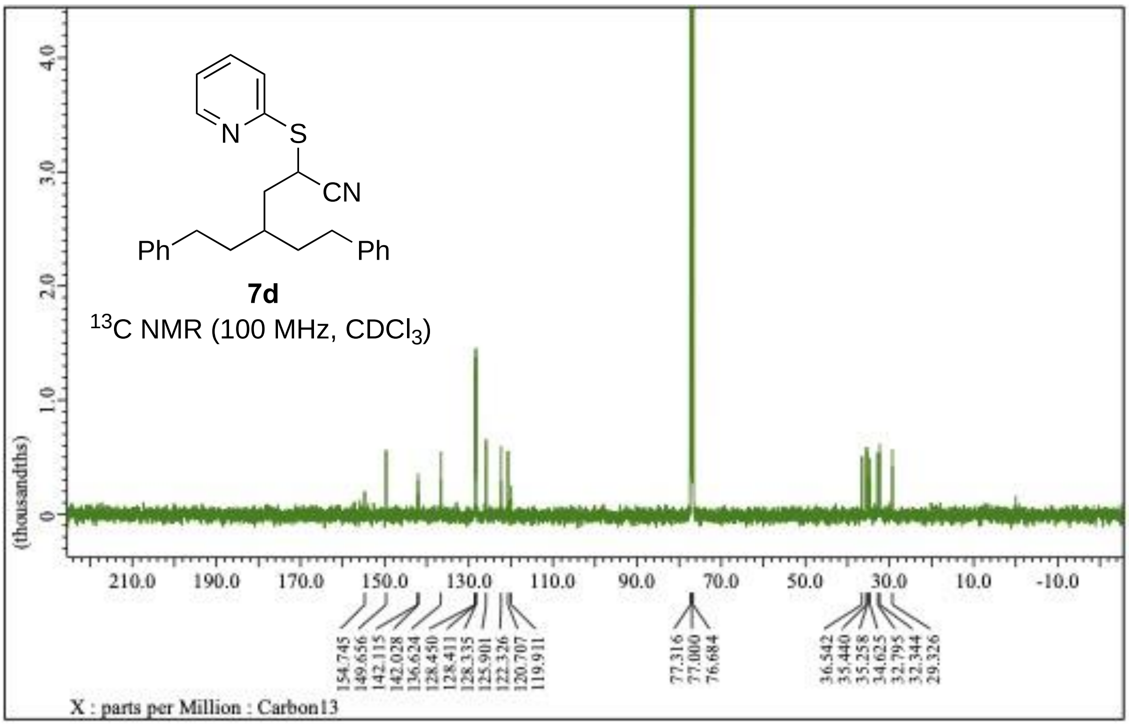
N
S
CN
Ph
Ph
7d
13C NMR (100 MHz, CDCl3)
(thousandths)
X : parts per Million : Carbon13

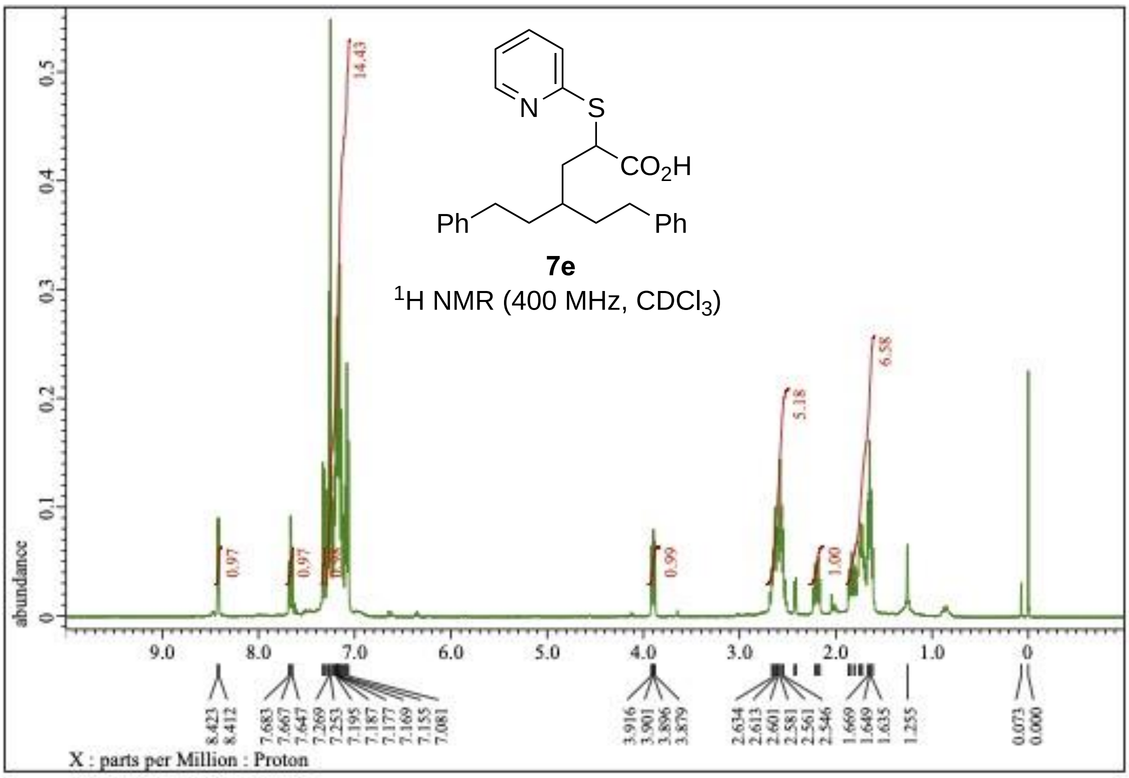

N
S
CO_2H
Ph
Ph
7e
^1H NMR (400 MHz, $CDCl_3$)
abundance
X : parts per Million : Proton

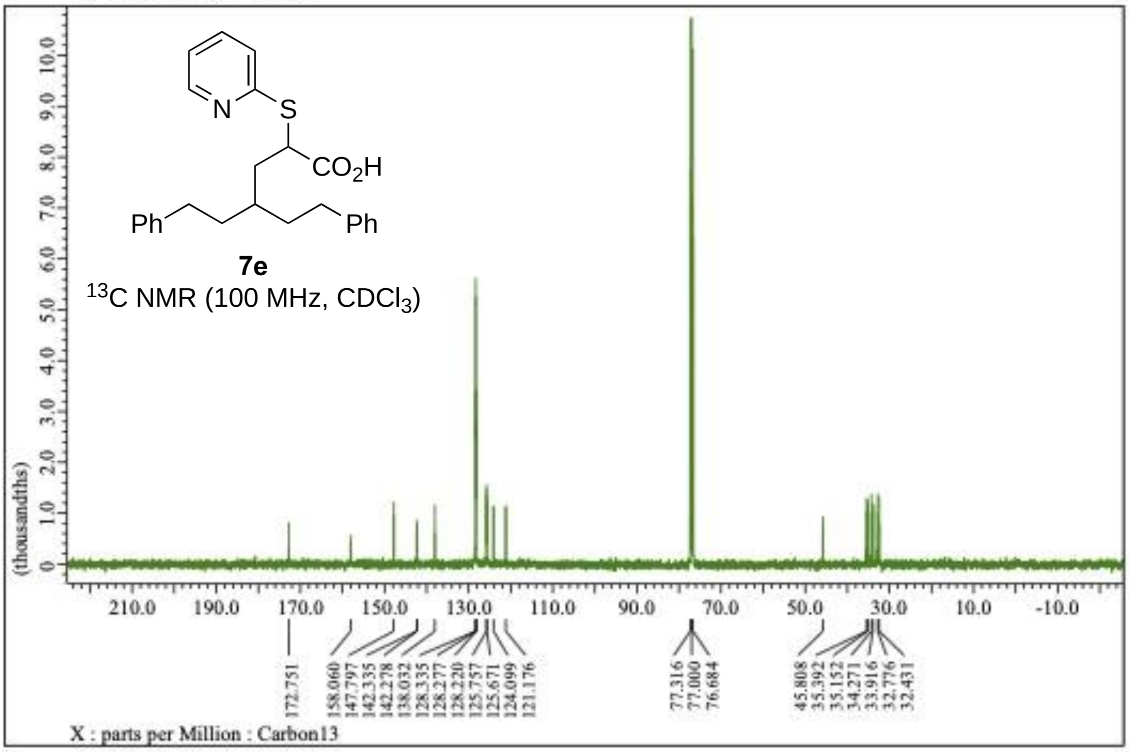

N
S
CO_2H
Ph
Ph
7e
^{13}C NMR (100 MHz, $CDCl_3$)
(thousandths)
X : parts per Million : Carbon13

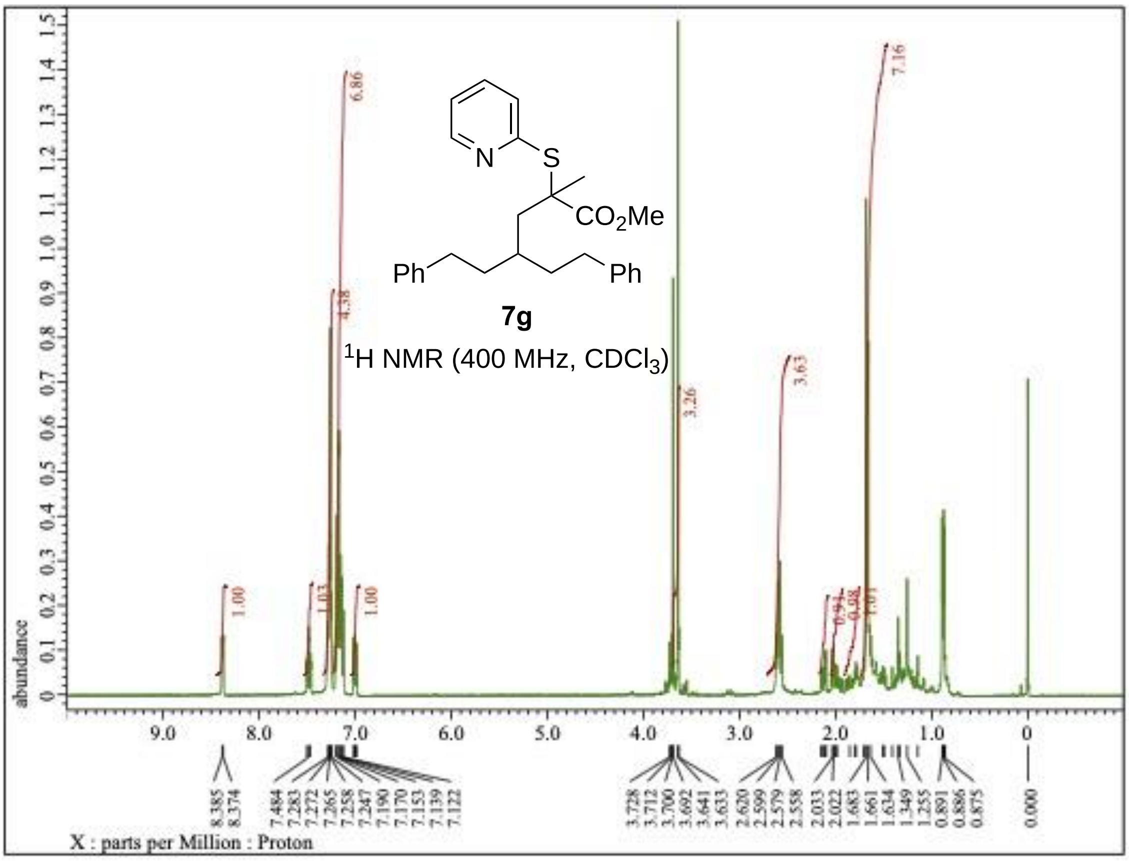
N
S
CO2Me
Ph
Ph
7g
1H NMR (400 MHz, CDCl3)
abundance
X : parts per Million : Proton

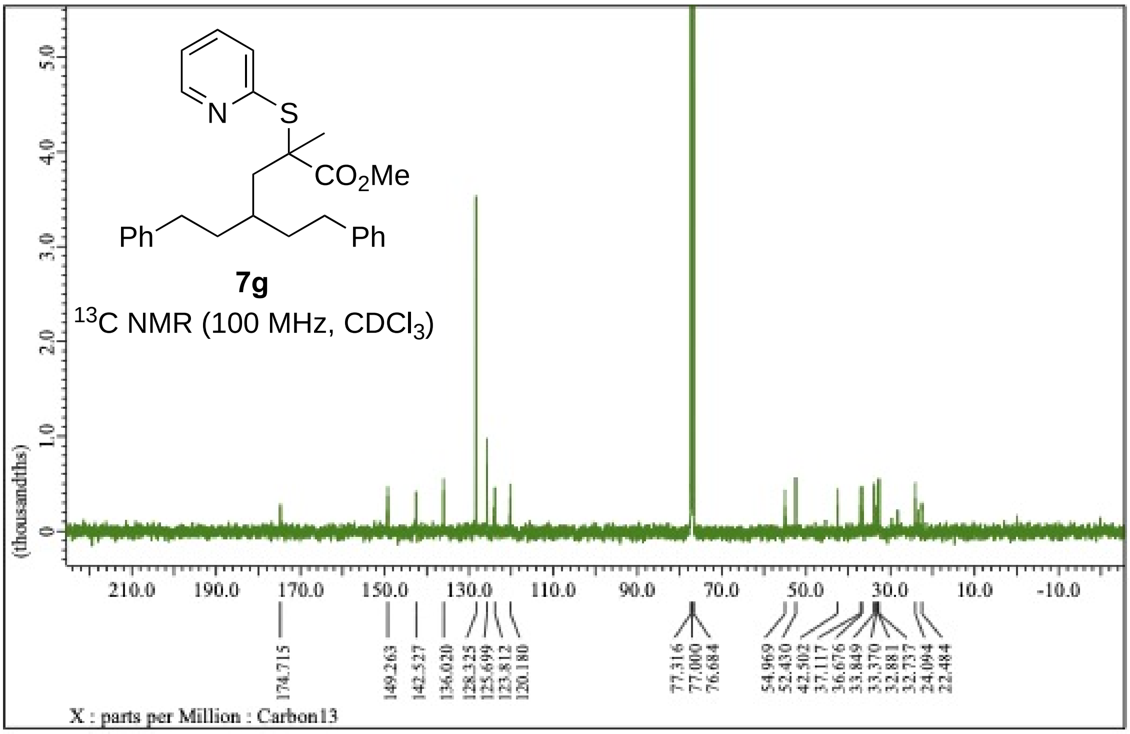
N
S
CO2Me
Ph
Ph
7g
13C NMR (100 MHz, CDCl3)
(thousandths)
X : parts per Million : Carbon13

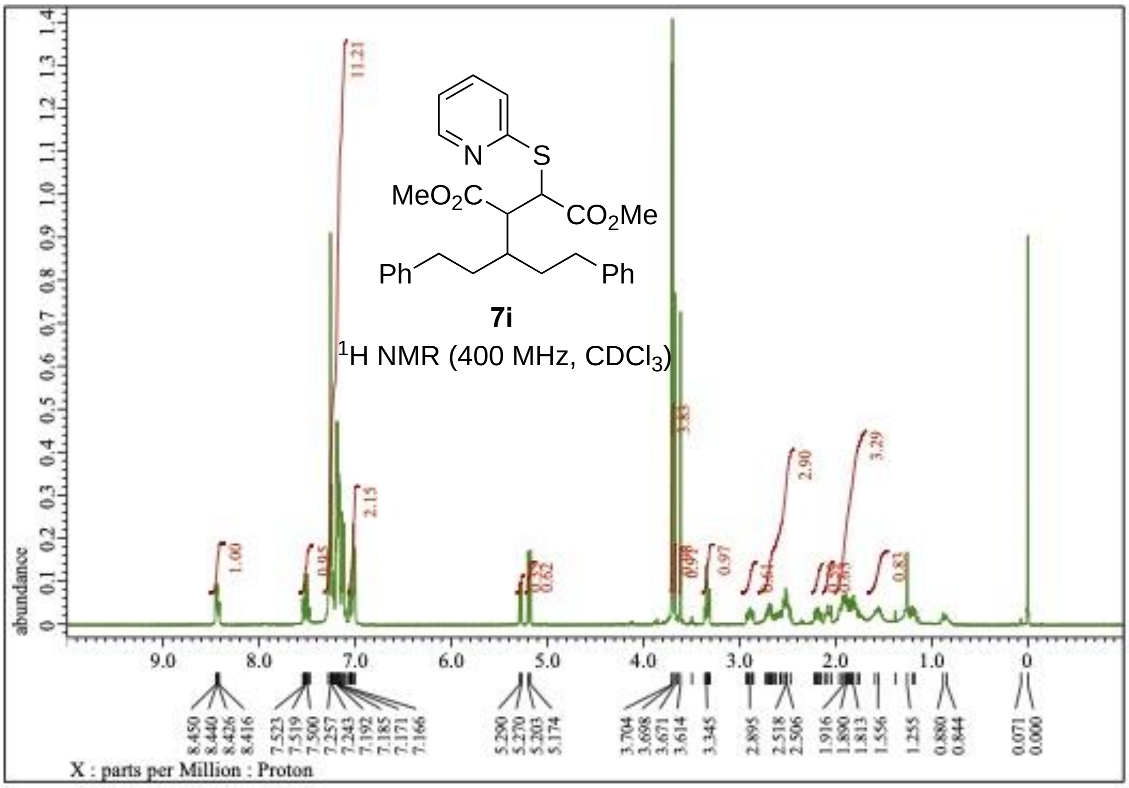
N
S
MeO2C
CO2Me
Ph
Ph
7i
1H NMR (400 MHz, CDCl3)
abundance
X : parts per Million : Proton

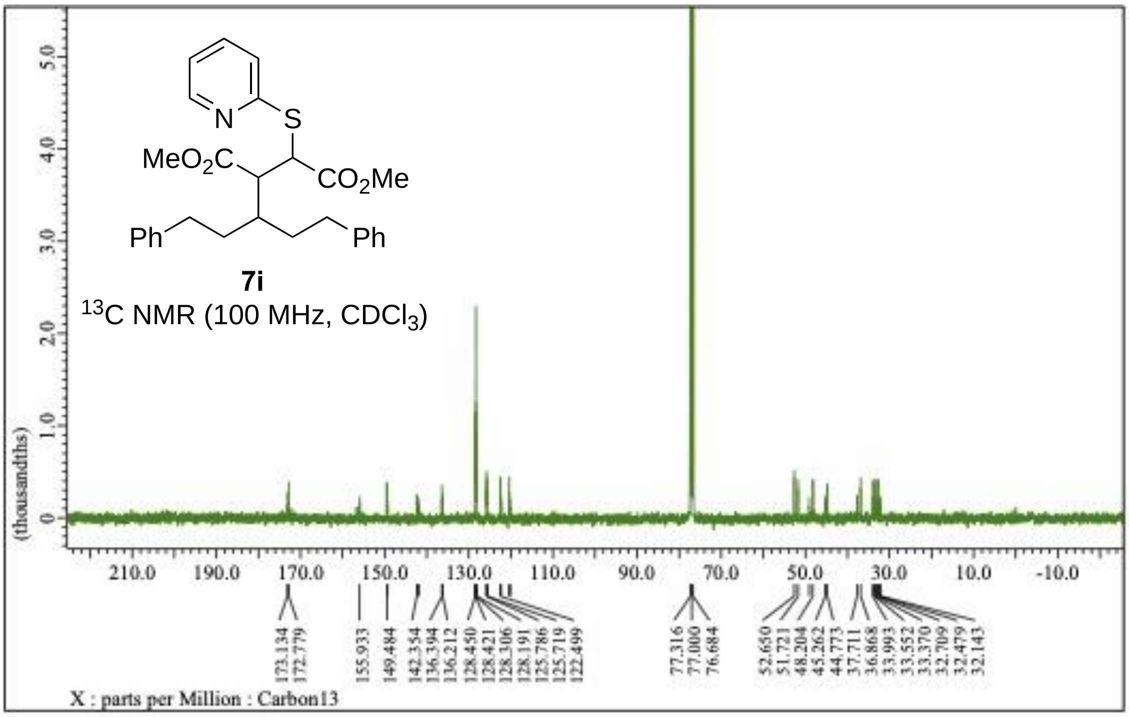
N
S
MeO2C
CO2Me
Ph
Ph
7i
13C NMR (100 MHz, CDCl3)
(thousandths)
X : parts per Million : Carbon13

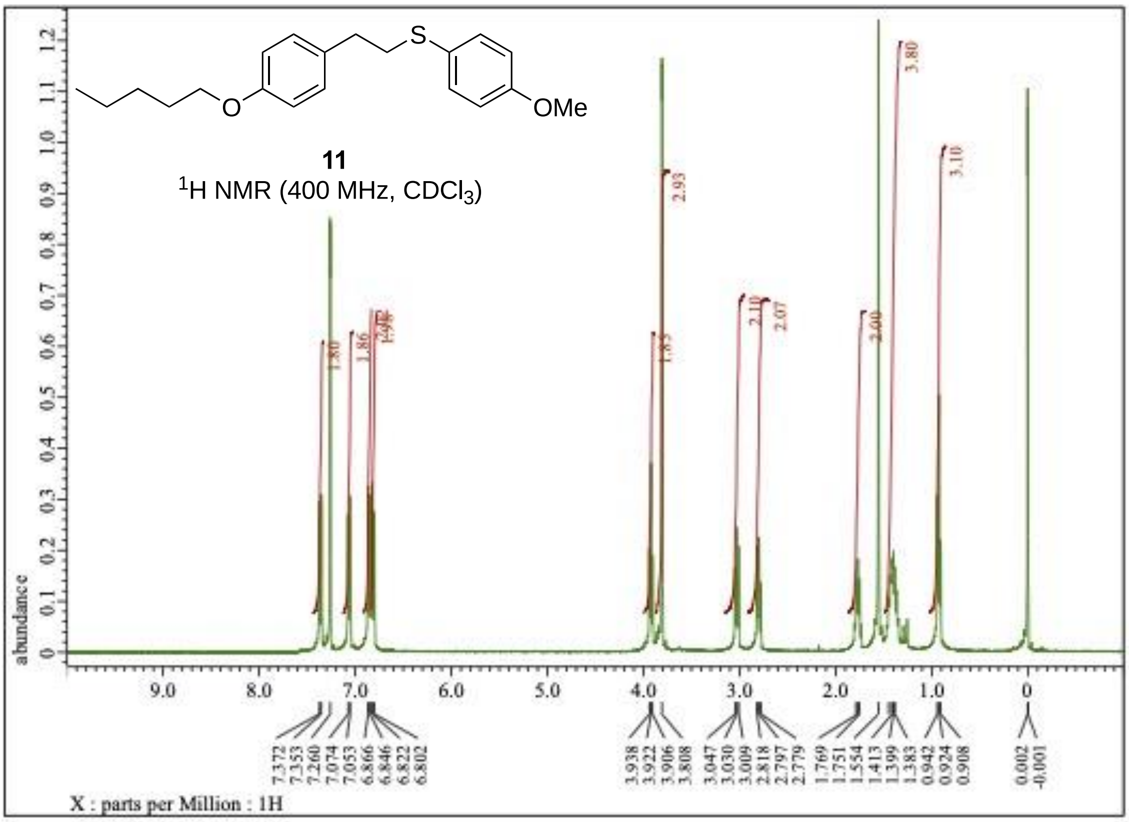

S
O
OMe
11
¹H NMR (400 MHz, CDCl3)
abundance
X : parts per Million : 1H

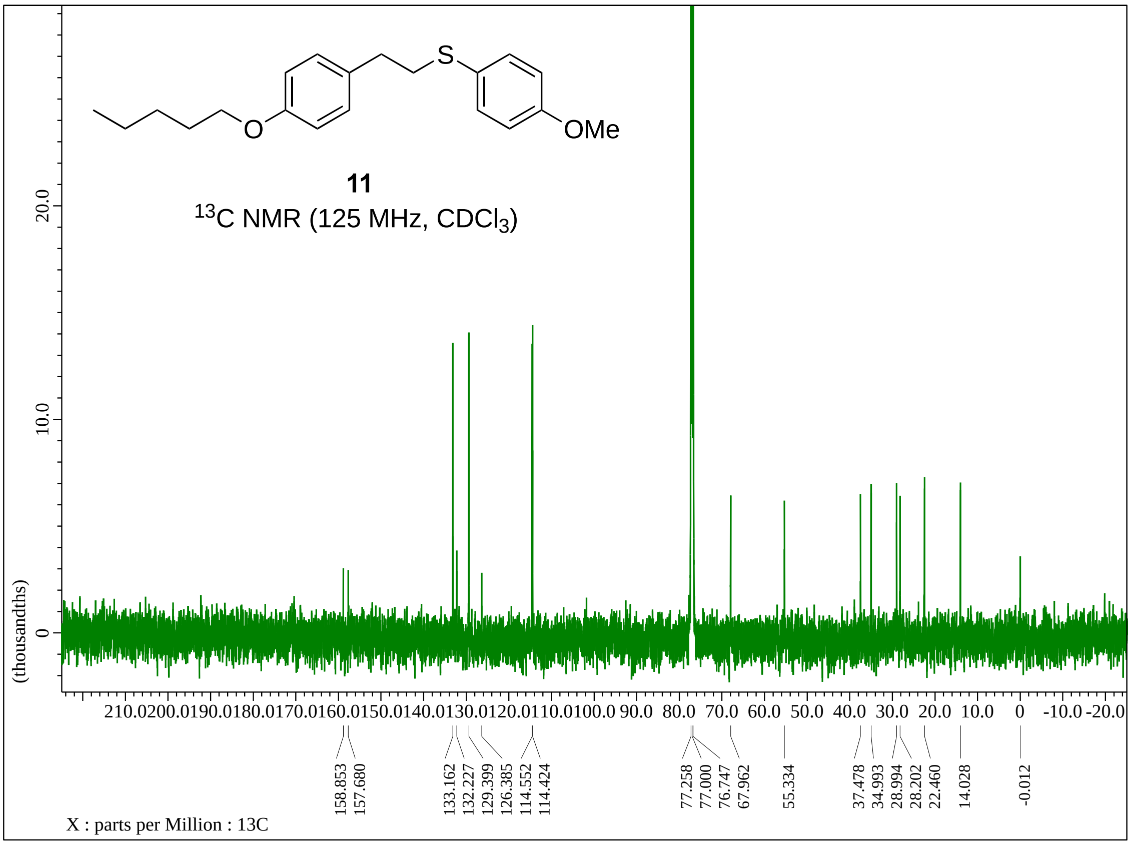

S
O
OMe
11
¹³C NMR (125 MHz, CDCl3)
(thousandths)
X : parts per Million : 13C

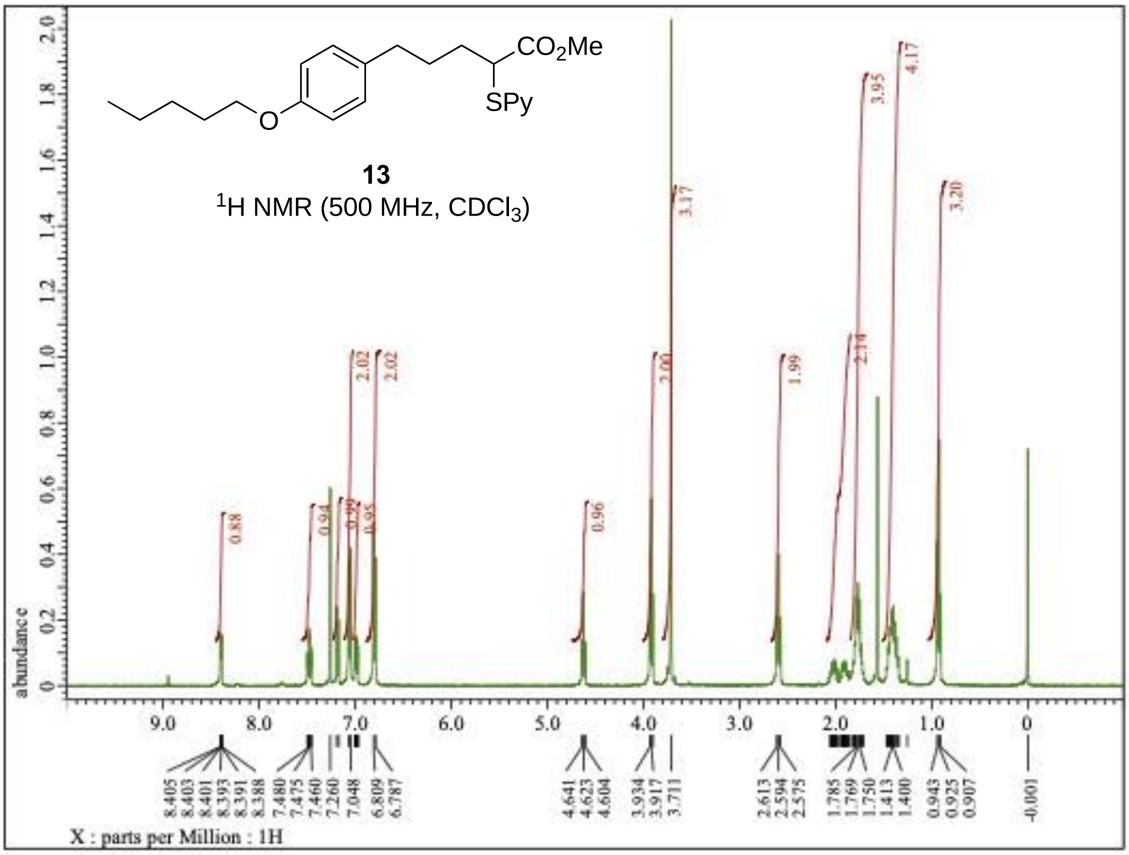

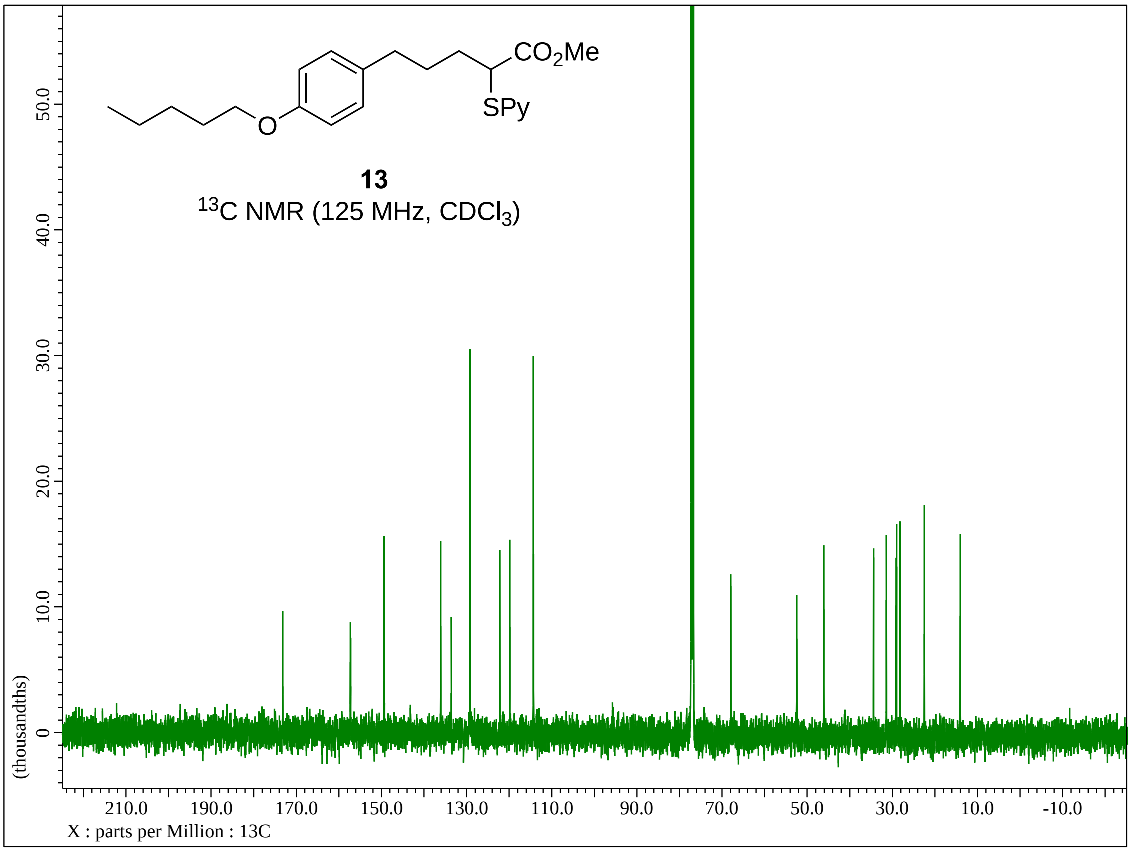

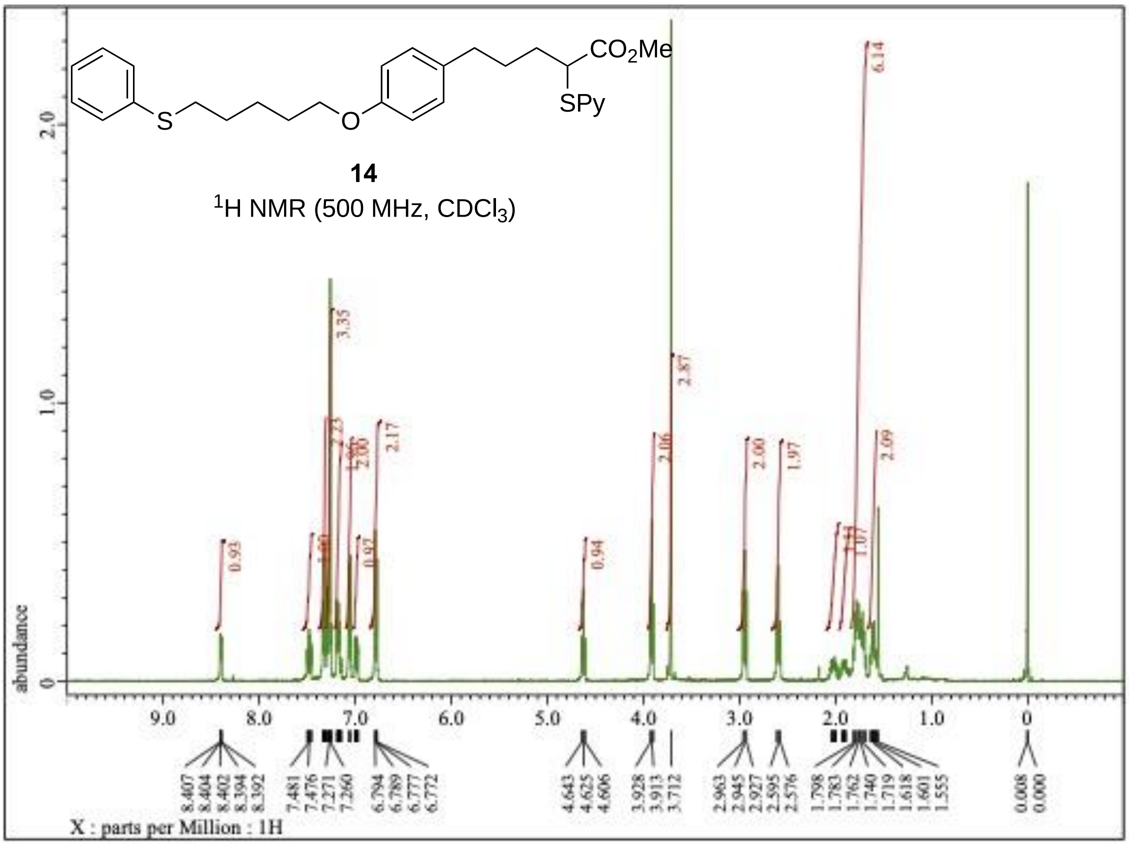

CO2Me
S
O
SPy
14
1H NMR (500 MHz, CDCl3)
abundance
X : parts per Million : 1H

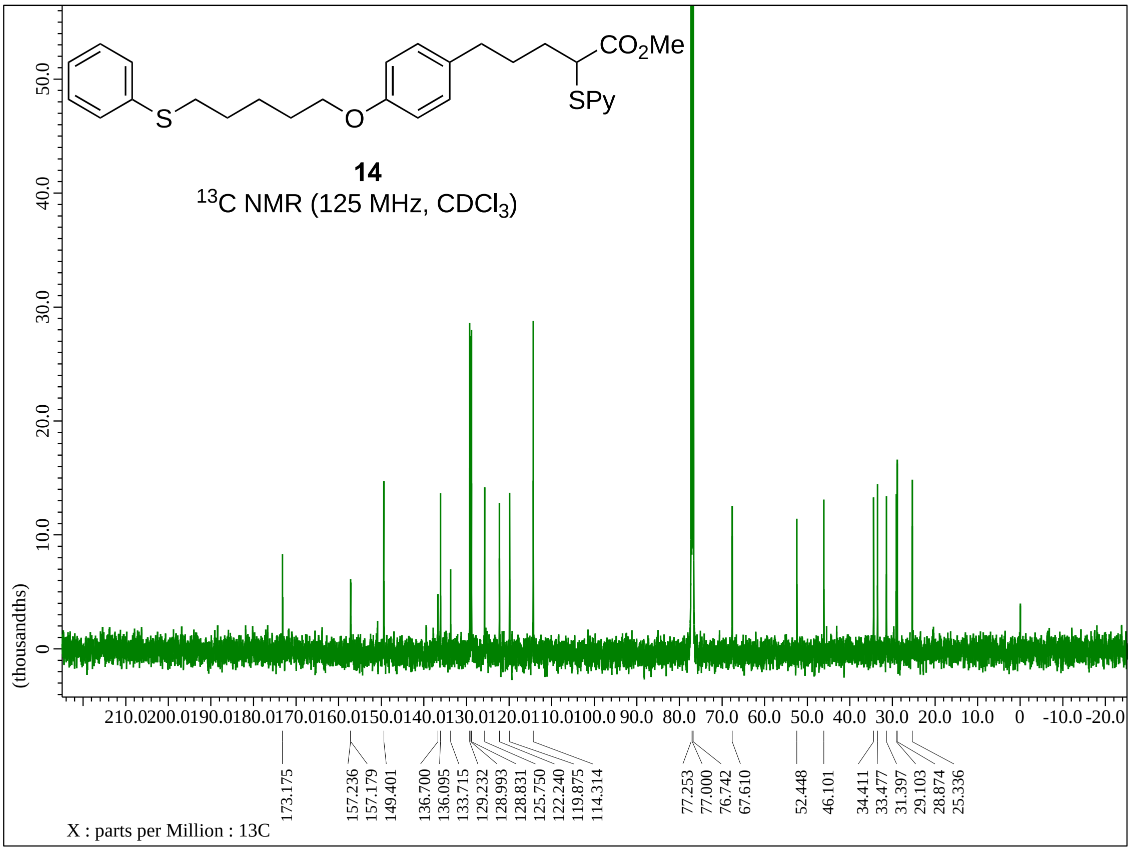

CO2Me
S
O
SPy
14
13C NMR (125 MHz, CDCl3)
(thousandths)
X : parts per Million : 13C
173.175
157.236
157.179
149.401
136.700
136.095
133.715
129.232
128.993
128.831
125.750
122.240
119.875
114.314
77.253
77.000
76.742
67.610
52.448
46.101
34.411
33.477
31.397
29.103
28.874
25.336

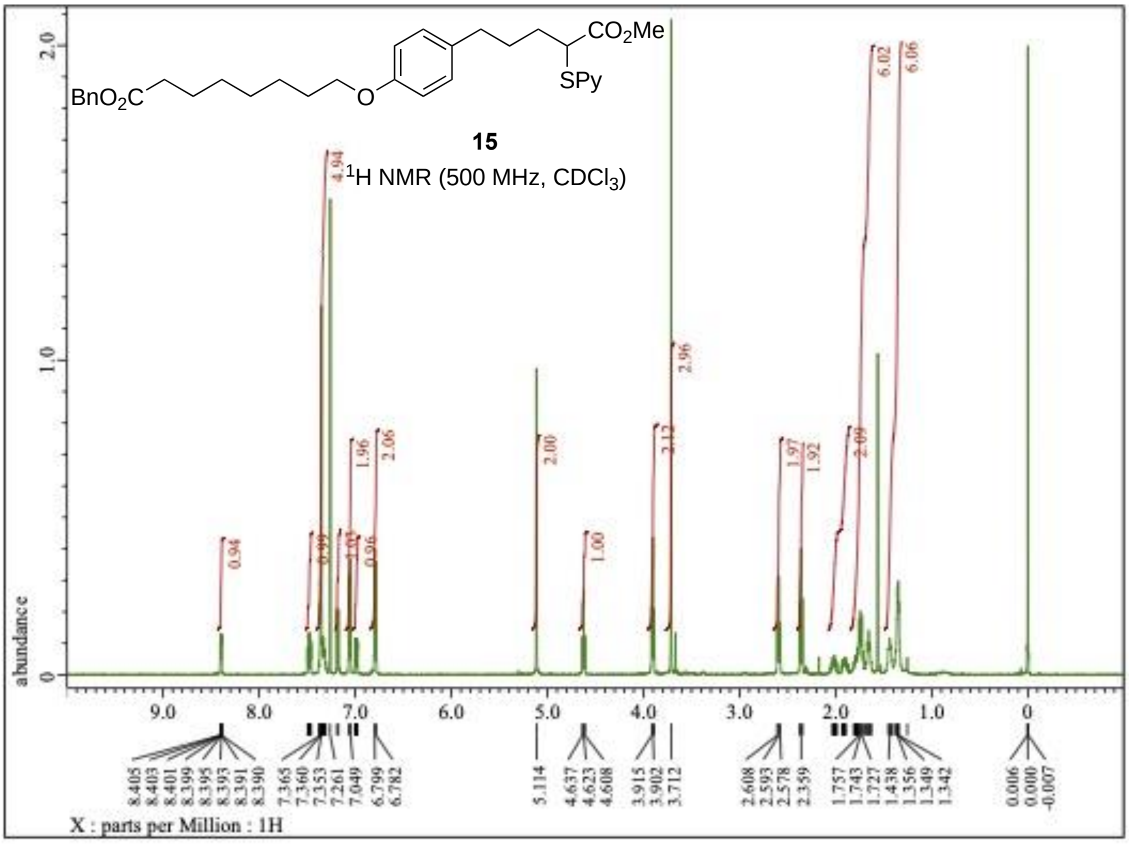

15
¹H NMR (500 MHz, CDCl3)
BnO2C
CO2Me
SPy
abundance
X : parts per Million : 1H

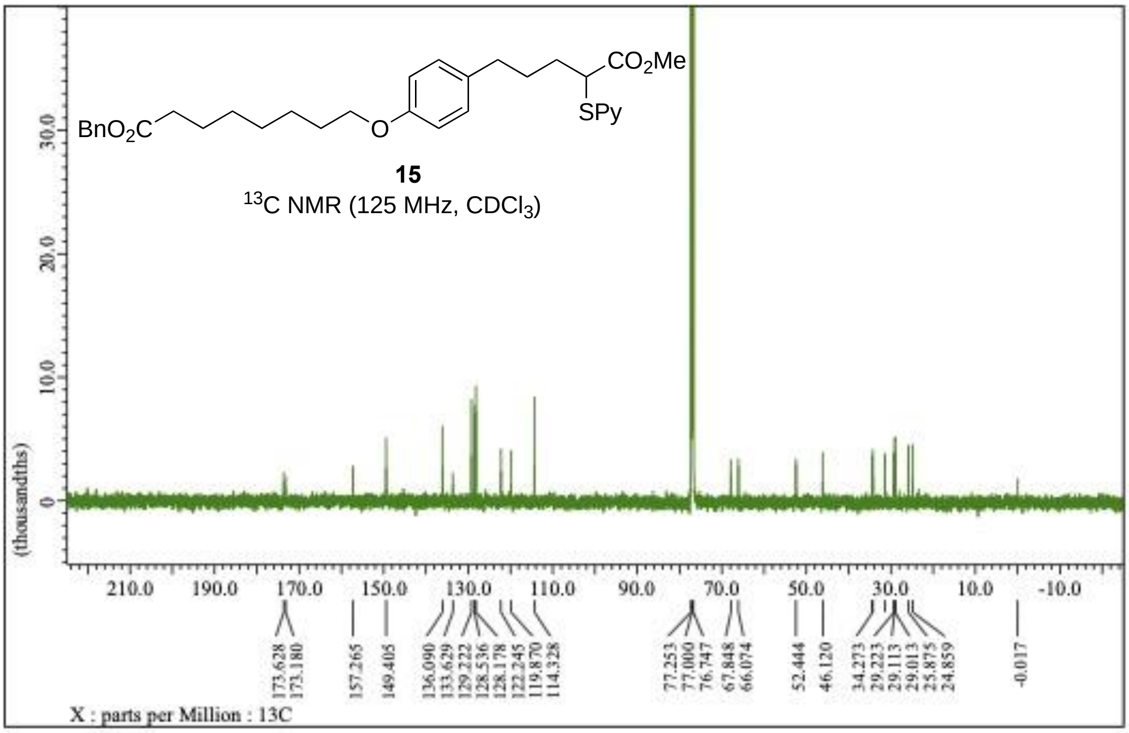

15
¹³C NMR (125 MHz, CDCl3)
BnO2C
CO2Me
SPy
(thousandths)
X : parts per Million : 13C

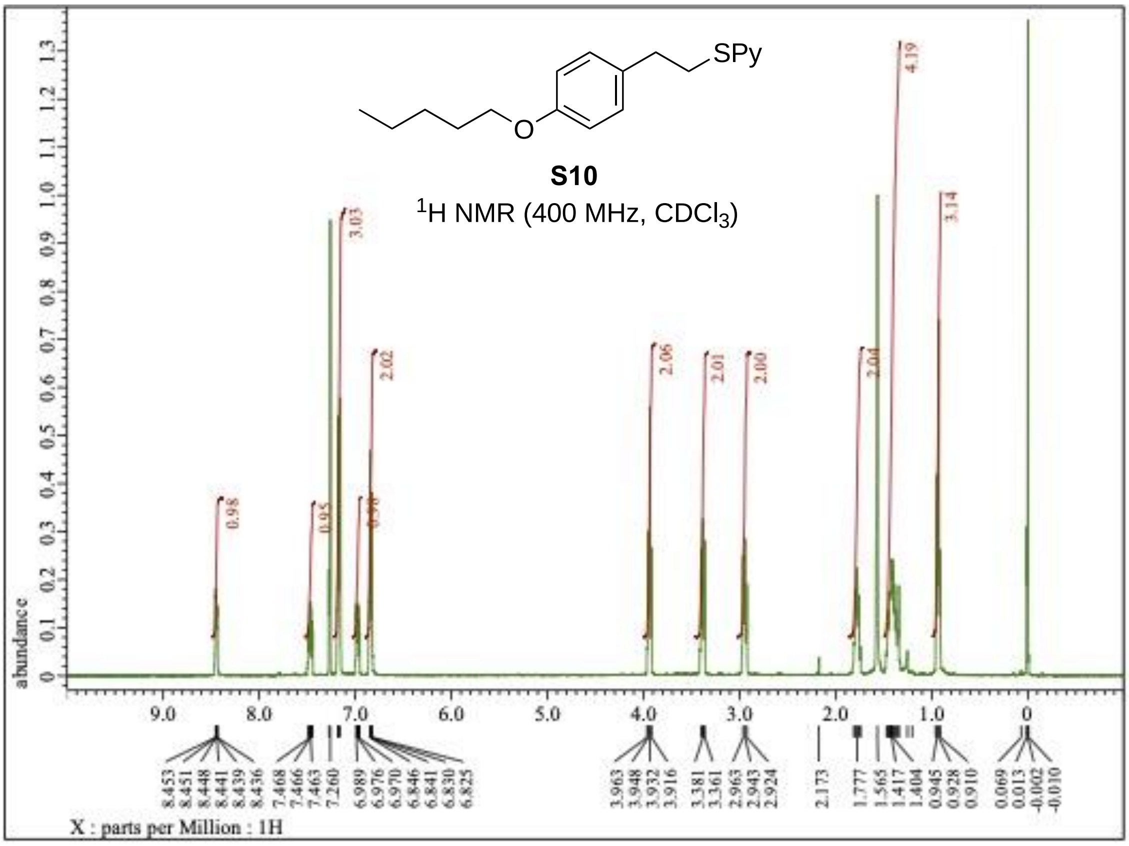
SPy
O
S10
1H NMR (400 MHz, CDCl3)
X : parts per Million : 1H
abundance

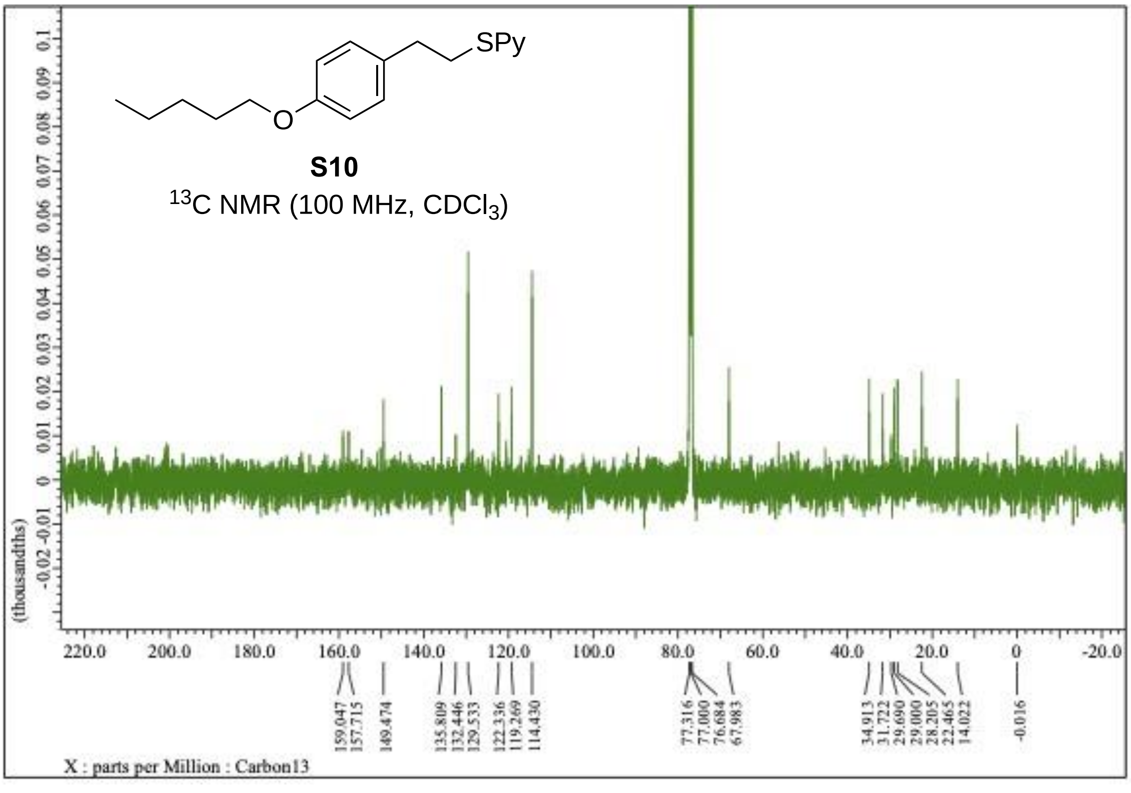
SPy
O
S10
13C NMR (100 MHz, CDCl3)
X : parts per Million : Carbon13
(thousandths)

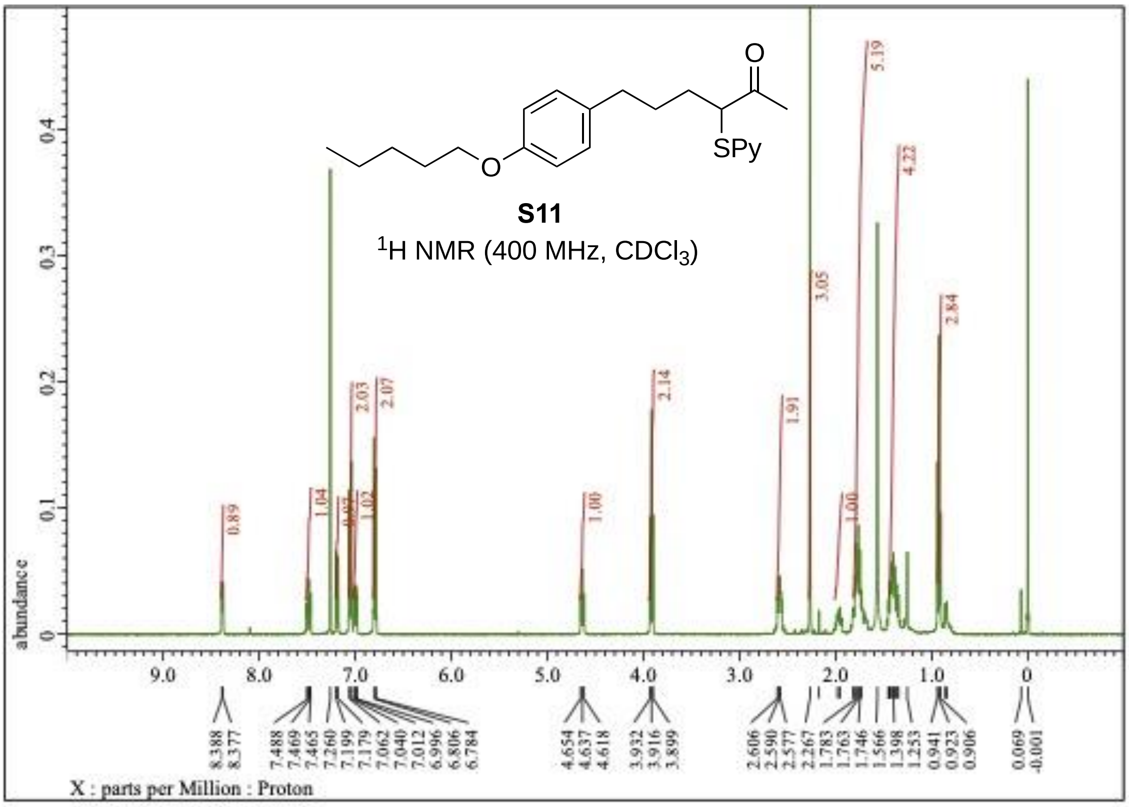
O
SPy
O
S11
^1H NMR (400 MHz, $CDCl_3$)
X : parts per Million : Proton
abundance

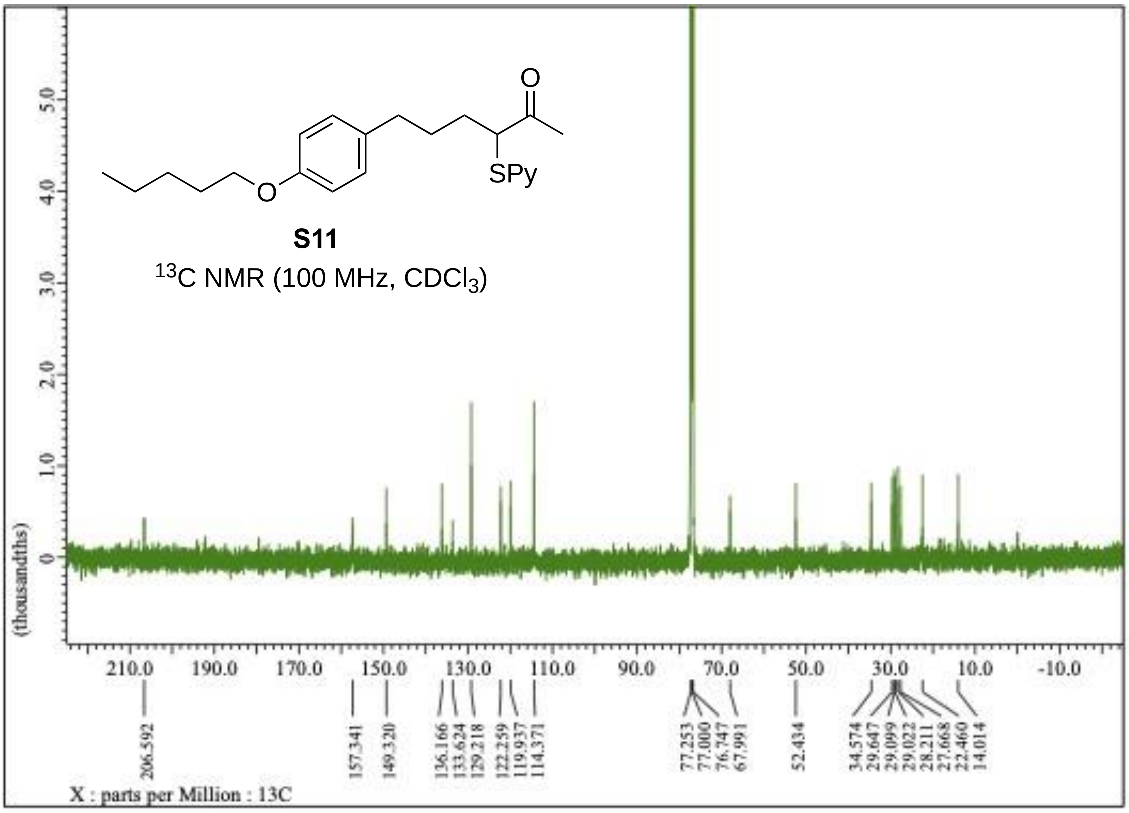
O
SPy
O
S11
^{13}C NMR (100 MHz, $CDCl_3$)
X : parts per Million : 13C
(thousandths)

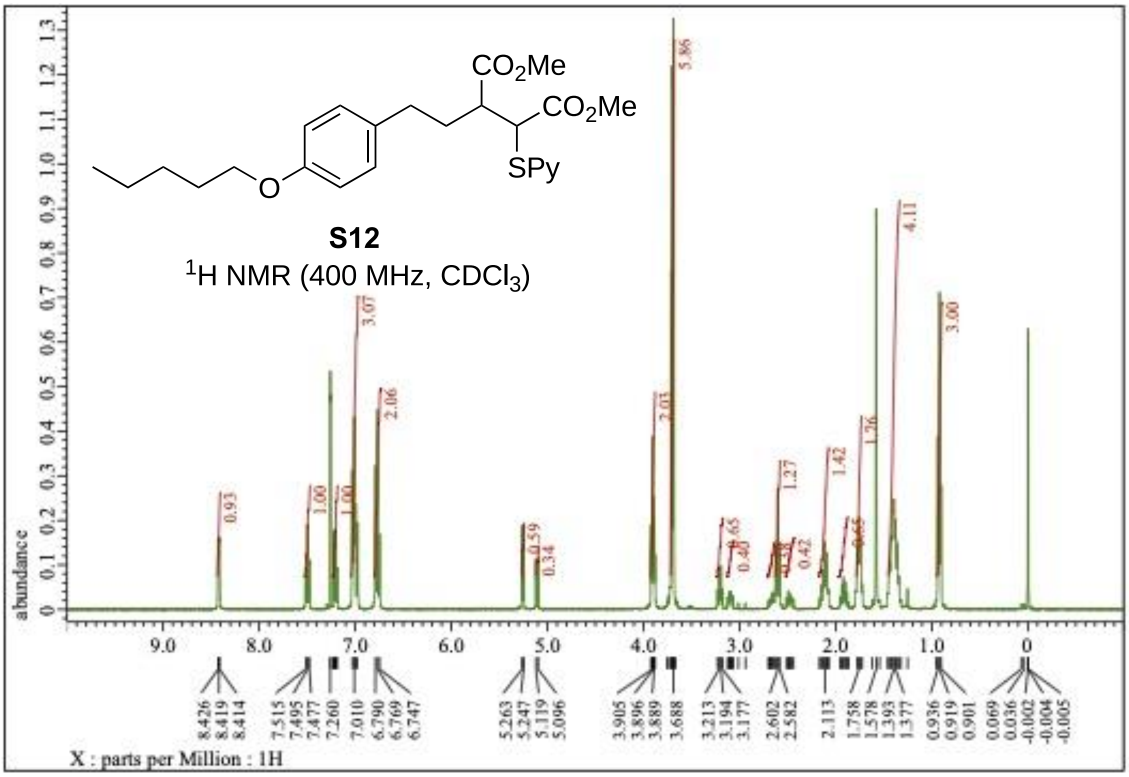
CO2Me
CO2Me
SPy
O
S12
1H NMR (400 MHz, CDCl3)
abundance
X : parts per Million : 1H

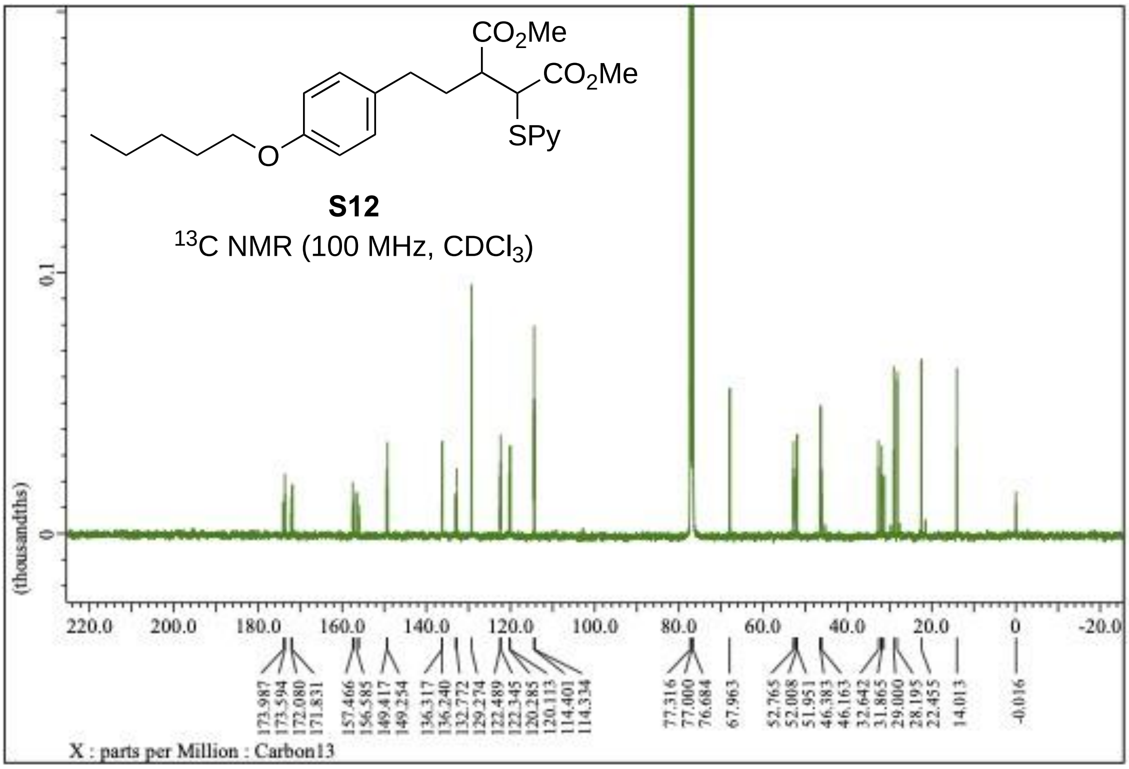
CO2Me
CO2Me
SPy
O
S12
13C NMR (100 MHz, CDCl3)
(thousandths)
X : parts per Million : Carbon13

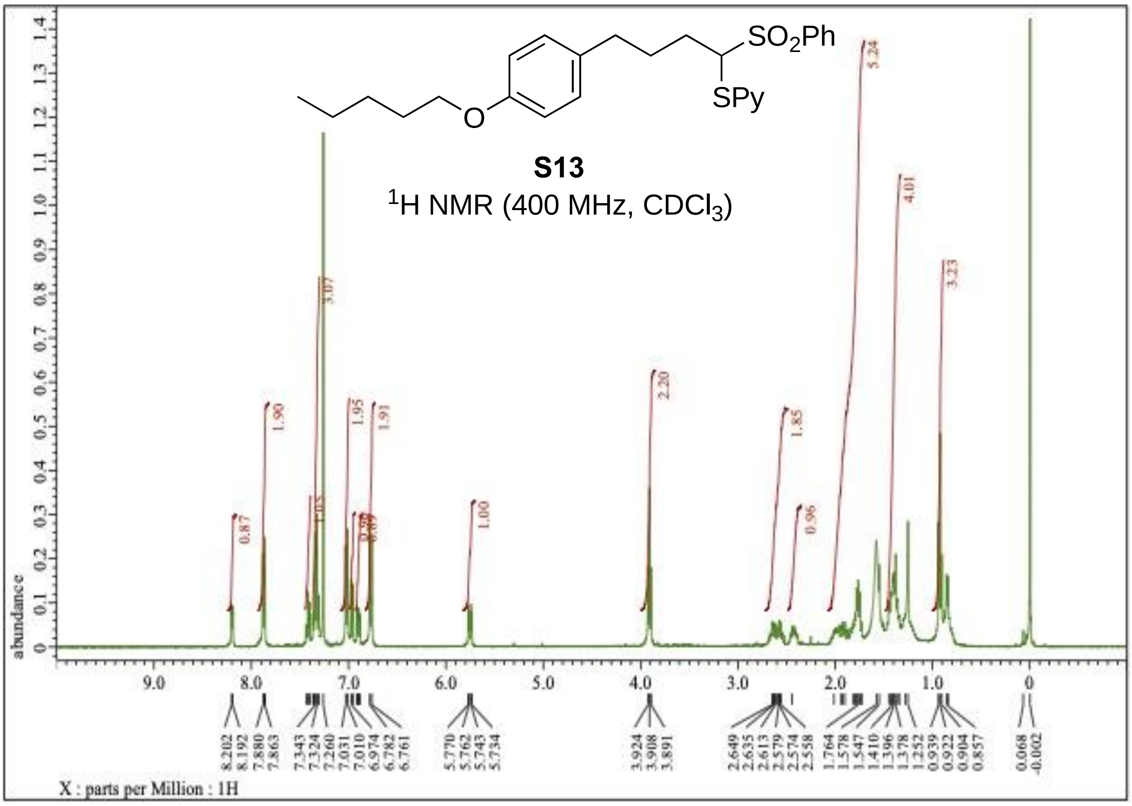
SO2Ph
SPy
O
S13
1H NMR (400 MHz, CDCl3)
abundance
X : parts per Million : 1H

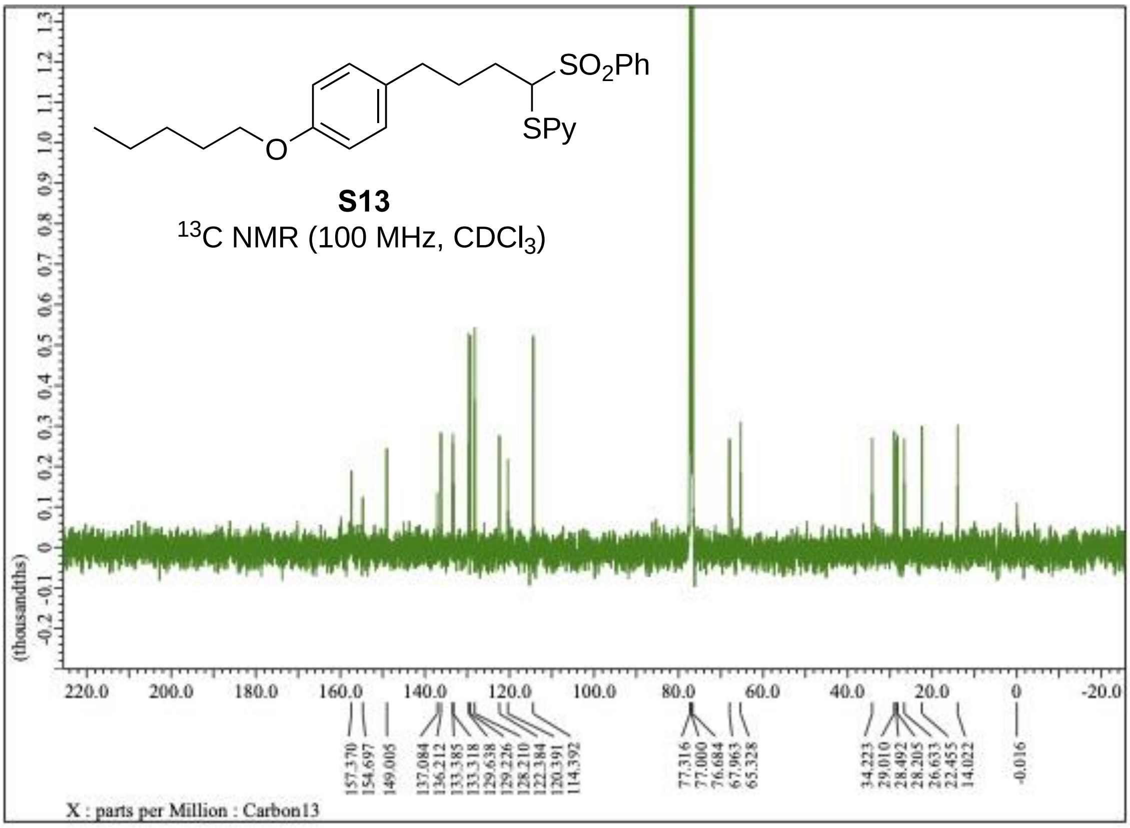
SO2Ph
SPy
O
S13
13C NMR (100 MHz, CDCl3)
(thousandths)
X : parts per Million : Carbon13

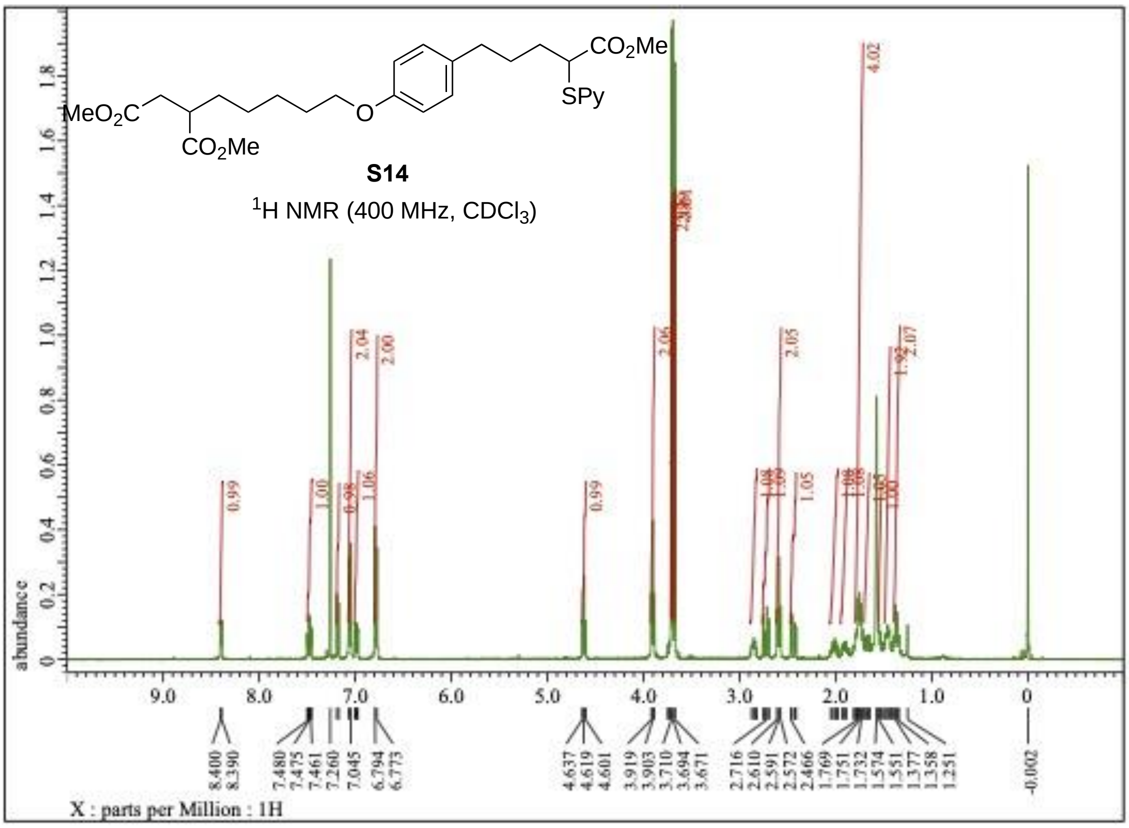

CO2Me
SPy
MeO2C
CO2Me
S14
1H NMR (400 MHz, CDCl3)
X : parts per Million : 1H
abundance

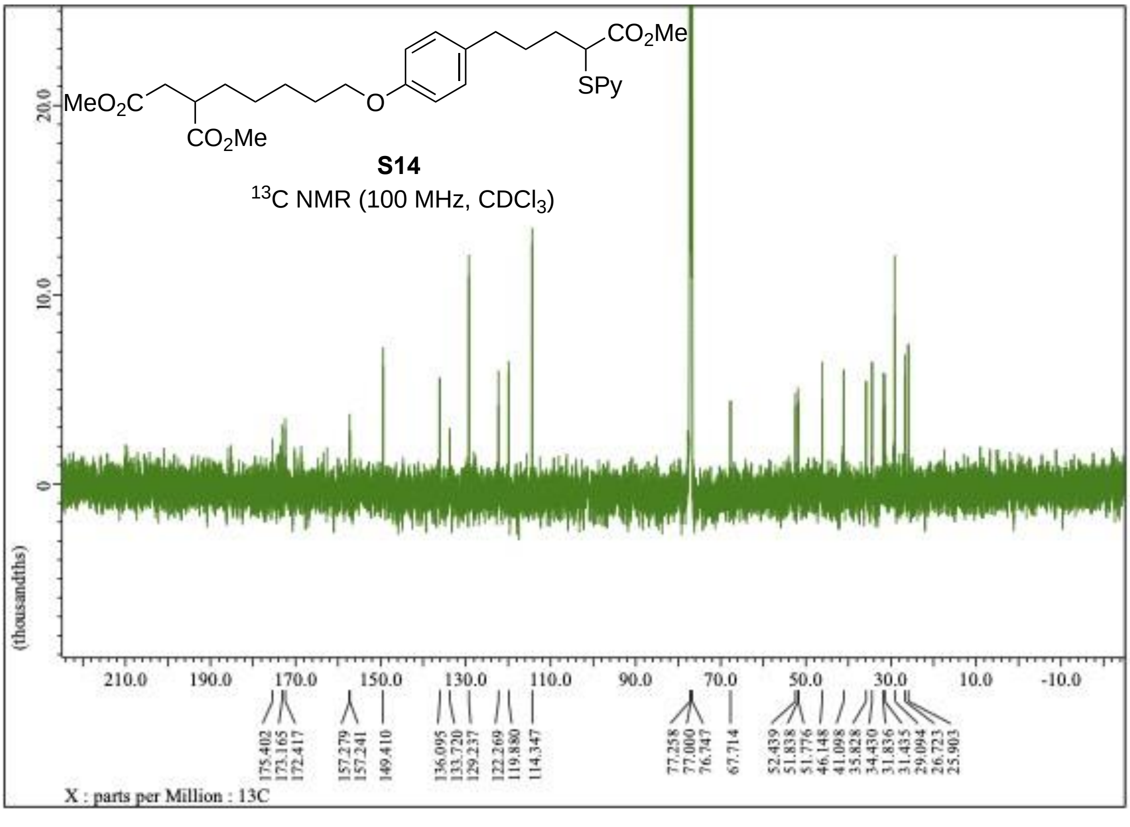

CO2Me
SPy
MeO2C
CO2Me
S14
13C NMR (100 MHz, CDCl3)
X : parts per Million : 13C
(thousandths)

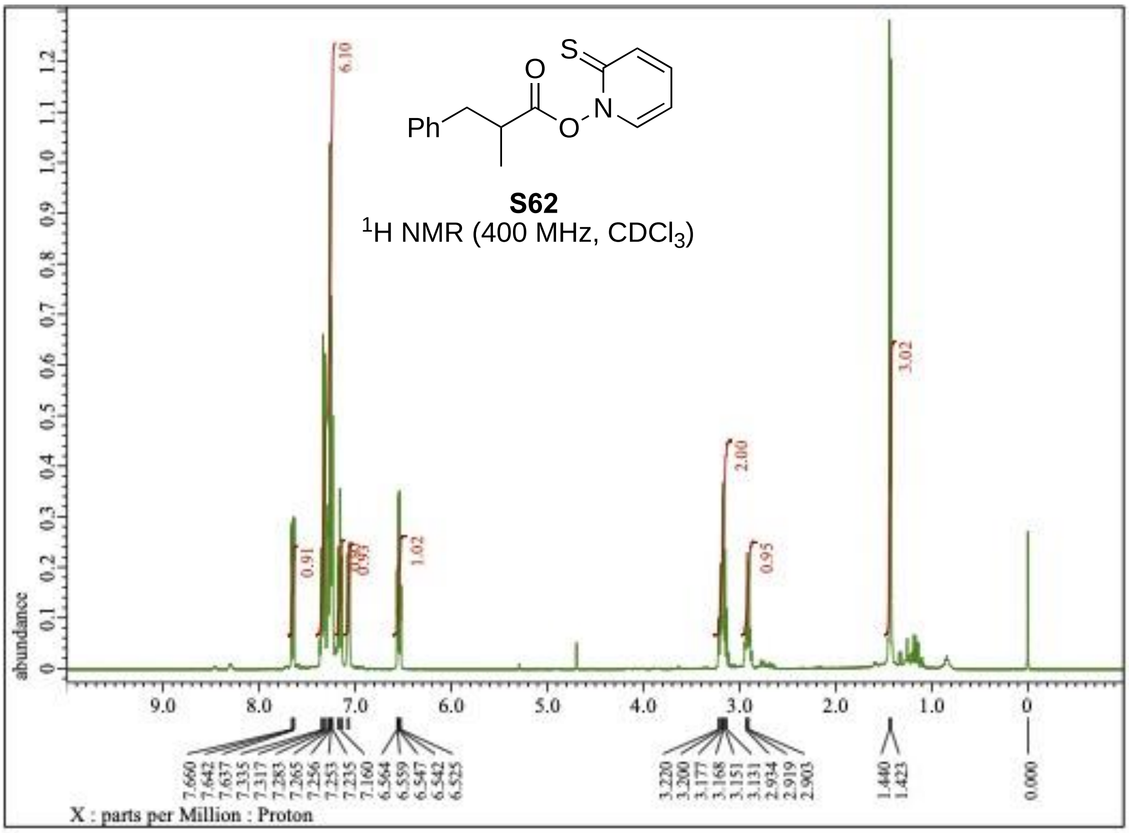
Ph
O
O
N
S
S62
^{1}H NMR (400 MHz, CDCl$_3$)
abundance
X : parts per Million : Proton

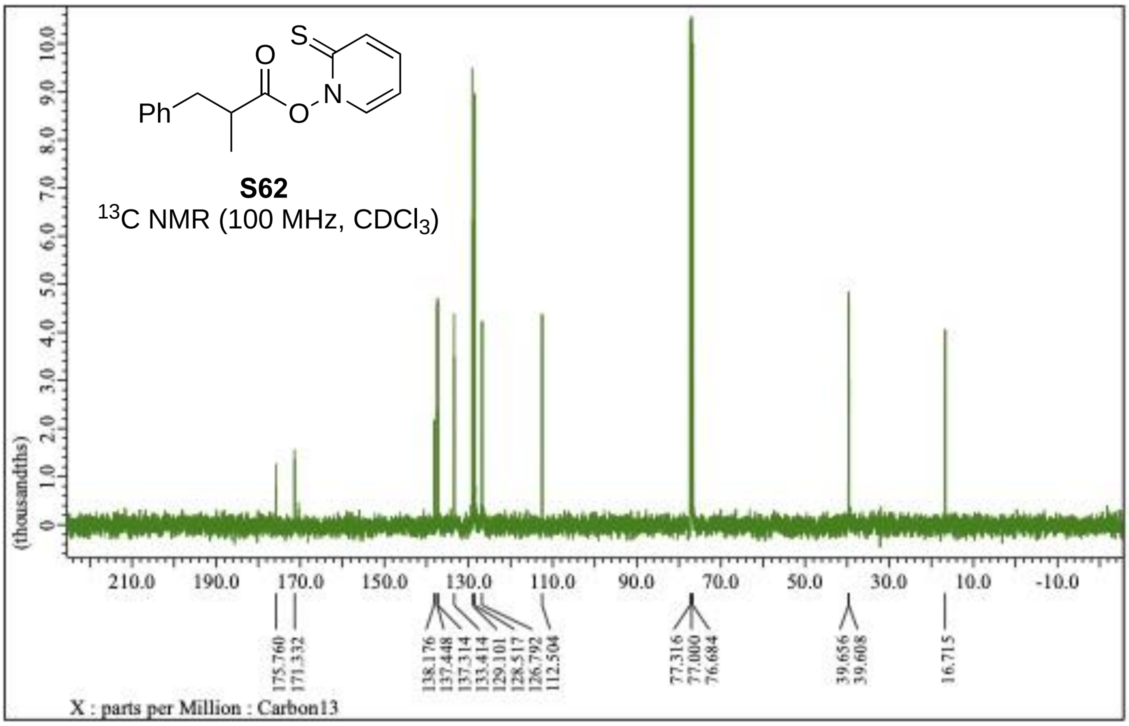
Ph
O
O
N
S
S62
^{13}C NMR (100 MHz, CDCl$_3$)
(thousandths)
X : parts per Million : Carbon13

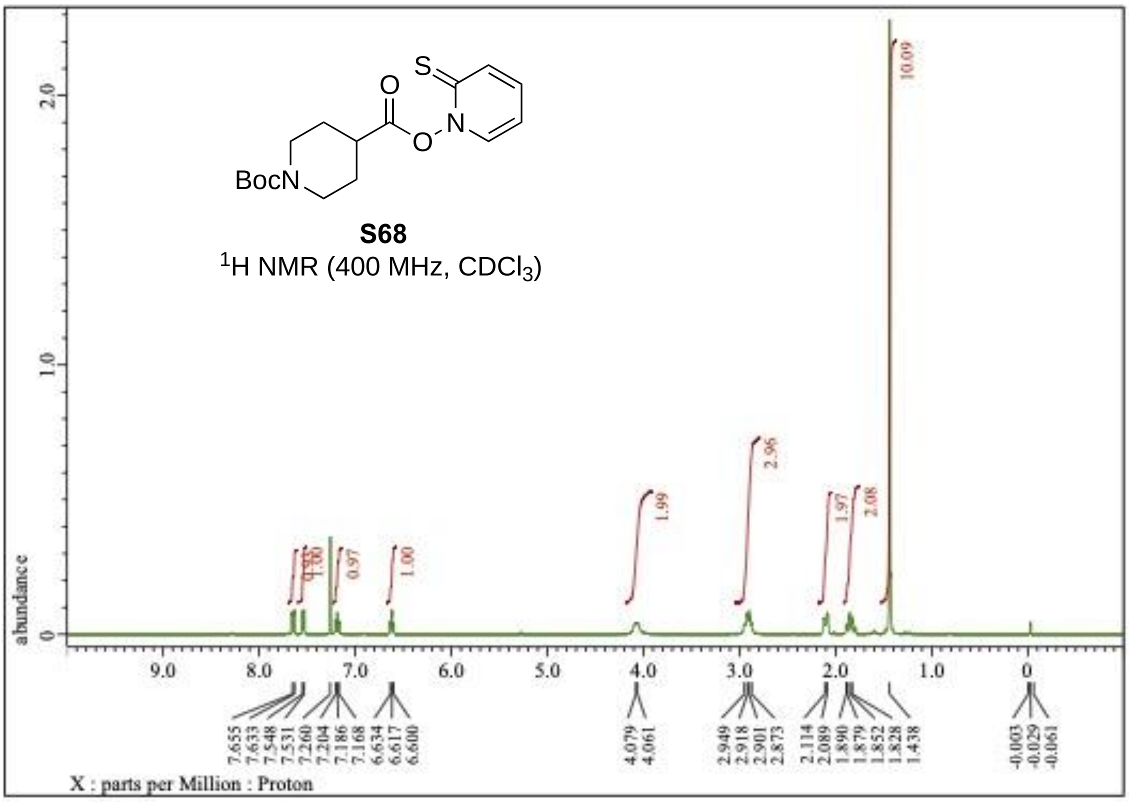
S68
^{1}H NMR (400 MHz, $CDCl_3$)
X : parts per Million : Proton

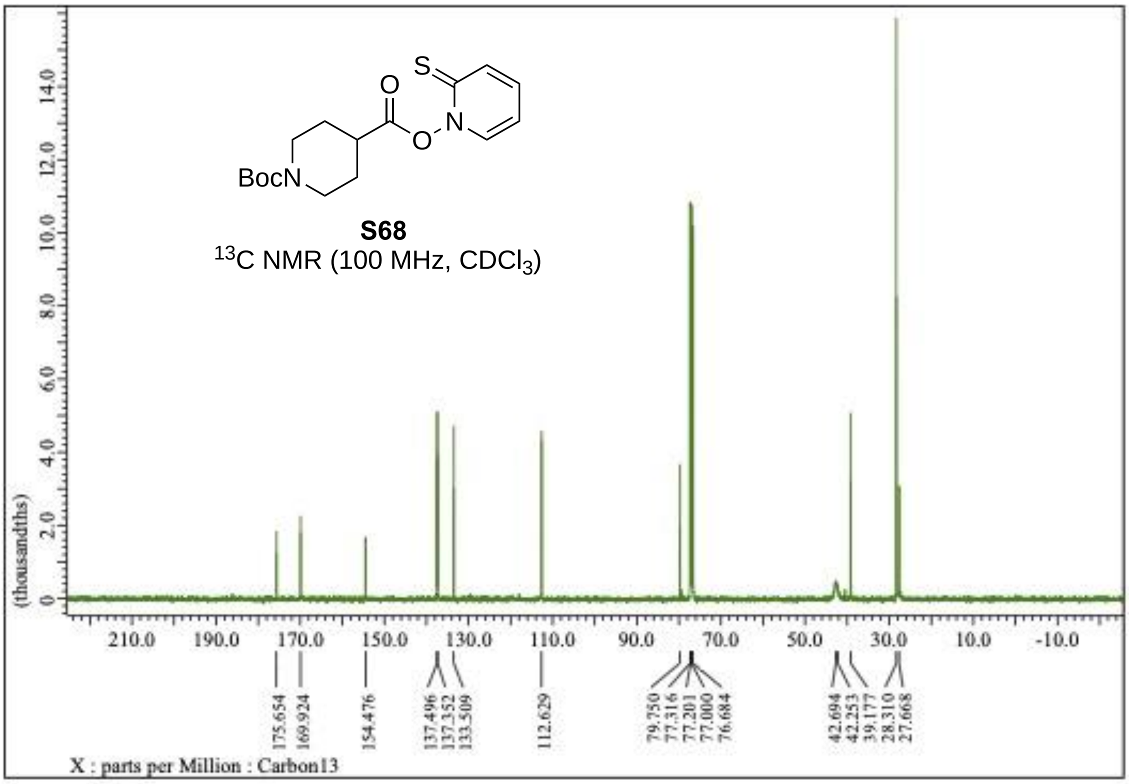
S68
^{13}C NMR (100 MHz, $CDCl_3$)
X : parts per Million : Carbon13

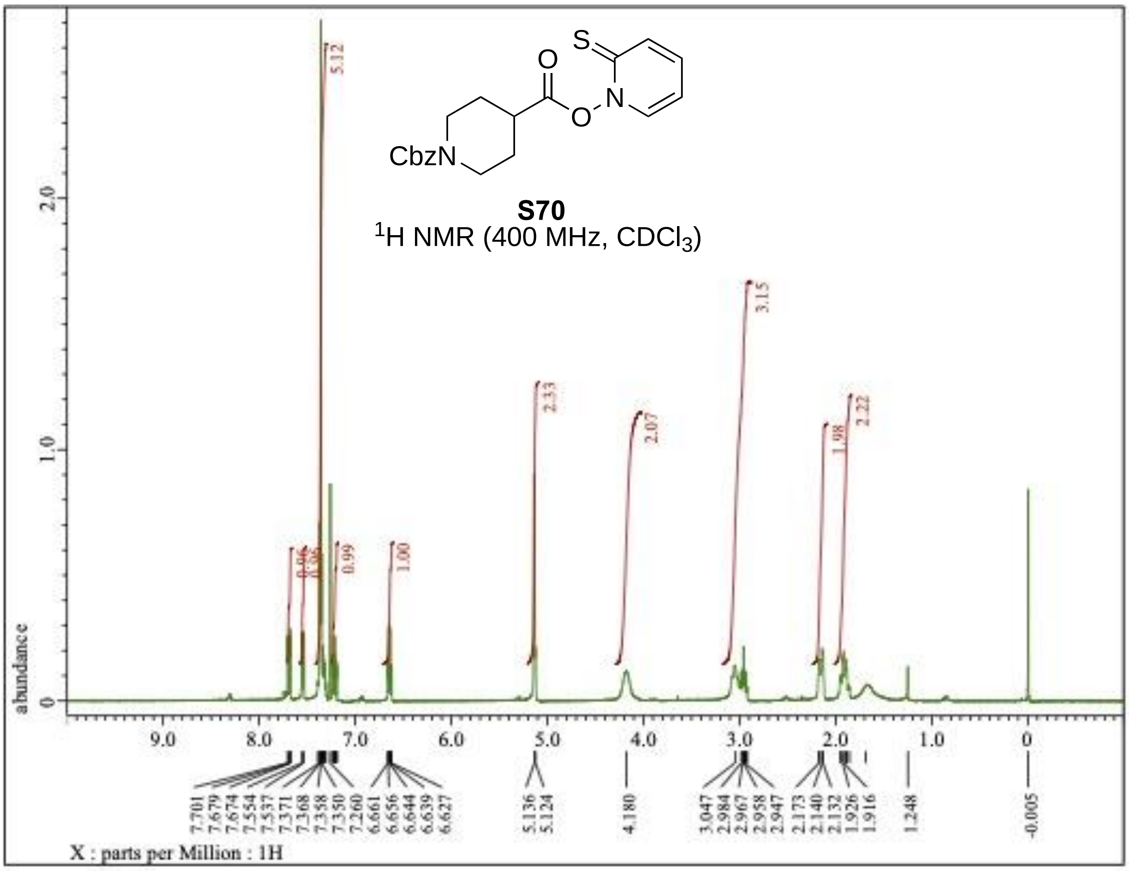
S70
^{1}H NMR (400 MHz, CDCl$_3$)
X : parts per Million : 1H
abundance

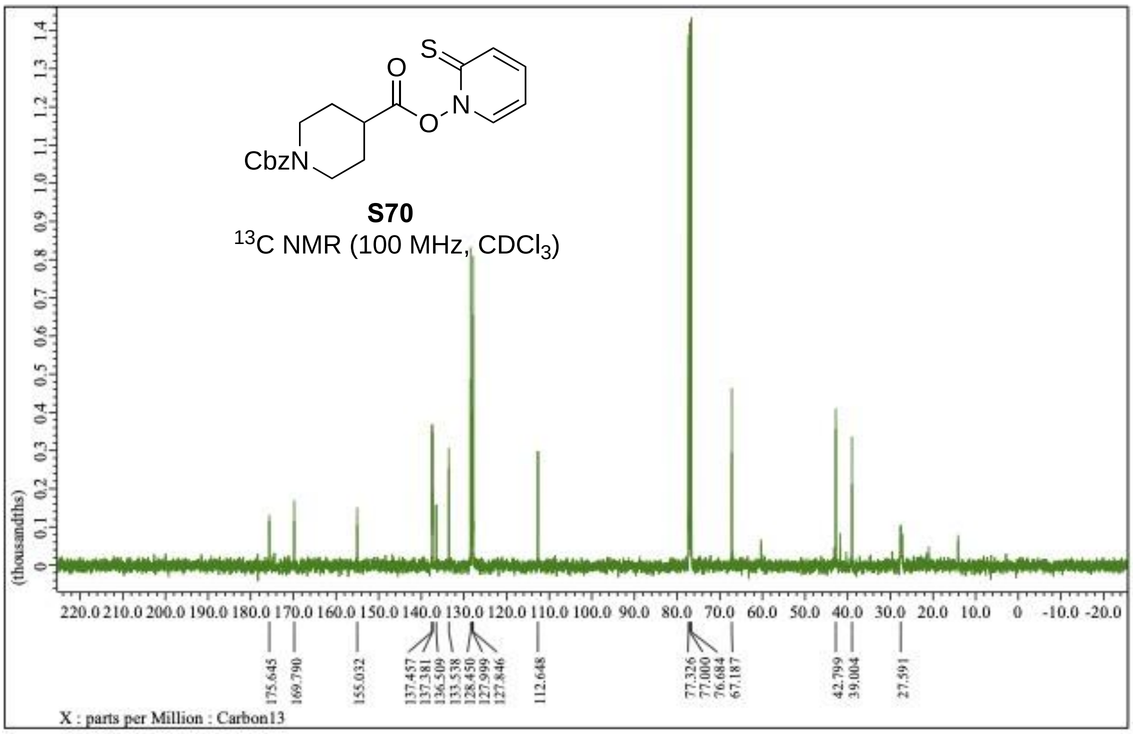
S70
^{13}C NMR (100 MHz, CDCl$_3$)
X : parts per Million : Carbon13
(thousandths)

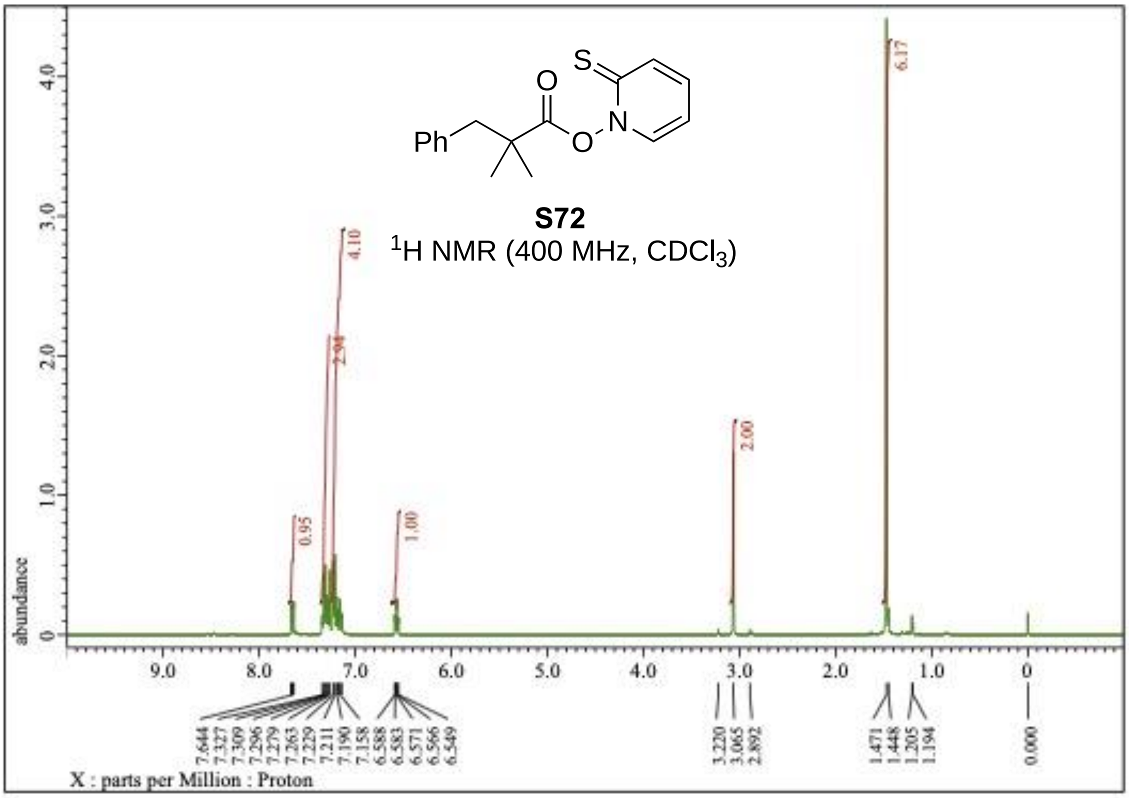
S72
^{1}H NMR (400 MHz, $CDCl_3$)
Ph
O
S
N
abundance
0.95
2.94
4.10
1.00
2.00
6.17
7.644
7.327
7.309
7.296
7.279
7.263
7.229
7.211
7.190
7.158
6.588
6.583
6.571
6.566
6.549
3.220
3.065
2.892
1.471
1.448
1.205
1.194
0.000
X : parts per Million : Proton

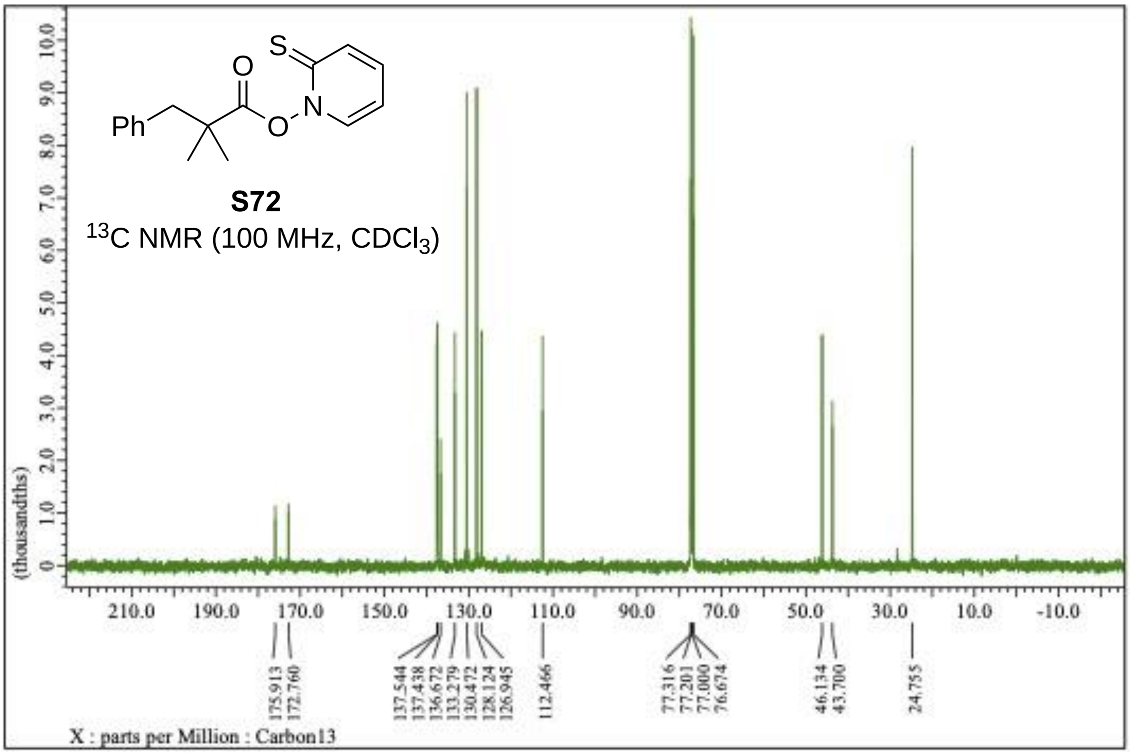
S72
^{13}C NMR (100 MHz, $CDCl_3$)
Ph
O
S
N
(thousandths)
175.913
172.760
137.544
137.438
136.672
133.279
130.472
128.124
126.945
112.466
77.316
77.201
77.000
76.674
46.134
43.700
24.755
X : parts per Million : Carbon13

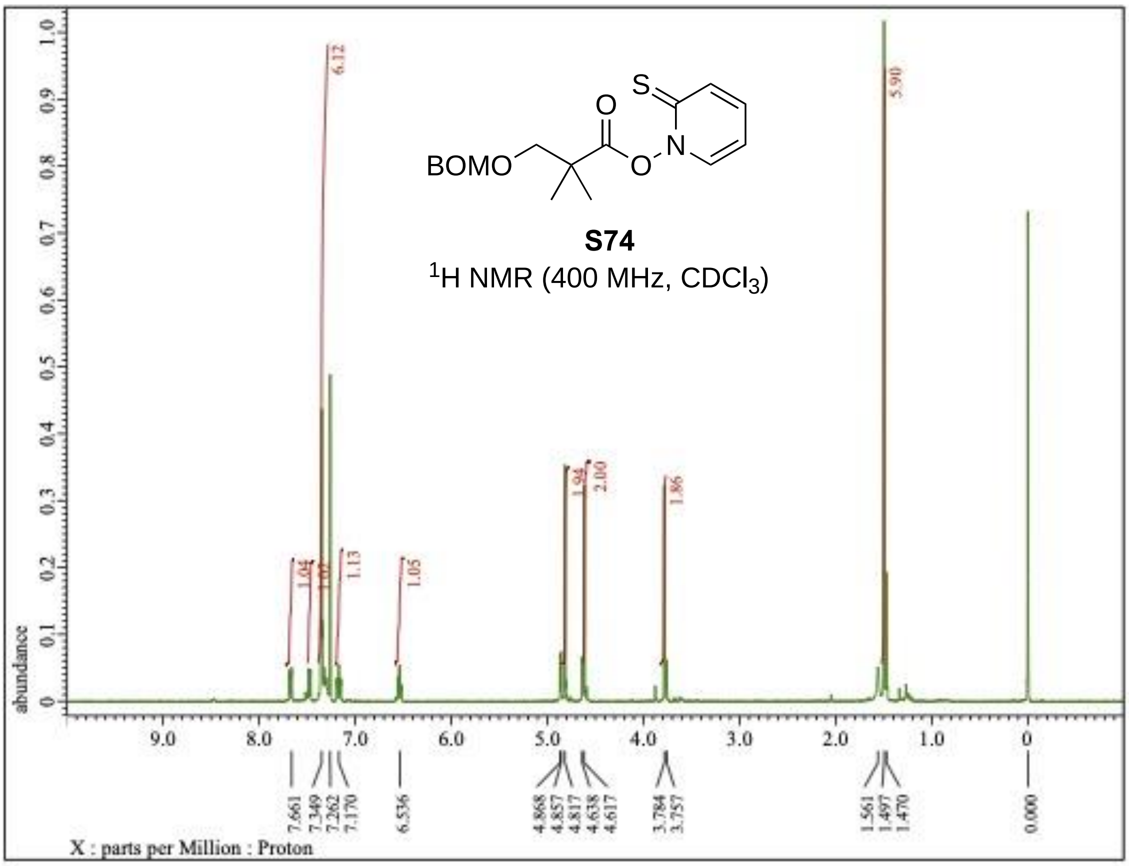
BOMO
S74
^{1}H NMR (400 MHz, $CDCl_3$)
X : parts per Million : Proton
abundance

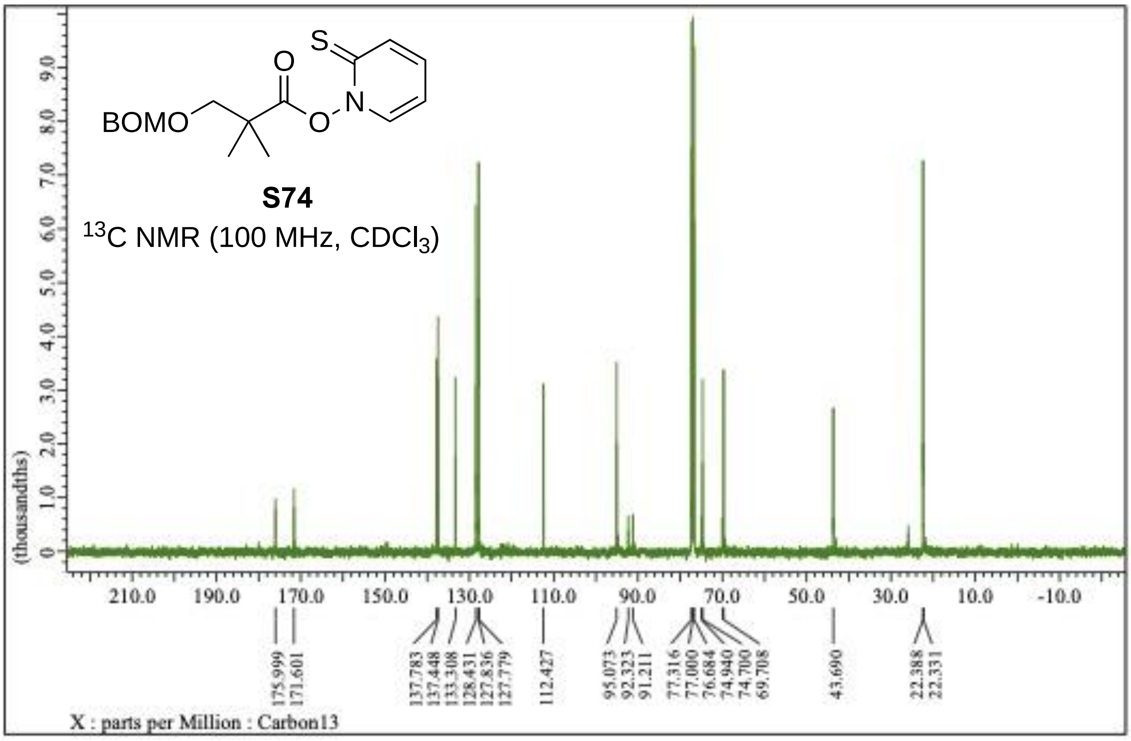
BOMO
S74
^{13}C NMR (100 MHz, $CDCl_3$)
X : parts per Million : Carbon13
(thousandths)

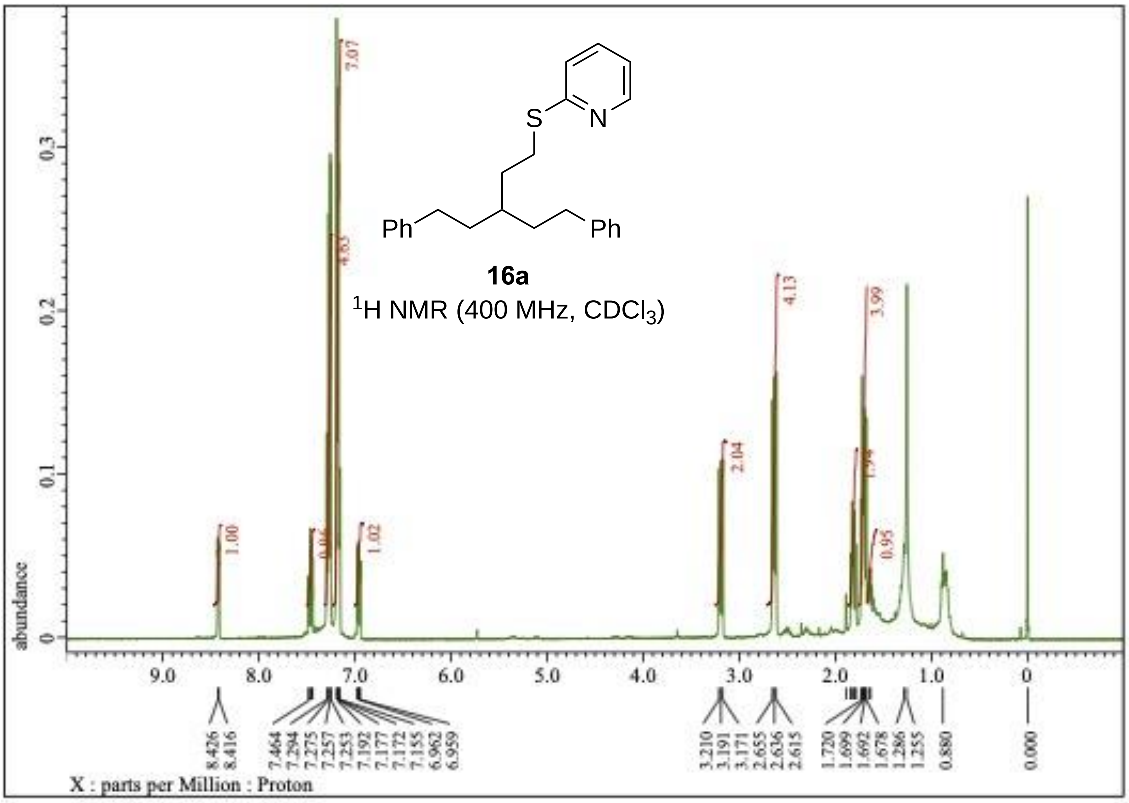

16a
^{1}H NMR (400 MHz, $CDCl_3$)
X : parts per Million : Proton
abundance

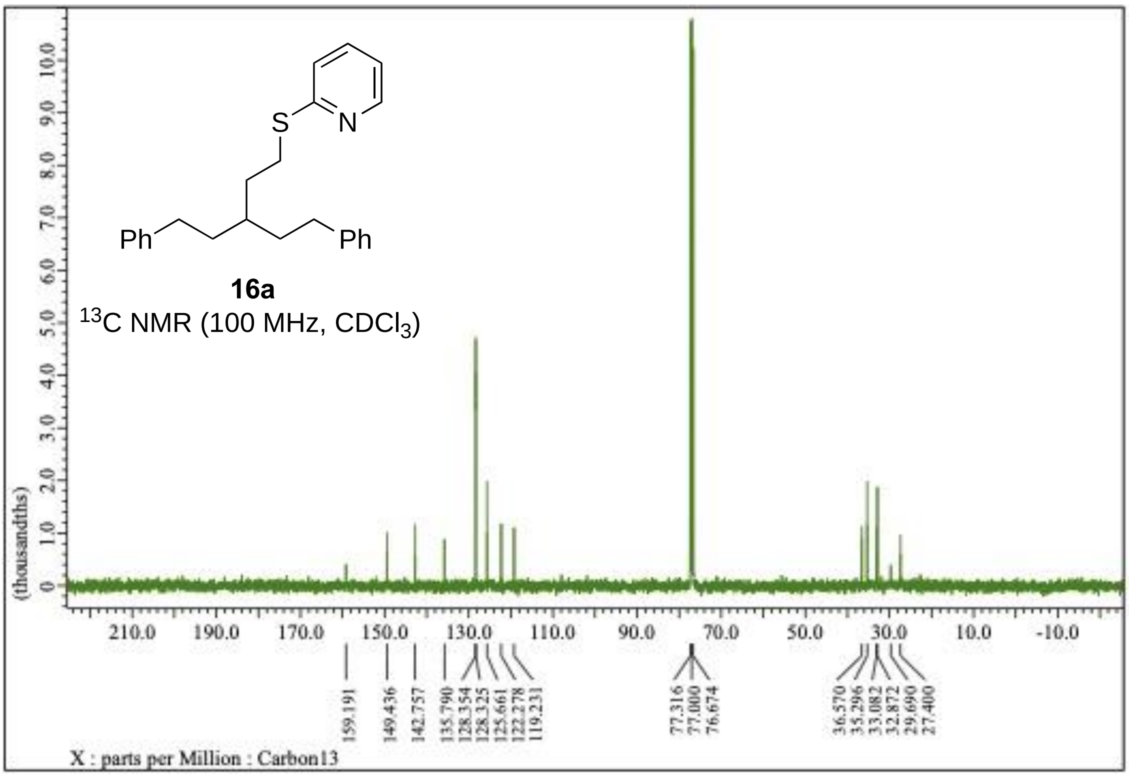

16a
^{13}C NMR (100 MHz, $CDCl_3$)
X : parts per Million : Carbon13
(thousandths)

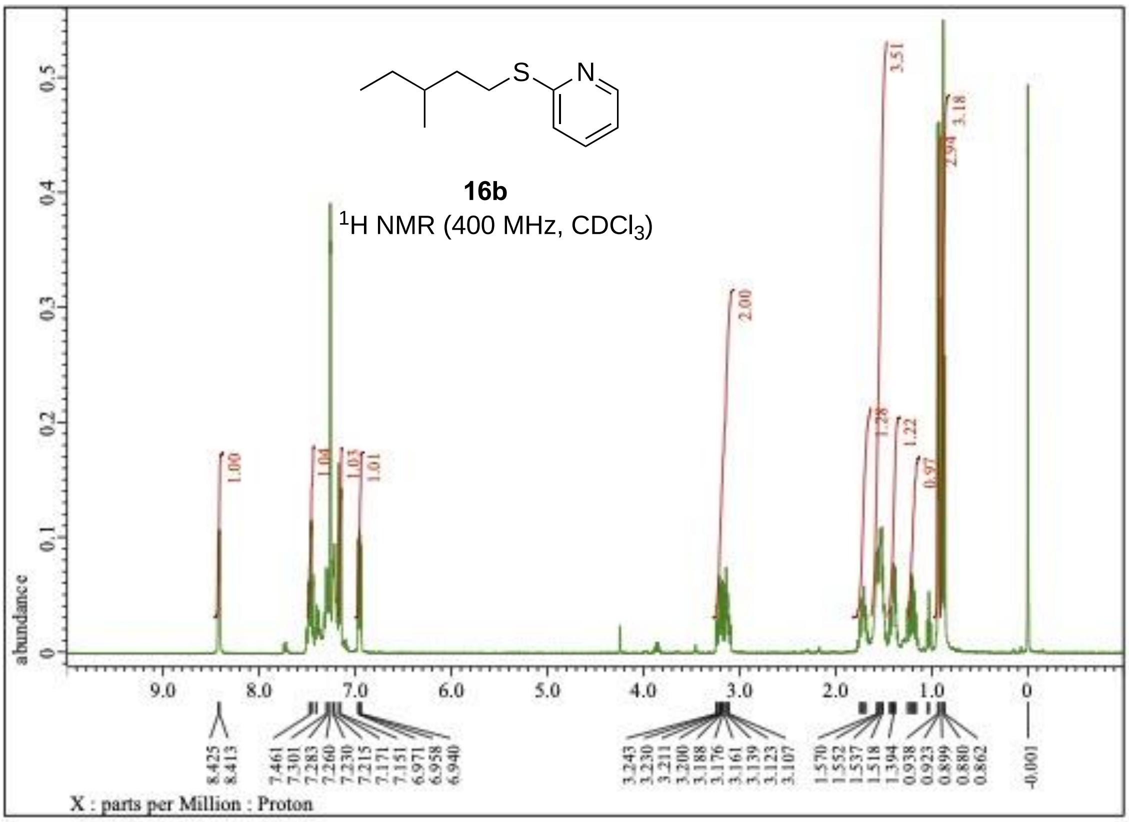
S
N
16b
^{1}H NMR (400 MHz, $CDCl_3$)
abundance
X : parts per Million : Proton
8.425
8.413
7.461
7.301
7.283
7.260
7.230
7.215
7.171
7.151
6.971
6.958
6.940
3.243
3.230
3.211
3.200
3.188
3.176
3.161
3.139
3.123
3.107
1.570
1.552
1.537
1.518
1.394
0.938
0.923
0.899
0.880
0.862
-0.001

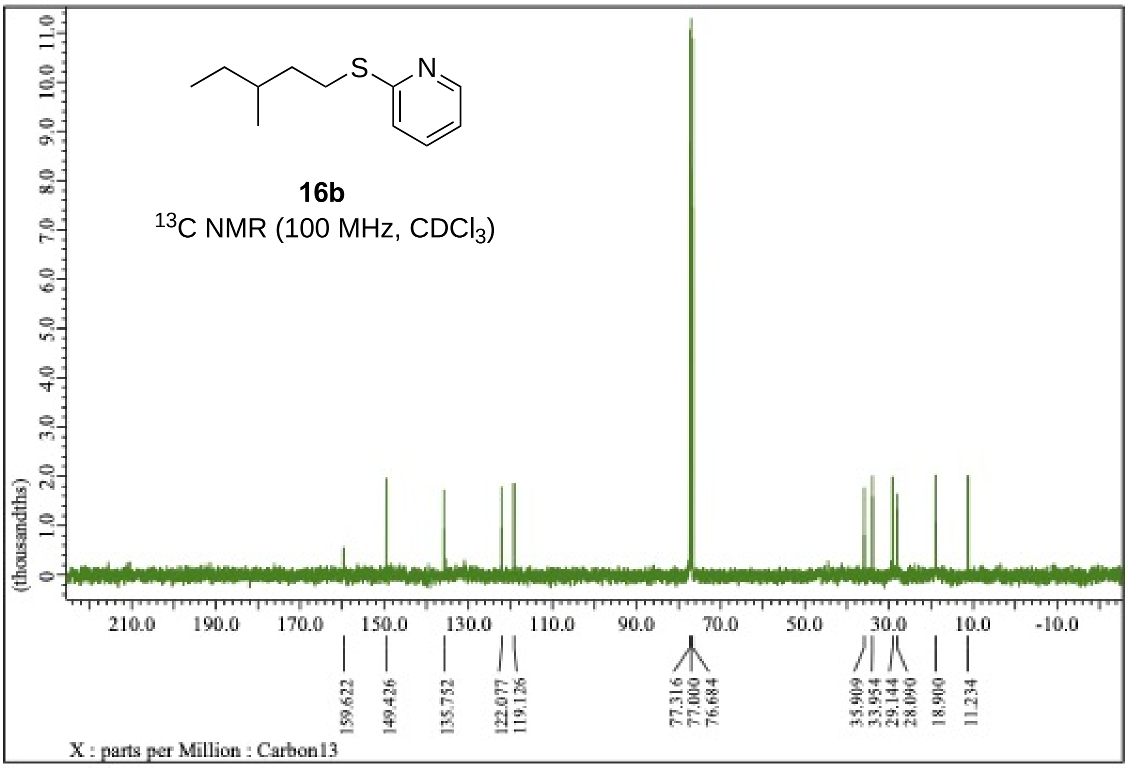
S
N
16b
^{13}C NMR (100 MHz, $CDCl_3$)
(thousandths)
X : parts per Million : Carbon13
159.622
149.426
135.752
122.077
119.126
77.316
77.000
76.684
35.909
33.954
29.144
28.090
18.900
11.234

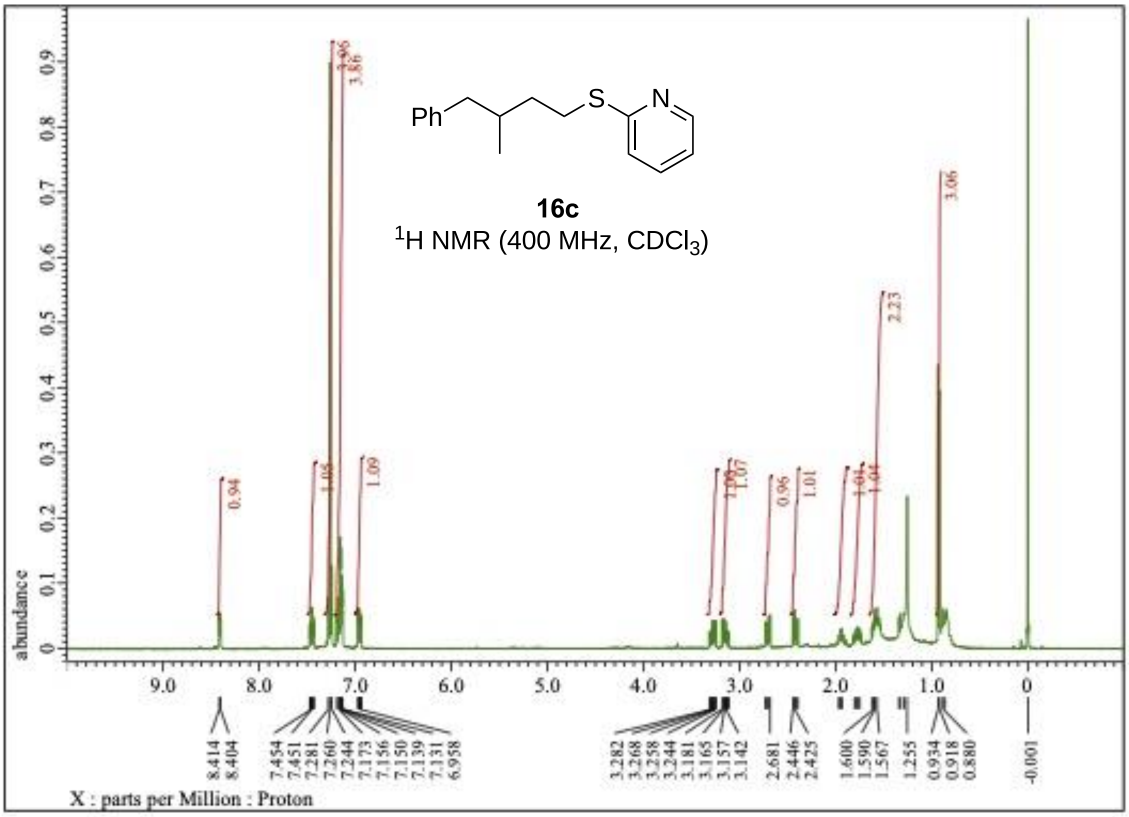

Ph
S
N
16c
1H NMR (400 MHz, CDCl3)
abundance
X : parts per Million : Proton
8.414
8.404
7.454
7.451
7.281
7.260
7.244
7.173
7.156
7.150
7.139
7.131
6.958
3.282
3.268
3.258
3.244
3.181
3.165
3.157
3.142
2.681
2.446
2.425
1.600
1.590
1.567
1.255
0.934
0.918
0.880
-0.001

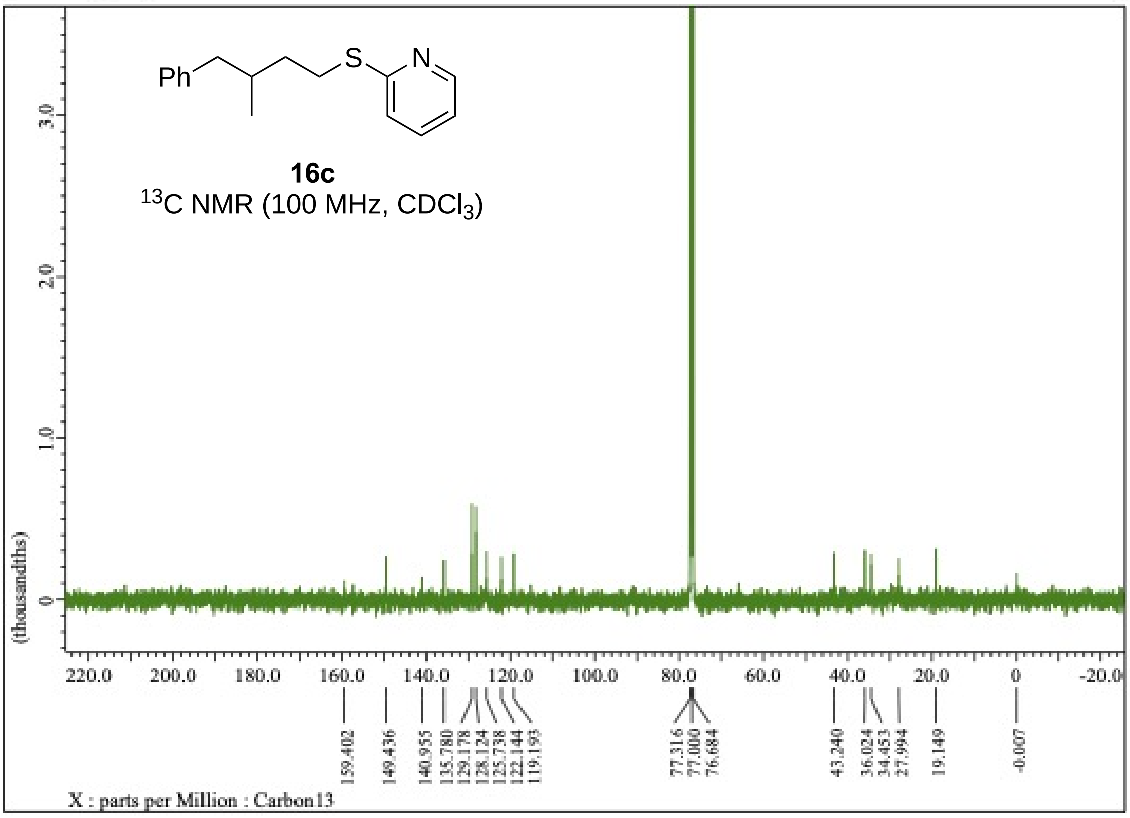

Ph
S
N
16c
13C NMR (100 MHz, CDCl3)
(thousandths)
X : parts per Million : Carbon13
159.402
149.436
140.955
135.780
129.178
128.124
125.738
122.144
119.193
77.316
77.000
76.684
43.240
36.024
34.453
27.994
19.149
-0.007

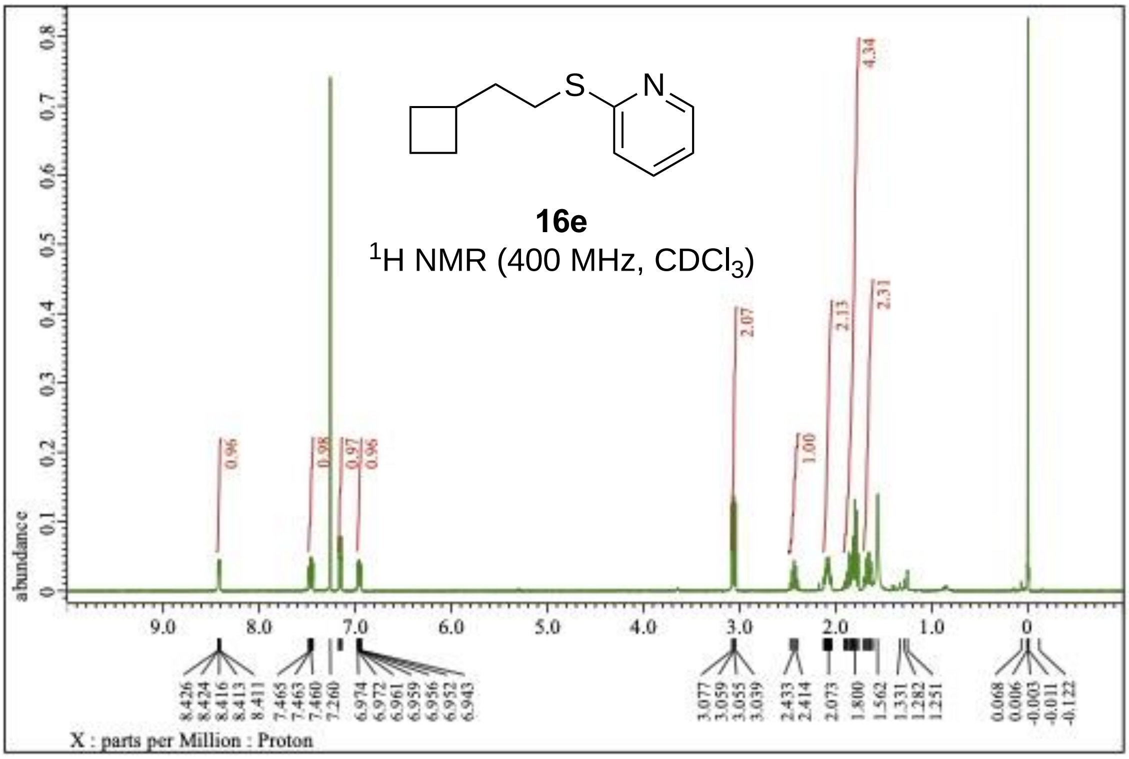
16e
1H NMR (400 MHz, CDCl3)
X : parts per Million : Proton
abundance

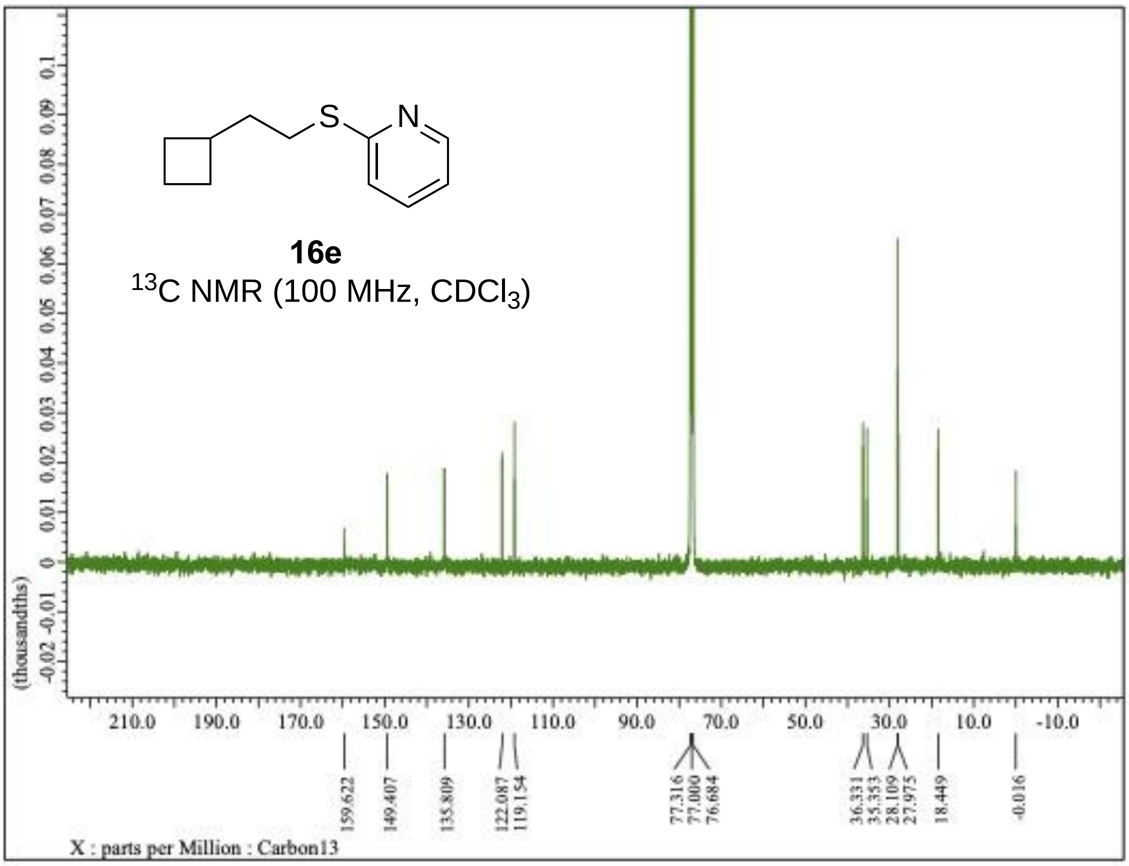
16e
13C NMR (100 MHz, CDCl3)
X : parts per Million : Carbon13
(thousandths)

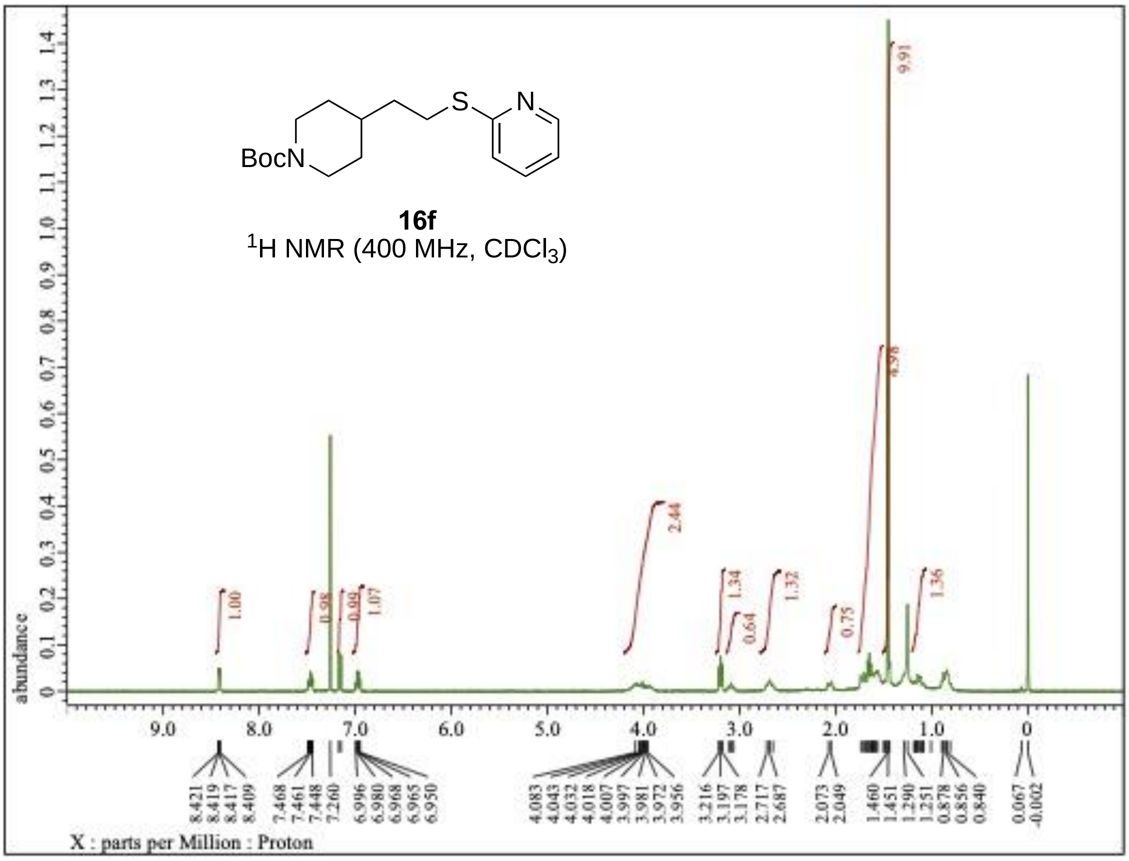
BocN
S
N
16f
1H NMR (400 MHz, CDCl3)
abundance
X : parts per Million : Proton

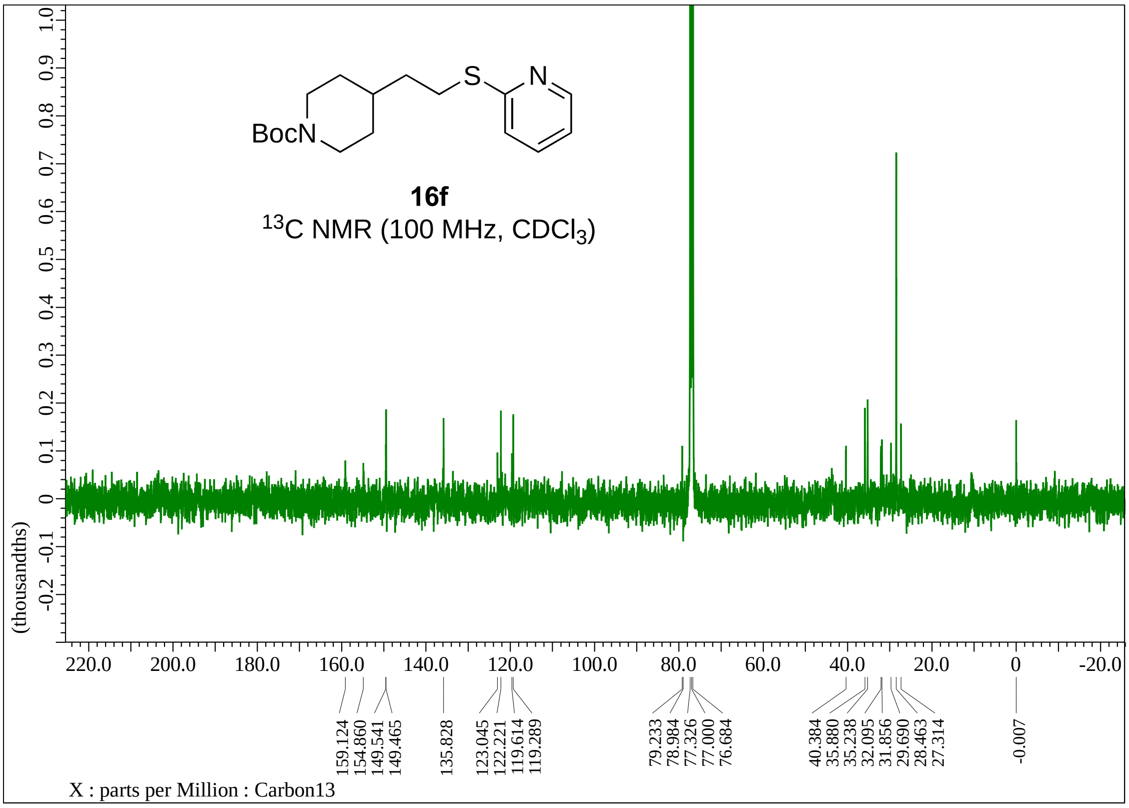
BocN
S
N
16f
13C NMR (100 MHz, CDCl3)
(thousandths)
X : parts per Million : Carbon13

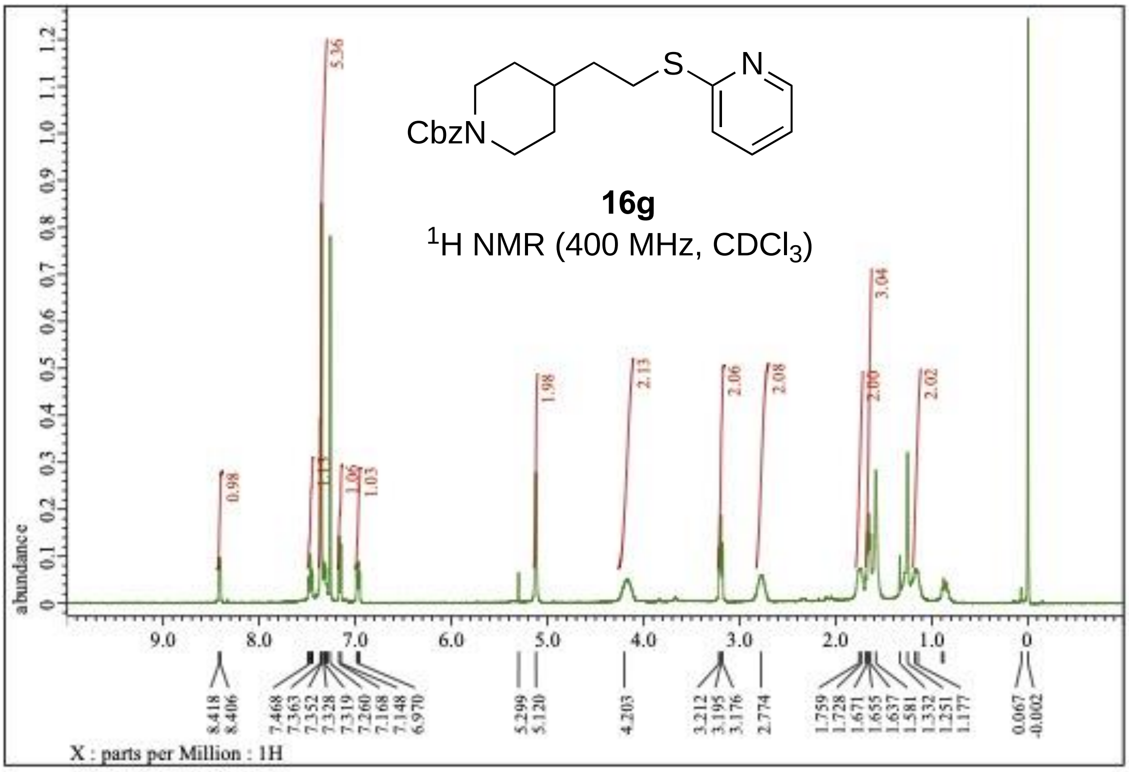

CbzN
S
N
16g
1H NMR (400 MHz, CDCl3)
abundance
X : parts per Million : 1H
8.418
8.406
7.468
7.363
7.352
7.328
7.319
7.260
7.168
7.148
6.970
5.299
5.120
4.203
3.212
3.195
3.176
2.774
1.759
1.728
1.671
1.655
1.637
1.581
1.332
1.251
1.177
0.067
-0.002

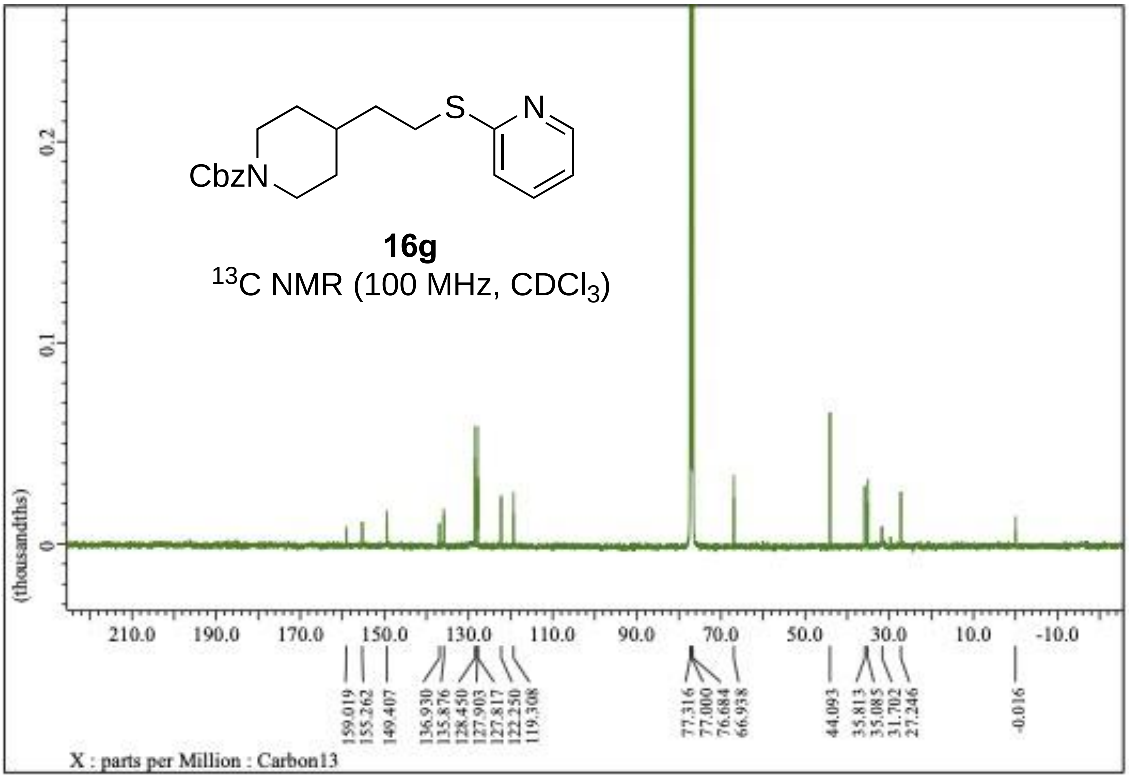

CbzN
S
N
16g
13C NMR (100 MHz, CDCl3)
(thousandths)
X : parts per Million : Carbon13
159.019
155.262
149.407
136.930
135.876
128.450
127.903
127.817
122.250
119.308
77.316
77.000
76.684
66.938
44.093
35.813
35.085
31.702
27.246
-0.016

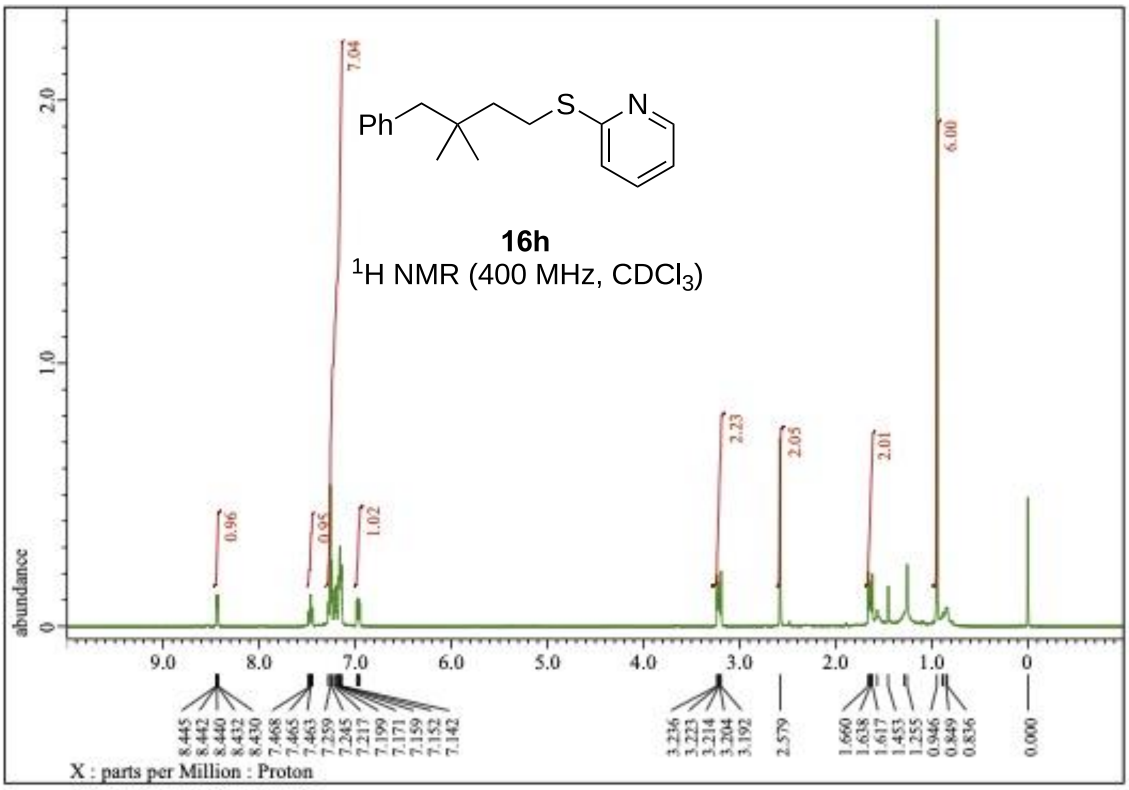
16h
1H NMR (400 MHz, CDCl3)
abundance
X : parts per Million : Proton
0.96
0.95
7.04
1.02
2.23
2.05
2.01
6.00
8.445 8.442 8.440 8.432 8.430 7.468 7.465 7.463 7.259 7.245 7.217 7.199 7.171 7.159 7.152 7.142
3.236 3.223 3.214 3.204 3.192
2.579
1.660 1.638 1.617 1.453 1.255 0.946 0.849 0.836
0.000

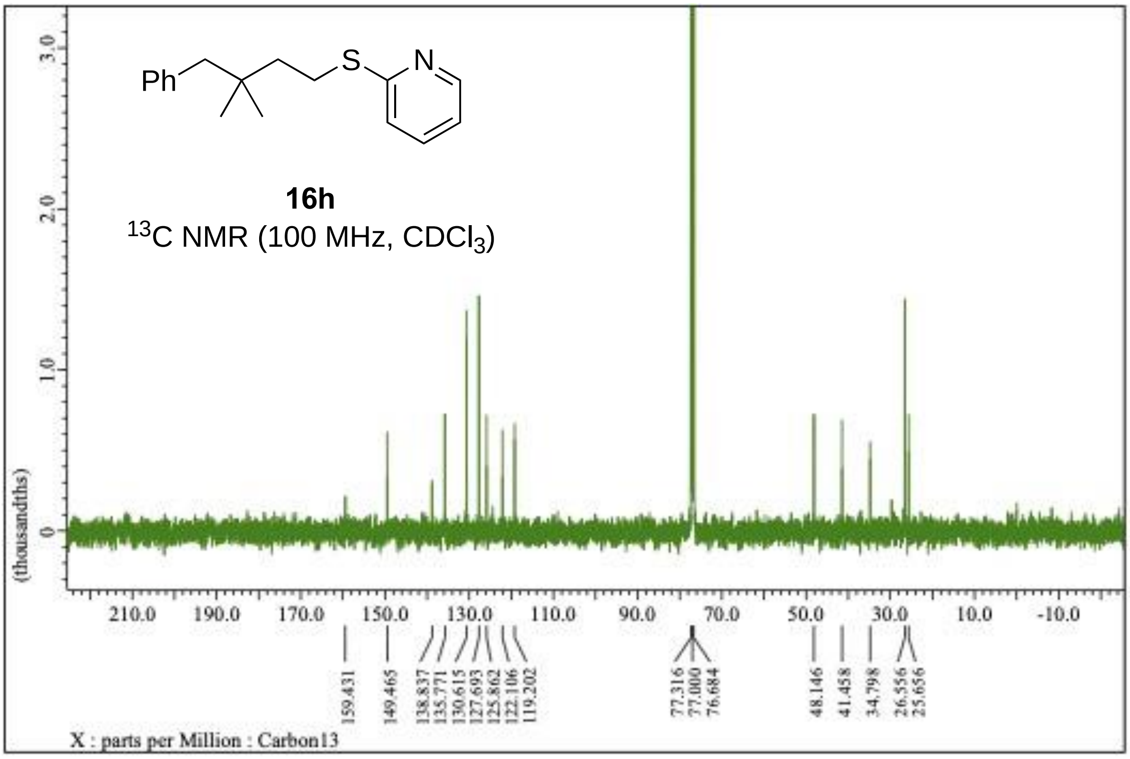
16h
13C NMR (100 MHz, CDCl3)
(thousandths)
X : parts per Million : Carbon13
159.431 149.465 138.837 135.771 130.615 127.693 125.862 122.106 119.202
77.316 77.000 76.684
48.146 41.458 34.798 26.556 25.656

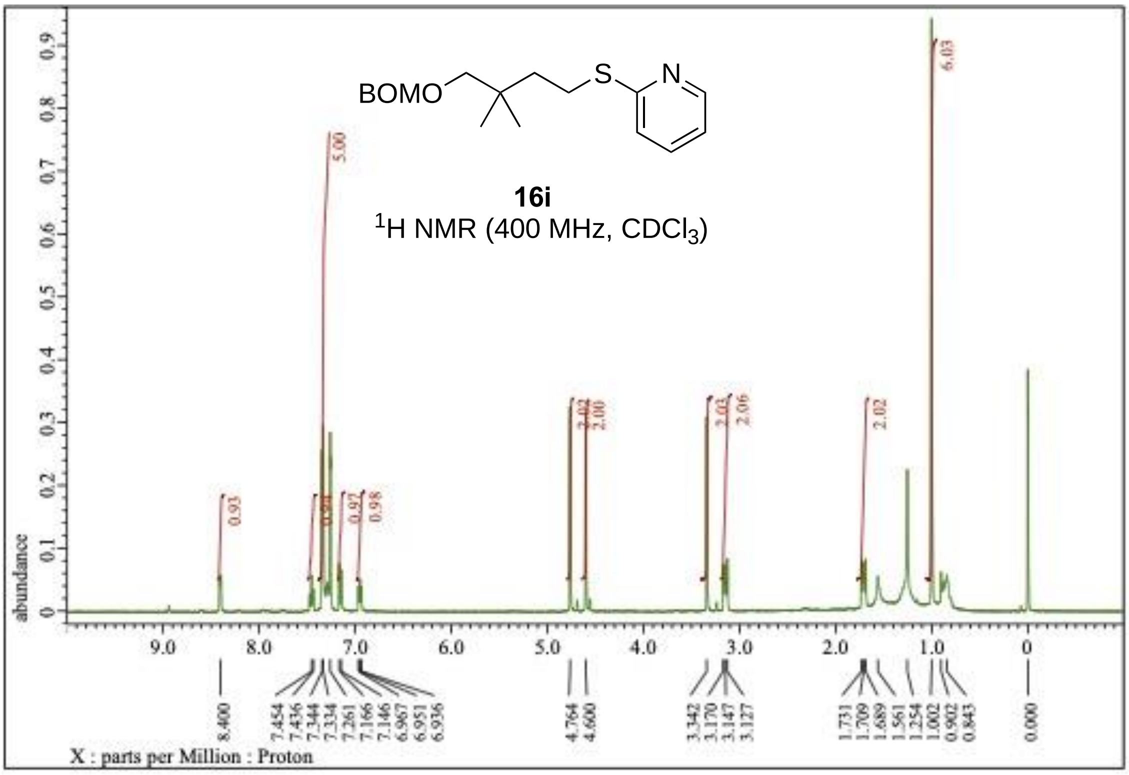
BOMO
S
N
16i
^{1}H NMR (400 MHz, $CDCl_3$)
abundance
X : parts per Million : Proton

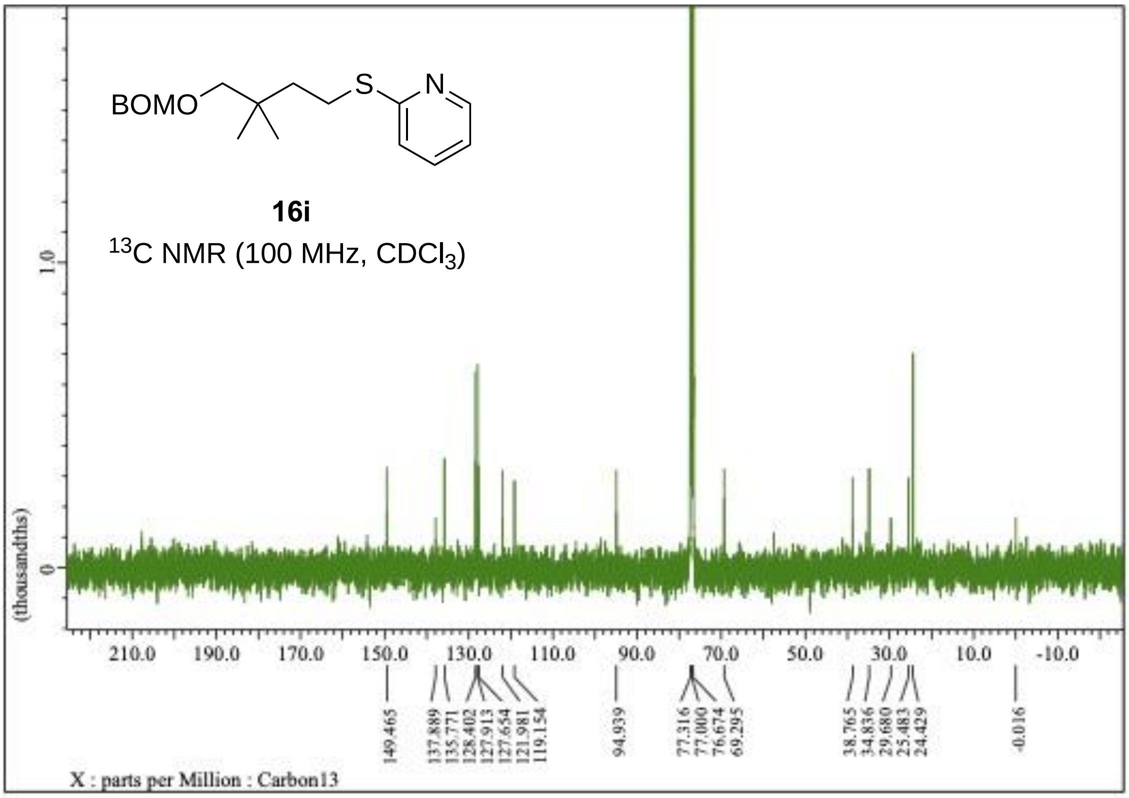
BOMO
S
N
16i
^{13}C NMR (100 MHz, $CDCl_3$)
(thousandths)
X : parts per Million : Carbon13

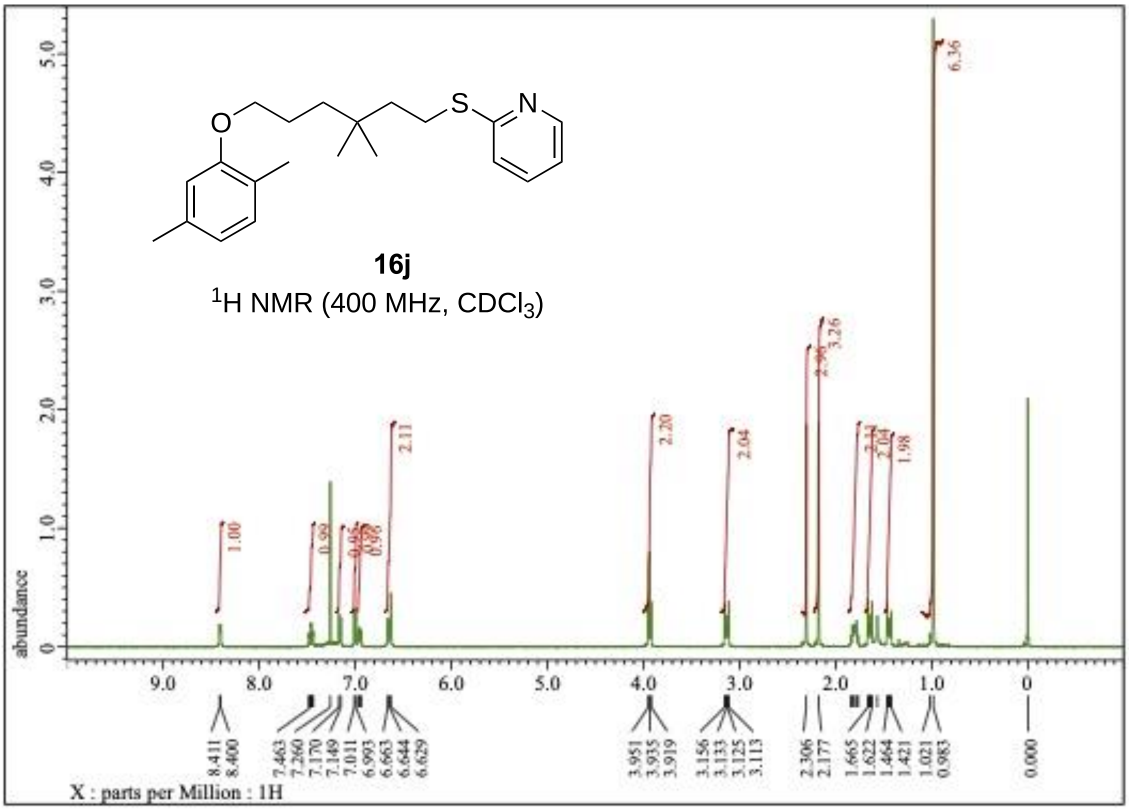

16j
^{1}H NMR (400 MHz, $CDCl_3$)
X : parts per Million : 1H
abundance

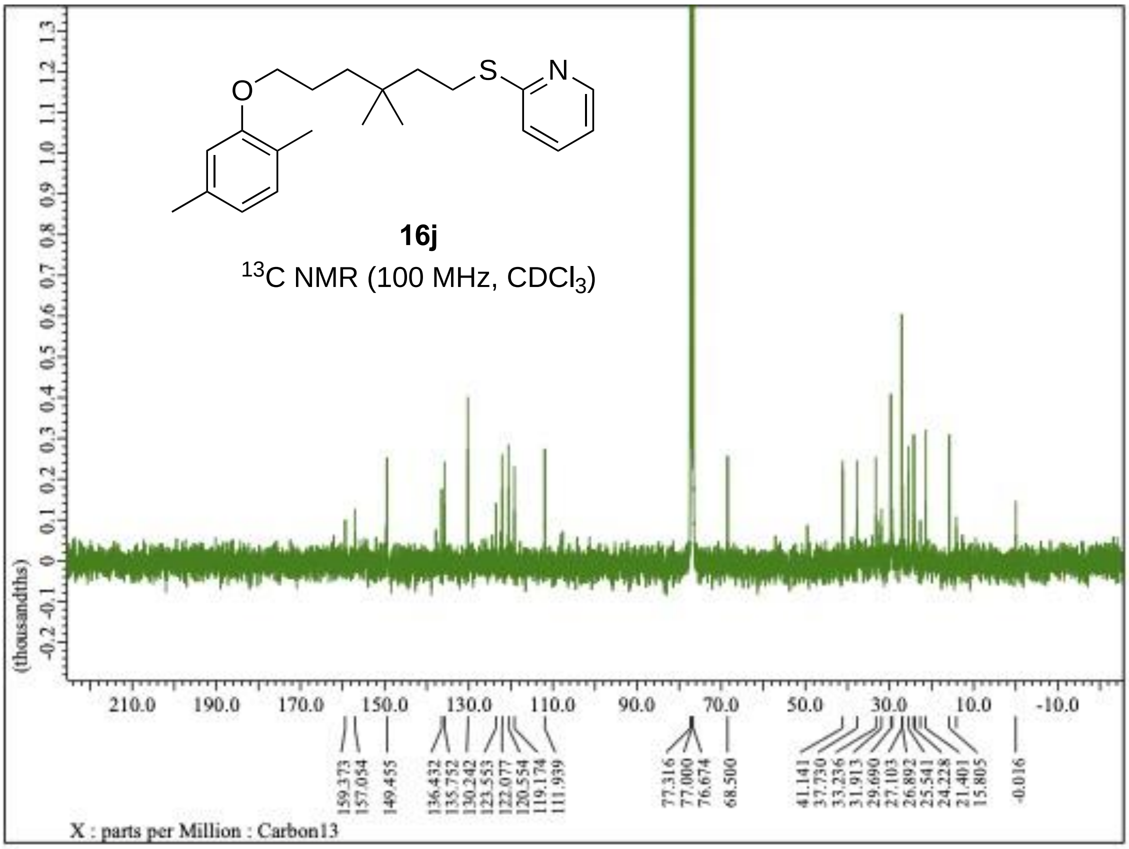

16j
^{13}C NMR (100 MHz, $CDCl_3$)
X : parts per Million : Carbon13
(thousandths)

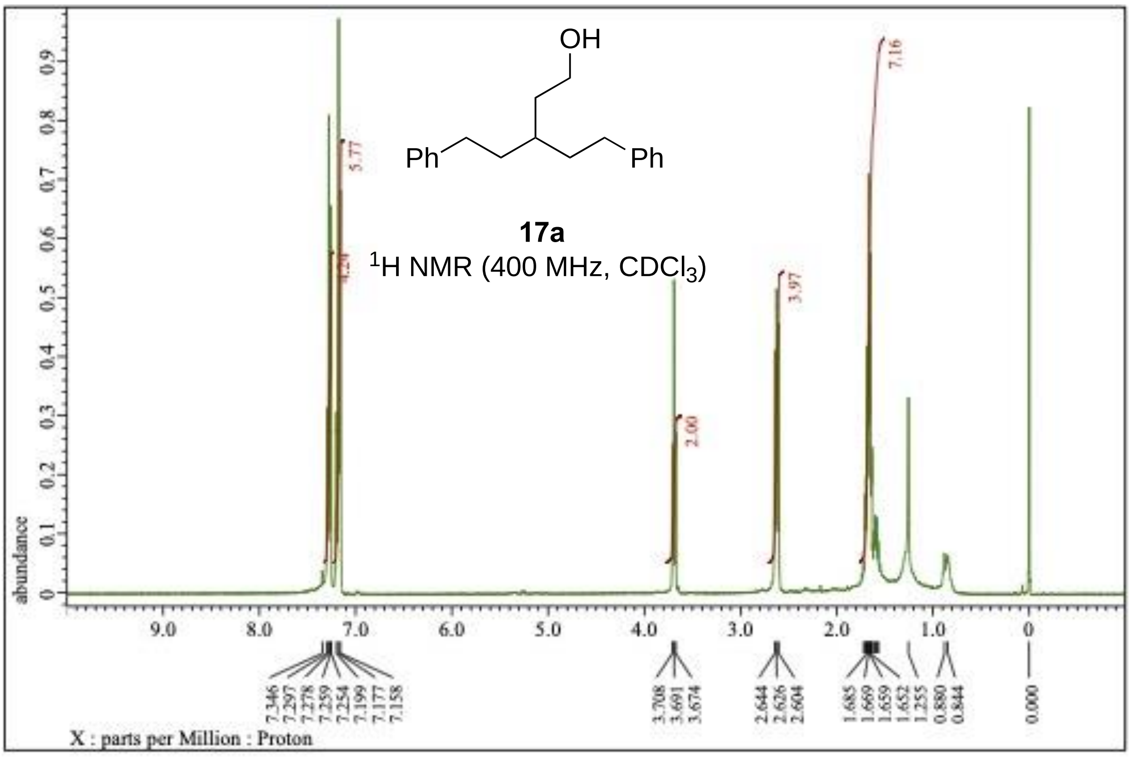
OH
Ph
Ph
17a
1H NMR (400 MHz, CDCl3)
5.77
4.24
2.00
3.97
7.16
abundance
7.346 7.297 7.278 7.259 7.254 7.199 7.177 7.158
3.708 3.691 3.674
2.644 2.626 2.604
1.685 1.669 1.659 1.652 1.255 0.880 0.844
0.000
X : parts per Million : Proton

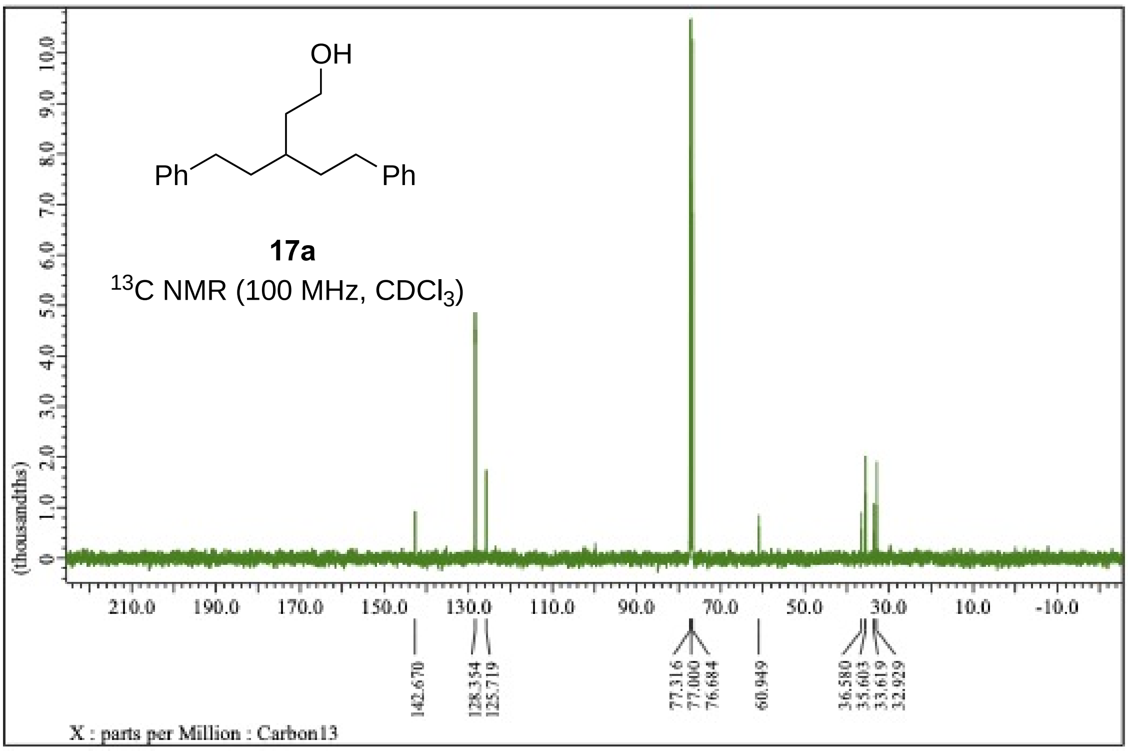
OH
Ph
Ph
17a
13C NMR (100 MHz, CDCl3)
(thousandths)
142.670
128.354 125.719
77.316 77.000 76.684
60.949
36.580 35.603 33.619 32.929
X : parts per Million : Carbon13

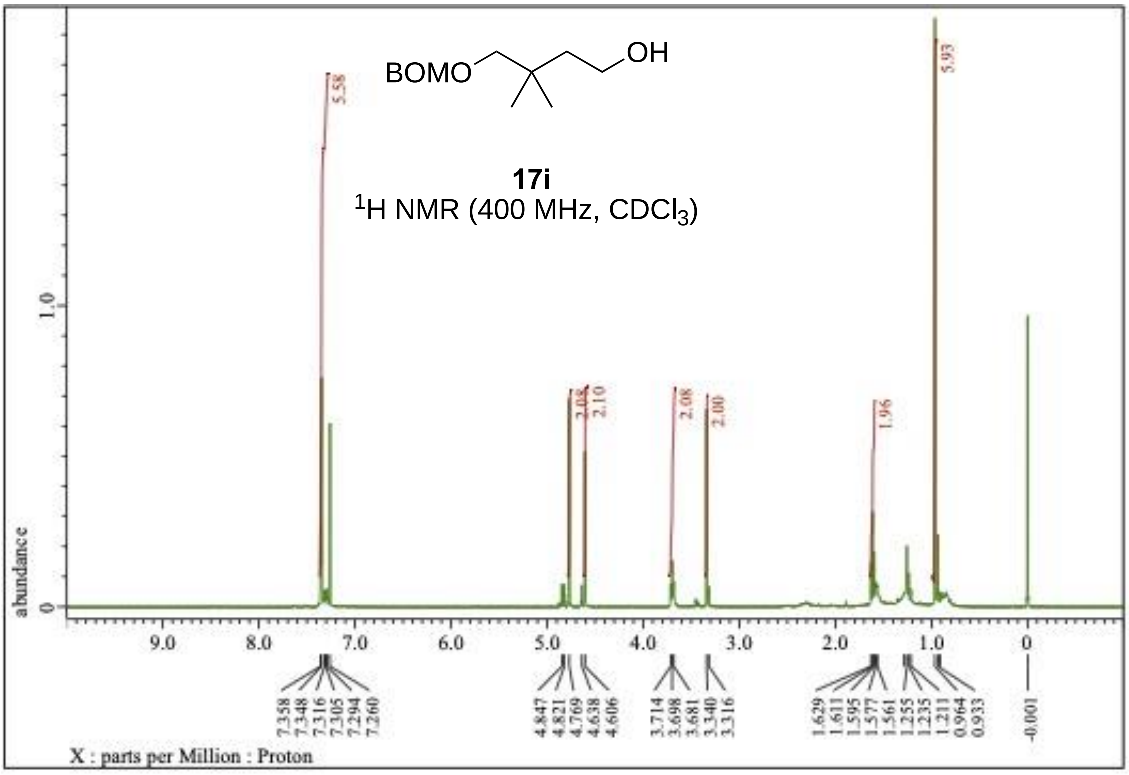
BOMO
OH
17i
1H NMR (400 MHz, CDCl3)
X : parts per Million : Proton
abundance

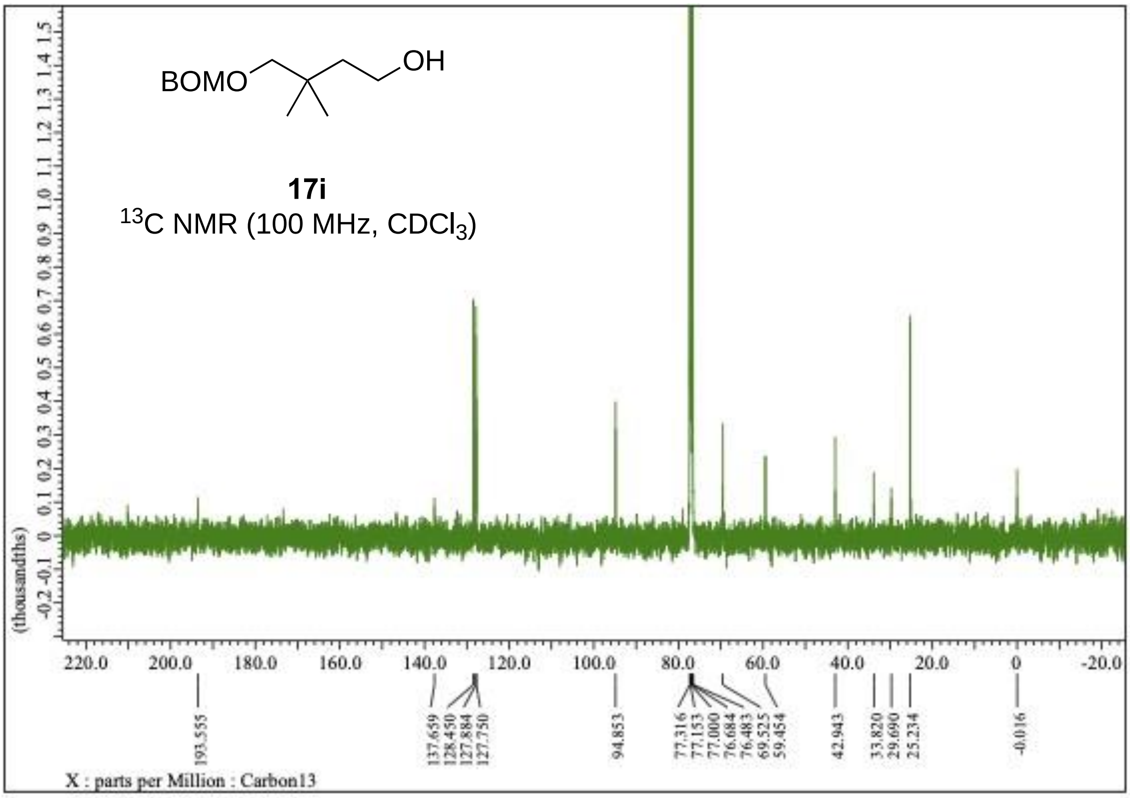
BOMO
OH
17i
13C NMR (100 MHz, CDCl3)
X : parts per Million : Carbon13
(thousandths)

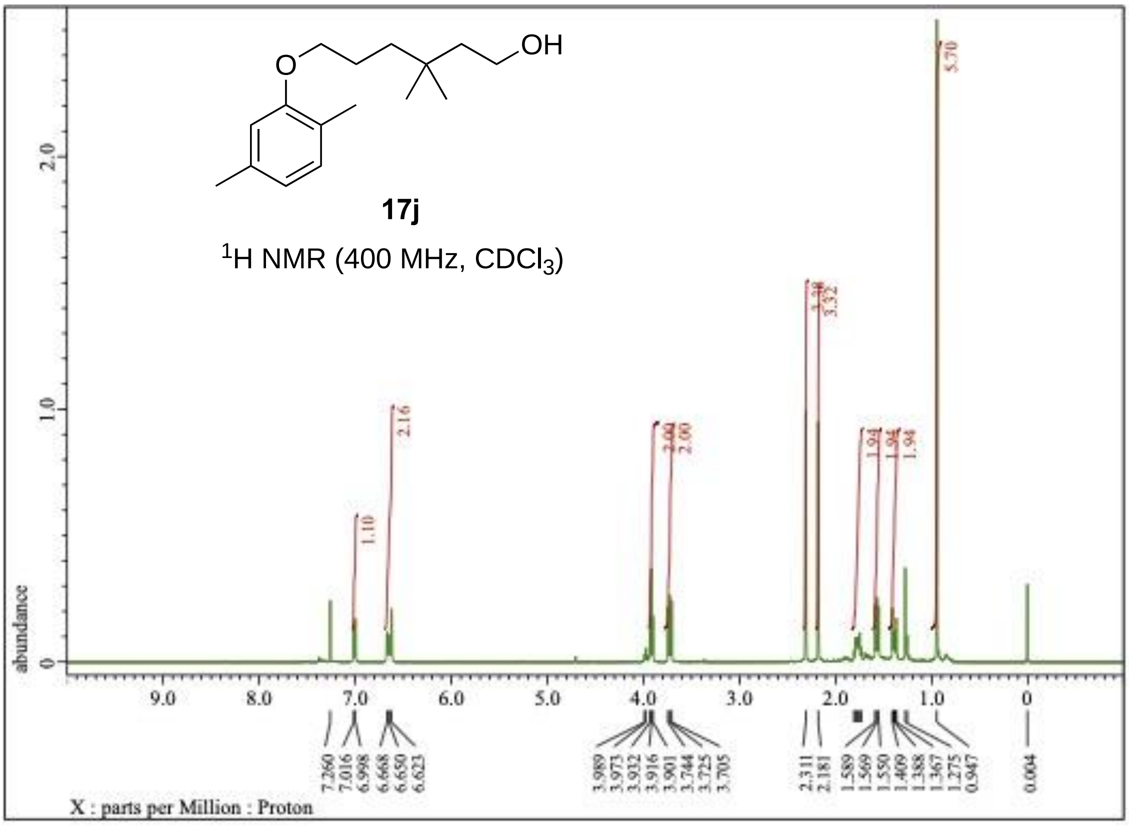
OH
O
17j
1H NMR (400 MHz, CDCl3)
abundance
5.70
3.28
3.32
2.16
1.10
2.02
2.00
1.94
1.94
1.94
9.0 8.0 7.0 6.0 5.0 4.0 3.0 2.0 1.0 0
7.260 7.016 6.998 6.668 6.650 6.623
3.989 3.973 3.932 3.916 3.901 3.744 3.725 3.705
2.311 2.181
1.589 1.569 1.550 1.409 1.388 1.367 1.275 0.947
0.004
X : parts per Million : Proton

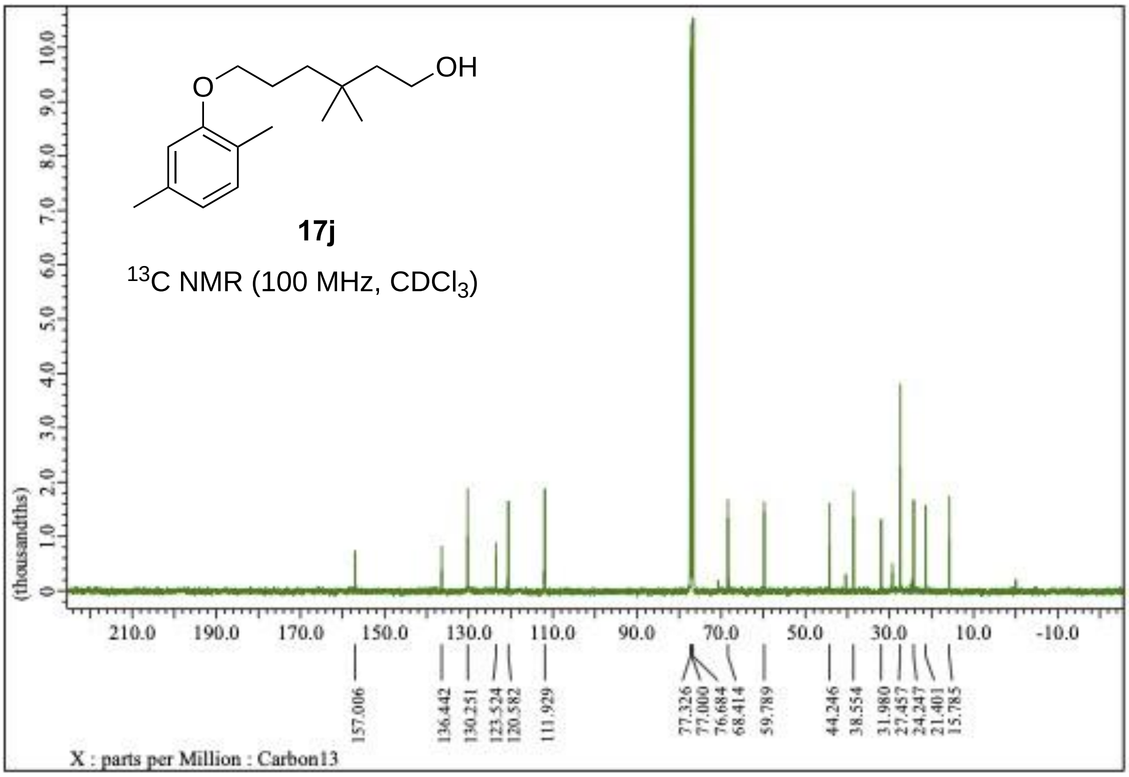
OH
O
17j
13C NMR (100 MHz, CDCl3)
(thousandths)
210.0 190.0 170.0 150.0 130.0 110.0 90.0 70.0 50.0 30.0 10.0 -10.0
157.006
136.442 130.251 123.524 120.582
111.929
77.326 77.000 76.684 68.414
59.789
44.246 38.554 31.980 27.457 24.247 21.401 15.785
X : parts per Million : Carbon13
